# Supplementary material for: Pharmacological targeting of caspase-8/c-FLIPL heterodimer enhances complex II assembly and elimination of pancreatic cancer cells
Source: Commun Biol. 2025 Jan 3;8:4. doi: 10.1038/s42003-024-07409-6 (PMC11698904; doi:10.1038/s42003-024-07409-6)
Supplement: Supplementary file 1 — Supplementary Information [file 42003_2024_7409_MOESM1_ESM.pdf]

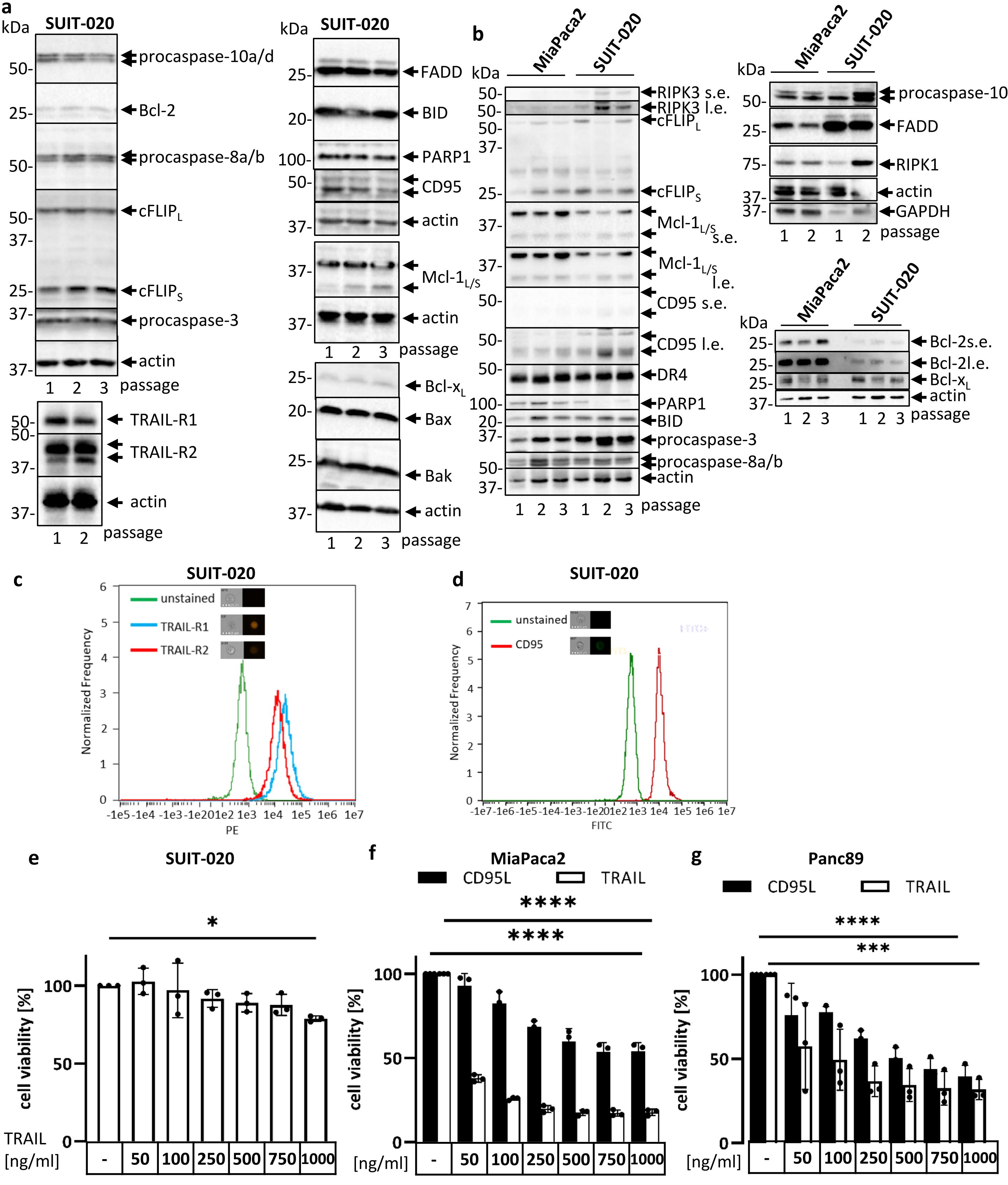

Supplementary figure 1: Checking extrinsic pathway network in SUIT-020, Panc89 and MiaPaca2 cells (**a, b**) Total cell lysates from three or two different passages as indicated of SUIT-020 cells (**a, b**) or MiaPaca2 cells (**b**) were analyzed by Western Blot with the indicated antibodies. Actin served as loading control for each corresponding Western Blot. (**c, d**) SUIT-020 cells were stained with PE anti-human TRAIL antibody (**c**) and FITC anti-human CD95 antibody (**d**). The cells were analyzed and compared with unstained cells using Imaging Flow Cytometry. Representative pictures are shown. **(e-g)** SUIT-020 cells (**e**), MiaPaca2 cells (**f**) or Panc89 cells (**g**) were treated with indicated concentrations of TRAIL (**e-g**) or CD95L (**f, g**) for 22 h. ATP content was measured using the Cell Titer-Glo®-Luminescent Cell Viability Assay. Mean and standard deviation from three independent experiments are shown. For statistical analysis One-way ANOVA tests were used to compare in a group. The following values were used: ****p<0.0001; ***p<0.001; **p<0.01; *p<0.05; ns not significant. Abbreviations: s.e. short exposure, l.e. long exposure

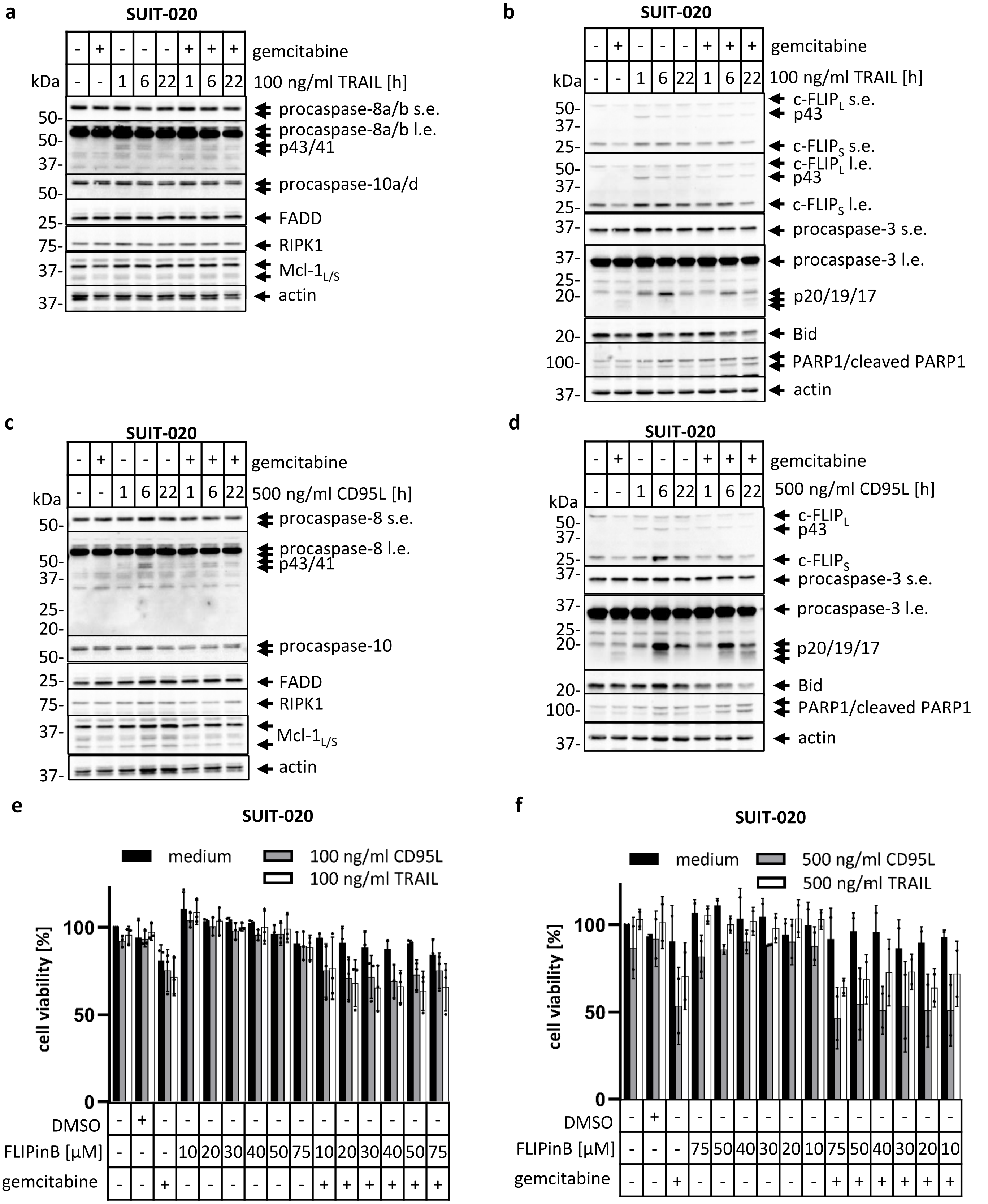

Supplementary figure 2: SUIT-020 cells show minor effects upon DL/gemcitabine and DL/gemcitabine/FLIPinB treatment (**a-d**) SUIT-020 cells were pretreated for 24 h with 10 ng/ml gemcitabine and afterwards with 100 ng/ml TRAIL (**a, b**) or 500 ng/ml CD95L (**c, d**) for 1, 6 or 22 h. Total cell lysates were analyzed by Western Blot with the respective antibodies. Actin served as loading control. One representative Western Blot out of three is shown. (**e, f**) SUIT-020 cells were pretreated with 10 ng/ml gemcitabine for 24 h. Afterwards the cells were stimulated with indicated concentrations of FLIPinB for 2 h. Subsequently, cells were treated for 22 h with 100 ng/ml CD95L and TRAIL (**e**) or 500 ng/ml CD95L and TRAIL (**f**). ATP content was measured using the Cell Titer-Glo®-Luminescent Cell Viability Assay. Mean and standard deviation from three independent experiments are shown. Abbreviations: s.e. short exposure, l.e. long exposure

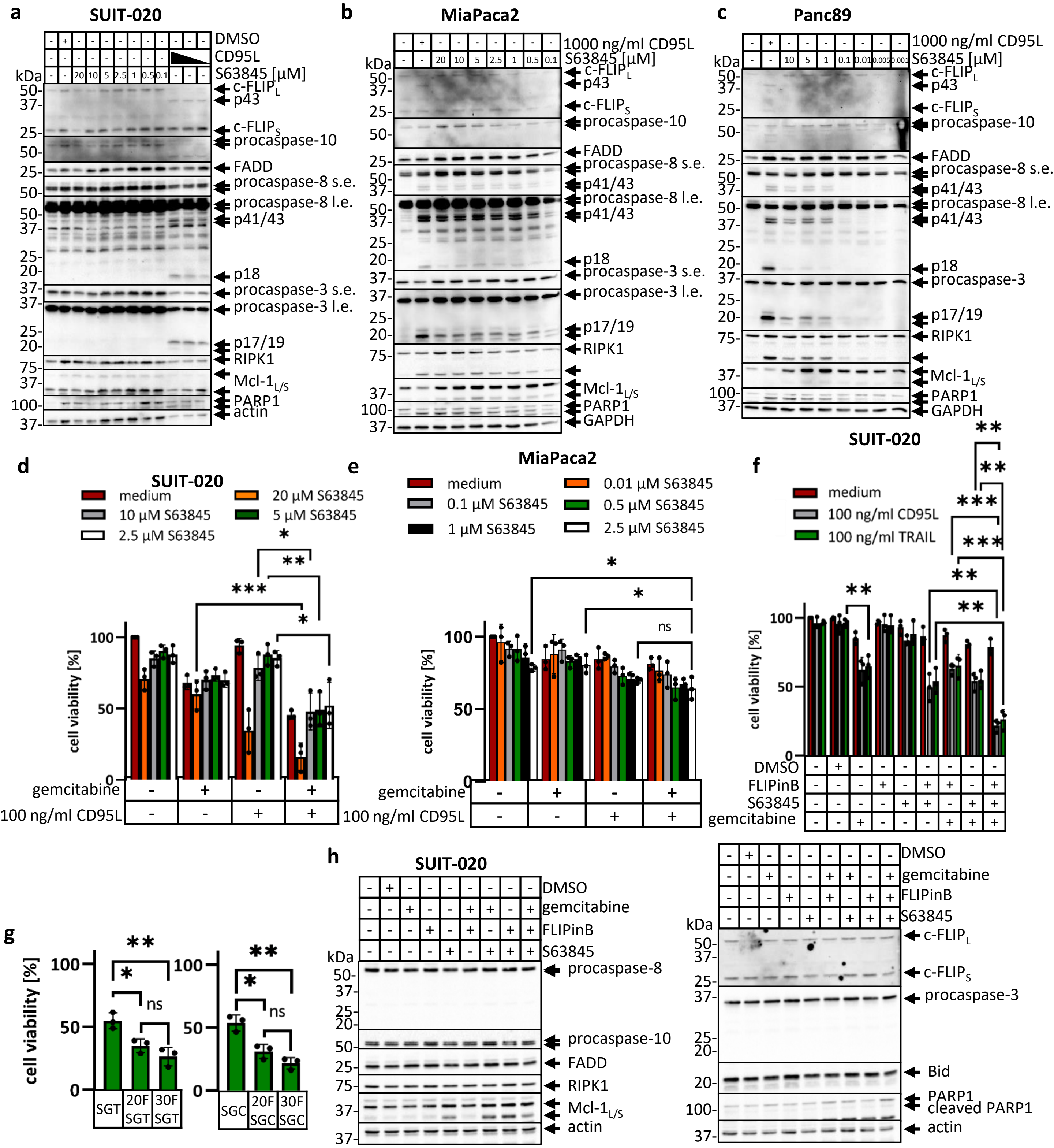

Supplementary figure 3: The analysis of effects of S63845 and FLIPinB in pancreatic cancer cells (a-c) SUIT-020 (**a**), MiaPaca2 (**b**), Panc89 (**c**) cells were treated with indicated concentrations of S63845 for 6 h or CD95L for 3 h (500,1000,2000 ng/ml). Cell lysates were analysed using Western Blot with the indicated antibodies. Actin/GAPDH was used as loading control. One representative Western Blot out of three is shown. (**d, e**) SUIT-020 cells (**d**) or MiaPaca2 cells (**e**) were pretreated with 10 ng/ml gemcitabine for 24 h and 10 µM (**d**) or 2.5 µM (**e**) S63845 and 20 µM FLIPinB for 2 h and afterwards with CD95L for 22 h. (**f**) SUIT-020 cells were pretreated for 24 h with 10 ng/ml gemcitabine, followed by 2 h treatment with 10 µM S63845 and 30 µM FLIPinB. Afterwards, the cells were stimulated with TRAIL or CD95L. ATP content was measured using the Cell Titer-Glo®-Luminescent Cell Viability Assay. Mean and standard deviation from three independent experiments are shown. (**g**) Results from Figure 4a, b and Supplementary Figure 3d, d are shown together for more detailed comparison. (**h**) SUIT-020 cells were pretreated with 10 ng/ml gemcitabine for 24 h and then treated for 2 h with 20 µM FLIPinB and 10 µM S63845. Total cell lysates were analysed using the corresponding antibodies and actin served as loading control. One representative Western Blot from three is shown. For statistical analysis in One-way ANOVA with Tukey post hoc tests were used to compare two conditions. The following values were used: ****p<0.0001; ***p<0.001; **p<0.01; *p<0.05; ns not significant. Abbreviations: SGT/C 10 ng/ml gemcitabine +10 µM S63845+100 ng/ml TRAIL/CD95L, 20FSGT/C 10 ng/ml gemcitabine +20 µM FLIPinB +10 µM S63845+100 ng/ml TRAIL/CD95L, 30FSGT/C 10 ng/ml gemcitabine +30 µM FLIPinB +10 µM S63845+100 ng/ml TRAIL/CD95L

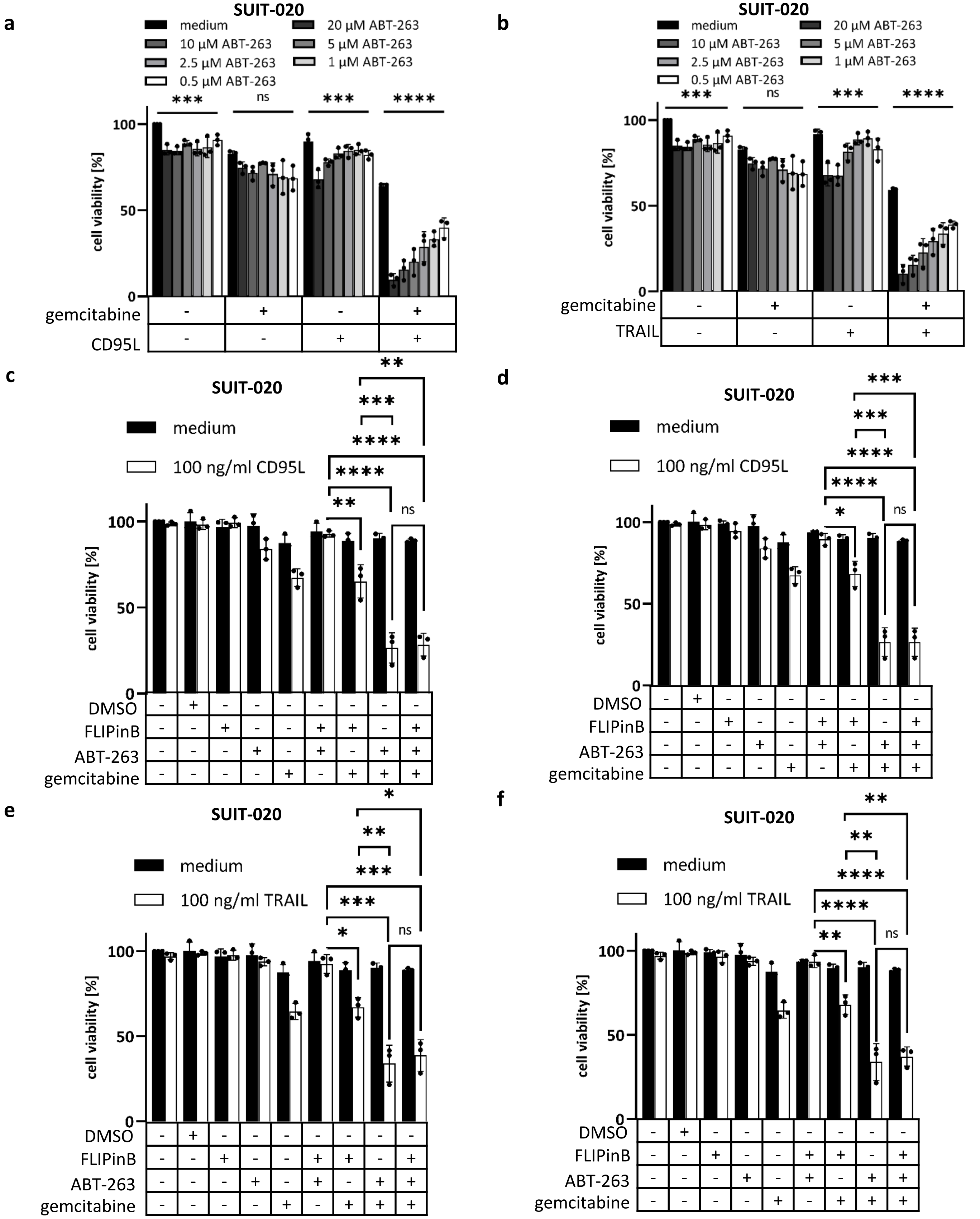

Supplementary figure 4: The analysis of effects of combined treatment with DL, gemcitabine, FLIPinB and ABT-263 in SUIT-020 cells (**a, b**) SUIT-020 cells were pretreated with 10 ng/ml gemcitabine for 24 h and subsequently with different concentrations of ABT-263 for 2 h. Afterwards cells were treated for 22 h with 100 ng/ml CD95L (**a**) or TRAIL (**b**). (**c-f**) SUIT-0202 cells were pretreated for 24 with 10 ng/ml gemcitabine and for 2 h with 20 µM (**c, e**) or 30 µM (**d, f**) FLIPinB and 1 µM ABT-263. Subsequently, cells were stimulated for 22 h with 100 ng/ml CD95L (**c, d**) or TRAIL (**e, f**). ATP content was measured using the Cell Titer-Glo®-Luminescent Cell Viability Assay. Mean and standard deviation from three independent experiments are shown. For statistical analysis One-way ANOVA tests were used to compare in a group (**a, b**) or One-way ANOVA with Tukey post hoc tests were used to compare two conditions (**c-f**). The following values were used: ****$p<0.0001$; ***$p<0.001$; **$p<0.01$; *$p<0.05$; ns not significant.

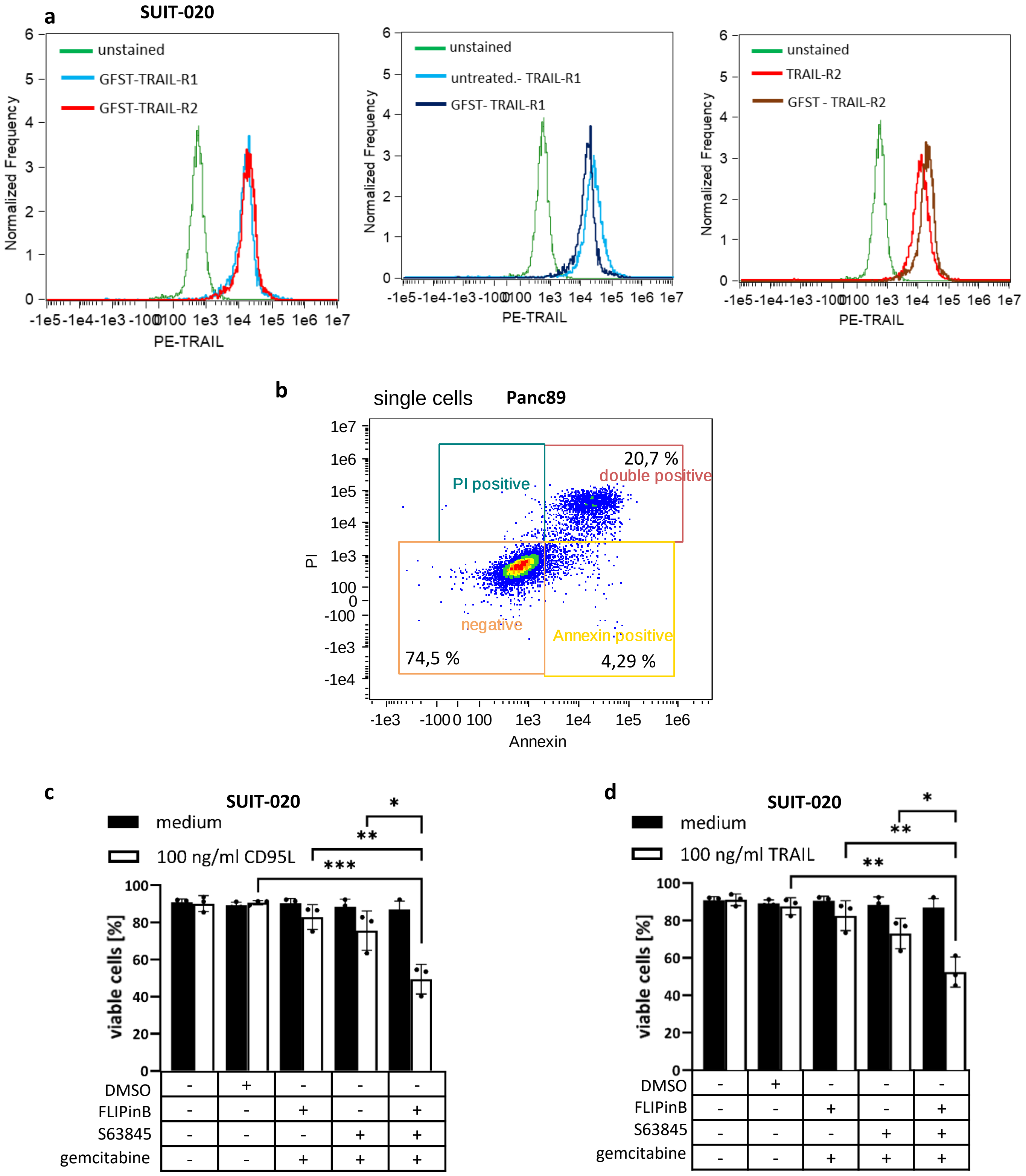

Supplementary Figure 5: The analysis of TRAIL-R expression and cell death in SUIT-020 cells upon DL/gemcitabine/FLIPinB/S63845 treatment (**a**) SUIT-020 cells were pretreated for 24 h with 10 ng/ml gemcitabine. Afterwards the cells were treated for 2 h with 20 µM FLIPinB and 10 µM S63845. Subsequently, the cells were stimulated for 22 h with 100 ng/ml TRAIL. Treated and untreated SUIT-020 cells were stained with PE anti-human TRAIL-R1 or -R2 antibody. The cells were analyzed and compared with unstained cells using Imaging Flow cytometry. Representative pictures are shown. (**b**) Gating strategy for Annexin-V-FITC/PI staining in Panc89 cells. (**c, d**) SUIT-020 cells were treated for 24 h with 10 ng/ml gemcitabine, followed by 2 h treatment with 20 µM FLIPinB and 10 µM S63845 and 22 h CD95L (**b**) or TRAIL (**c**). Viable cells were measured using Imaging Flow cytometry after PI staining. Mean and standard deviation from three independent experiments are shown. For statistical analysis One-way ANOVA with Tukey post hoc tests were used to compare two conditions. The following values were used: ****p<0.0001; ***p<0.001; **p<0.01; *p<0.05; ns not significant. Abbreviations: GFST 10 ng/ml gemcitabine+20 µM FLIPinB+10 µM S63845+100 ng/ml TRAIL

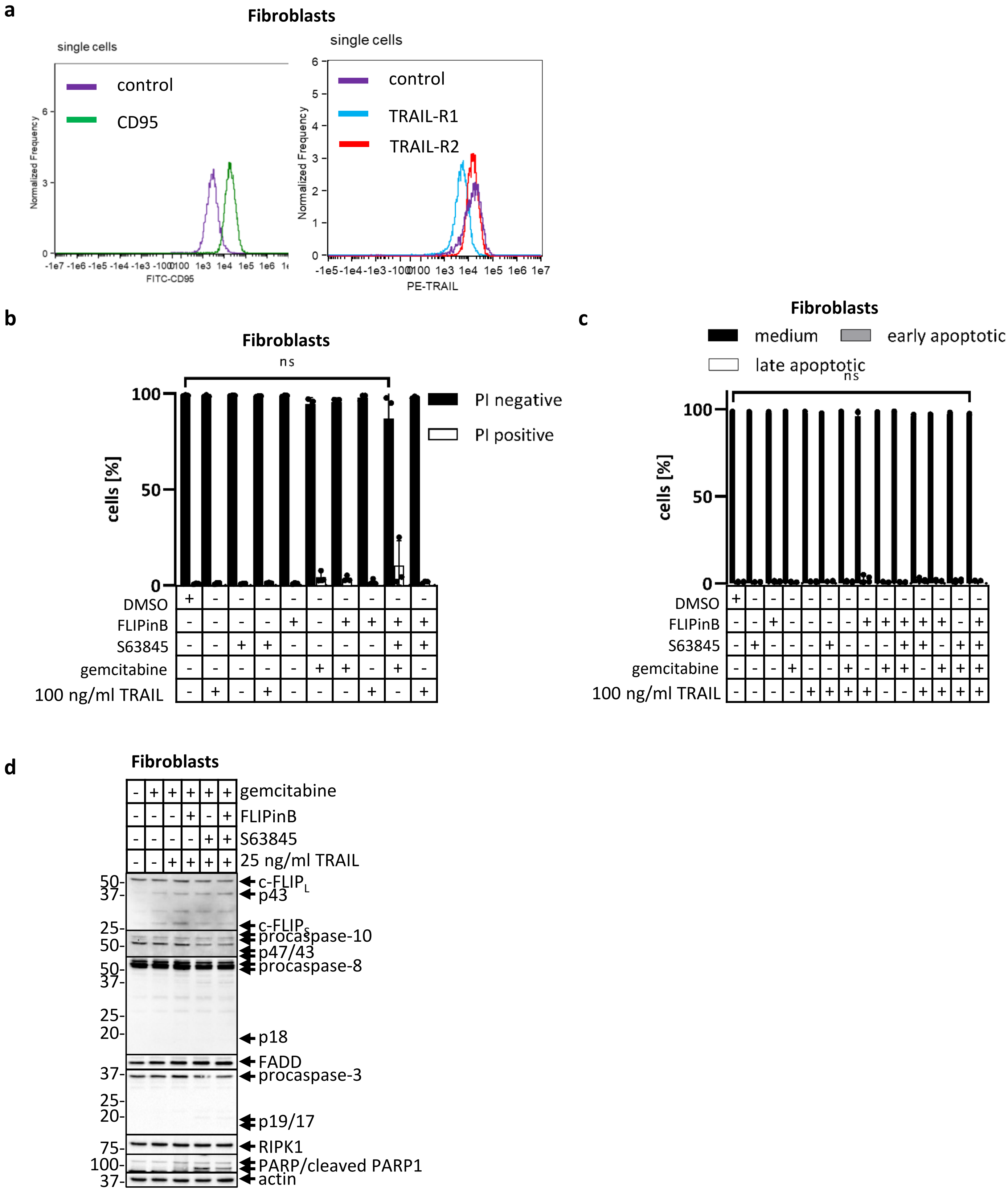

Supplementary figure 6: Human fibroblasts do not respond towards combined treatment. (**a**) Fibroblasts were stained with PE anti-human TRAIL antibody and FITC anti-human CD95 antibody. The cells were analysed using Imaging Flow Cytometry. (**b, c**) Fibroblasts were pretreated for 24 h with 10 ng/ml gemcitabine and for 2 h with 20 µM FLIPinB and 0.005 µM (**c**) or 10 µM (**b**) S63845. Afterwards the cells were stimulated with indicated concentrations of TRAIL for 22 h. Cells were stained with PI only (**b**) or AnnexinV-FITC/PI (**c**). Cells were gated for negative (viable), PI only (late apoptotic), Annexin-V-FITC (early apoptotic) and Annexin-V-FITC/PI (late apoptotic) cells. (**d**) Fibroblasts were pretreated for 24 h with 10 ng/ml gemcitabine and for 2 h with 20 µM FLIPinB and 0.005 µM S63845. Afterwards the cells were stimulated with TRAIL for 3 h. Cell lysates were analyzed by Western Blot with the indicated antibodies. Actin served as loading control. One representative Western Blot out of three is shown.

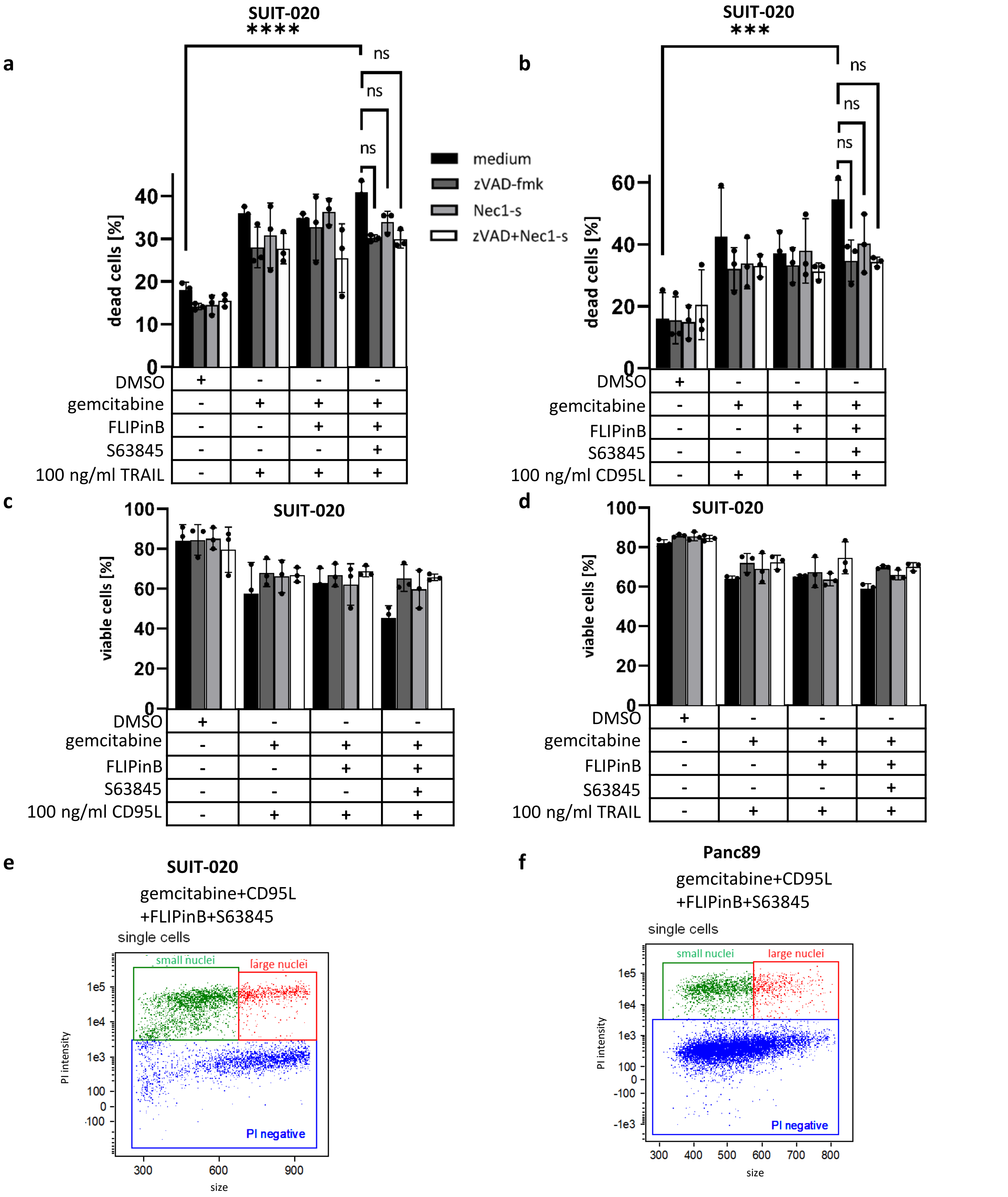

Supplementary figure 7: The measurement of cell viability and cell death upon administration of zVAD-fmk and necroptosis inhibitors (**a, b**) SUIT-020 cells were treated for 24 h with 10 ng/ml gemcitabine, 2 h with 20 µM FLIPinB and 10 µM S63845 and 22 h TRAIL (**a**) or CD95L (**b**). 1 h before CD95L or TRAIL treatment 50 µM zVAD-fmk and/or 10 µM Nec-1s were added. The amount of dead (PI-positive) cells was measured using PI staining and Imaging Flow Cytometry. Mean and standard deviation from three independent experiments are shown. (**c, d**) The amount of viable cells from the experiment (**a, b**) were measured using Imaging Flow cytometry after PI staining. Mean and standard deviation from three independent experiments are shown. ATP content was measured using the Cell Titer-Glo®-Luminescent Cell Viability Assay. Mean and standard deviation from three independent experiments are shown. (**e,f**) Gating strategy for Figure 7 b,c. Single cells are shown in dot plot and cells were separated in PI negative (viable) cells and cells with small or large nuclei (PI positive). For statistical analysis One-way ANOVA with Tukey post hoc tests were used to compare two conditions. The following values were used: ****p<0.0001; ***p<0.001; **p<0.01; *p<0.05; ns not significant.

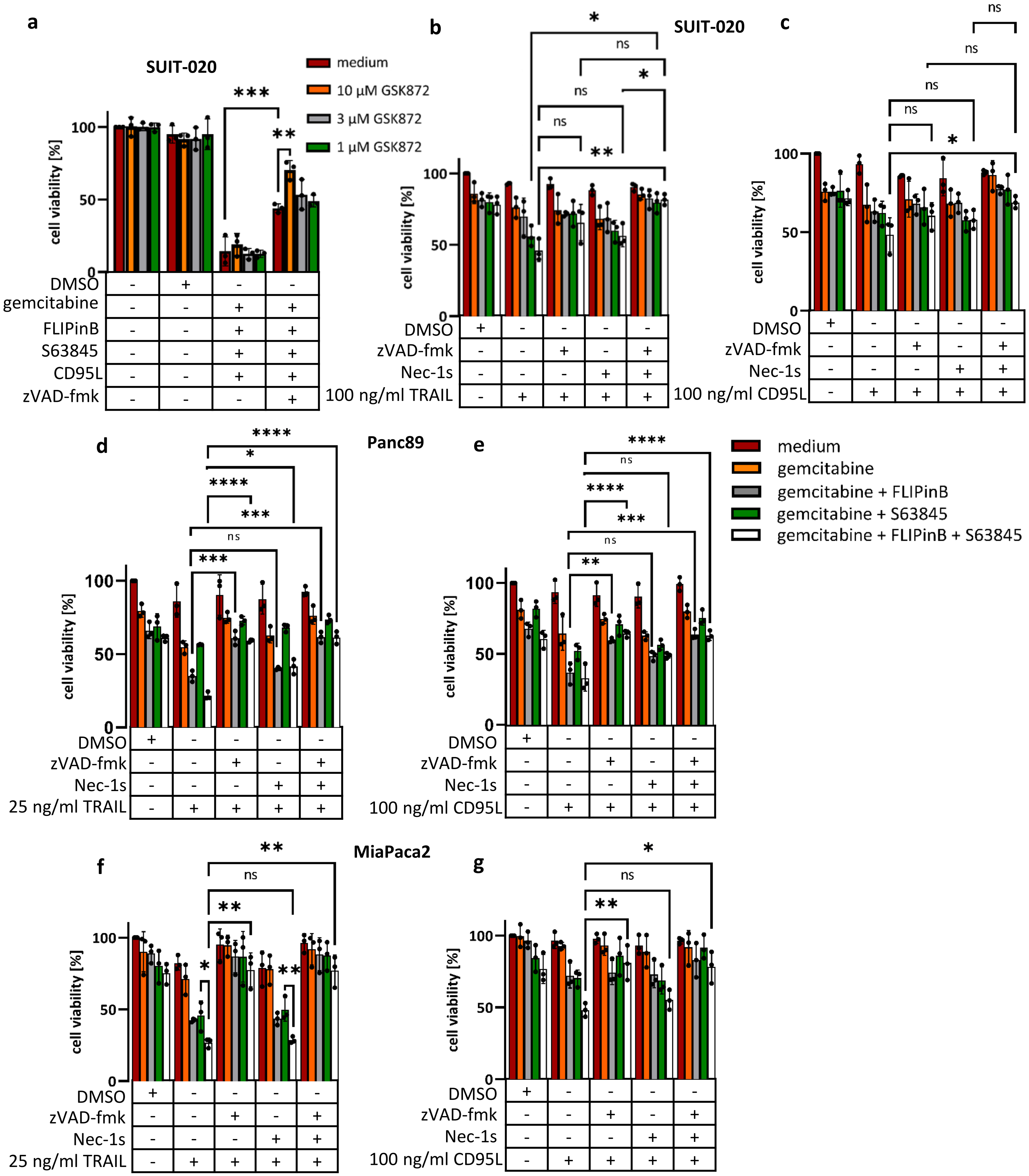

Supplementary figure 8: The analysis of effects of zVAD-fmk and Nec-1s on DL/gemcitabine/FLIPinB/S63845 treatment (**a**) SUIT-020 cells were pretreated for 24 h with 10 ng/ml gemcitabine, 2 h 20 µM FLIPinB and 10 µM S63845. Cells were pretreated for 1 h with different concentrations of GSK872. Cells were afterwards treated for 22 h with 100 ng/ml CD95L.(**b-g**) SUIT-020 cells (**b,c**), Panc89 cells (**d,e**) or MiaPaca2 cells (**f,g**) were pretreated for 24 h with 10 ng/ml gemcitabine and afterwards for 2 h with 20 µM (**b-e**) or 50 µM (**f,g**) FLIPinB and 10 µM (**b,c**), 0.005 µM (**d,e**) or 2.5 µM (**f,g**) S63845 and for 1 h with 10 µM Nec-1s and 50 µM zVAD-fmk. Cells were afterwards treated for 22 h with TRAIL (**b,d,f**) or CD95L (**c,e,g**). ATP content was measured using the Cell Titer-Glo®-Luminescent Cell Viability Assay. Mean and standard deviation from three independent experiments are shown. For statistical analysis One-way ANOVA with Tukey post hoc tests were used to compare two conditions. The following values were used: ****p<0.0001; ***p<0.001; **p<0.01; *p<0.05; ns not significant.

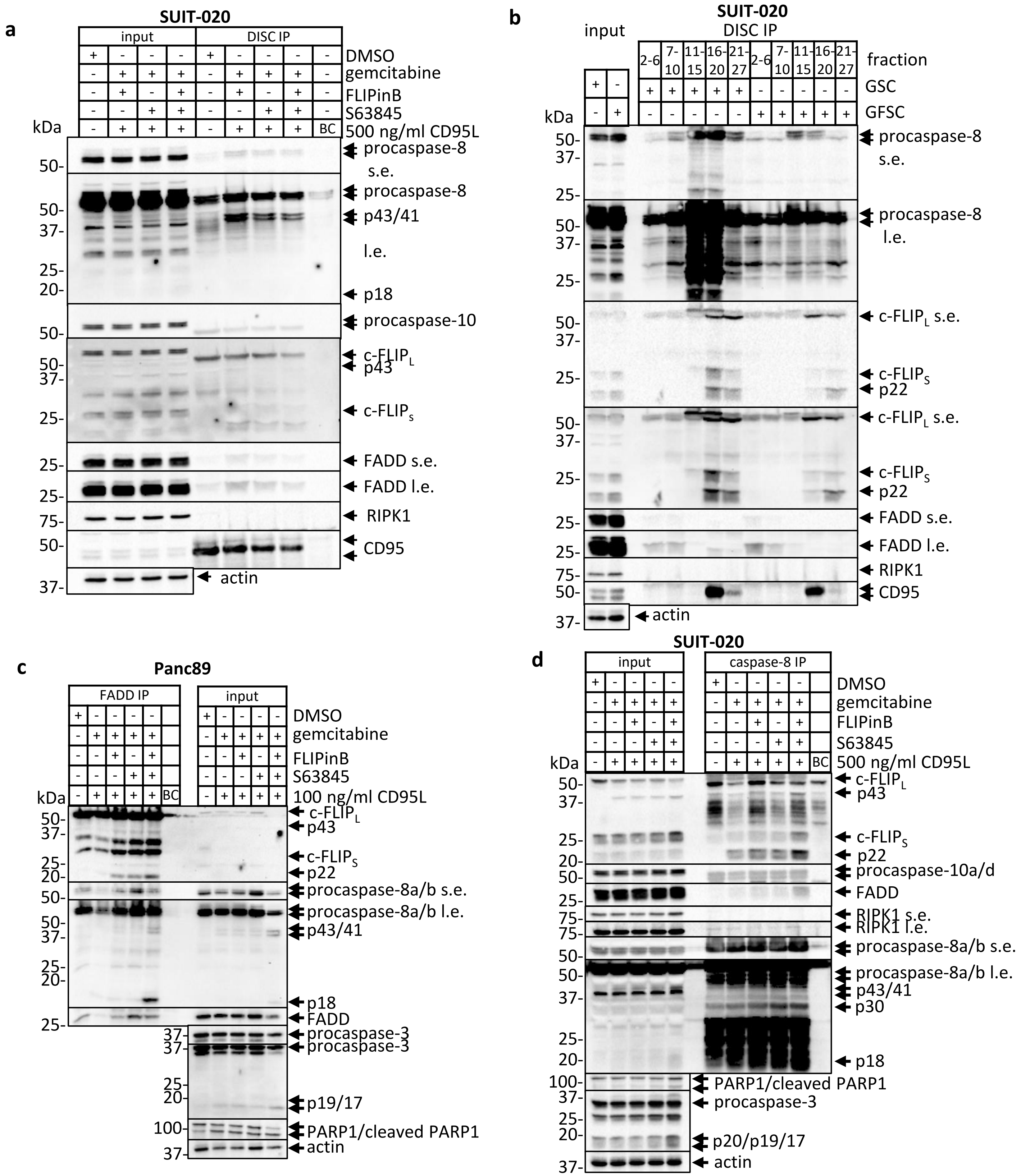

Supplementary figure 9: The analysis of macromolecular complexes upon DL/gemcitabine/FLIPinB, DL/gemcitabine/S63845 and DL/gemcitabine/FLIPinB/S63845 treatments (**a**,**c**,**d**) SUIT-020 (**a**,**d**) or Panc89 cells (**c**) cells were pretreated for 24 h with 10 ng/ml gemcitabine, which was followed by treatment for 2 h with 20 µM FLIPinB and 10 µM (**a**,**d**) or 0.005 µM (**c**) S63845. Afterwards the cells were stimulated with CD95L for 2 h (**a**,**d**) or 5 h (**c**). Cell lysates were immunoprecipitated using anti-APO-1 (**a**), anti-FADD (FADD-IP) (**c**) or anti-caspase-8 (caspase-8 IP) (**d**) antibodies antibody. IPs were analyzed using Western Blot. Total cell lysates were analyzed in parallel (input). One representative Western Blot out of two (**a**) or three (**c**,**d**) are shown. CD95, FADD and caspase-8 served as loading controls for DISC-, FADD-, caspase-8-IP and actin as a loading control for lysates. (**b**) SUIT-020 cells were treated like in (**a**). Cell lysates were analyzed by gelfiltration. After gelfiltration, fractions were merged like indicated and immunoprecipitation was carried out using anti-APO-1 antibody (DISC-IP). DISC-IP was analyzed using corresponding antibodies by Western Blot. Total cell lysates were analyzed in parallel. One representative Western Blot out of two is shown. CD95 served as loading control for DISC-IP and actin as loading control for lysates. Abbreviations: s.e. short exposure, l.e. long exposure, IP immunoprecipitation, BC Bead control, GSC 10 ng/ml gemcitabine+10 µM S63845+ 500 ng/ml CD95L, GFSC 10 ng/ml gemcitabine+10 µM S63845+ 20 µM FLIPinB+ 500 ng/ml CD95L.

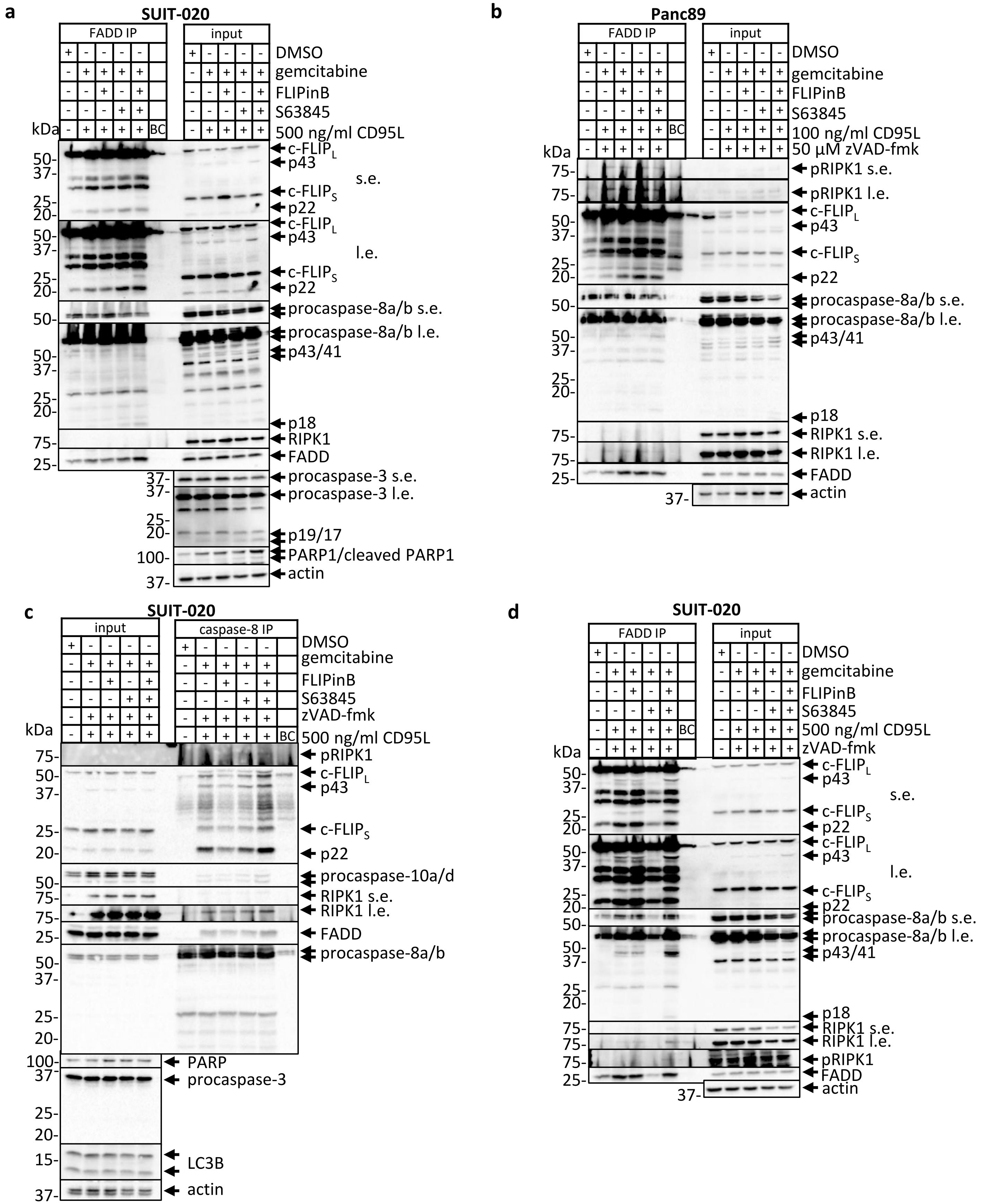

Supplementary figure 10: Increase in complex II formation upon DL/gemcitabine/FLIPinB/S63845 treatments in comparison to DL/gemcitabine/S63845 treatment (**a-d**) SUIT-020 cells (**a, c, d**) or Panc89 cells (**b**) were pretreated with 10 ng/ml gemcitabine for 24 h and subsequently with 10 µM (**a,c,d**) or 0.005 µM (**b**) S63845 and 20 µM FLIPinB for 2 h. Afterwards the cells were treated with CD95L for 5 h. 50 µM zVAD-fmk was added 1 h before CD95L treatment. The immunoprecipitation was carried out with anti-FADD (FADD-IP) (**a, b, d**) or anti-caspase-8 (caspase-8 IP) (**c**) antibodies. The IPs were analyzed using the corresponding antibodies. Western Blot analysis of caspase-8 or FADD serves as a loading control for IPs, respectively. Total cell lysates were analyzed, additionally (input). Actin served as loading control for total cell lysates. Abbreviations: s.e. short exposure, l.e. long exposure, IP immunoprecipitation, BC Bead control.

1a

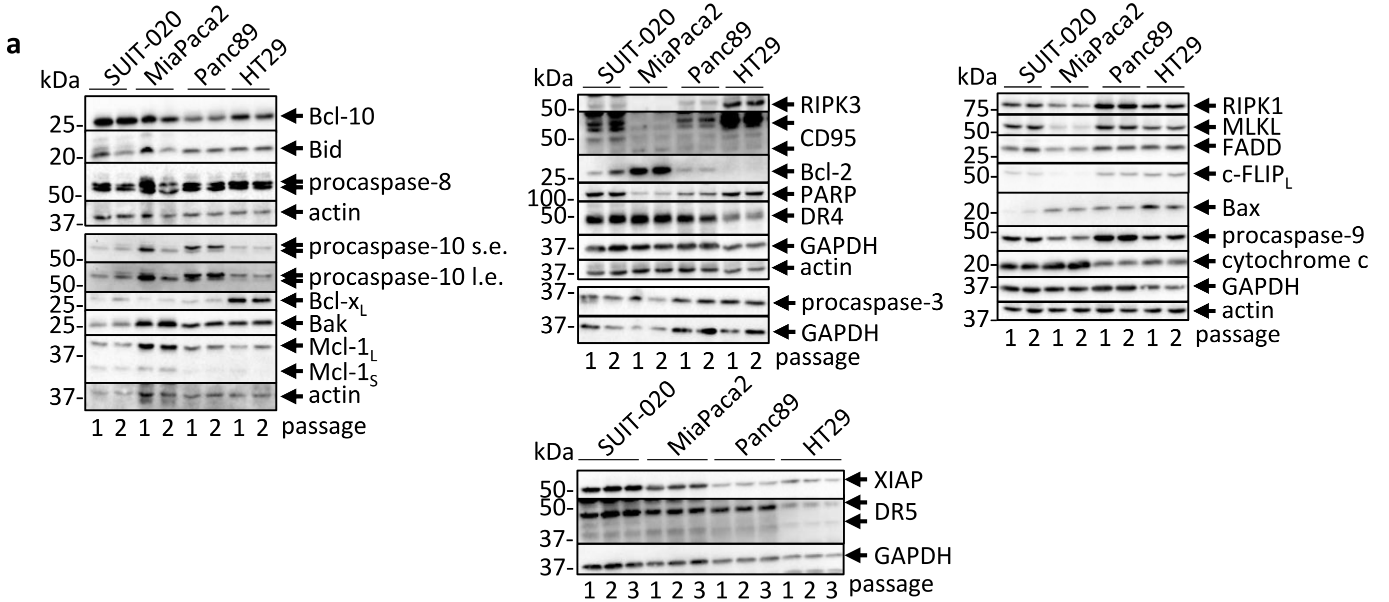
a
kDa
SUIT-020
MiaPaca2
Panc89
HT29
Bcl-10
Bid
procaspase-8
actin
procaspase-10 s.e.
procaspase-10 l.e.
Bcl-xL
Bak
Mcl-1L
Mcl-1S
actin
passage
RIPK3
CD95
Bcl-2
PARP
DR4
GAPDH
actin
procaspase-3
GAPDH
passage
RIPK1
MLKL
FADD
c-FLIPL
Bax
procaspase-9
cytochrome c
GAPDH
actin
passage
XIAP
DR5
GAPDH
passage

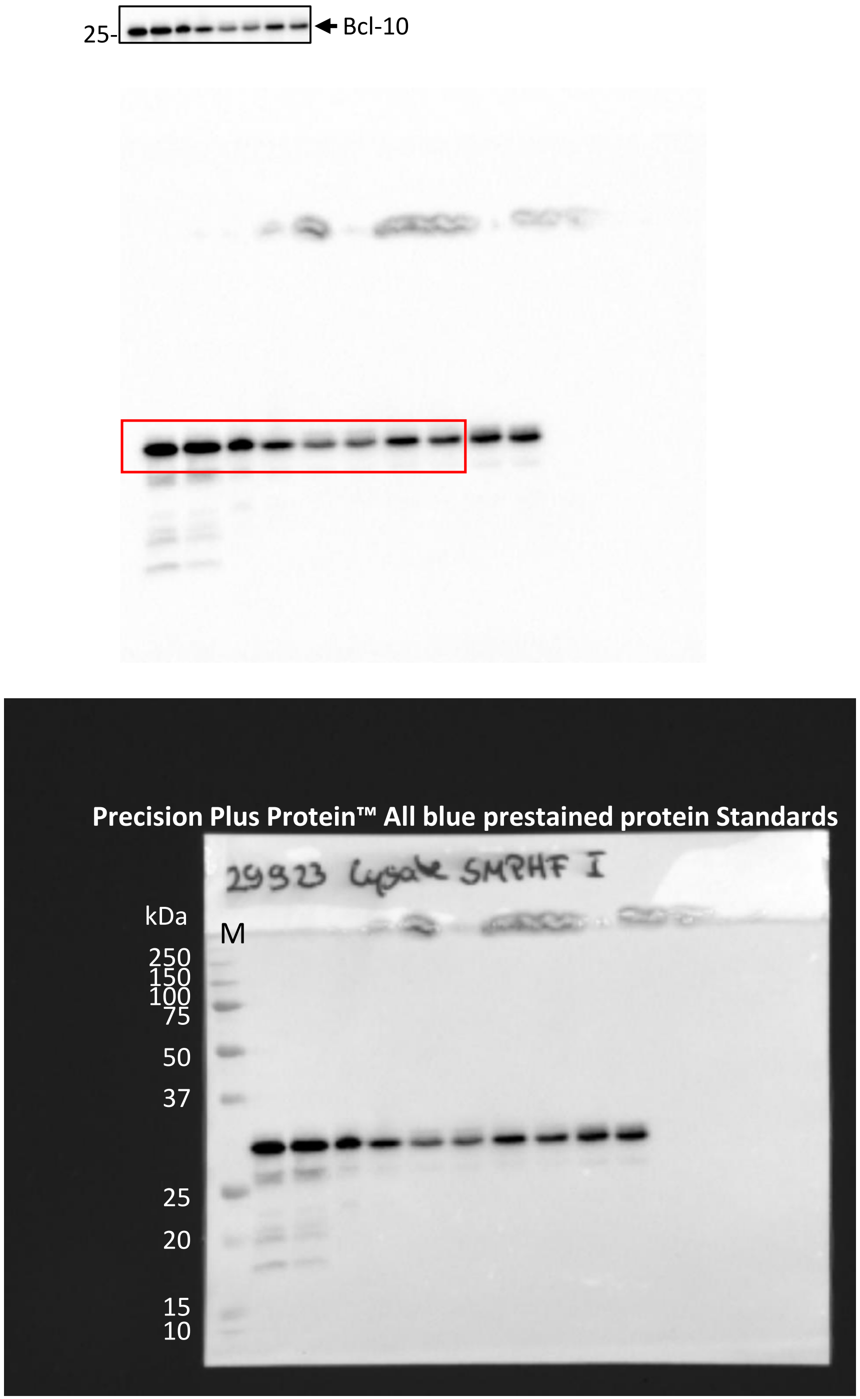
25-
Bcl-10
Precision Plus Protein™ All blue prestained protein Standards
kDa
M
250
150
100
75
50
37
25
20
15
10

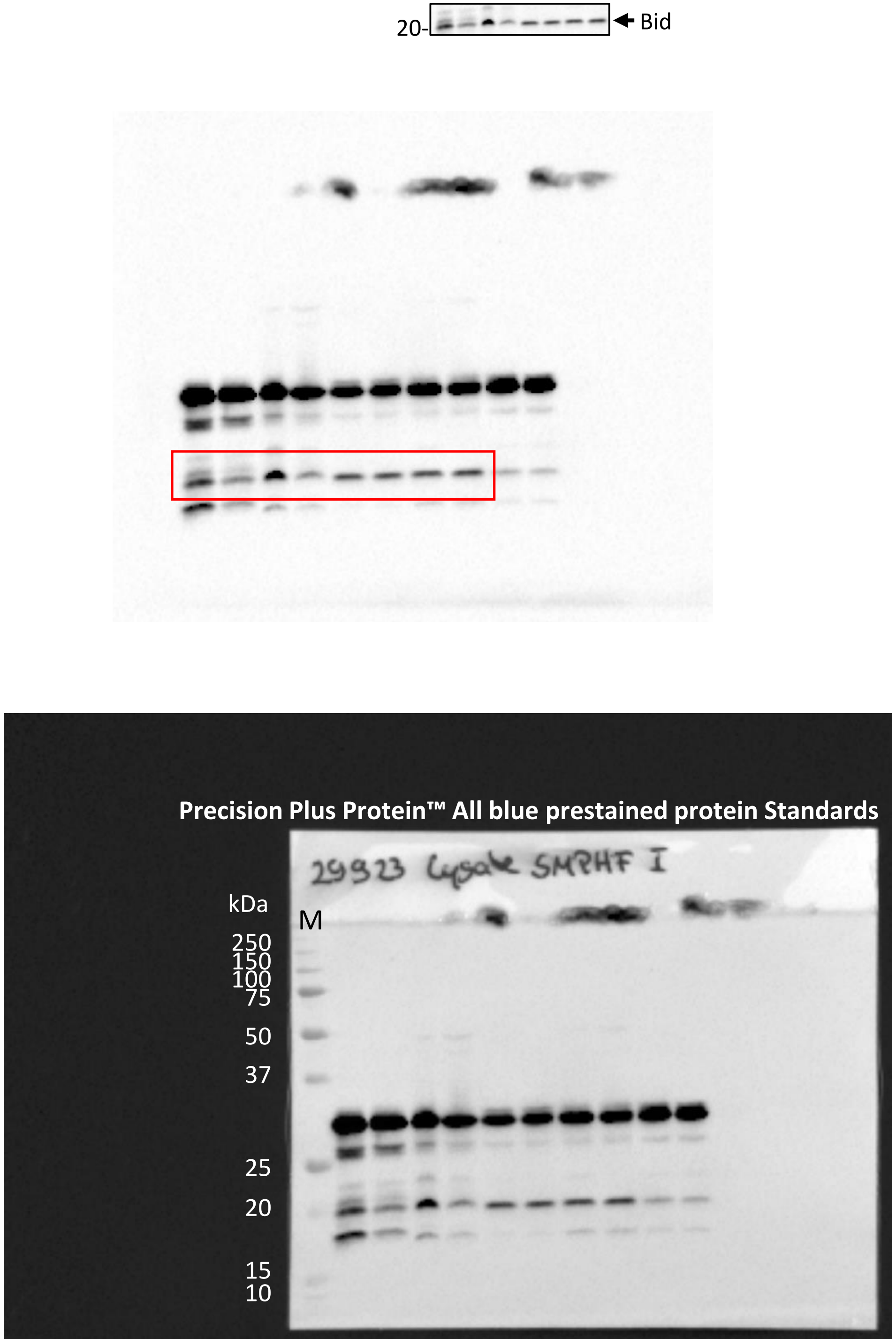
20-
Bid
Precision Plus Protein™ All blue prestained protein Standards
kDa
M
250
150
100
75
50
37
25
20
15
10

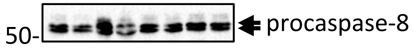
50-
procaspase-8

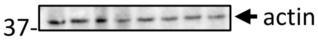
37-
actin

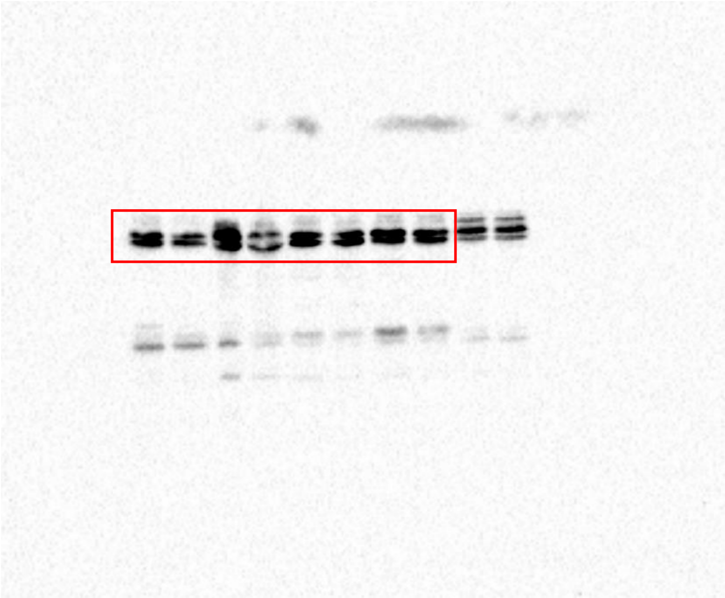

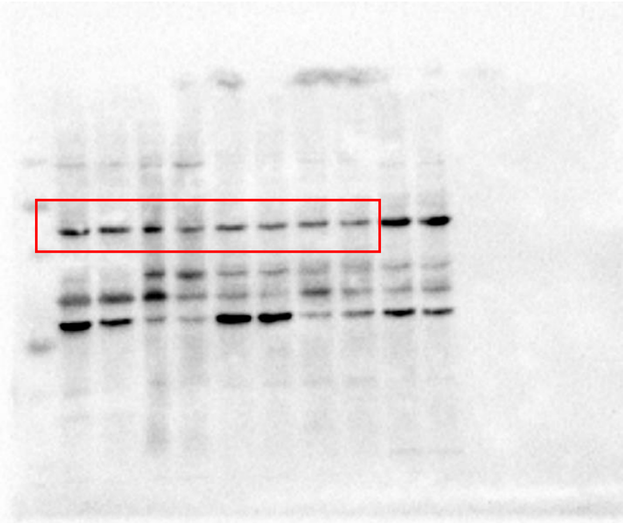

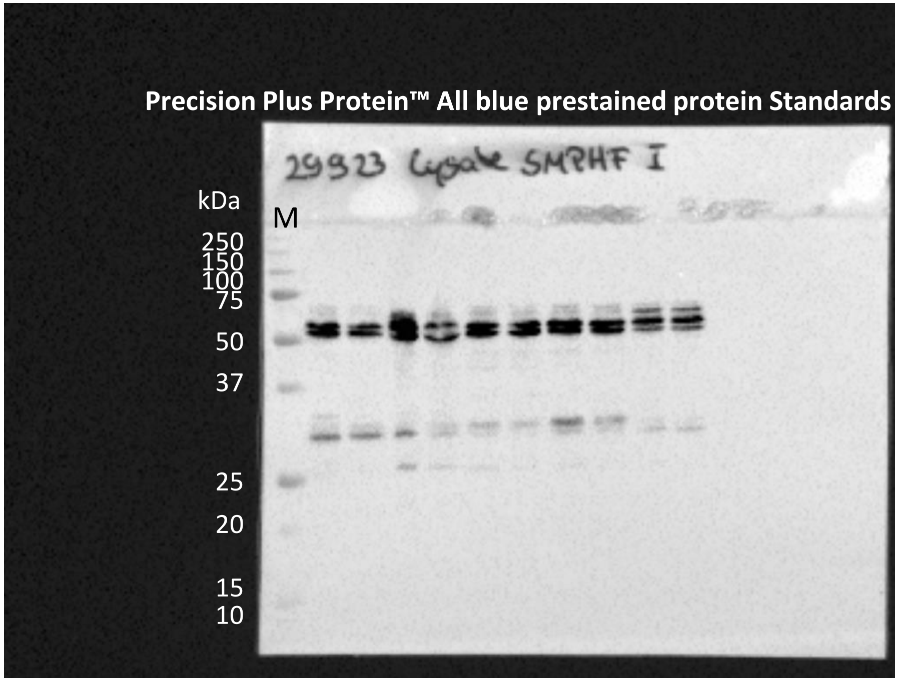
Precision Plus Protein™ All blue prestained protein Standards
kDa
M
250
150
100
75
50
37
25
20
15
10

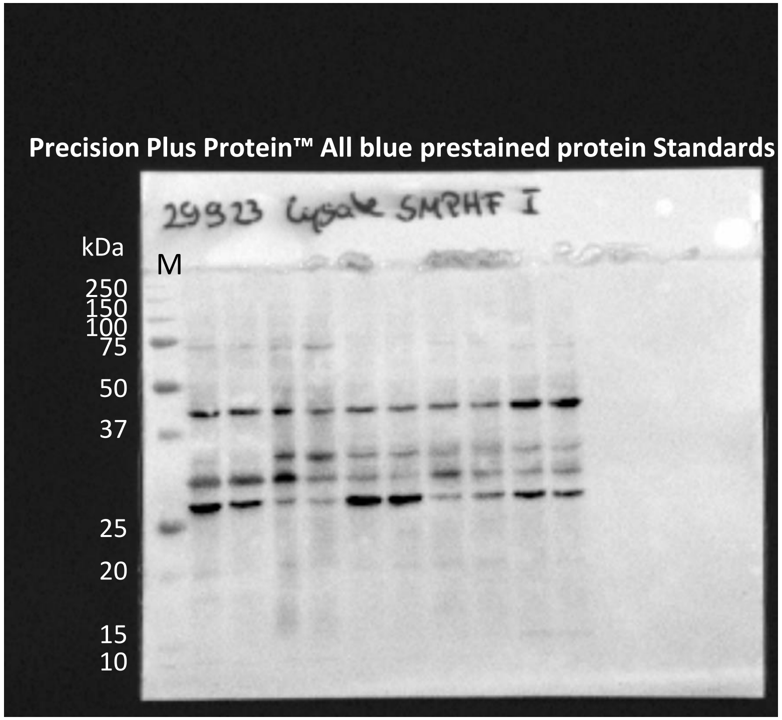
Precision Plus Protein™ All blue prestained protein Standards
kDa
M
250
150
100
75
50
37
25
20
15
10

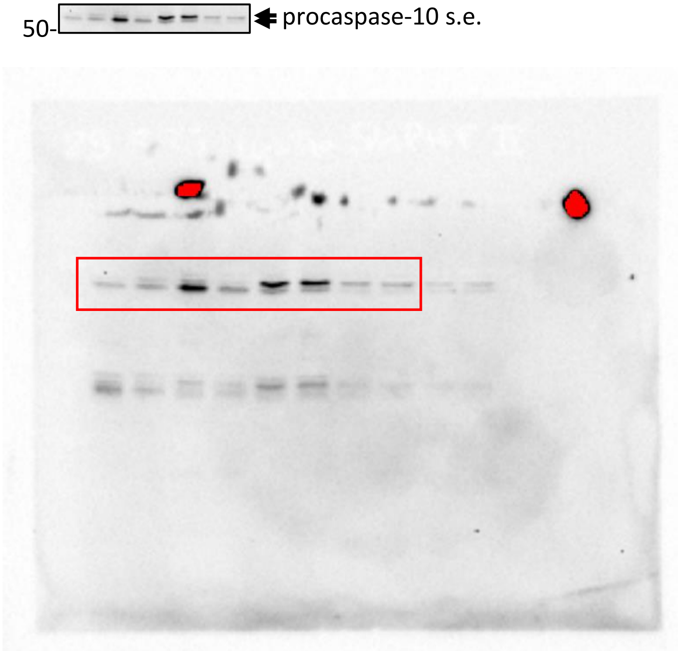
50-
procaspase-10 s.e.

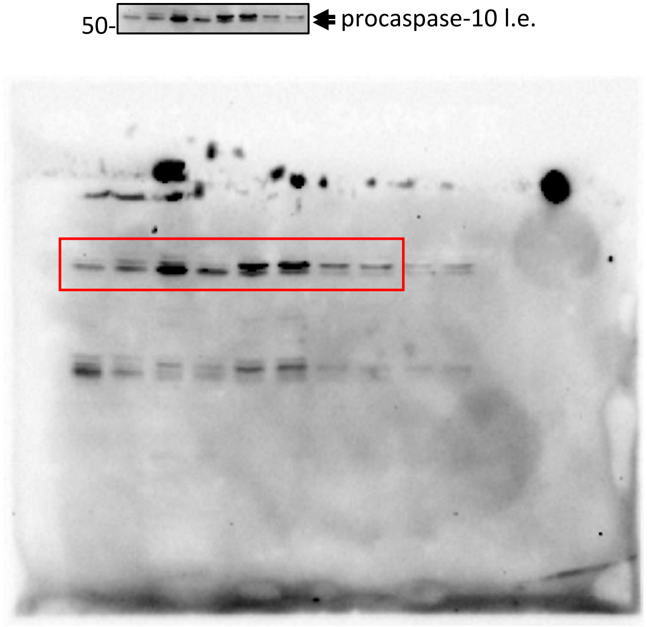
50-
procaspase-10 l.e.

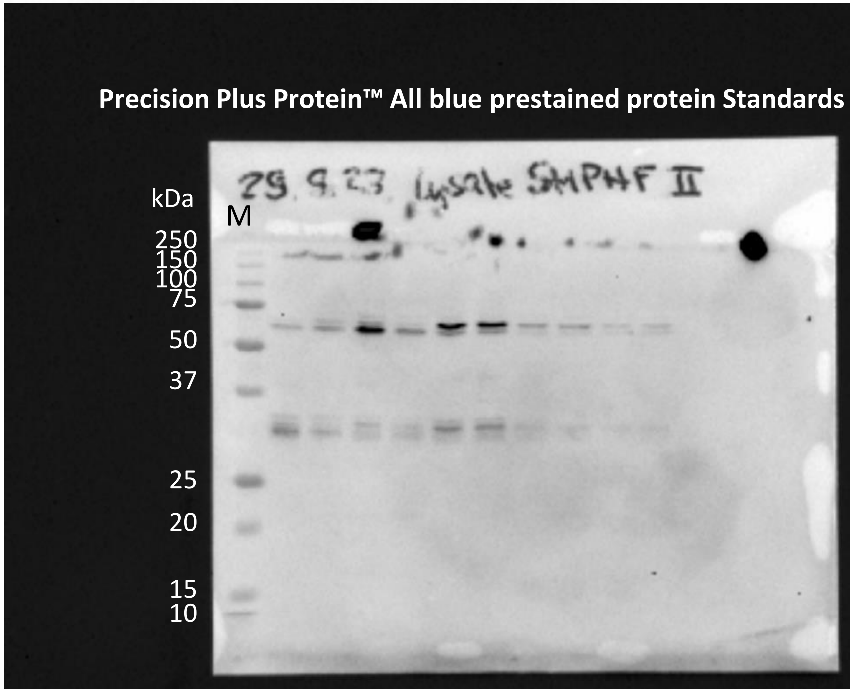
Precision Plus Protein™ All blue prestained protein Standards
kDa
M
250
150
100
75
50
37
25
20
15
10
29.9.23 lysate SMPHF II

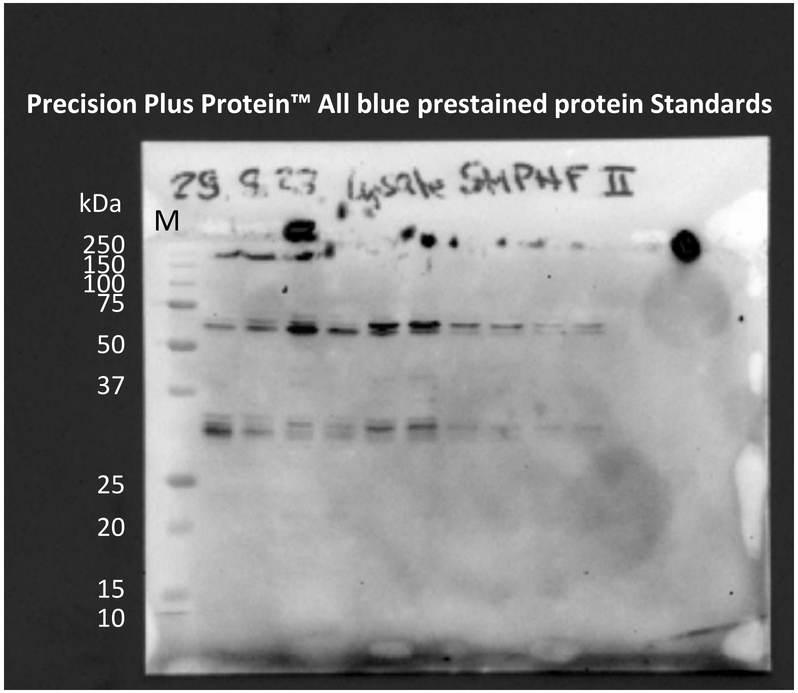
Precision Plus Protein™ All blue prestained protein Standards
kDa
M
250
150
100
75
50
37
25
20
15
10
29.9.23 lysate SMPHF II

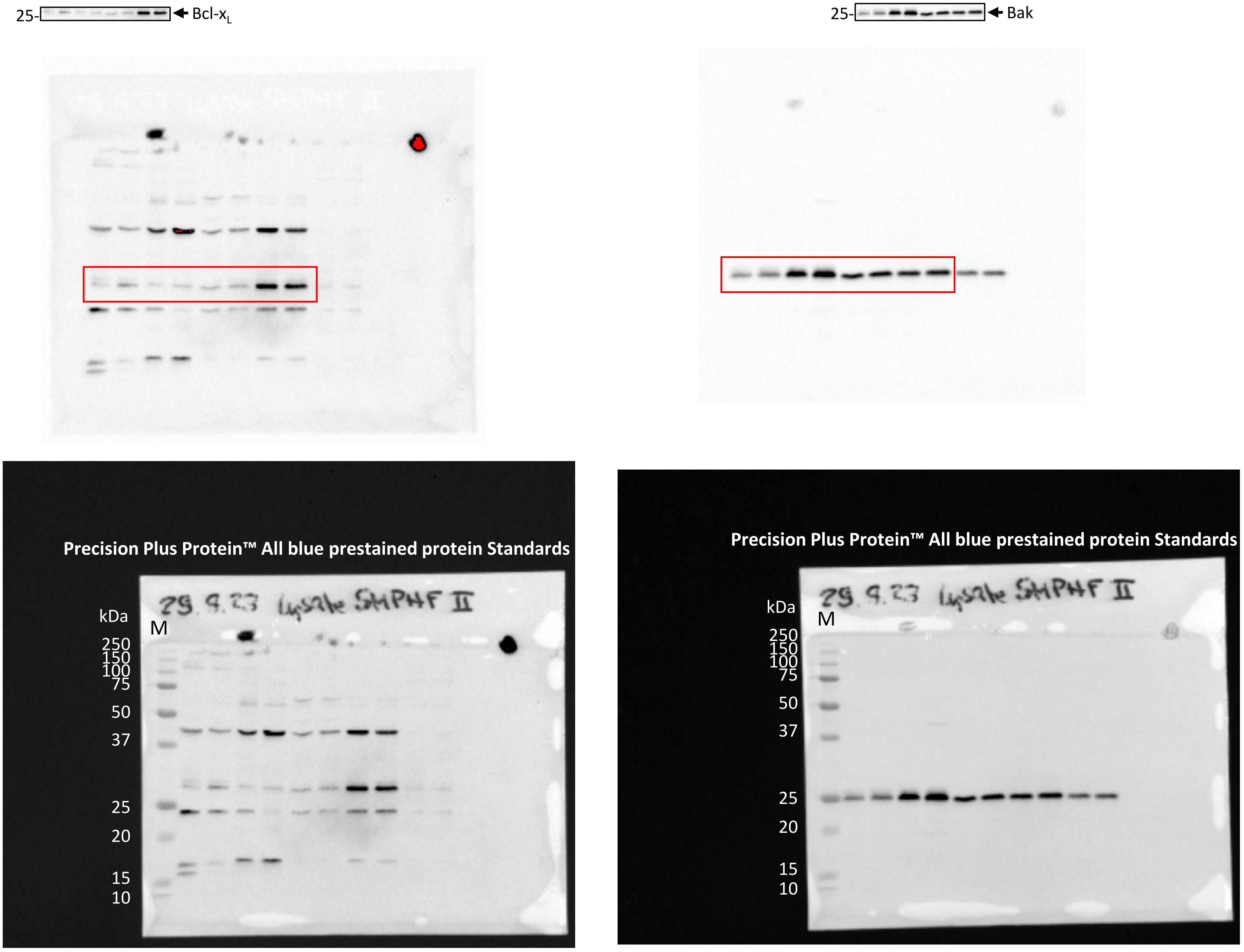

25-
Bcl-x$_L$
25-
Bak
Precision Plus Protein™ All blue prestained protein Standards
kDa
M
29.9.23 lysate SMPHF II
250
150
100
75
50
37
25
20
15
10
Precision Plus Protein™ All blue prestained protein Standards
kDa
M
29.9.23 lysate SMPHF II
250
150
100
75
50
37
25
20
15
10

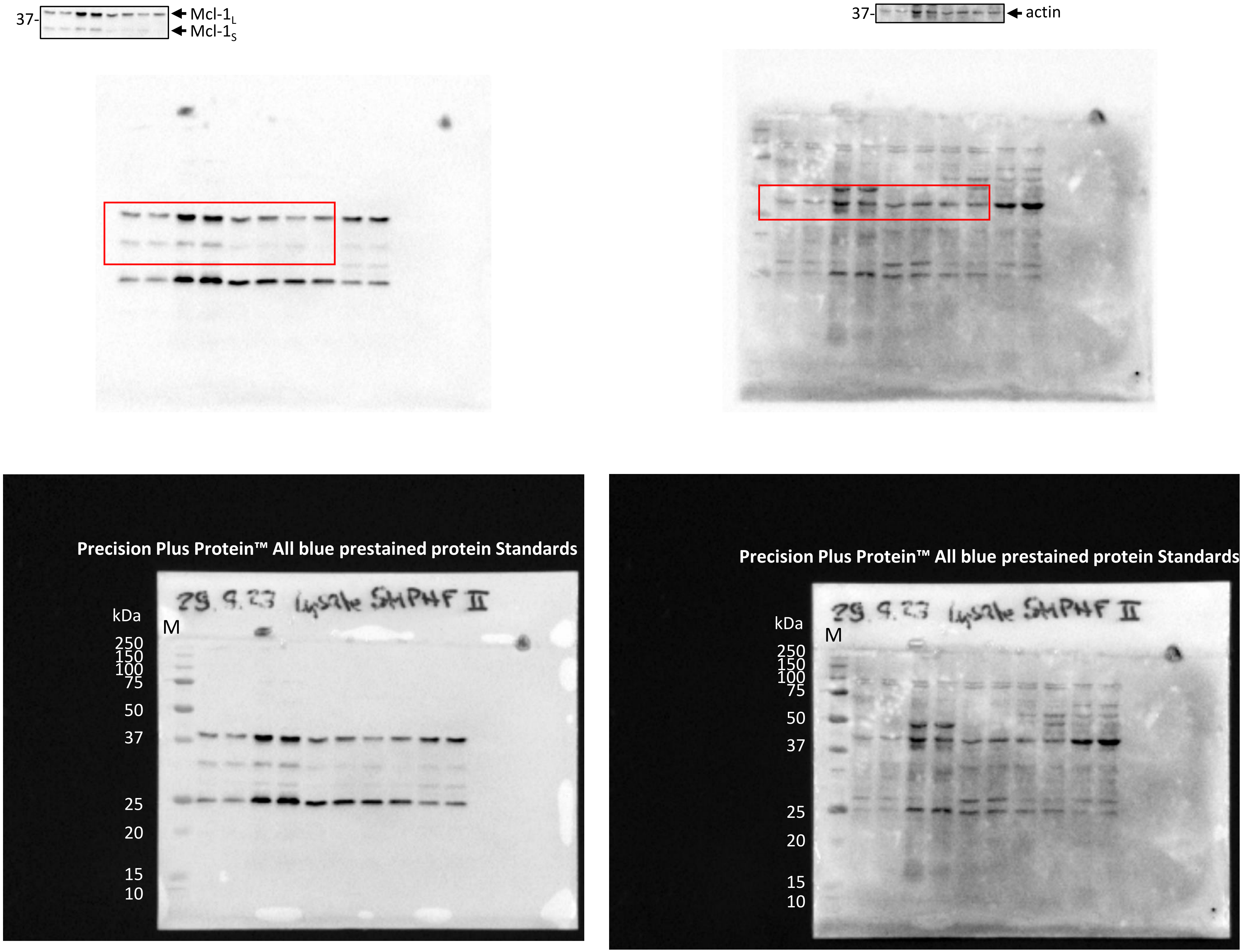
37-
Mcl-1$_L$
Mcl-1$_S$
37-
actin
Precision Plus Protein™ All blue prestained protein Standards
kDa
M
250
150
100
75
50
37
25
20
15
10
Precision Plus Protein™ All blue prestained protein Standards
kDa
M
250
150
100
75
50
37
25
20
15
10

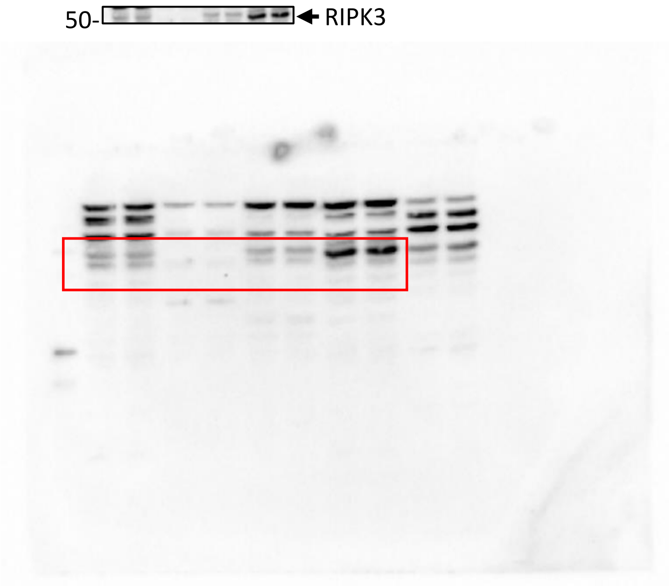
50-
RIPK3

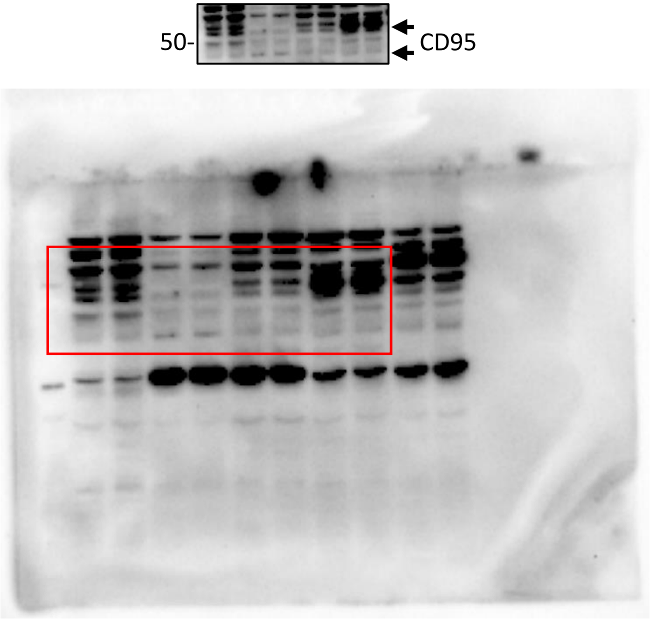
50-
CD95

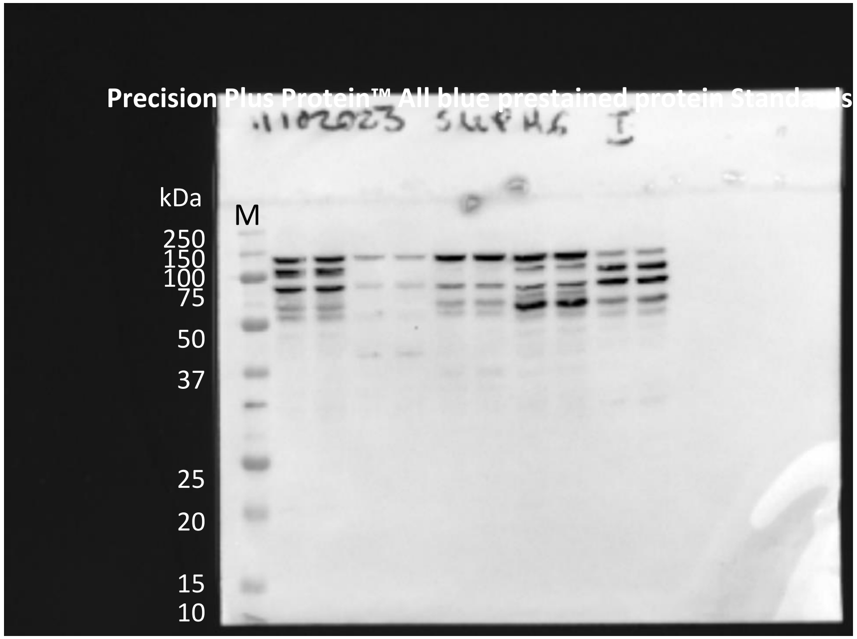
Precision Plus Protein™ All blue prestained protein Standards
kDa
M
250
150
100
75
50
37
25
20
15
10

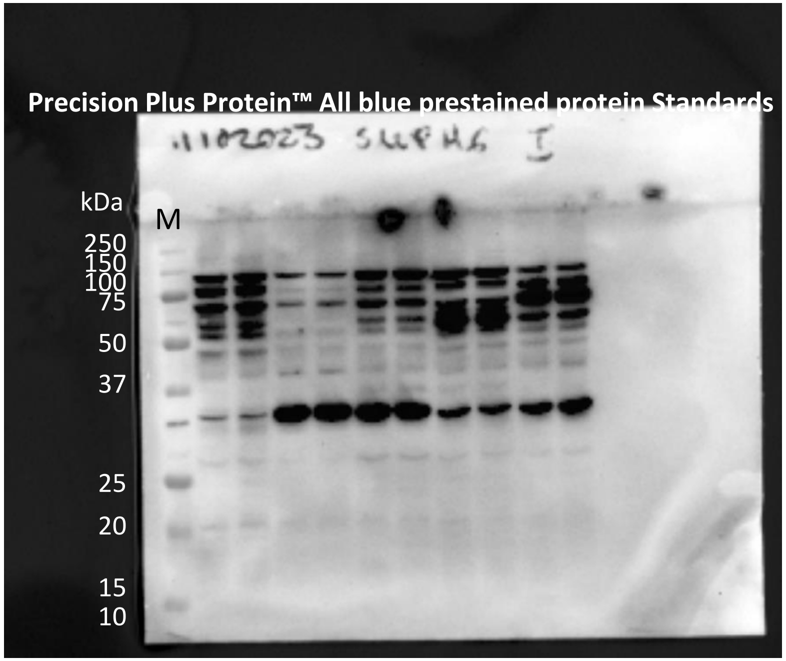
Precision Plus Protein™ All blue prestained protein Standards
kDa
M
250
150
100
75
50
37
25
20
15
10

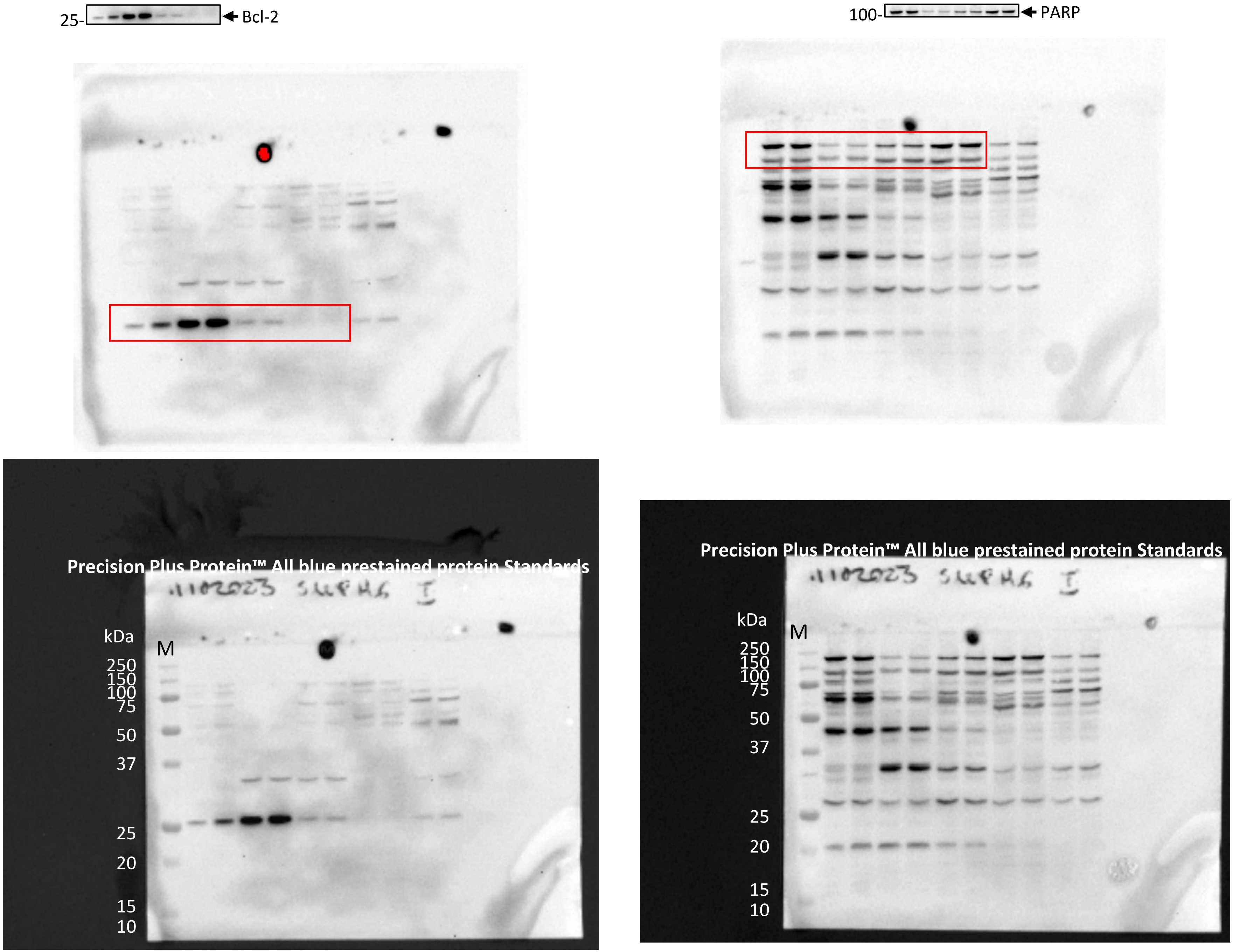

25-
Bcl-2
100-
PARP
Precision Plus Protein™ All blue prestained protein Standards
kDa
M
250
150
100
75
50
37
25
20
15
10
Precision Plus Protein™ All blue prestained protein Standards
kDa
M
250
150
100
75
50
37
25
20
15
10

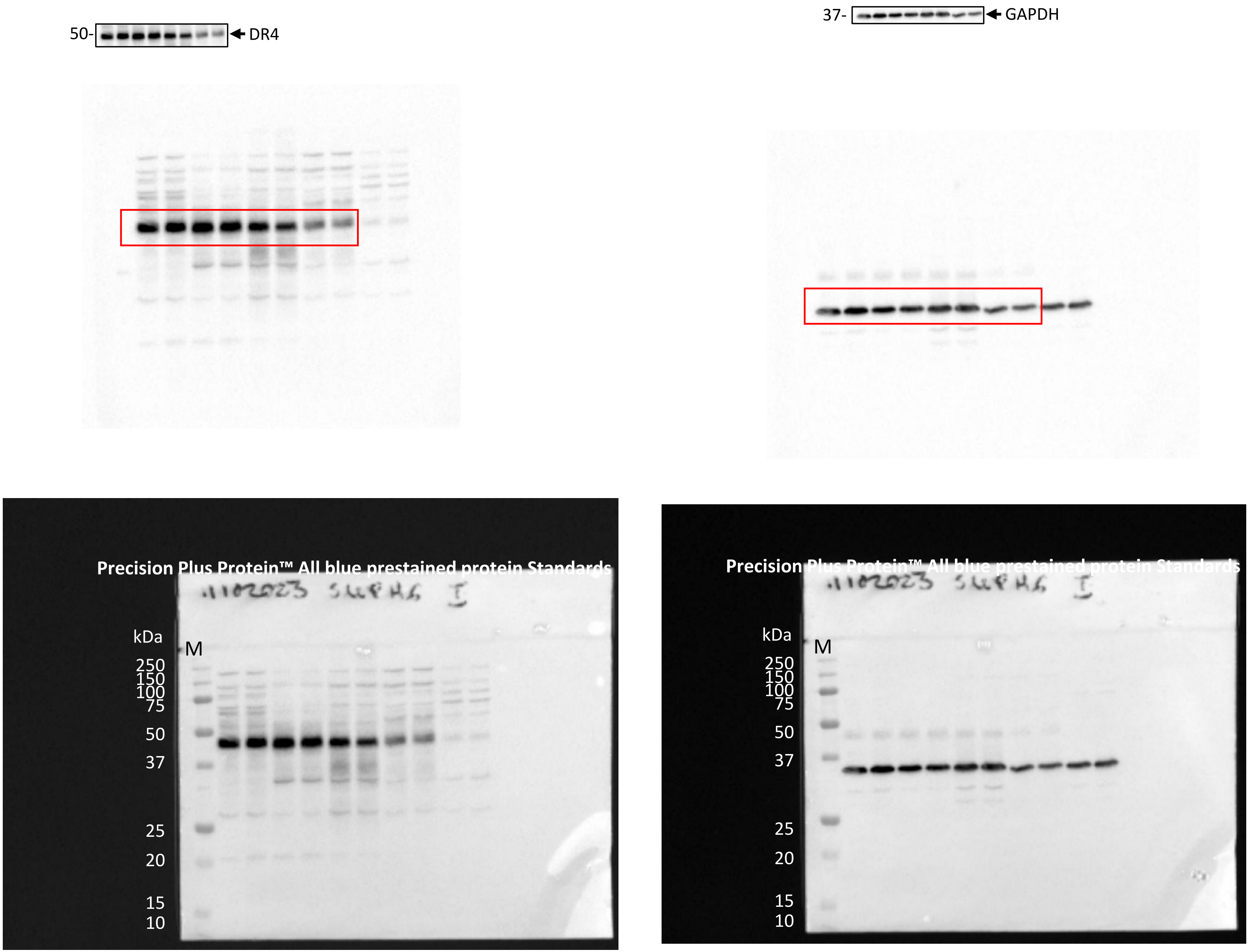

50-
DR4
37-
GAPDH
Precision Plus Protein™ All blue prestained protein Standards
kDa
M
250
150
100
75
50
37
25
20
15
10
Precision Plus Protein™ All blue prestained protein Standards
kDa
M
250
150
100
75
50
37
25
20
15
10

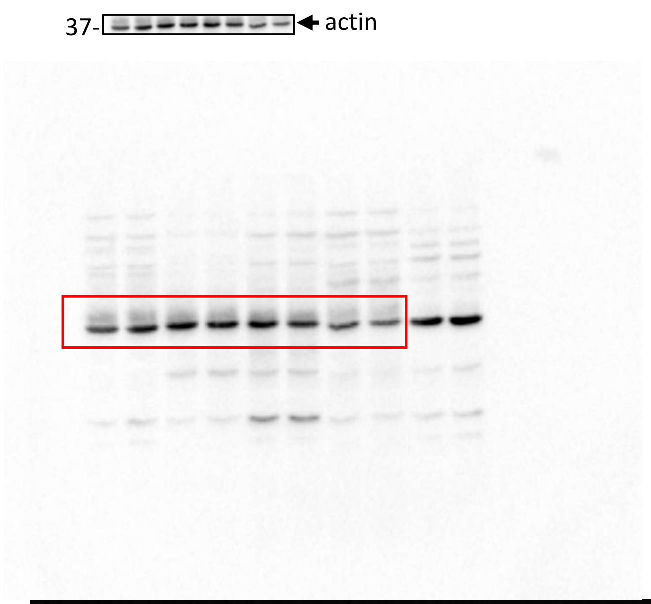
37-
actin

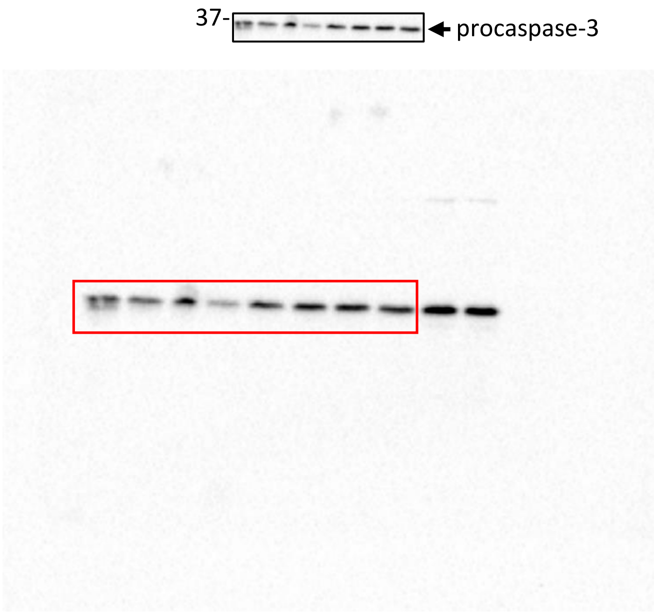
37-
procaspase-3

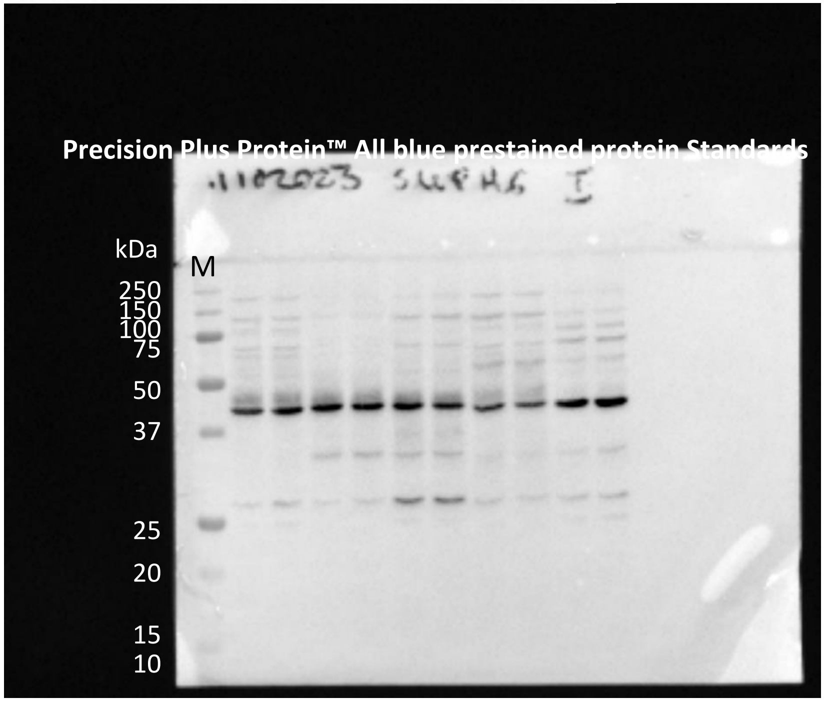
Precision Plus Protein™ All blue prestained protein Standards
kDa
M
250
150
100
75
50
37
25
20
15
10

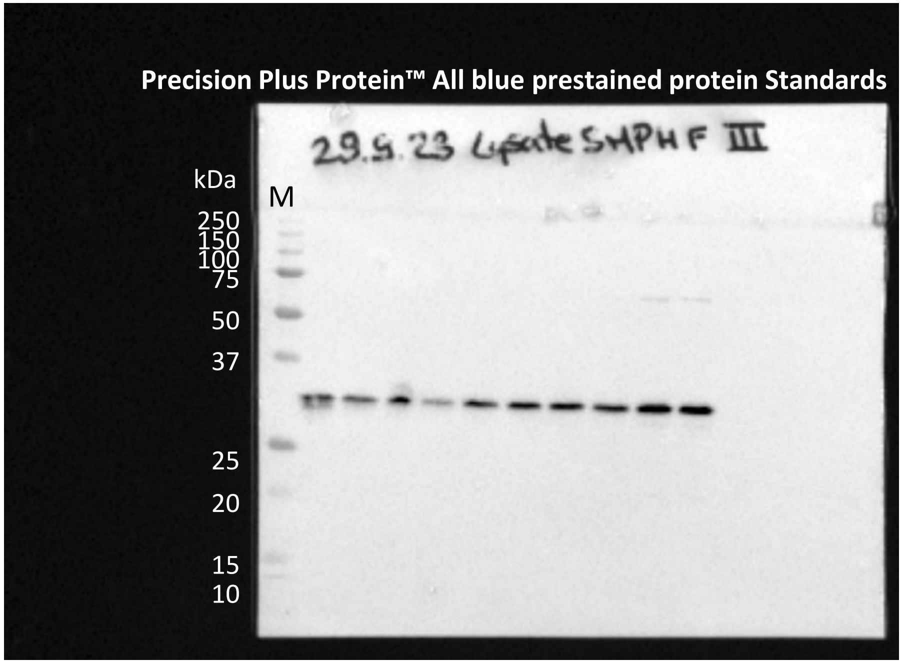
Precision Plus Protein™ All blue prestained protein Standards
kDa
M
250
150
100
75
50
37
25
20
15
10

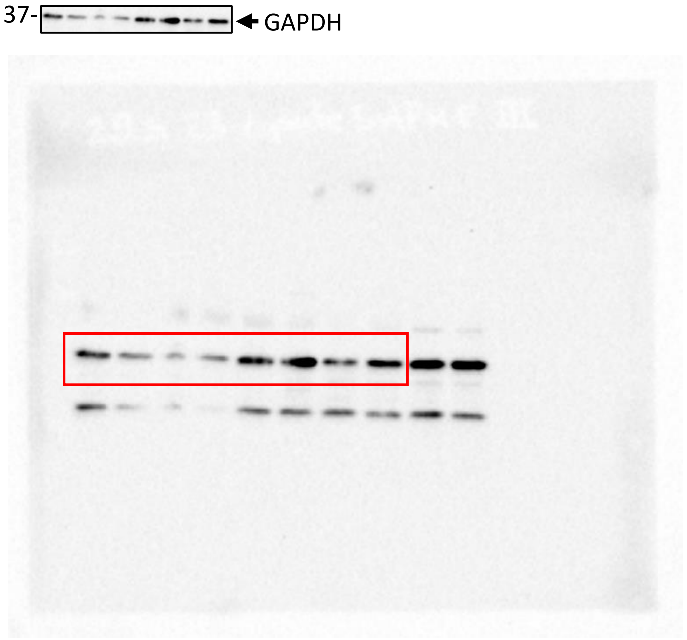
37-
GAPDH

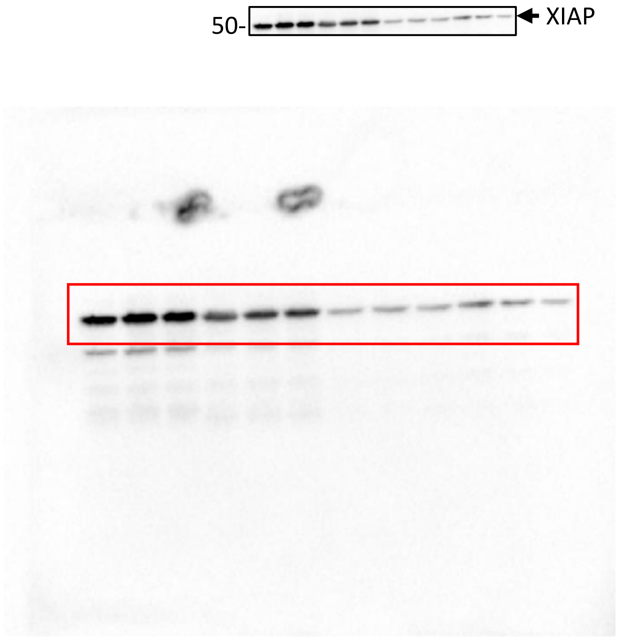
50-
XIAP

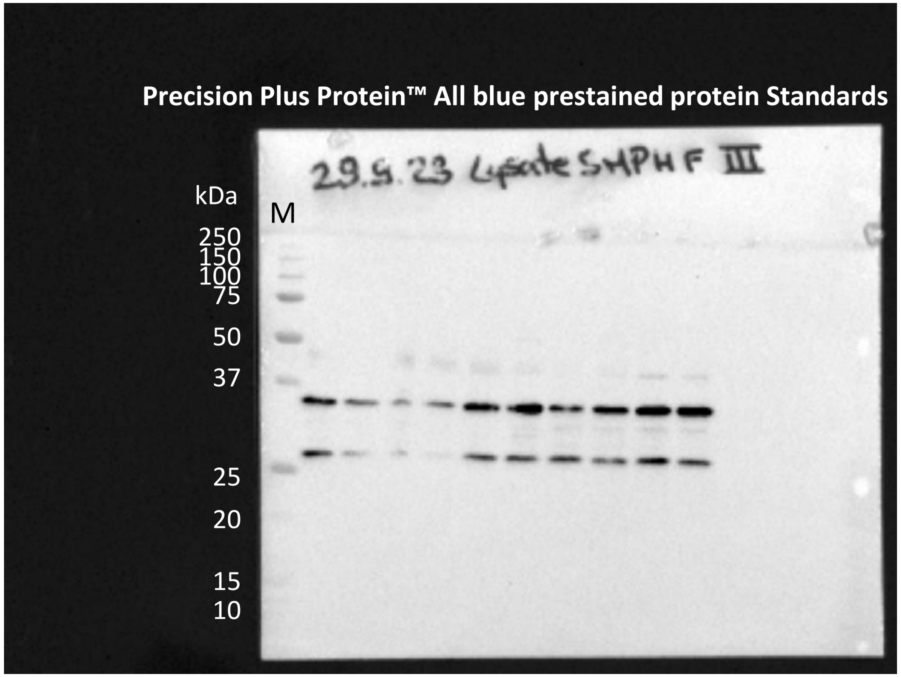
Precision Plus Protein™ All blue prestained protein Standards
kDa
M
250
150
100
75
50
37
25
20
15
10

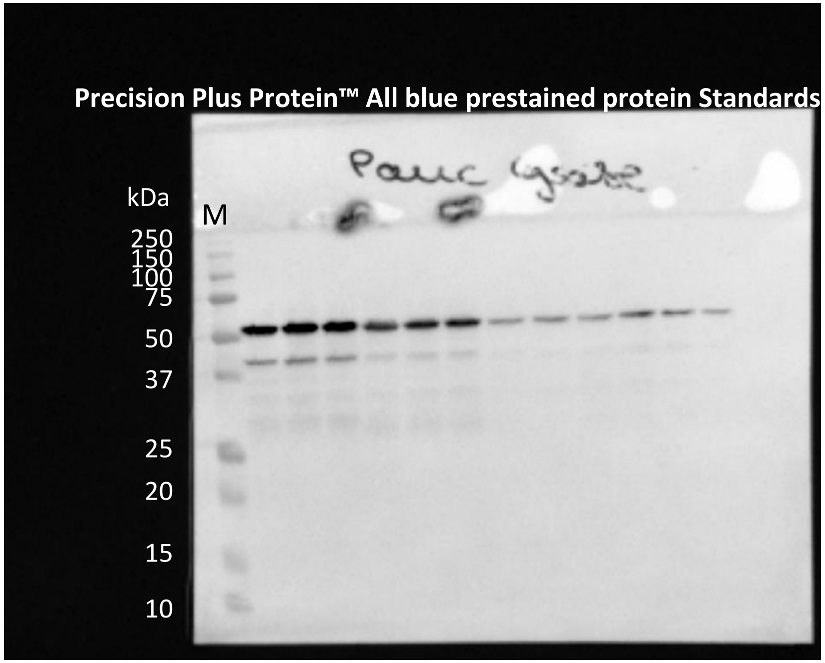
Precision Plus Protein™ All blue prestained protein Standards
kDa
M
250
150
100
75
50
37
25
20
15
10

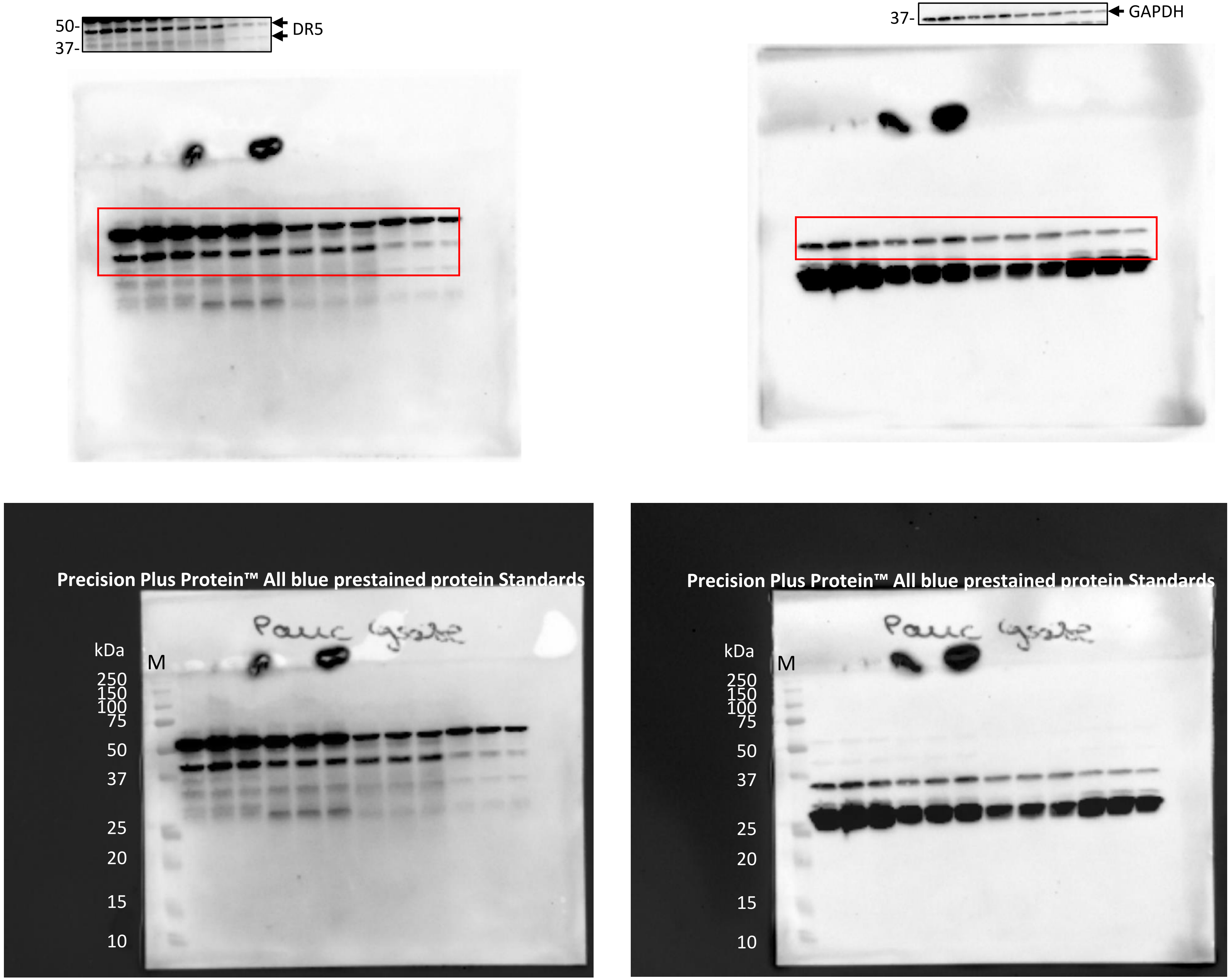

50-
37-
DR5
37-
GAPDH
Precision Plus Protein™ All blue prestained protein Standards
kDa
M
250
150
100
75
50
37
25
20
15
10
Precision Plus Protein™ All blue prestained protein Standards
kDa
M
250
150
100
75
50
37
25
20
15
10

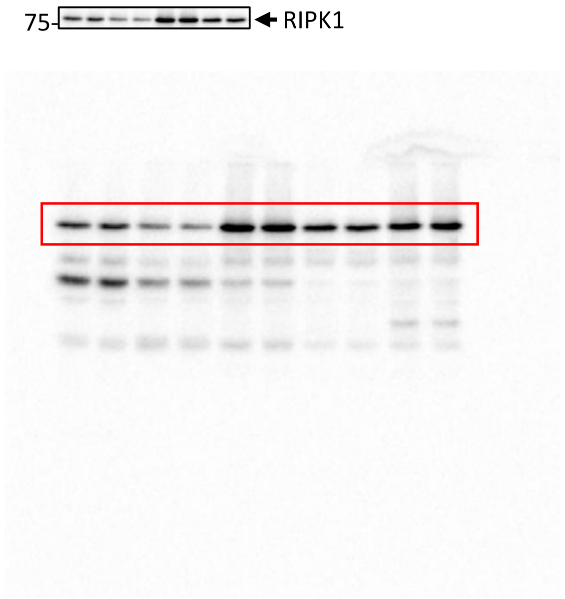
75
RIPK1

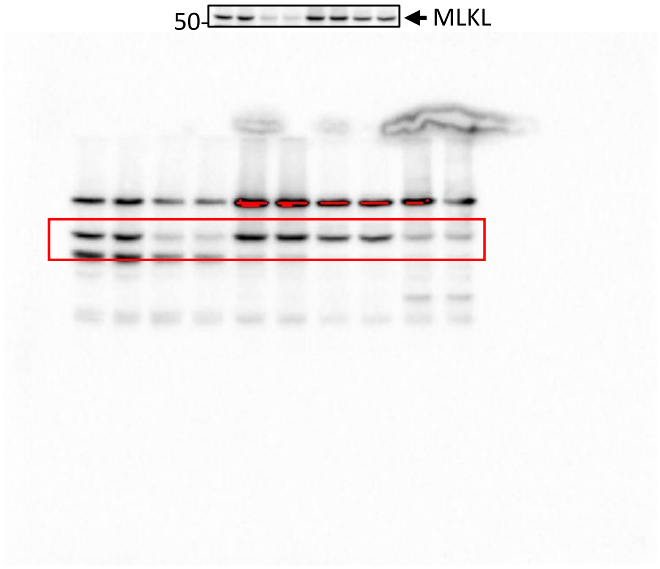
50
MLKL

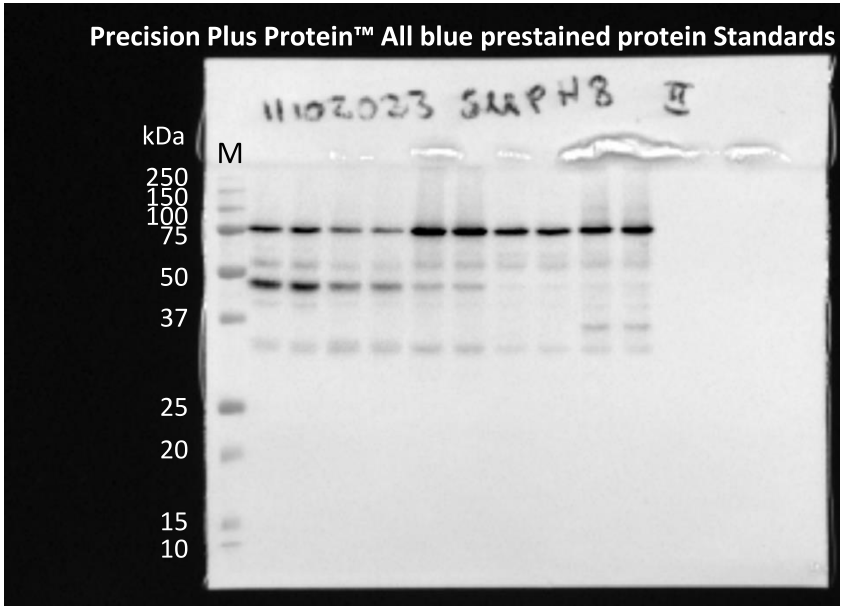
Precision Plus Protein™ All blue prestained protein Standards
kDa
M
250
150
100
75
50
37
25
20
15
10

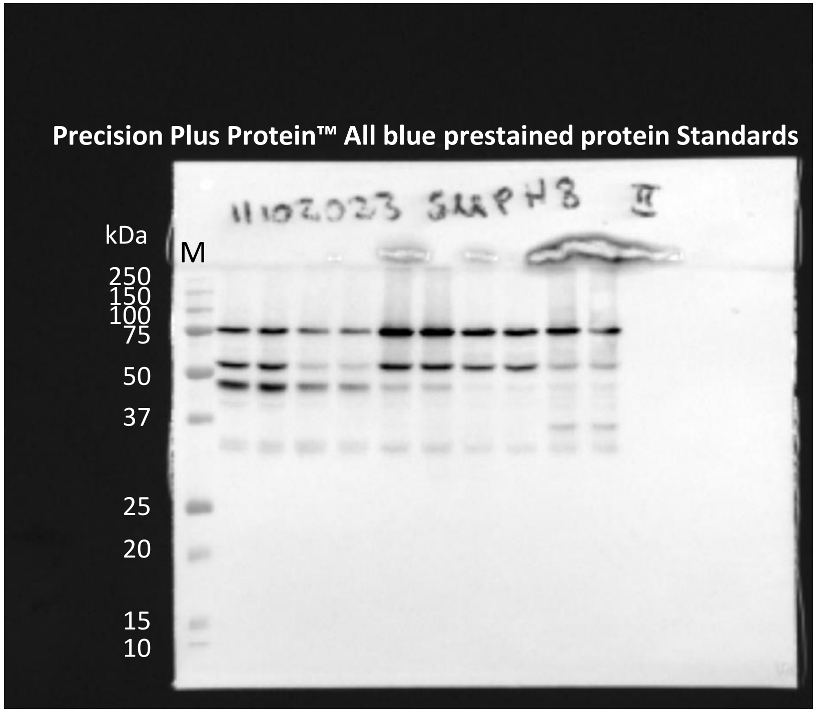
Precision Plus Protein™ All blue prestained protein Standards
kDa
M
250
150
100
75
50
37
25
20
15
10

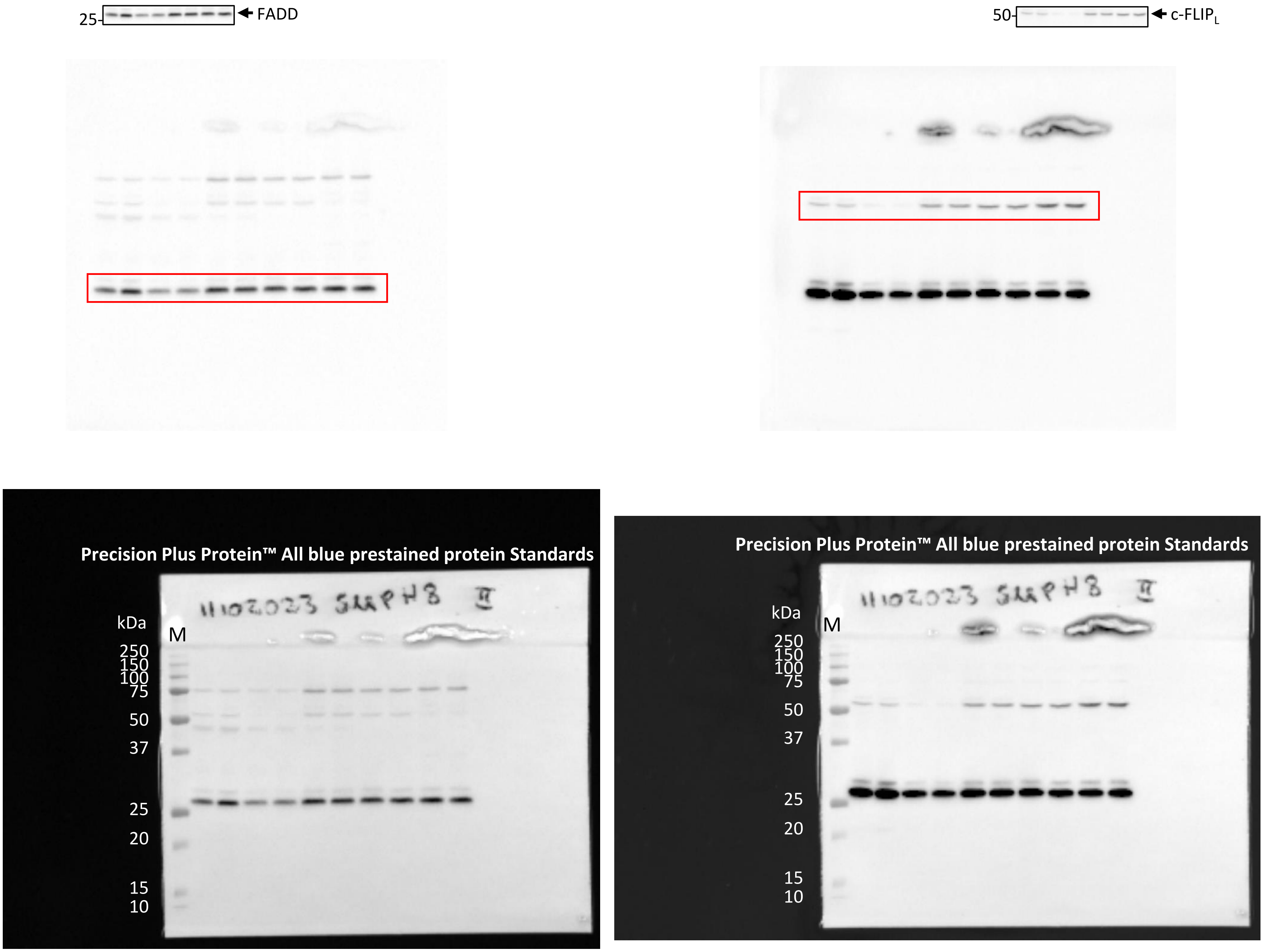

25-
FADD
50-
c-FLIP$_L$
Precision Plus Protein™ All blue prestained protein Standards
kDa
M
250
150
100
75
50
37
25
20
15
10
Precision Plus Protein™ All blue prestained protein Standards
kDa
M
250
150
100
75
50
37
25
20
15
10

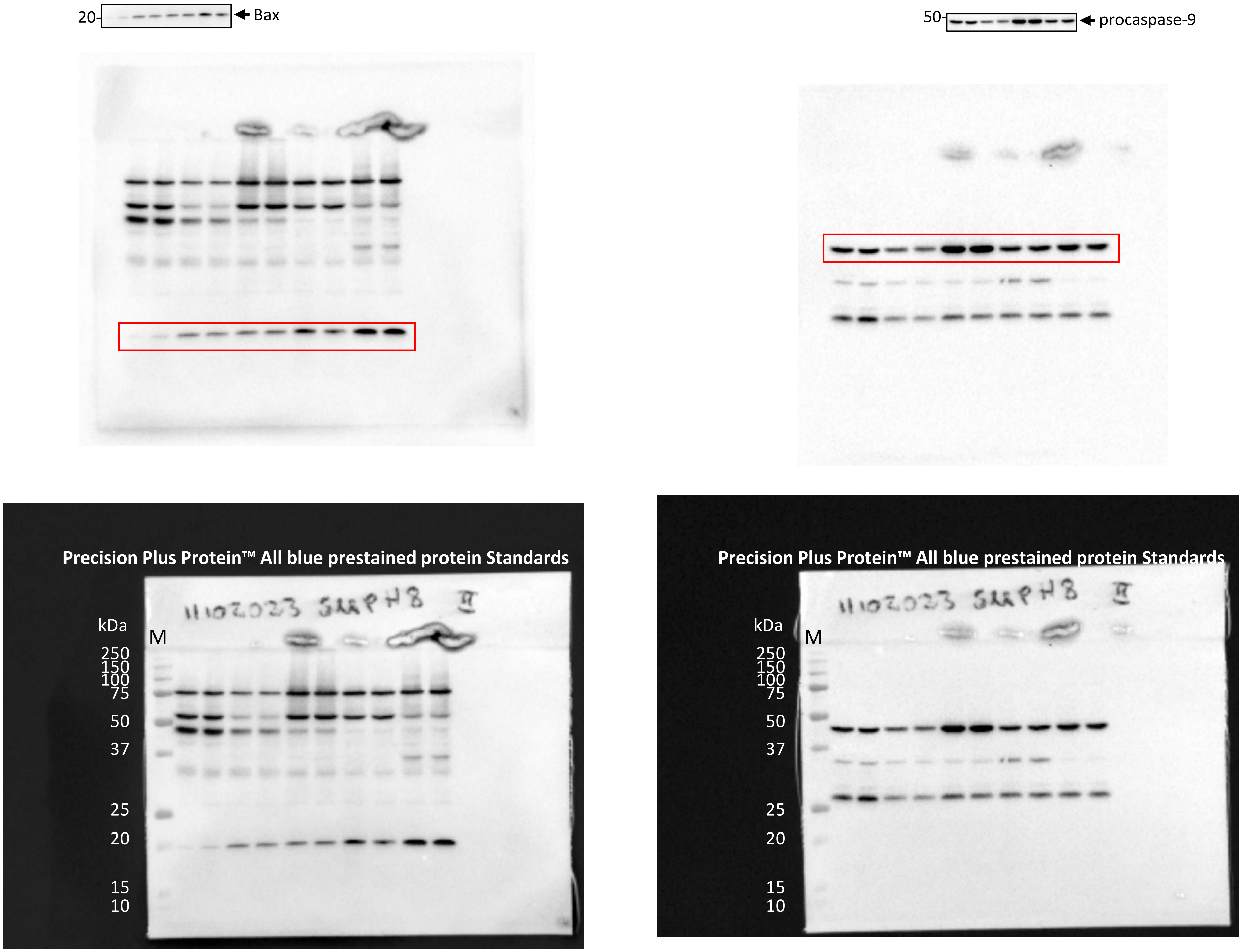
20
Bax
50
procaspase-9
Precision Plus Protein™ All blue prestained protein Standards
kDa
M
250
150
100
75
50
37
25
20
15
10
Precision Plus Protein™ All blue prestained protein Standards
kDa
M
250
150
100
75
50
37
25
20
15
10

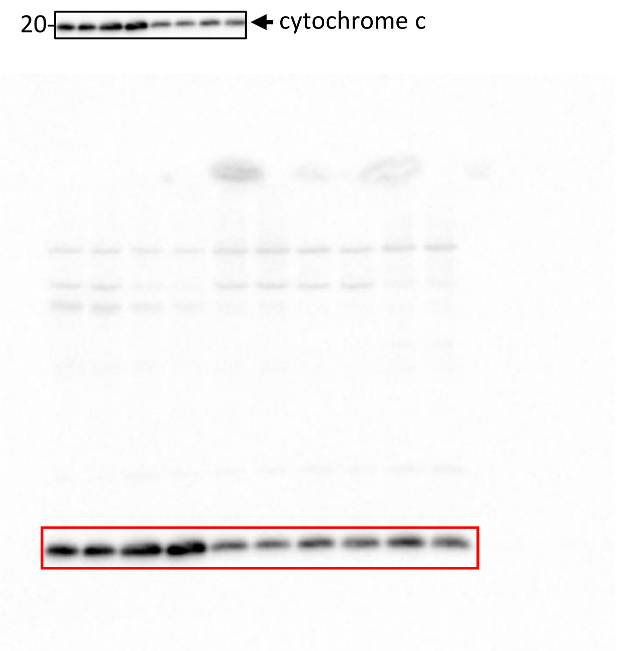
20
cytochrome c

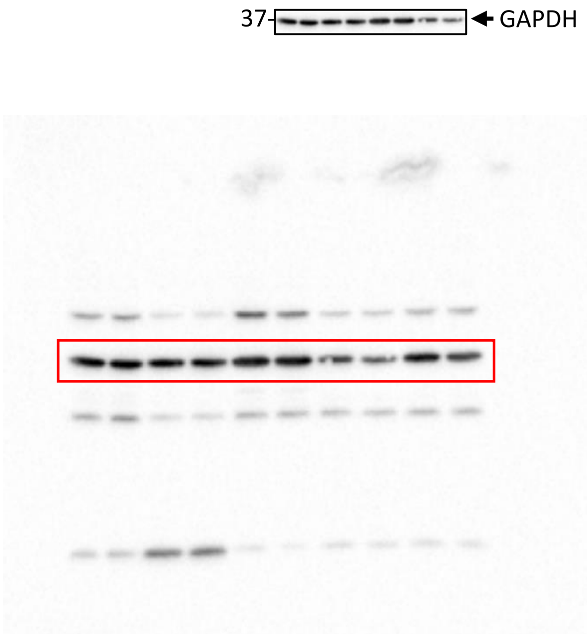
37
GAPDH

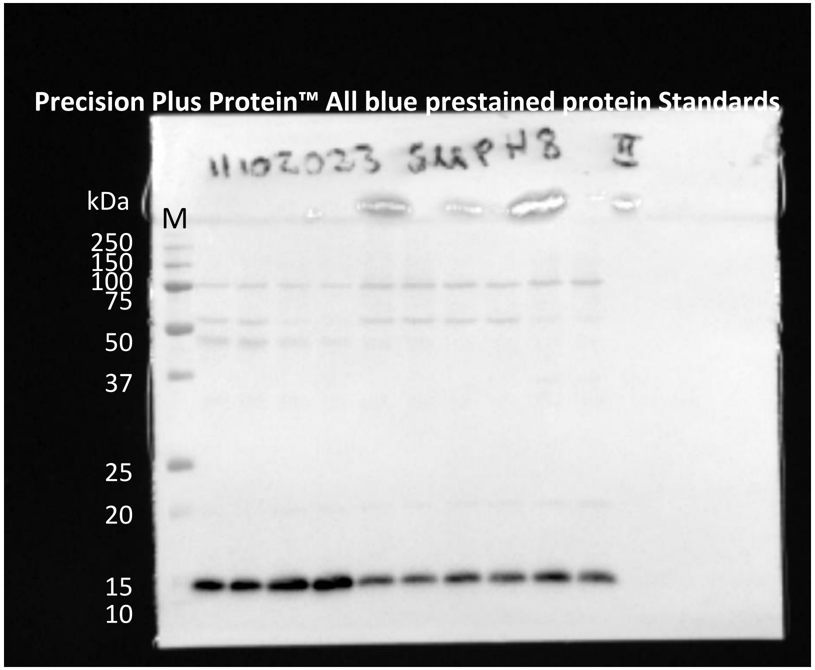
Precision Plus Protein™ All blue prestained protein Standards
kDa
M
250
150
100
75
50
37
25
20
15
10

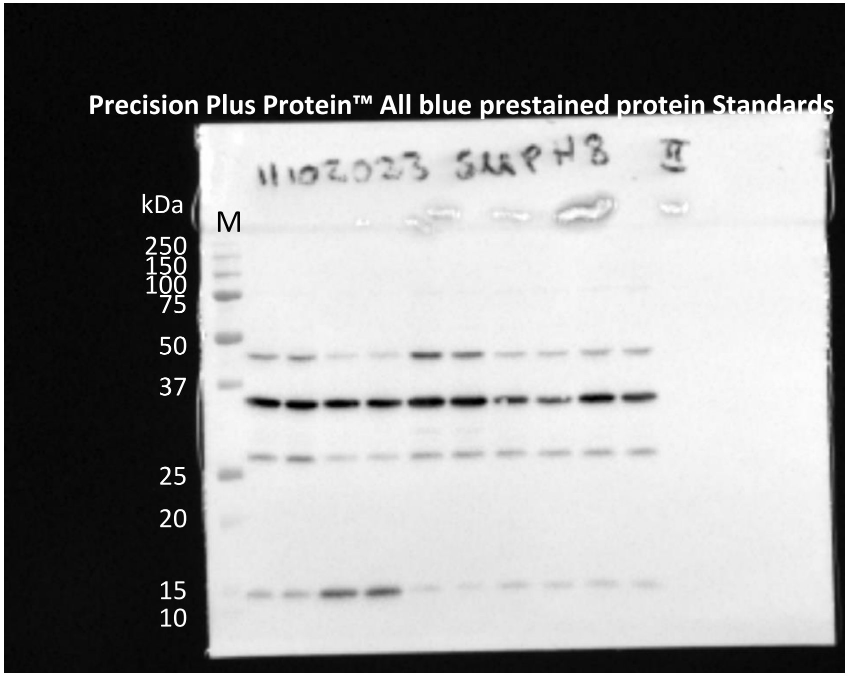
Precision Plus Protein™ All blue prestained protein Standards
kDa
M
250
150
100
75
50
37
25
20
15
10

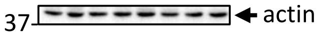
37
actin

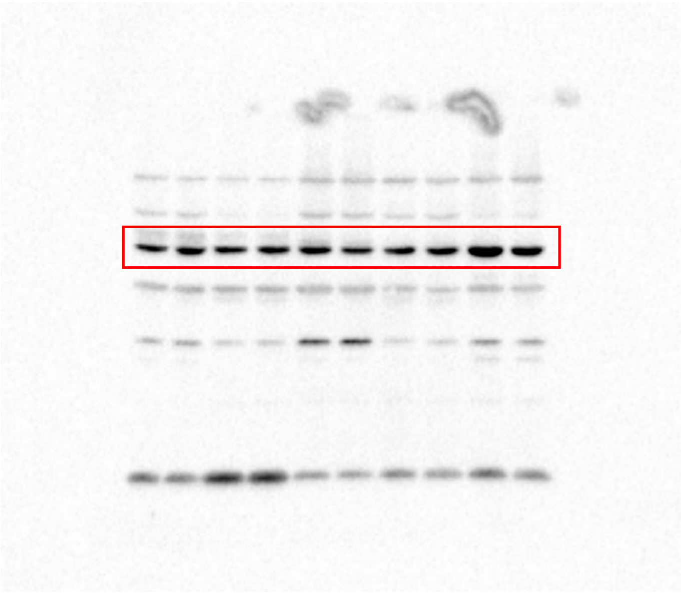

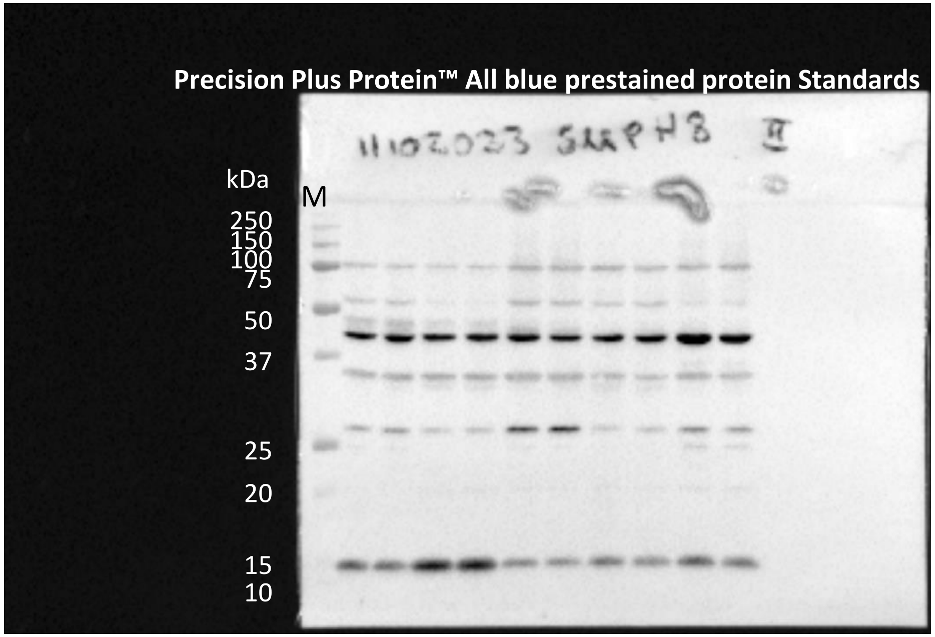
Precision Plus Protein™ All blue prestained protein Standards
kDa
M
250
150
100
75
50
37
25
20
15
10

7d

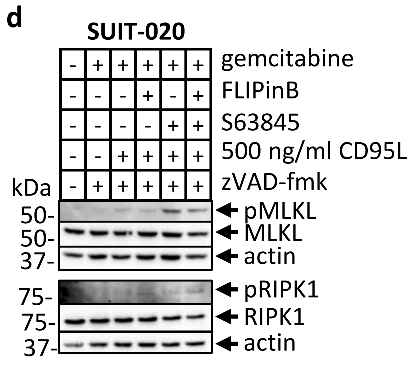
d
SUIT-020
gemcitabine
FLIPinB
S63845
500 ng/ml CD95L
zVAD-fmk
kDa
50-
50-
37-
75-
75-
37-
pMLKL
MLKL
actin
pRIPK1
RIPK1
actin

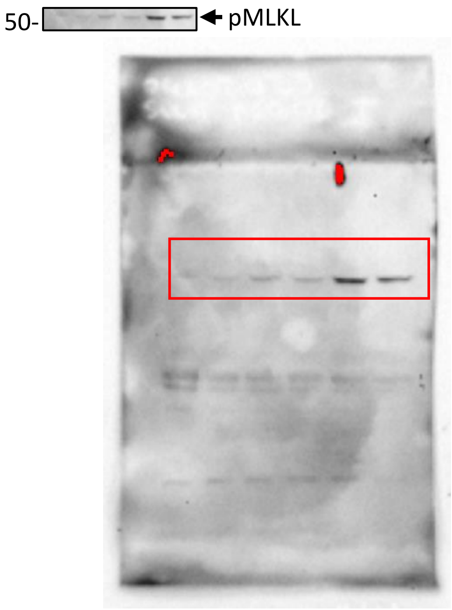
50-
pMLKL

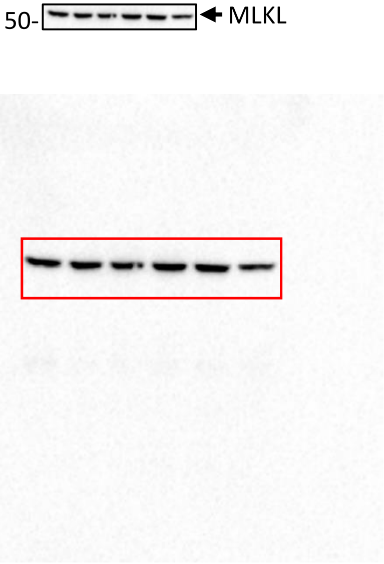
50-
MLKL

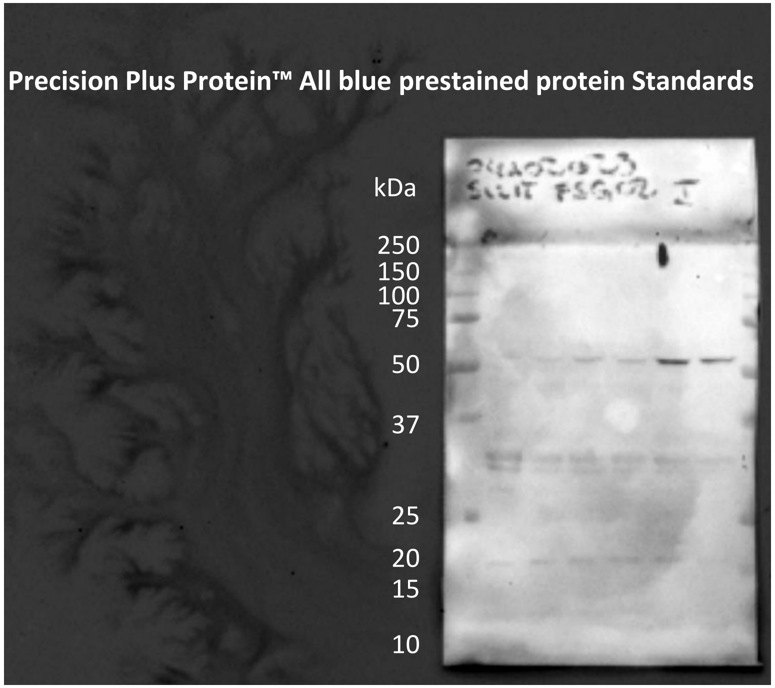
Precision Plus Protein™ All blue prestained protein Standards
kDa
250
150
100
75
50
37
25
20
15
10

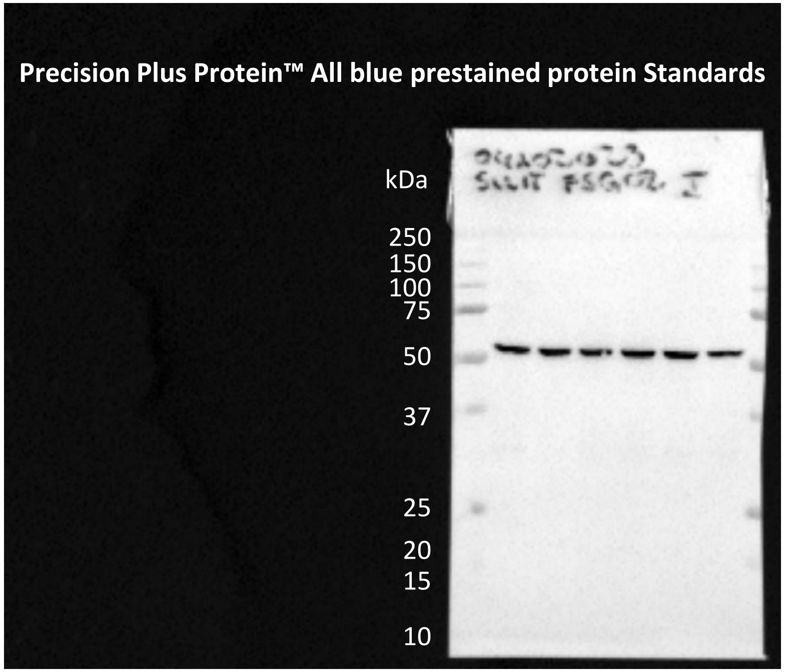
Precision Plus Protein™ All blue prestained protein Standards
kDa
250
150
100
75
50
37
25
20
15
10

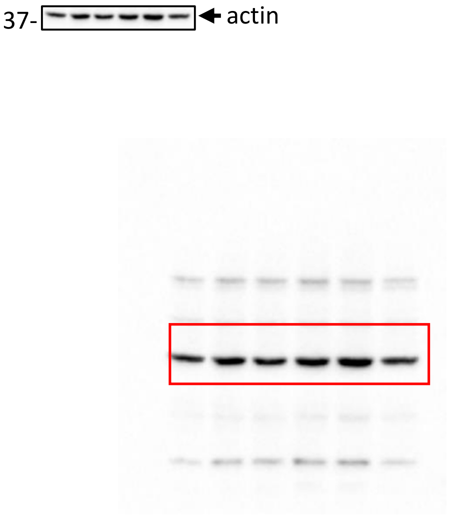
37-
actin

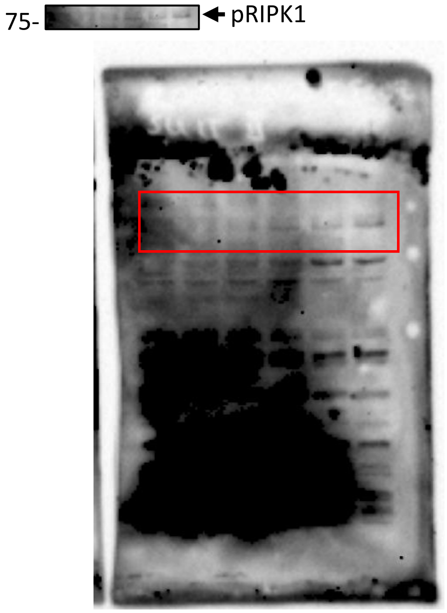
75-
pRIPK1

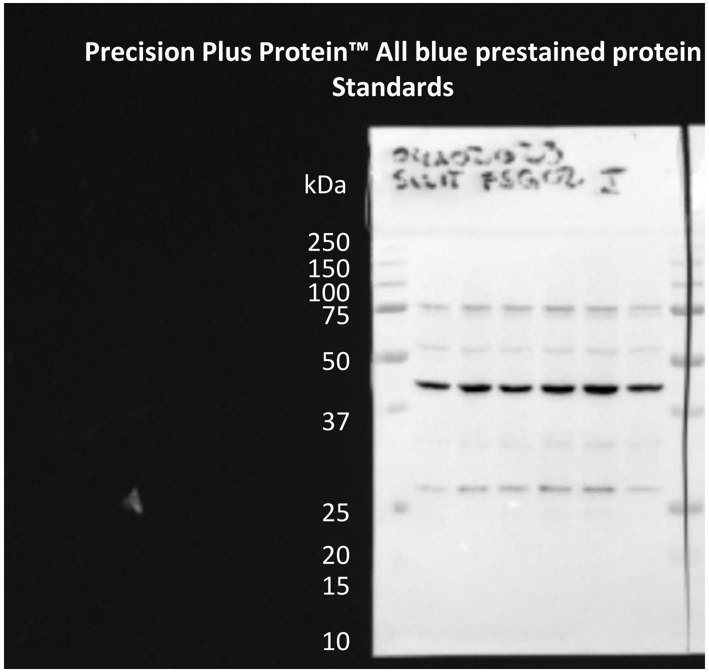
Precision Plus Protein™ All blue prestained protein Standards
kDa
250
150
100
75
50
37
25
20
15
10

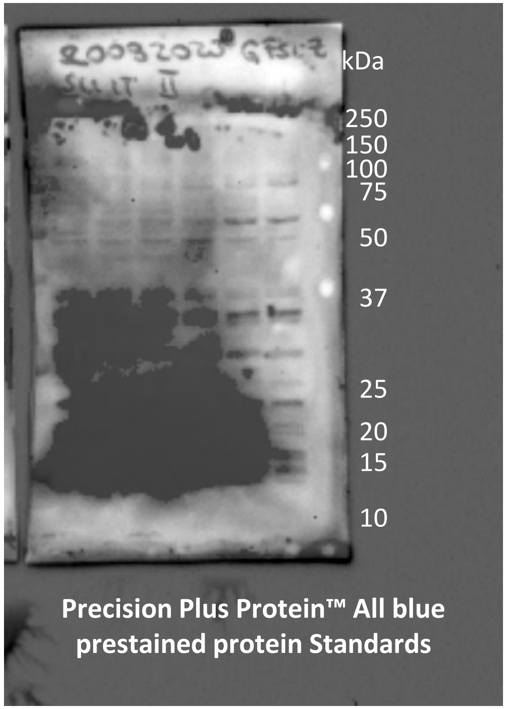
kDa
250
150
100
75
50
37
25
20
15
10
Precision Plus Protein™ All blue prestained protein Standards

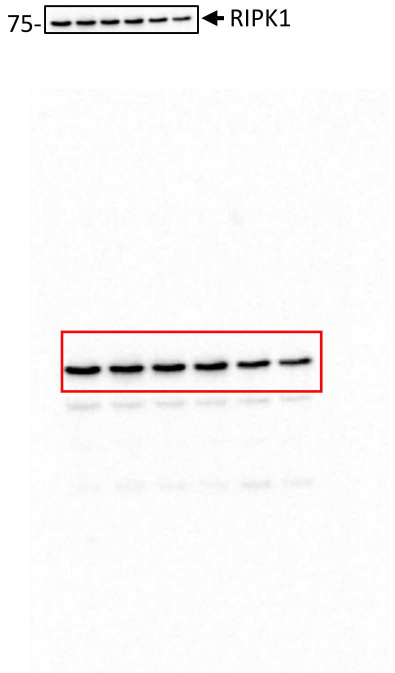
75-
RIPK1

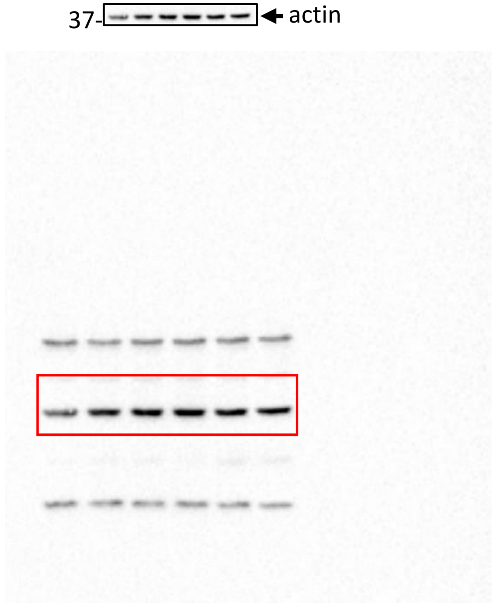
37-
actin

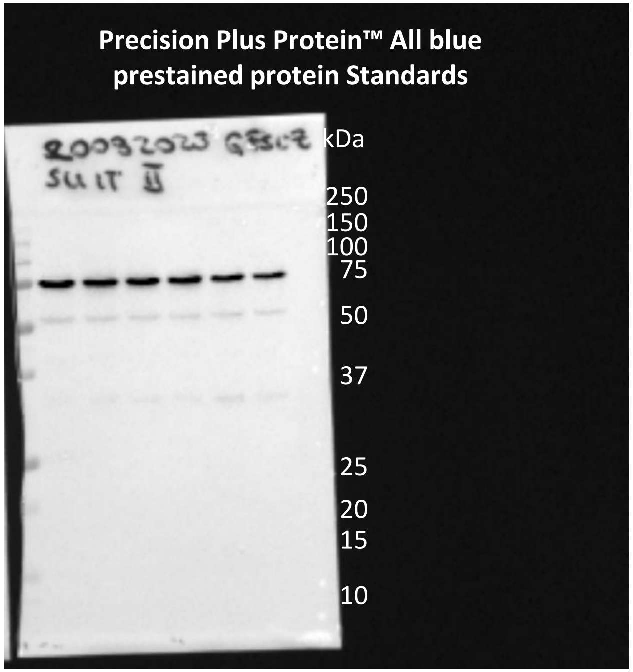
Precision Plus Protein™ All blue
prestained protein Standards
kDa
250
150
100
75
50
37
25
20
15
10

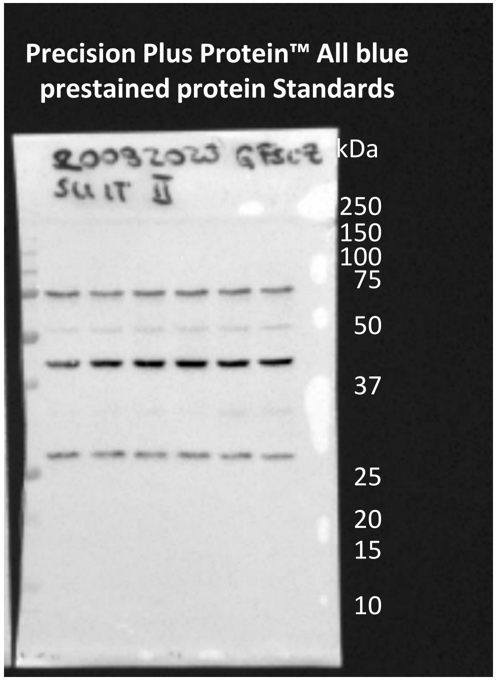
Precision Plus Protein™ All blue
prestained protein Standards
kDa
250
150
100
75
50
37
25
20
15
10

7e

e

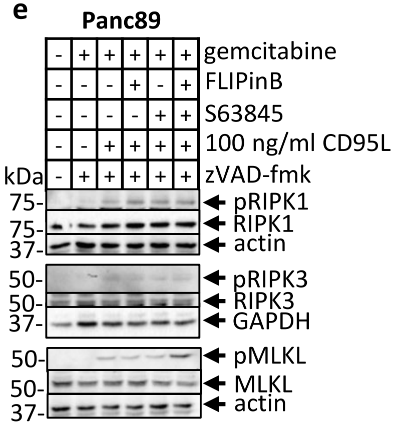

Panc89
gemcitabine
FLIPinB
S63845
100 ng/ml CD95L
zVAD-fmk
kDa
75-
pRIPK1
75-
RIPK1
37-
actin
50-
pRIPK3
50-
RIPK3
37-
GAPDH
50-
pMLKL
50-
MLKL
37-
actin

f

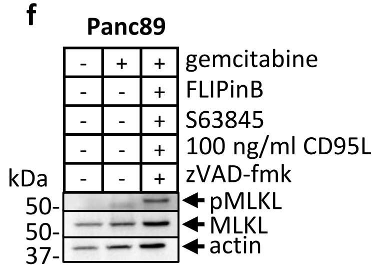

Panc89
gemcitabine
FLIPinB
S63845
100 ng/ml CD95L
zVAD-fmk
kDa
50-
pMLKL
50-
MLKL
37-
actin

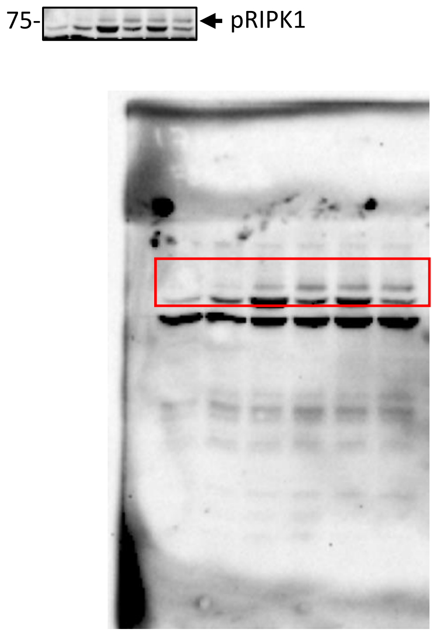
75-
pRIPK1

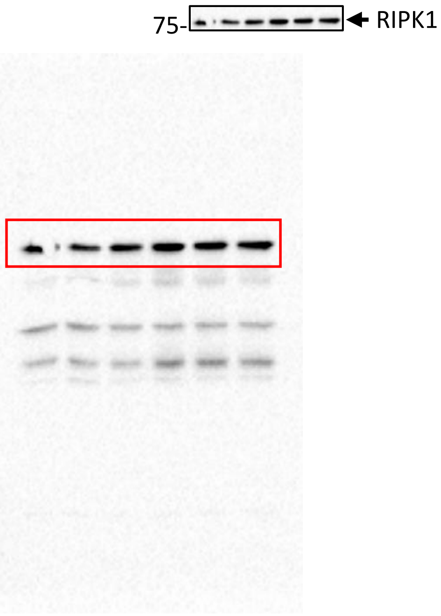
75-
RIPK1

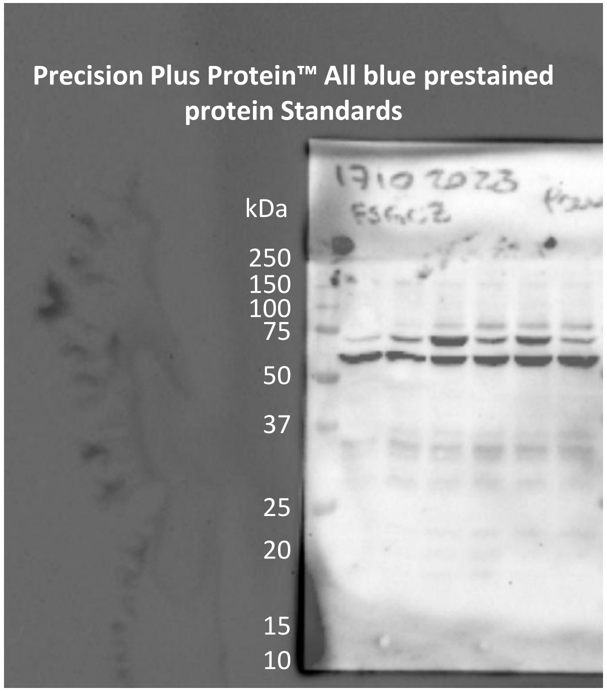
Precision Plus Protein™ All blue prestained protein Standards
kDa
250
150
100
75
50
37
25
20
15
10

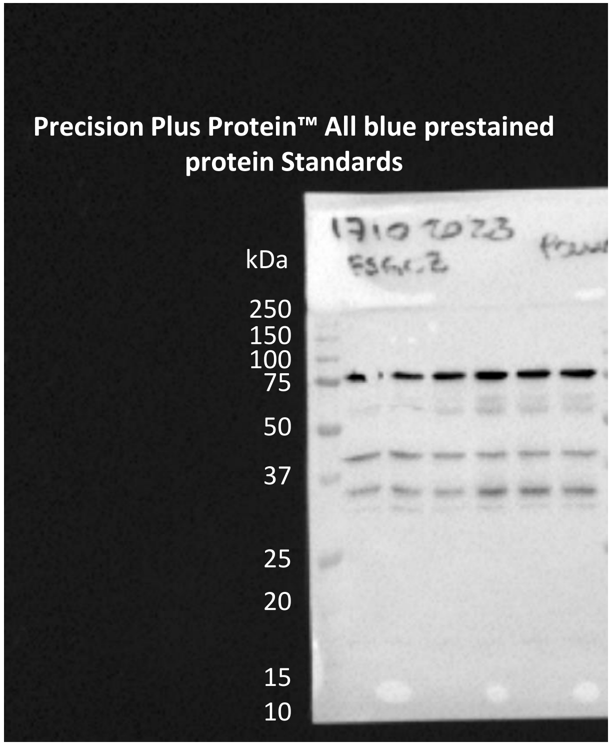
Precision Plus Protein™ All blue prestained protein Standards
kDa
250
150
100
75
50
37
25
20
15
10

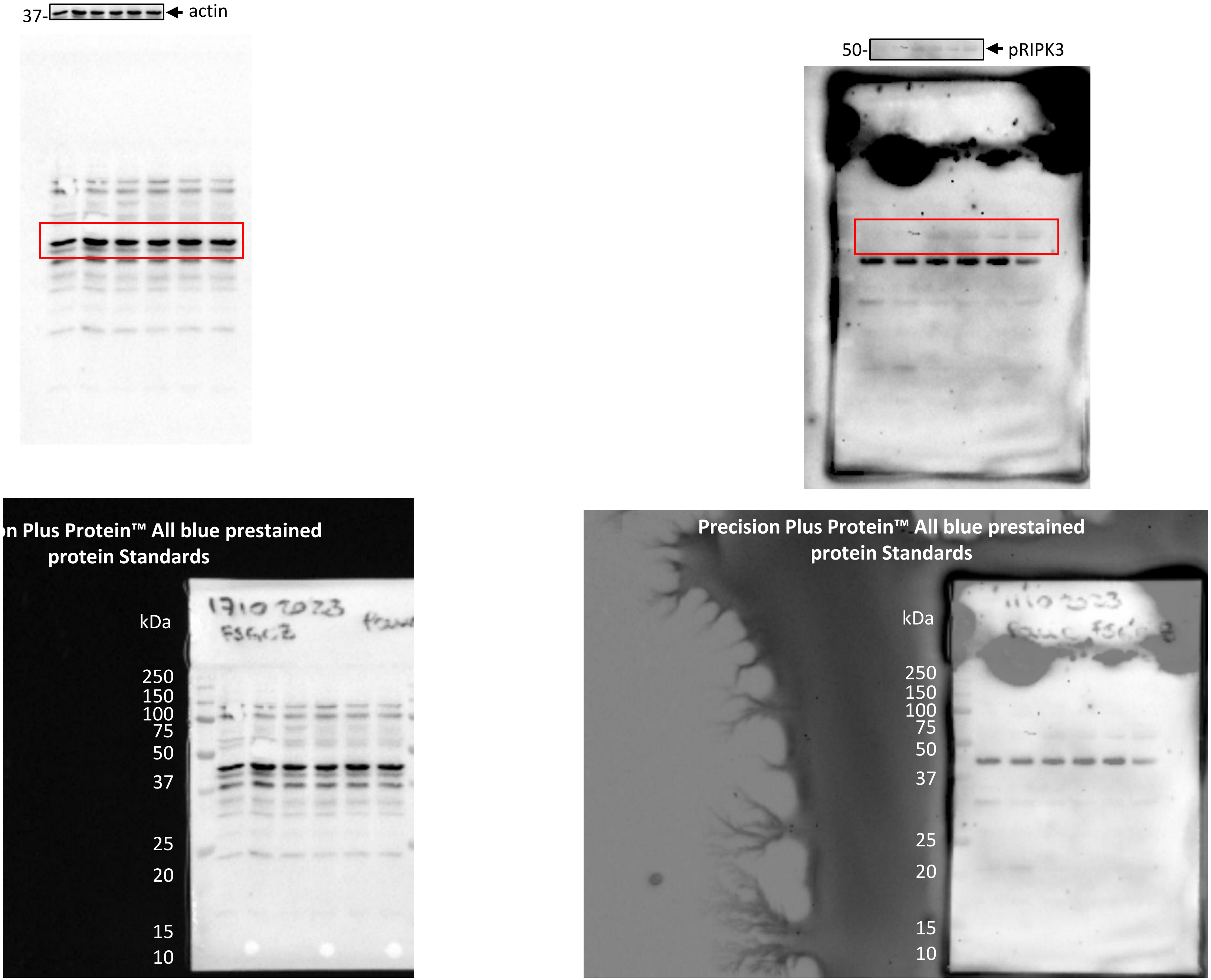
37-
actin
50-
pRIPK3
n Plus Protein™ All blue prestained
protein Standards
kDa
250
150
100
75
50
37
25
20
15
10
Precision Plus Protein™ All blue prestained
protein Standards
kDa
250
150
100
75
50
37
25
20
15
10

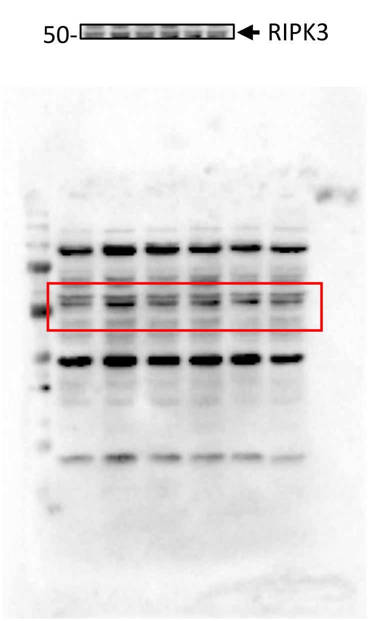
50-
RIPK3

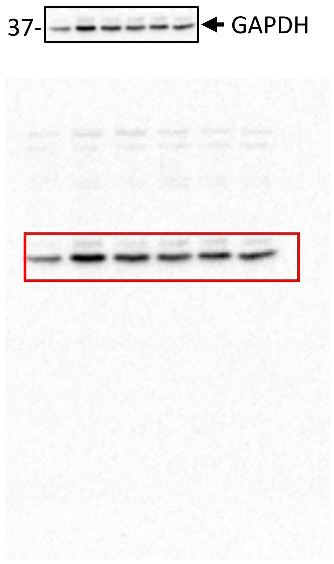
37-
GAPDH

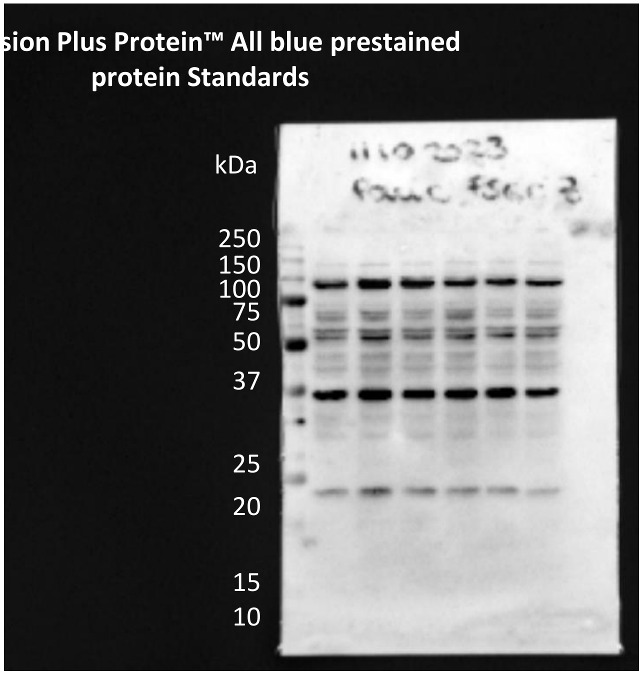
sion Plus Protein™ All blue prestained
protein Standards
kDa
250
150
100
75
50
37
25
20
15
10

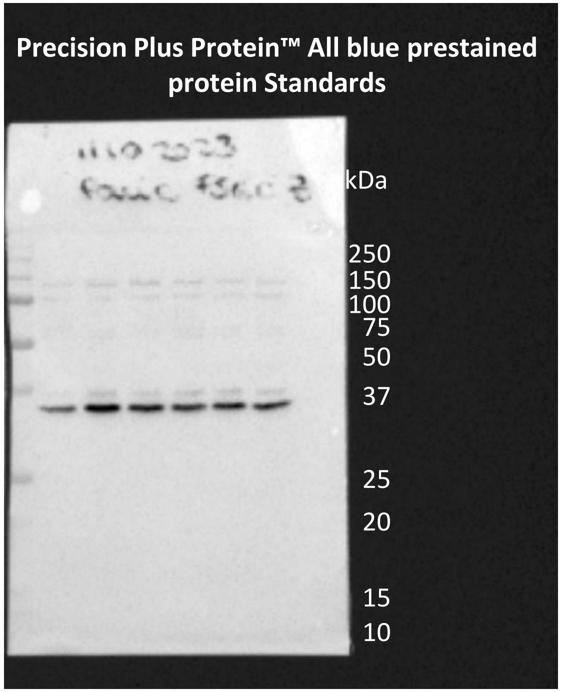
Precision Plus Protein™ All blue prestained
protein Standards
kDa
250
150
100
75
50
37
25
20
15
10

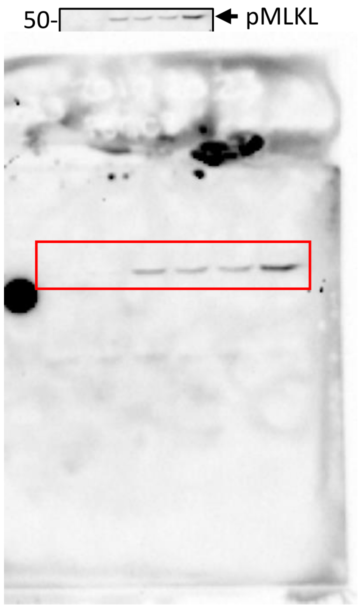
50-
pMLKL

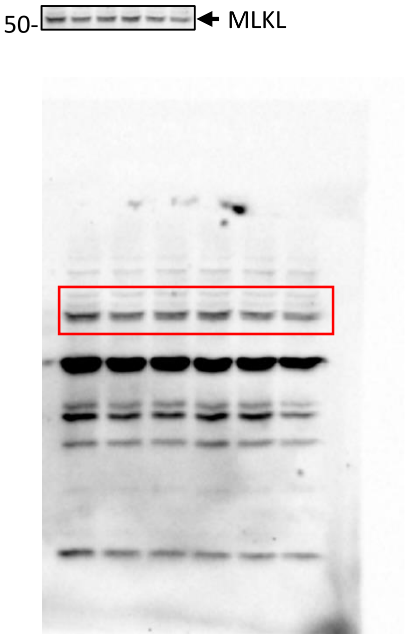
50-
MLKL

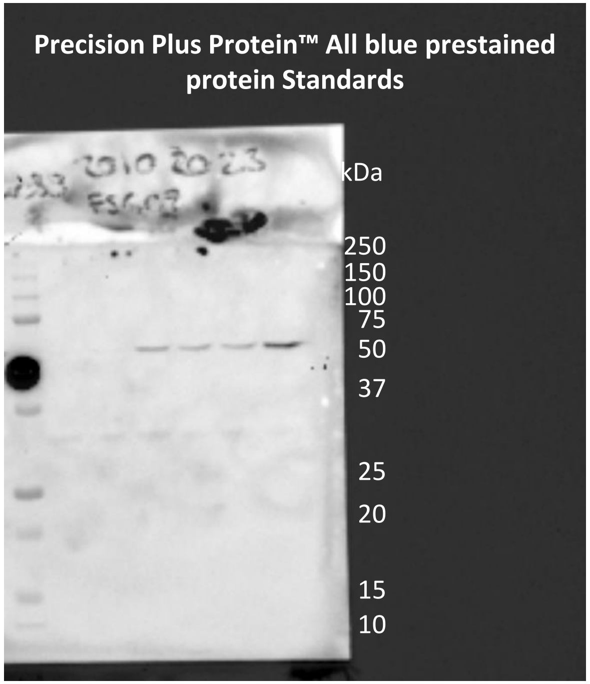
Precision Plus Protein™ All blue prestained protein Standards
kDa
250
150
100
75
50
37
25
20
15
10

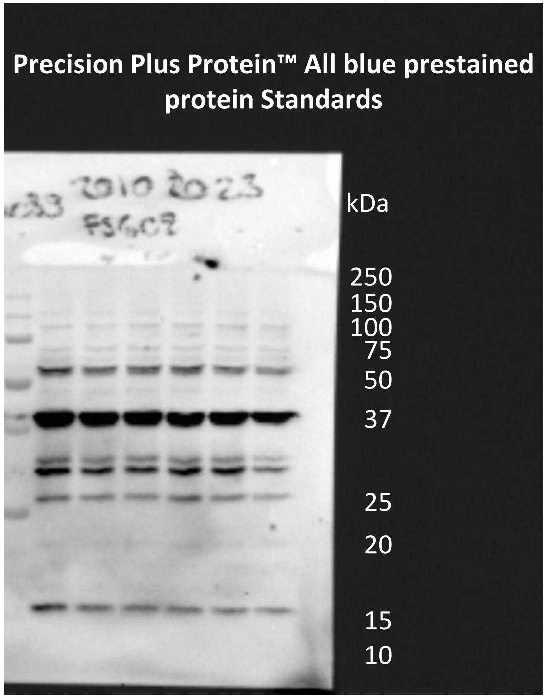
Precision Plus Protein™ All blue prestained protein Standards
kDa
250
150
100
75
50
37
25
20
15
10

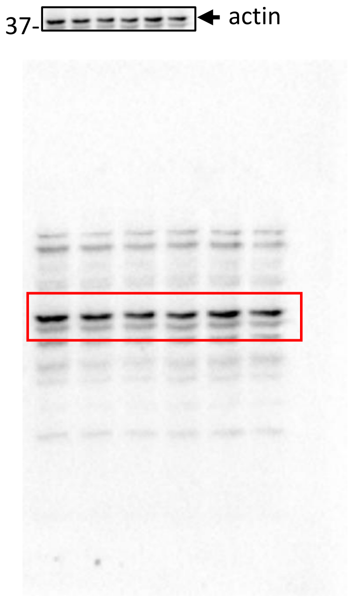
37-
actin

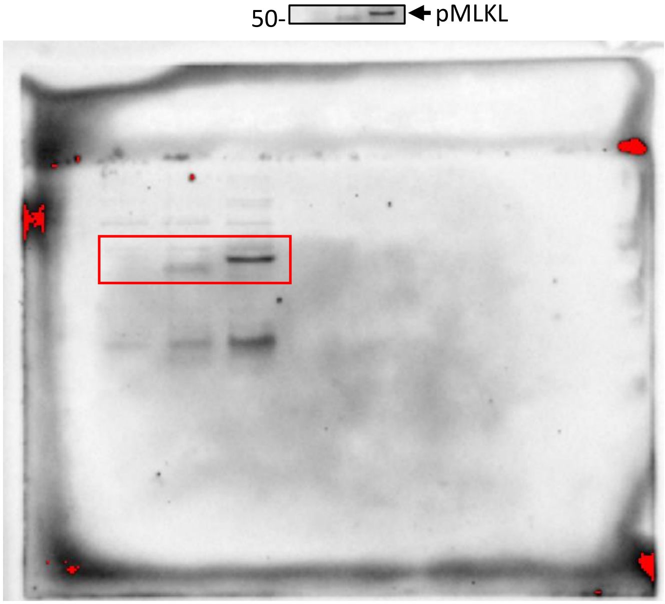
50-
pMLKL

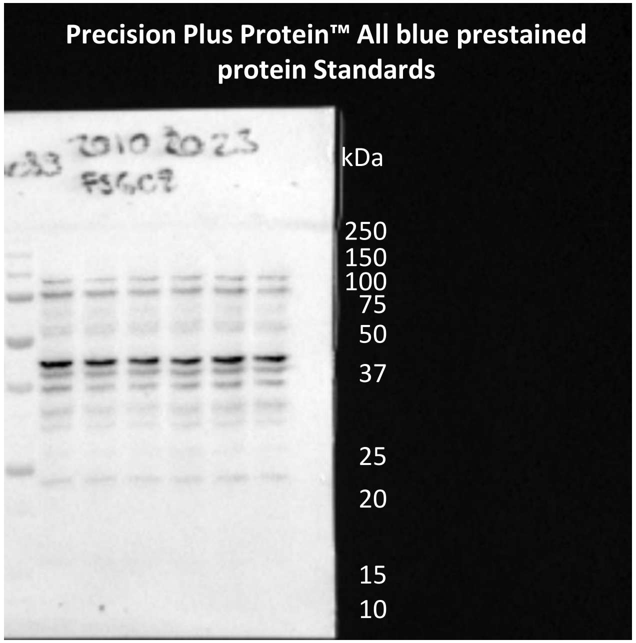
Precision Plus Protein™ All blue prestained protein Standards
kDa
250
150
100
75
50
37
25
20
15
10

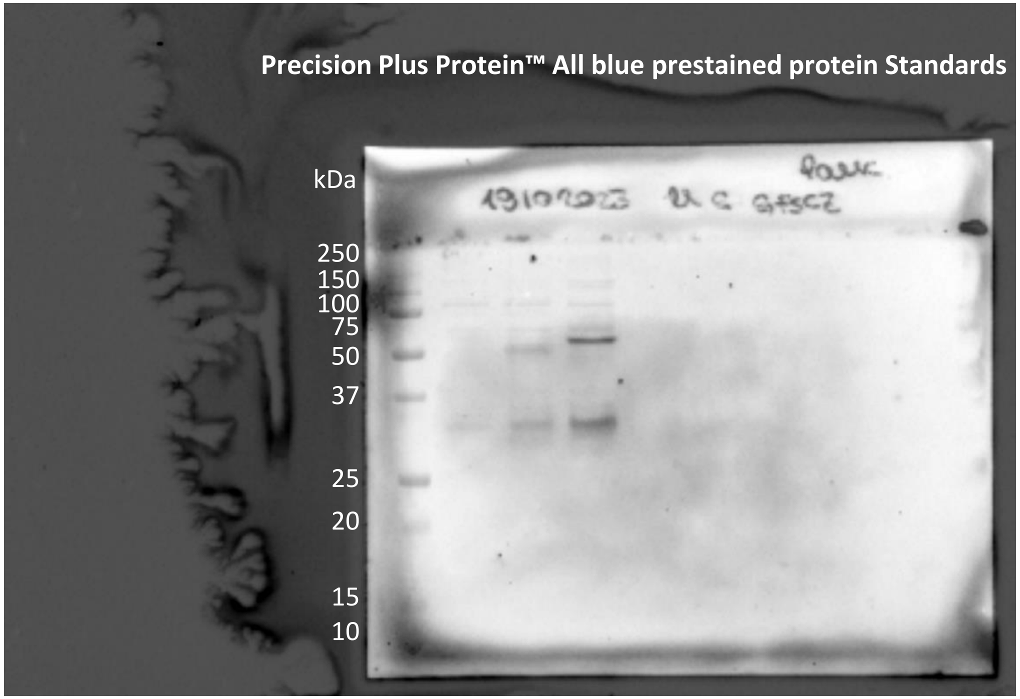
Precision Plus Protein™ All blue prestained protein Standards
kDa
250
150
100
75
50
37
25
20
15
10

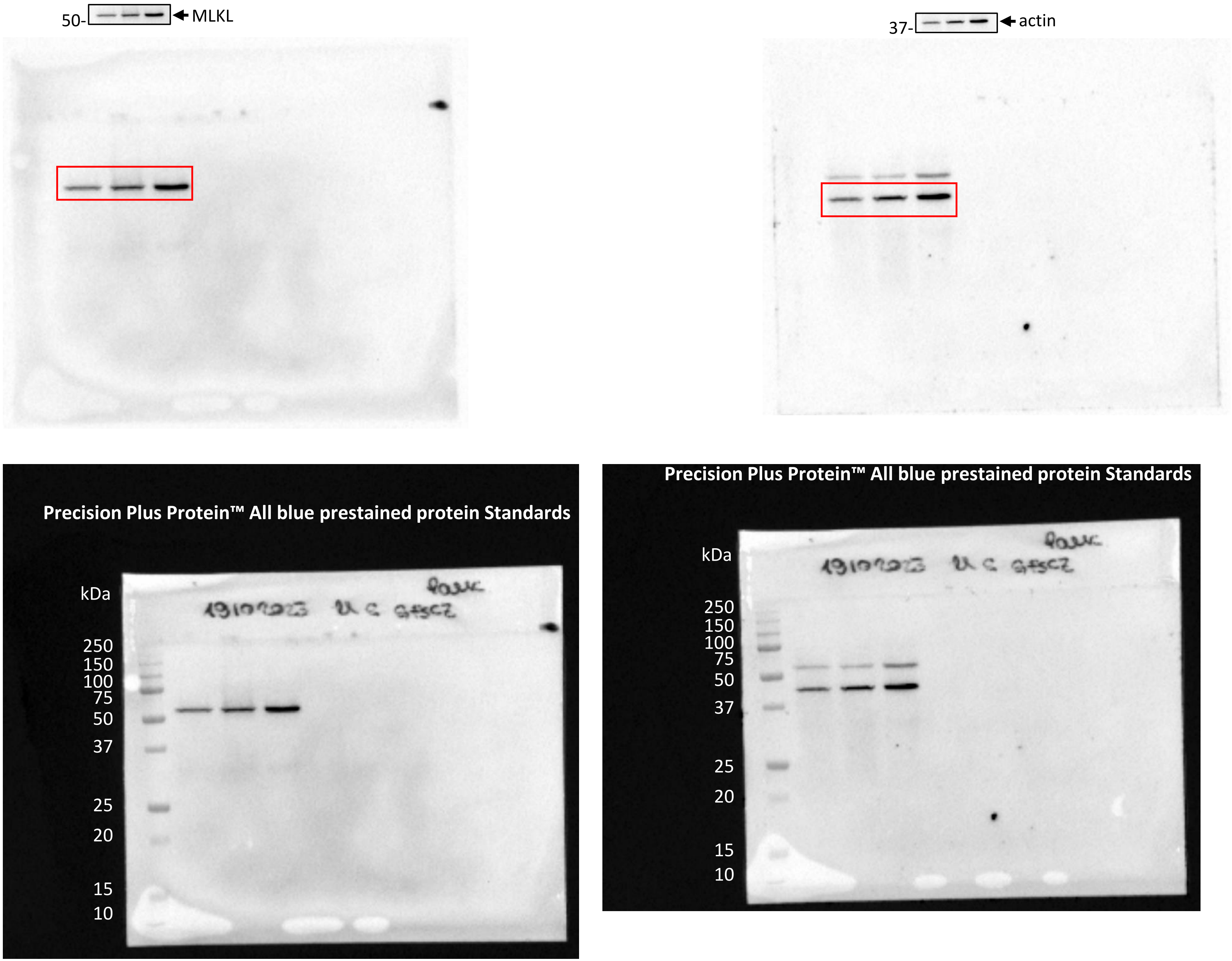
50-
MLKL
37-
actin
Precision Plus Protein™ All blue prestained protein Standards
kDa
250
150
100
75
50
37
25
20
15
10
Precision Plus Protein™ All blue prestained protein Standards
kDa
250
150
100
75
50
37
25
20
15
10

8a

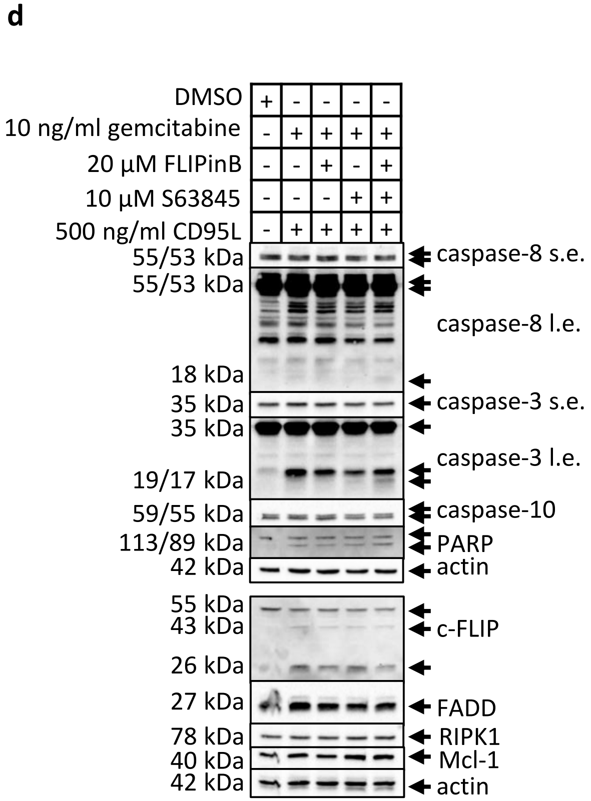
d
DMSO
10 ng/ml gemcitabine
20 µM FLIPinB
10 µM S63845
500 ng/ml CD95L
+ - - - -
- + + + +
- - + - +
- - - + +
- + + + +
55/53 kDa
caspase-8 s.e.
55/53 kDa
caspase-8 l.e.
18 kDa
35 kDa
caspase-3 s.e.
35 kDa
caspase-3 l.e.
19/17 kDa
59/55 kDa
caspase-10
113/89 kDa
PARP
42 kDa
actin
55 kDa
43 kDa
c-FLIP
26 kDa
27 kDa
FADD
78 kDa
RIPK1
40 kDa
Mcl-1
42 kDa
actin

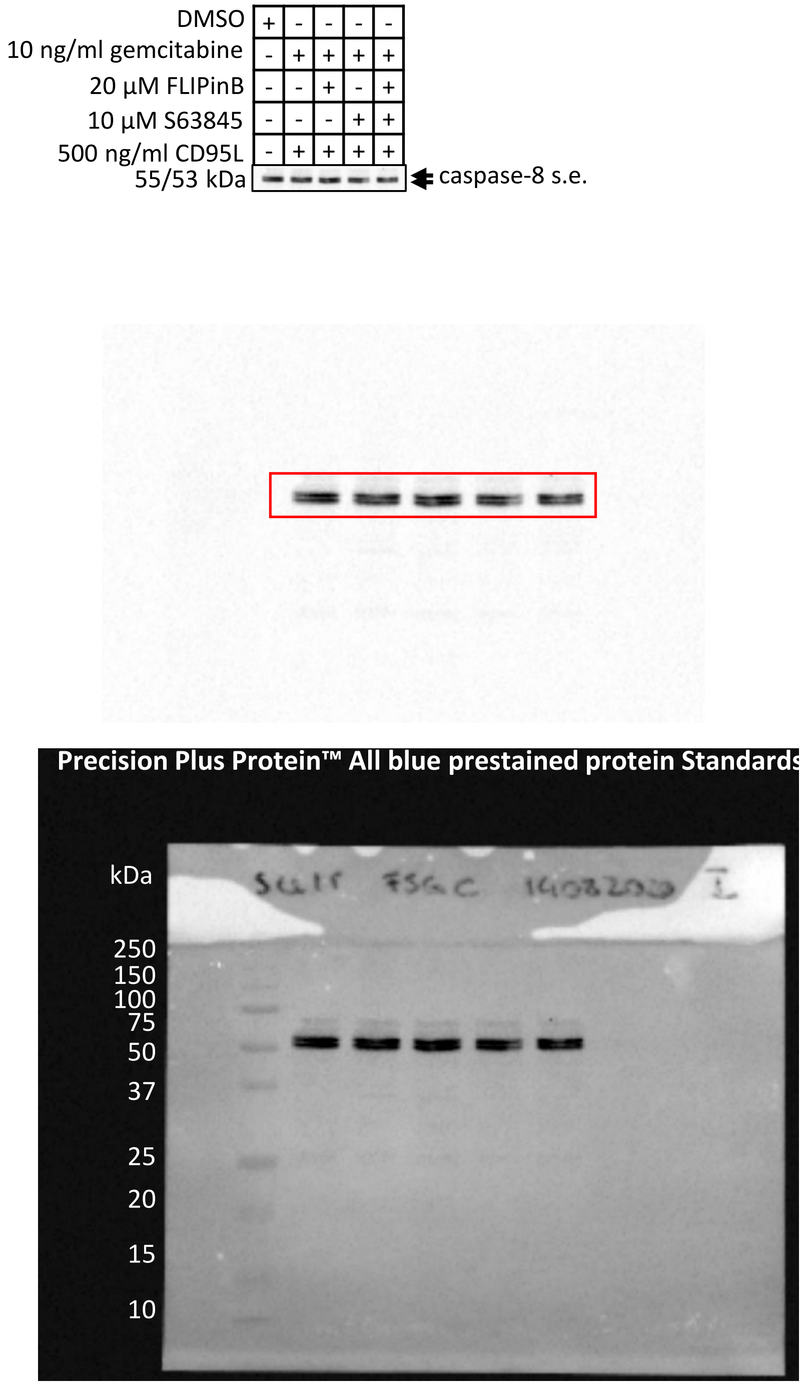
DMSO + - - - -
10 ng/ml gemcitabine - + + + +
20 µM FLIPinB - - + - +
10 µM S63845 - - - + +
500 ng/ml CD95L - + + + +
55/53 kDa
caspase-8 s.e.
Precision Plus Protein™ All blue prestained protein Standards
kDa
250
150
100
75
50
37
25
20
15
10

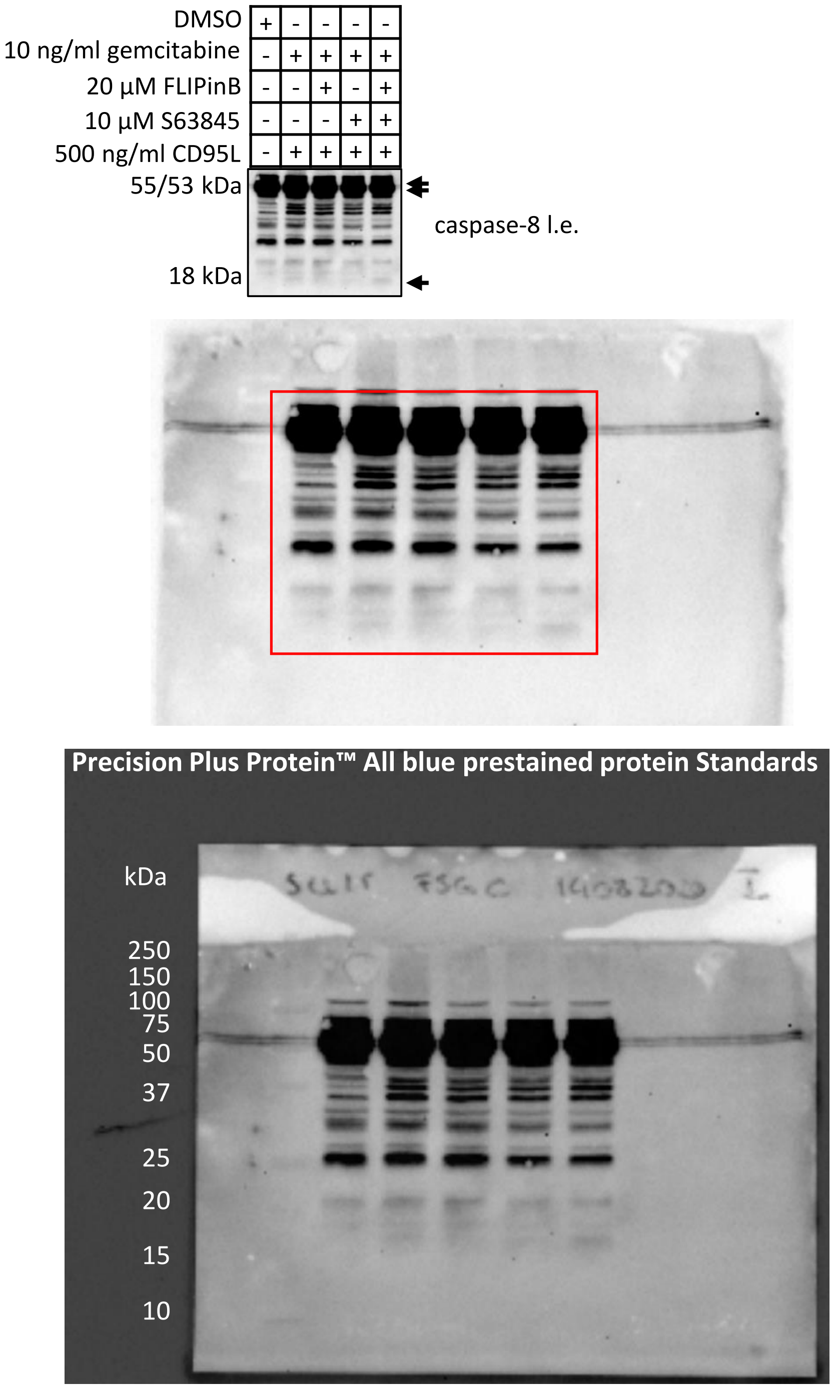
DMSO + - - - -
10 ng/ml gemcitabine - + + + +
20 µM FLIPinB - - + - +
10 µM S63845 - - - + +
500 ng/ml CD95L - + + + +
55/53 kDa
caspase-8 l.e.
18 kDa
Precision Plus Protein™ All blue prestained protein Standards
kDa
250
150
100
75
50
37
25
20
15
10

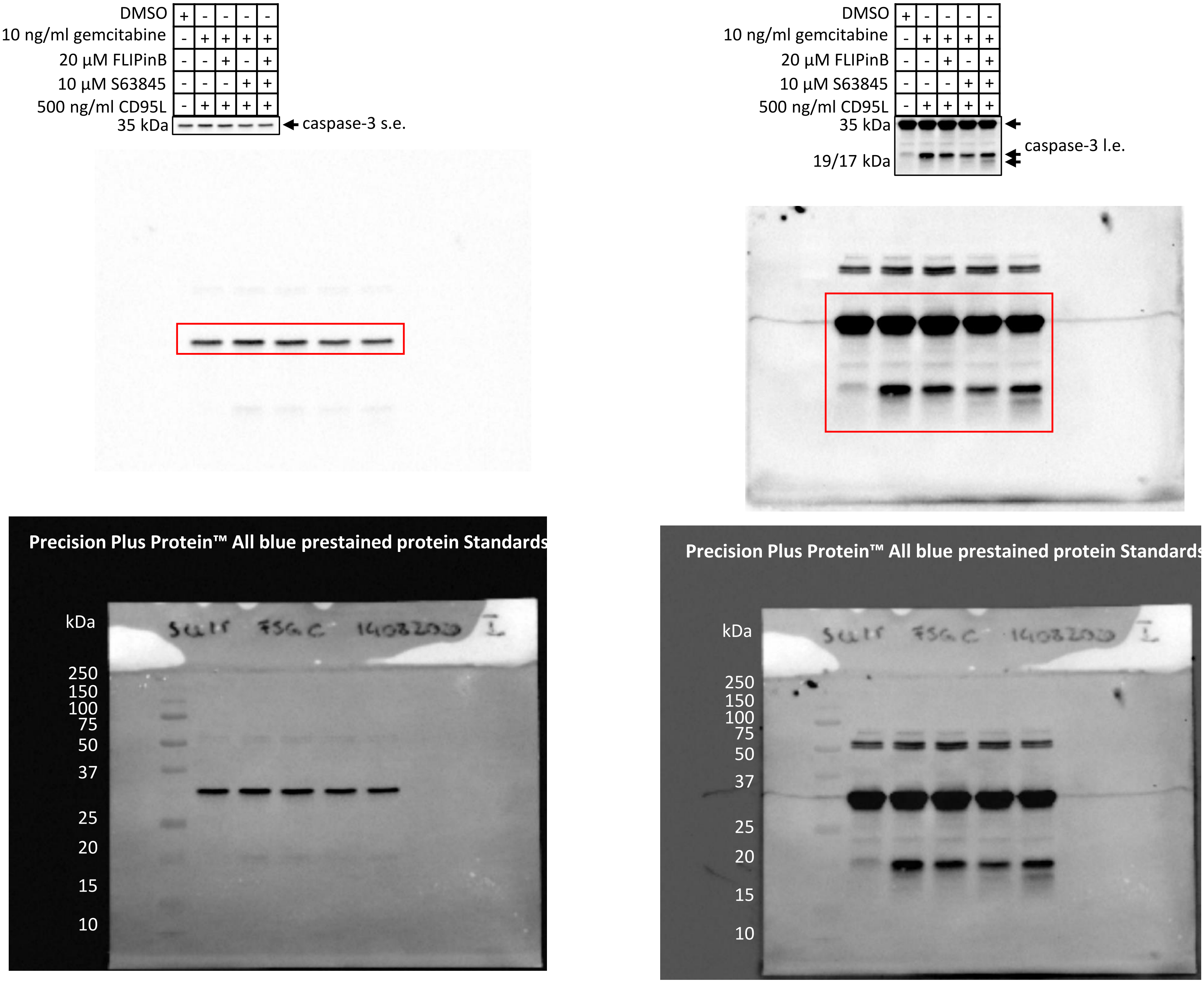
DMSO
10 ng/ml gemcitabine
20 µM FLIPinB
10 µM S63845
500 ng/ml CD95L
35 kDa
caspase-3 s.e.
19/17 kDa
caspase-3 l.e.
Precision Plus Protein™ All blue prestained protein Standards
kDa
250
150
100
75
50
37
25
20
15
10

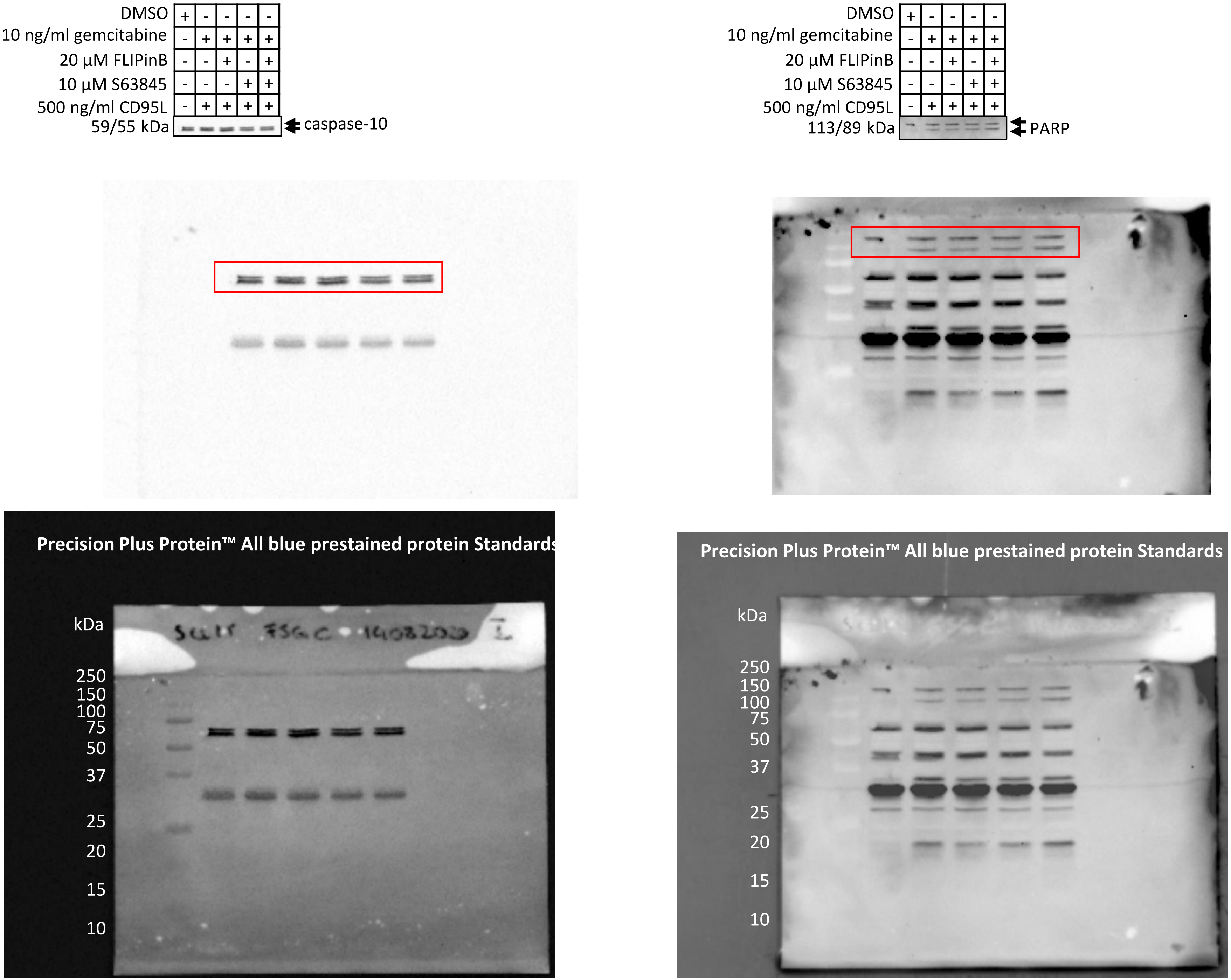
DMSO
10 ng/ml gemcitabine
20 µM FLIPinB
10 µM S63845
500 ng/ml CD95L
59/55 kDa
caspase-10
DMSO
10 ng/ml gemcitabine
20 µM FLIPinB
10 µM S63845
500 ng/ml CD95L
113/89 kDa
PARP
Precision Plus Protein™ All blue prestained protein Standards
kDa
250
150
100
75
50
37
25
20
15
10
Precision Plus Protein™ All blue prestained protein Standards
kDa
250
150
100
75
50
37
25
20
15
10

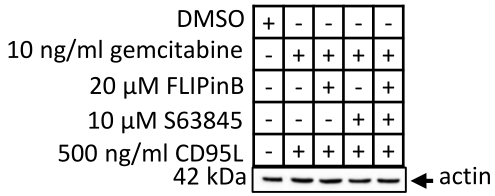
DMSO + - - - -
10 ng/ml gemcitabine - + + + +
20 μM FLIPinB - - + - +
10 μM S63845 - - - + +
500 ng/ml CD95L - + + + +
42 kDa
actin

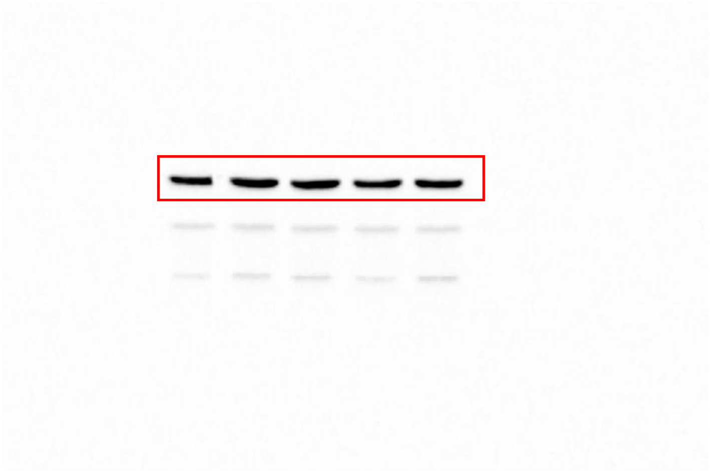

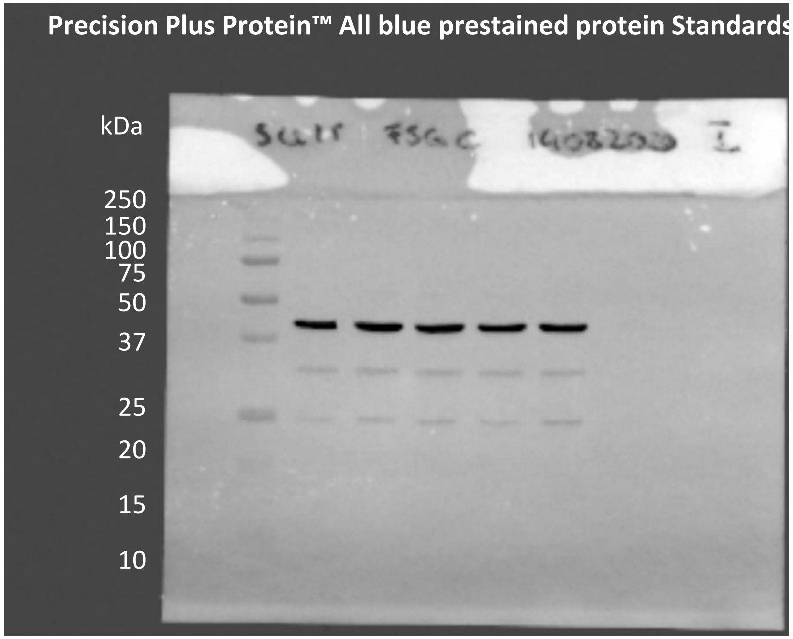
Precision Plus Protein™ All blue prestained protein Standards
kDa
250
150
100
75
50
37
25
20
15
10

DMSO	+	-	-	-	-
10 ng/ml gemcitabine	-	+	+	+	+
20 µM FLIPinB	-	-	+	-	+
10 µM S63845	-	-	-	+	+
500 ng/ml CD95L	-	+	+	+	+

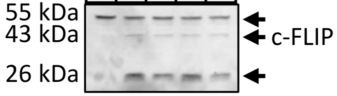

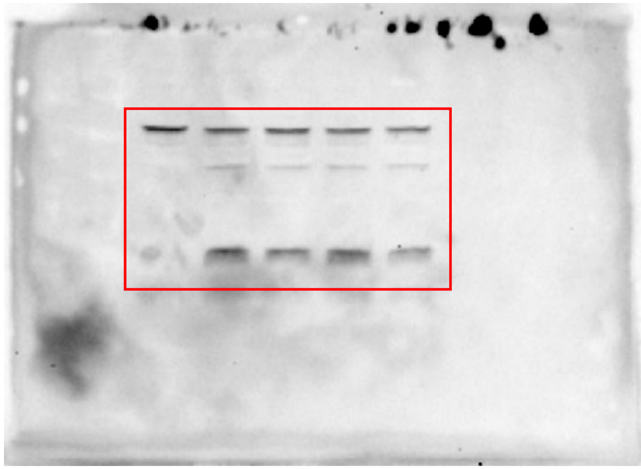

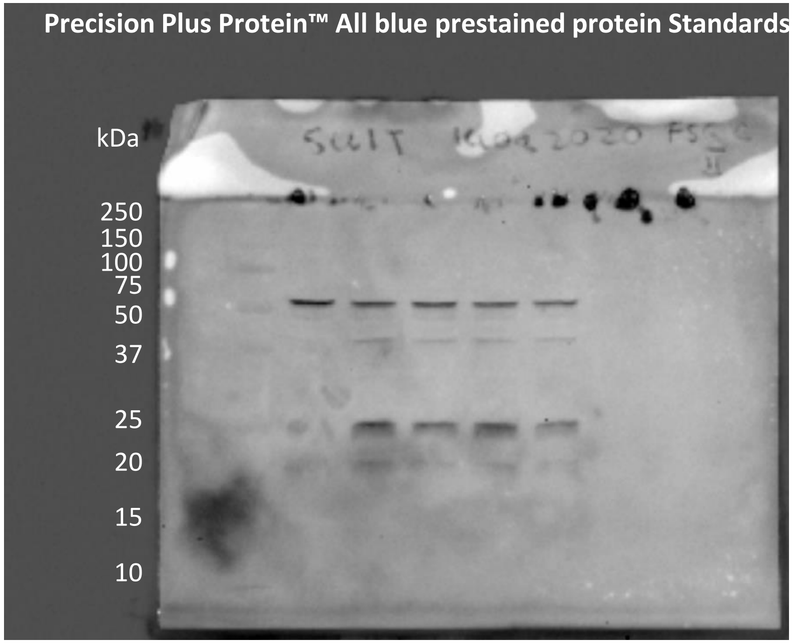

DMSO	+	-	-	-	-
10 ng/ml gemcitabine	-	+	+	+	+
20 µM FLIPinB	-	-	+	-	+
10 µM S63845	-	-	-	+	+
500 ng/ml CD95L	-	+	+	+	+

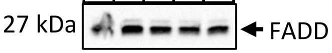

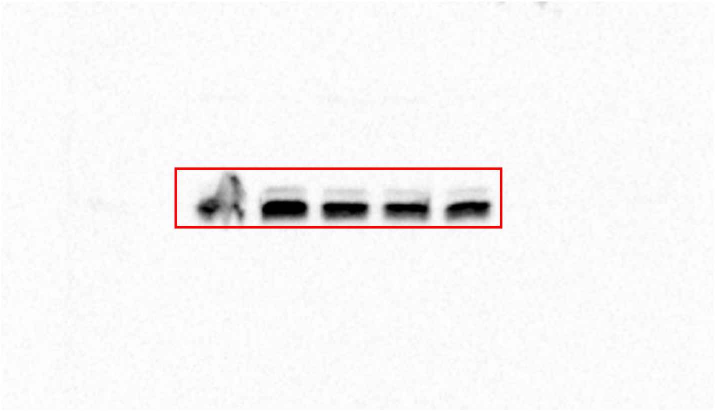

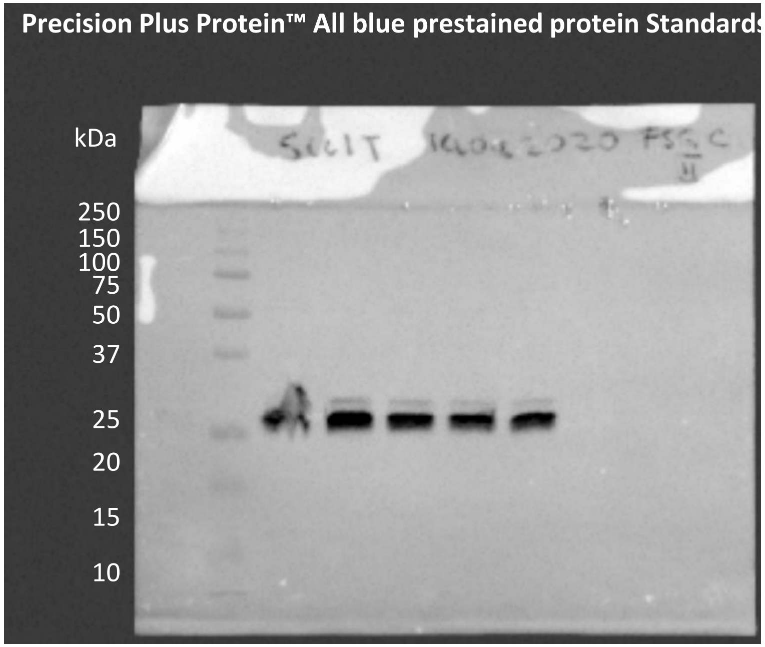

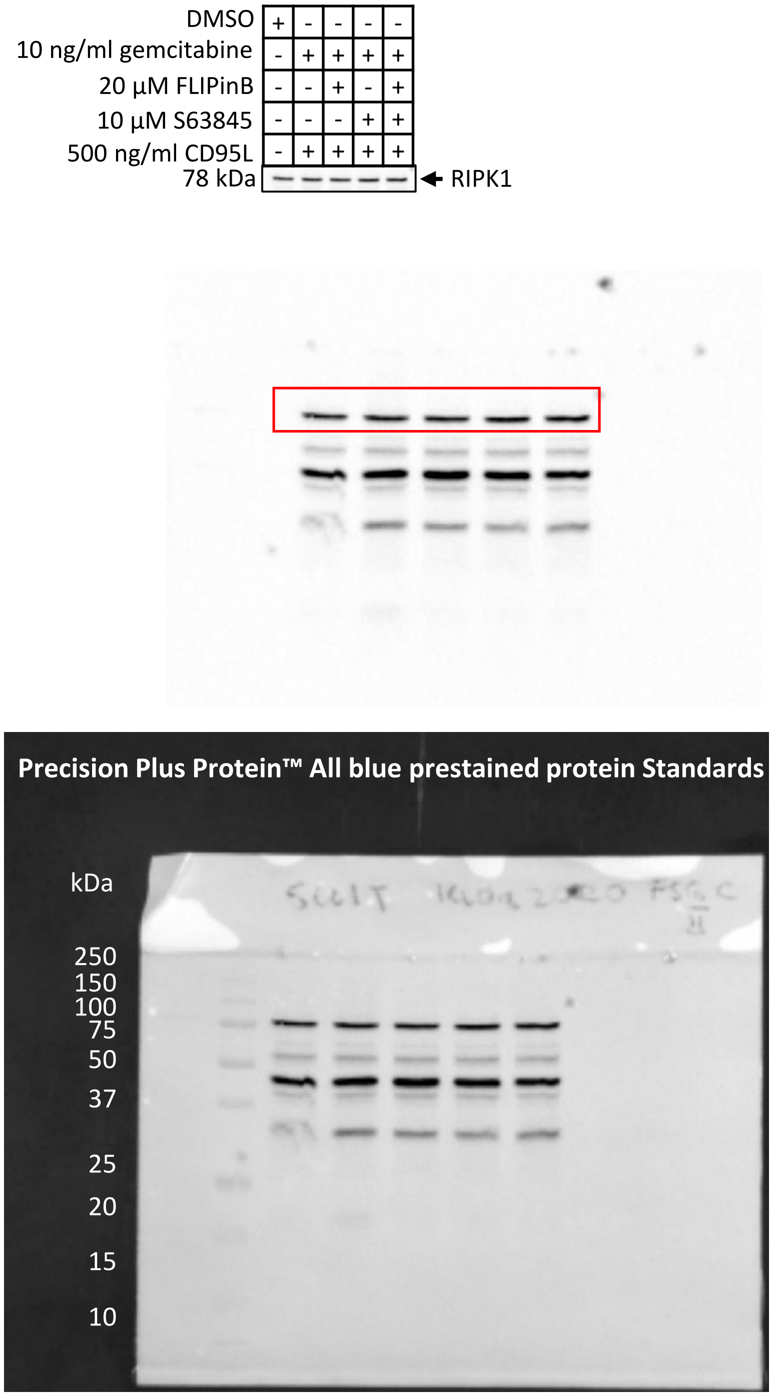
DMSO + - - - -
10 ng/ml gemcitabine - + + + +
20 µM FLIPinB - - + - +
10 µM S63845 - - - + +
500 ng/ml CD95L - + + + +
78 kDa
RIPK1
Precision Plus Protein™ All blue prestained protein Standards
kDa
250
150
100
75
50
37
25
20
15
10

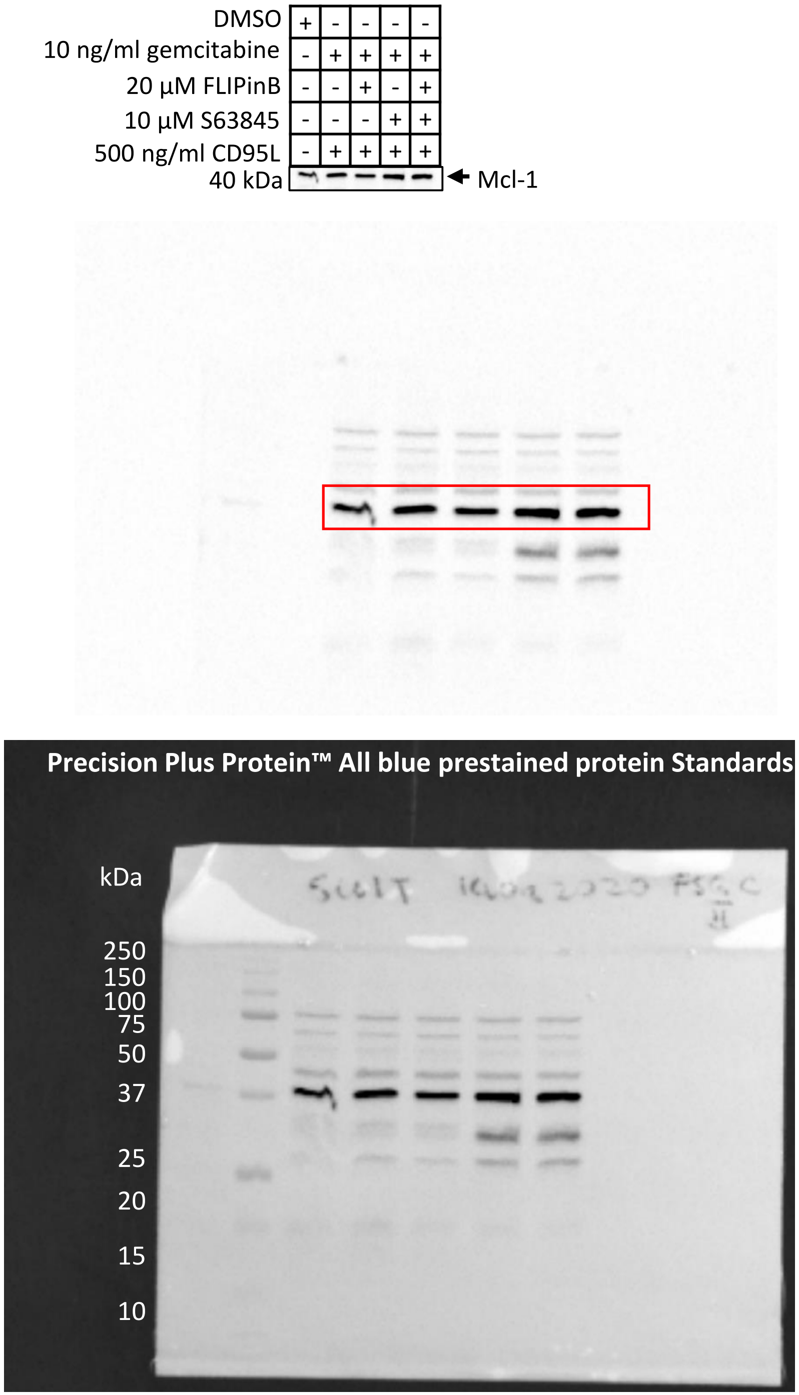
DMSO + - - - -
10 ng/ml gemcitabine - + + + +
20 µM FLIPinB - - + - +
10 µM S63845 - - - + +
500 ng/ml CD95L - + + + +
40 kDa
Mcl-1
Precision Plus Protein™ All blue prestained protein Standards
kDa
250
150
100
75
50
37
25
20
15
10

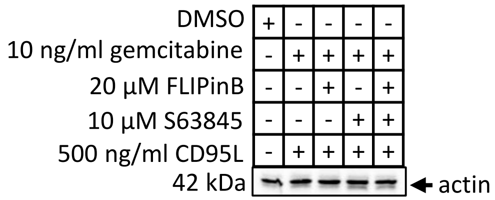
DMSO
10 ng/ml gemcitabine
20 µM FLIPinB
10 µM S63845
500 ng/ml CD95L
42 kDa
actin

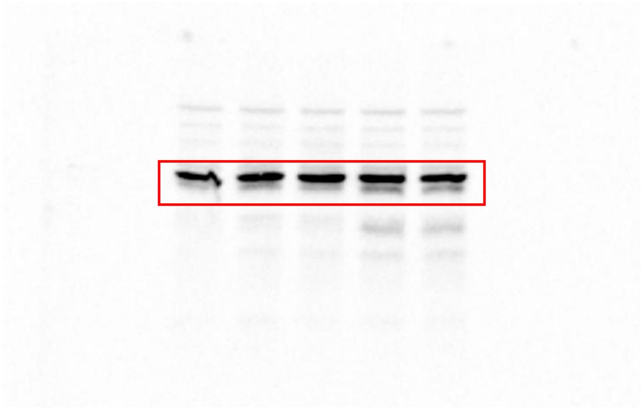

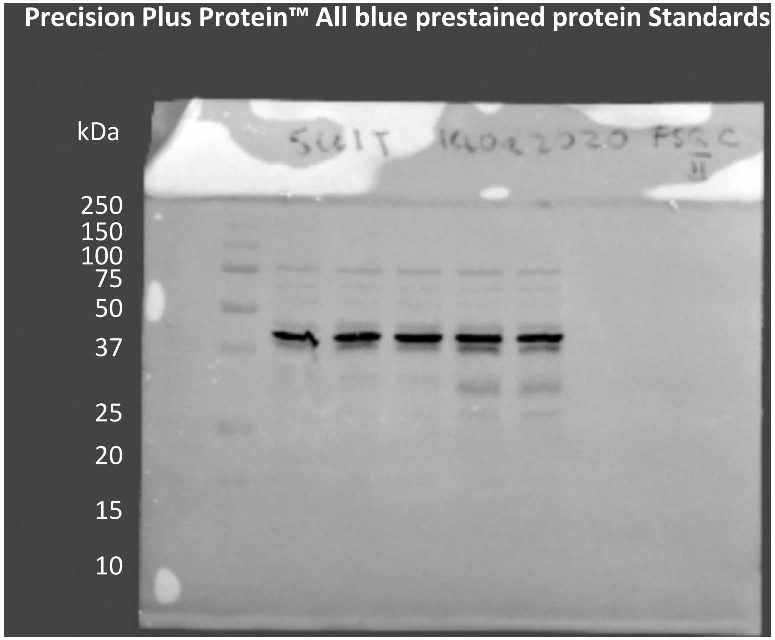
Precision Plus Protein™ All blue prestained protein Standards
kDa
250
150
100
75
50
37
25
20
15
10

c

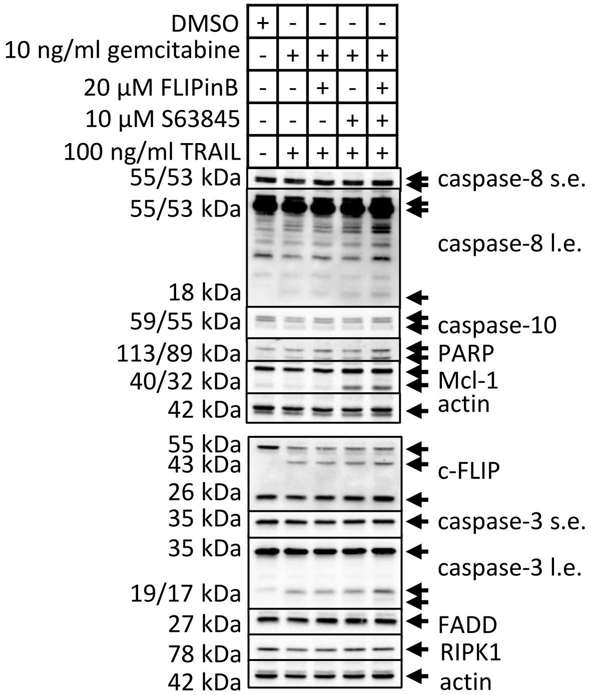
DMSO
10 ng/ml gemcitabine
20 µM FLIPinB
10 µM S63845
100 ng/ml TRAIL
55/53 kDa
caspase-8 s.e.
55/53 kDa
caspase-8 l.e.
18 kDa
59/55 kDa
caspase-10
113/89 kDa
PARP
40/32 kDa
Mcl-1
42 kDa
actin
55 kDa
43 kDa
c-FLIP
26 kDa
35 kDa
caspase-3 s.e.
35 kDa
caspase-3 l.e.
19/17 kDa
27 kDa
FADD
78 kDa
RIPK1
42 kDa
actin

DMSO	+	-	-	-	-
10 ng/ml gemcitabine	-	+	+	+	+
20 µM FLIPinB	-	-	+	-	+
10 µM S63845	-	-	-	+	+
100 ng/ml TRAIL	-	+	+	+	+

55/53 kDa

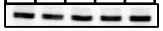

caspase-8 s.e.

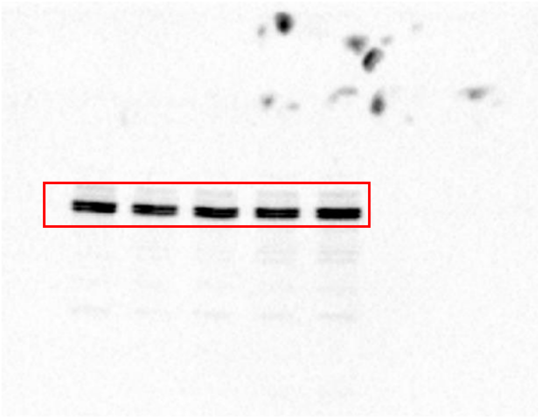

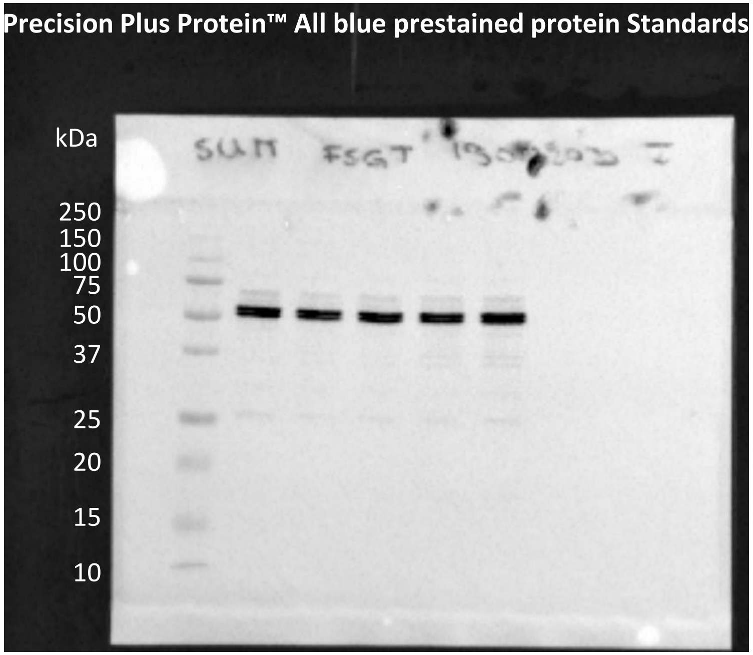

DMSO	+	-	-	-	-
10 ng/ml gemcitabine	-	+	+	+	+
20 µM FLIPinB	-	-	+	-	+
10 µM S63845	-	-	-	+	+
100 ng/ml TRAIL	-	+	+	+	+

55/53 kDa

18 kDa

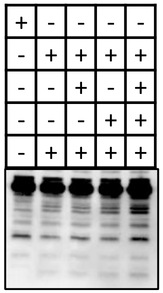

caspase-8 l.e.

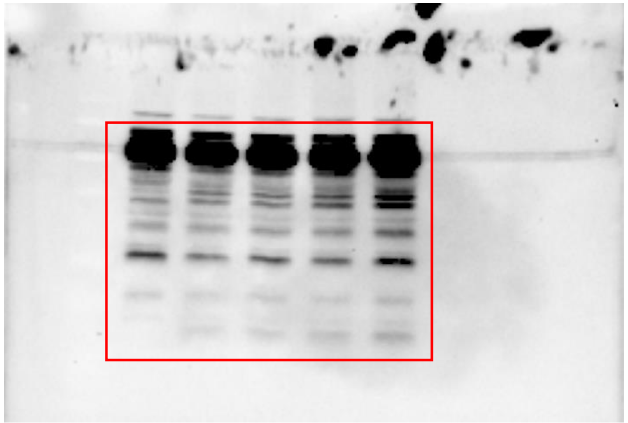

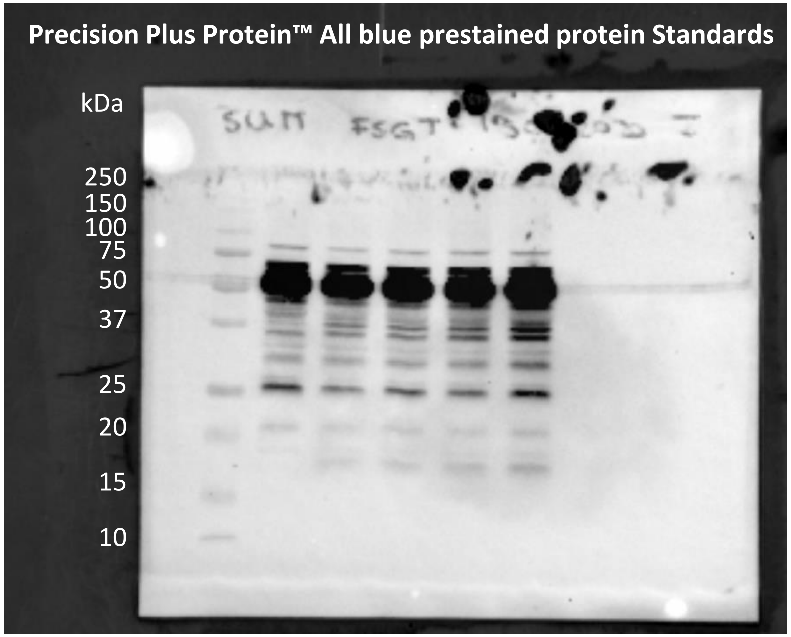

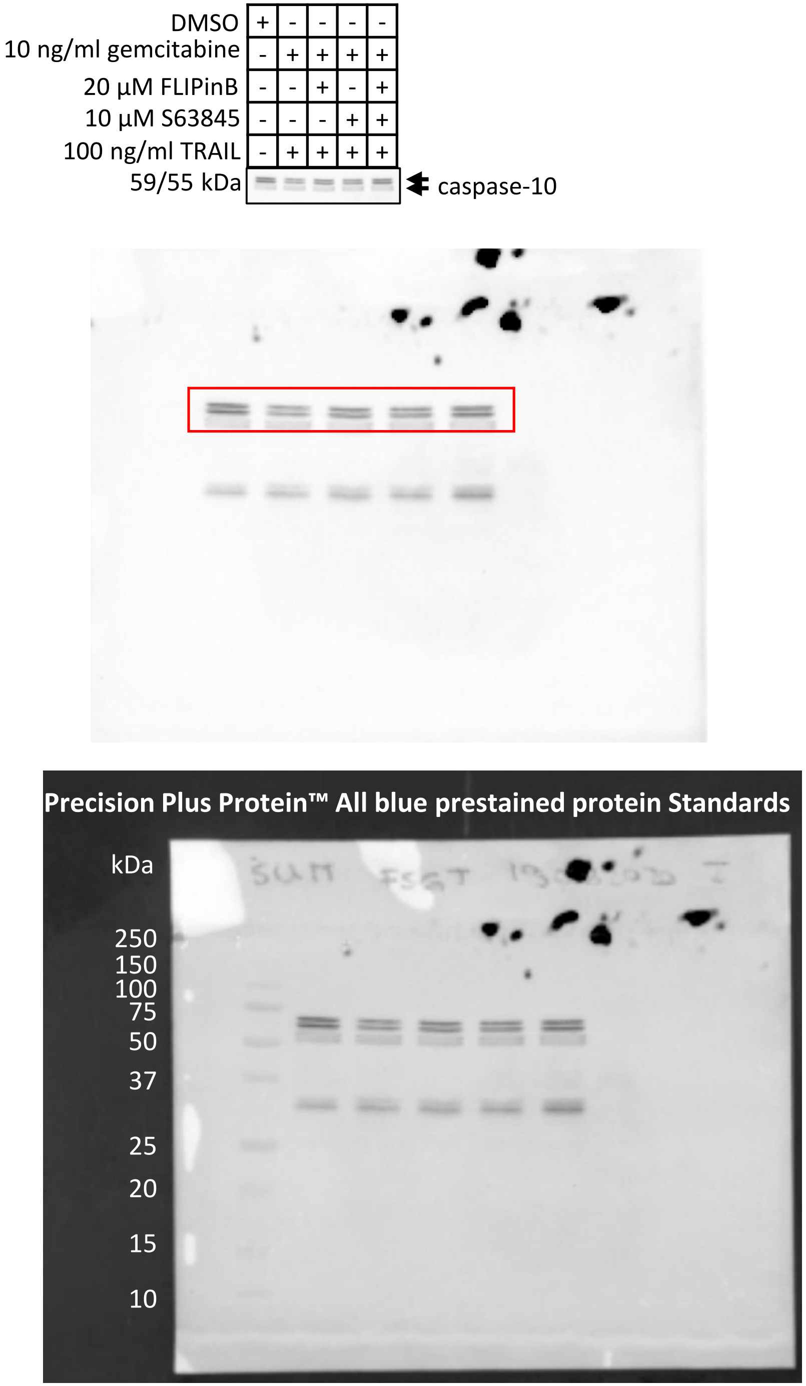
DMSO + - - - -
10 ng/ml gemcitabine - + + + +
20 µM FLIPinB - - + - +
10 µM S63845 - - - + +
100 ng/ml TRAIL - + + + +
59/55 kDa
caspase-10
Precision Plus Protein™ All blue prestained protein Standards
kDa
250
150
100
75
50
37
25
20
15
10

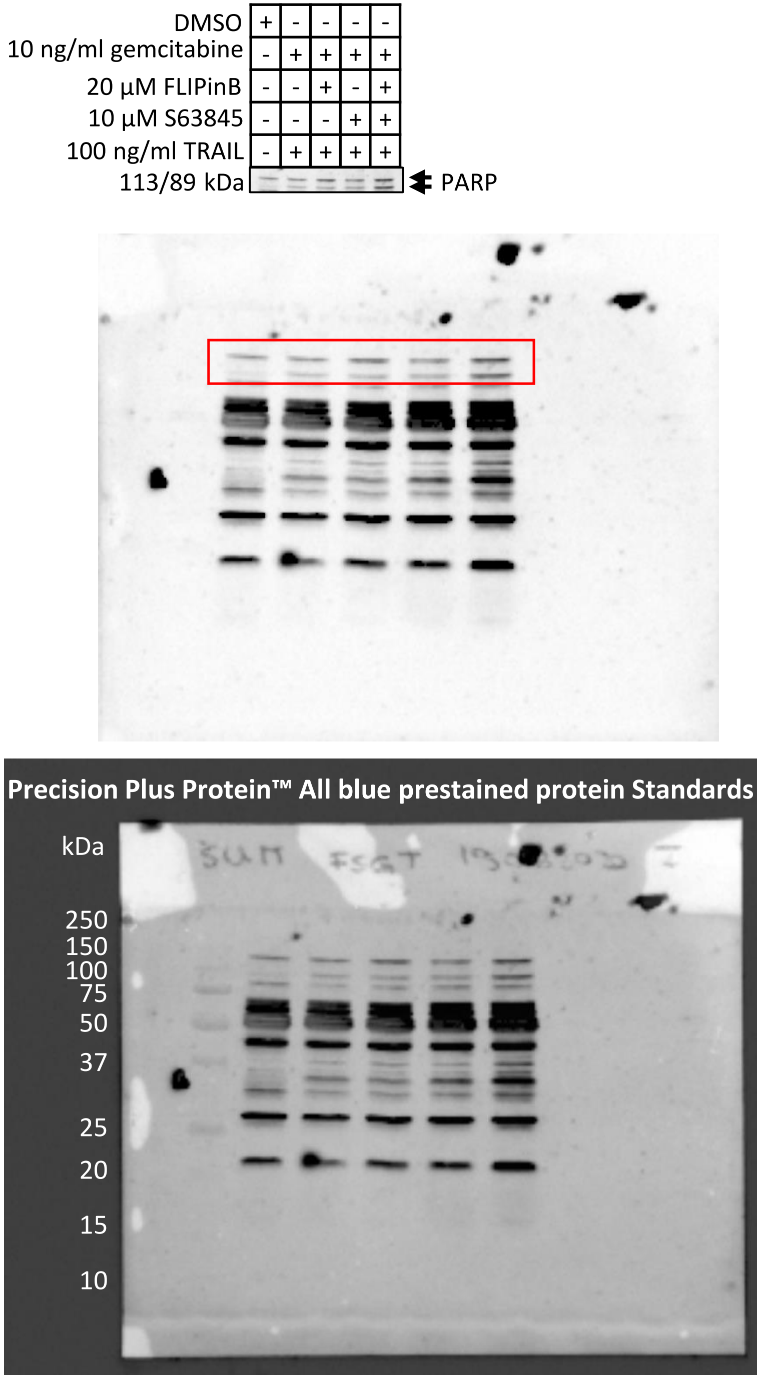
DMSO + - - - -
10 ng/ml gemcitabine - + + + +
20 µM FLIPinB - - + - +
10 µM S63845 - - - + +
100 ng/ml TRAIL - + + + +
113/89 kDa
PARP
Precision Plus Protein™ All blue prestained protein Standards
kDa
250
150
100
75
50
37
25
20
15
10

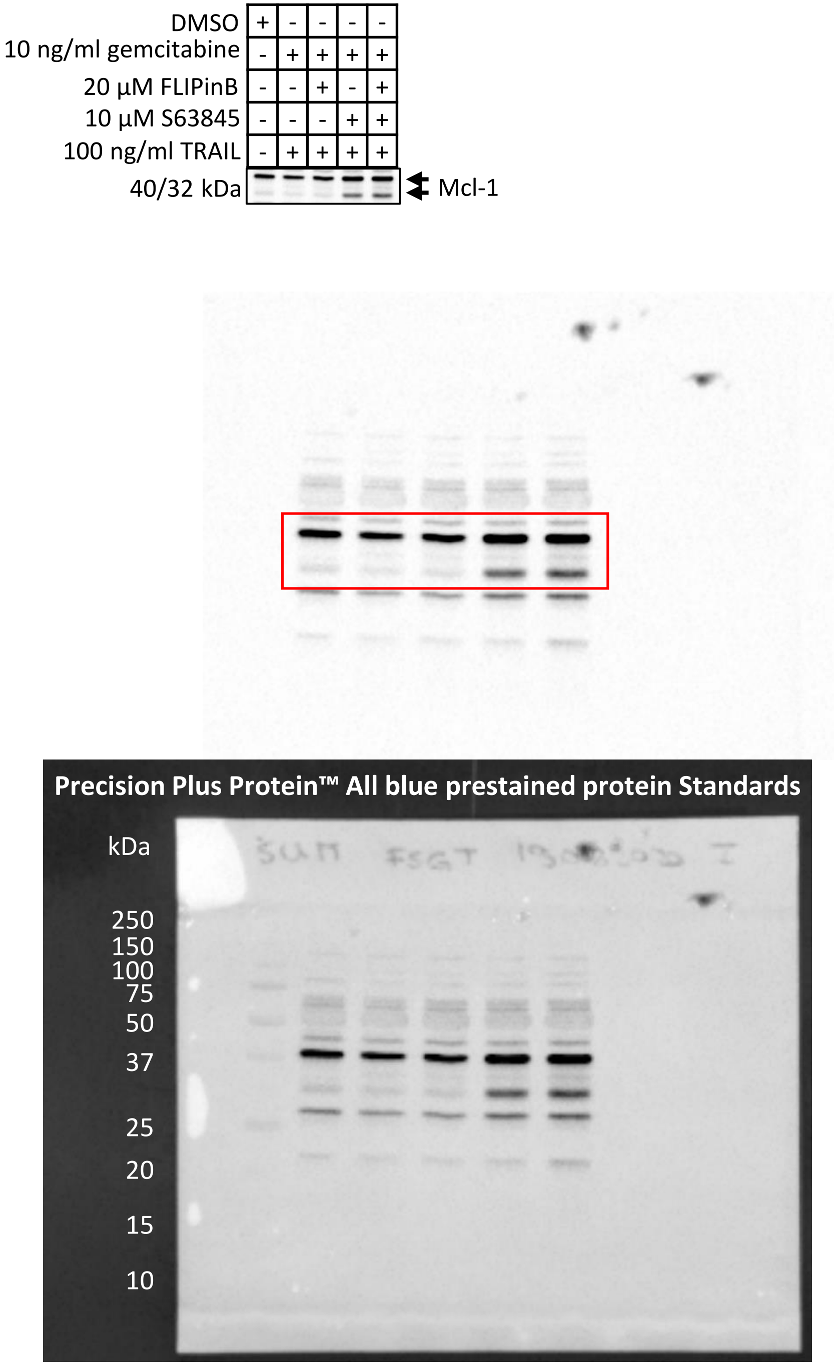
DMSO + - - - -
10 ng/ml gemcitabine - + + + +
20 µM FLIPinB - - + - +
10 µM S63845 - - - + +
100 ng/ml TRAIL - + + + +
40/32 kDa
Mcl-1
Precision Plus Protein™ All blue prestained protein Standards
kDa
250
150
100
75
50
37
25
20
15
10

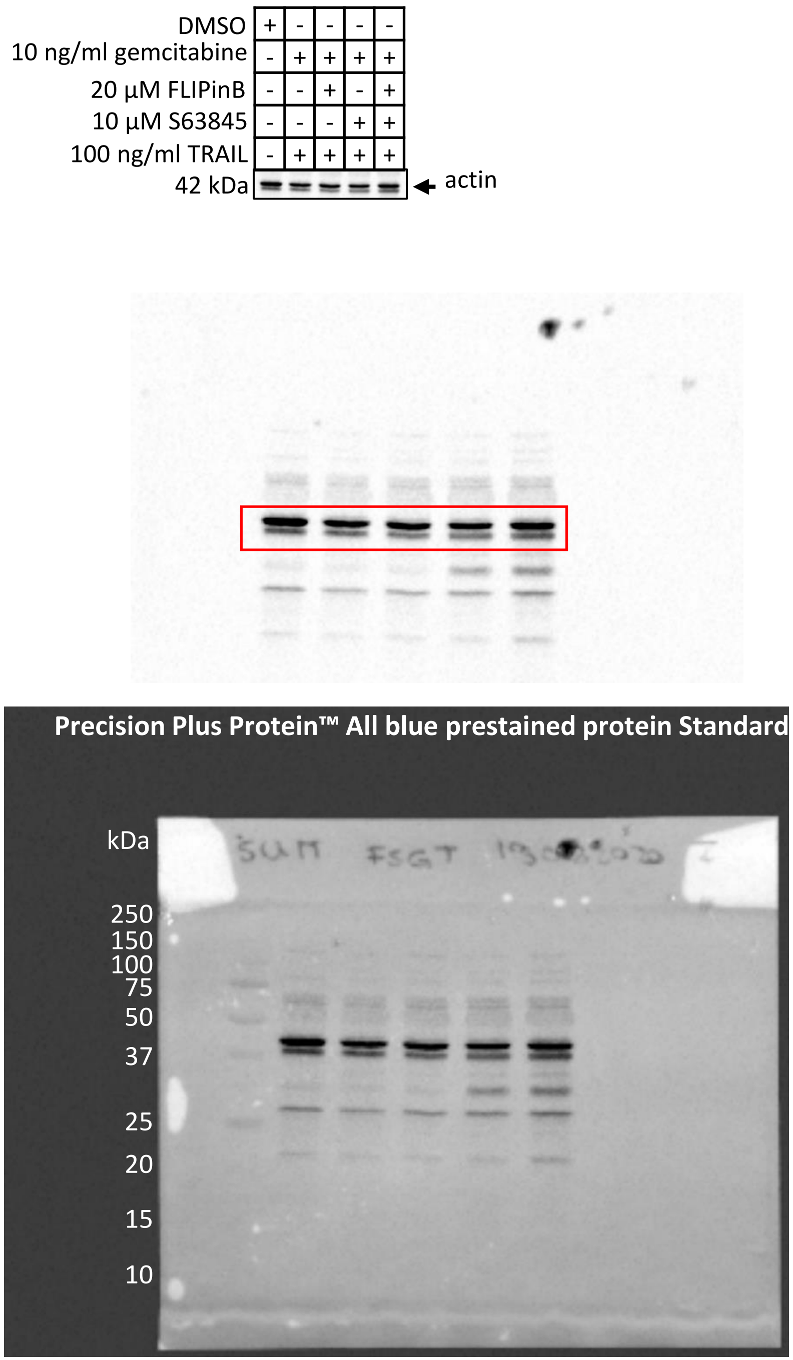
DMSO + - - - -
10 ng/ml gemcitabine - + + + +
20 µM FLIPinB - - + - +
10 µM S63845 - - - + +
100 ng/ml TRAIL - + + + +
42 kDa
actin
Precision Plus Protein™ All blue prestained protein Standard
kDa
250
150
100
75
50
37
25
20
15
10

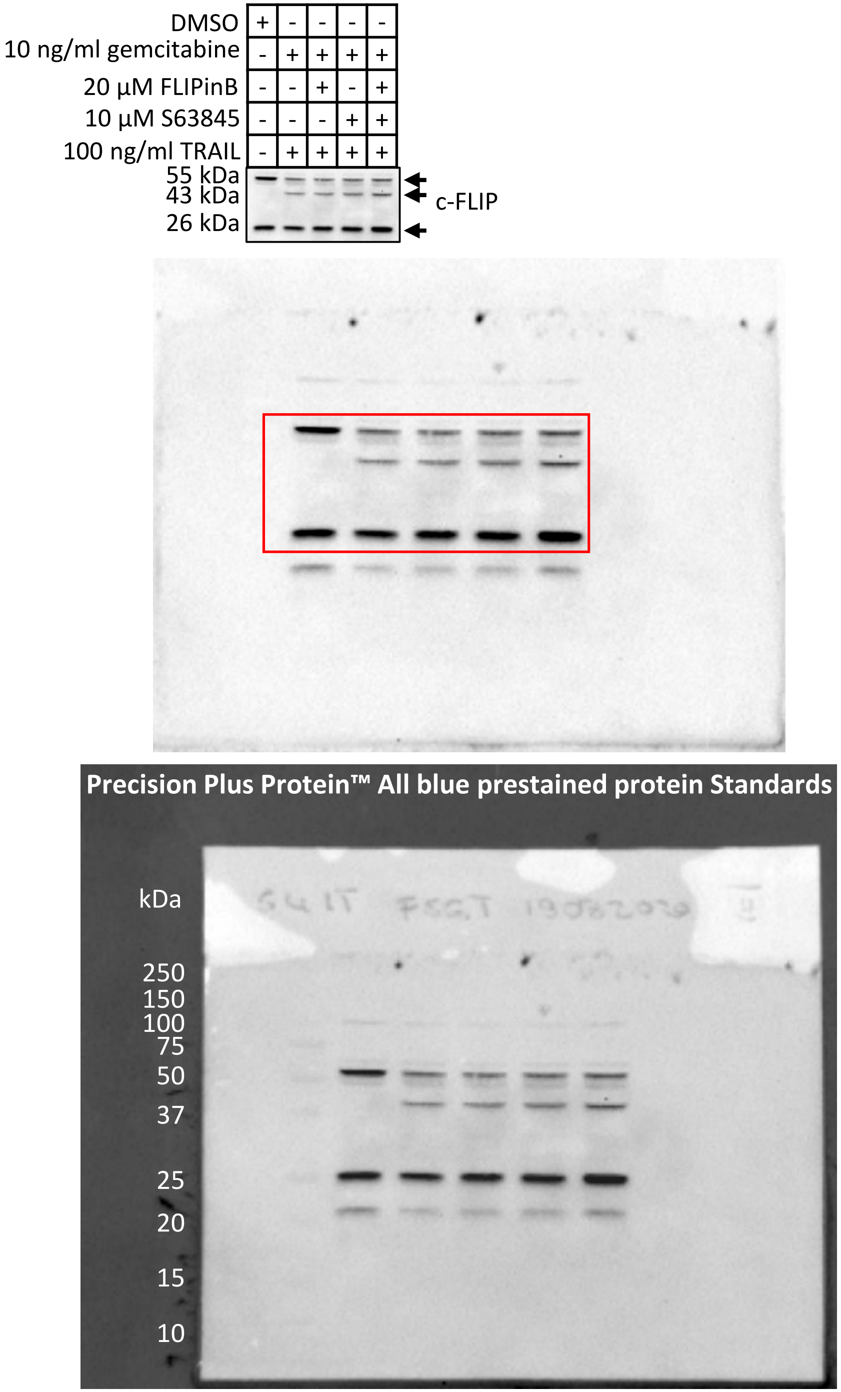
DMSO + - - - -
10 ng/ml gemcitabine - + + + +
20 µM FLIPinB - - + - +
10 µM S63845 - - - + +
100 ng/ml TRAIL - + + + +
55 kDa
43 kDa
26 kDa
c-FLIP
Precision Plus Protein™ All blue prestained protein Standards
kDa
250
150
100
75
50
37
25
20
15
10

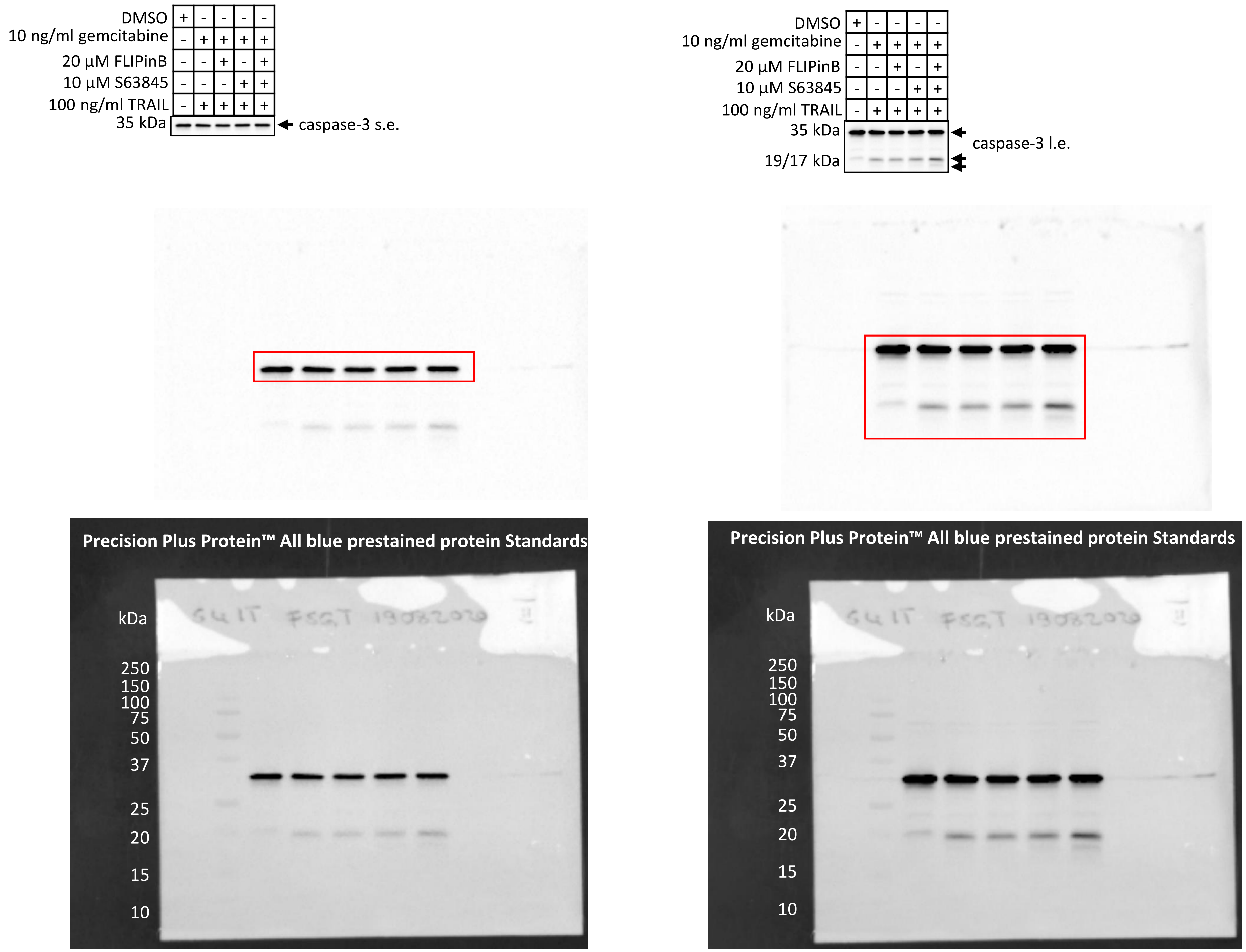
DMSO
10 ng/ml gemcitabine
20 µM FLIPinB
10 µM S63845
100 ng/ml TRAIL
35 kDa
caspase-3 s.e.
DMSO
10 ng/ml gemcitabine
20 µM FLIPinB
10 µM S63845
100 ng/ml TRAIL
35 kDa
19/17 kDa
caspase-3 l.e.
Precision Plus Protein™ All blue prestained protein Standards
kDa
250
150
100
75
50
37
25
20
15
10
Precision Plus Protein™ All blue prestained protein Standards
kDa
250
150
100
75
50
37
25
20
15
10

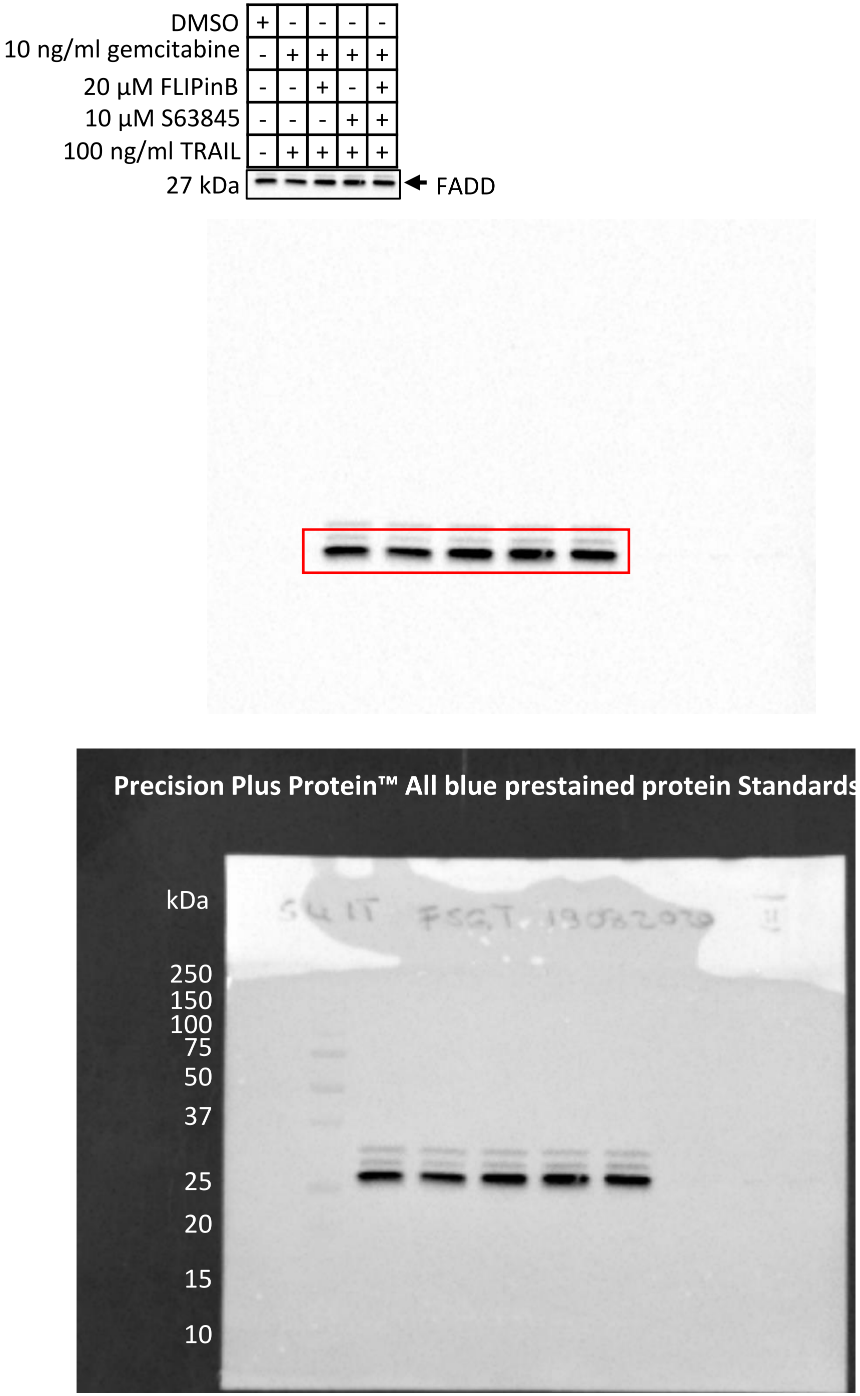
DMSO + - - - -
10 ng/ml gemcitabine - + + + +
20 µM FLIPinB - - + - +
10 µM S63845 - - - + +
100 ng/ml TRAIL - + + + +
27 kDa
FADD
Precision Plus Protein™ All blue prestained protein Standards
kDa
250
150
100
75
50
37
25
20
15
10

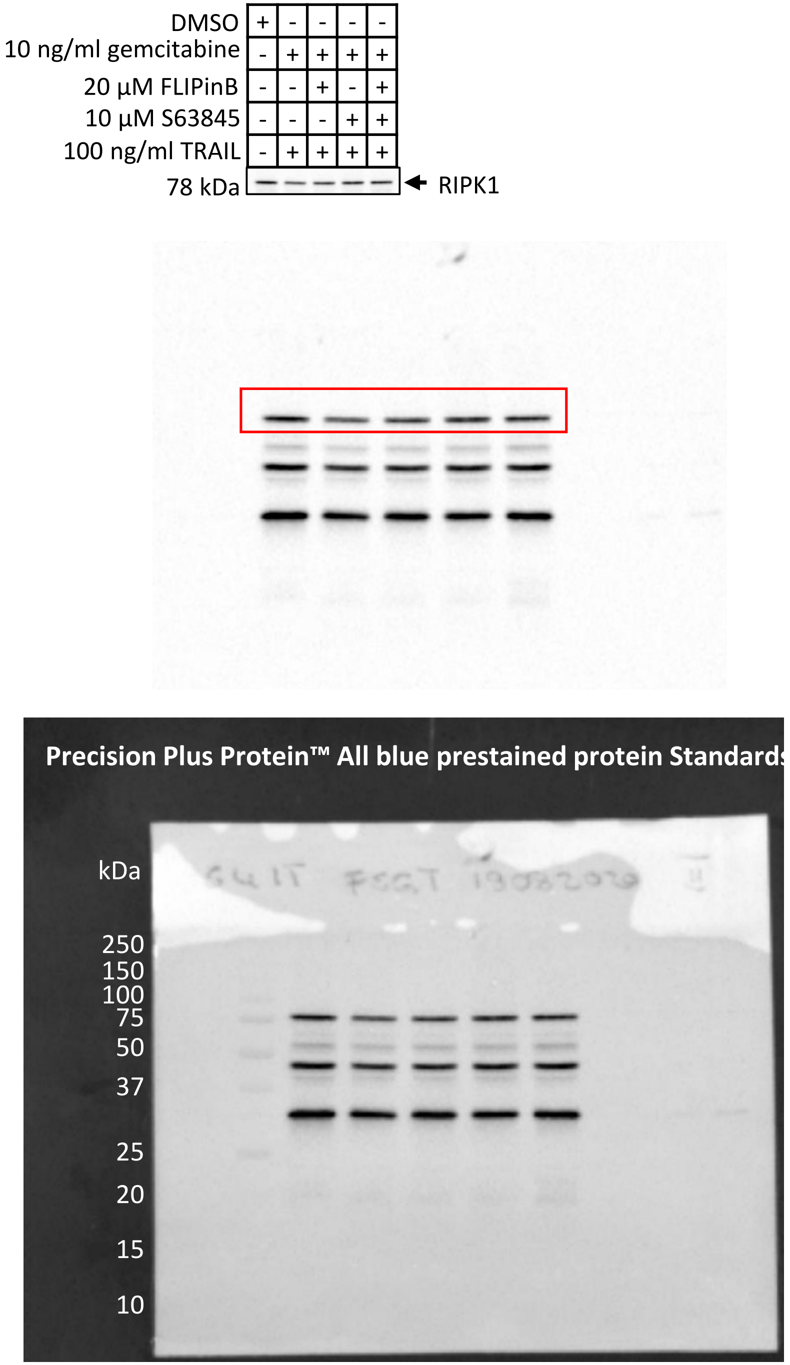
DMSO + - - - -
10 ng/ml gemcitabine - + + + +
20 µM FLIPinB - - + - +
10 µM S63845 - - - + +
100 ng/ml TRAIL - + + + +
78 kDa
RIPK1
Precision Plus Protein™ All blue prestained protein Standards
kDa
250
150
100
75
50
37
25
20
15
10

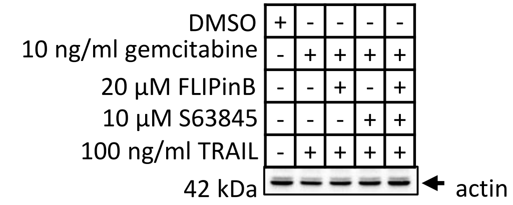

DMSO + - - - -
10 ng/ml gemcitabine - + + + +
20 µM FLIPinB - - + - +
10 µM S63845 - - - + +
100 ng/ml TRAIL - + + + +
42 kDa
actin

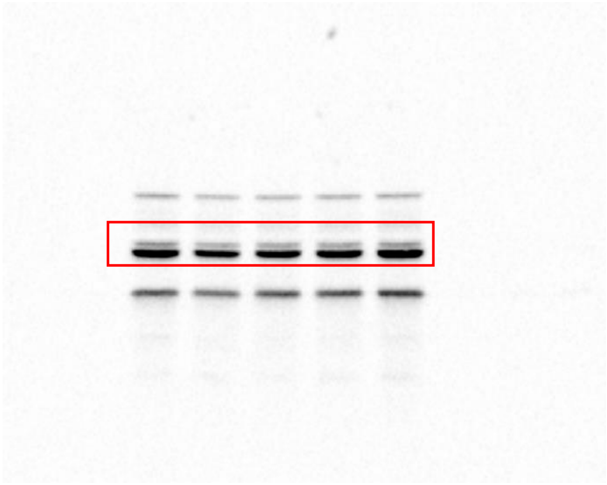

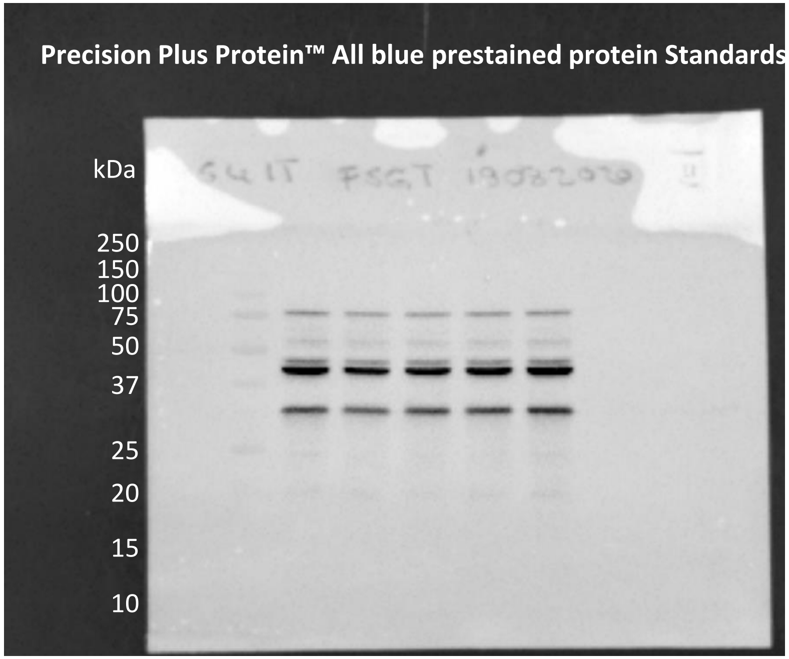

Precision Plus Protein™ All blue prestained protein Standards
kDa
250
150
100
75
50
37
25
20
15
10

c

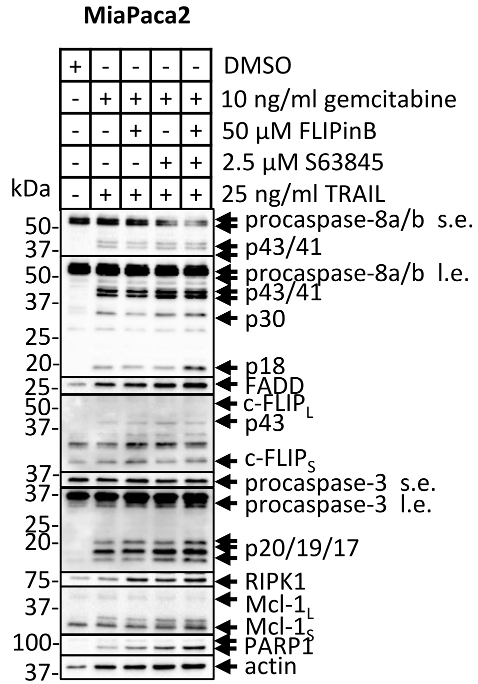

MiaPaca2
DMSO
10 ng/ml gemcitabine
50 µM FLIPinB
2.5 µM S63845
25 ng/ml TRAIL
kDa
procaspase-8a/b s.e.
p43/41
procaspase-8a/b l.e.
p43/41
p30
p18
FADD
c-FLIPL
p43
c-FLIPS
procaspase-3 s.e.
procaspase-3 l.e.
p20/19/17
RIPK1
Mcl-1L
Mcl-1S
PARP1
actin

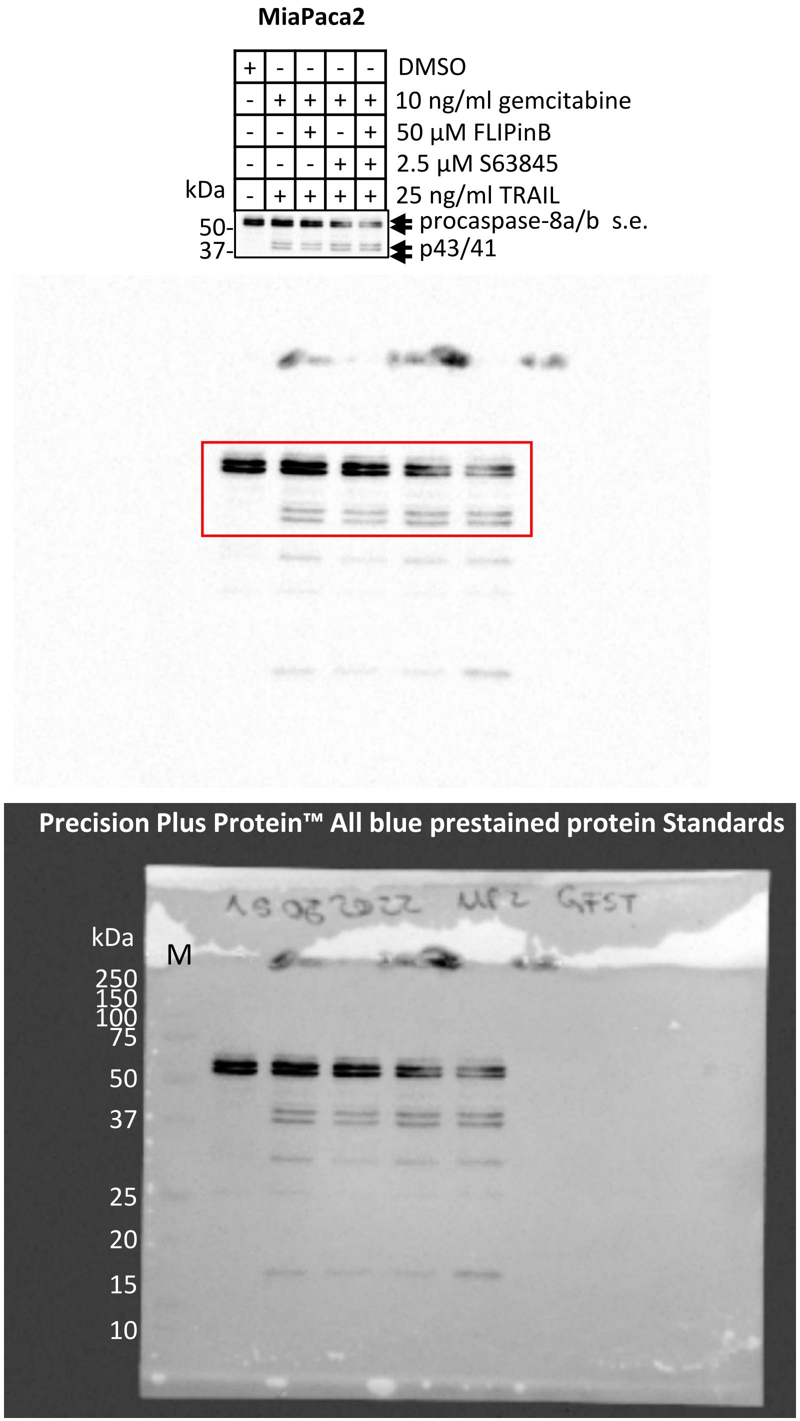
MiaPaca2
DMSO
10 ng/ml gemcitabine
50 µM FLIPinB
2.5 µM S63845
25 ng/ml TRAIL
kDa
50-
37-
procaspase-8a/b s.e.
p43/41
Precision Plus Protein™ All blue prestained protein Standards
kDa
M
250
150
100
75
50
37
25
20
15
10

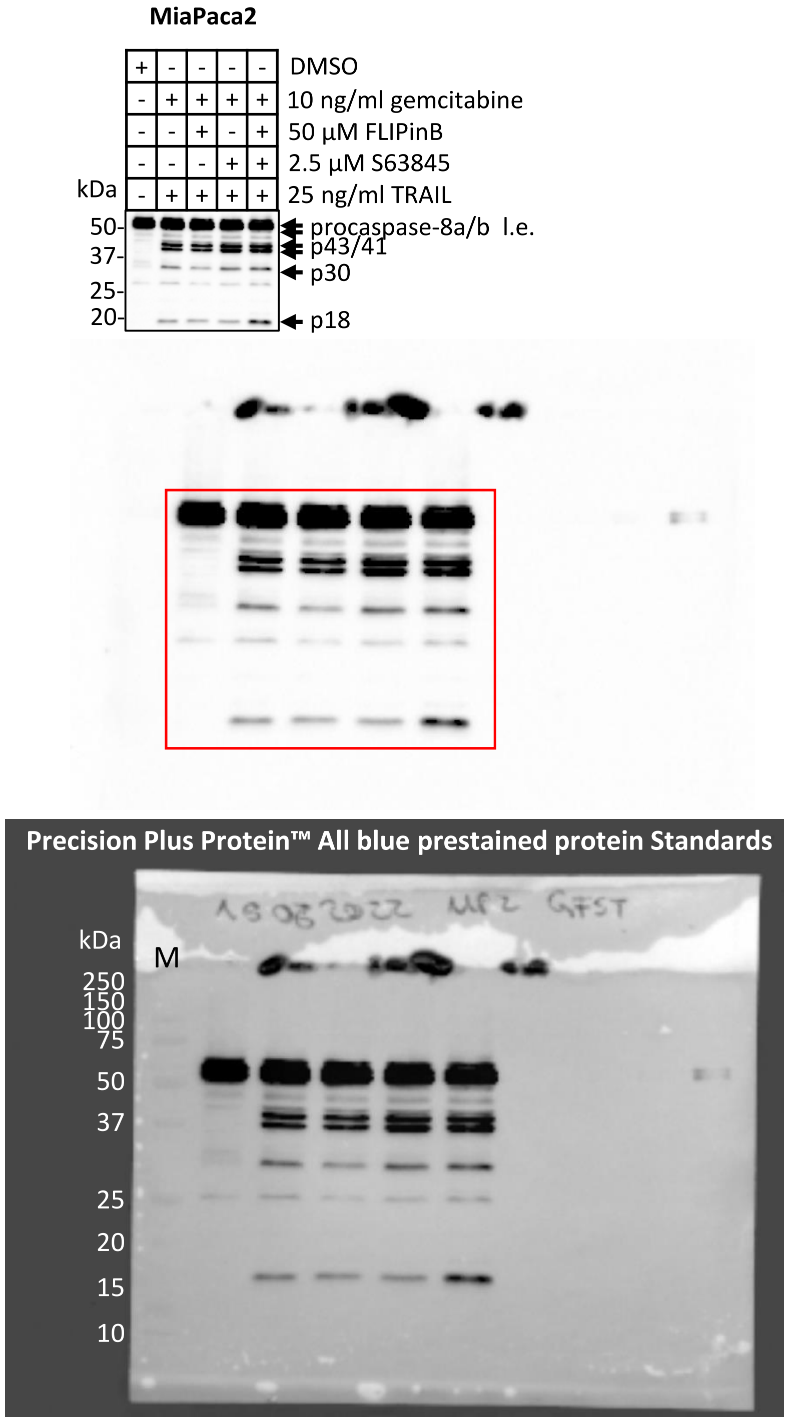
MiaPaca2
DMSO
10 ng/ml gemcitabine
50 µM FLIPinB
2.5 µM S63845
25 ng/ml TRAIL
kDa
50-
37-
25-
20-
procaspase-8a/b l.e.
p43/41
p30
p18
Precision Plus Protein™ All blue prestained protein Standards
kDa
M
250
150
100
75
50
37
25
20
15
10

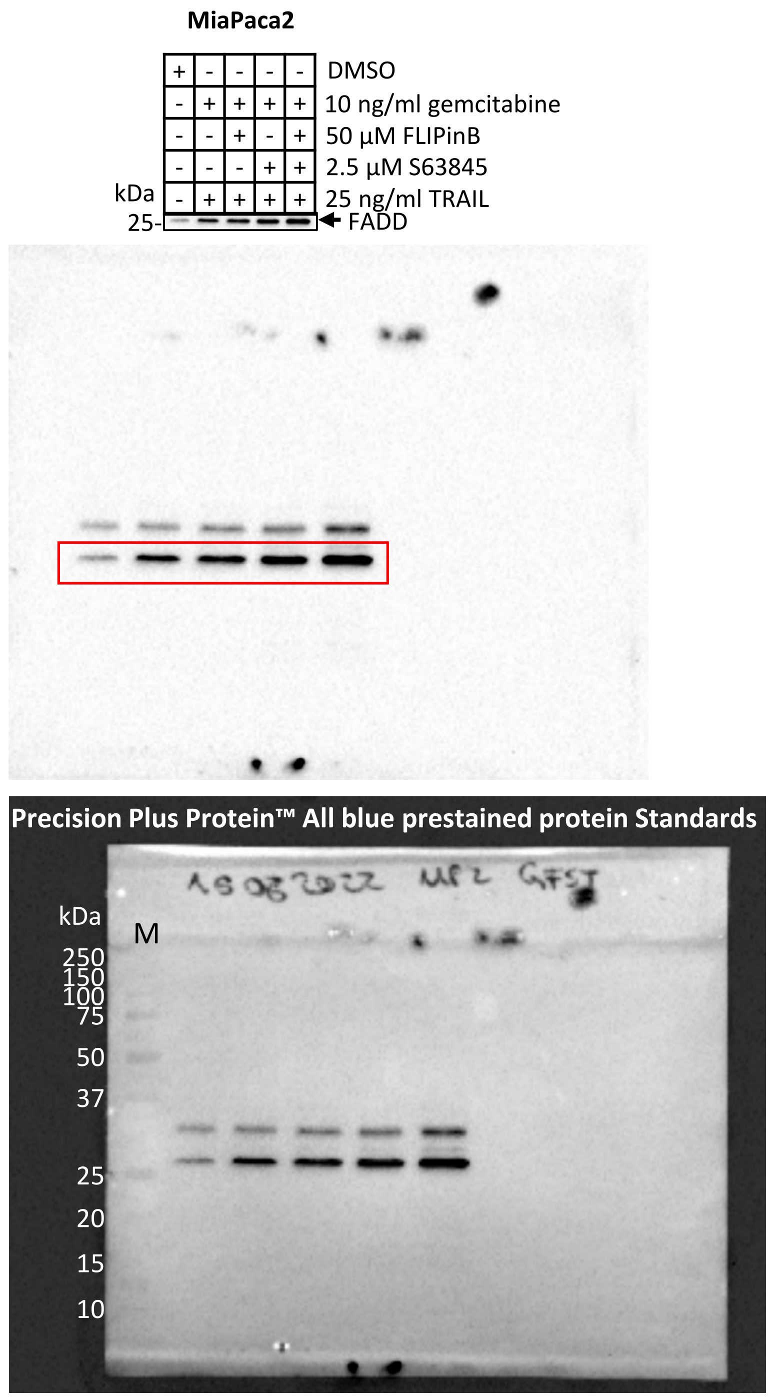
MiaPaca2
+ - - - - DMSO
- + + + + 10 ng/ml gemcitabine
- - + - + 50 µM FLIPinB
- - - + + 2.5 µM S63845
- + + + + 25 ng/ml TRAIL
kDa
25-
FADD
Precision Plus Protein™ All blue prestained protein Standards
kDa
M
250
150
100
75
50
37
25
20
15
10

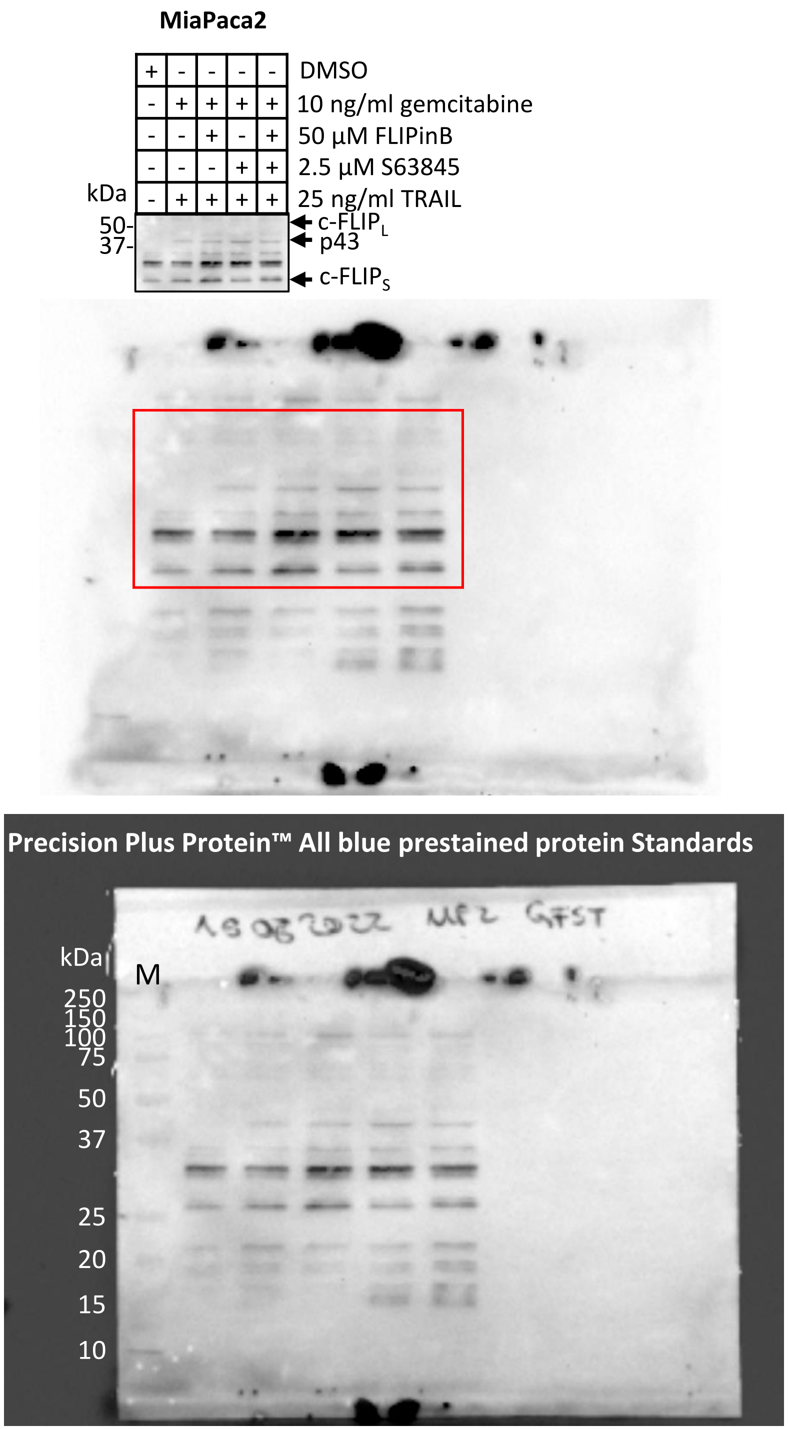
MiaPaca2
+ - - - - DMSO
- + + + + 10 ng/ml gemcitabine
- - + - + 50 µM FLIPinB
- - - + + 2.5 µM S63845
- + + + + 25 ng/ml TRAIL
kDa
50-
37-
c-FLIP$_L$
p43
c-FLIP$_S$
Precision Plus Protein™ All blue prestained protein Standards
kDa
M
250
150
100
75
50
37
25
20
15
10

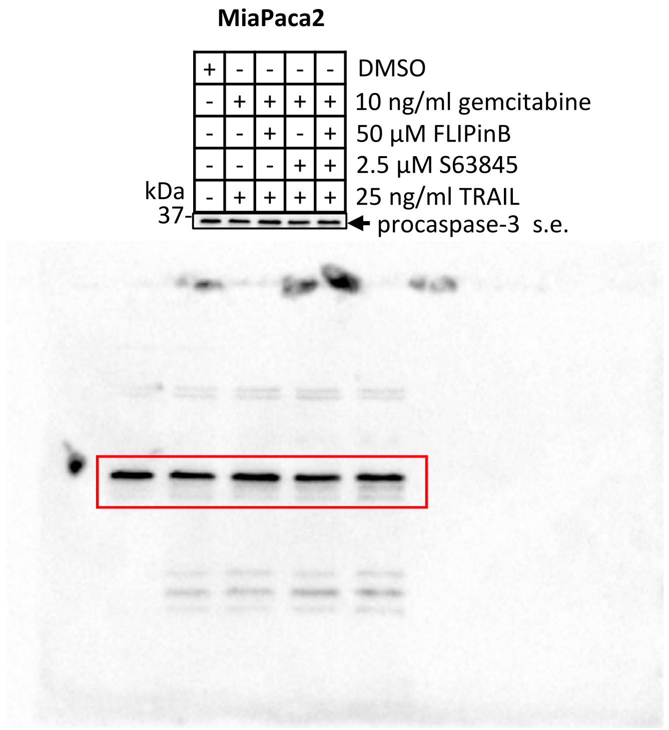
MiaPaca2
+ - - - - DMSO
- + + + + 10 ng/ml gemcitabine
- - + - + 50 µM FLIPinB
- - - + + 2.5 µM S63845
- + + + + 25 ng/ml TRAIL
kDa
37
procaspase-3 s.e.

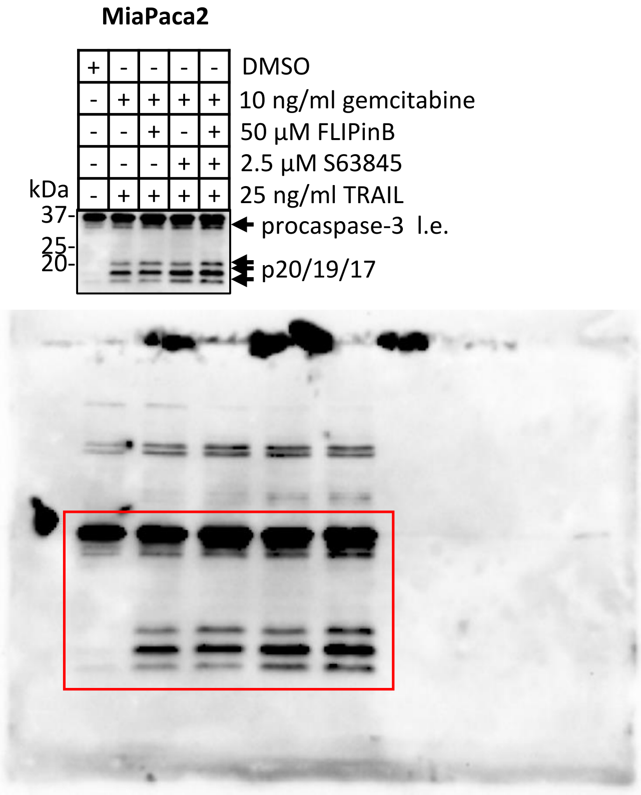
MiaPaca2
+ - - - - DMSO
- + + + + 10 ng/ml gemcitabine
- - + - + 50 µM FLIPinB
- - - + + 2.5 µM S63845
- + + + + 25 ng/ml TRAIL
kDa
37
25
20
procaspase-3 l.e.
p20/19/17

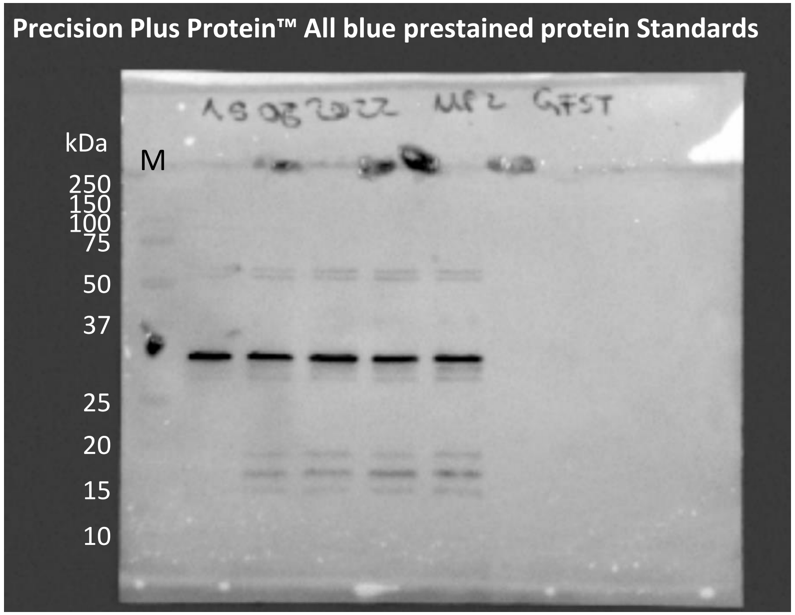
Precision Plus Protein™ All blue prestained protein Standards
kDa
M
250
150
100
75
50
37
25
20
15
10

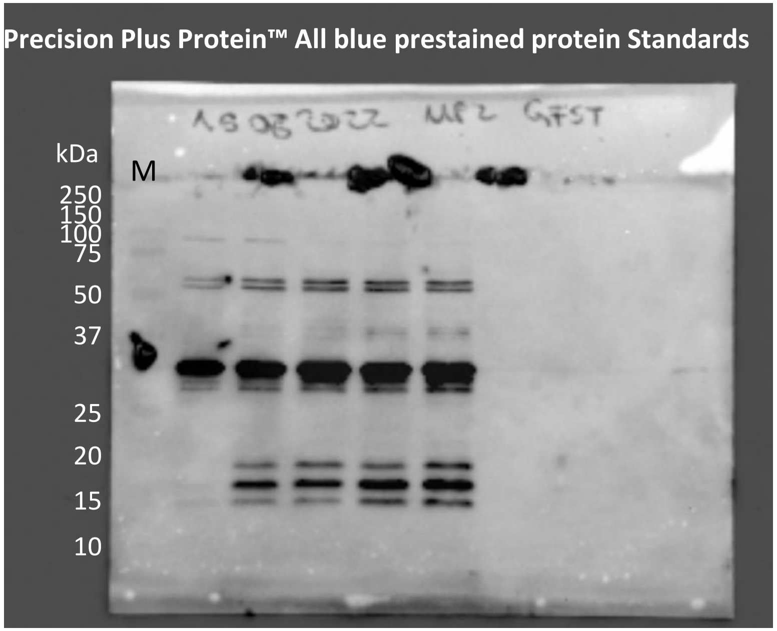
Precision Plus Protein™ All blue prestained protein Standards
kDa
M
250
150
100
75
50
37
25
20
15
10

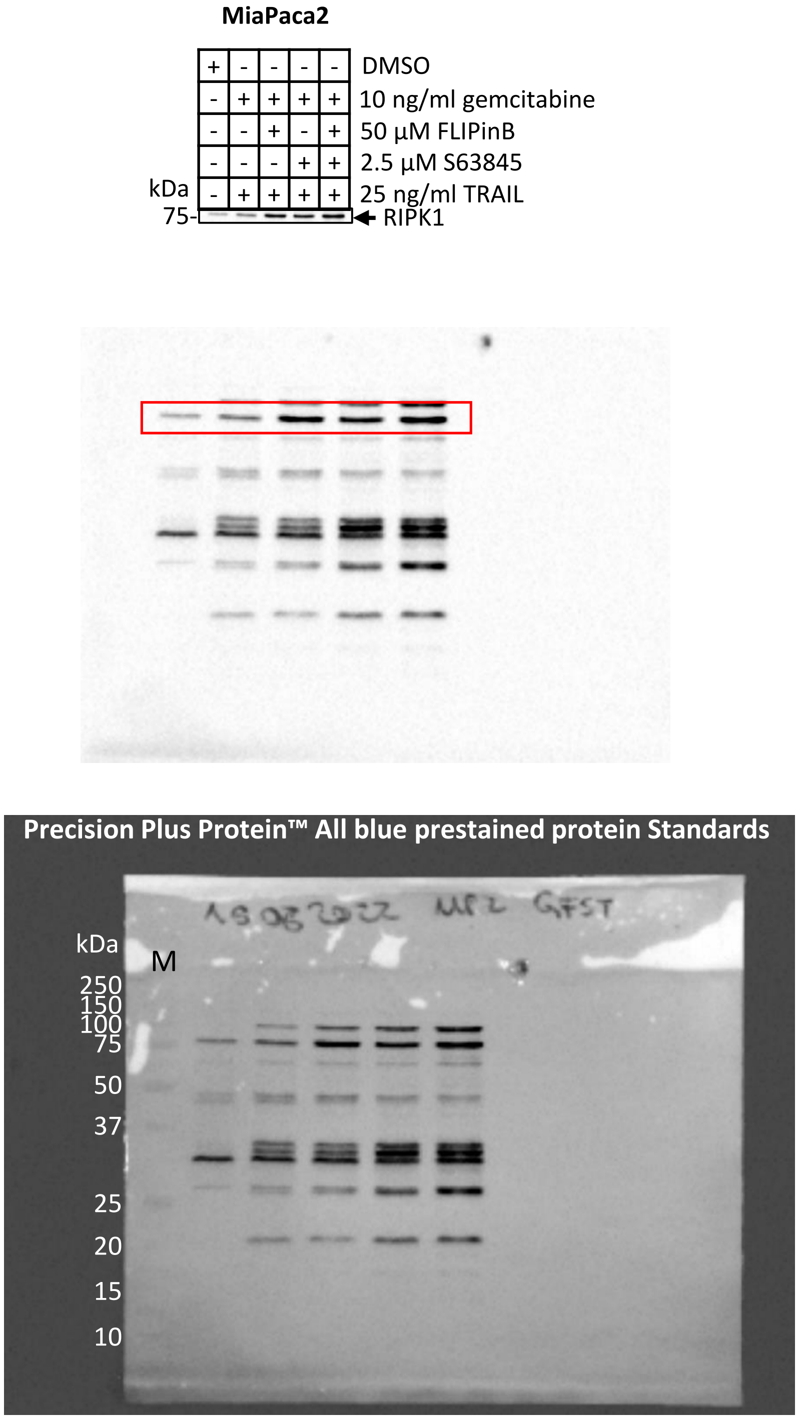
MiaPaca2
+ - - - - DMSO
- + + + + 10 ng/ml gemcitabine
- - + - + 50 µM FLIPinB
- - - + + 2.5 µM S63845
- + + + + 25 ng/ml TRAIL
kDa
75-
RIPK1
Precision Plus Protein™ All blue prestained protein Standards
kDa
M
250
150
100
75
50
37
25
20
15
10

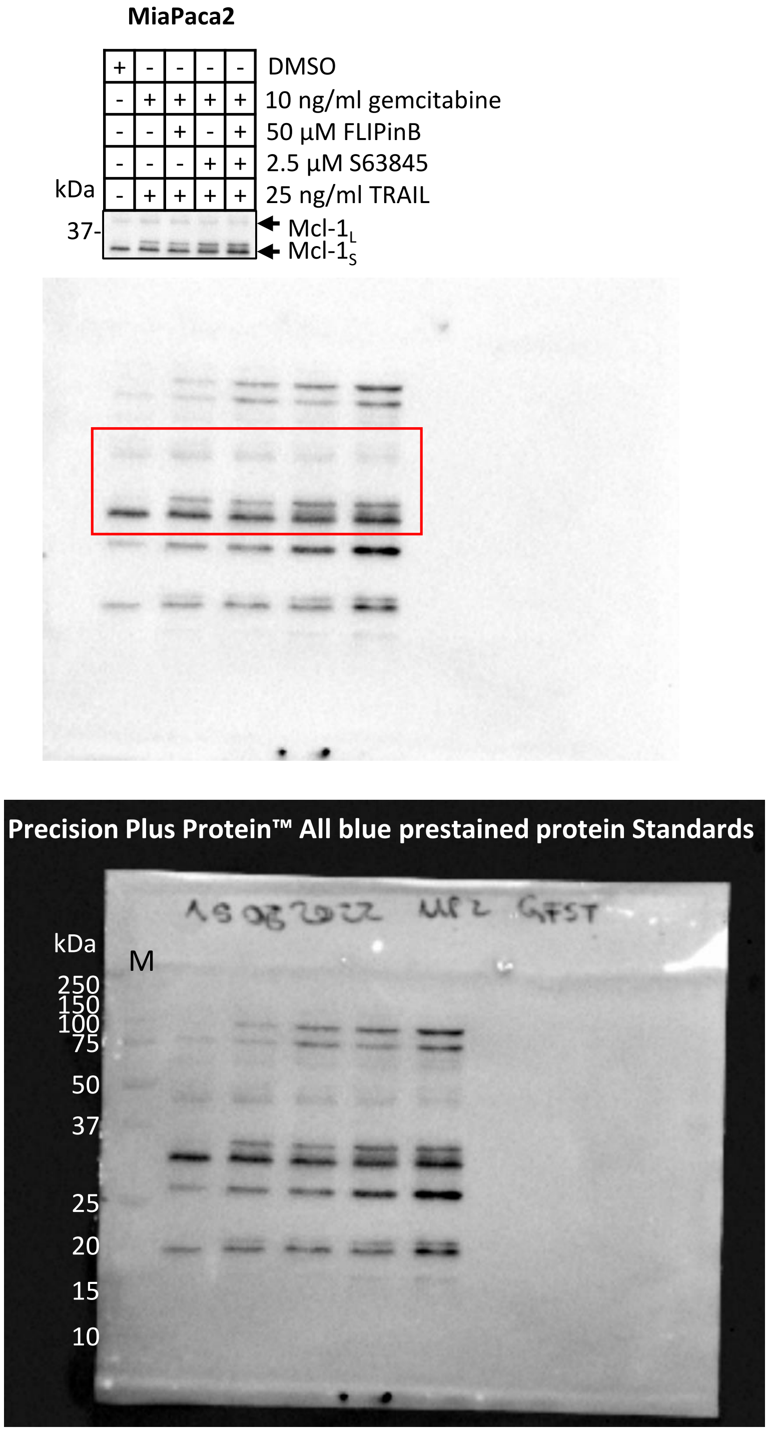
MiaPaca2
+ - - - - DMSO
- + + + + 10 ng/ml gemcitabine
- - + - + 50 µM FLIPinB
- - - + + 2.5 µM S63845
- + + + + 25 ng/ml TRAIL
kDa
37-
Mcl-1L
Mcl-1S
Precision Plus Protein™ All blue prestained protein Standards
kDa
M
250
150
100
75
50
37
25
20
15
10

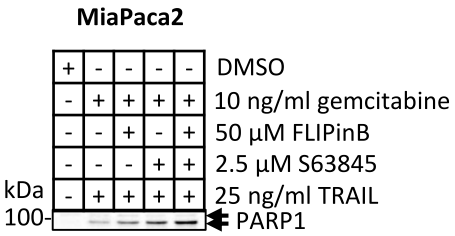
MiaPaca2
+ - - - - DMSO
- + + + + 10 ng/ml gemcitabine
- - + - + 50 µM FLIPinB
- - - + + 2.5 µM S63845
- + + + + 25 ng/ml TRAIL
kDa
100-
PARP1

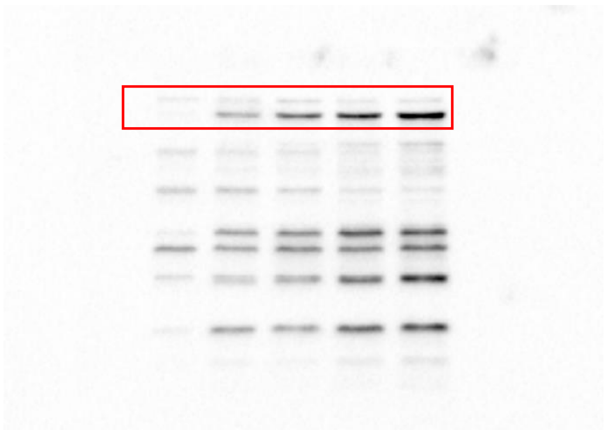

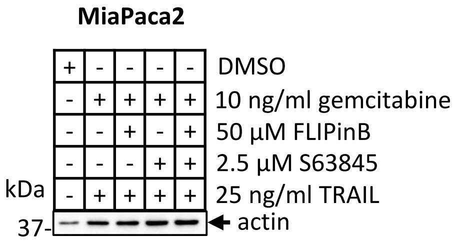
MiaPaca2
+ - - - - DMSO
- + + + + 10 ng/ml gemcitabine
- - + - + 50 µM FLIPinB
- - - + + 2.5 µM S63845
- + + + + 25 ng/ml TRAIL
kDa
37-
actin

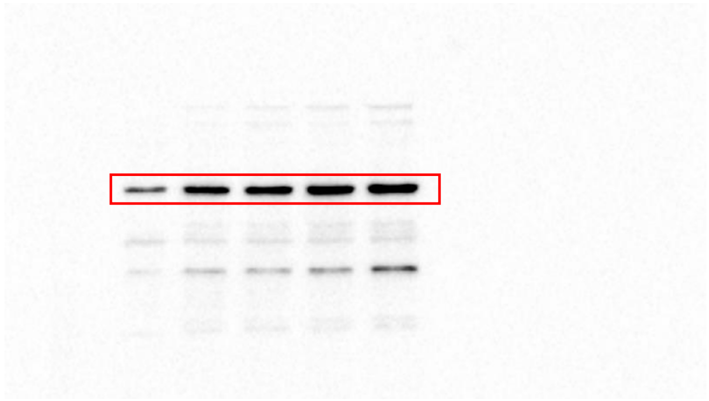

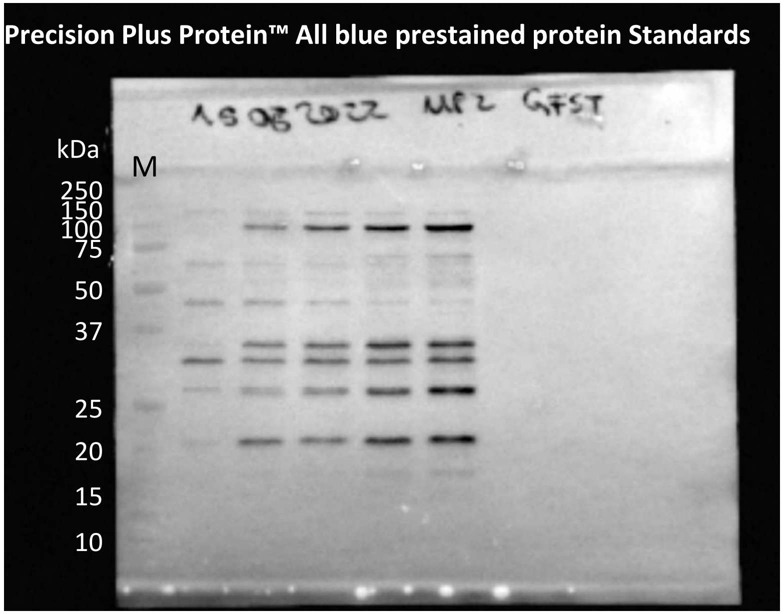
Precision Plus Protein™ All blue prestained protein Standards
kDa
M
250
150
100
75
50
37
25
20
15
10

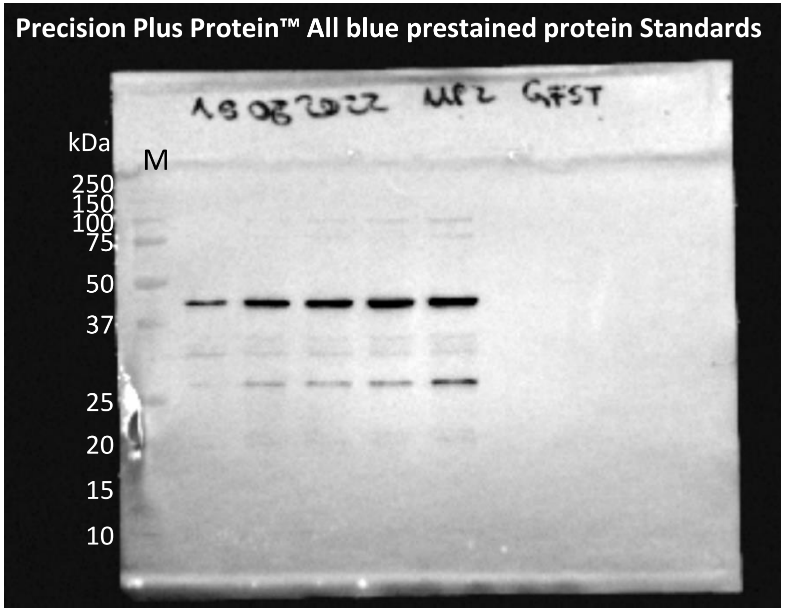
Precision Plus Protein™ All blue prestained protein Standards
kDa
M
250
150
100
75
50
37
25
20
15
10

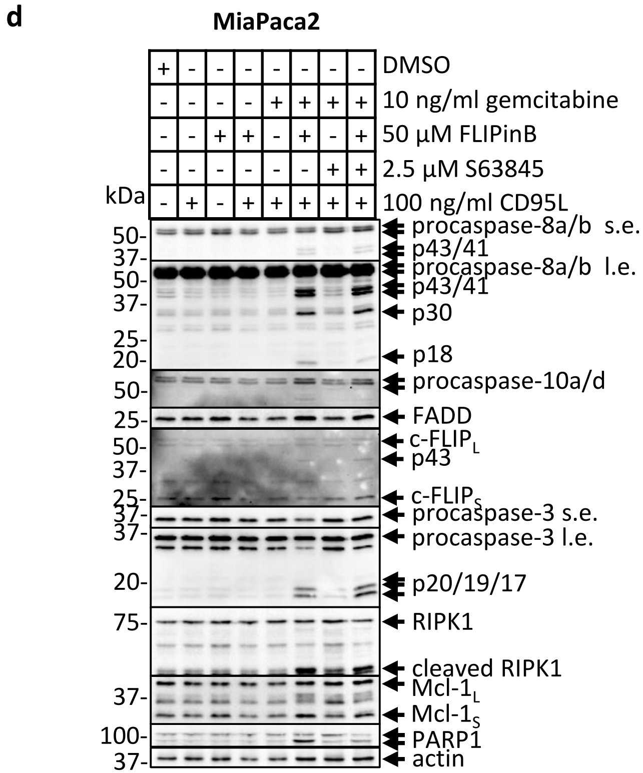

d
MiaPaca2
DMSO
10 ng/ml gemcitabine
50 µM FLIPinB
2.5 µM S63845
100 ng/ml CD95L
kDa
procaspase-8a/b s.e.
p43/41
procaspase-8a/b l.e.
p43/41
p30
p18
procaspase-10a/d
FADD
c-FLIPL
p43
c-FLIPS
procaspase-3 s.e.
procaspase-3 l.e.
p20/19/17
RIPK1
cleaved RIPK1
Mcl-1L
Mcl-1S
PARP1
actin

MiaPaca2

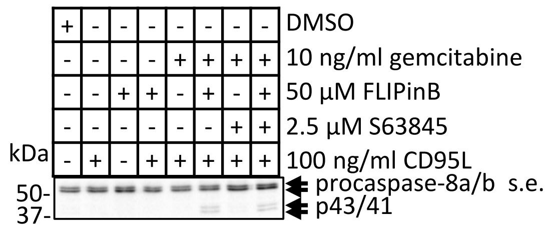

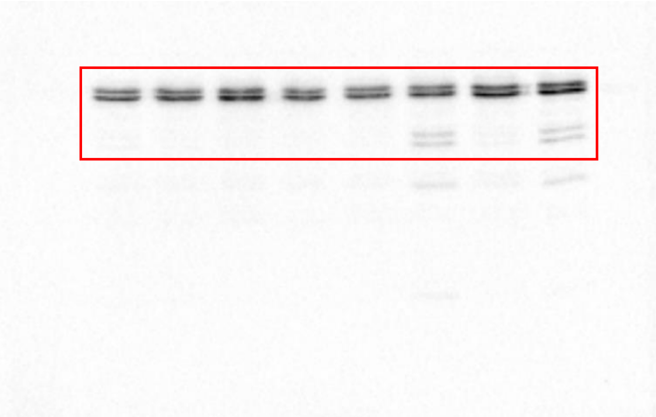

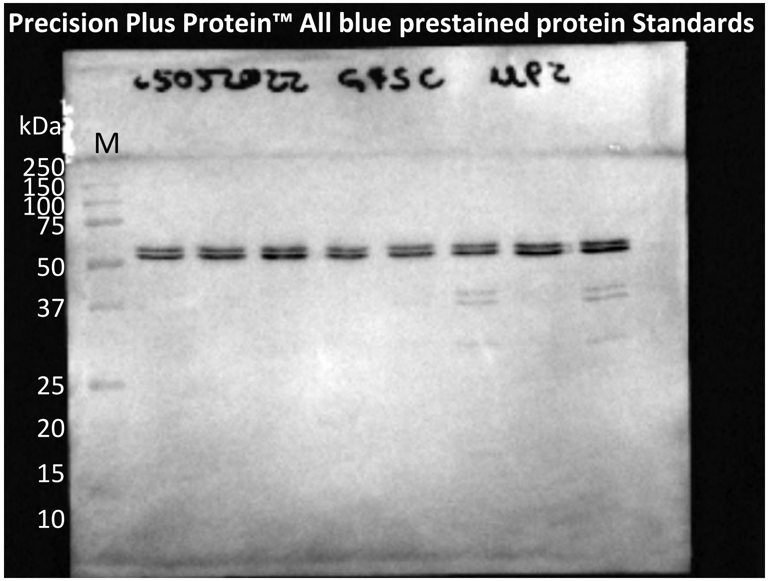

MiaPaca2

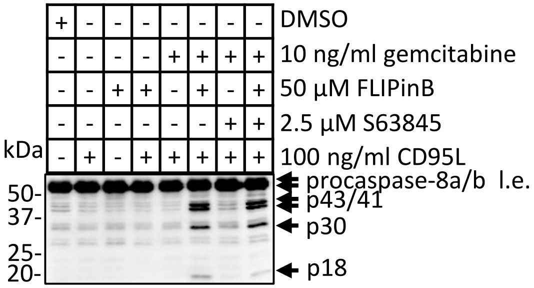

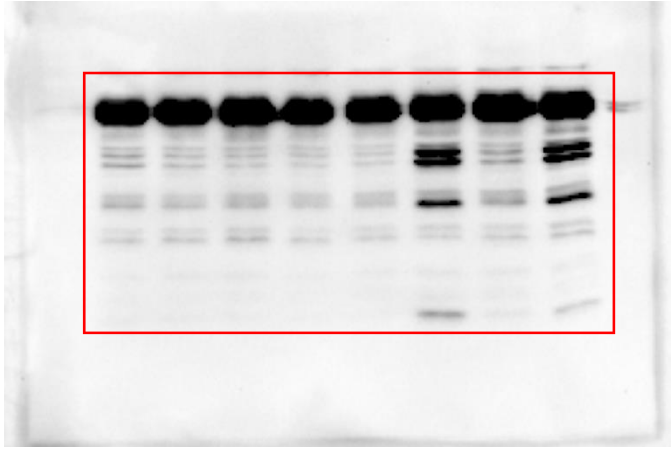

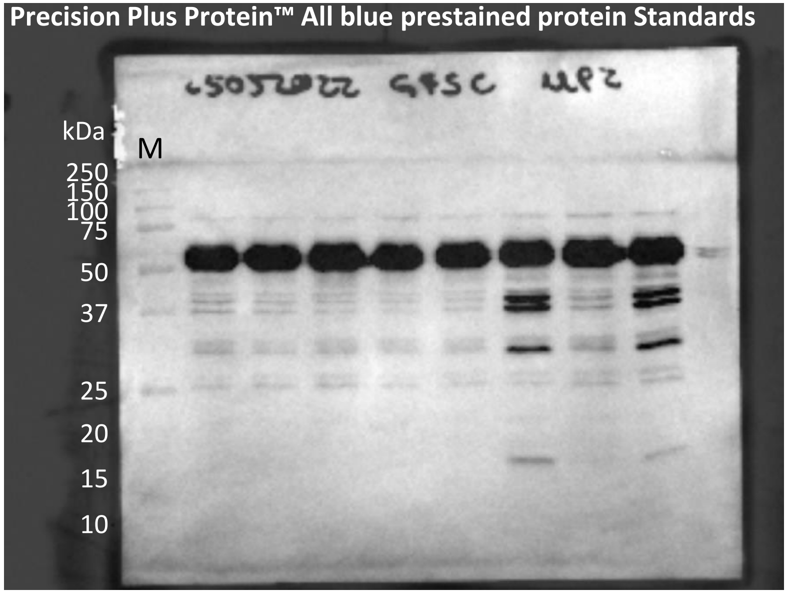

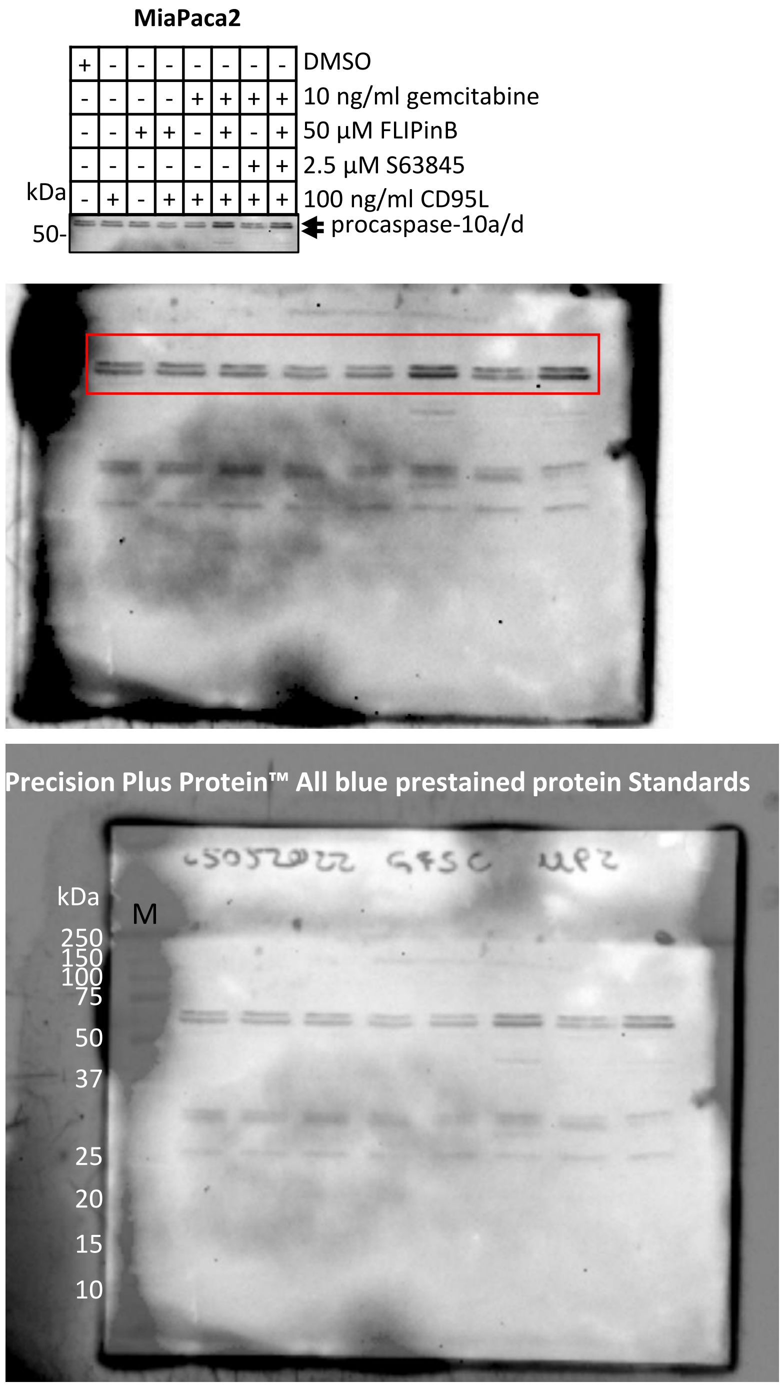
MiaPaca2
+ - - - - - - - DMSO
- - - - + + + + 10 ng/ml gemcitabine
- - + + - + - + 50 µM FLIPinB
- - - - - - + + 2.5 µM S63845
kDa - + - + + + + + 100 ng/ml CD95L
50- procaspase-10a/d
Precision Plus Protein™ All blue prestained protein Standards
kDa
M
250
150
100
75
50
37
25
20
15
10

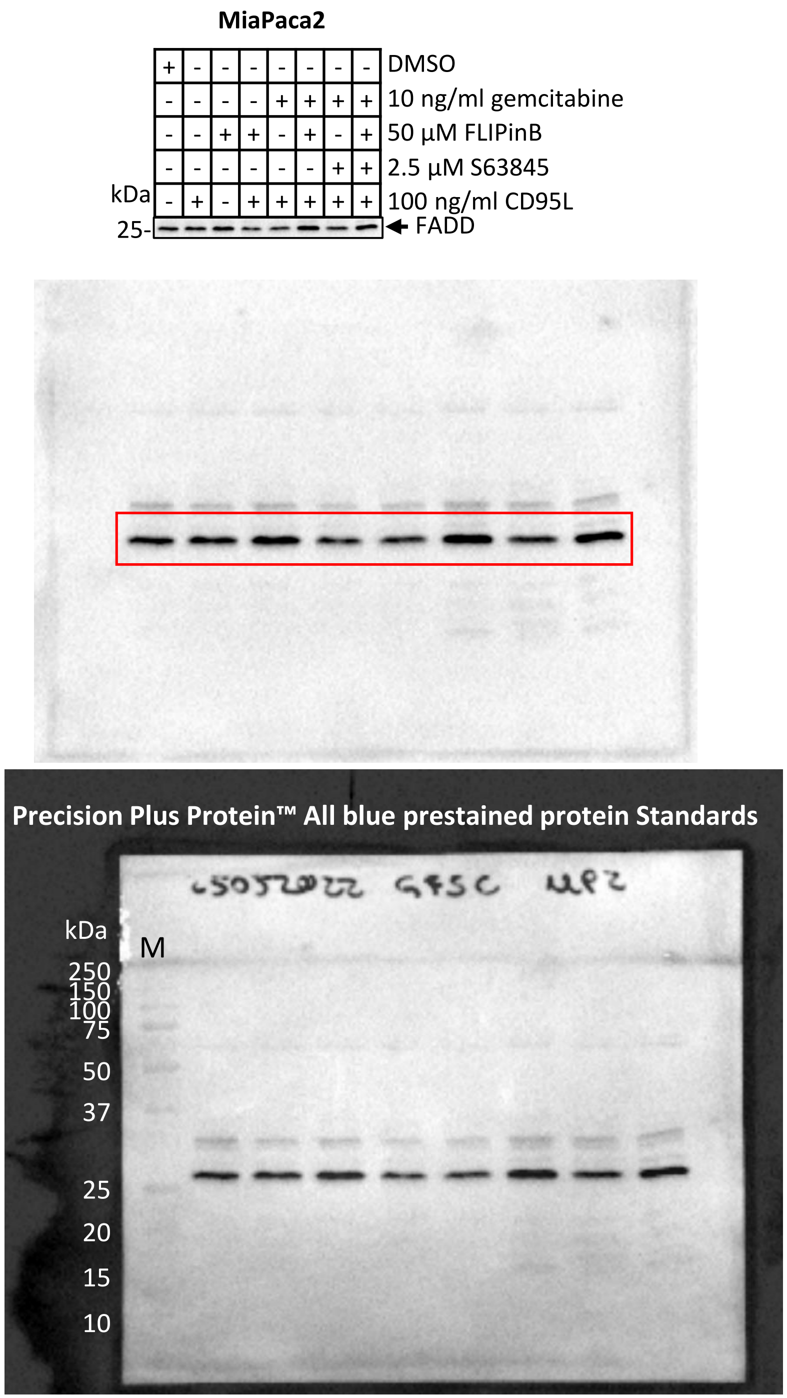
MiaPaca2
+ - - - - - - - DMSO
- - - - + + + + 10 ng/ml gemcitabine
- - + + - + - + 50 µM FLIPinB
- - - - - - + + 2.5 µM S63845
kDa - + - + + + + + 100 ng/ml CD95L
25- FADD
Precision Plus Protein™ All blue prestained protein Standards
kDa
M
250
150
100
75
50
37
25
20
15
10

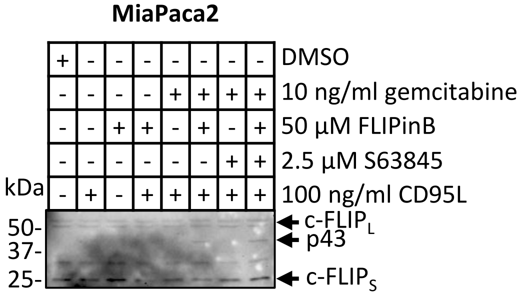
MiaPaca2
DMSO
10 ng/ml gemcitabine
50 µM FLIPinB
2.5 µM S63845
100 ng/ml CD95L
kDa
50-
37-
25-
c-FLIPL
p43
c-FLIPS

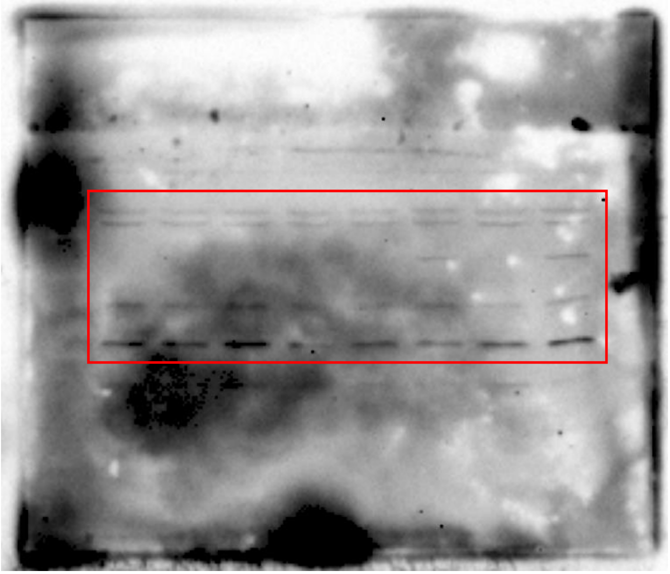

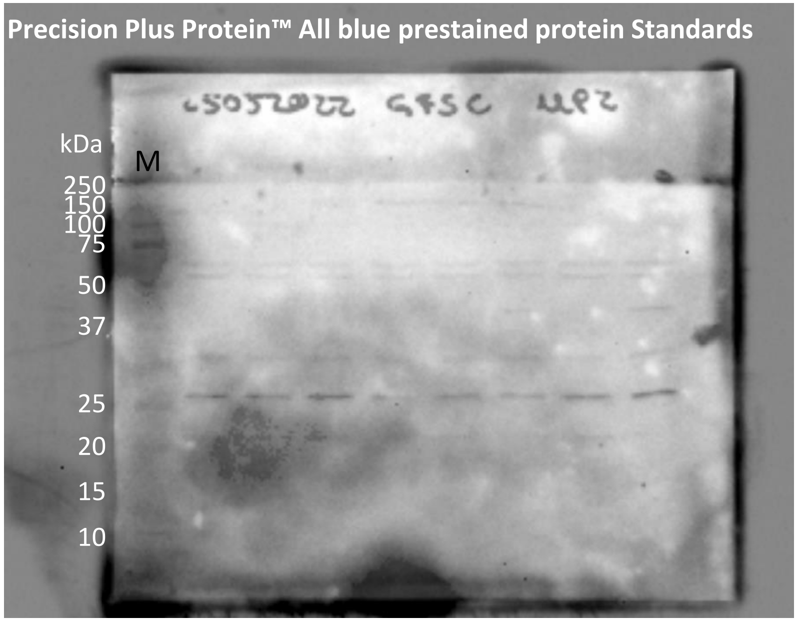
Precision Plus Protein™ All blue prestained protein Standards
kDa
M
250
150
100
75
50
37
25
20
15
10

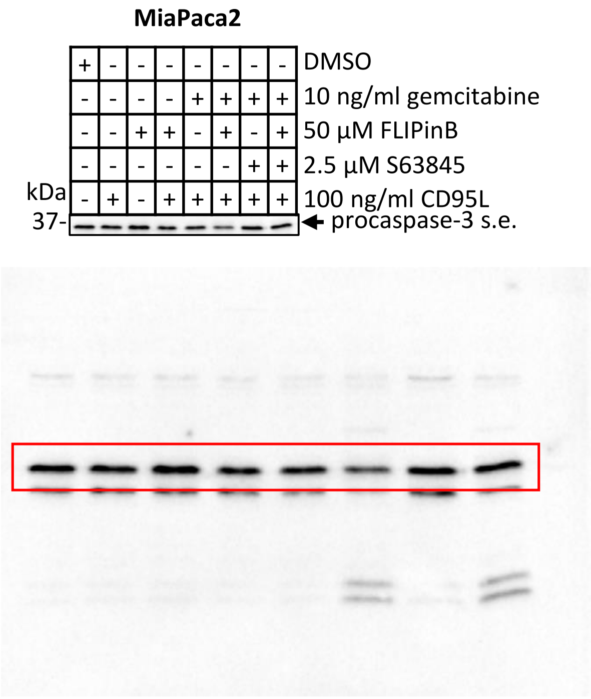
MiaPaca2
+ - - - - - - - DMSO
- - - - + + + + 10 ng/ml gemcitabine
- - + + - + - + 50 µM FLIPinB
- - - - - - + + 2.5 µM S63845
kDa - + - + + + + + 100 ng/ml CD95L
37- procaspase-3 s.e.

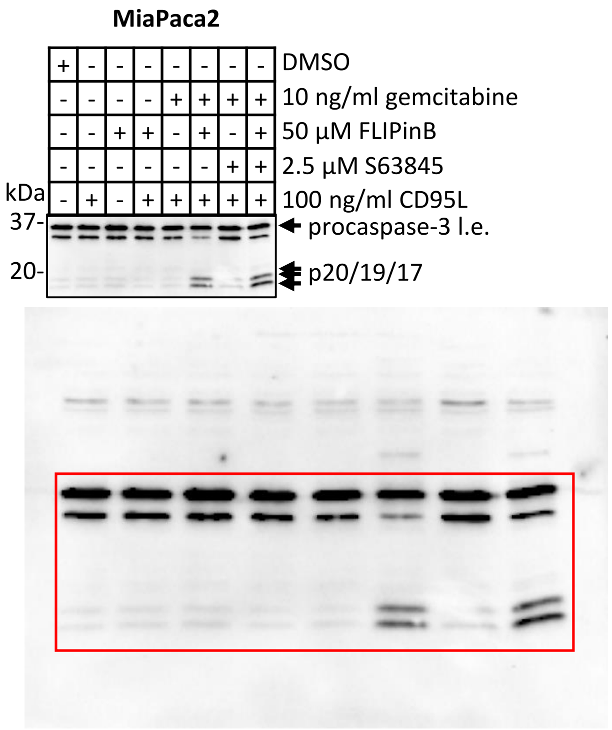
MiaPaca2
+ - - - - - - - DMSO
- - - - + + + + 10 ng/ml gemcitabine
- - + + - + - + 50 µM FLIPinB
- - - - - - + + 2.5 µM S63845
kDa - + - + + + + + 100 ng/ml CD95L
37- procaspase-3 l.e.
20- p20/19/17

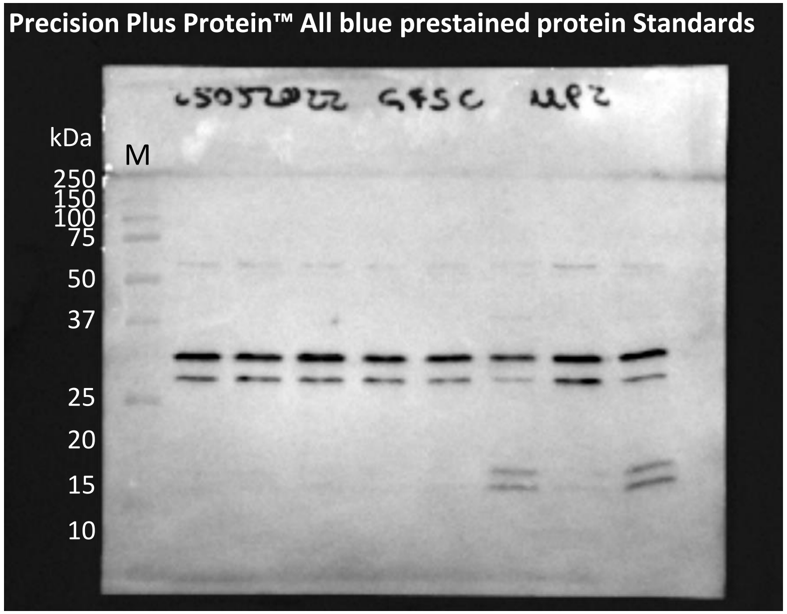
Precision Plus Protein™ All blue prestained protein Standards
kDa
M
250
150
100
75
50
37
25
20
15
10

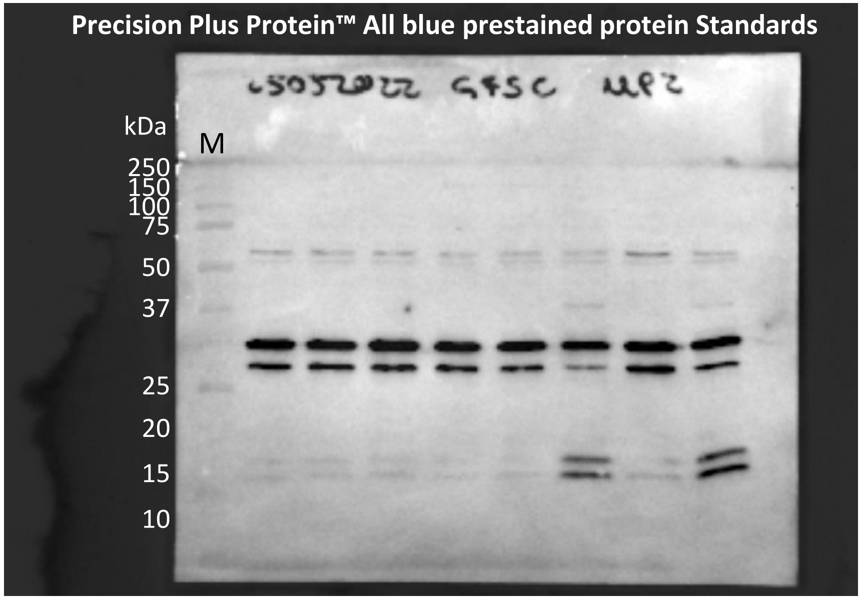
Precision Plus Protein™ All blue prestained protein Standards
kDa
M
250
150
100
75
50
37
25
20
15
10

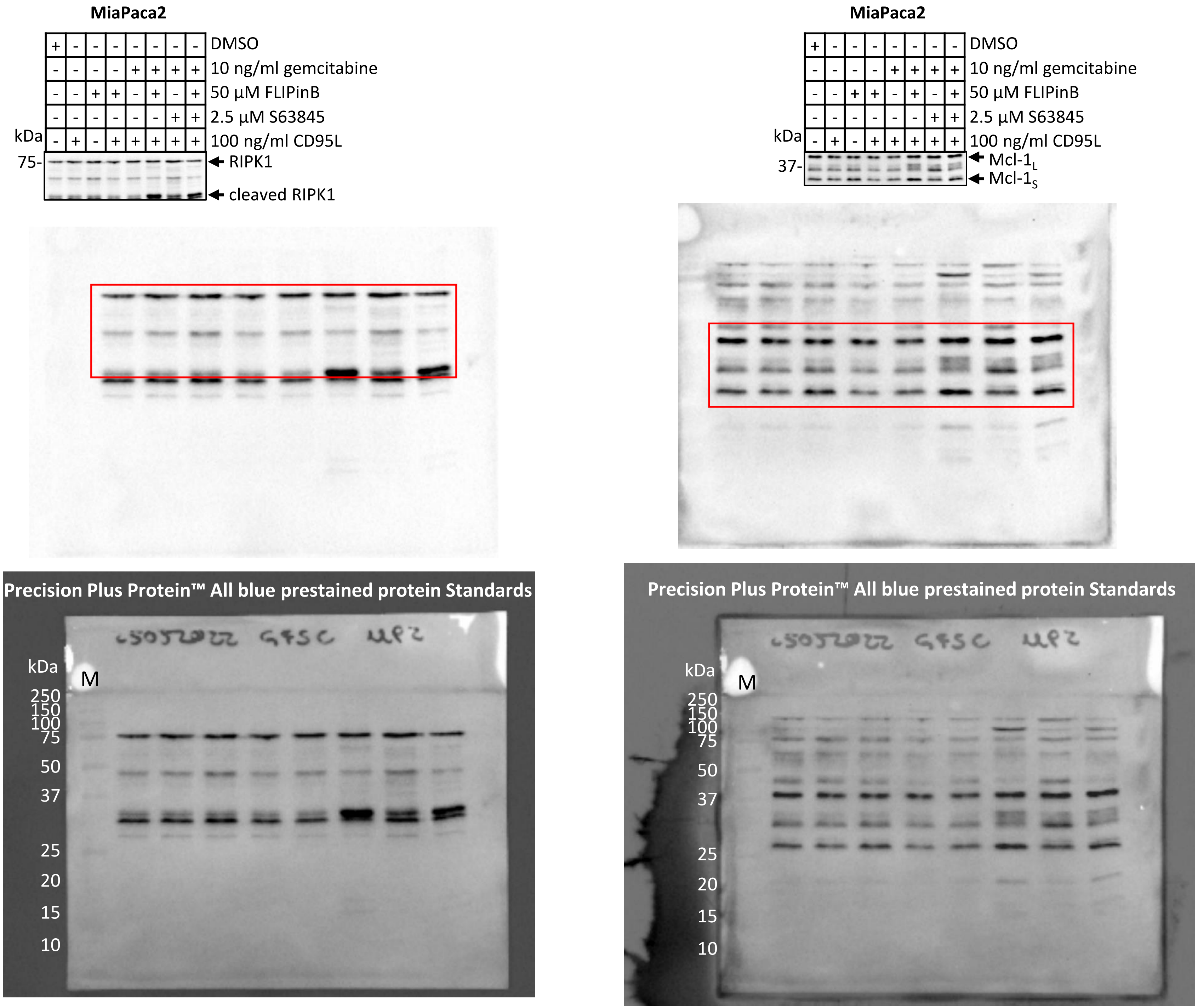
MiaPaca2
DMSO
10 ng/ml gemcitabine
50 µM FLIPinB
2.5 µM S63845
100 ng/ml CD95L
kDa
75-
RIPK1
cleaved RIPK1
Precision Plus Protein™ All blue prestained protein Standards
kDa
M
250
150
100
75
50
37
25
20
15
10
MiaPaca2
DMSO
10 ng/ml gemcitabine
50 µM FLIPinB
2.5 µM S63845
100 ng/ml CD95L
kDa
37-
Mcl-1L
Mcl-1S
Precision Plus Protein™ All blue prestained protein Standards
kDa
M
250
150
100
75
50
37
25
20
15
10

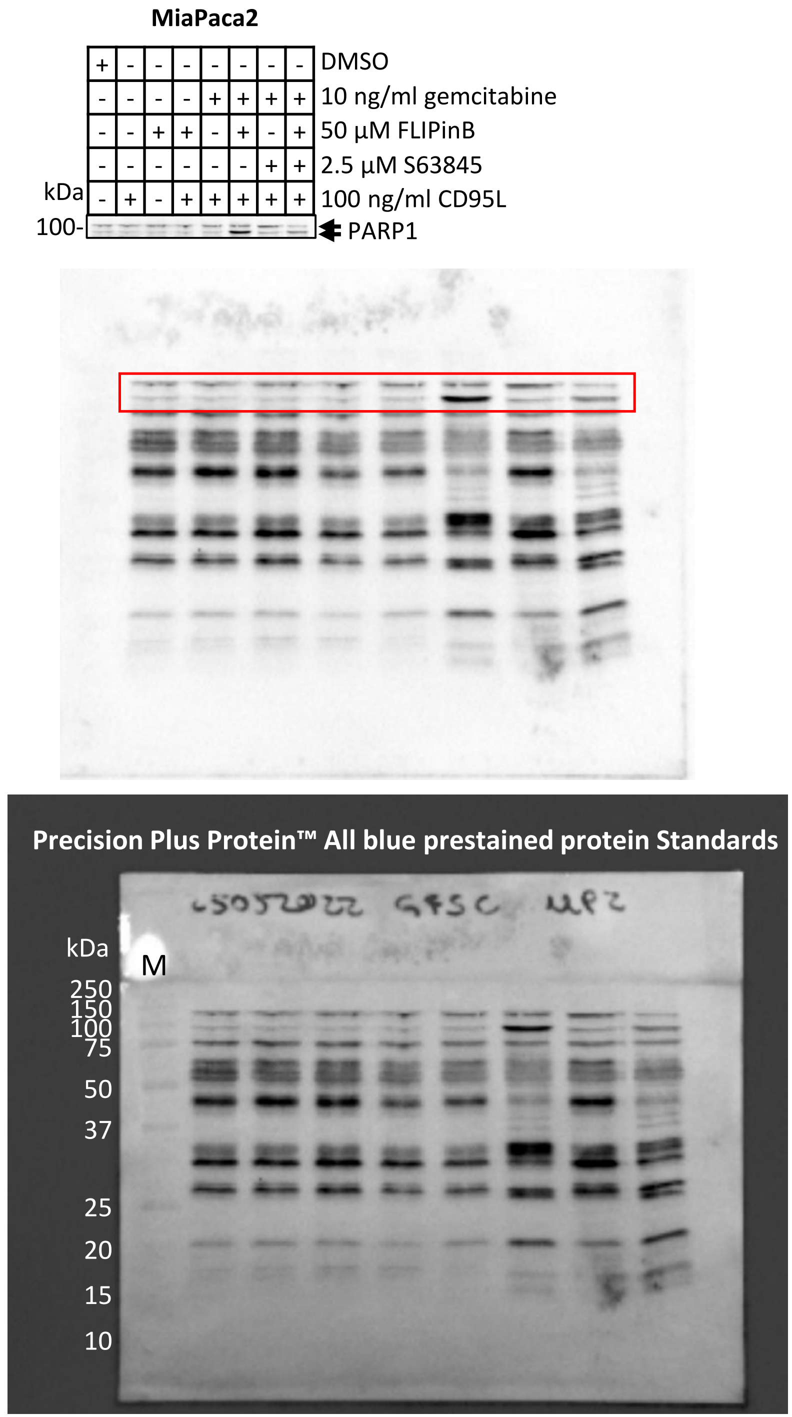
MiaPaca2
DMSO
10 ng/ml gemcitabine
50 µM FLIPinB
2.5 µM S63845
100 ng/ml CD95L
kDa
100-
PARP1
Precision Plus Protein™ All blue prestained protein Standards
kDa
M
250
150
100
75
50
37
25
20
15
10

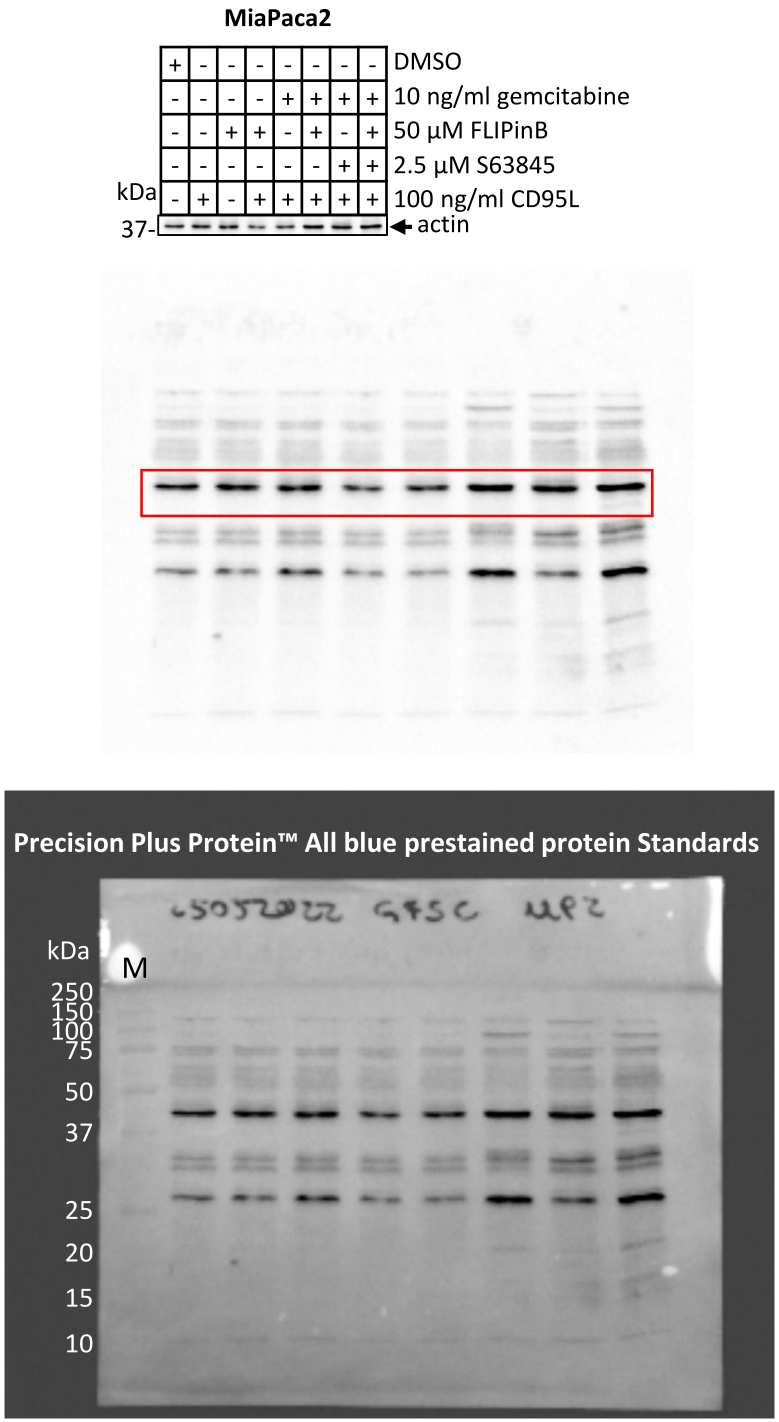
MiaPaca2
DMSO
10 ng/ml gemcitabine
50 µM FLIPinB
2.5 µM S63845
100 ng/ml CD95L
kDa
37-
actin
Precision Plus Protein™ All blue prestained protein Standards
kDa
M
250
150
100
75
50
37
25
20
15
10

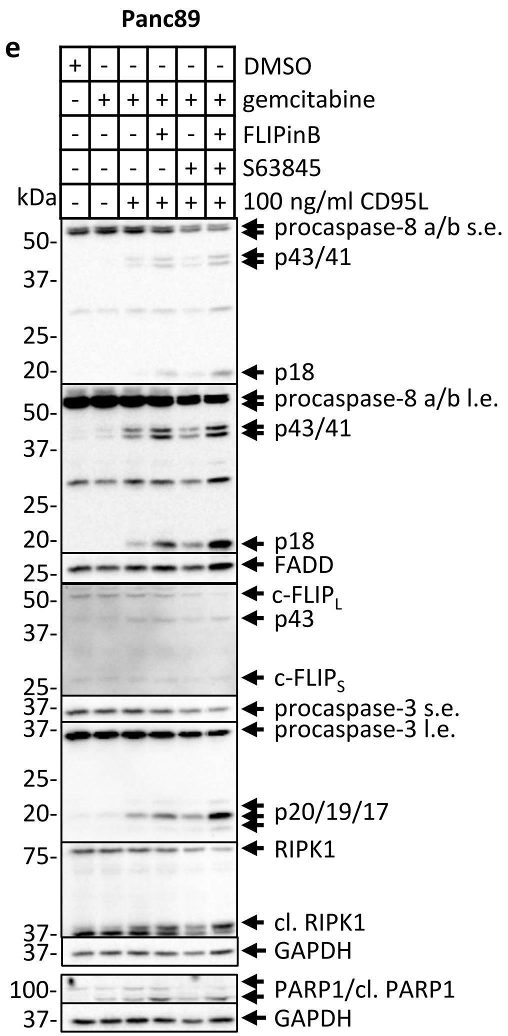
e
Panc89
DMSO
gemcitabine
FLIPinB
S63845
100 ng/ml CD95L
kDa
procaspase-8 a/b s.e.
p43/41
p18
procaspase-8 a/b l.e.
p43/41
p18
FADD
c-FLIP_L
p43
c-FLIP_S
procaspase-3 s.e.
procaspase-3 l.e.
p20/19/17
RIPK1
cl. RIPK1
GAPDH
PARP1/cl. PARP1
GAPDH

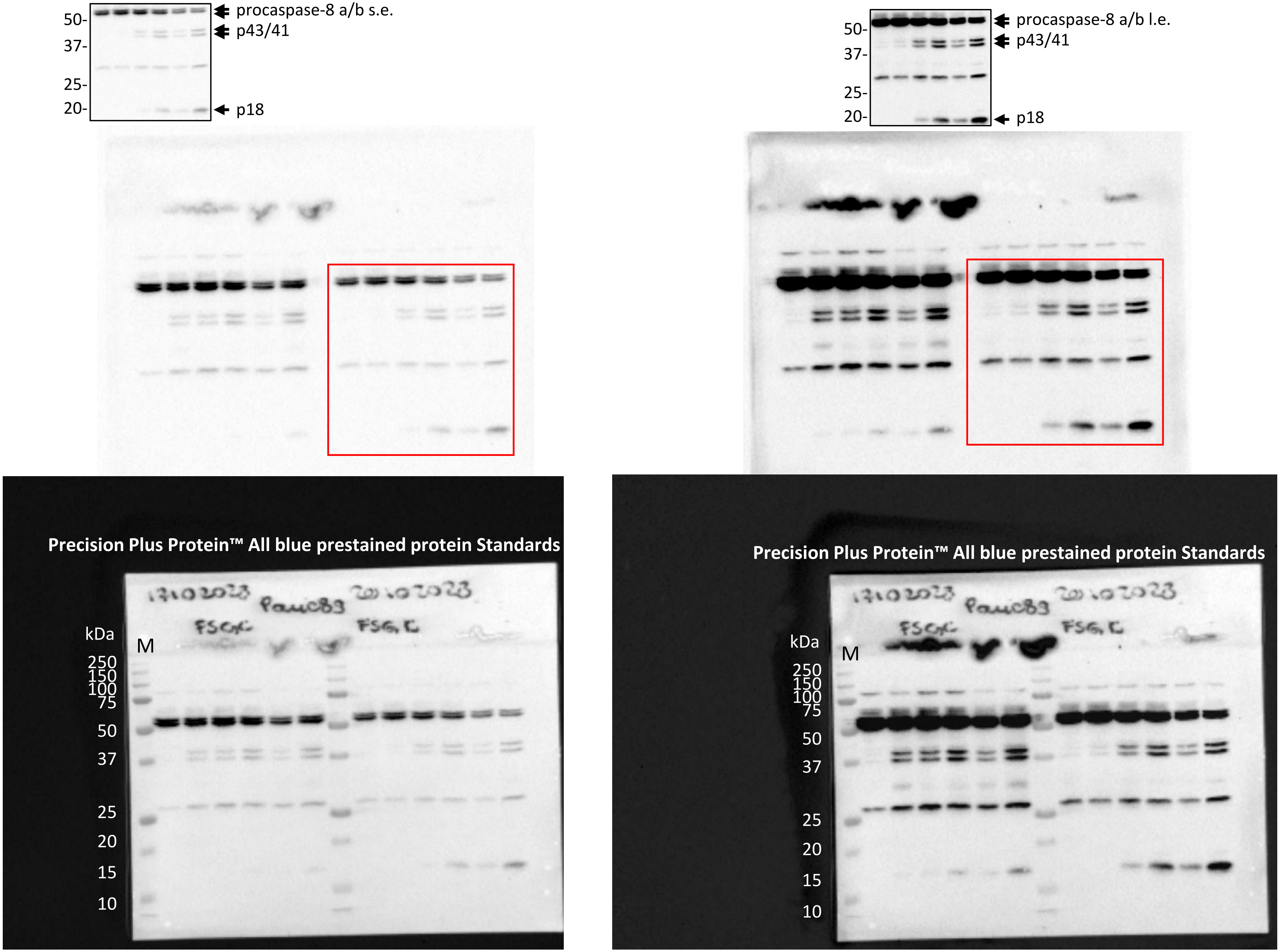

50-
37-
25-
20-
procaspase-8 a/b s.e.
p43/41
p18
50-
37-
25-
20-
procaspase-8 a/b l.e.
p43/41
p18
Precision Plus Protein™ All blue prestained protein Standards
kDa
M
250
150
100
75
50
37
25
20
15
10
Precision Plus Protein™ All blue prestained protein Standards
kDa
M
250
150
100
75
50
37
25
20
15
10

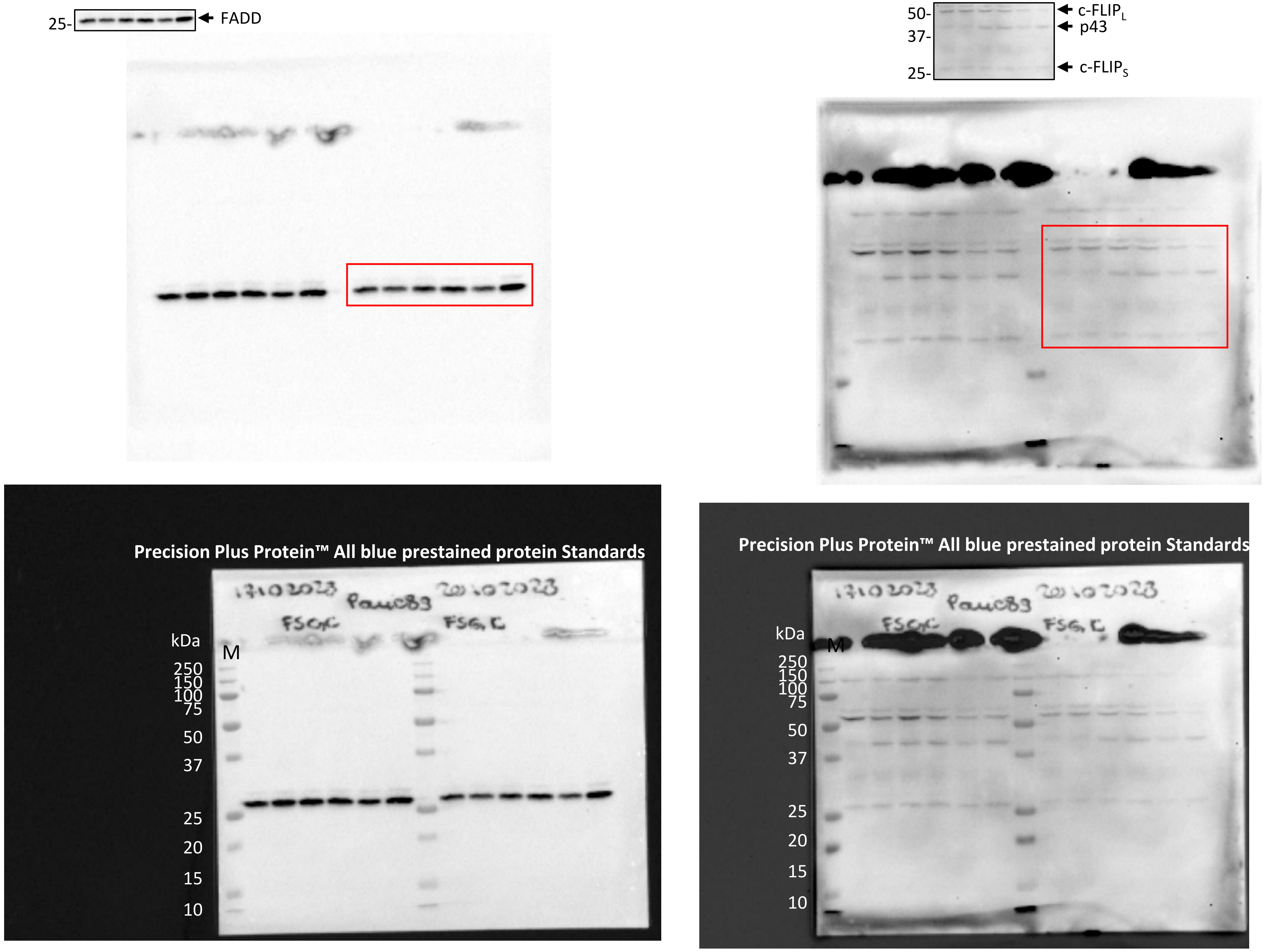

25-
FADD
50-
37-
25-
c-FLIP_L
p43
c-FLIP_S
Precision Plus Protein™ All blue prestained protein Standards
kDa
M
250
150
100
75
50
37
25
20
15
10
Precision Plus Protein™ All blue prestained protein Standards
kDa
M
250
150
100
75
50
37
25
20
15
10

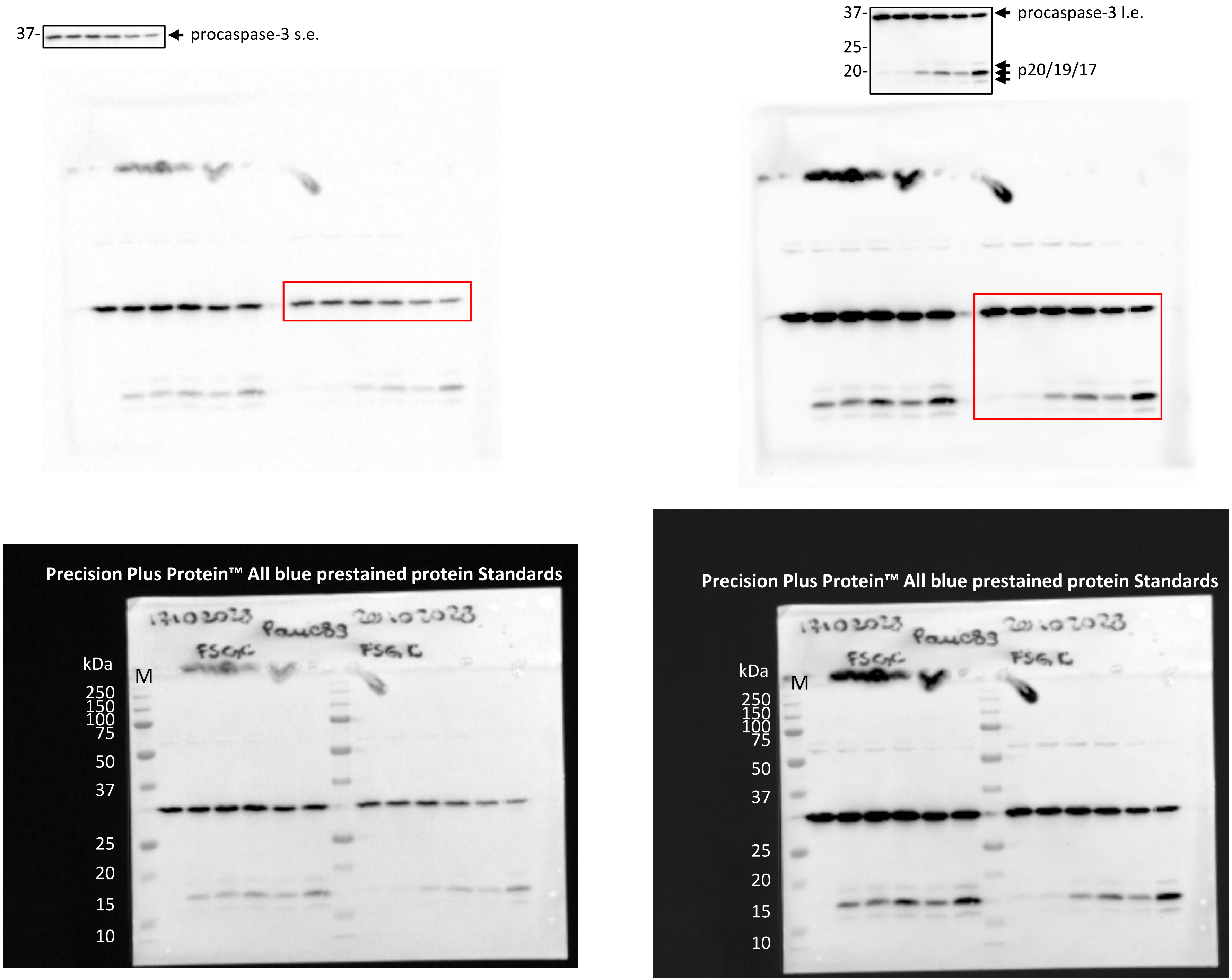
37-
procaspase-3 s.e.
37-
25-
20-
procaspase-3 l.e.
p20/19/17
Precision Plus Protein™ All blue prestained protein Standards
kDa
M
250
150
100
75
50
37
25
20
15
10
Precision Plus Protein™ All blue prestained protein Standards
kDa
M
250
150
100
75
50
37
25
20
15
10

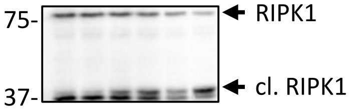
75-
37-
RIPK1
cl. RIPK1

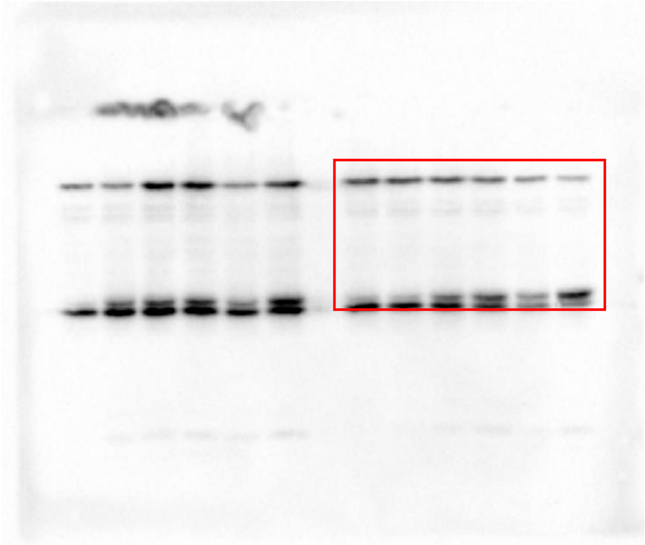

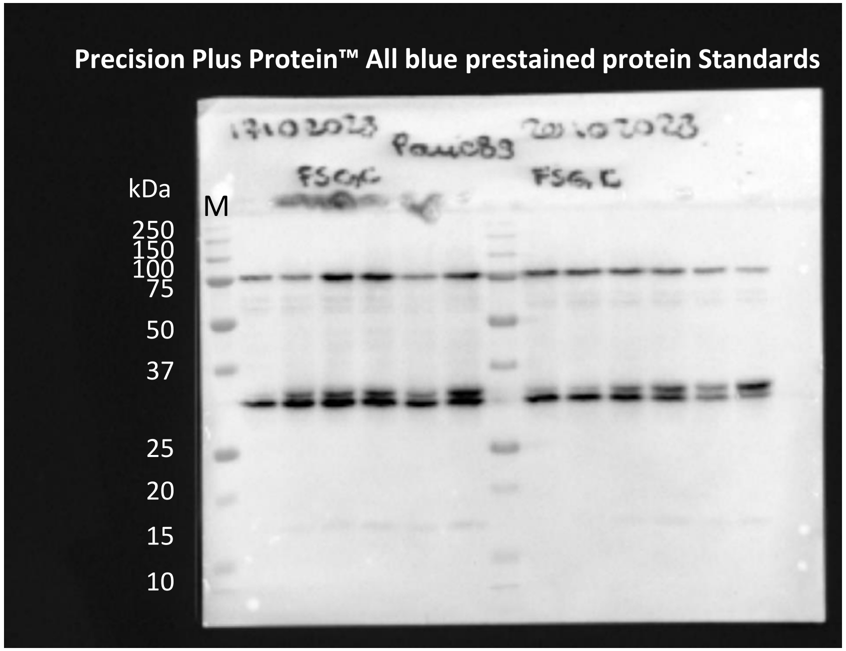
Precision Plus Protein™ All blue prestained protein Standards
kDa
M
250
150
100
75
50
37
25
20
15
10

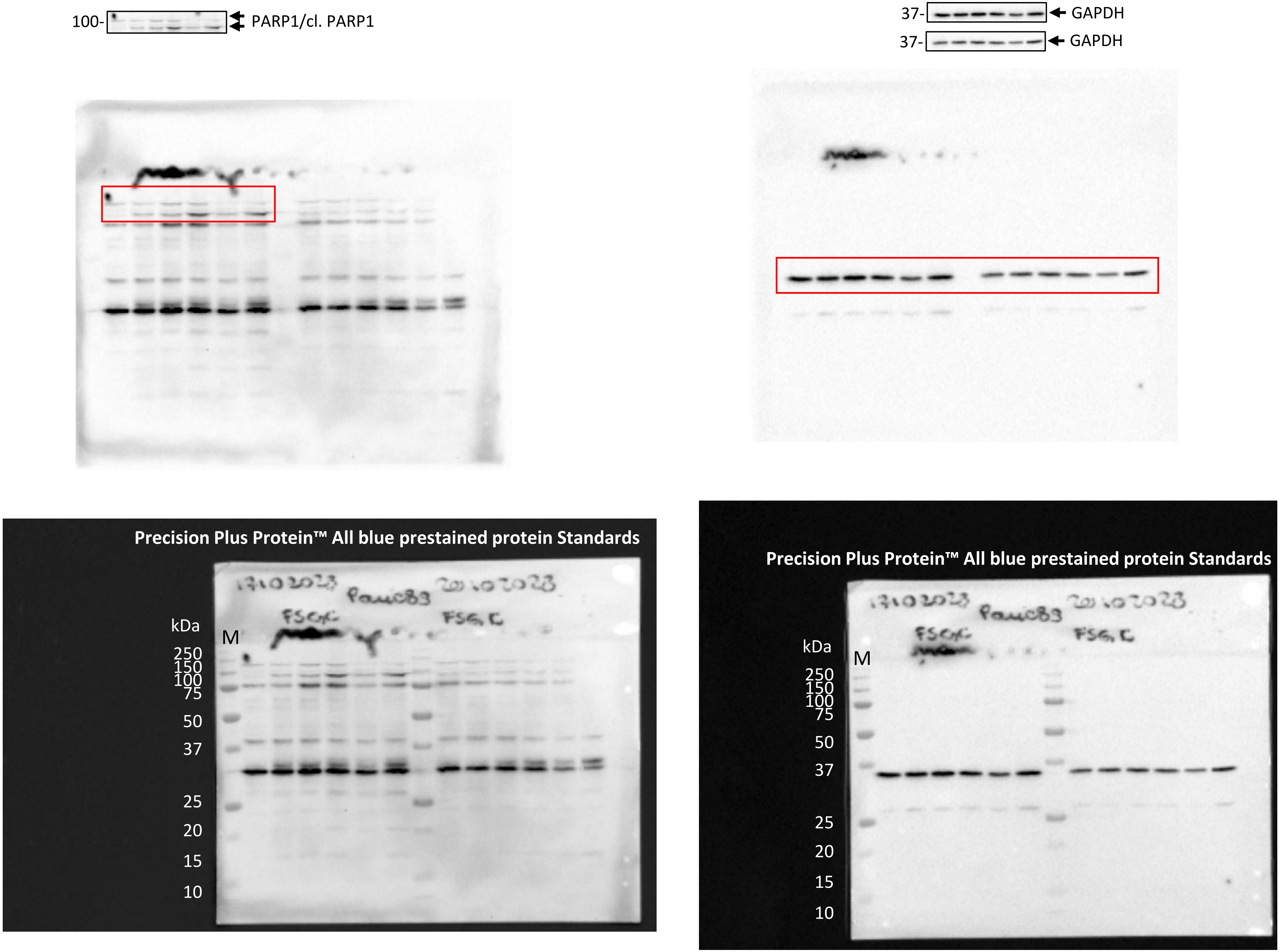
100-
PARP1/cl. PARP1
37-
GAPDH
37-
GAPDH
Precision Plus Protein™ All blue prestained protein Standards
kDa
M
250
150
100
75
50
37
25
20
15
10
Precision Plus Protein™ All blue prestained protein Standards
kDa
M
250
150
100
75
50
37
25
20
15
10

MiaPaca2

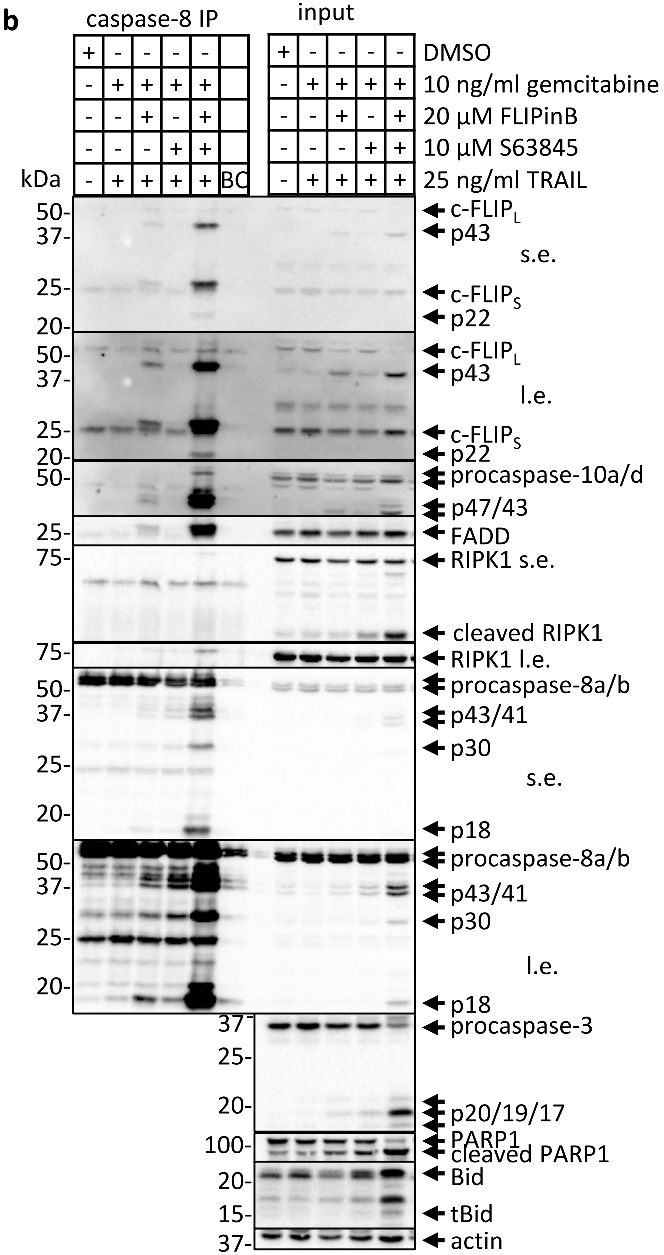

b
caspase-8 IP
input
DMSO
10 ng/ml gemcitabine
20 µM FLIPinB
10 µM S63845
25 ng/ml TRAIL
kDa
BC
c-FLIPL
p43
s.e.
c-FLIPS
p22
l.e.
procaspase-10a/d
p47/43
FADD
RIPK1 s.e.
cleaved RIPK1
RIPK1 l.e.
procaspase-8a/b
p43/41
p30
p18
procaspase-3
p20/19/17
PARP1
cleaved PARP1
Bid
tBid
actin

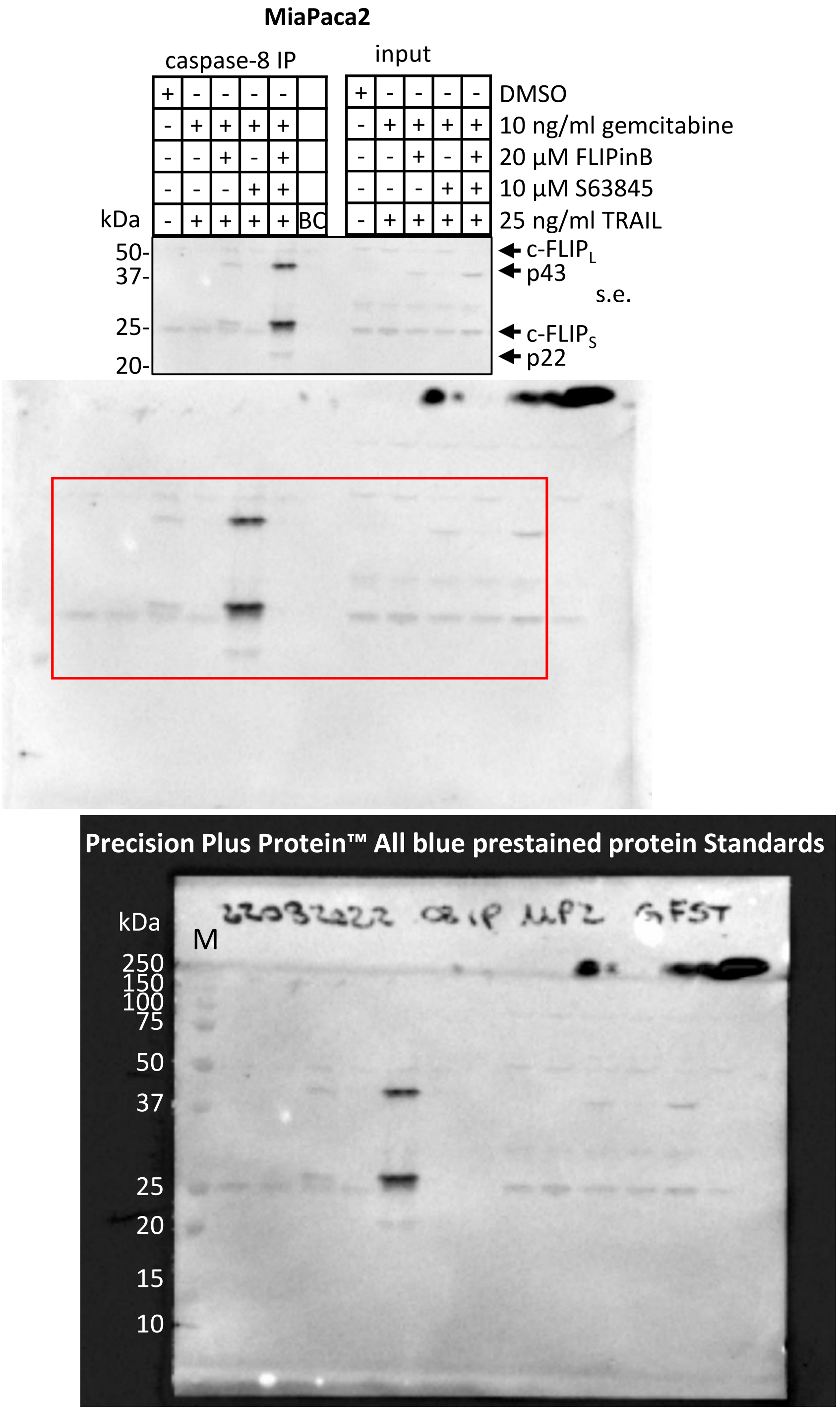
MiaPaca2
caspase-8 IP
input
DMSO
10 ng/ml gemcitabine
20 µM FLIPinB
10 µM S63845
25 ng/ml TRAIL
kDa
BC
50-
37-
25-
20-
c-FLIPL
p43
s.e.
c-FLIPS
p22
Precision Plus Protein™ All blue prestained protein Standards
kDa
M
250
150
100
75
50
37
25
20
15
10

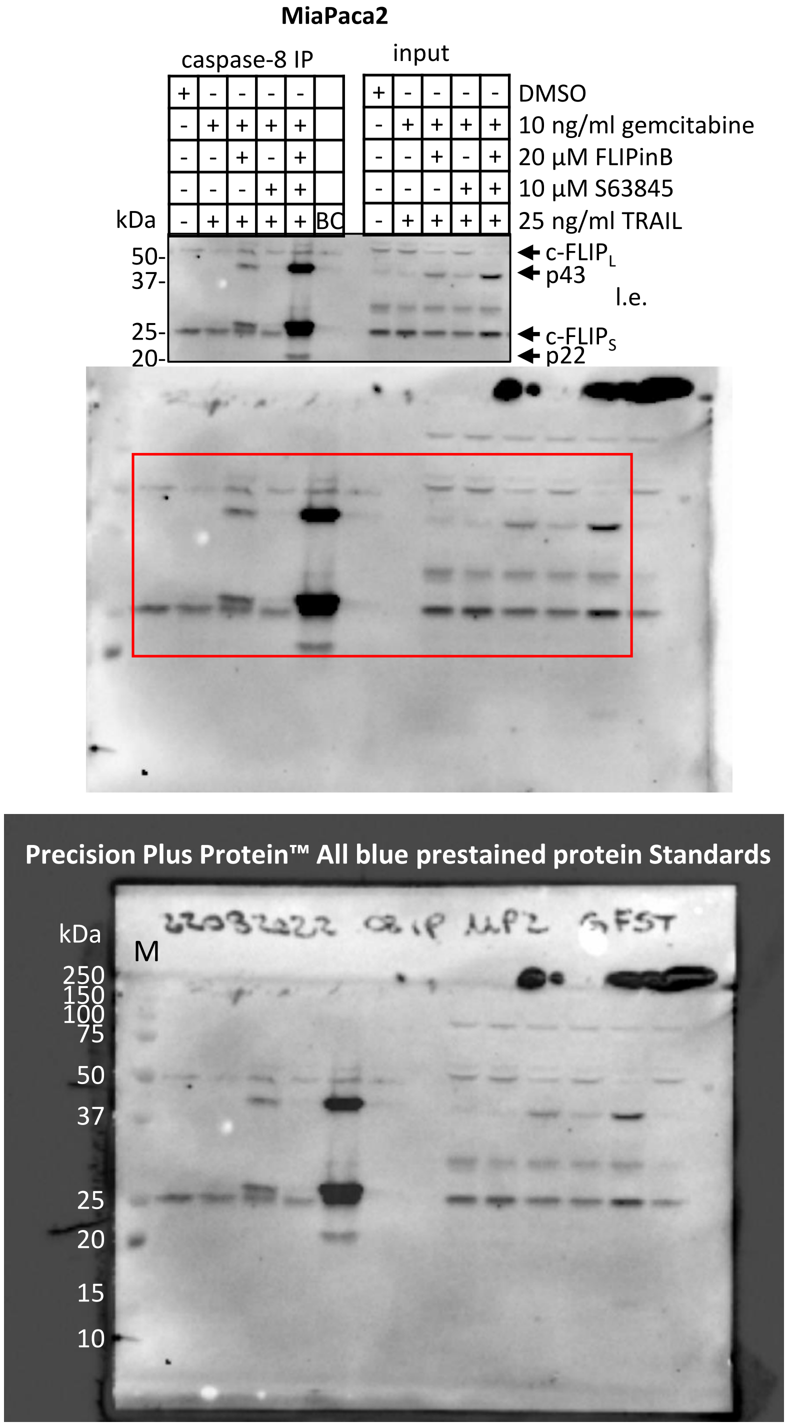
MiaPaca2
caspase-8 IP
input
DMSO
10 ng/ml gemcitabine
20 µM FLIPinB
10 µM S63845
25 ng/ml TRAIL
kDa
BC
50-
37-
25-
20-
c-FLIPL
p43
l.e.
c-FLIPS
p22
Precision Plus Protein™ All blue prestained protein Standards
kDa
M
250
150
100
75
50
37
25
20
15
10

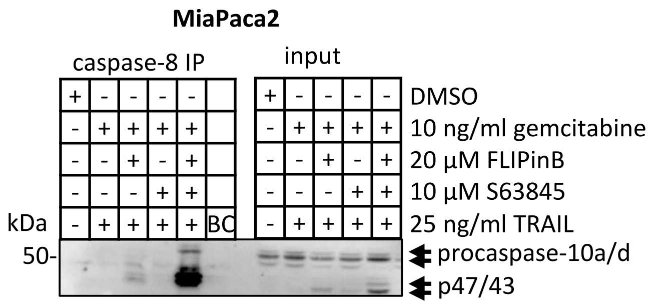
MiaPaca2
caspase-8 IP
input
DMSO
10 ng/ml gemcitabine
20 μM FLIPinB
10 μM S63845
25 ng/ml TRAIL
kDa
BC
50-
procaspase-10a/d
p47/43

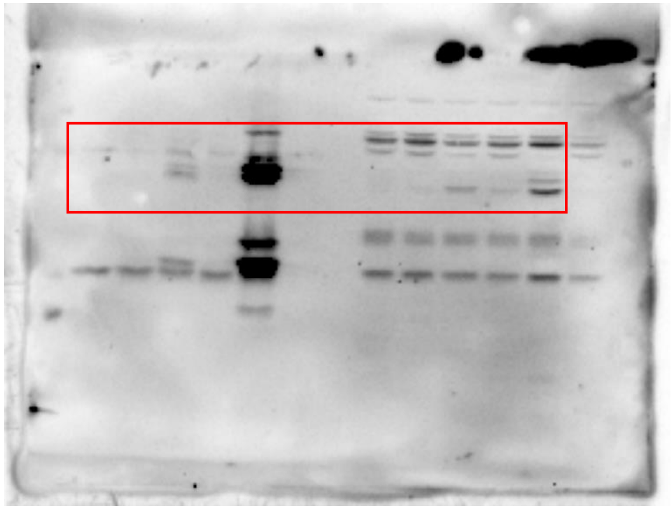

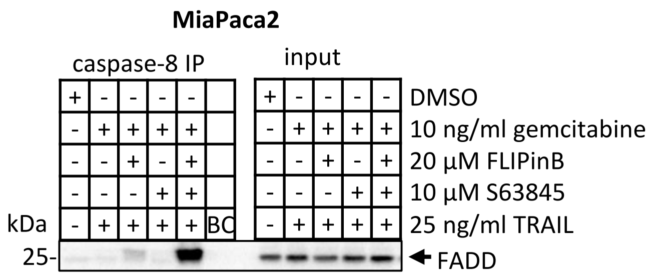
MiaPaca2
caspase-8 IP
input
DMSO
10 ng/ml gemcitabine
20 μM FLIPinB
10 μM S63845
25 ng/ml TRAIL
kDa
BC
25-
FADD

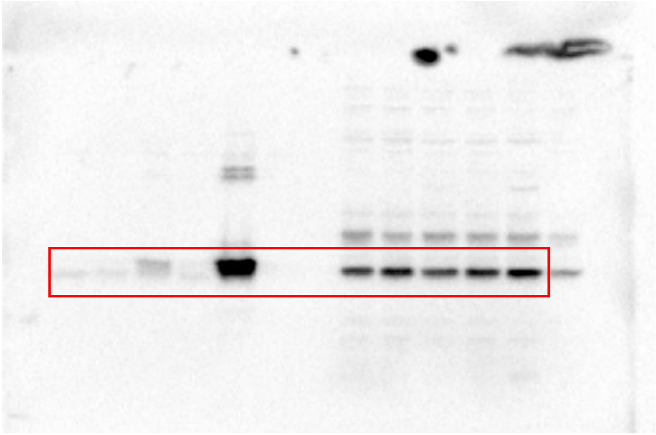

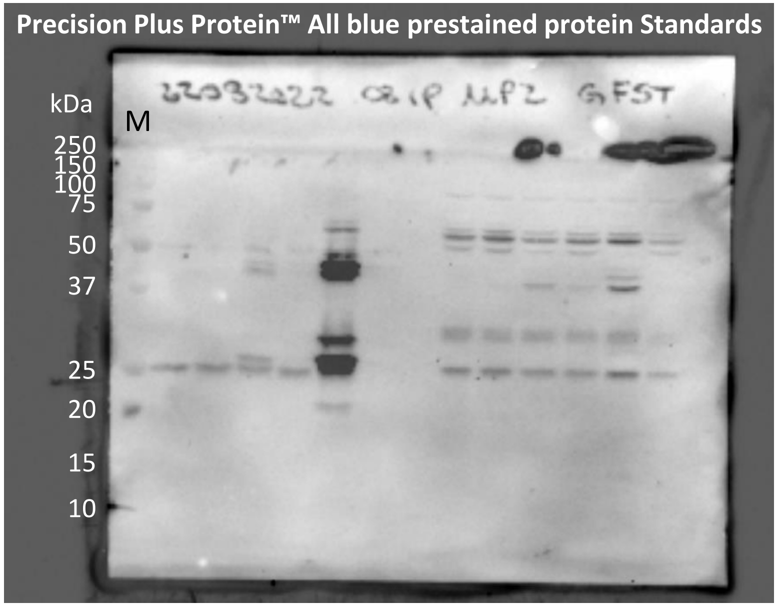
Precision Plus Protein™ All blue prestained protein Standards
kDa
M
250
150
100
75
50
37
25
20
15
10

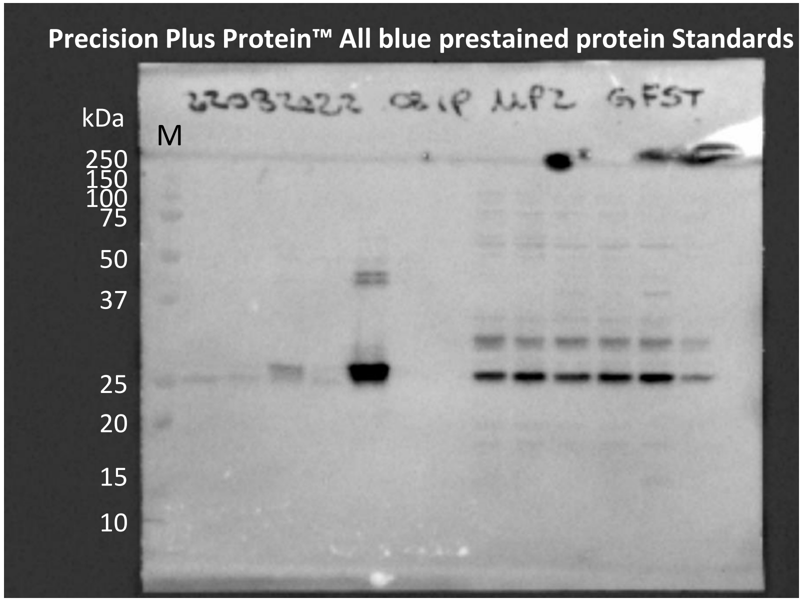
Precision Plus Protein™ All blue prestained protein Standards
kDa
M
250
150
100
75
50
37
25
20
15
10

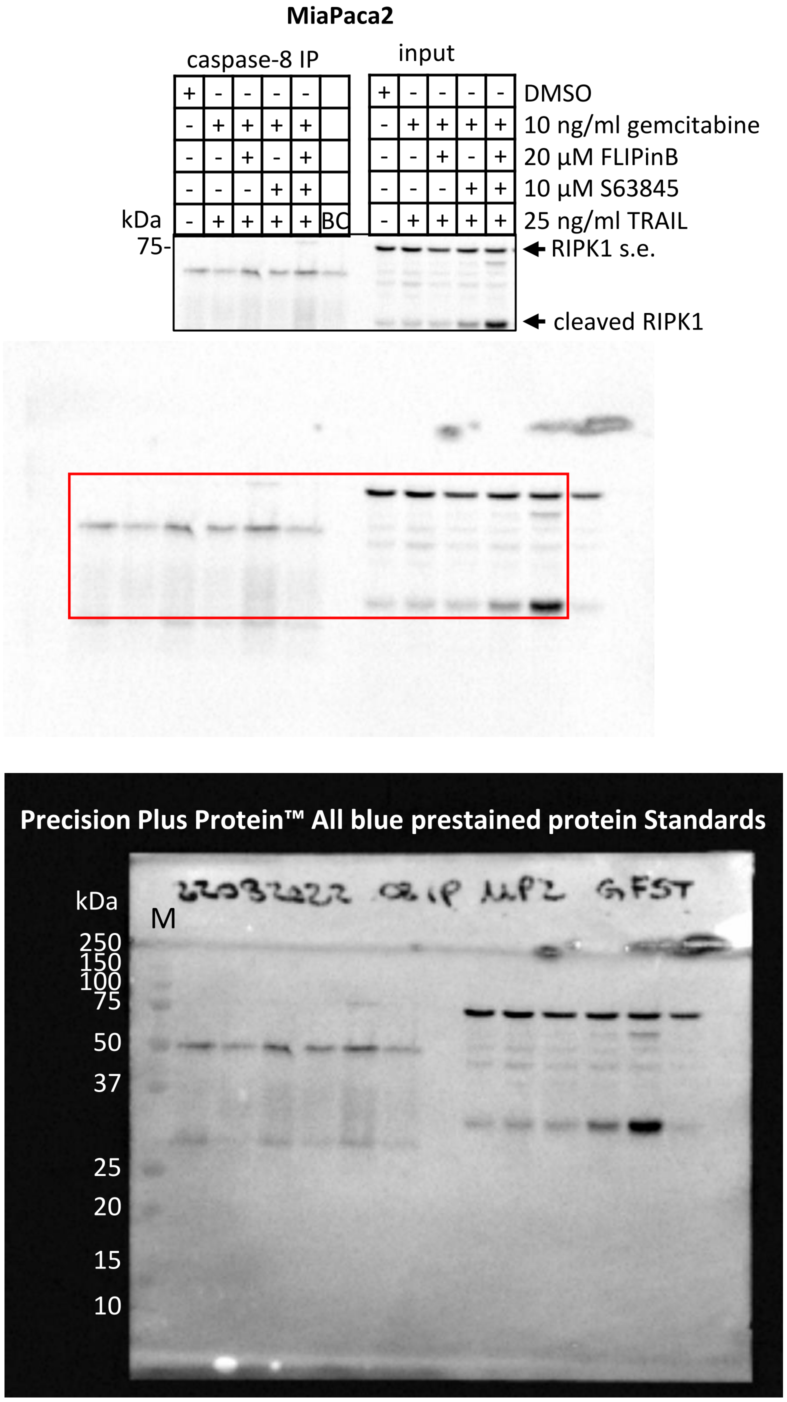
MiaPaca2
caspase-8 IP
input
DMSO
10 ng/ml gemcitabine
20 µM FLIPinB
10 µM S63845
25 ng/ml TRAIL
kDa
75-
BC
RIPK1 s.e.
cleaved RIPK1
Precision Plus Protein™ All blue prestained protein Standards
kDa
M
250
150
100
75
50
37
25
20
15
10

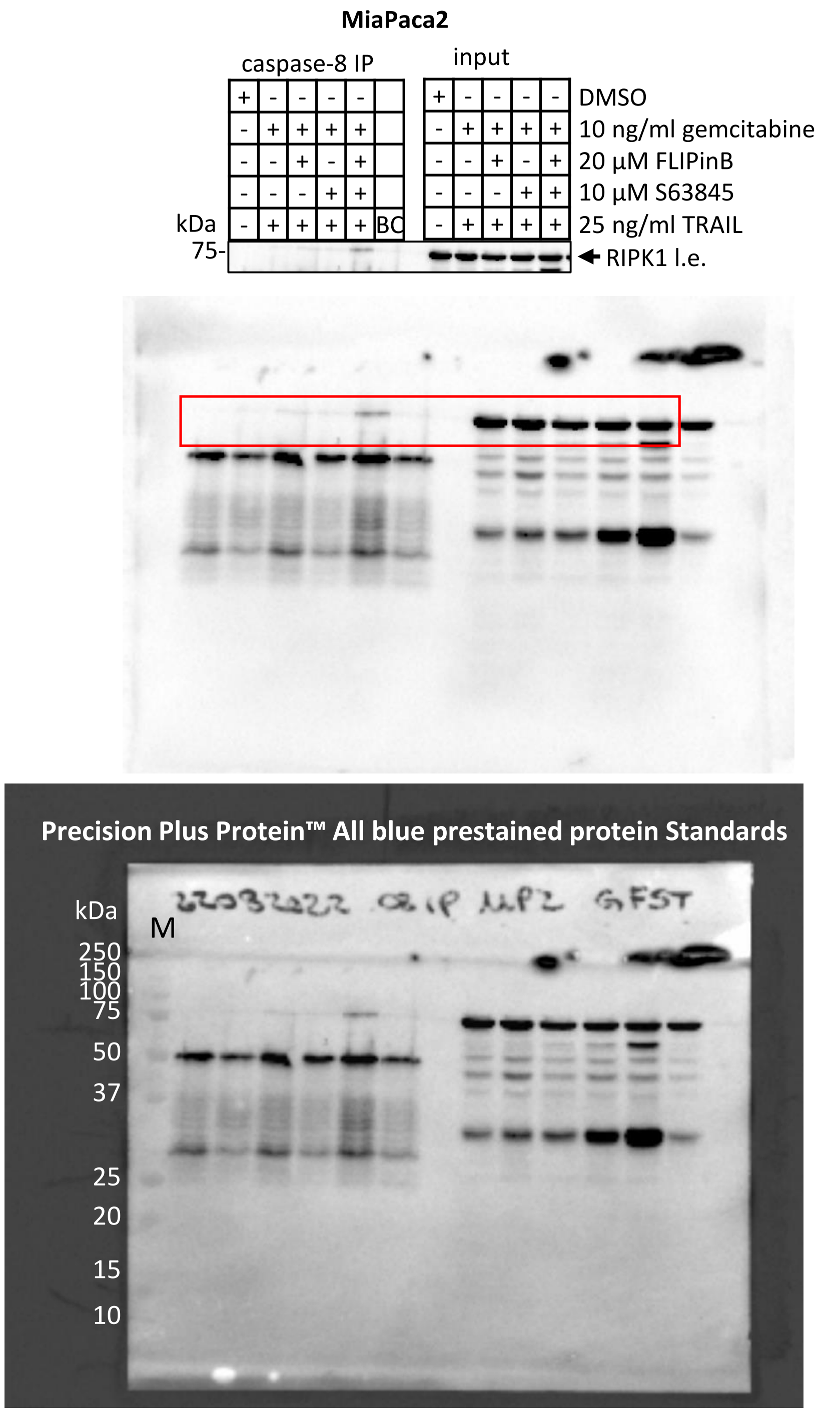
MiaPaca2
caspase-8 IP
input
DMSO
10 ng/ml gemcitabine
20 µM FLIPinB
10 µM S63845
25 ng/ml TRAIL
kDa
75-
BC
RIPK1 l.e.
Precision Plus Protein™ All blue prestained protein Standards
kDa
M
250
150
100
75
50
37
25
20
15
10

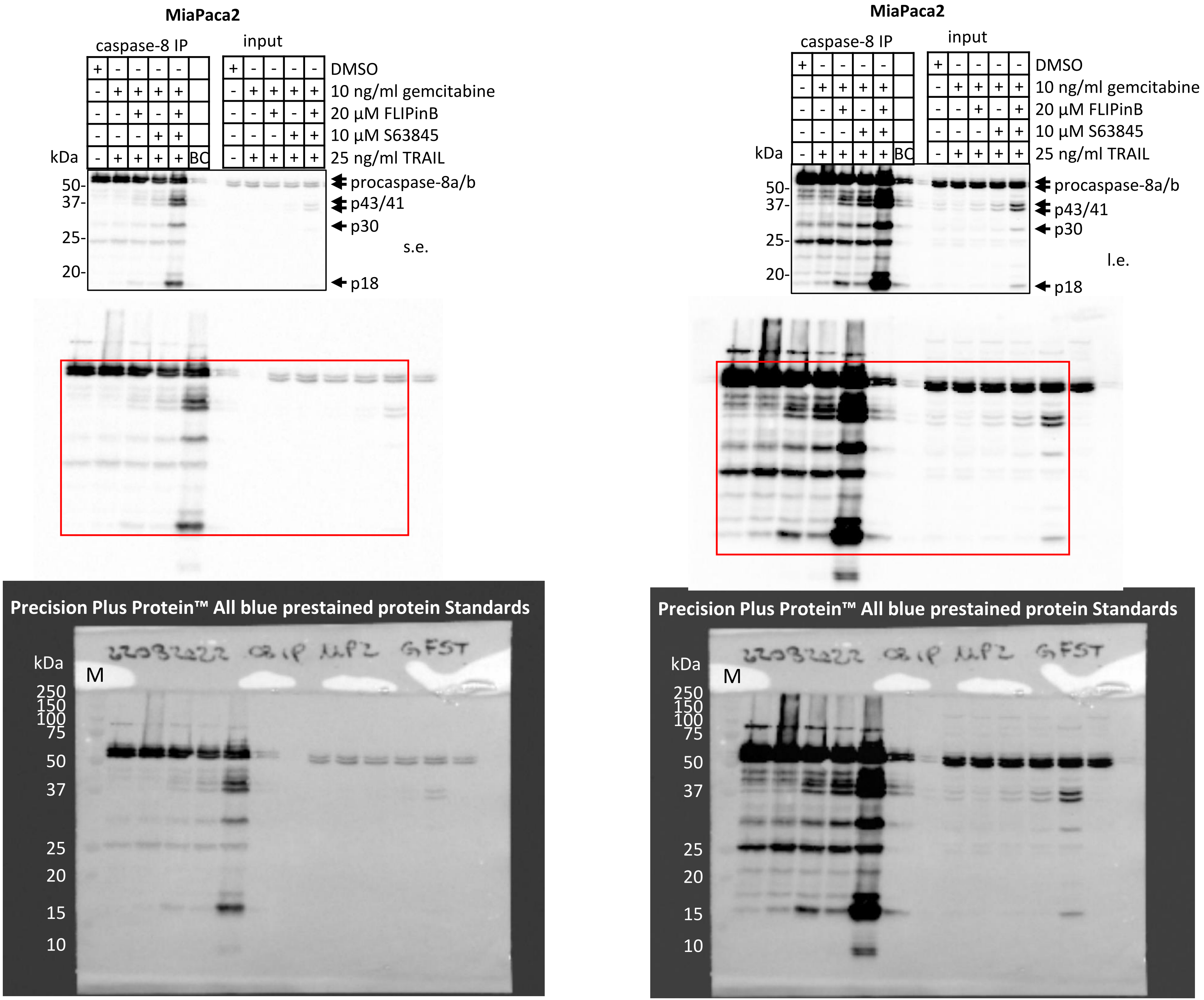

MiaPaca2
caspase-8 IP
input
DMSO
10 ng/ml gemcitabine
20 µM FLIPinB
10 µM S63845
25 ng/ml TRAIL
kDa
BC
50-
37-
25-
20-
procaspase-8a/b
p43/41
p30
p18
s.e.
Precision Plus Protein™ All blue prestained protein Standards
kDa
M
250
150
100
75
50
37
25
20
15
10
MiaPaca2
caspase-8 IP
input
DMSO
10 ng/ml gemcitabine
20 µM FLIPinB
10 µM S63845
25 ng/ml TRAIL
kDa
BC
50-
37-
25-
20-
procaspase-8a/b
p43/41
p30
p18
l.e.
Precision Plus Protein™ All blue prestained protein Standards
kDa
M
250
150
100
75
50
37
25
20
15
10

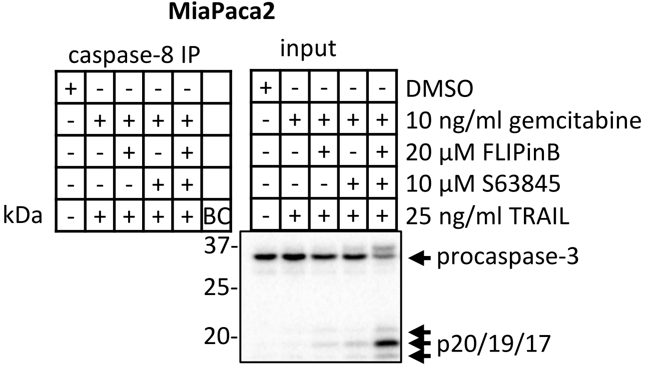
MiaPaca2
caspase-8 IP
input
DMSO
10 ng/ml gemcitabine
20 µM FLIPinB
10 µM S63845
25 ng/ml TRAIL
kDa
BC
37-
25-
20-
procaspase-3
p20/19/17

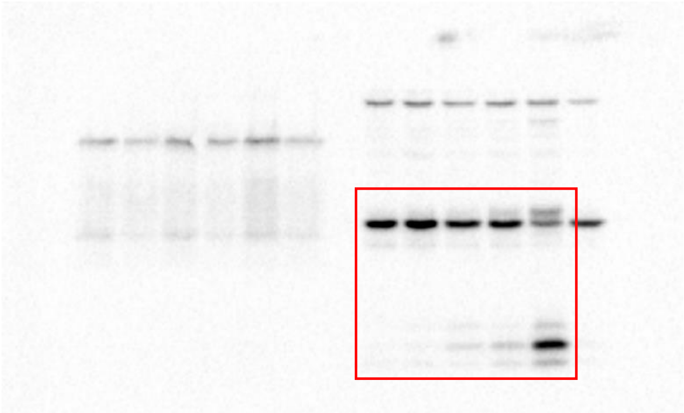

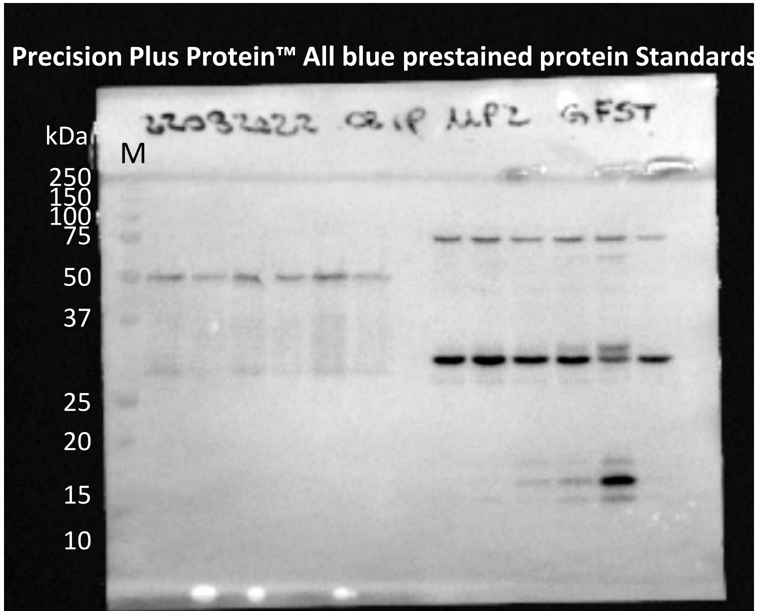
Precision Plus Protein™ All blue prestained protein Standards
kDa
M
250
150
100
75
50
37
25
20
15
10

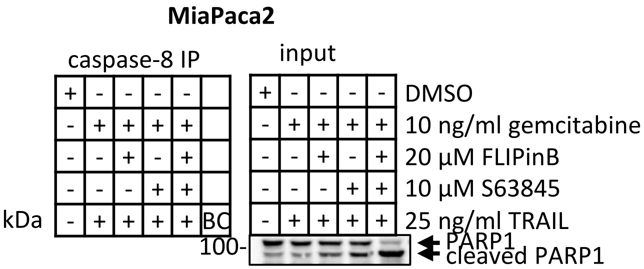
MiaPaca2
caspase-8 IP
input
DMSO
10 ng/ml gemcitabine
20 µM FLIPinB
10 µM S63845
25 ng/ml TRAIL
kDa
BC
100-
PARP1
cleaved PARP1

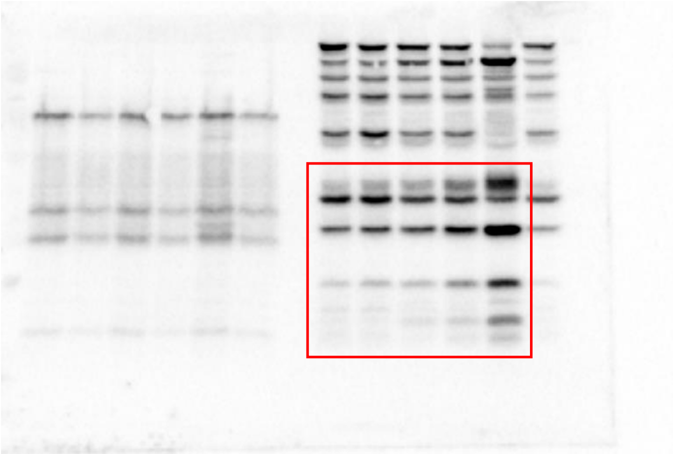

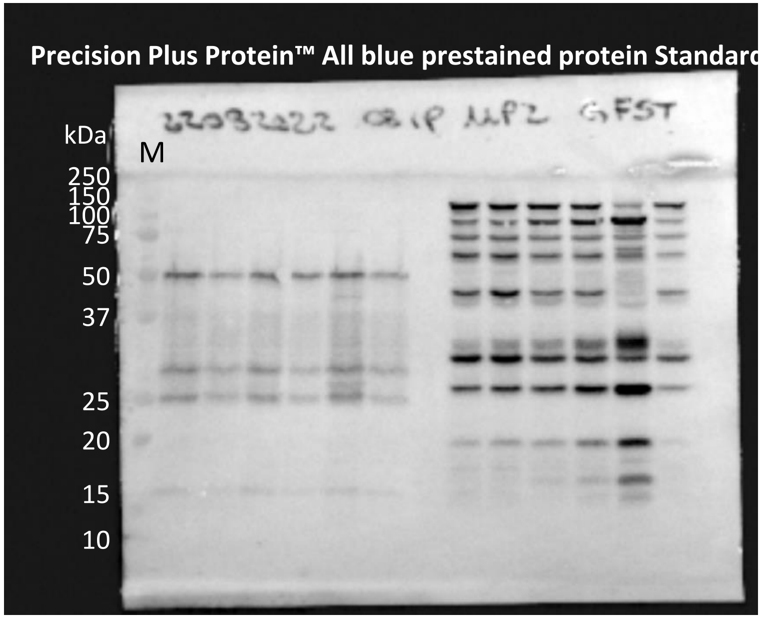
Precision Plus Protein™ All blue prestained protein Standard
kDa
M
250
150
100
75
50
37
25
20
15
10

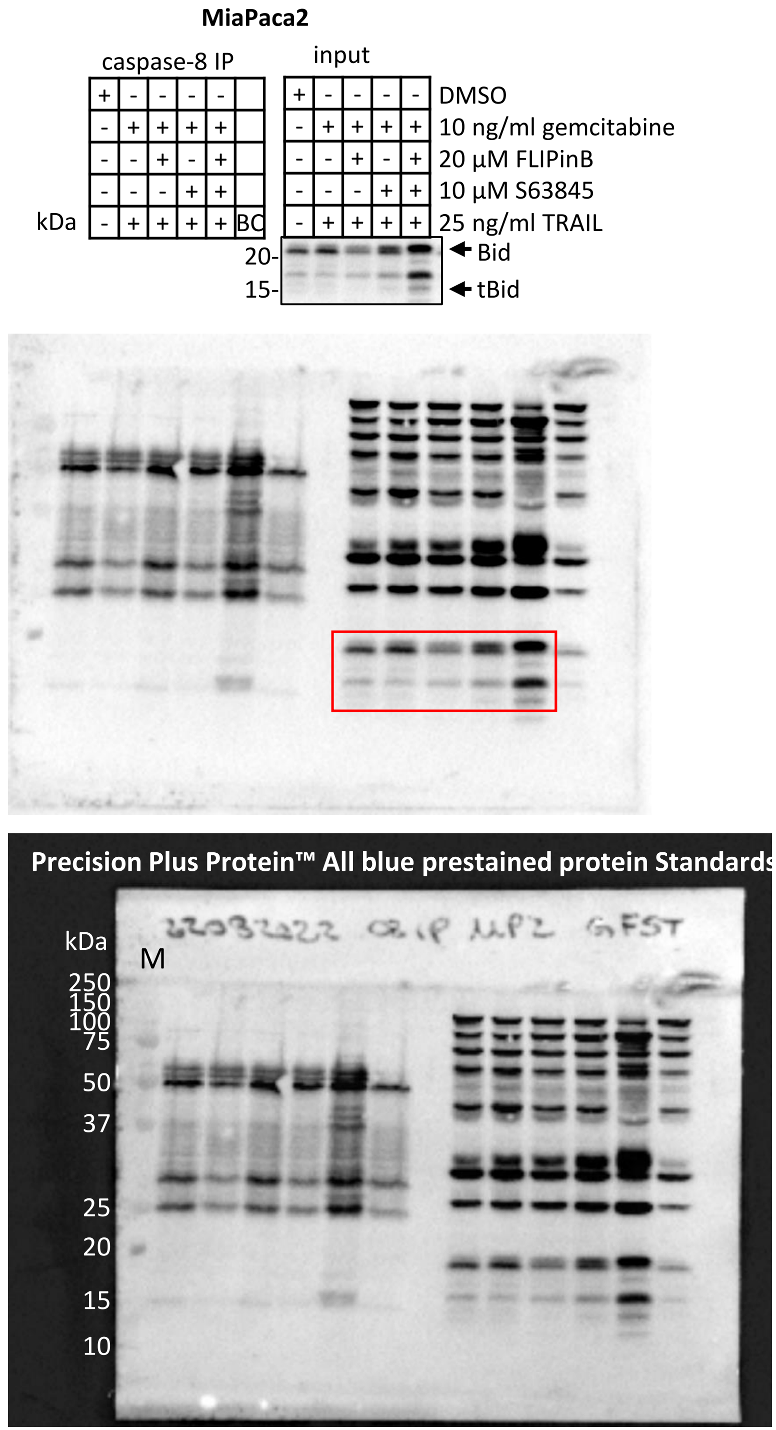

MiaPaca2
caspase-8 IP
input
DMSO
10 ng/ml gemcitabine
20 µM FLIPinB
10 µM S63845
25 ng/ml TRAIL
kDa
BC
20-
15-
Bid
tBid
Precision Plus Protein™ All blue prestained protein Standards
kDa
M
250
150
100
75
50
37
25
20
15
10

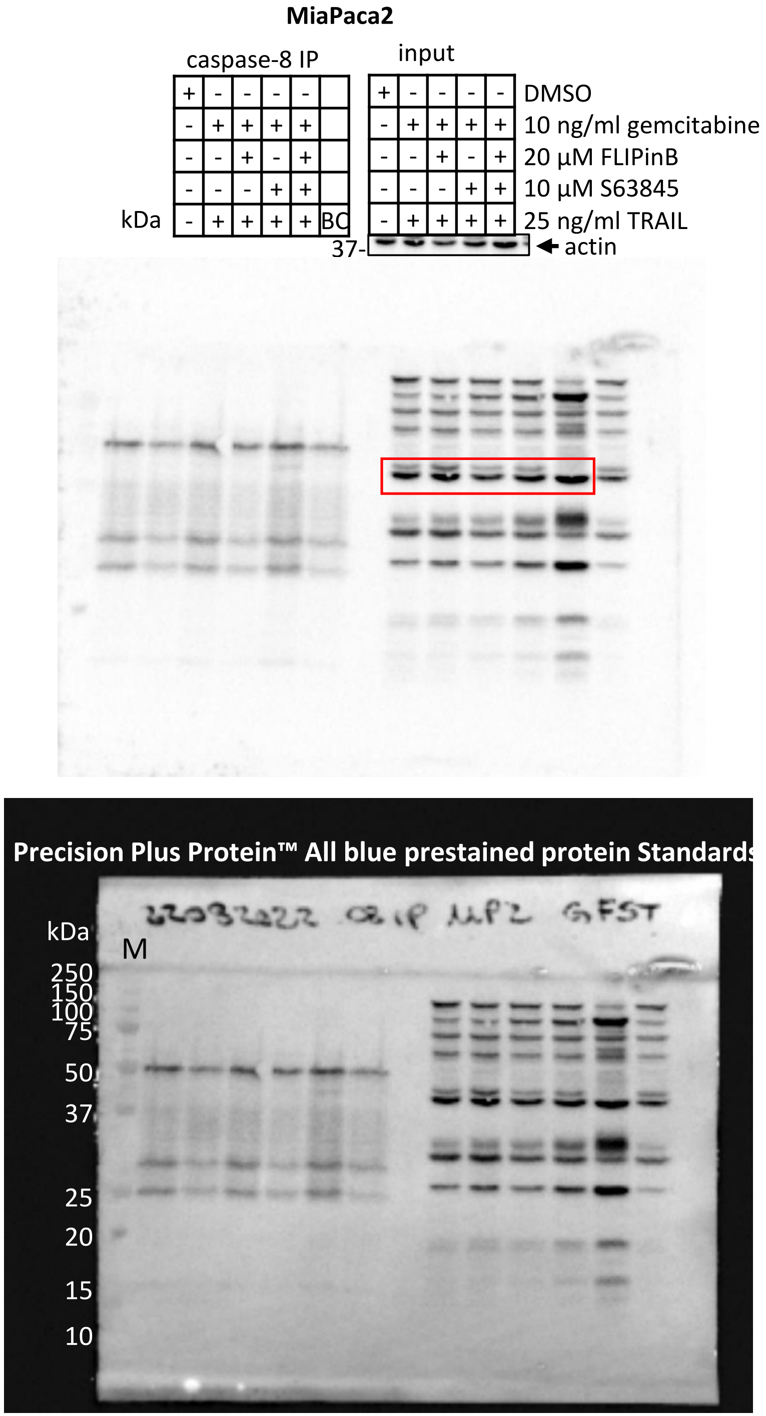

MiaPaca2
caspase-8 IP
input
DMSO
10 ng/ml gemcitabine
20 µM FLIPinB
10 µM S63845
25 ng/ml TRAIL
kDa
BC
37-
actin
Precision Plus Protein™ All blue prestained protein Standards
kDa
M
250
150
100
75
50
37
25
20
15
10

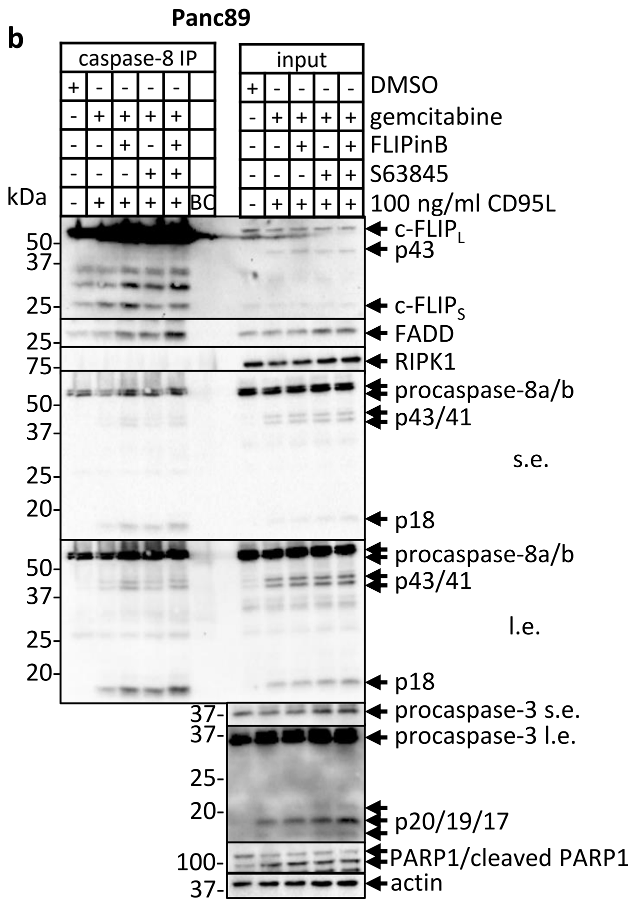
b
Panc89
caspase-8 IP
input
DMSO
gemcitabine
FLIPinB
S63845
100 ng/ml CD95L
kDa
BC
c-FLIPL
p43
c-FLIPS
FADD
RIPK1
procaspase-8a/b
p43/41
s.e.
p18
procaspase-8a/b
p43/41
l.e.
p18
procaspase-3 s.e.
procaspase-3 l.e.
p20/19/17
PARP1/cleaved PARP1
actin

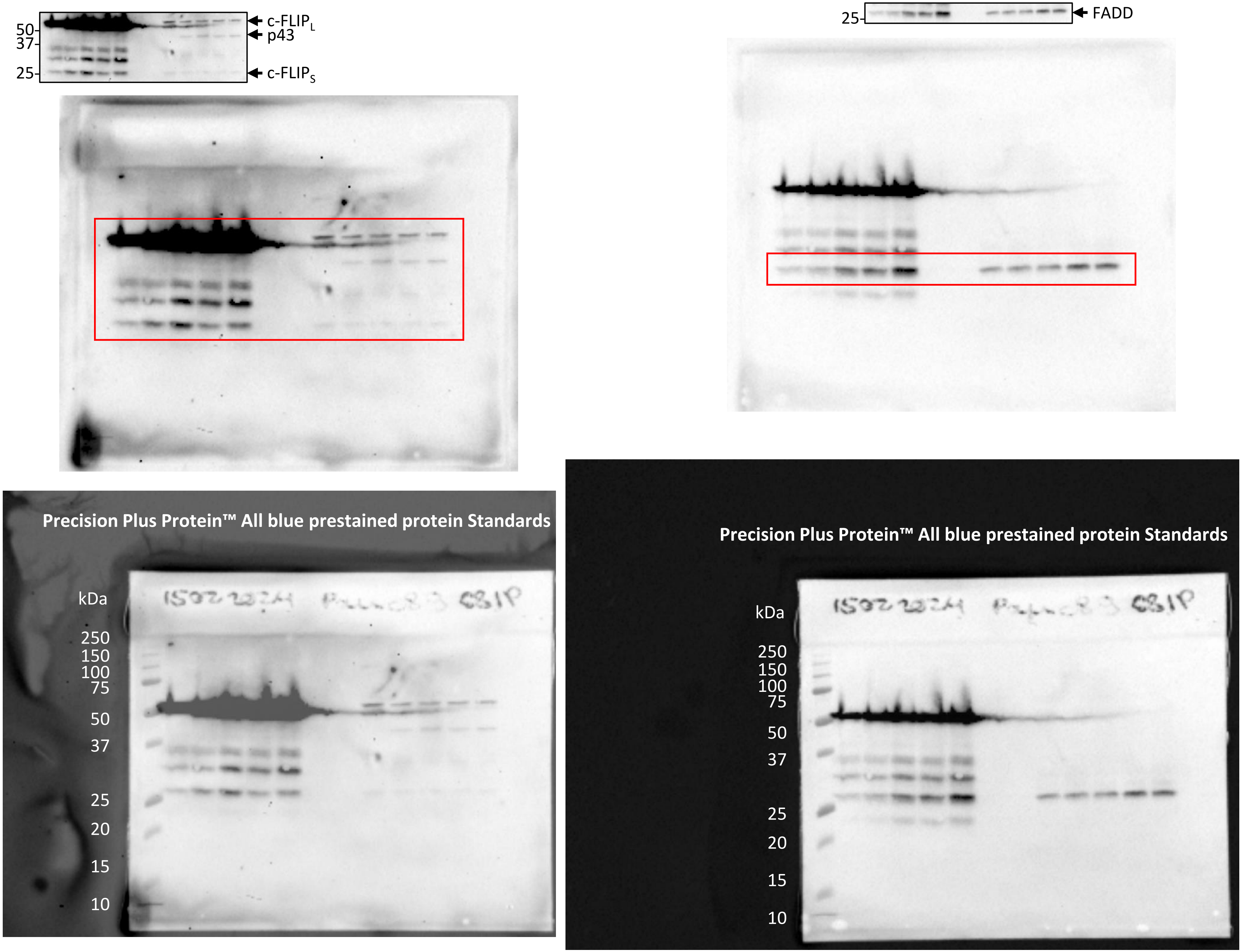
50
37
25
c-FLIP$_L$
p43
c-FLIP$_S$
25
FADD
Precision Plus Protein™ All blue prestained protein Standards
kDa
250
150
100
75
50
37
25
20
15
10
Precision Plus Protein™ All blue prestained protein Standards
kDa
250
150
100
75
50
37
25
20
15
10

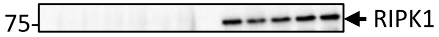
75
RIPK1

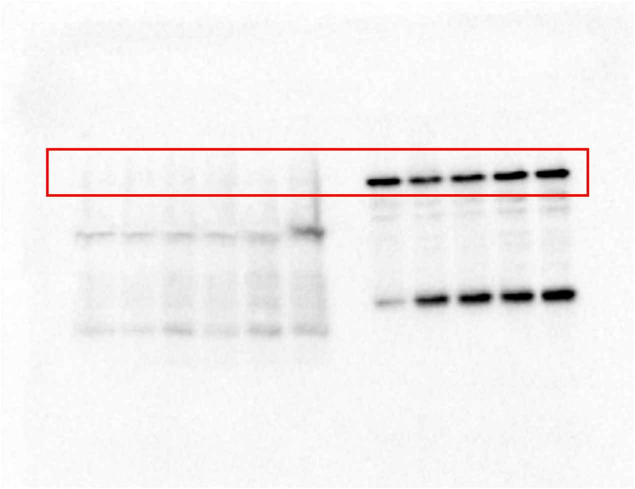

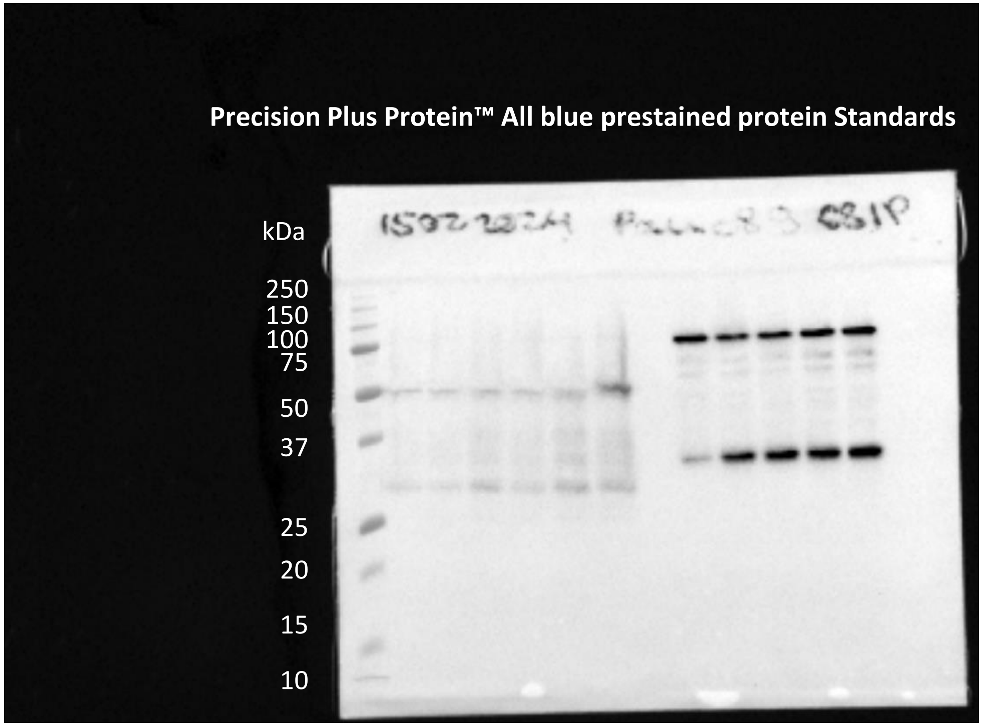
Precision Plus Protein™ All blue prestained protein Standards
kDa
250
150
100
75
50
37
25
20
15
10

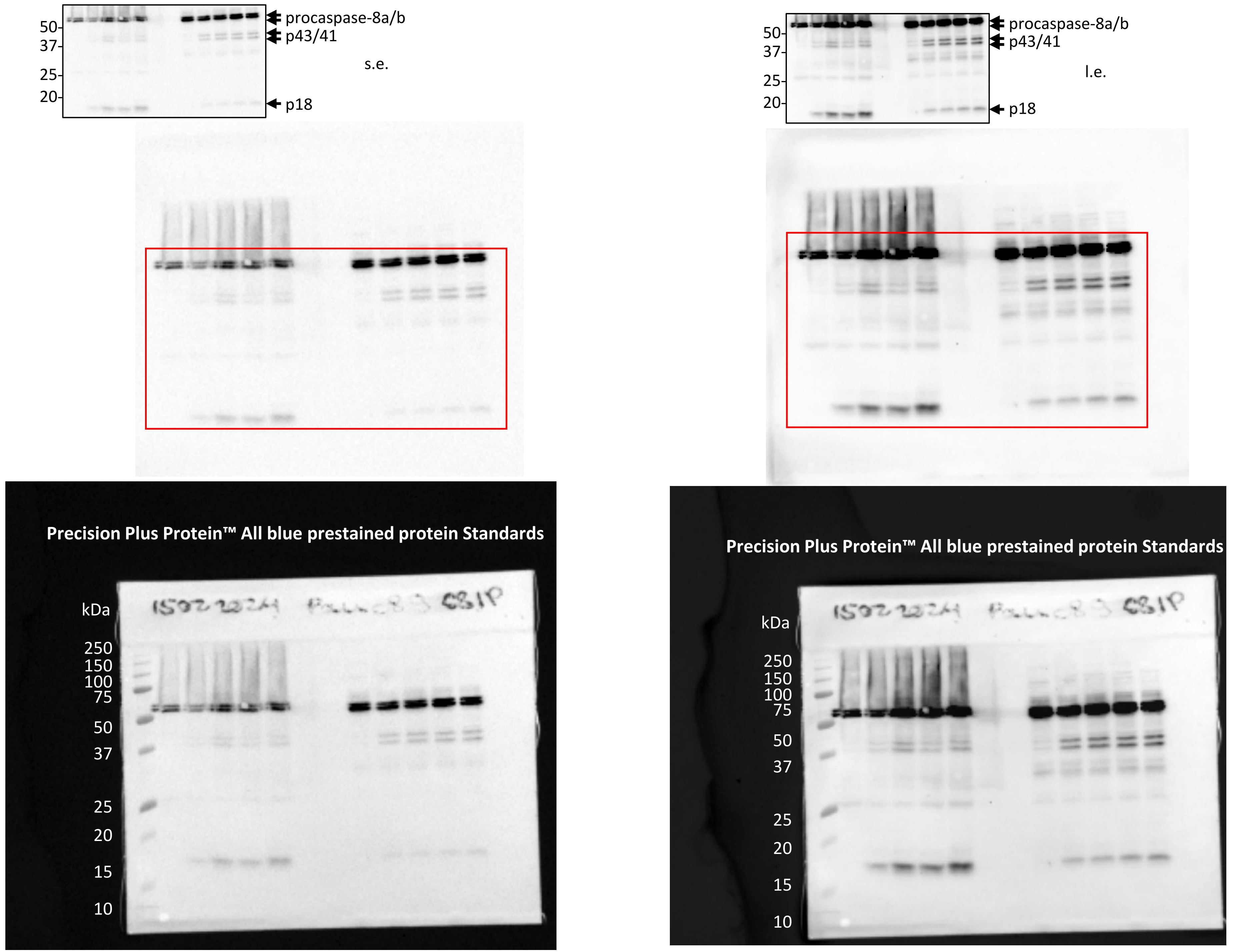

50-
37-
25-
20-
procaspase-8a/b
p43/41
p18
s.e.
l.e.
Precision Plus Protein™ All blue prestained protein Standards
kDa
250
150
100
75
50
37
25
20
15
10

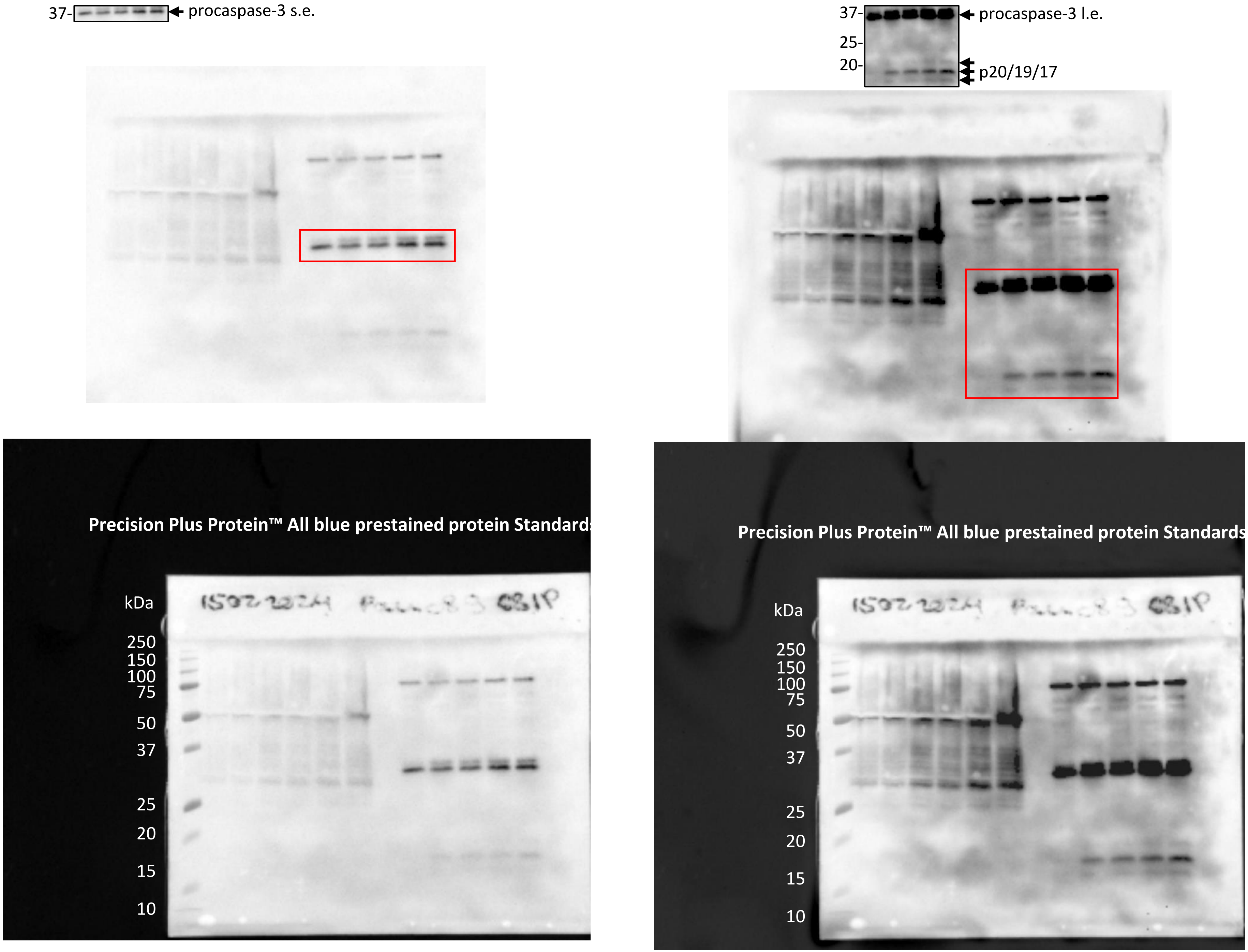
37-
procaspase-3 s.e.
37-
25-
20-
procaspase-3 l.e.
p20/19/17
Precision Plus Protein™ All blue prestained protein Standards
kDa
250
150
100
75
50
37
25
20
15
10
Precision Plus Protein™ All blue prestained protein Standards
kDa
250
150
100
75
50
37
25
20
15
10

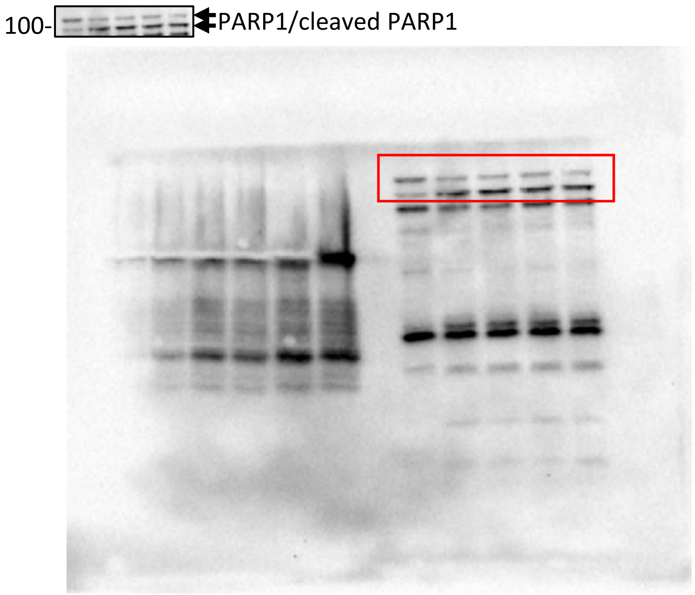
100-
PARP1/cleaved PARP1

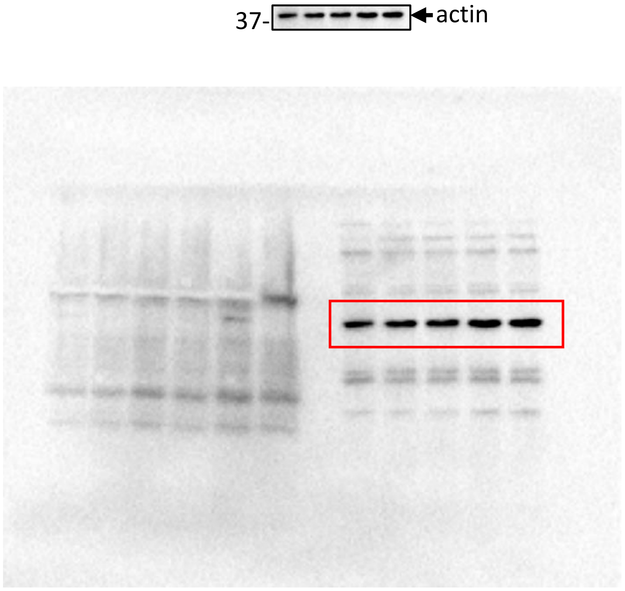
37-
actin

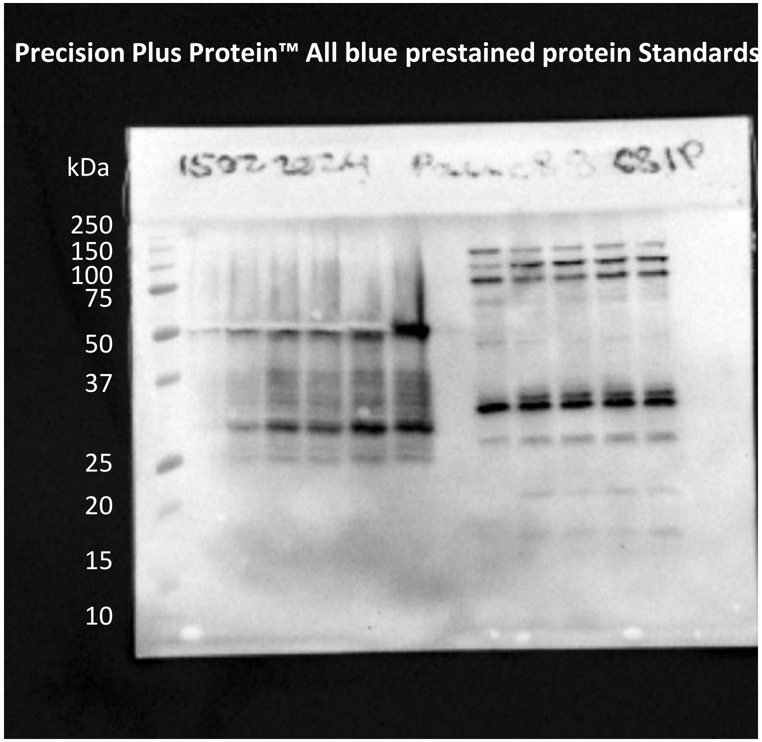
Precision Plus Protein™ All blue prestained protein Standards
kDa
250
150
100
75
50
37
25
20
15
10

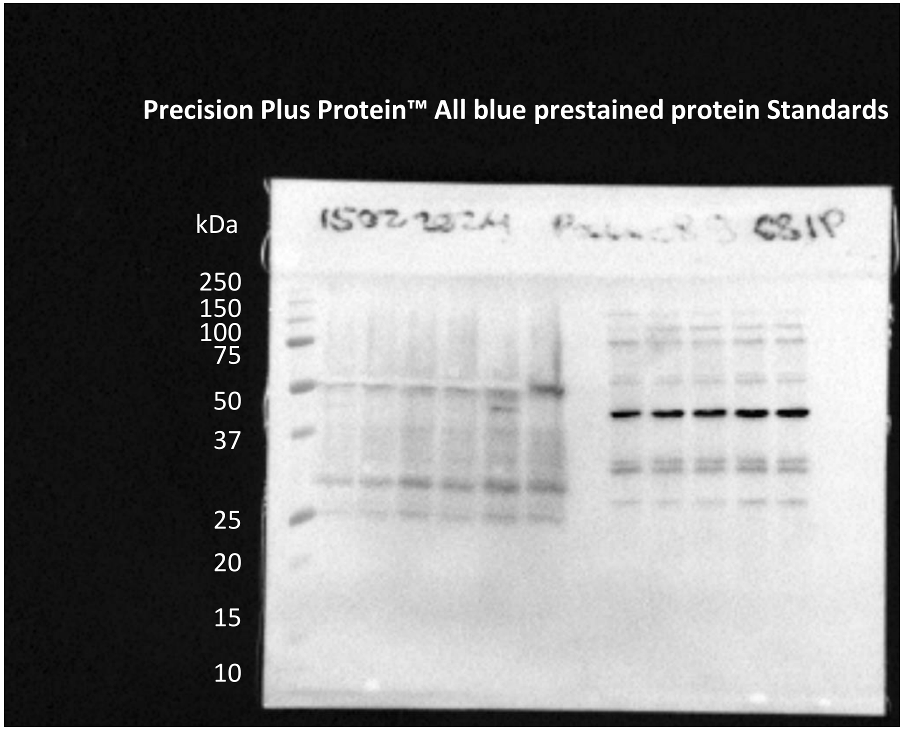
Precision Plus Protein™ All blue prestained protein Standards
kDa
250
150
100
75
50
37
25
20
15
10

c

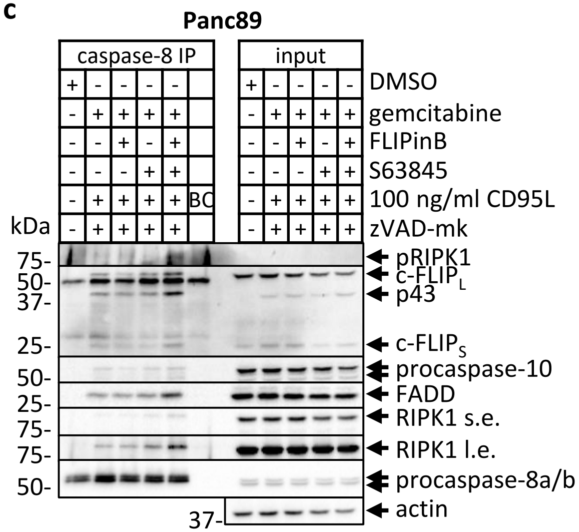

Panc89
caspase-8 IP
input
DMSO
gemcitabine
FLIPinB
S63845
100 ng/ml CD95L
zVAD-mk
BC
kDa
75-
50-
37-
25-
50-
25-
75-
75-
50-
37-
pRIPK1
c-FLIPL
p43
c-FLIPS
procaspase-10
FADD
RIPK1 s.e.
RIPK1 l.e.
procaspase-8a/b
actin

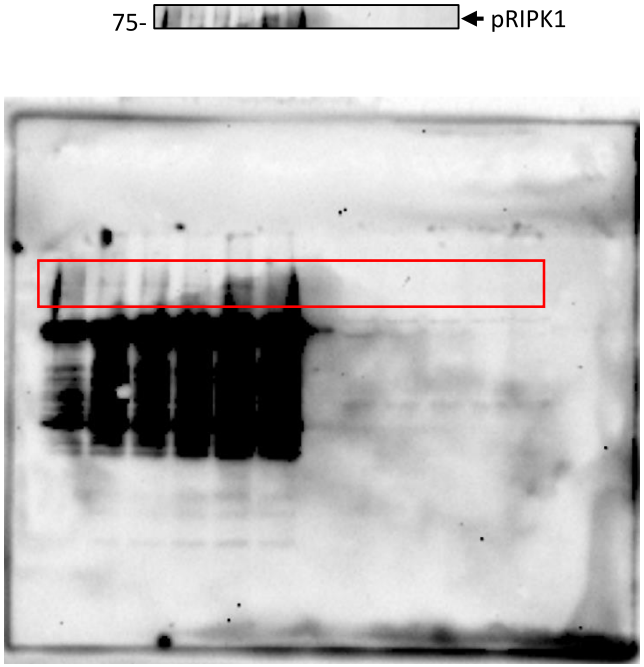
75-
pRIPK1

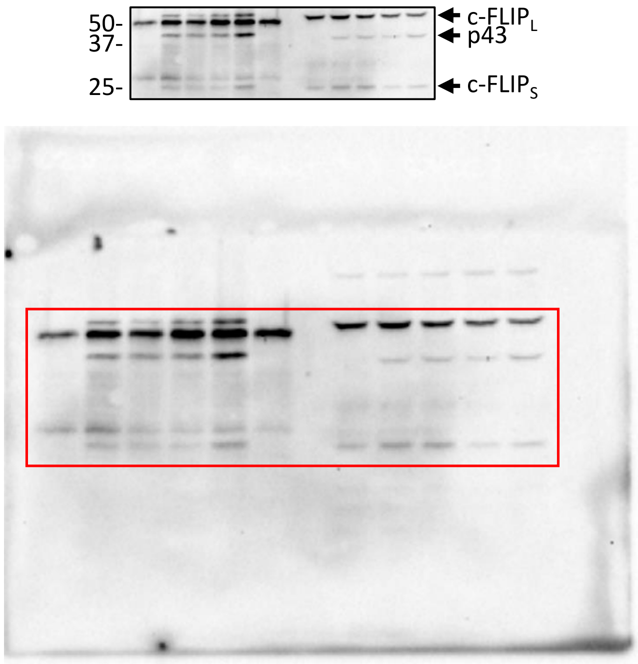
50-
37-
25-
c-FLIP$_L$
p43
c-FLIP$_S$

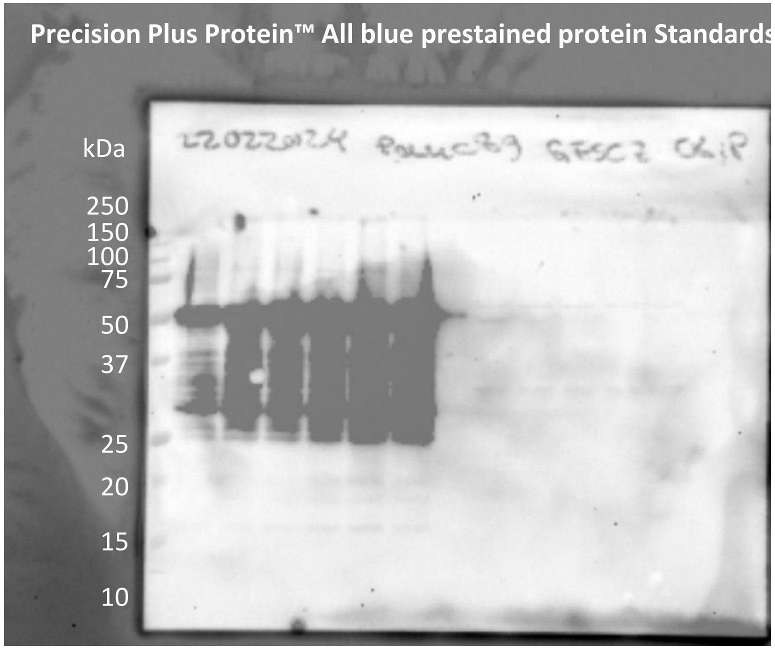
Precision Plus Protein™ All blue prestained protein Standards
kDa
250
150
100
75
50
37
25
20
15
10

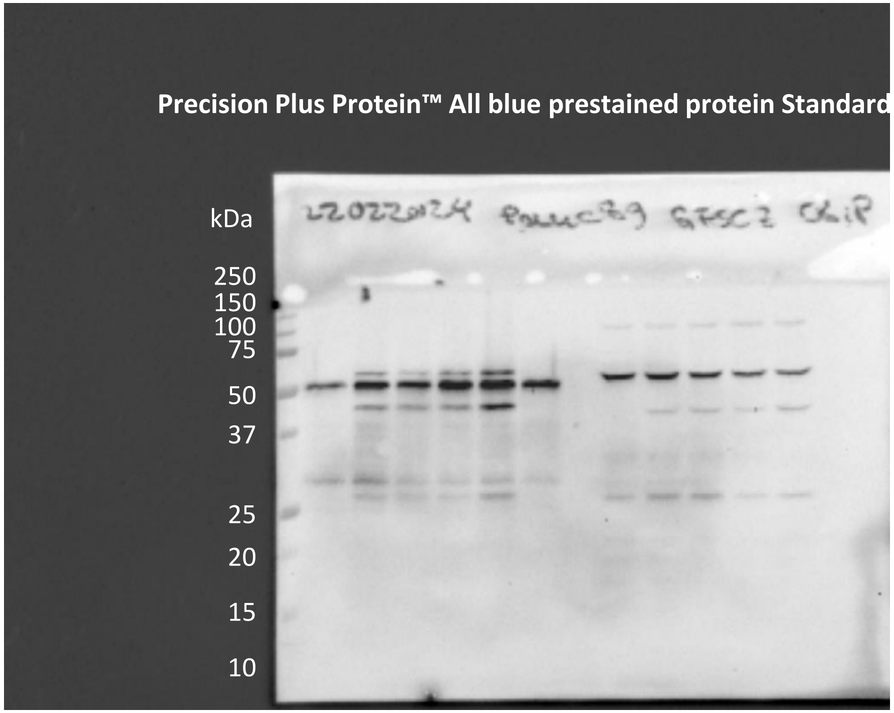
Precision Plus Protein™ All blue prestained protein Standard
kDa
250
150
100
75
50
37
25
20
15
10

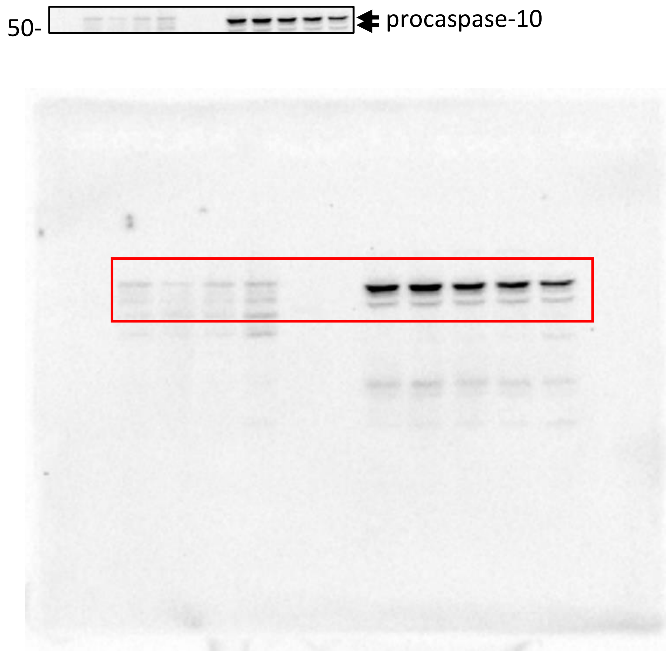
50-
procaspase-10

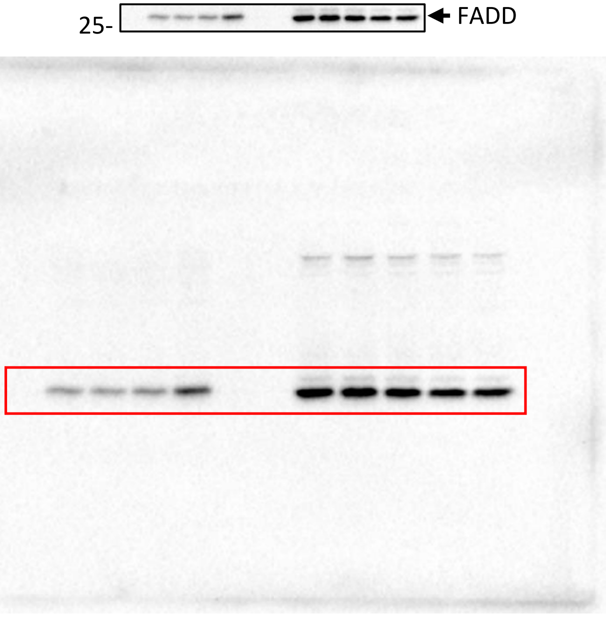
25-
FADD

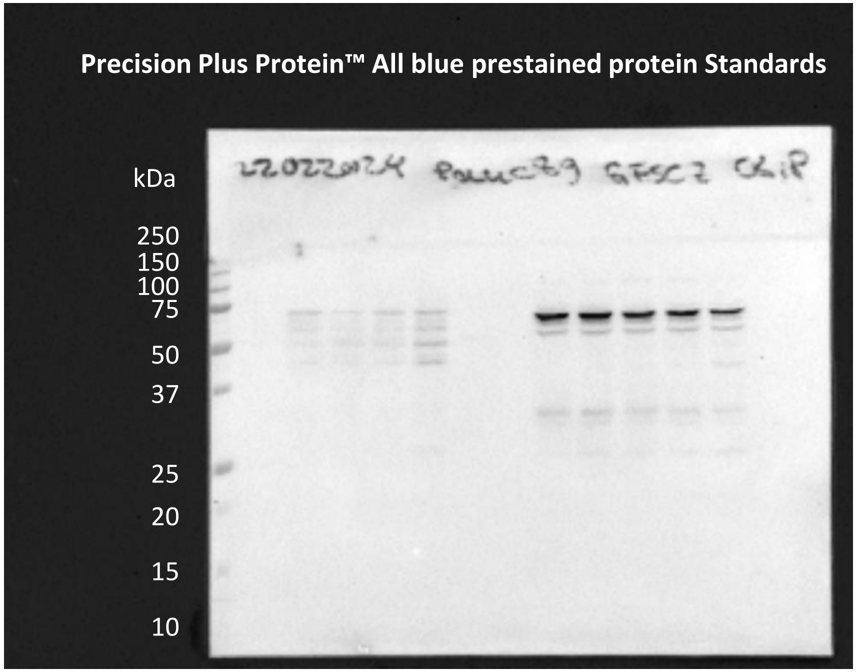
Precision Plus Protein™ All blue prestained protein Standards
kDa
250
150
100
75
50
37
25
20
15
10

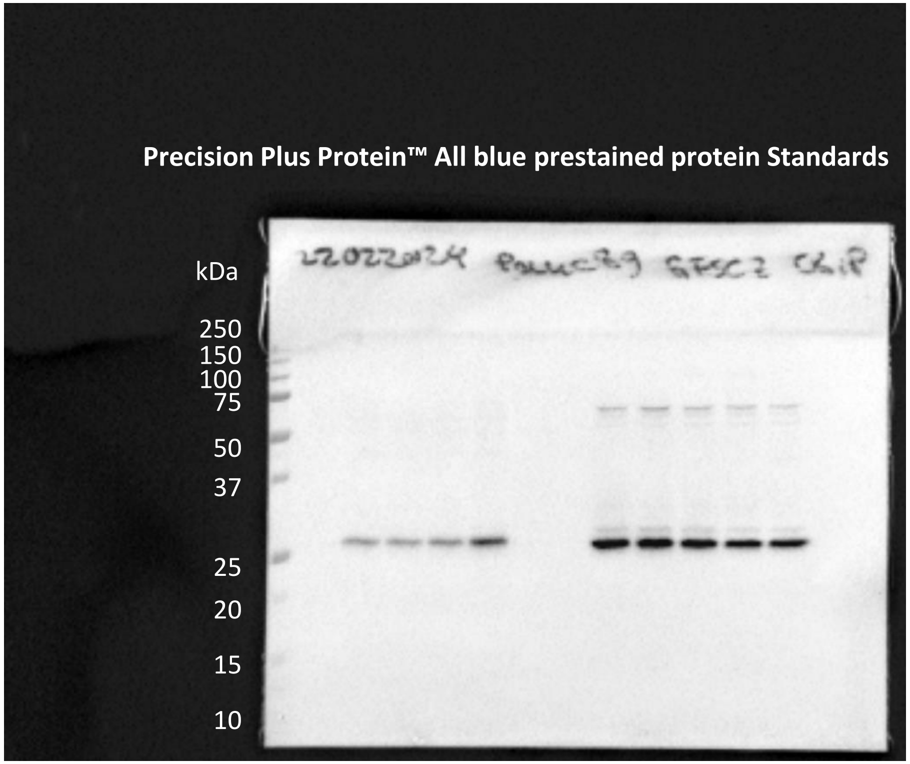
Precision Plus Protein™ All blue prestained protein Standards
kDa
250
150
100
75
50
37
25
20
15
10

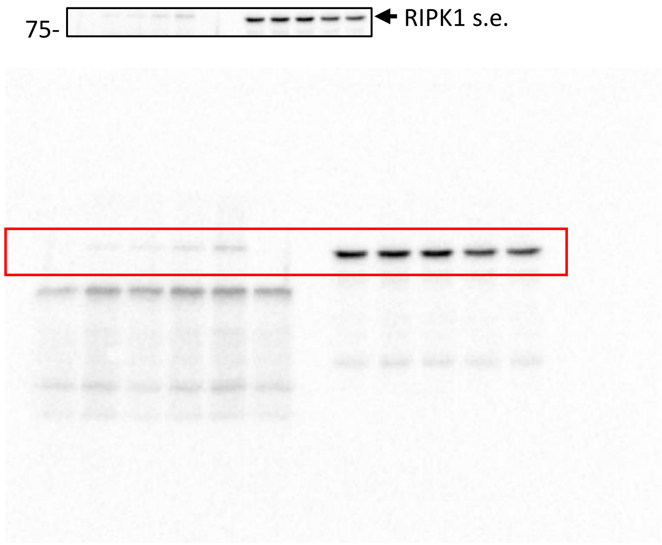
75-
RIPK1 s.e.

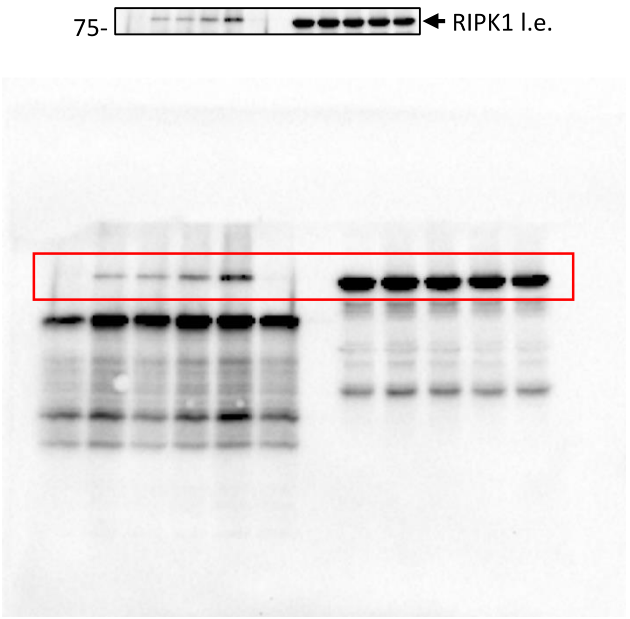
75-
RIPK1 l.e.

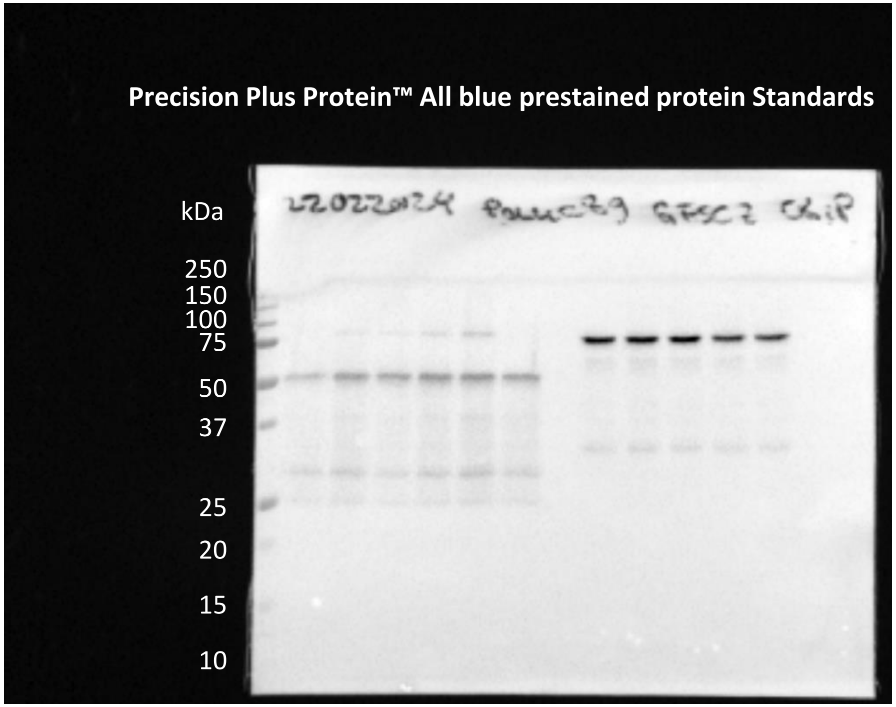
Precision Plus Protein™ All blue prestained protein Standards
kDa
250
150
100
75
50
37
25
20
15
10

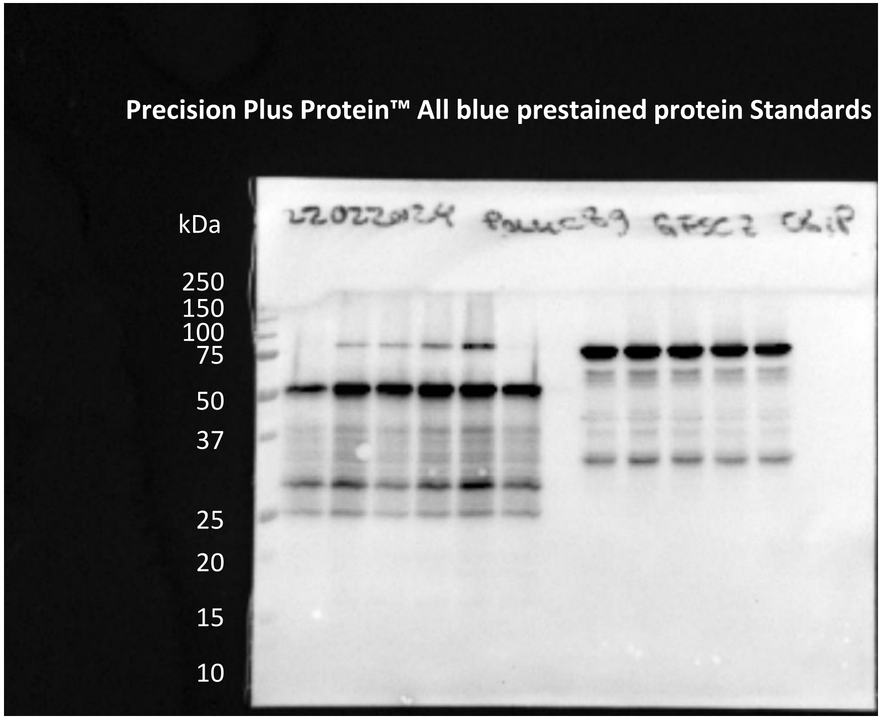
Precision Plus Protein™ All blue prestained protein Standards
kDa
250
150
100
75
50
37
25
20
15
10

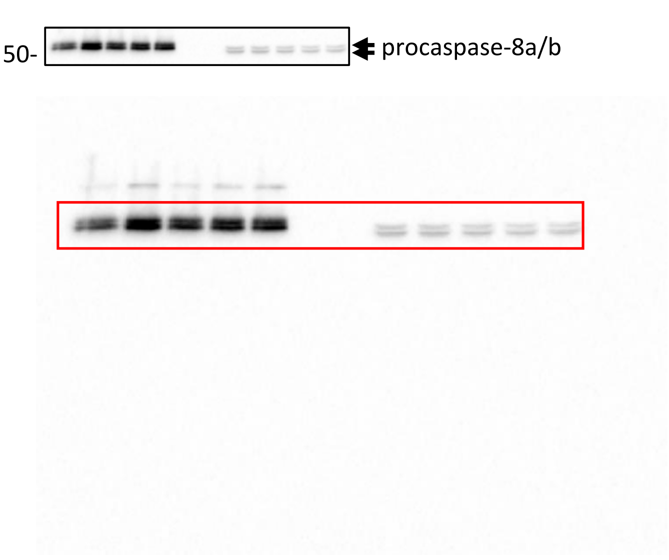
50-
procaspase-8a/b

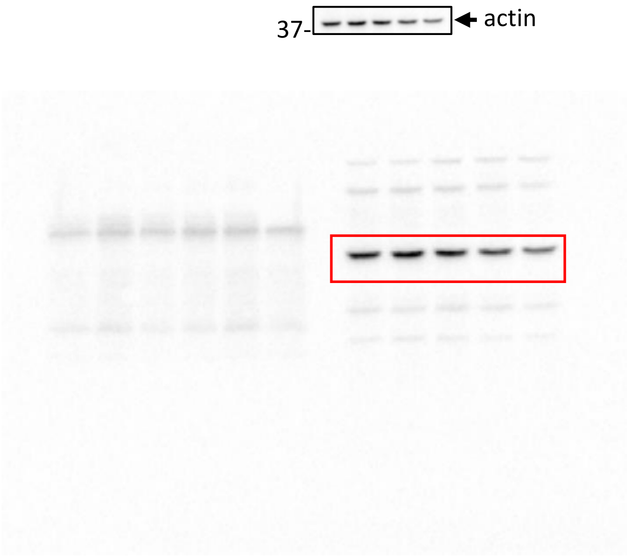
37-
actin

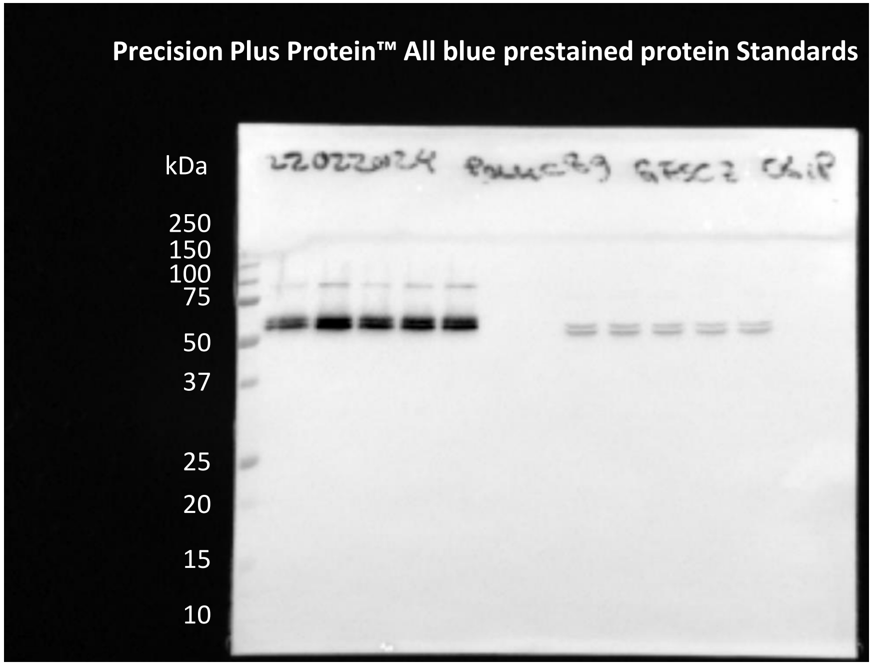
Precision Plus Protein™ All blue prestained protein Standards
kDa
250
150
100
75
50
37
25
20
15
10

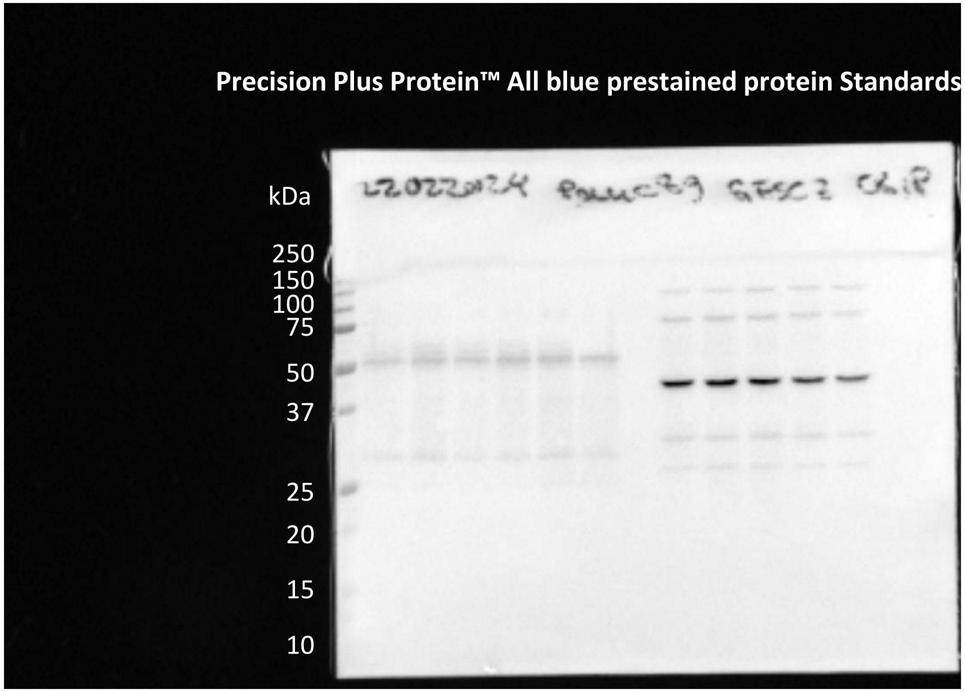
Precision Plus Protein™ All blue prestained protein Standards
kDa
250
150
100
75
50
37
25
20
15
10

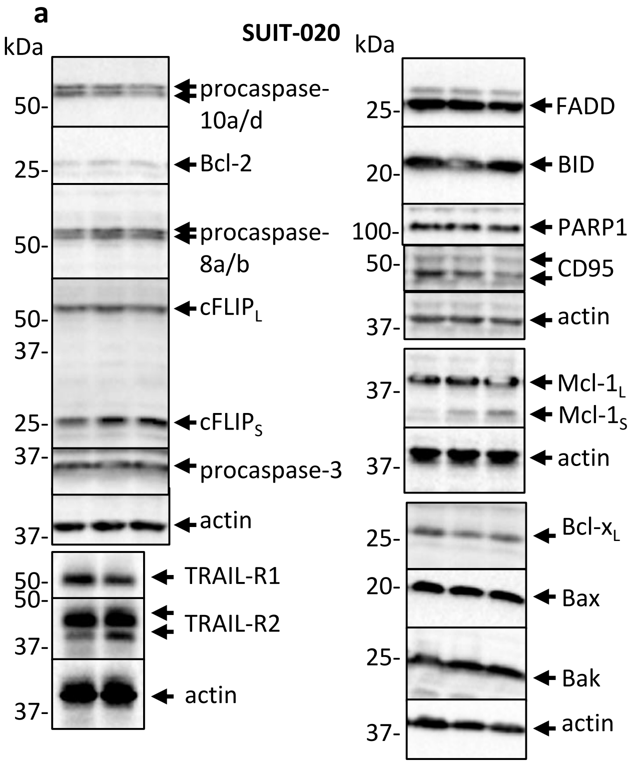

a
SUIT-020
kDa
50-
procaspase-10a/d
25-
Bcl-2
50-
procaspase-8a/b
50-
cFLIP$_L$
37-
25-
cFLIP$_S$
37-
procaspase-3
37-
actin
50-
TRAIL-R1
50-
TRAIL-R2
37-
37-
actin
kDa
25-
FADD
20-
BID
100-
PARP1
50-
CD95
37-
actin
37-
Mcl-1$_L$
Mcl-1$_S$
37-
actin
25-
Bcl-x$_L$
20-
Bax
25-
Bak
37-
actin

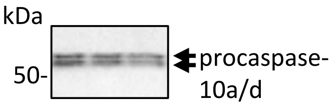
kDa
50-
procaspase-
10a/d

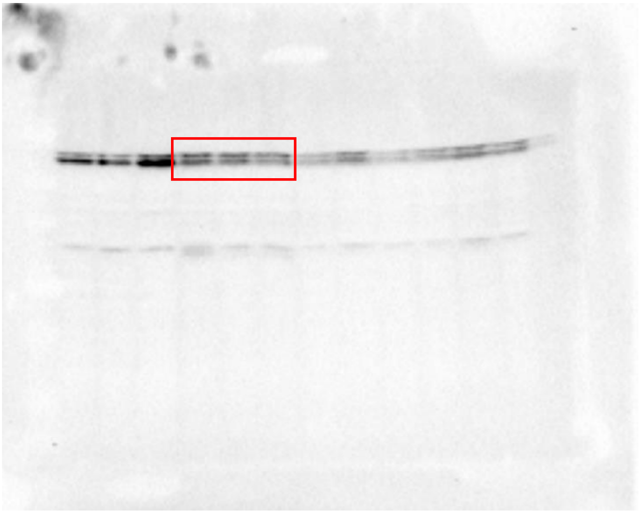

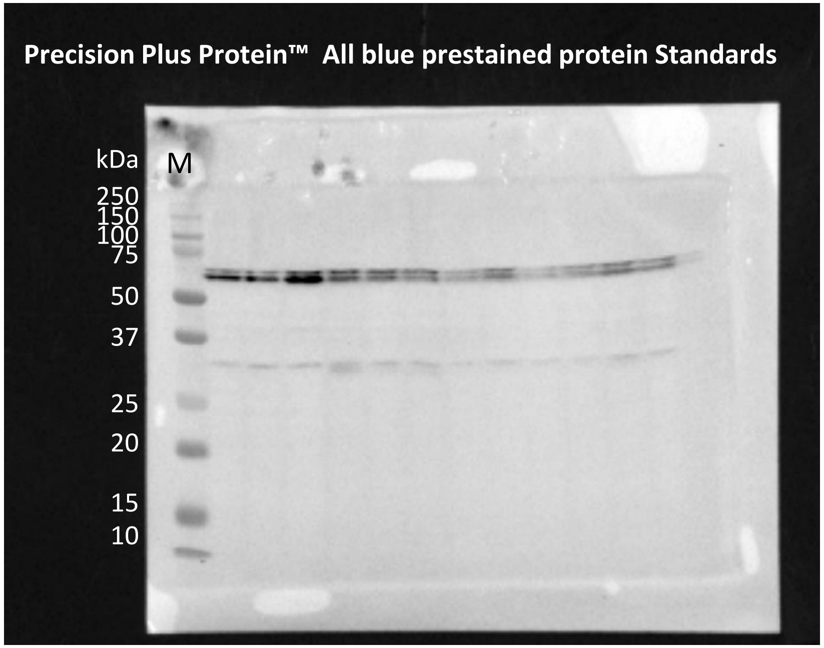
Precision Plus Protein™ All blue prestained protein Standards
kDa
M
250
150
100
75
50
37
25
20
15
10

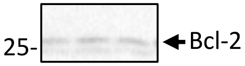
25-
Bcl-2

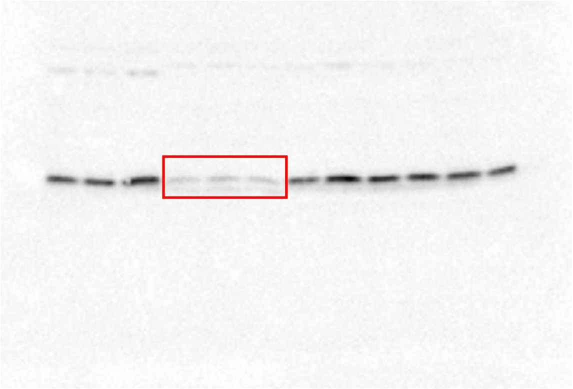

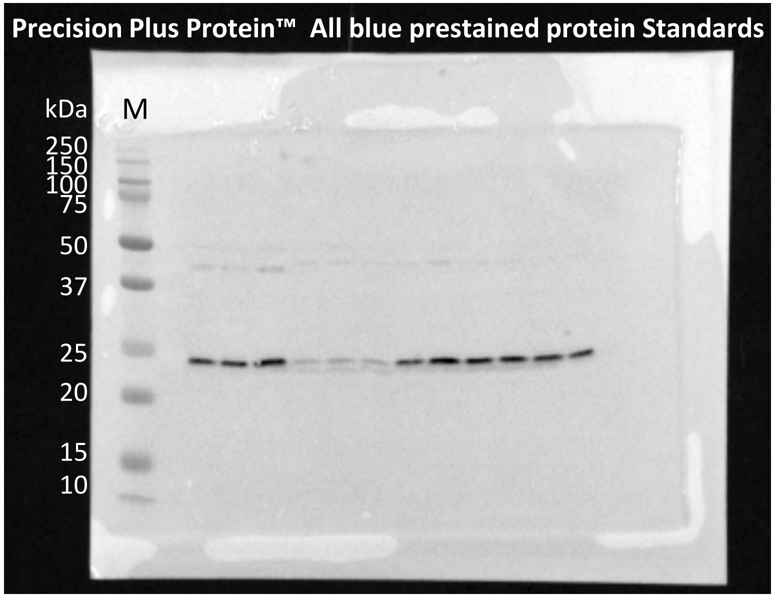
Precision Plus Protein™ All blue prestained protein Standards
kDa
M
250
150
100
75
50
37
25
20
15
10

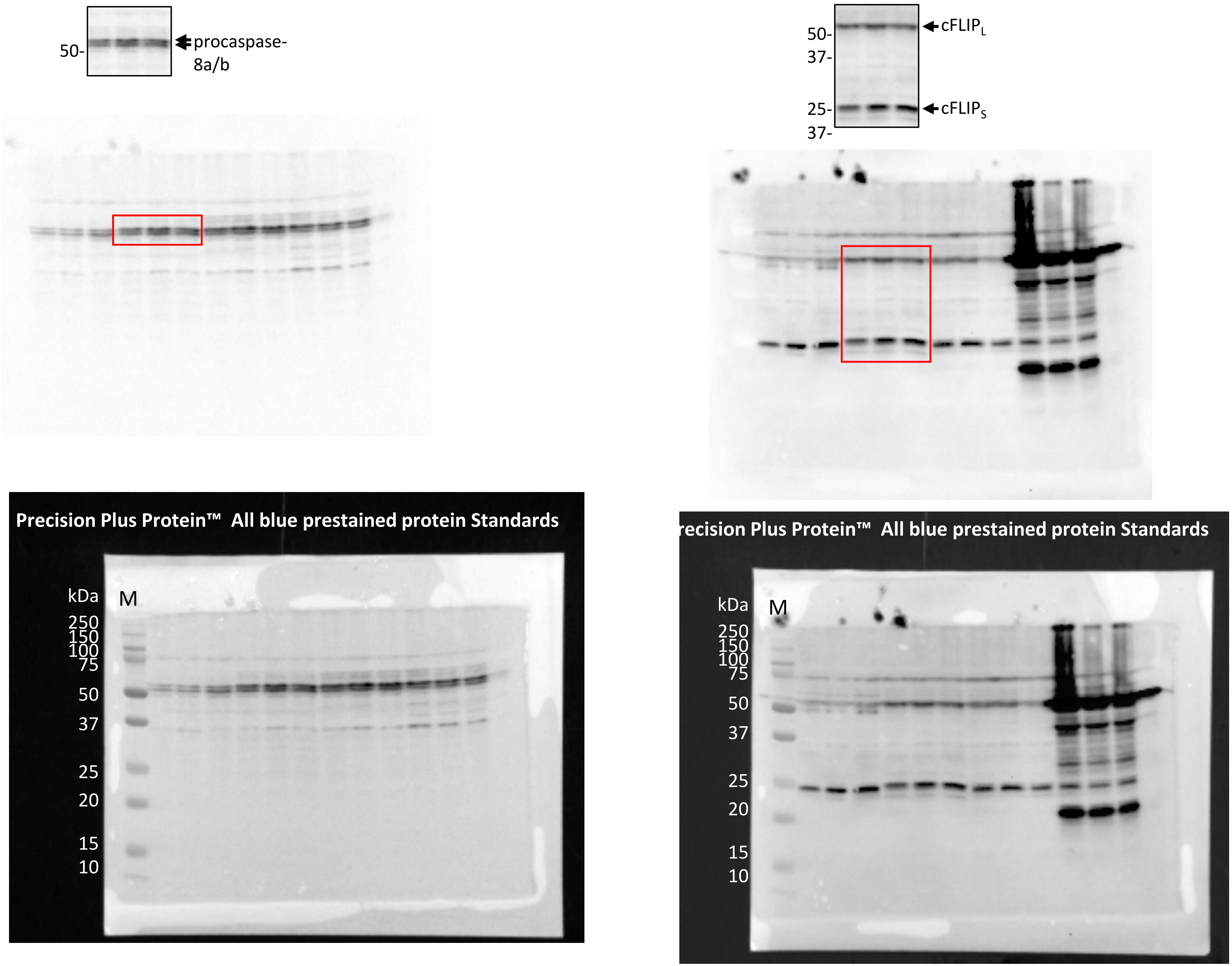

50-
procaspase-8a/b
50-
37-
25-
37-
cFLIP$_L$
cFLIP$_S$
Precision Plus Protein™ All blue prestained protein Standards
kDa
M
250
150
100
75
50
37
25
20
15
10
recision Plus Protein™ All blue prestained protein Standards
kDa
M
250
150
100
75
50
37
25
20
15
10

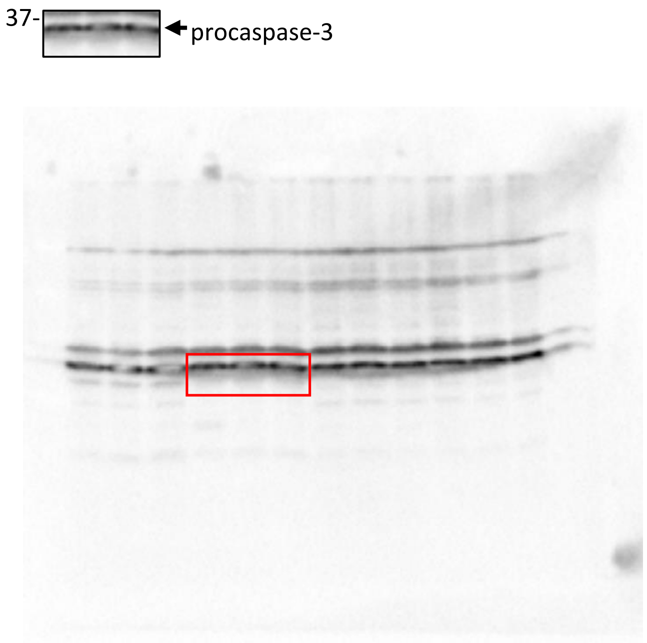
37-
procaspase-3

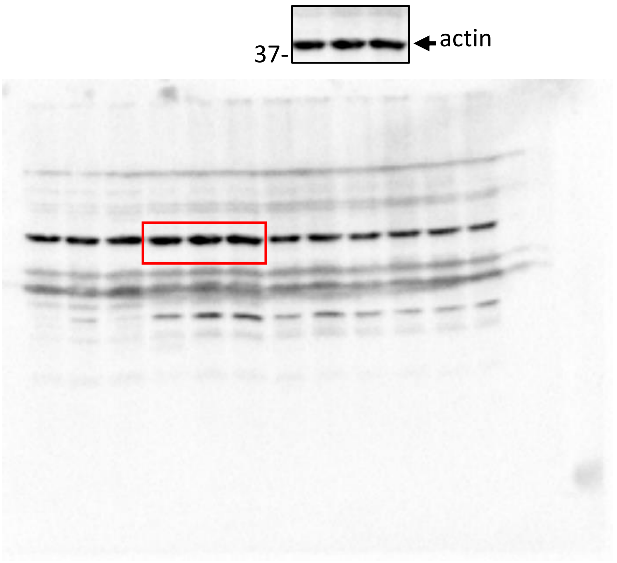
37-
actin

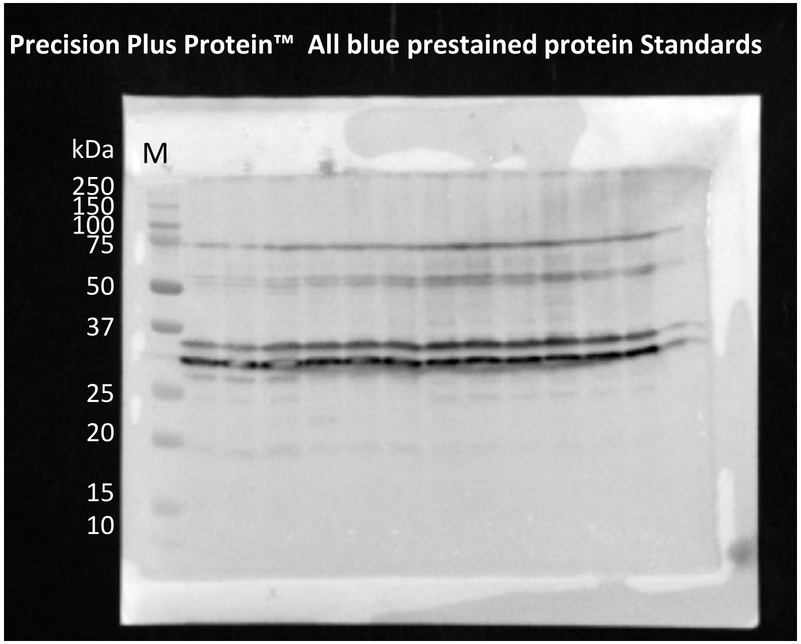
Precision Plus Protein™ All blue prestained protein Standards
kDa
M
250
150
100
75
50
37
25
20
15
10

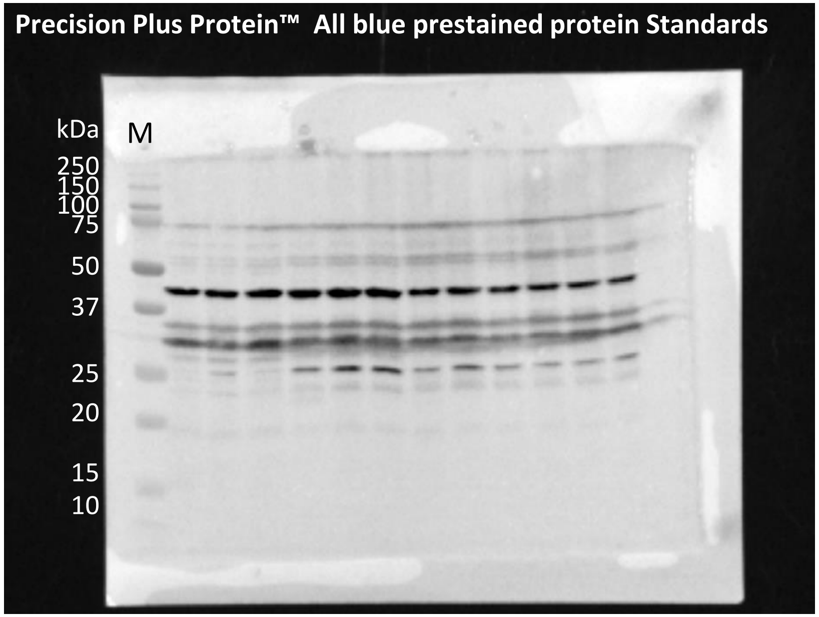
Precision Plus Protein™ All blue prestained protein Standards
kDa
M
250
150
100
75
50
37
25
20
15
10

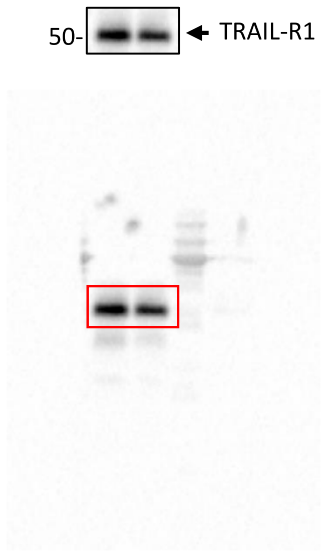

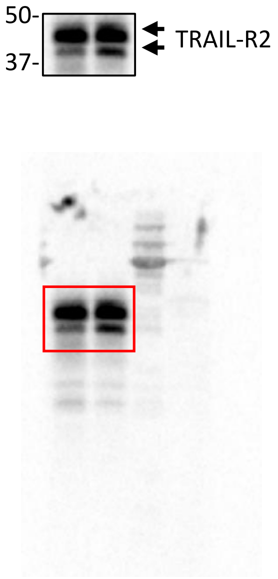

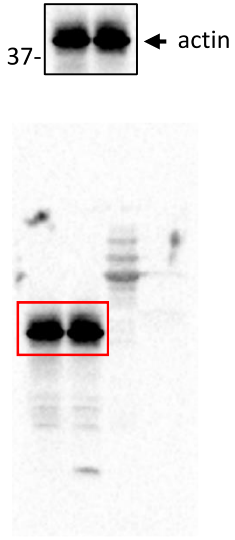

Precision Plus Protein™
All blue prestained protein Standards

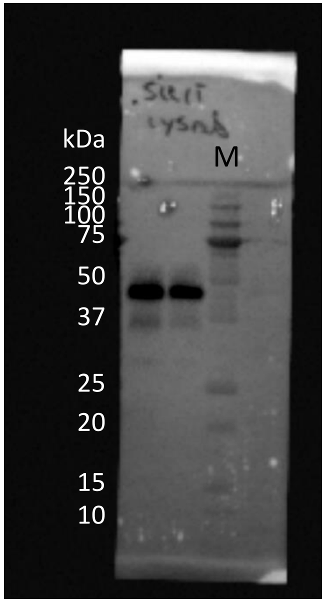

Precision Plus Protein™
All blue prestained protein Standards

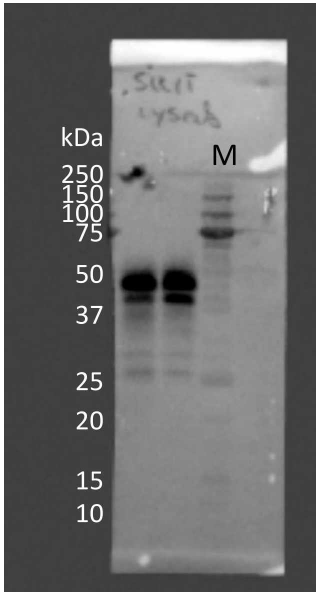

Precision Plus Protein™
All blue prestained protein Standards

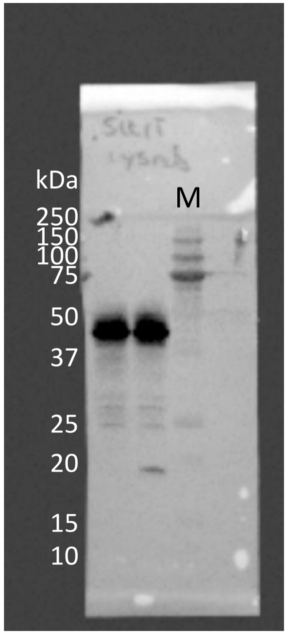

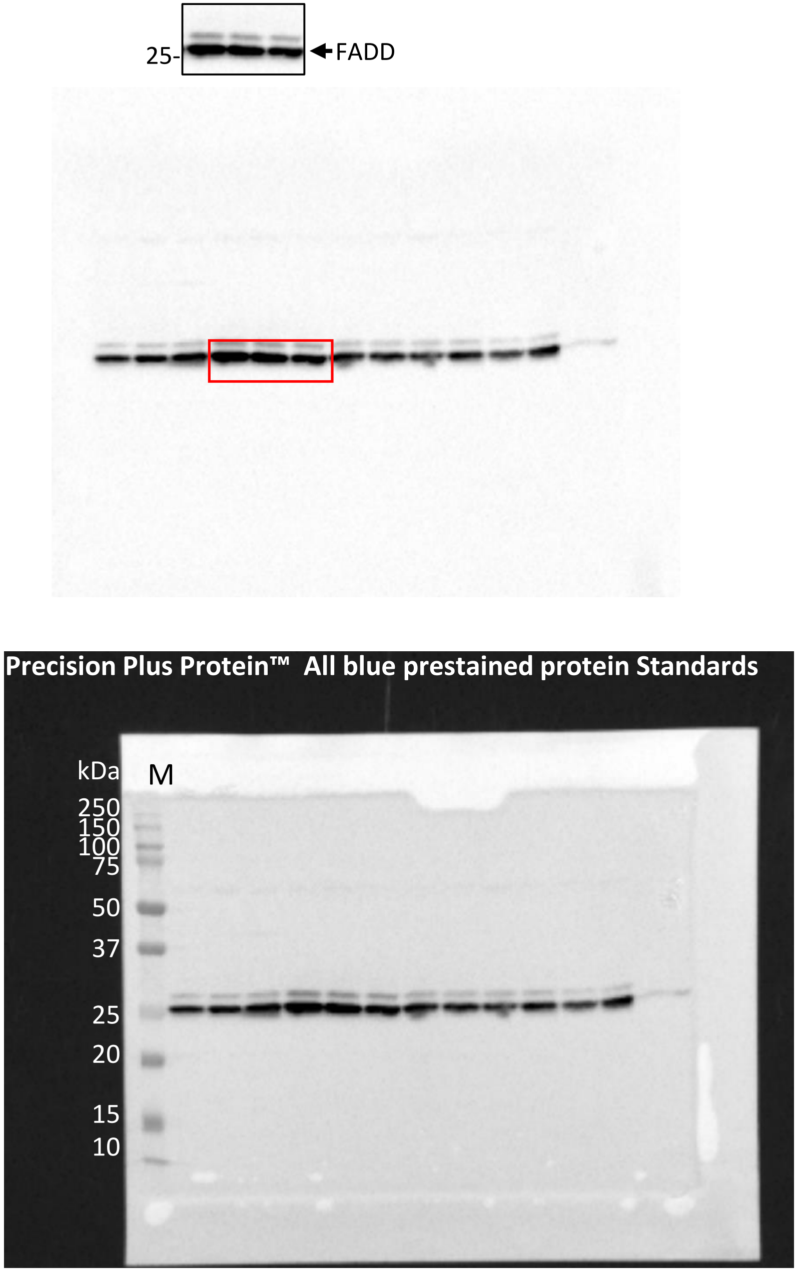
25-
FADD
Precision Plus Protein™ All blue prestained protein Standards
kDa
M
250
150
100
75
50
37
25
20
15
10

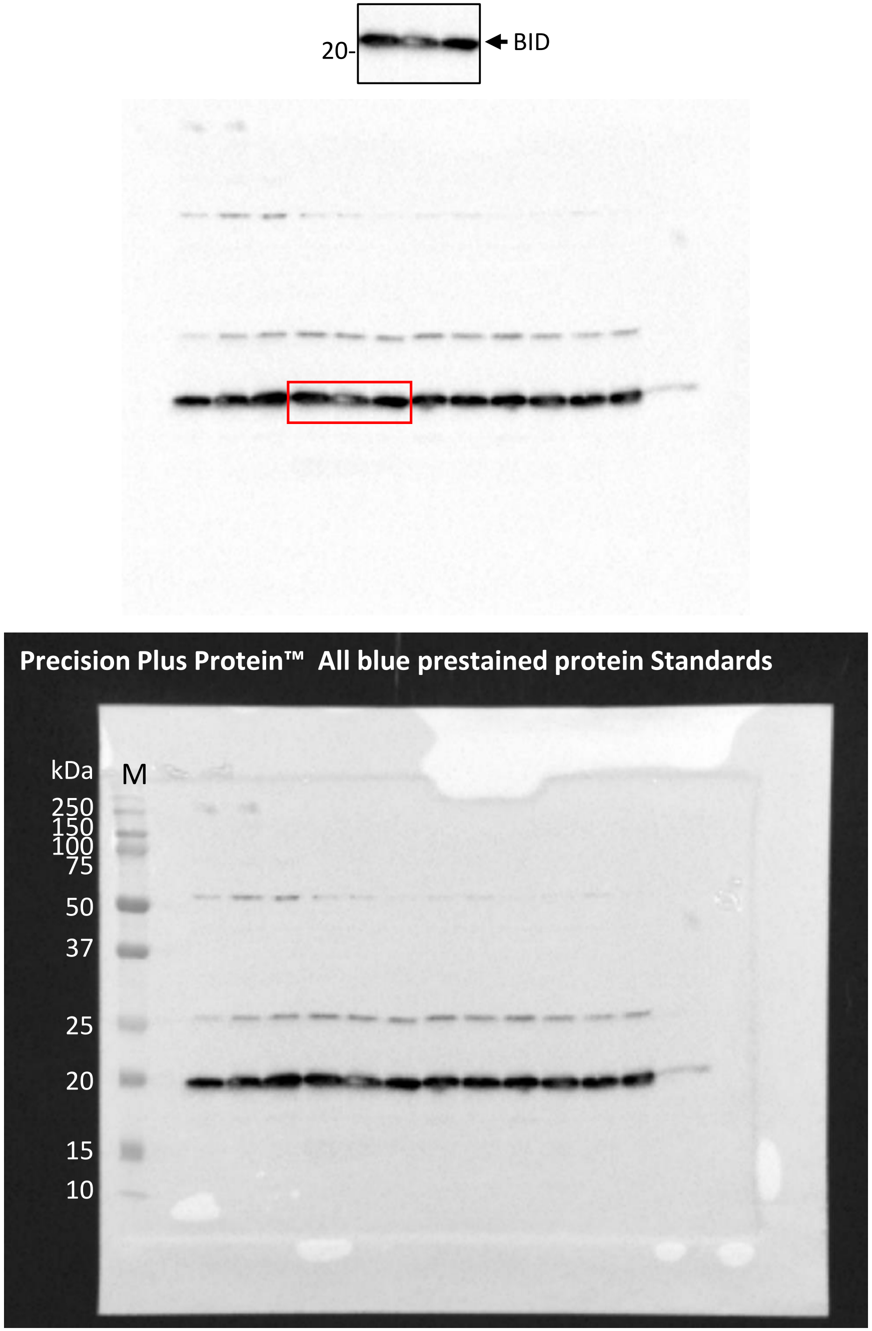
20-
BID
Precision Plus Protein™ All blue prestained protein Standards
kDa
M
250
150
100
75
50
37
25
20
15
10

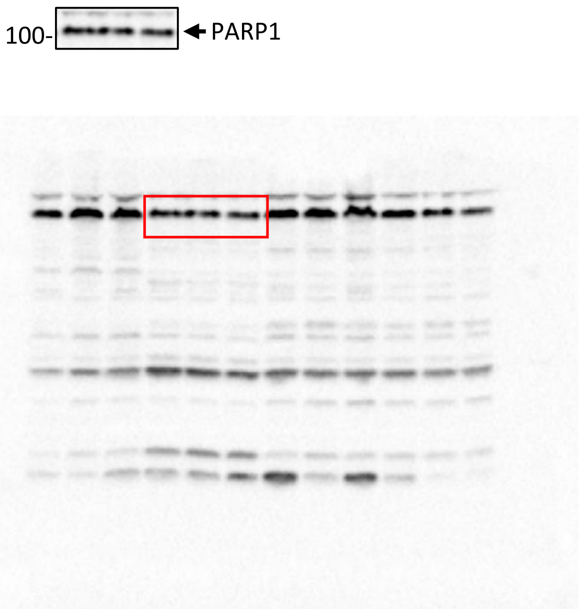
100-
PARP1

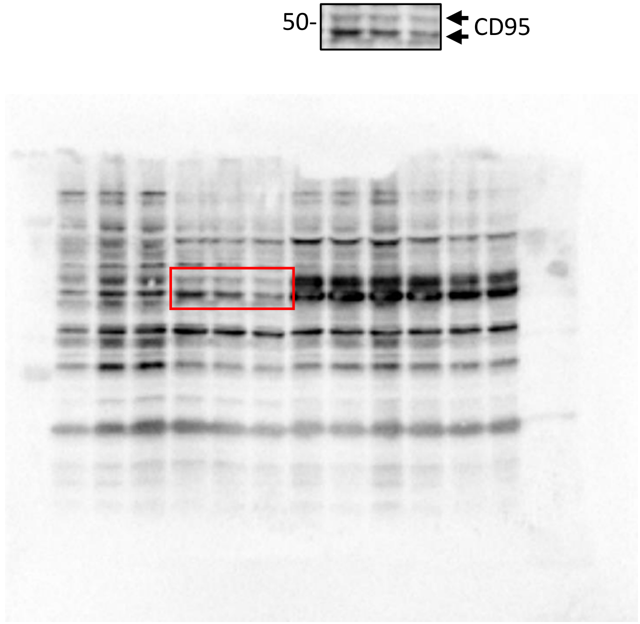
50-
CD95

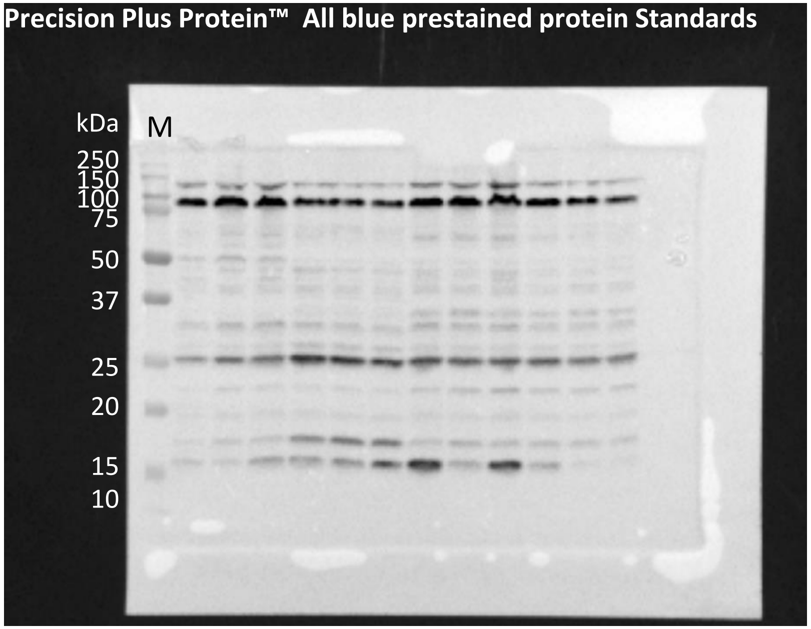
Precision Plus Protein™ All blue prestained protein Standards
kDa
M
250
150
100
75
50
37
25
20
15
10

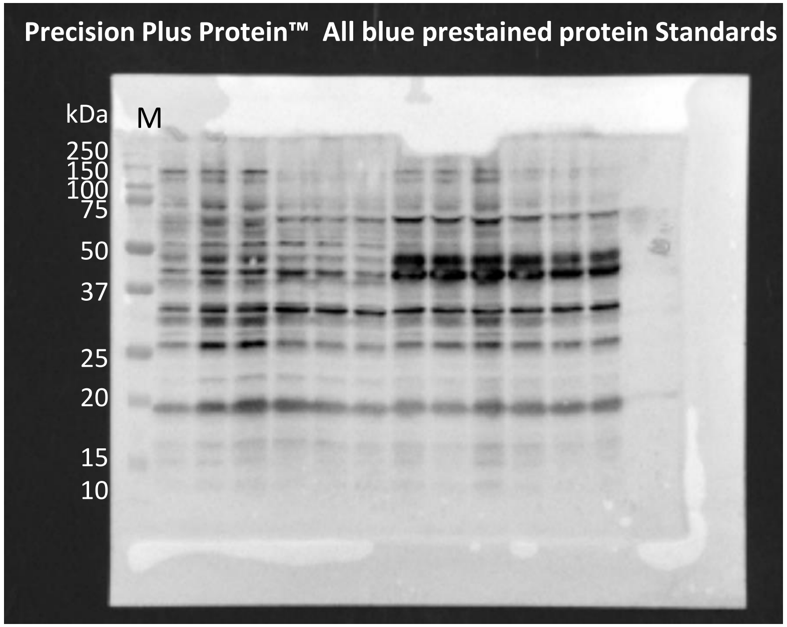
Precision Plus Protein™ All blue prestained protein Standards
kDa
M
250
150
100
75
50
37
25
20
15
10

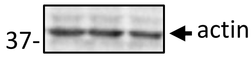
37-
actin

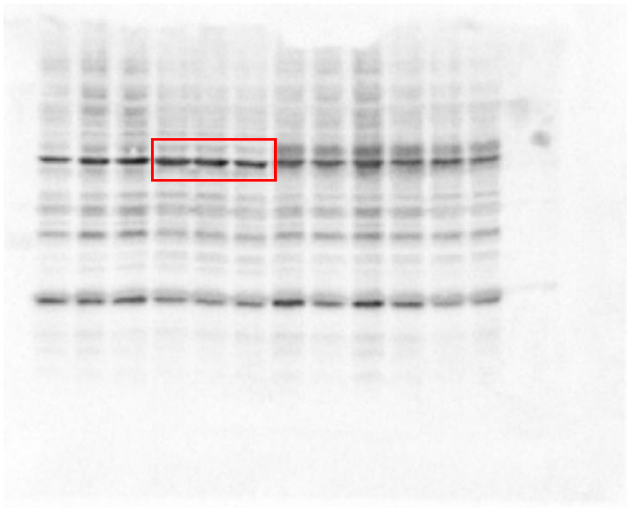

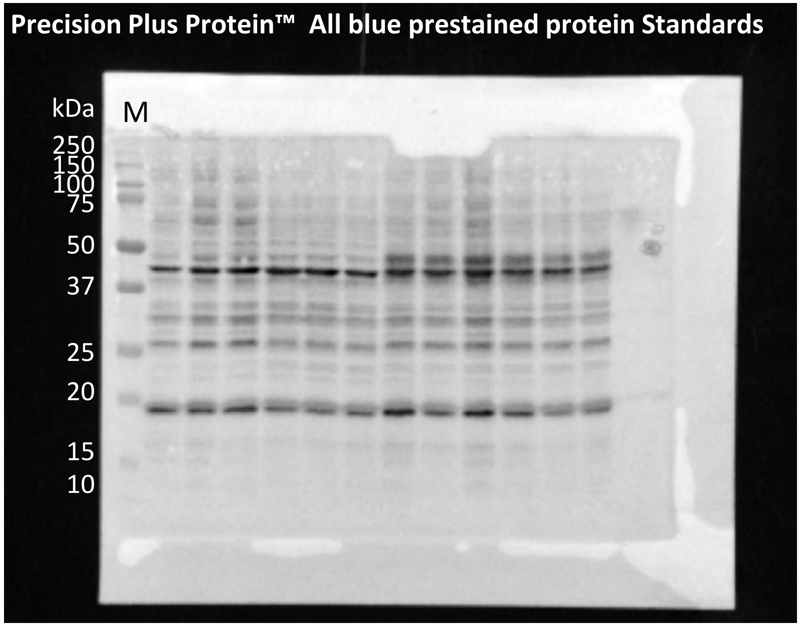
Precision Plus Protein™ All blue prestained protein Standards
kDa
M
250
150
100
75
50
37
25
20
15
10

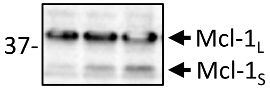
37-
Mcl-1L
Mcl-1S

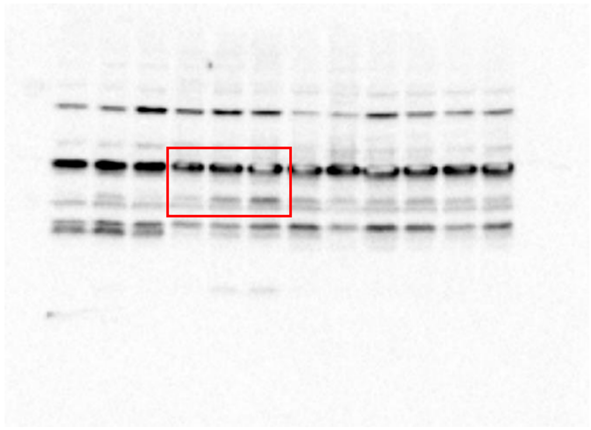

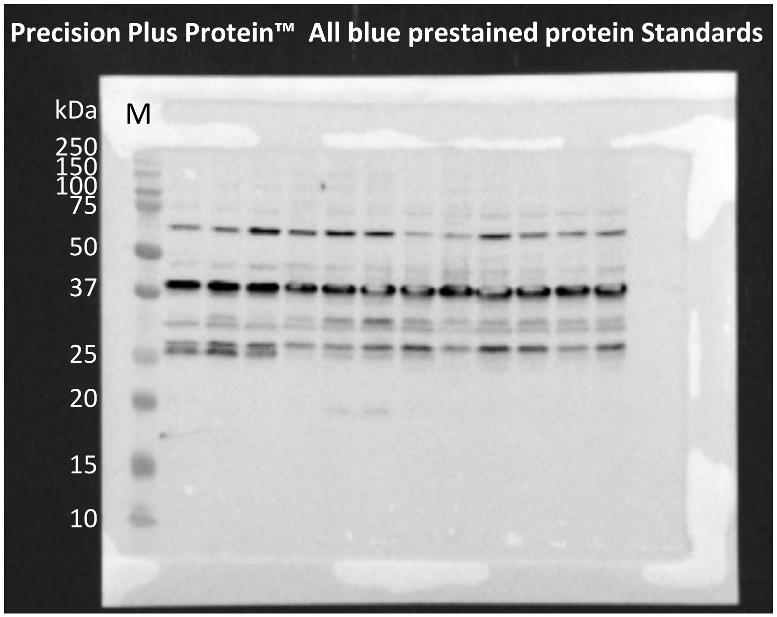
Precision Plus Protein™ All blue prestained protein Standards
kDa
M
250
150
100
75
50
37
25
20
15
10

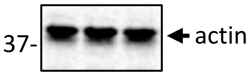
37-
actin

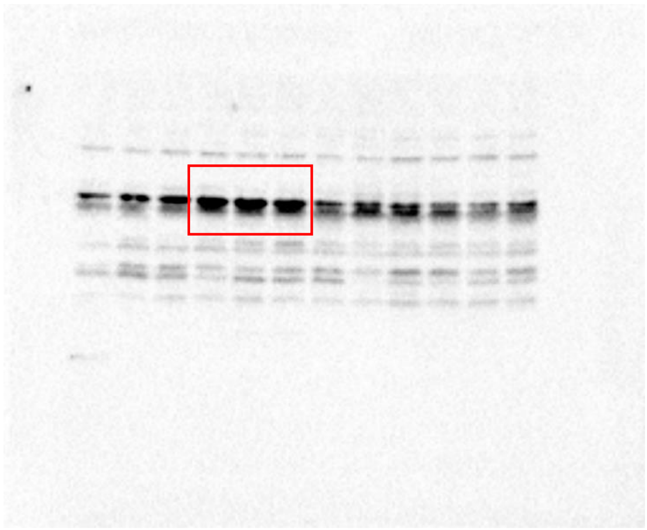

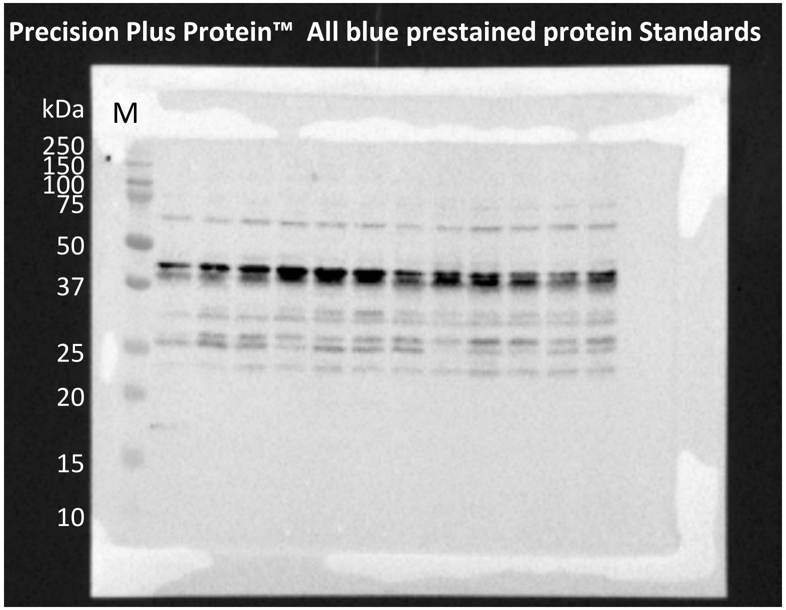
Precision Plus Protein™ All blue prestained protein Standards
kDa
M
250
150
100
75
50
37
25
20
15
10

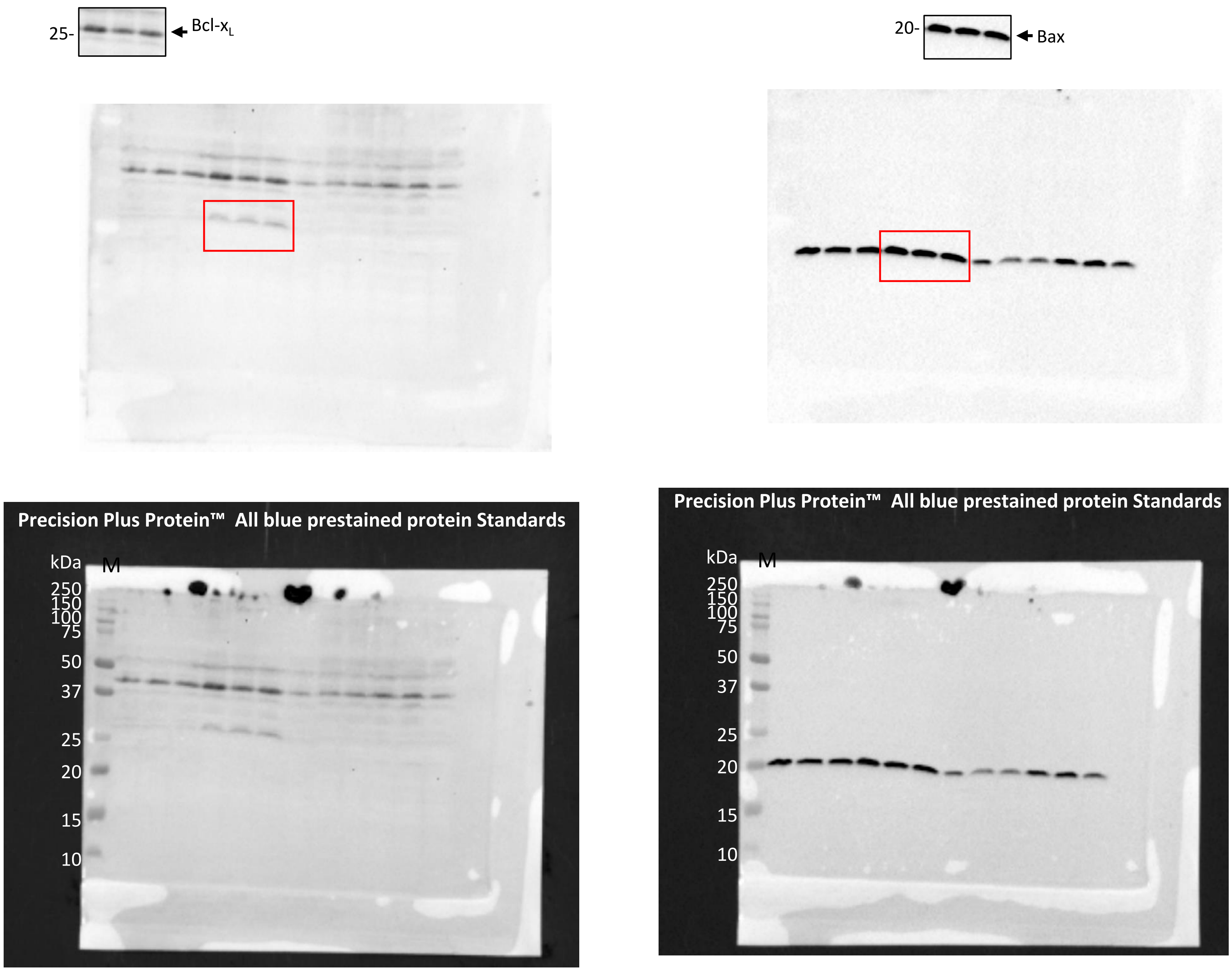
25-
Bcl-x$_L$
20-
Bax
Precision Plus Protein™ All blue prestained protein Standards
kDa
M
250
150
100
75
50
37
25
20
15
10
Precision Plus Protein™ All blue prestained protein Standards
kDa
M
250
150
100
75
50
37
25
20
15
10

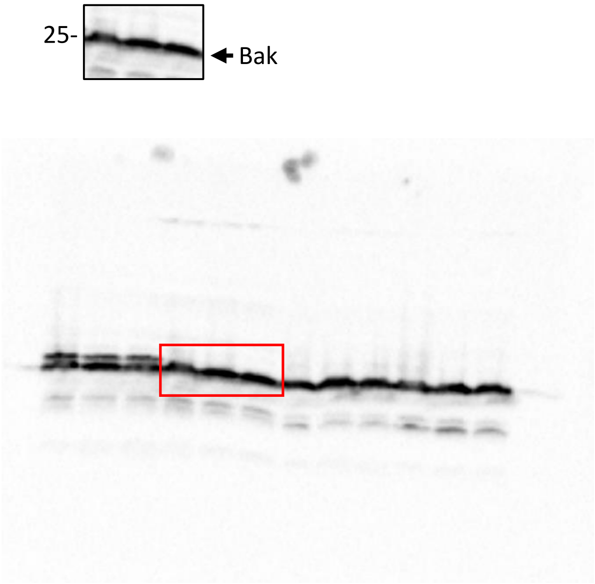
25-
Bak

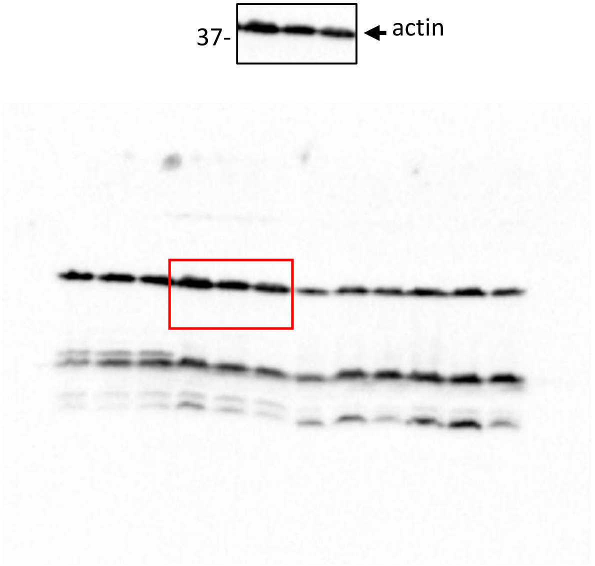
37-
actin

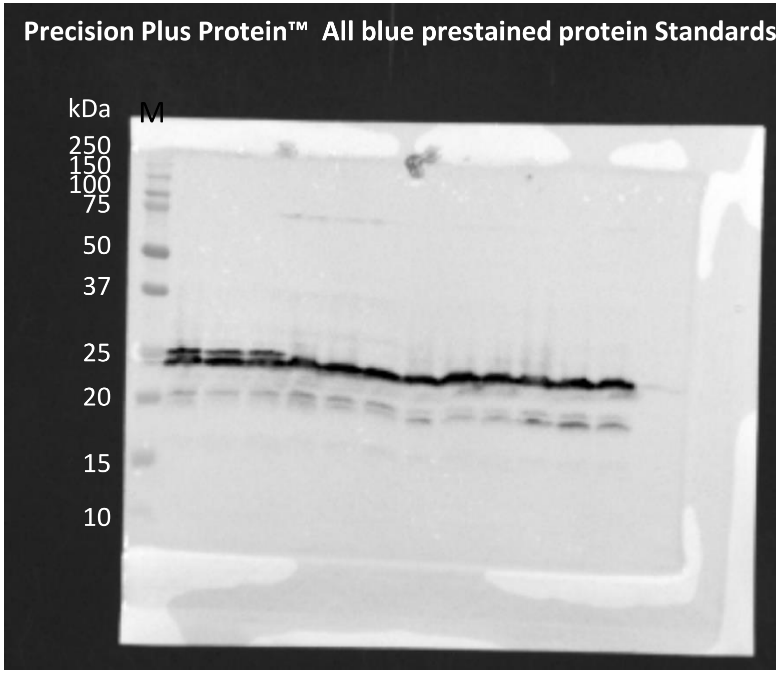
Precision Plus Protein™ All blue prestained protein Standards
kDa
M
250
150
100
75
50
37
25
20
15
10

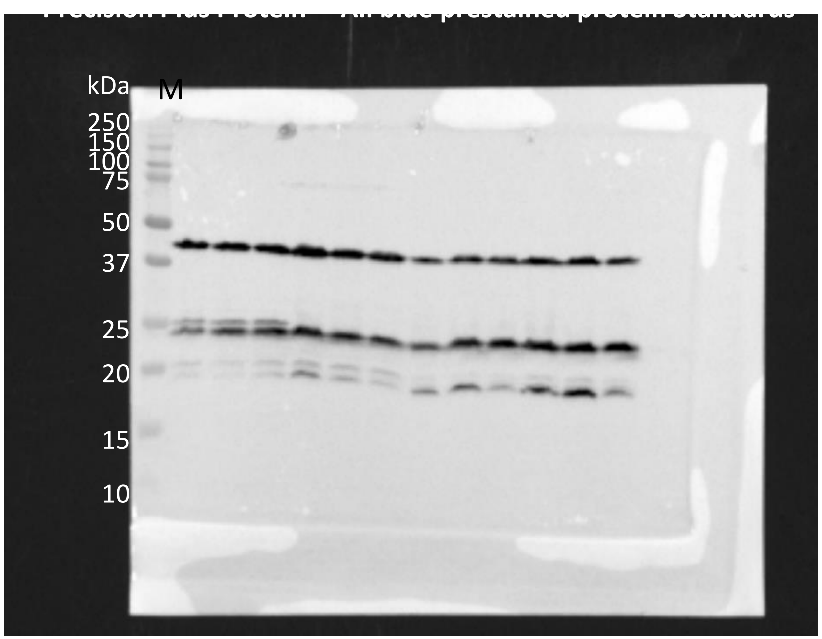
kDa
M
250
150
100
75
50
37
25
20
15
10

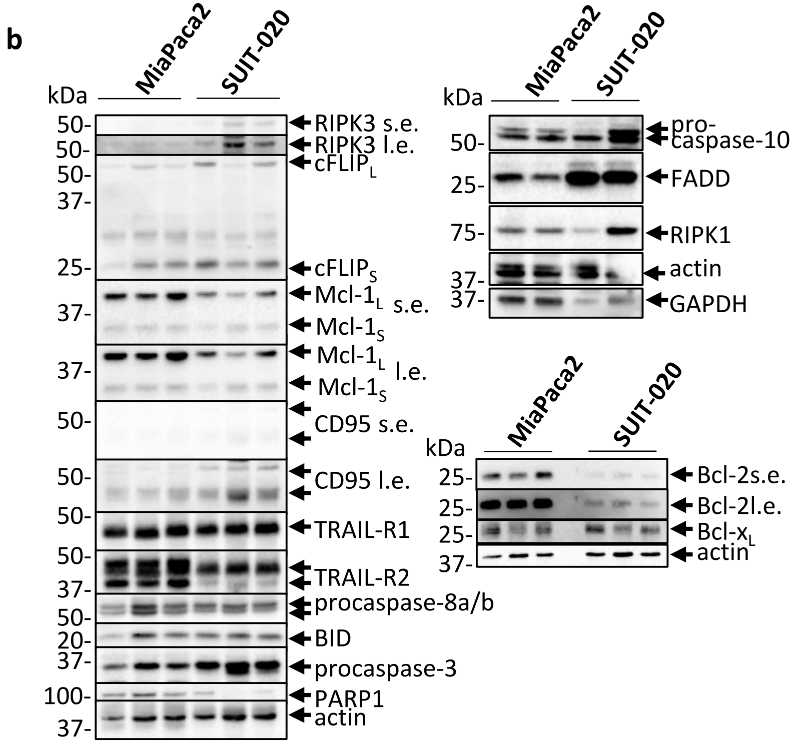
b
MiaPaca2
SUIT-020
kDa
50-
50-
50-
37-
25-
37-
37-
50-
50-
50-
50-
50-
37-
50-
20-
37-
100-
37-
RIPK3 s.e.
RIPK3 l.e.
cFLIP$_L$
cFLIP$_S$
Mcl-1$_L$
s.e.
Mcl-1$_S$
Mcl-1$_L$
l.e.
Mcl-1$_S$
CD95 s.e.
CD95 l.e.
TRAIL-R1
TRAIL-R2
procaspase-8a/b
BID
procaspase-3
PARP1
actin
MiaPaca2
SUIT-020
kDa
50-
25-
75-
37-
37-
pro-
caspase-10
FADD
RIPK1
actin
GAPDH
MiaPaca2
SUIT-020
kDa
25-
25-
25-
37-
Bcl-2s.e.
Bcl-2l.e.
Bcl-x$_L$
actin

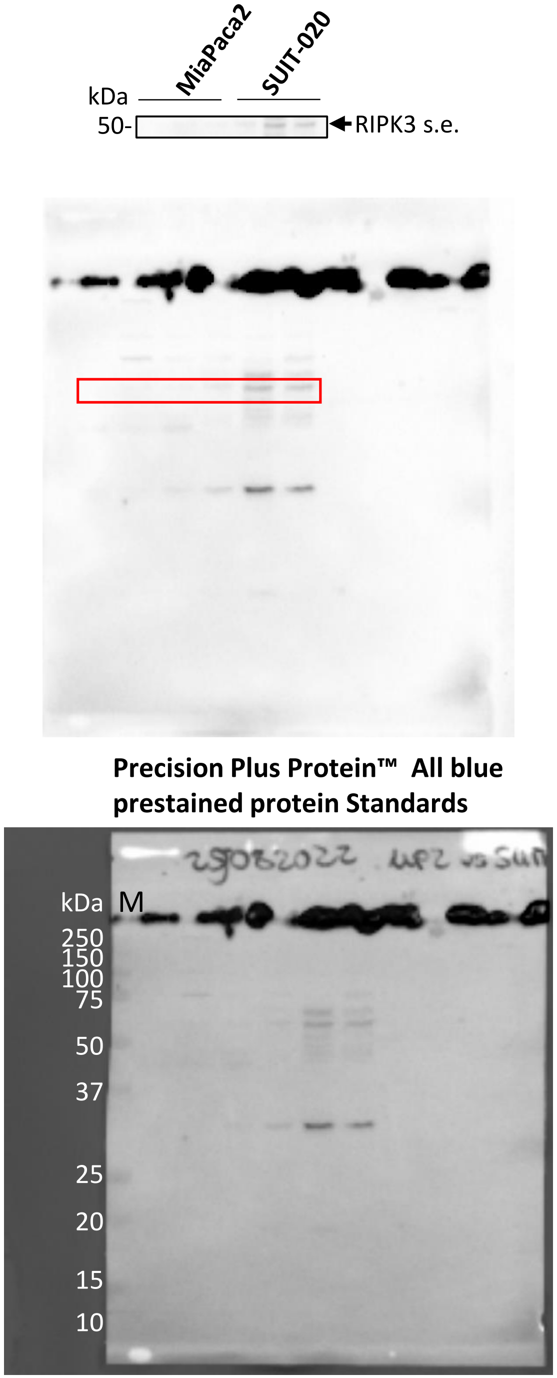
MiaPaca2
SUIT-020
kDa
50-
RIPK3 s.e.
Precision Plus Protein™ All blue prestained protein Standards
kDa
M
250
150
100
75
50
37
25
20
15
10

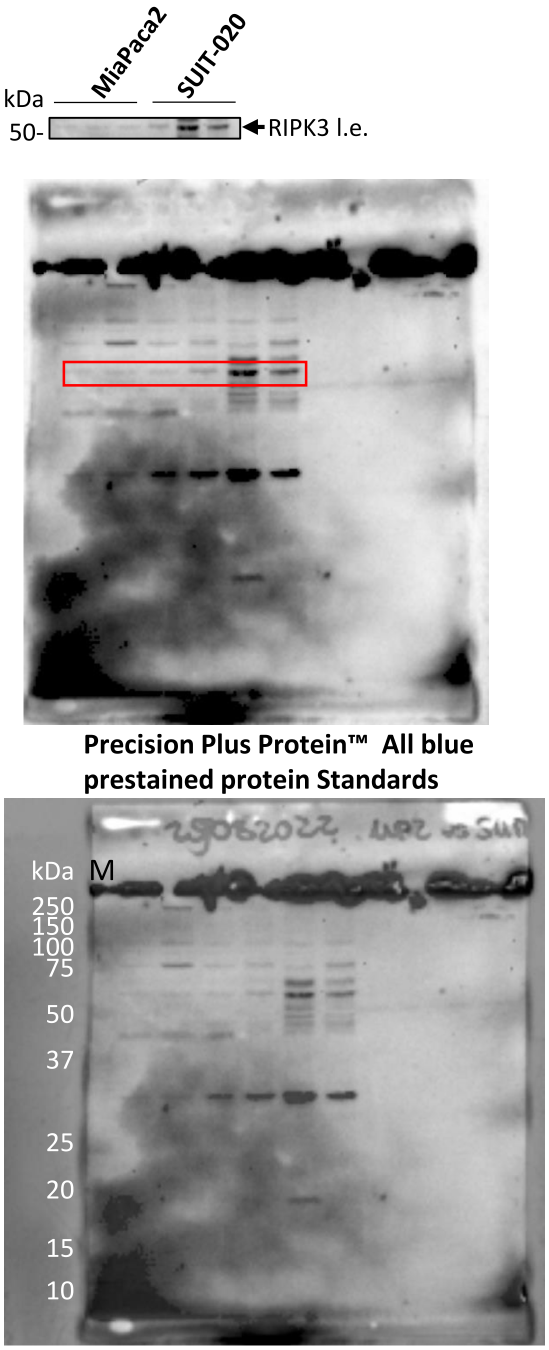
MiaPaca2
SUIT-020
kDa
50-
RIPK3 l.e.
Precision Plus Protein™ All blue prestained protein Standards
kDa
M
250
150
100
75
50
37
25
20
15
10

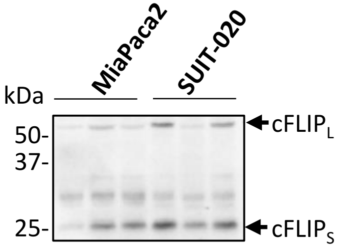

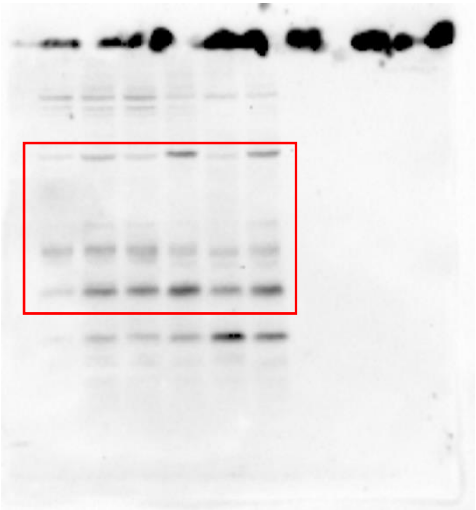

Precision Plus Protein™ All blue prestained protein Standards

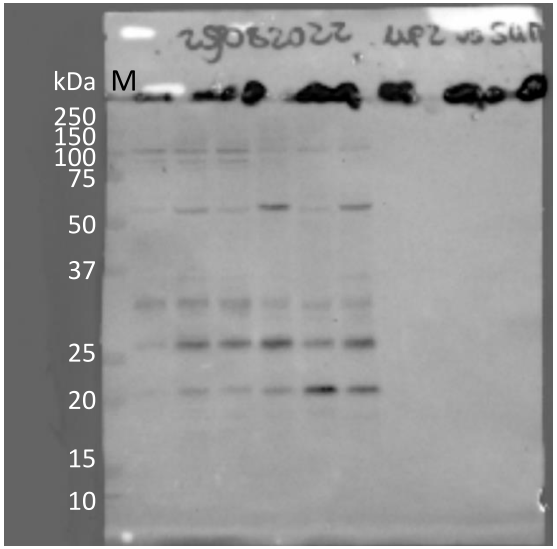

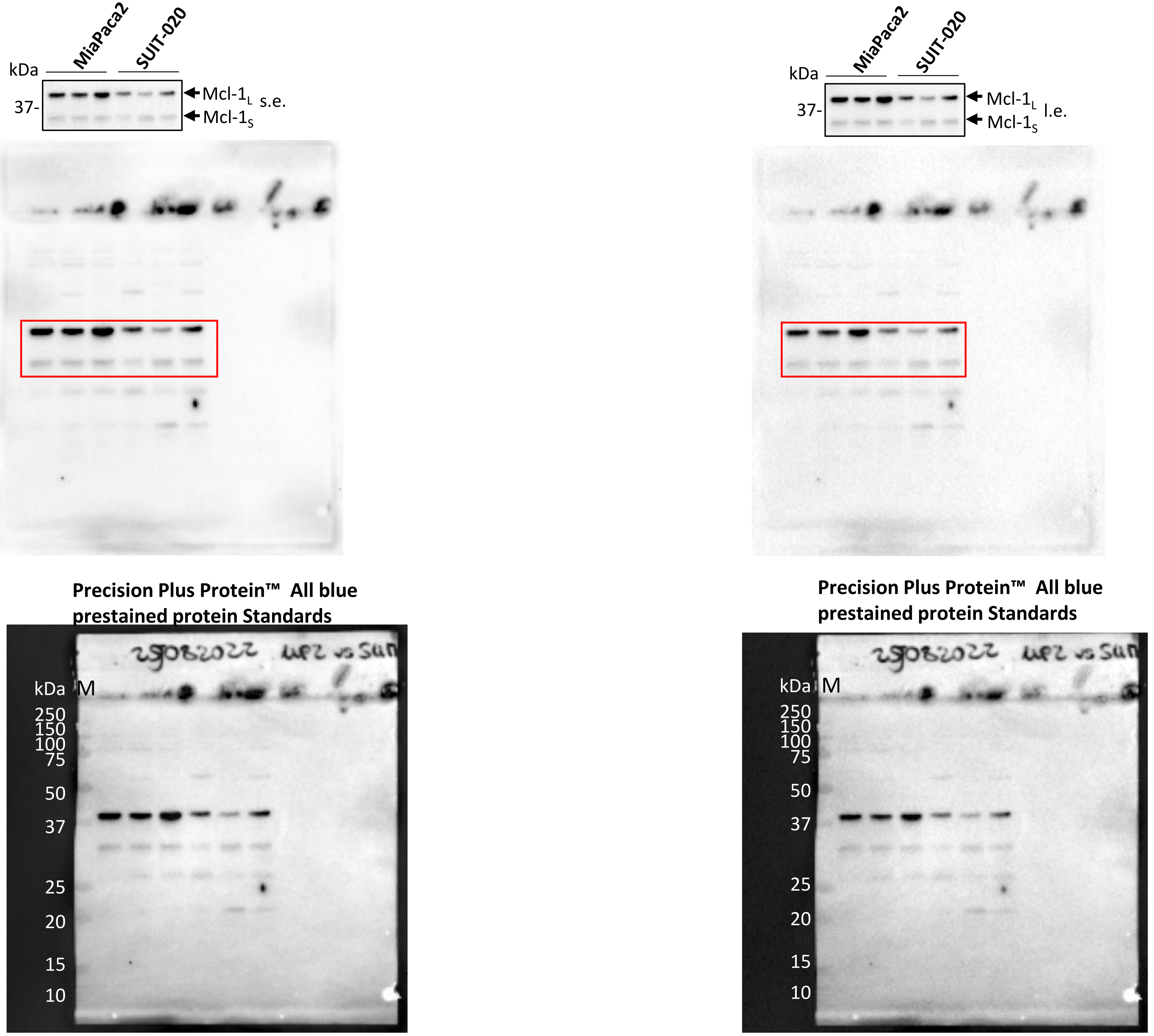
kDa
MiaPaca2
SUIT-020
37-
Mcl-1L
Mcl-1S
s.e.
l.e.
Precision Plus Protein™ All blue prestained protein Standards
kDa
M
250
150
100
75
50
37
25
20
15
10

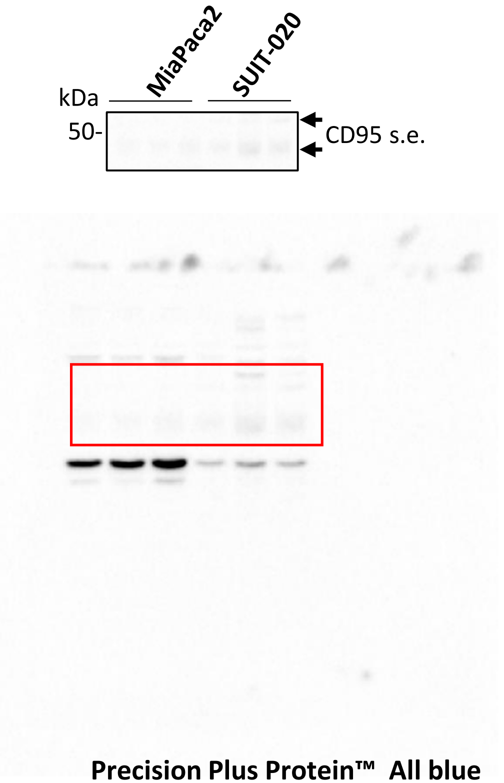

Precision Plus Protein™ All blue prestained protein Standards

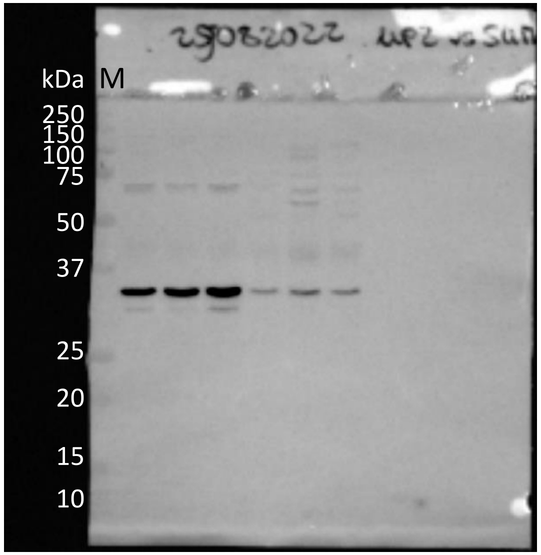

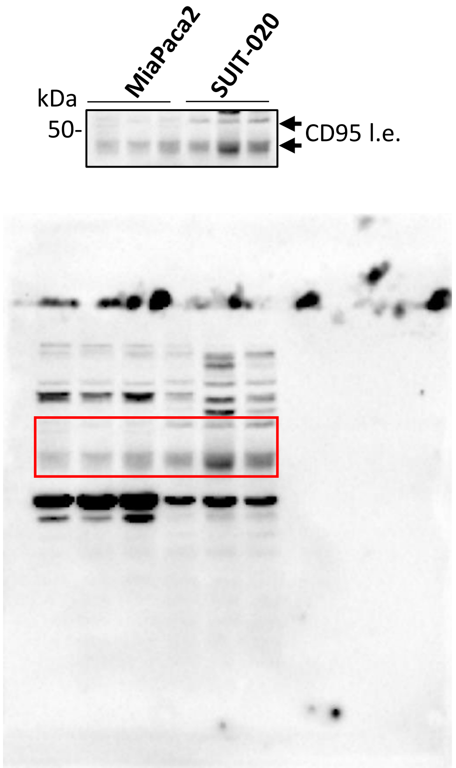

Precision Plus Protein™ All blue prestained protein Standards

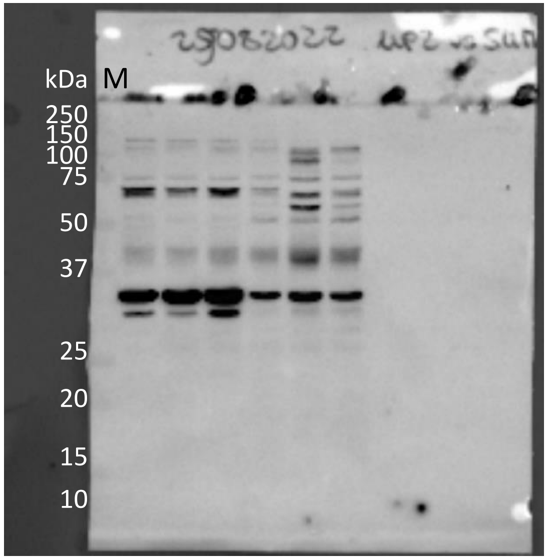

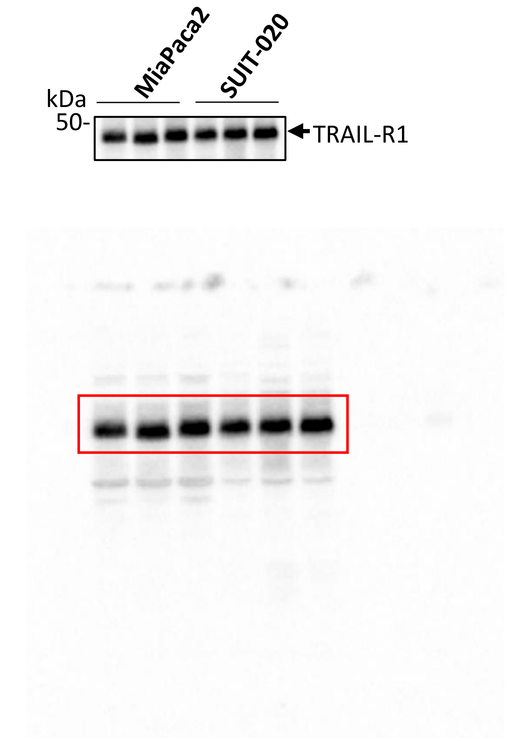

Precision Plus Protein™ All blue prestained protein Standards

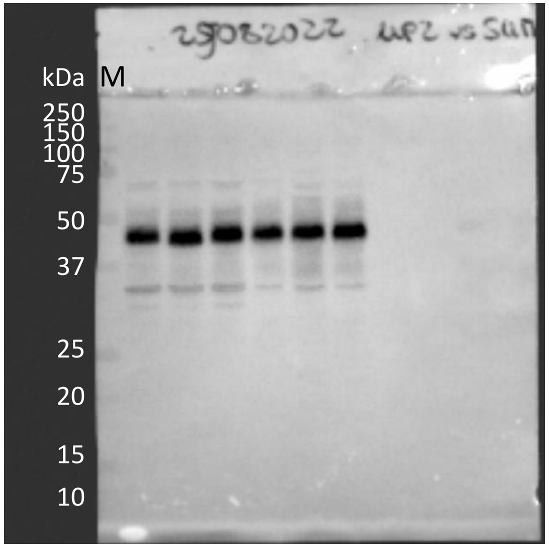

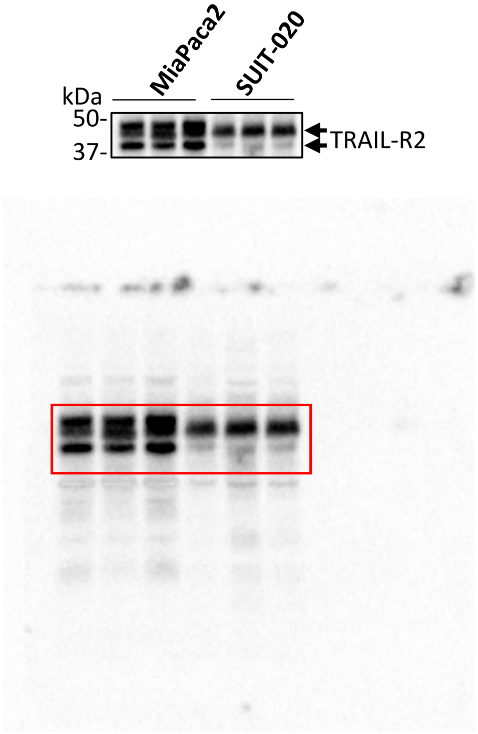

Precision Plus Protein™ All blue prestained protein Standards

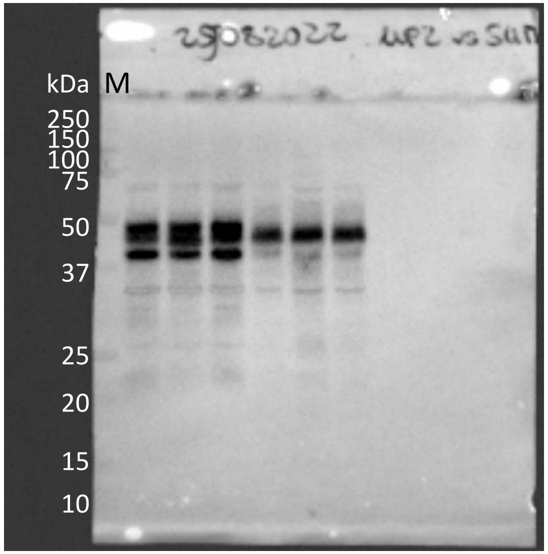

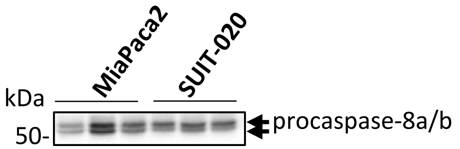

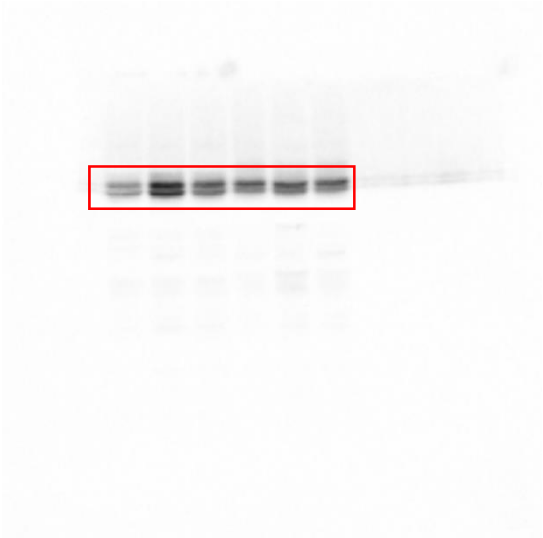

Precision Plus Protein™ All blue prestained protein Standards

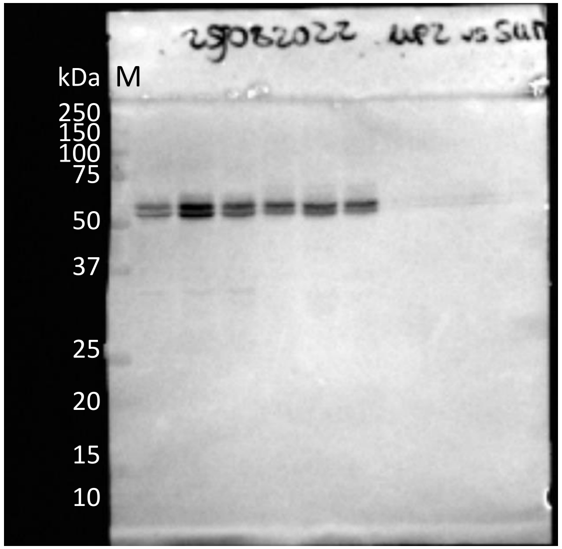

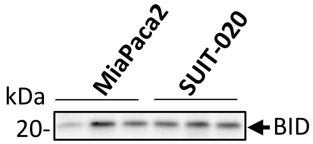

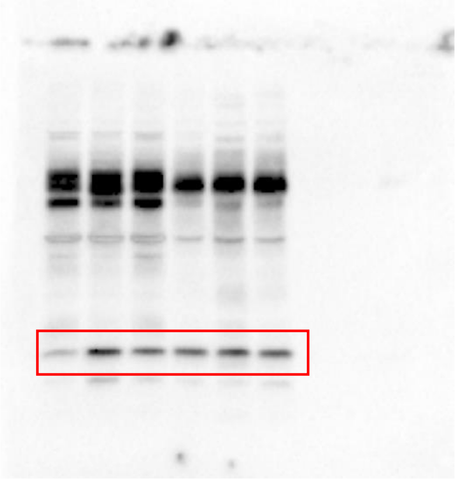

Precision Plus Protein™ All blue prestained protein Standards

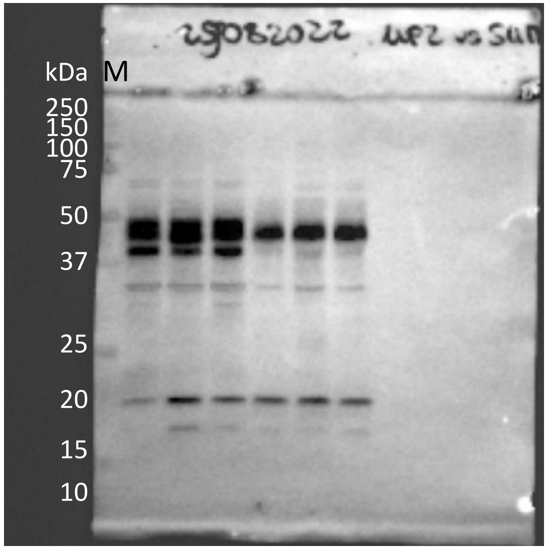

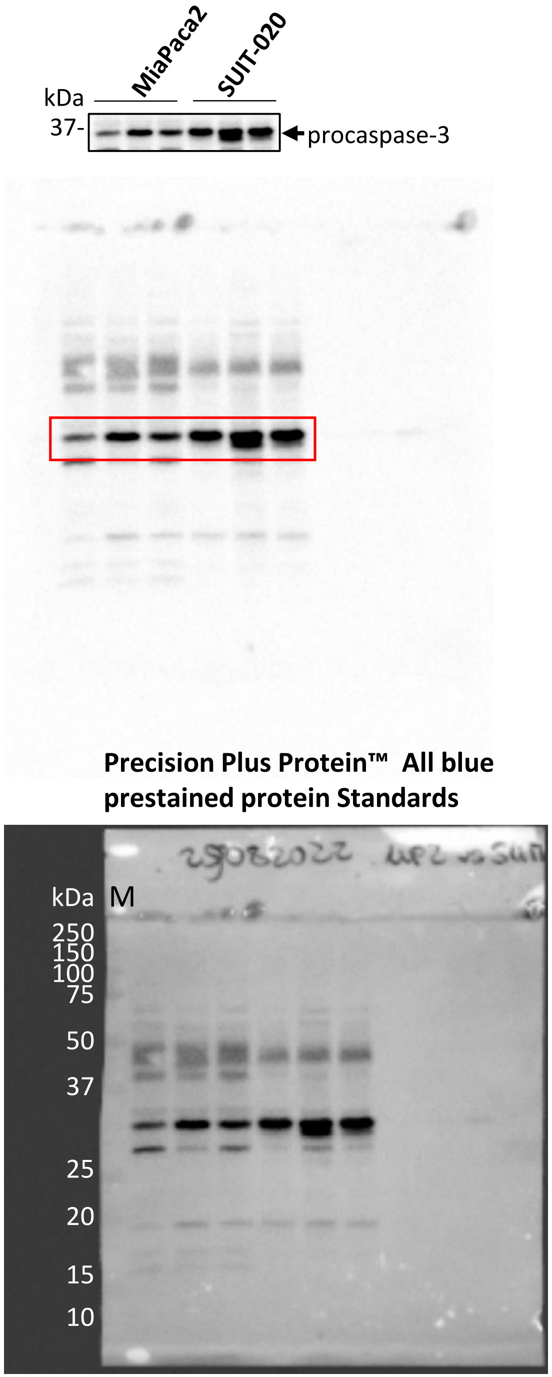
MiaPaca2
SUIT-020
kDa
37-
procaspase-3
Precision Plus Protein™ All blue prestained protein Standards
kDa
M
250
150
100
75
50
37
25
20
15
10

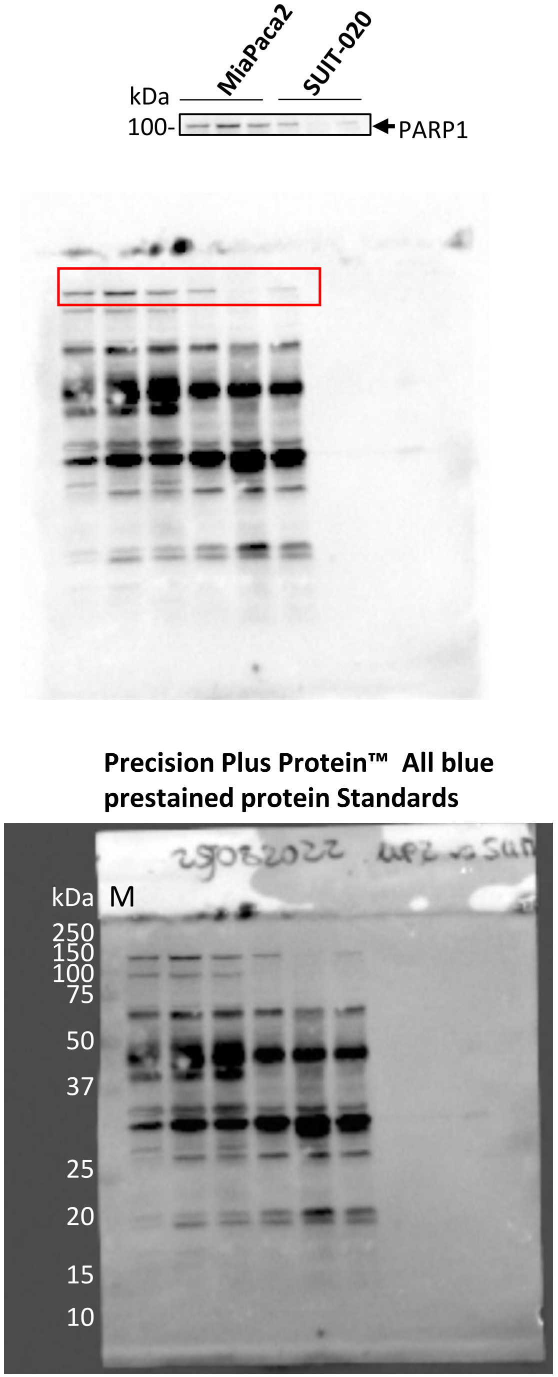
MiaPaca2
SUIT-020
kDa
100-
PARP1
Precision Plus Protein™ All blue prestained protein Standards
kDa
M
250
150
100
75
50
37
25
20
15
10

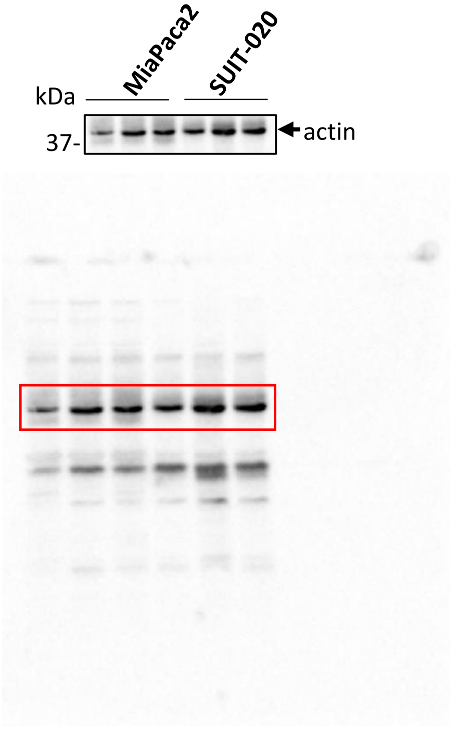

Precision Plus Protein™ All blue prestained protein Standards

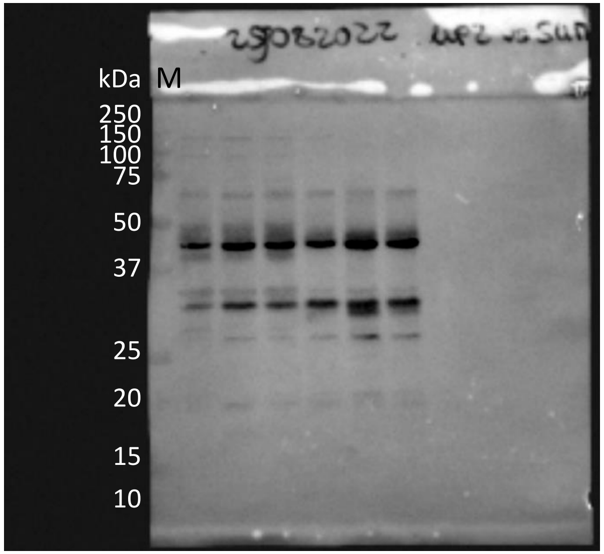

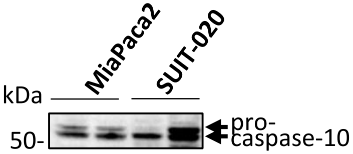

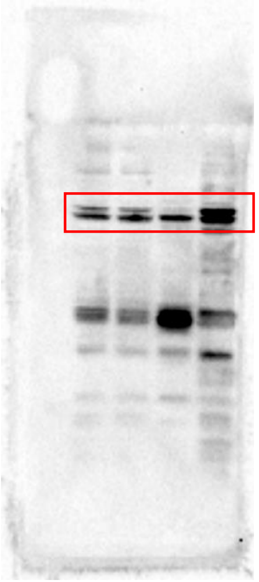

Precision Plus Protein™ All blue prestained protein Standards

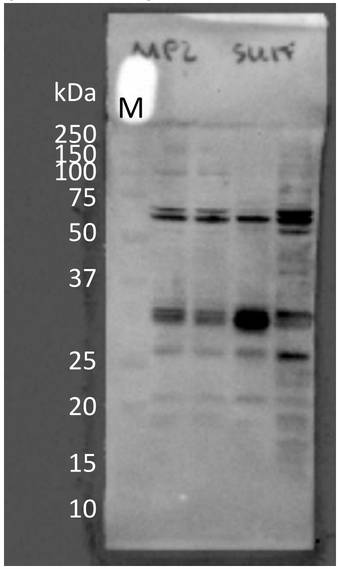

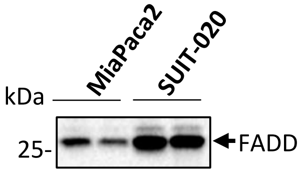

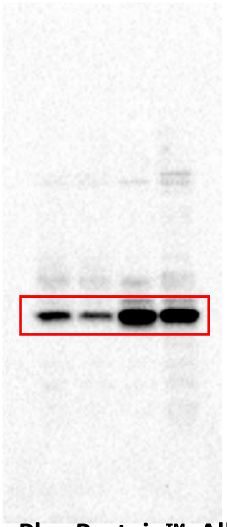

Precision Plus Protein™ All blue prestained protein Standards

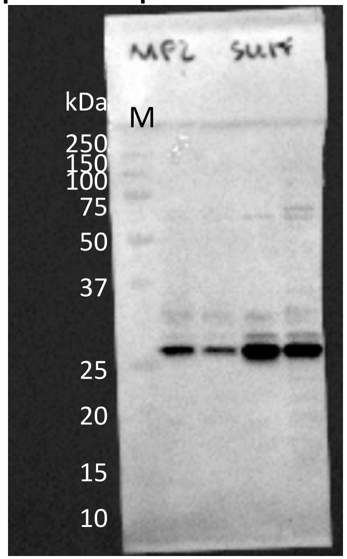

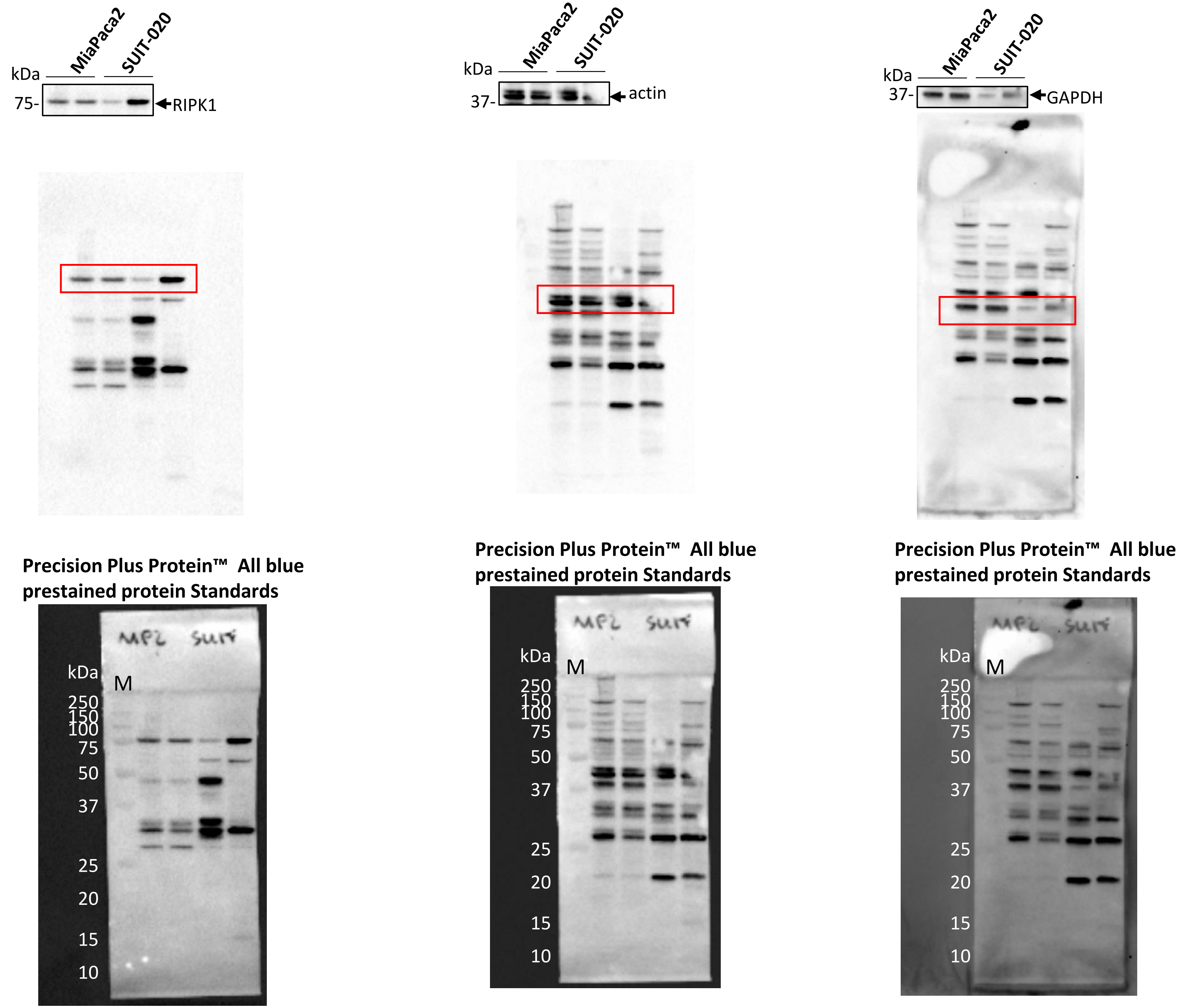

kDa
MiaPaca2
SUIT-020
75-
RIPK1
37-
actin
GAPDH
Precision Plus Protein™ All blue prestained protein Standards
M
250
150
100
75
50
37
25
20
15
10

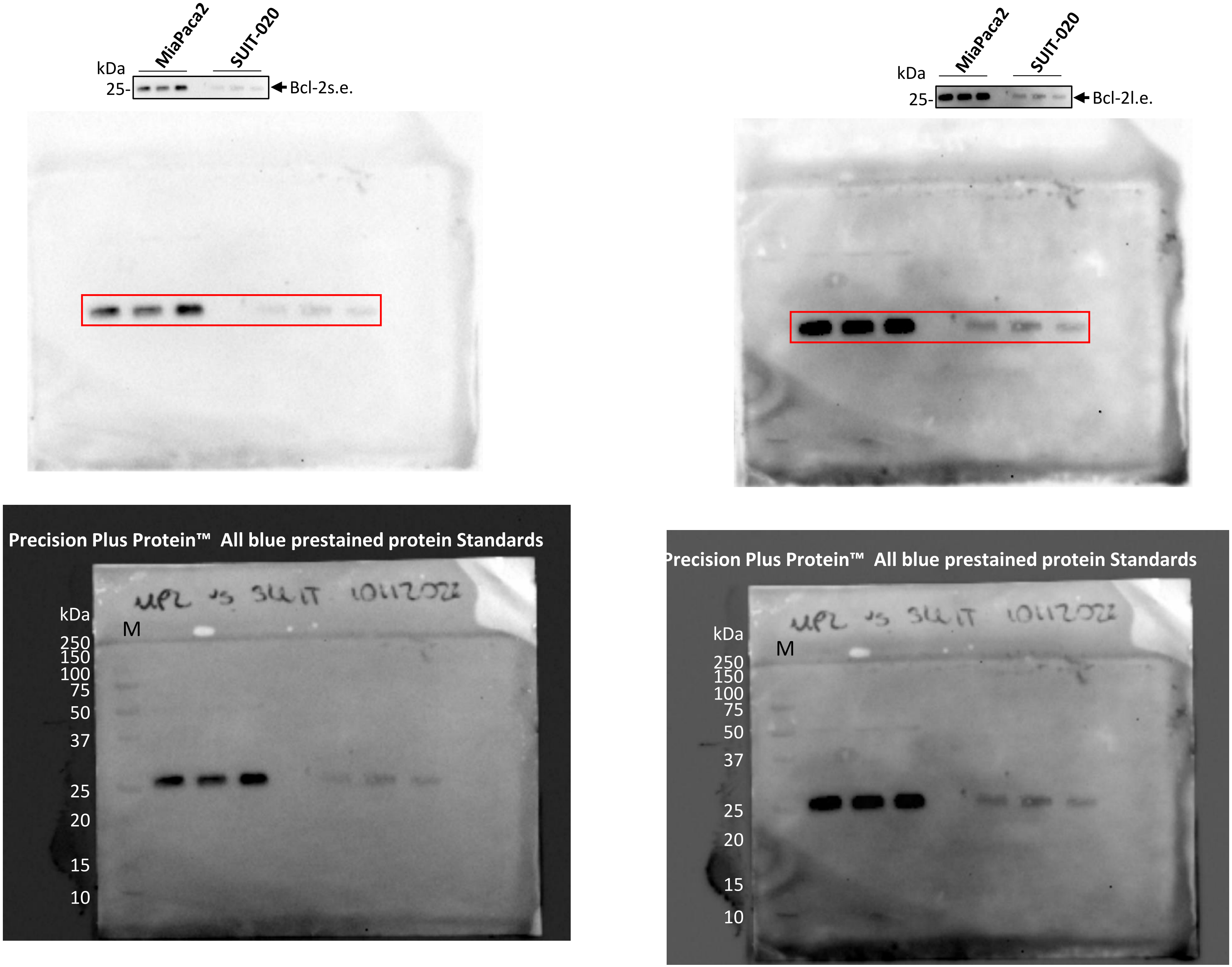

MiaPaca2
SUIT-020
kDa
25-
Bcl-2s.e.
MiaPaca2
SUIT-020
kDa
25-
Bcl-2l.e.
Precision Plus Protein™ All blue prestained protein Standards
kDa
M
250
150
100
75
50
37
25
20
15
10
Precision Plus Protein™ All blue prestained protein Standards
kDa
M
250
150
100
75
50
37
25
20
15
10

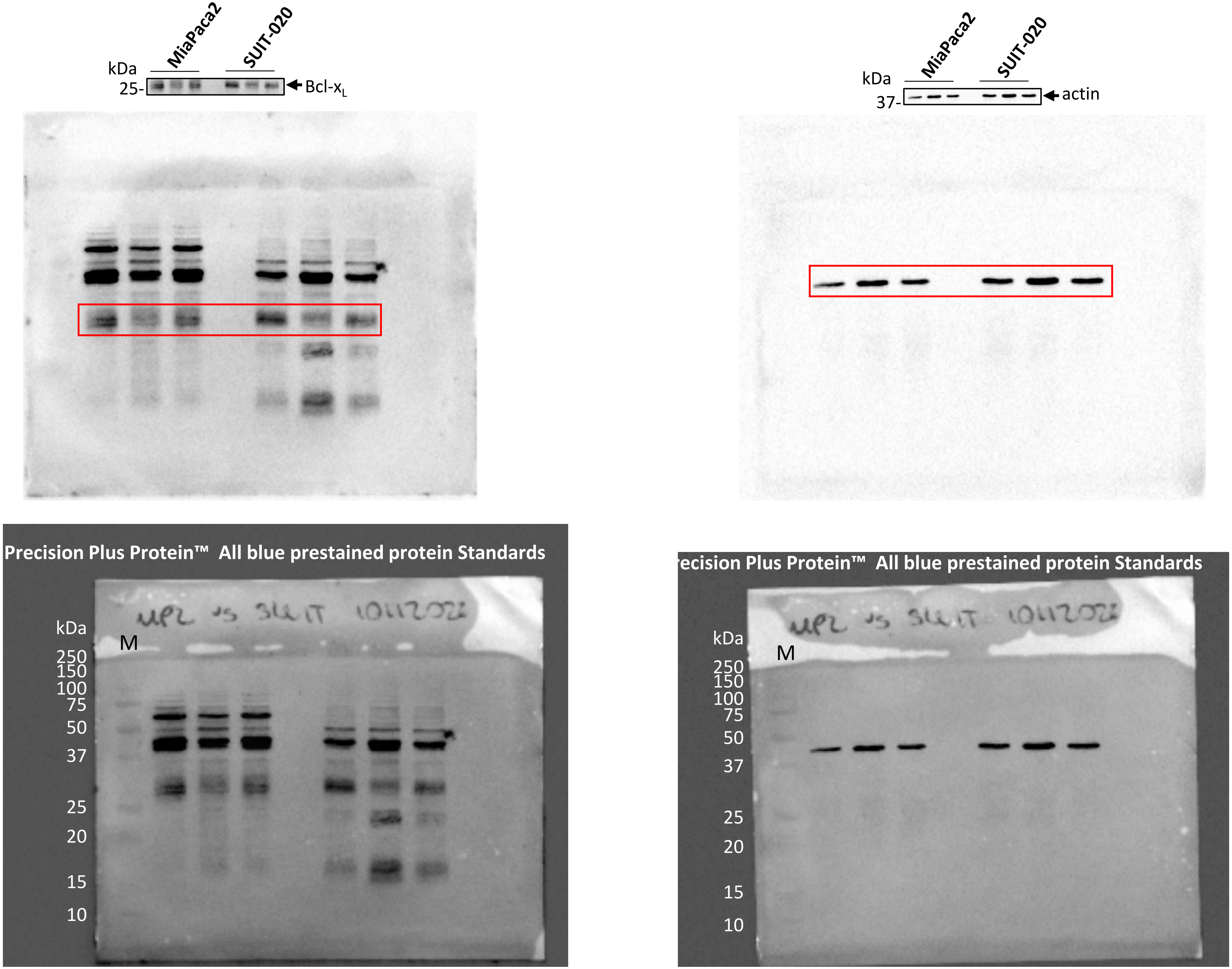
kDa
MiaPaca2
SUIT-020
25-
Bcl-xL
kDa
MiaPaca2
SUIT-020
37-
actin
Precision Plus Protein™ All blue prestained protein Standards
kDa
M
250
150
100
75
50
37
25
20
15
10
ecision Plus Protein™ All blue prestained protein Standards
kDa
M
250
150
100
75
50
37
25
20
15
10

d

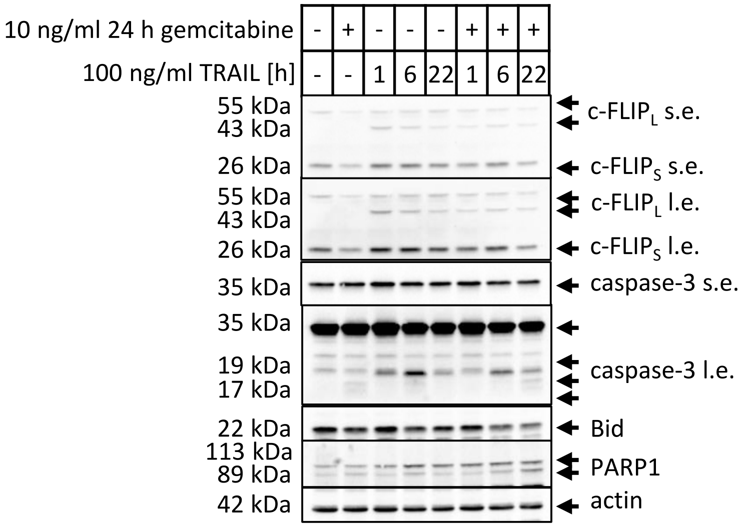
10 ng/ml 24 h gemcitabine
- + - - - + + +
100 ng/ml TRAIL [h]
- - 1 6 22 1 6 22
55 kDa
43 kDa
26 kDa
55 kDa
43 kDa
26 kDa
35 kDa
35 kDa
19 kDa
17 kDa
22 kDa
113 kDa
89 kDa
42 kDa
c-FLIP_L s.e.
c-FLIP_S s.e.
c-FLIP_L l.e.
c-FLIP_S l.e.
caspase-3 s.e.
caspase-3 l.e.
Bid
PARP1
actin

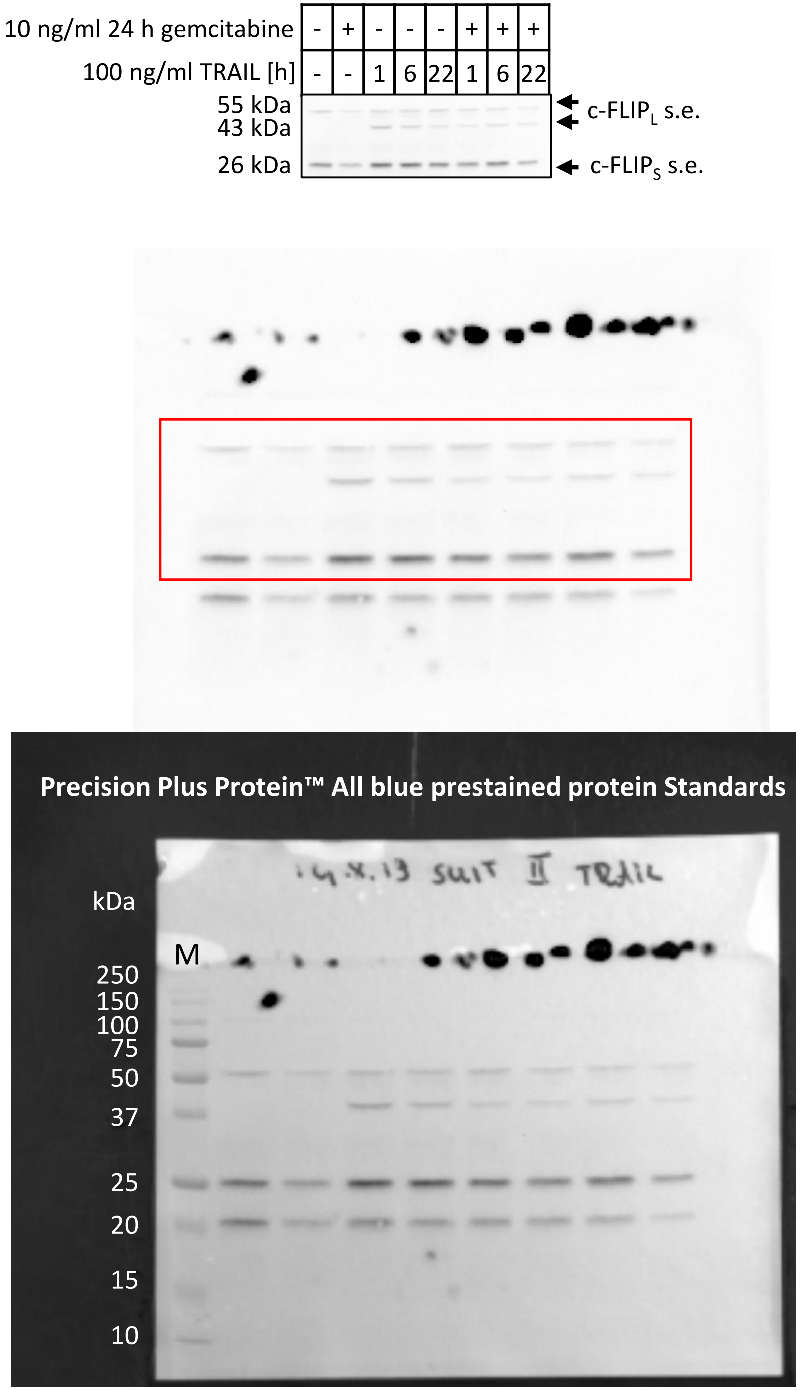
10 ng/ml 24 h gemcitabine
- + - - - + + +
100 ng/ml TRAIL [h]
- - 1 6 22 1 6 22
55 kDa
43 kDa
26 kDa
c-FLIP$_L$ s.e.
c-FLIP$_S$ s.e.
Precision Plus Protein™ All blue prestained protein Standards
kDa
M
250
150
100
75
50
37
25
20
15
10

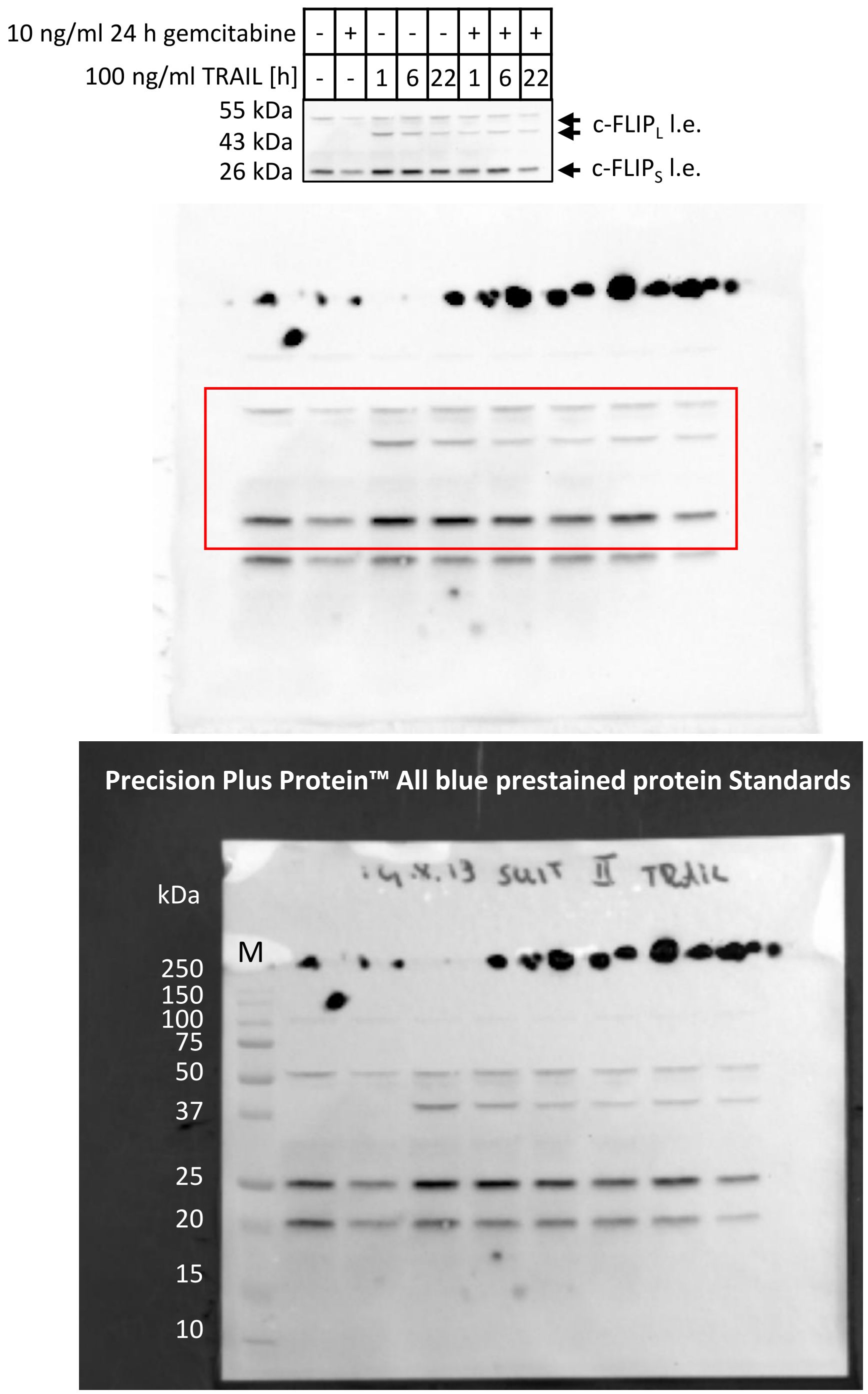
10 ng/ml 24 h gemcitabine
- + - - - + + +
100 ng/ml TRAIL [h]
- - 1 6 22 1 6 22
55 kDa
43 kDa
26 kDa
c-FLIP$_L$ l.e.
c-FLIP$_S$ l.e.
Precision Plus Protein™ All blue prestained protein Standards
kDa
M
250
150
100
75
50
37
25
20
15
10

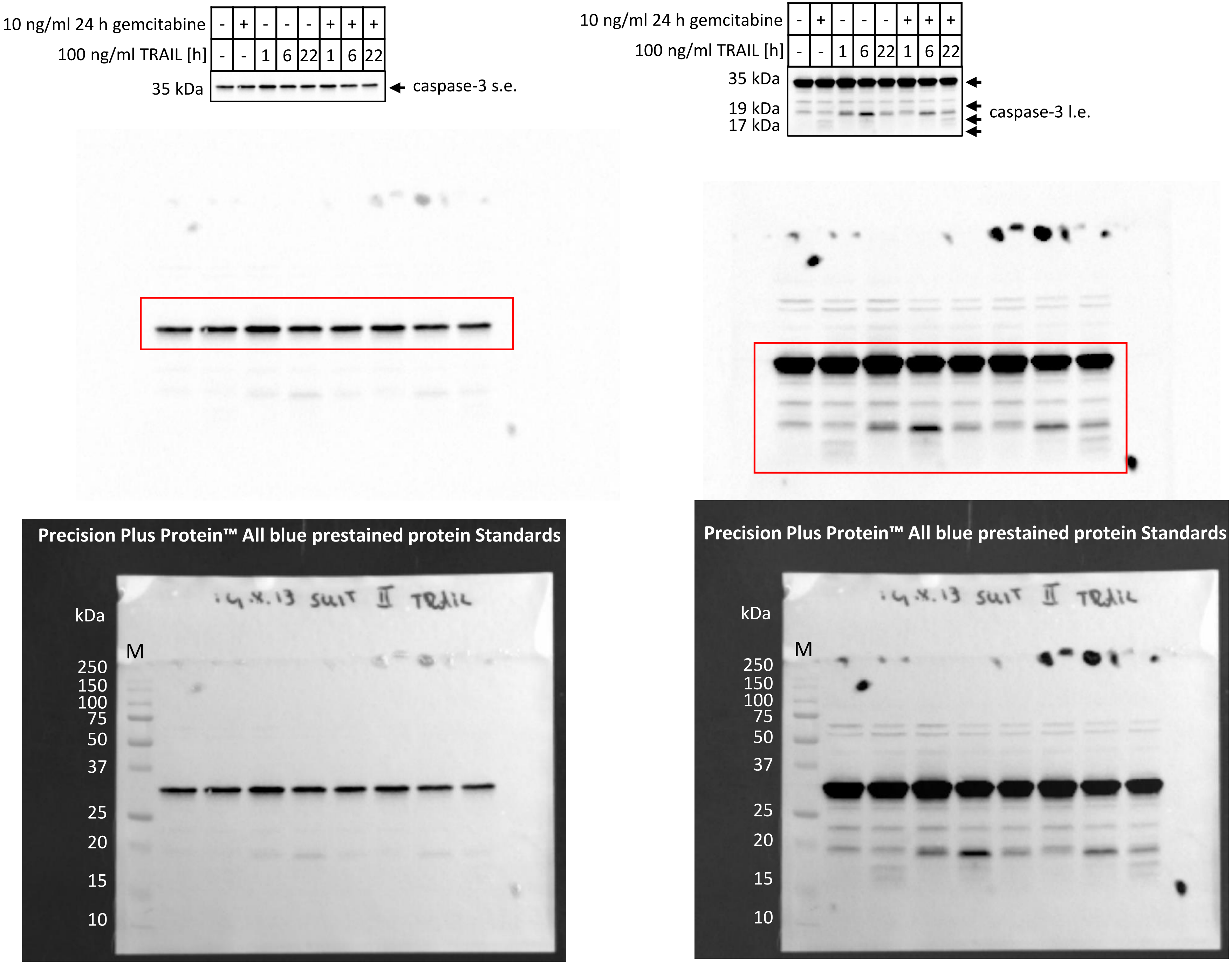

10 ng/ml 24 h gemcitabine
- + - - - + + +
100 ng/ml TRAIL [h]
- - 1 6 22 1 6 22
35 kDa
caspase-3 s.e.
10 ng/ml 24 h gemcitabine
- + - - - + + +
100 ng/ml TRAIL [h]
- - 1 6 22 1 6 22
35 kDa
19 kDa
17 kDa
caspase-3 l.e.
Precision Plus Protein™ All blue prestained protein Standards
kDa
M
250
150
100
75
50
37
25
20
15
10
Precision Plus Protein™ All blue prestained protein Standards
kDa
M
250
150
100
75
50
37
25
20
15
10

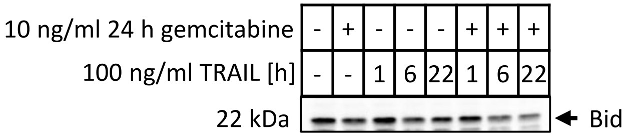
10 ng/ml 24 h gemcitabine
- + - - - + + +
100 ng/ml TRAIL [h]
- - 1 6 22 1 6 22
22 kDa
Bid

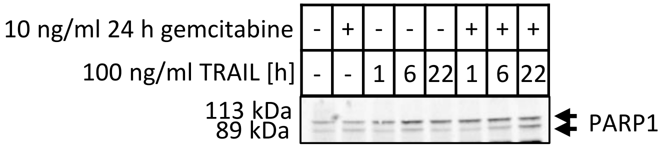
10 ng/ml 24 h gemcitabine
- + - - - + + +
100 ng/ml TRAIL [h]
- - 1 6 22 1 6 22
113 kDa
89 kDa
PARP1

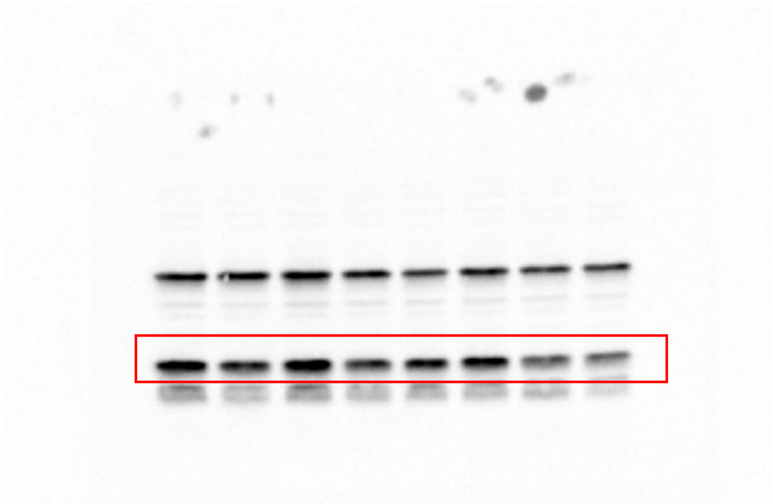

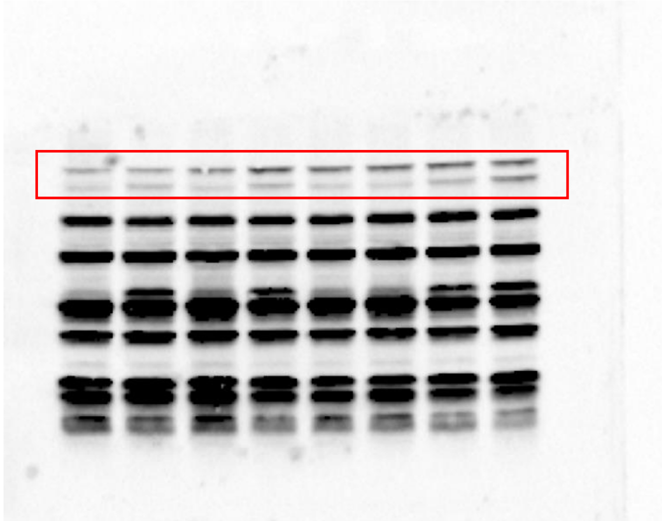

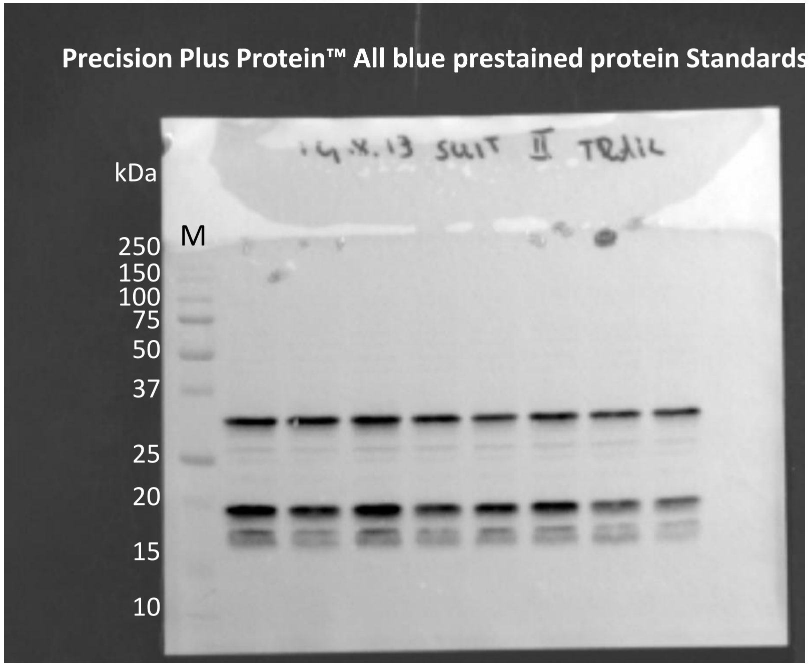
Precision Plus Protein™ All blue prestained protein Standards
kDa
M
250
150
100
75
50
37
25
20
15
10
14.X.13 SUIT II TRAIL

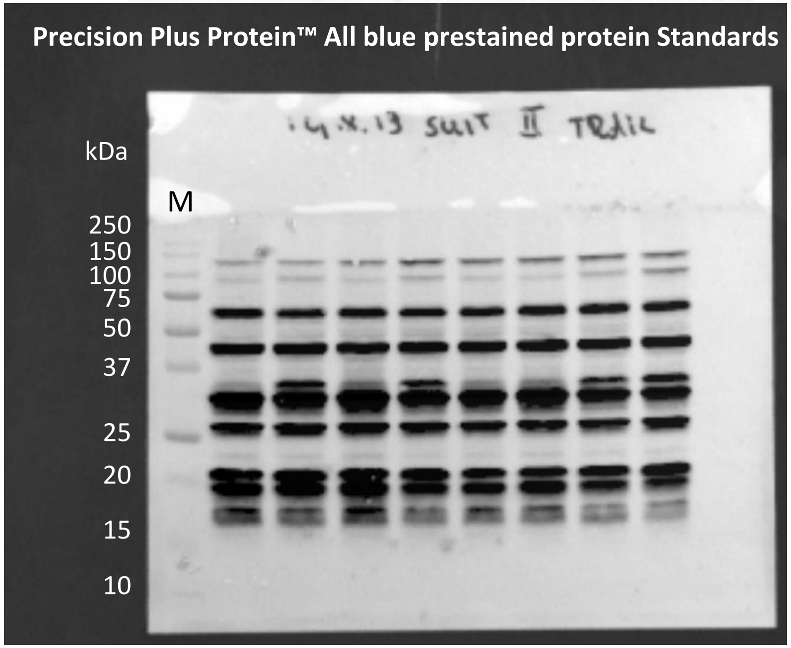
Precision Plus Protein™ All blue prestained protein Standards
kDa
M
250
150
100
75
50
37
25
20
15
10
14.X.13 SUIT II TRAIL

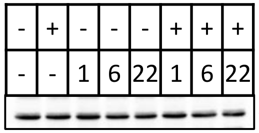
10 ng/ml 24 h gemcitabine
- + - - - + + +
100 ng/ml TRAIL [h]
- - 1 6 22 1 6 22
42 kDa
actin

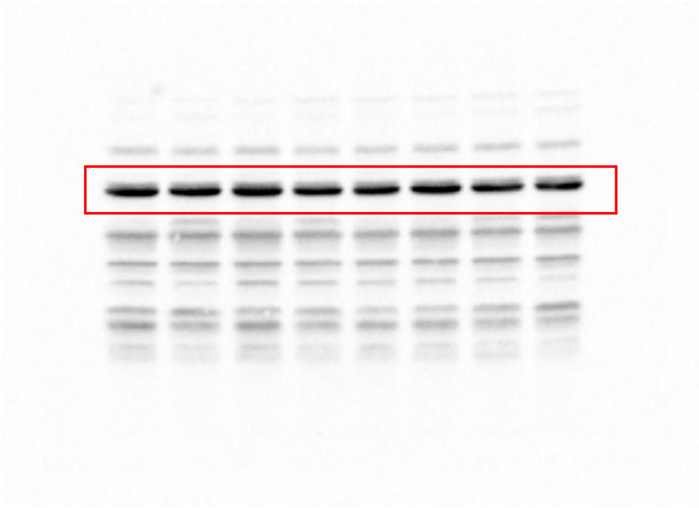

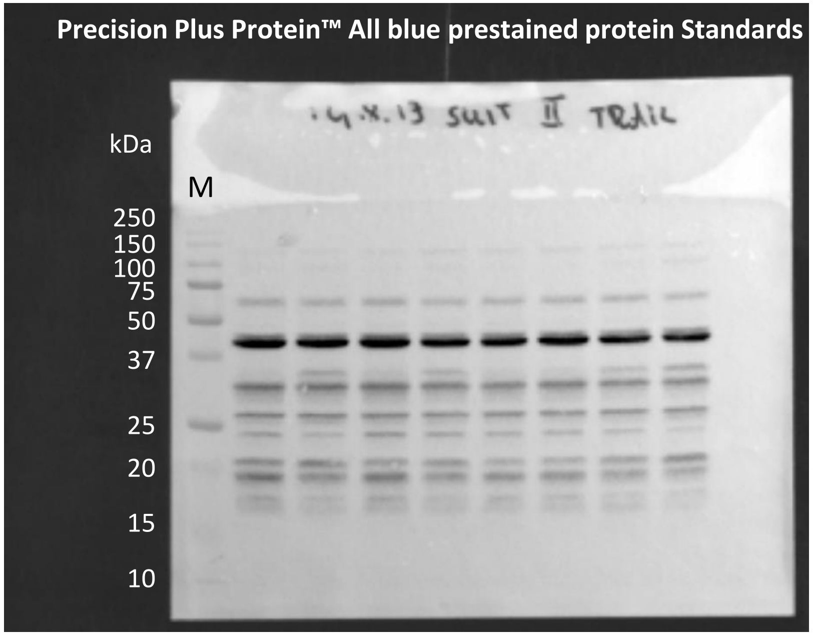
Precision Plus Protein™ All blue prestained protein Standards
kDa
M
250
150
100
75
50
37
25
20
15
10

c

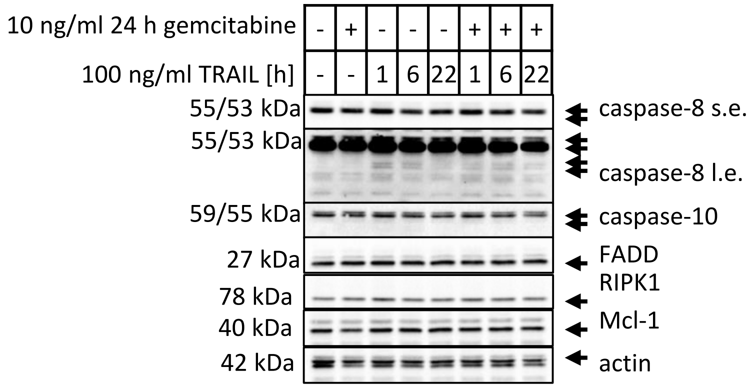
10 ng/ml 24 h gemcitabine
- + - - - + + +
100 ng/ml TRAIL [h]
- - 1 6 22 1 6 22
55/53 kDa
caspase-8 s.e.
55/53 kDa
caspase-8 l.e.
59/55 kDa
caspase-10
27 kDa
FADD
RIPK1
78 kDa
Mcl-1
40 kDa
42 kDa
actin

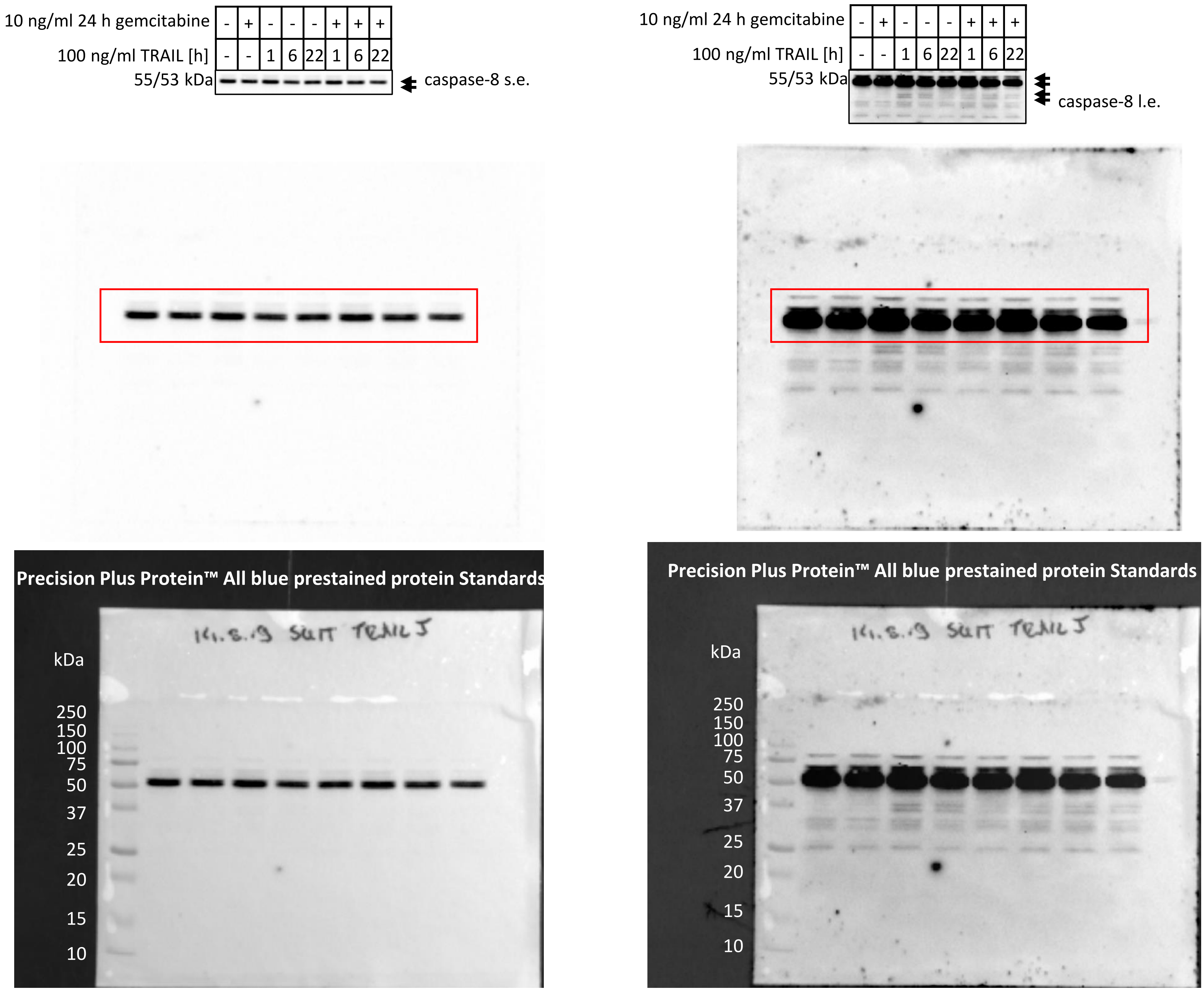
10 ng/ml 24 h gemcitabine
- + - - - + + +
100 ng/ml TRAIL [h]
- - 1 6 22 1 6 22
55/53 kDa
caspase-8 s.e.
10 ng/ml 24 h gemcitabine
- + - - - + + +
100 ng/ml TRAIL [h]
- - 1 6 22 1 6 22
55/53 kDa
caspase-8 l.e.
Precision Plus Protein™ All blue prestained protein Standards
kDa
250
150
100
75
50
37
25
20
15
10
Precision Plus Protein™ All blue prestained protein Standards
kDa
250
150
100
75
50
37
25
20
15
10

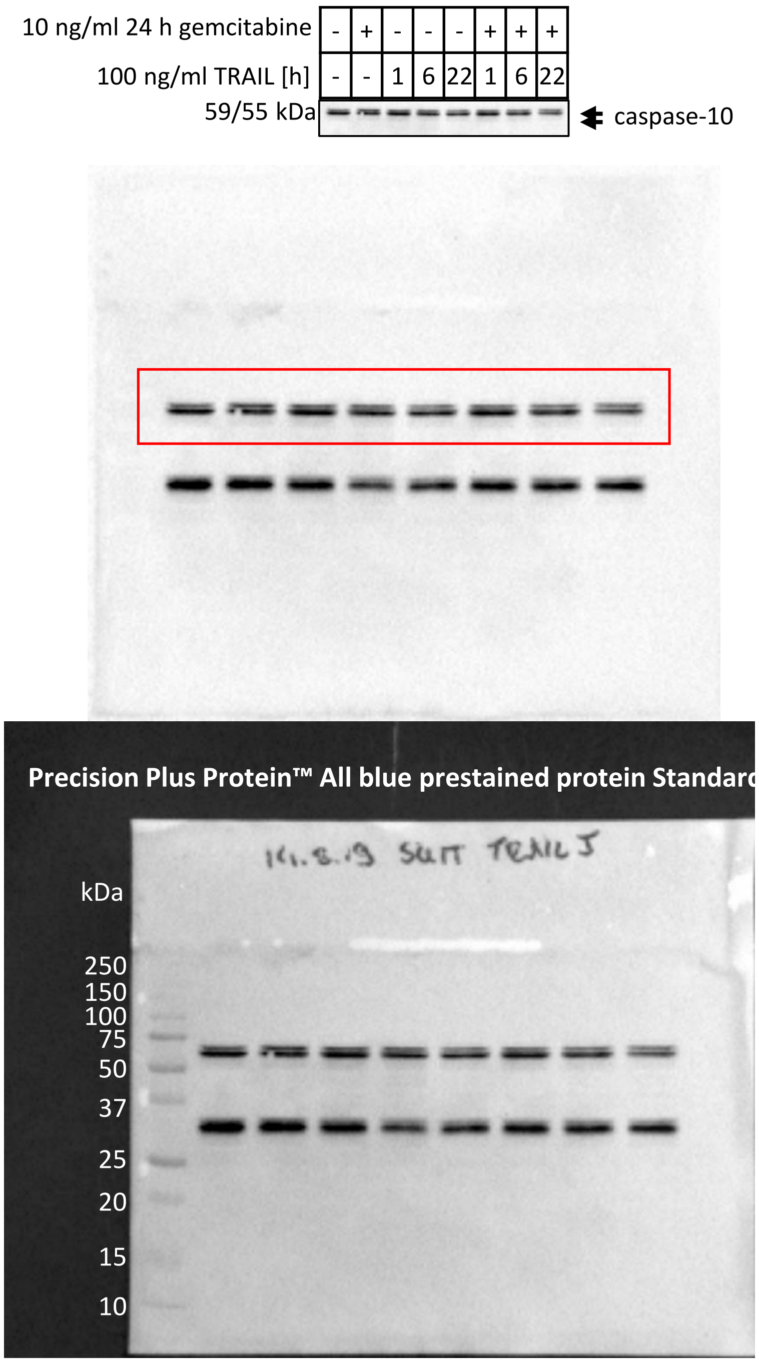
10 ng/ml 24 h gemcitabine
- + - - - + + +
100 ng/ml TRAIL [h]
- - 1 6 22 1 6 22
59/55 kDa
caspase-10
Precision Plus Protein™ All blue prestained protein Standard
kDa
250
150
100
75
50
37
25
20
15
10

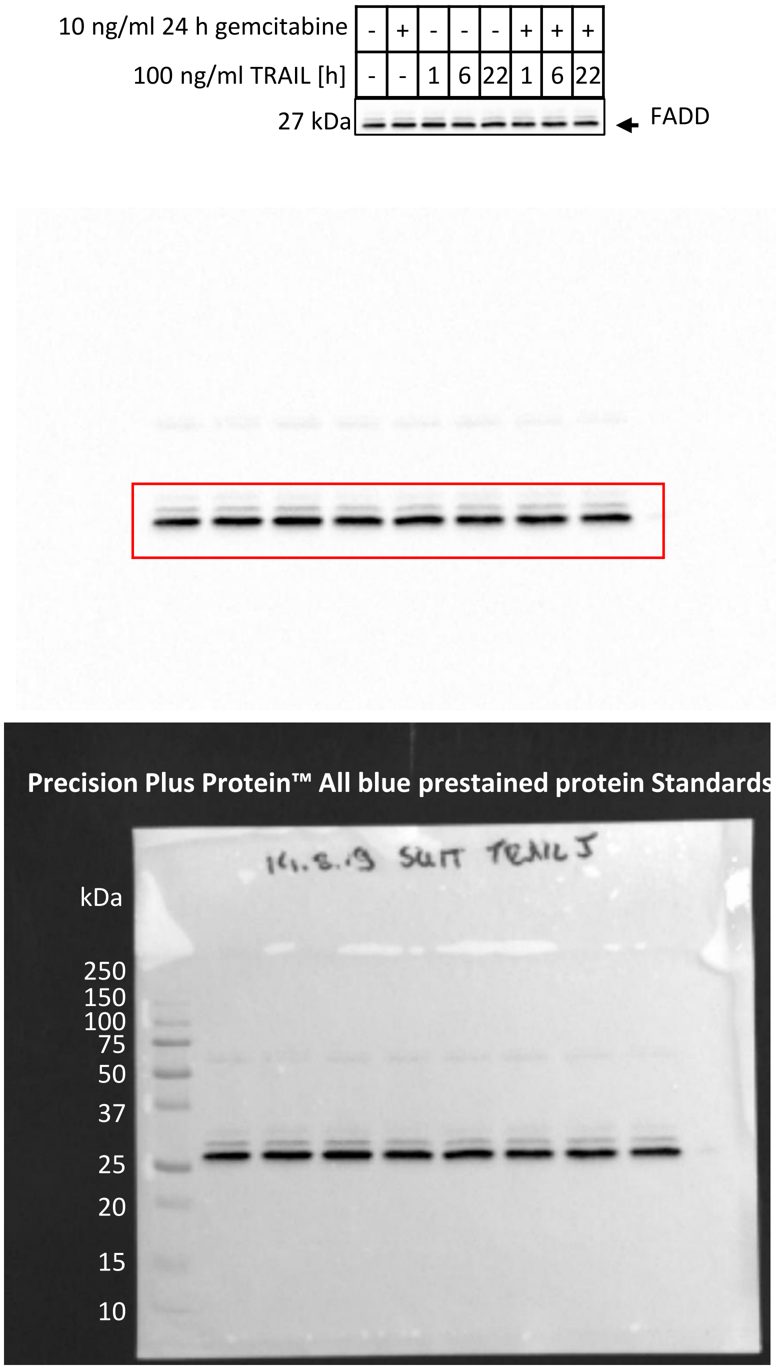
10 ng/ml 24 h gemcitabine
- + - - - + + +
100 ng/ml TRAIL [h]
- - 1 6 22 1 6 22
27 kDa
FADD
Precision Plus Protein™ All blue prestained protein Standards
kDa
250
150
100
75
50
37
25
20
15
10

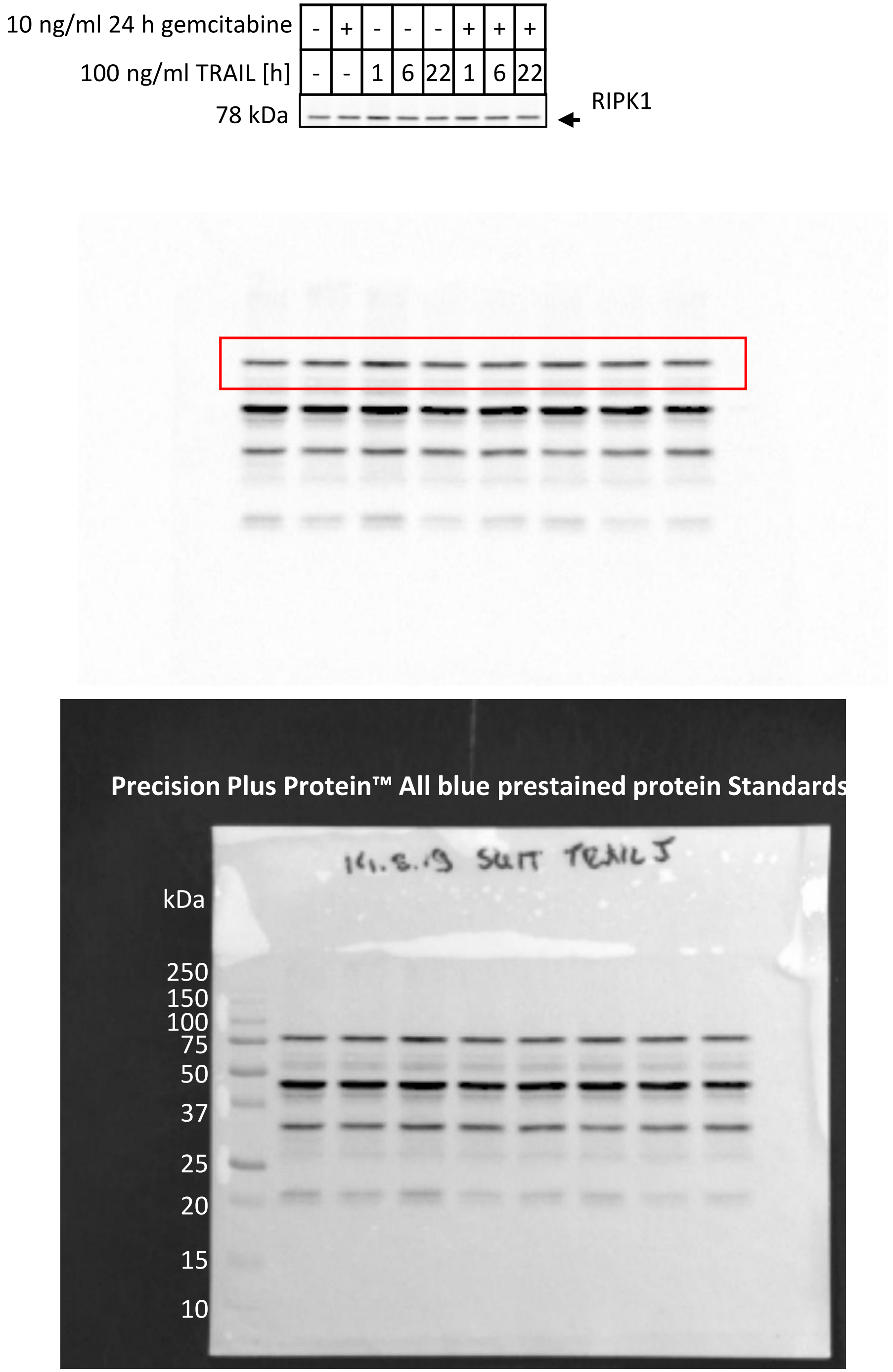
10 ng/ml 24 h gemcitabine - + - - - + + +
100 ng/ml TRAIL [h] - - 1 6 22 1 6 22
78 kDa
RIPK1
Precision Plus Protein™ All blue prestained protein Standards
kDa
250
150
100
75
50
37
25
20
15
10

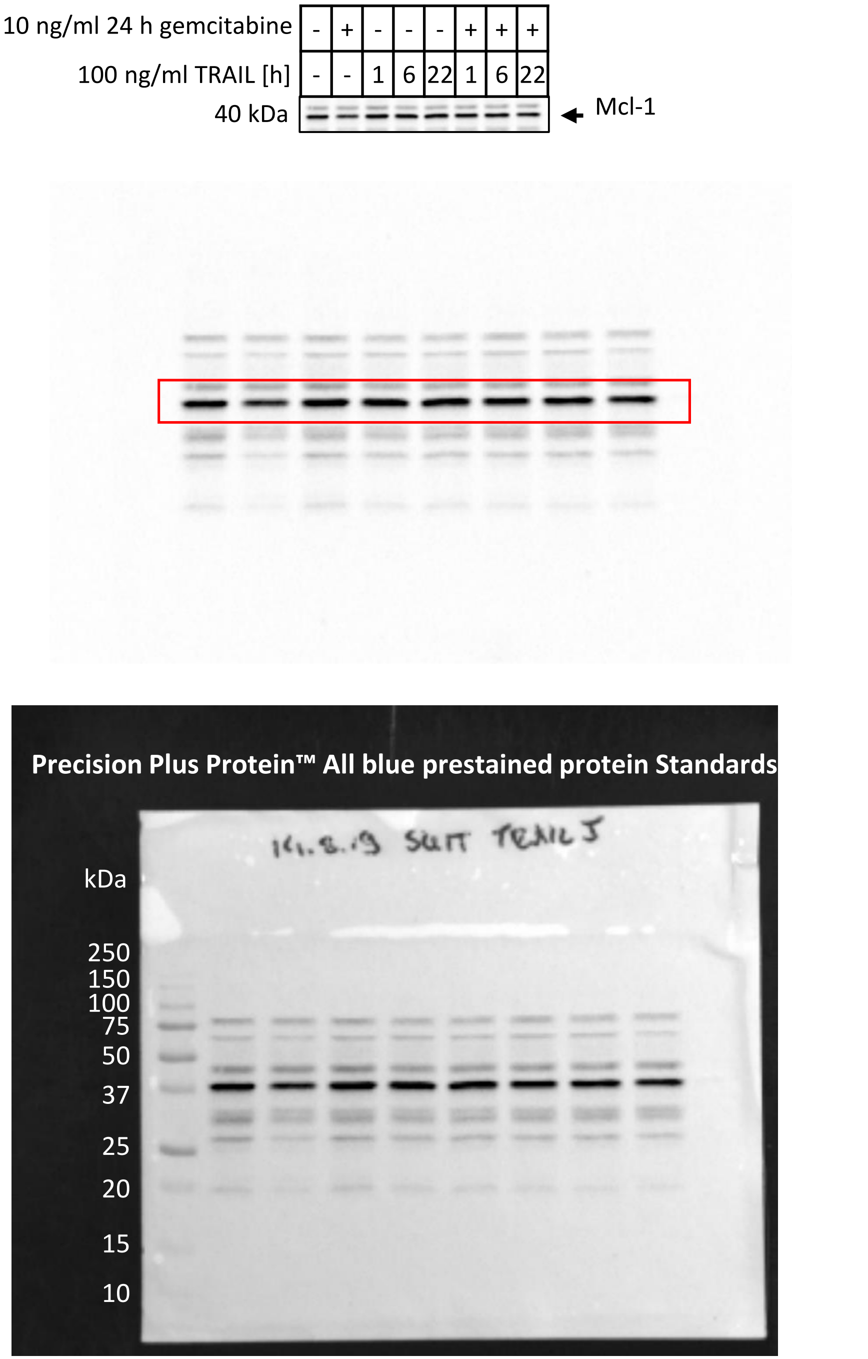
10 ng/ml 24 h gemcitabine - + - - - + + +
100 ng/ml TRAIL [h] - - 1 6 22 1 6 22
40 kDa
Mcl-1
Precision Plus Protein™ All blue prestained protein Standards
kDa
250
150
100
75
50
37
25
20
15
10

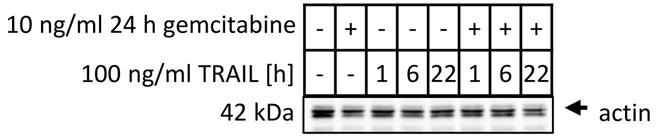
10 ng/ml 24 h gemcitabine
- + - - - + + +
100 ng/ml TRAIL [h]
- - 1 6 22 1 6 22
42 kDa
actin

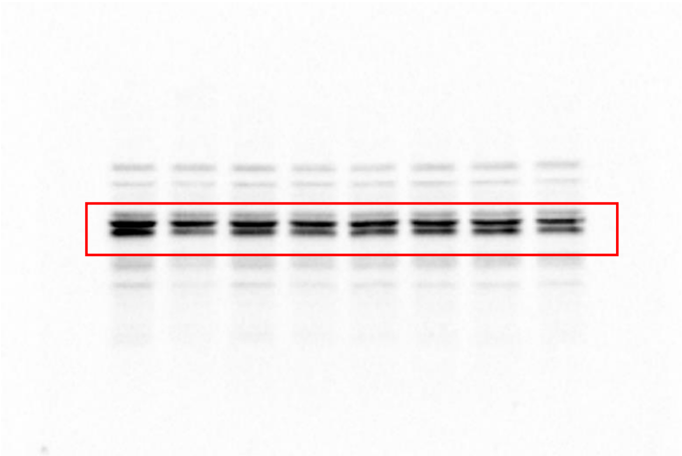

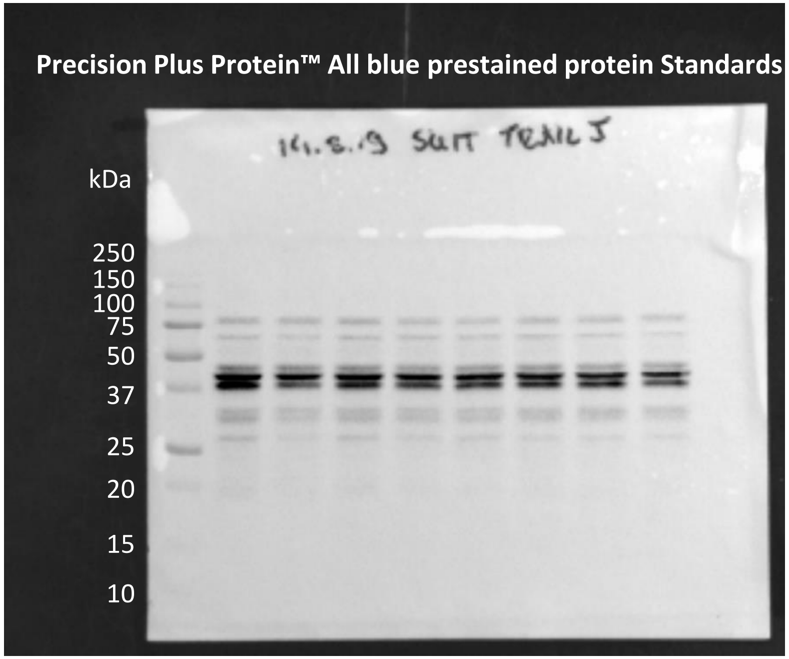
Precision Plus Protein™ All blue prestained protein Standards
kDa
250
150
100
75
50
37
25
20
15
10

e

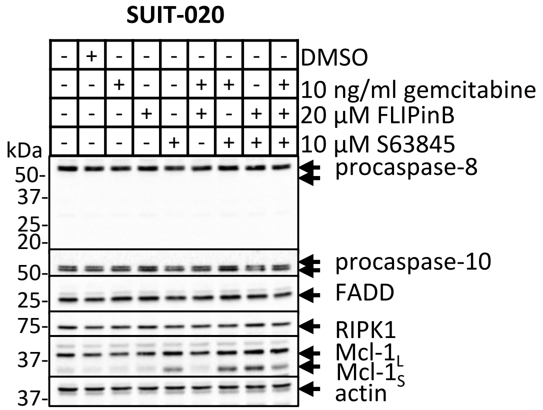
SUIT-020
DMSO
10 ng/ml gemcitabine
20 µM FLIPinB
10 µM S63845
kDa
procaspase-8
procaspase-10
FADD
RIPK1
Mcl-1L
Mcl-1S
actin

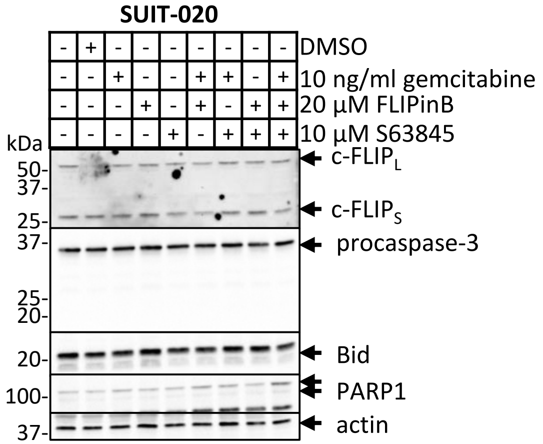
SUIT-020
DMSO
10 ng/ml gemcitabine
20 µM FLIPinB
10 µM S63845
kDa
c-FLIPL
c-FLIPS
procaspase-3
Bid
PARP1
actin

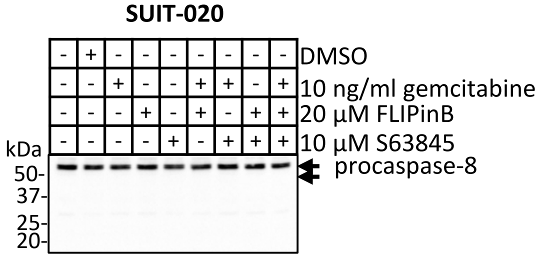
SUIT-020
DMSO
10 ng/ml gemcitabine
20 µM FLIPinB
10 µM S63845
kDa
50-
37-
25-
20-
procaspase-8

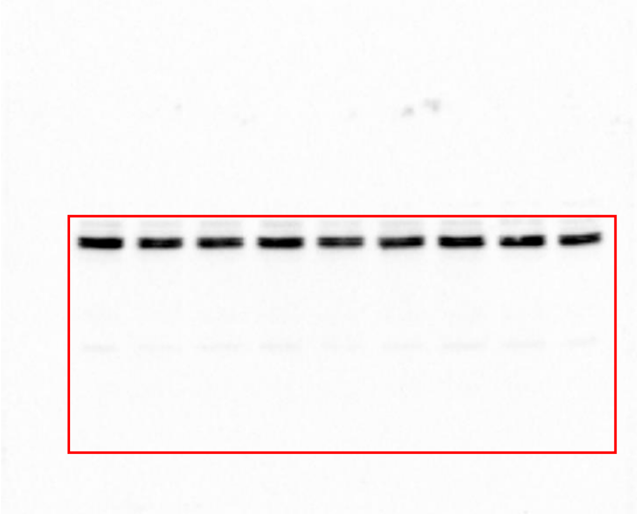

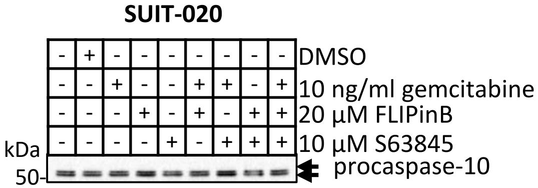
SUIT-020
DMSO
10 ng/ml gemcitabine
20 µM FLIPinB
10 µM S63845
kDa
50-
procaspase-10

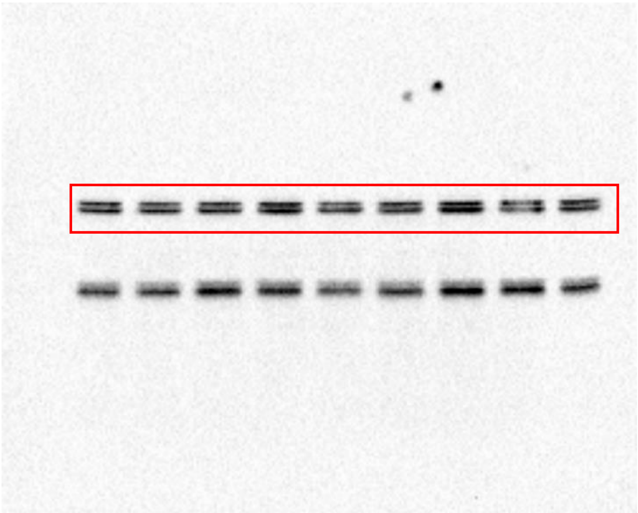

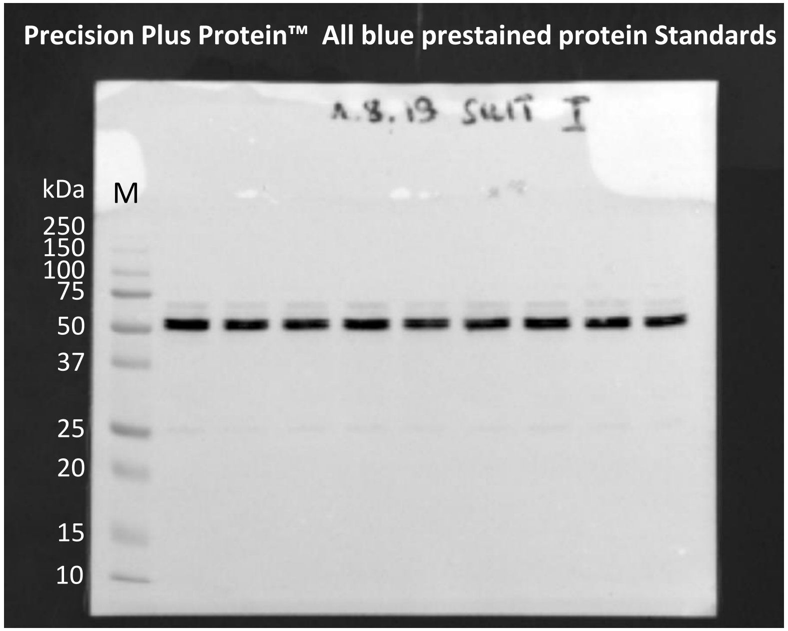
Precision Plus Protein™ All blue prestained protein Standards
kDa
M
250
150
100
75
50
37
25
20
15
10

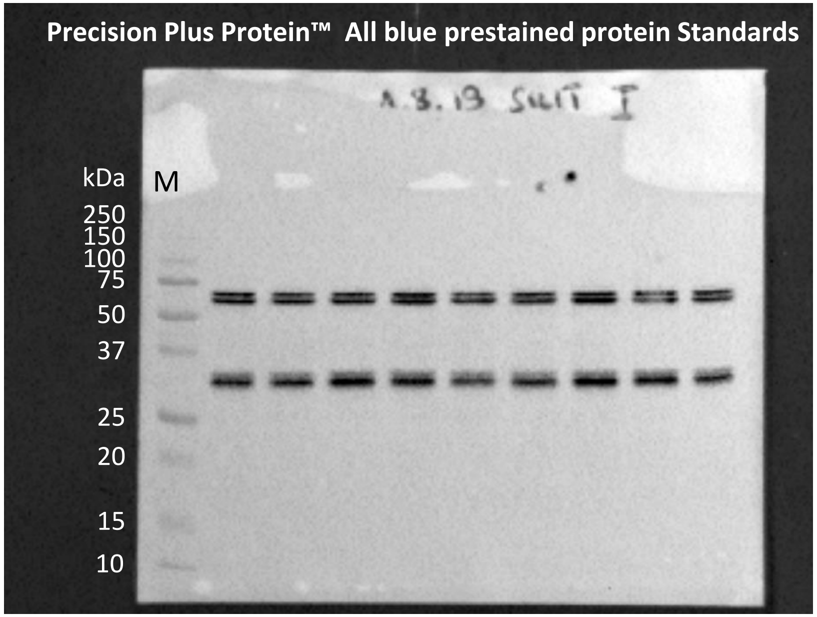
Precision Plus Protein™ All blue prestained protein Standards
kDa
M
250
150
100
75
50
37
25
20
15
10

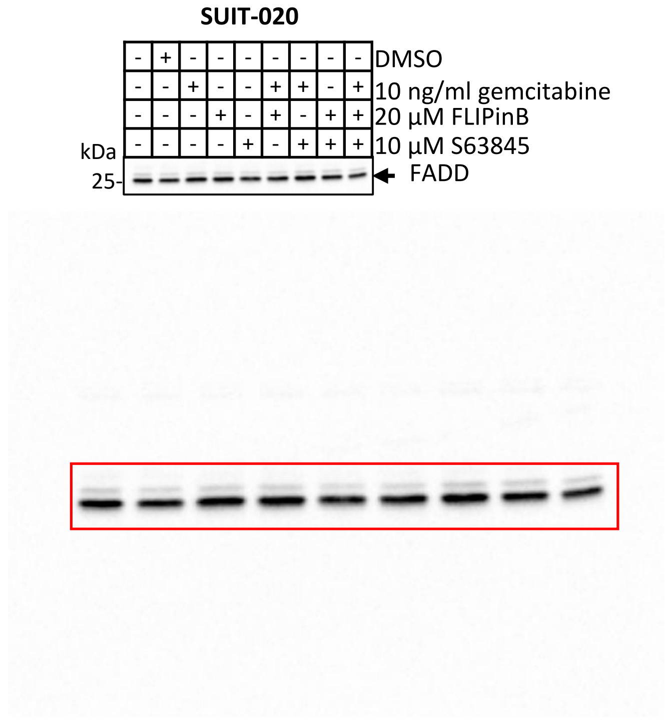
SUIT-020
DMSO: - + - - - - - - -
10 ng/ml gemcitabine: - - + - - + + - +
20 µM FLIPinB: - - - + - + - + +
10 µM S63845: - - - - + - + + +
kDa
25-
FADD

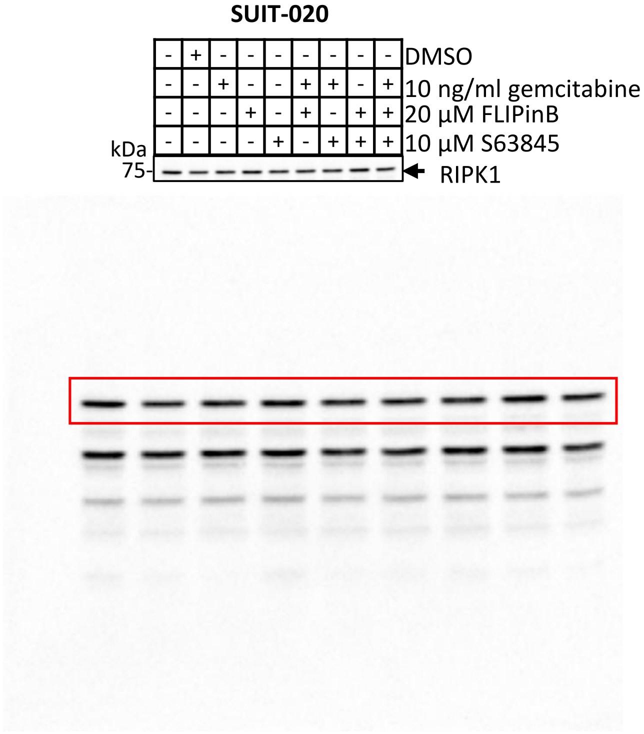
SUIT-020
DMSO: - + - - - - - - -
10 ng/ml gemcitabine: - - + - - + + - +
20 µM FLIPinB: - - - + - + - + +
10 µM S63845: - - - - + - + + +
kDa
75-
RIPK1

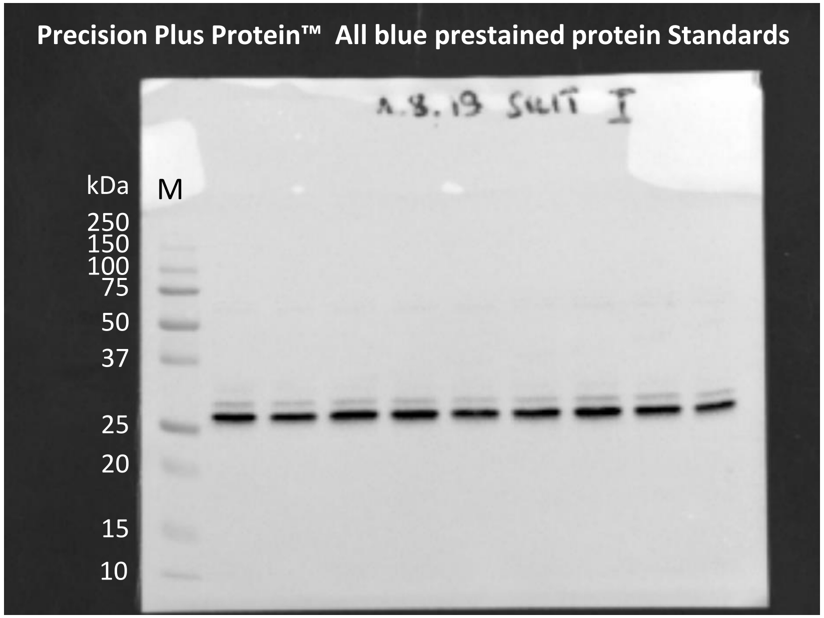
Precision Plus Protein™ All blue prestained protein Standards
kDa
M
250
150
100
75
50
37
25
20
15
10

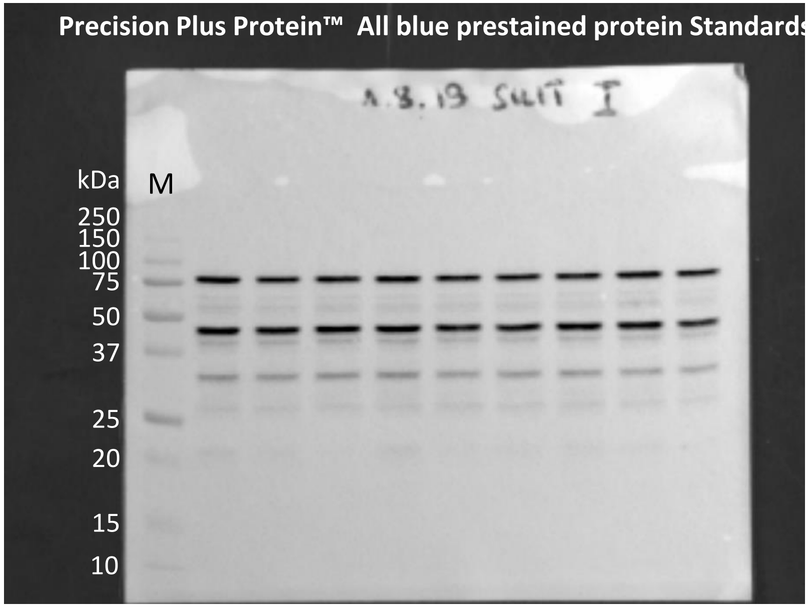
Precision Plus Protein™ All blue prestained protein Standards
kDa
M
250
150
100
75
50
37
25
20
15
10

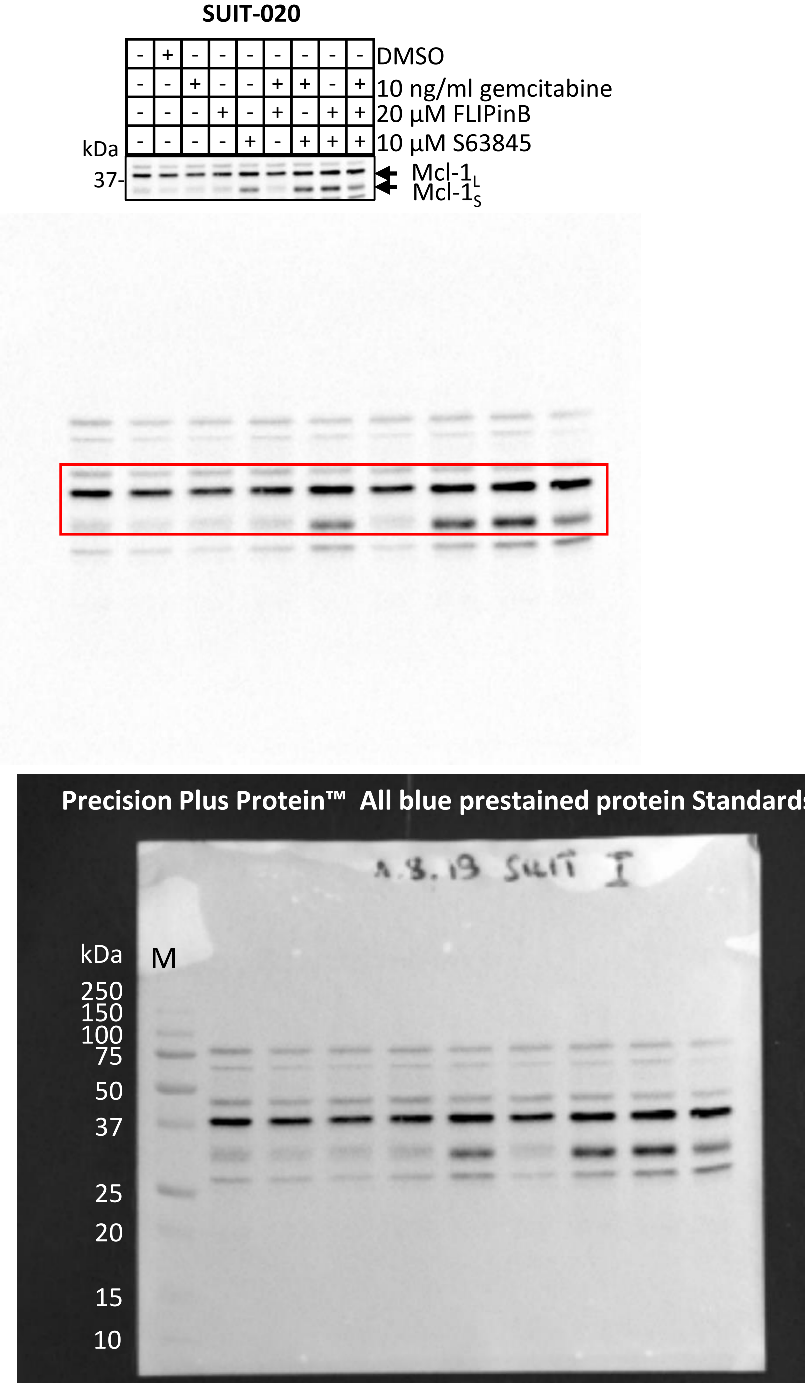
SUIT-020
- + - - - - - - - - DMSO
- - + - - + + - + 10 ng/ml gemcitabine
- - - + - + - + + 20 µM FLIPinB
- - - - + - + + + 10 µM S63845
kDa
37-
Mcl-1L
Mcl-1S
Precision Plus Protein™ All blue prestained protein Standards
kDa
M
250
150
100
75
50
37
25
20
15
10

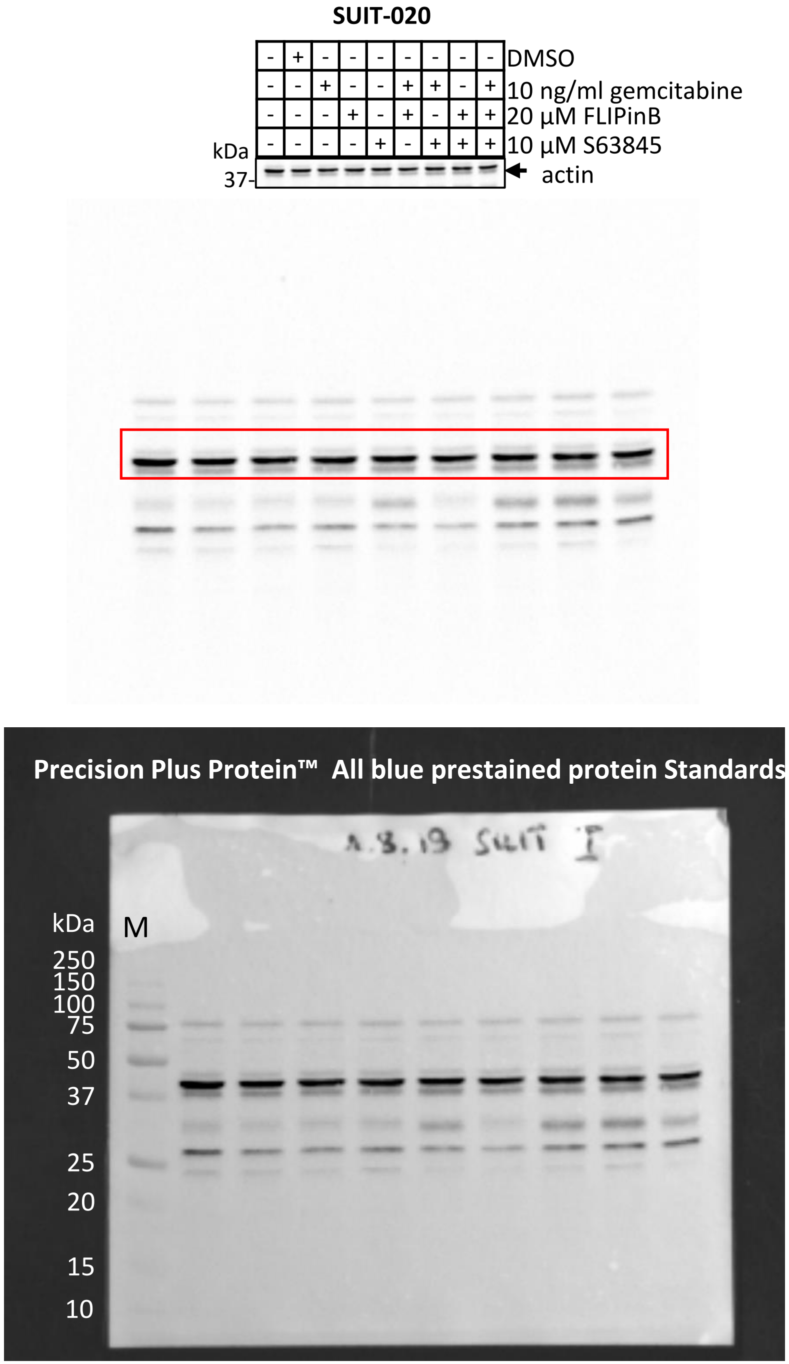
SUIT-020
- + - - - - - - - DMSO
- - + - - + + - + 10 ng/ml gemcitabine
- - - + - + - + + 20 µM FLIPinB
- - - - + - + + + 10 µM S63845
kDa
37-
actin
Precision Plus Protein™ All blue prestained protein Standards
kDa
M
250
150
100
75
50
37
25
20
15
10

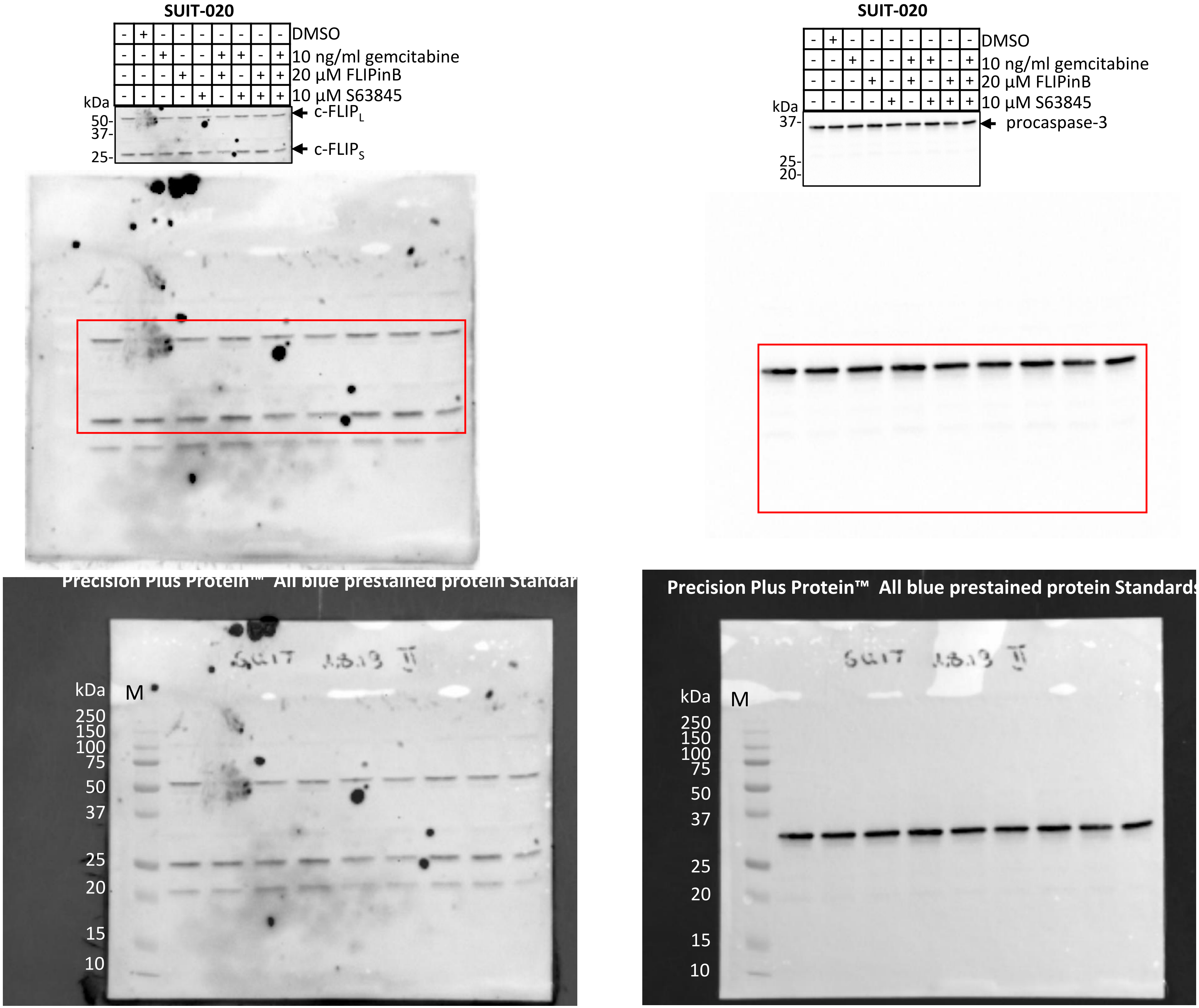
SUIT-020
DMSO
10 ng/ml gemcitabine
20 µM FLIPinB
10 µM S63845
kDa
50-
37-
25-
c-FLIPL
c-FLIPS
SUIT-020
DMSO
10 ng/ml gemcitabine
20 µM FLIPinB
10 µM S63845
kDa
37-
25-
20-
procaspase-3
Precision Plus Protein™ All blue prestained protein Standards
kDa
M
250
150
100
75
50
37
25
20
15
10
Precision Plus Protein™ All blue prestained protein Standards
kDa
M
250
150
100
75
50
37
25
20
15
10

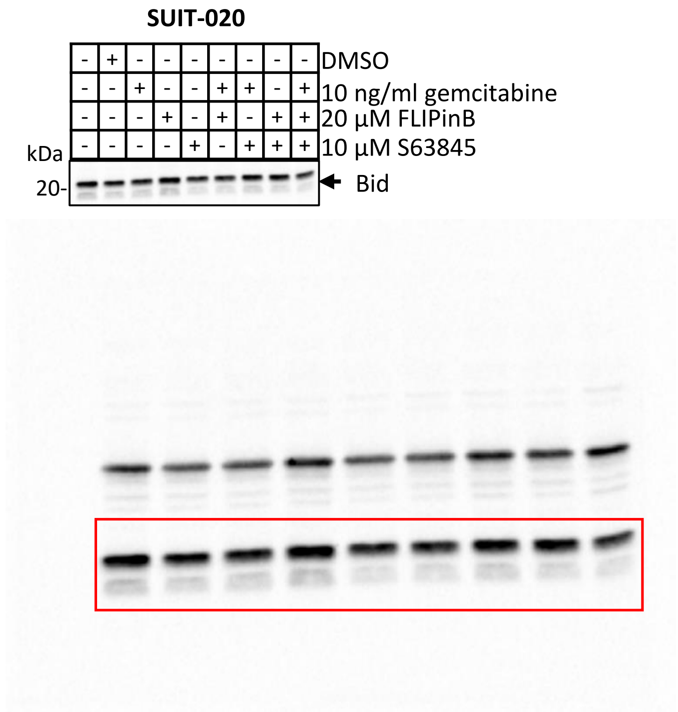
SUIT-020
DMSO
10 ng/ml gemcitabine
20 µM FLIPinB
10 µM S63845
kDa
20-
Bid

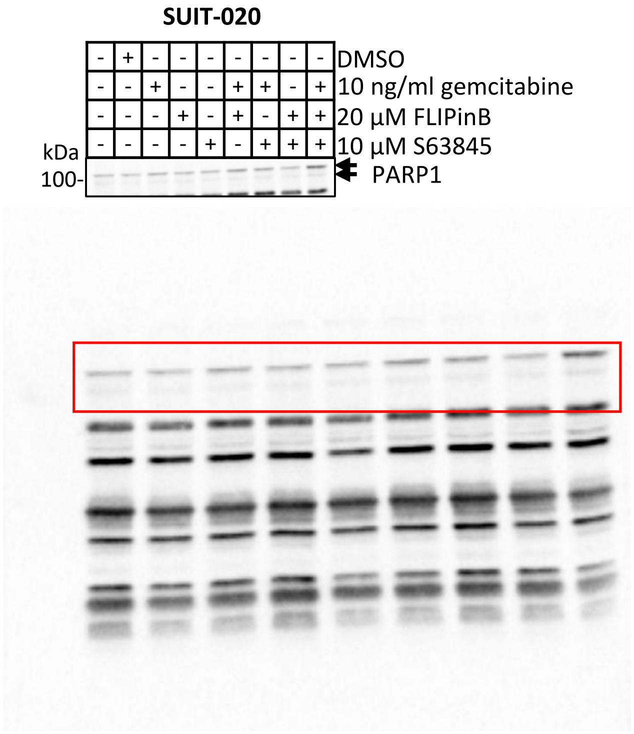
SUIT-020
DMSO
10 ng/ml gemcitabine
20 µM FLIPinB
10 µM S63845
kDa
100-
PARP1

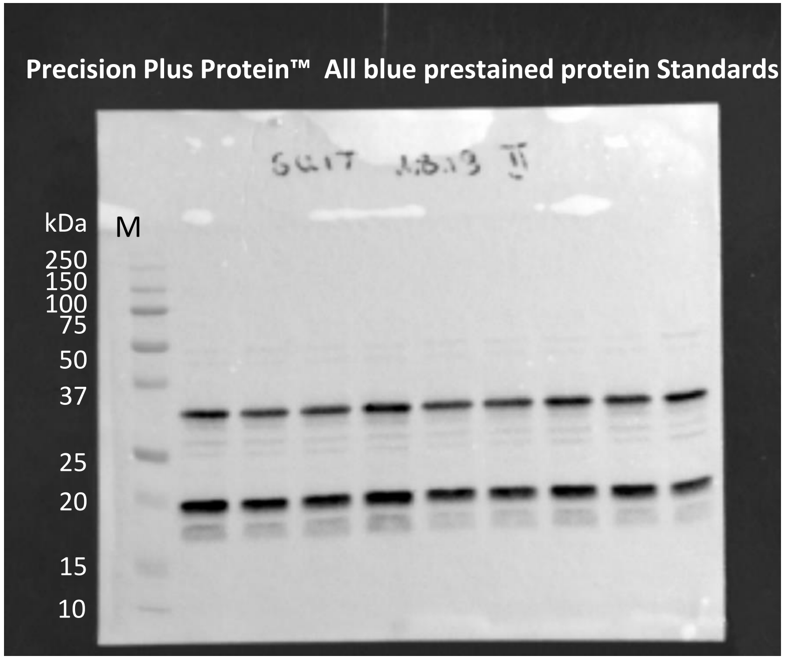
Precision Plus Protein™ All blue prestained protein Standards
kDa
M
250
150
100
75
50
37
25
20
15
10

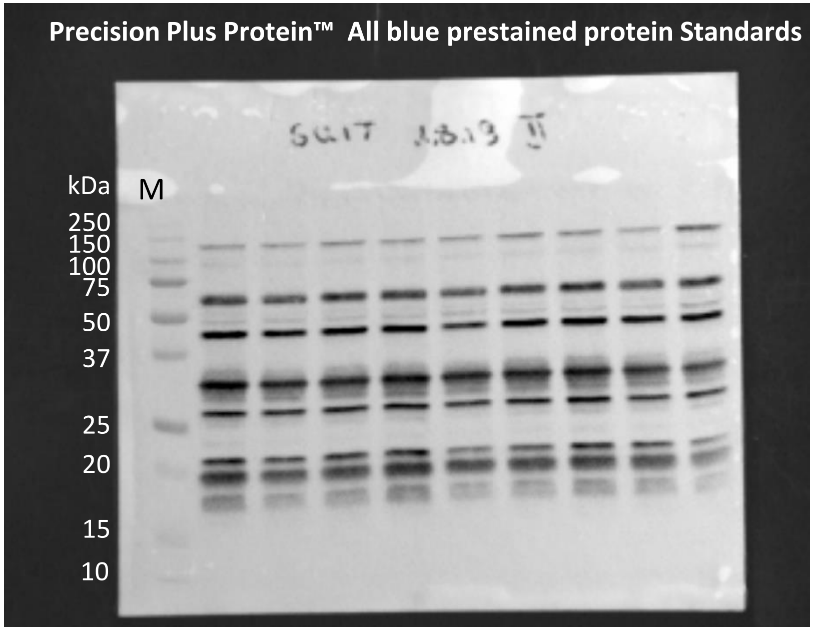
Precision Plus Protein™ All blue prestained protein Standards
kDa
M
250
150
100
75
50
37
25
20
15
10

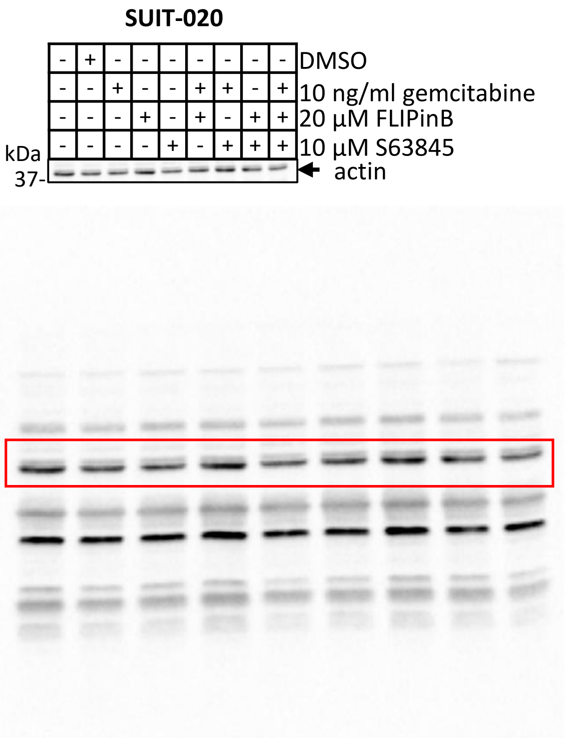
SUIT-020
DMSO: - + - - - - - - -
10 ng/ml gemcitabine: - - + - - + + - +
20 µM FLIPinB: - - - + - + - + +
10 µM S63845: - - - - + - + + +
kDa
37-
actin

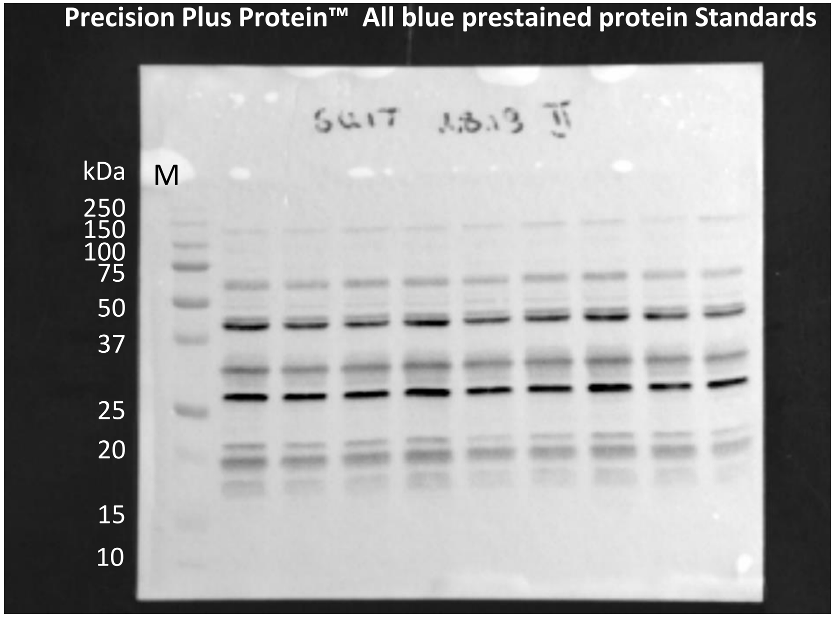
Precision Plus Protein™ All blue prestained protein Standards
kDa
M
250
150
100
75
50
37
25
20
15
10

d

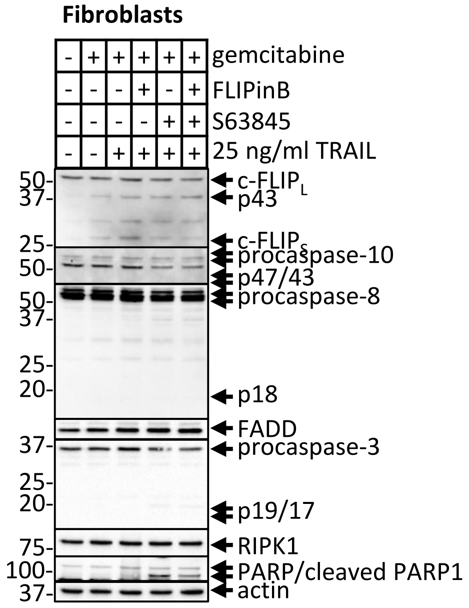
Fibroblasts
gemcitabine
FLIPinB
S63845
25 ng/ml TRAIL
c-FLIP$_L$
p43
c-FLIP$_S$
procaspase-10
p47/43
procaspase-8
p18
FADD
procaspase-3
p19/17
RIPK1
PARP/cleaved PARP1
actin
50-
37-
25-
50-
50-
37-
25-
20-
37-
25-
20-
75-
100-
37-

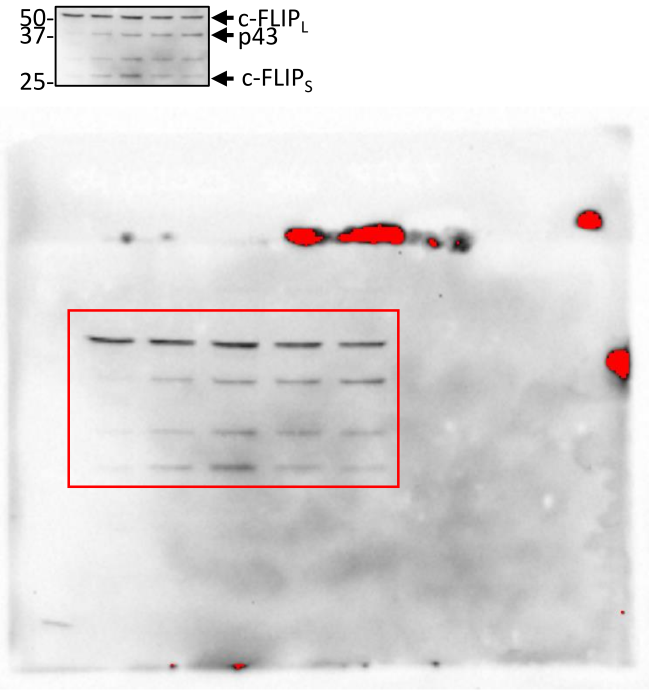

50-
37-
25-
c-FLIP$_L$
p43
c-FLIP$_S$

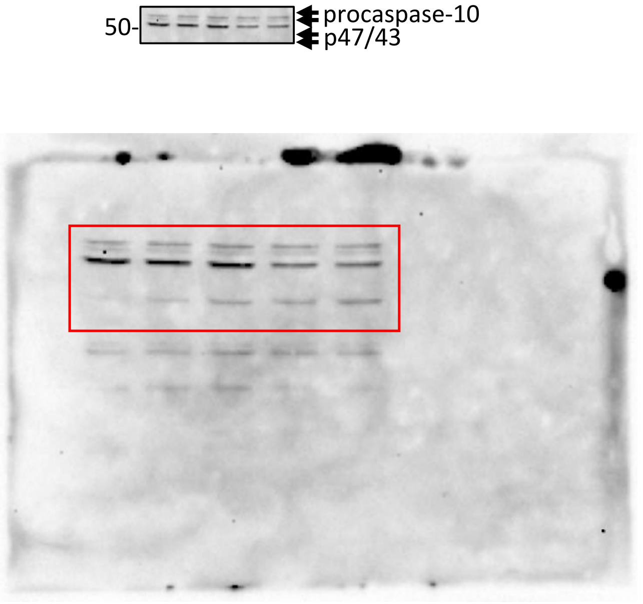

50-
procaspase-10
p47/43

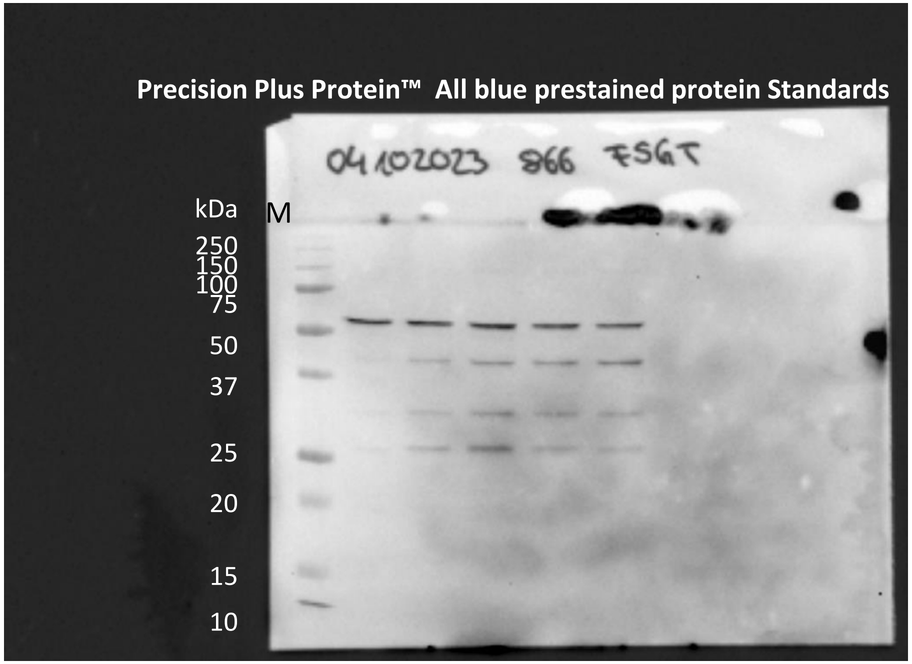

Precision Plus Protein™ All blue prestained protein Standards
kDa
M
250
150
100
75
50
37
25
20
15
10

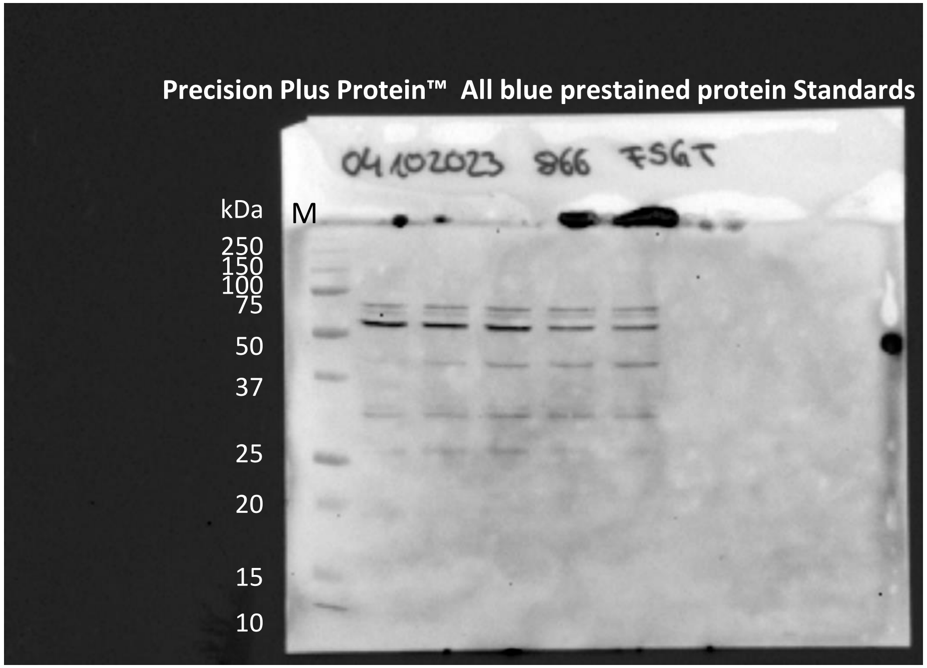

Precision Plus Protein™ All blue prestained protein Standards
kDa
M
250
150
100
75
50
37
25
20
15
10

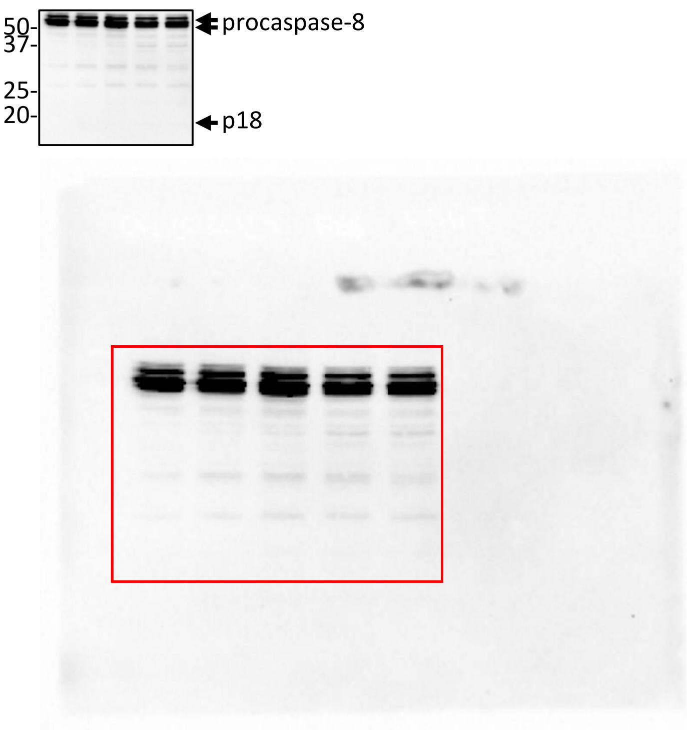

50-
37-
25-
20-
procaspase-8
p18

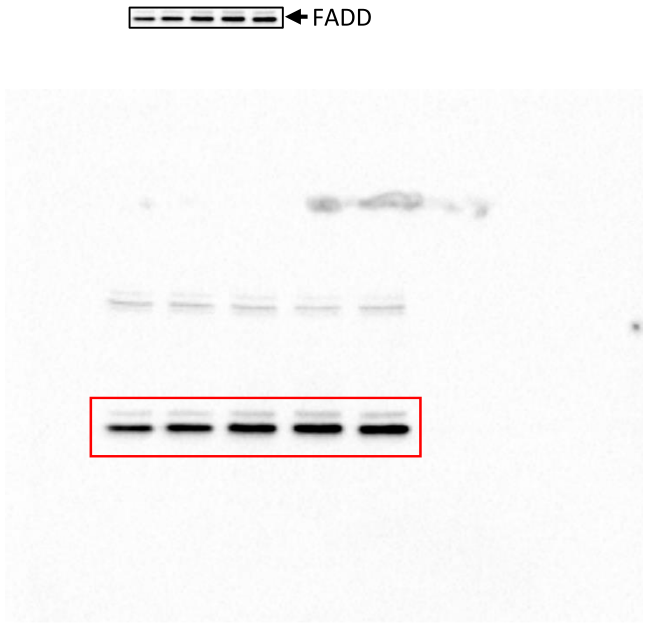

FADD

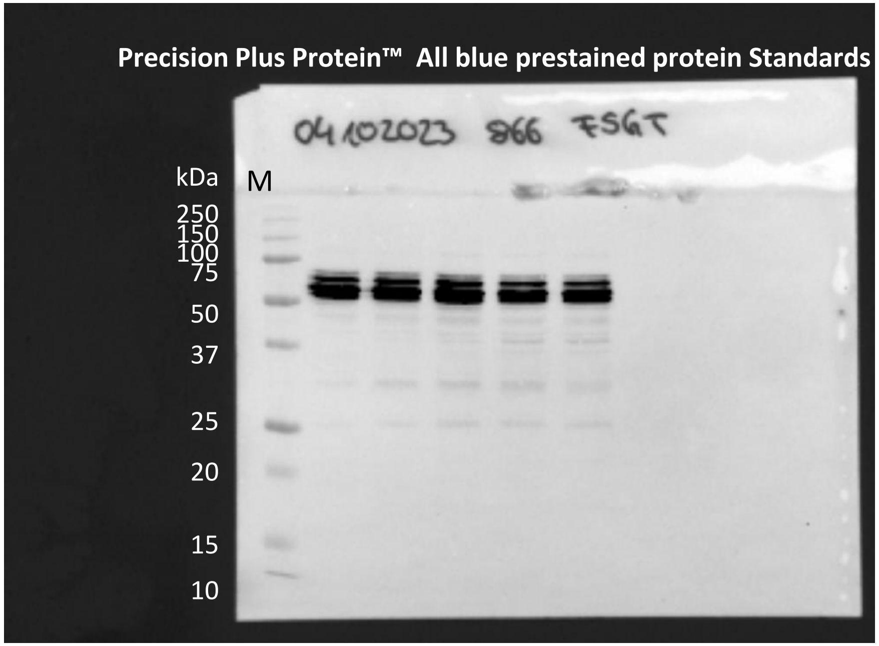

Precision Plus Protein™ All blue prestained protein Standards
kDa
M
250
150
100
75
50
37
25
20
15
10

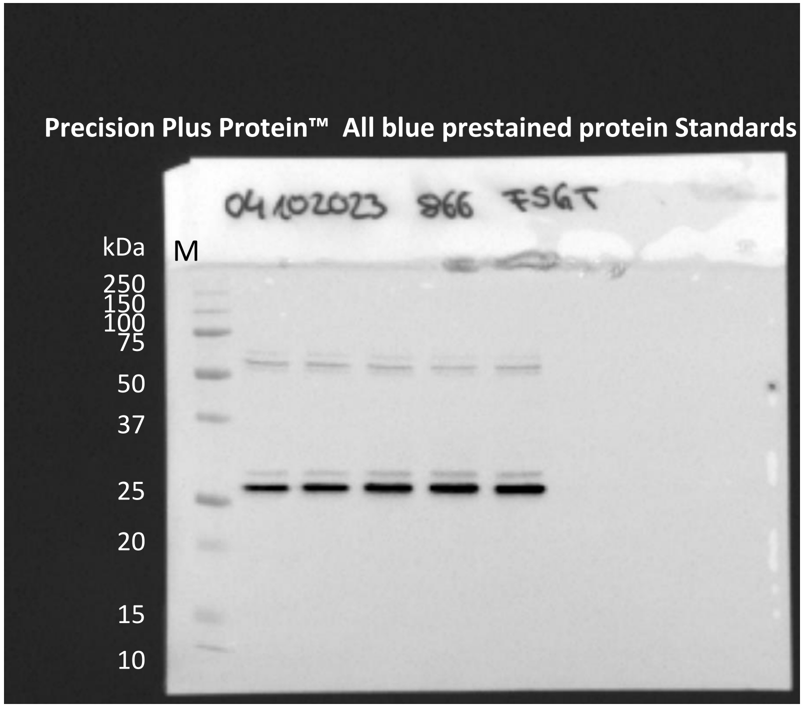

Precision Plus Protein™ All blue prestained protein Standards
kDa
M
250
150
100
75
50
37
25
20
15
10

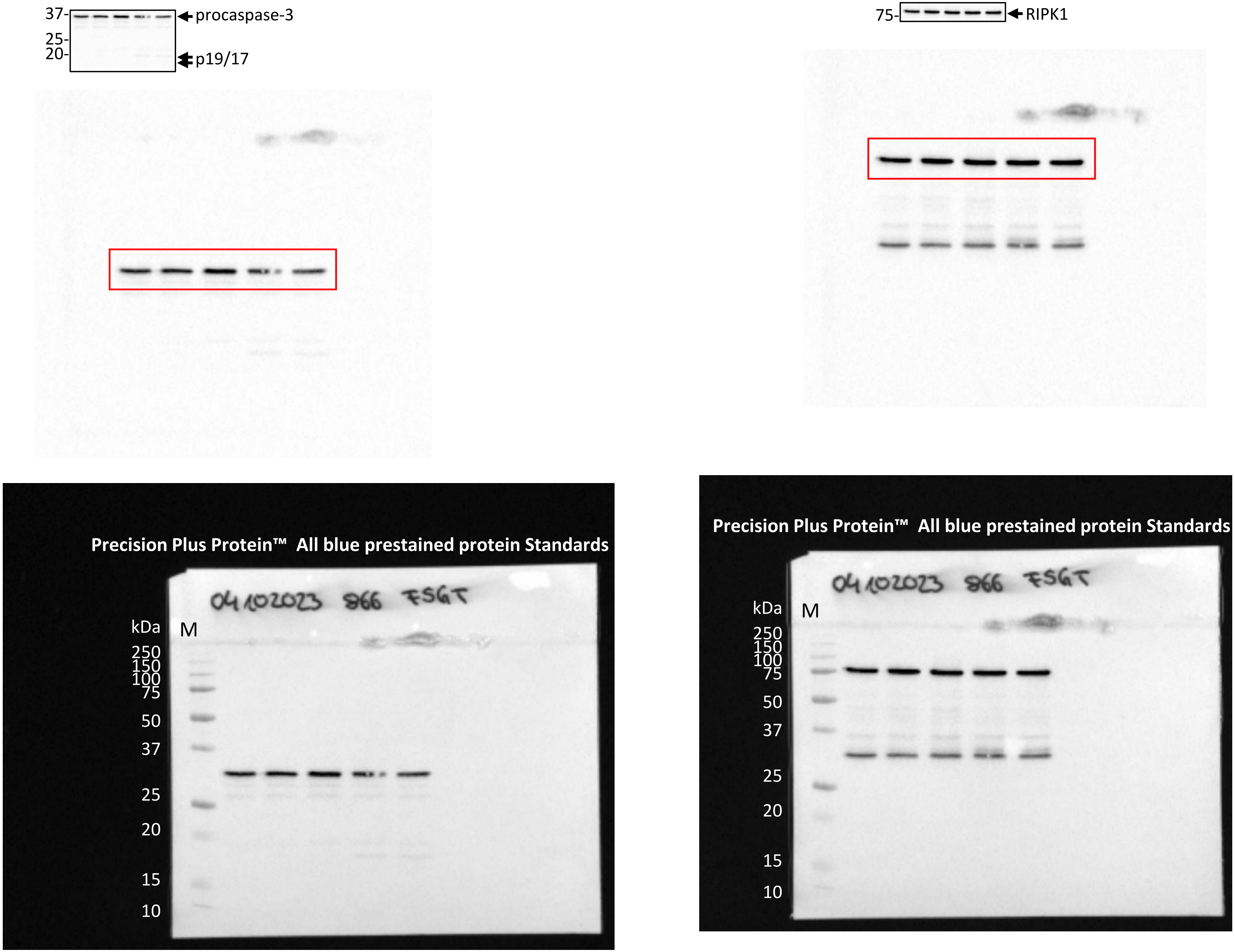
37-
procaspase-3
25-
20-
p19/17
75-
RIPK1
Precision Plus Protein™ All blue prestained protein Standards
kDa
M
250
150
100
75
50
37
25
20
15
10
Precision Plus Protein™ All blue prestained protein Standards
kDa
M
250
150
100
75
50
37
25
20
15
10

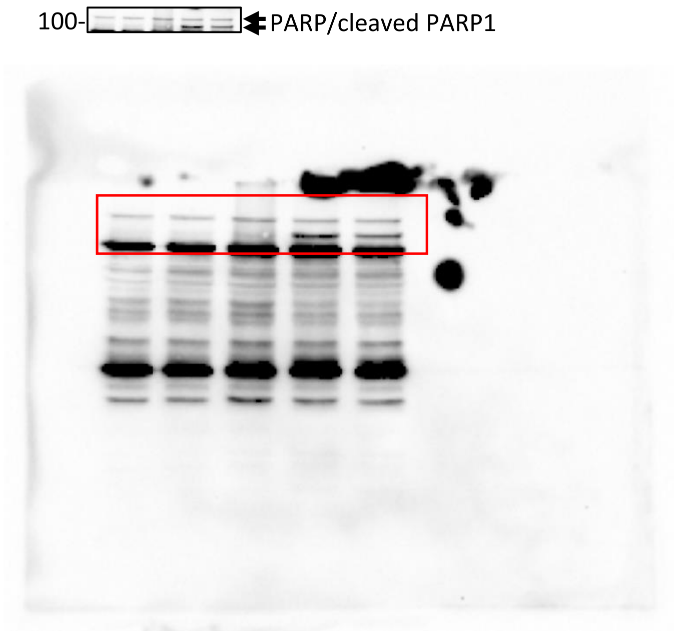
100-
PARP/cleaved PARP1

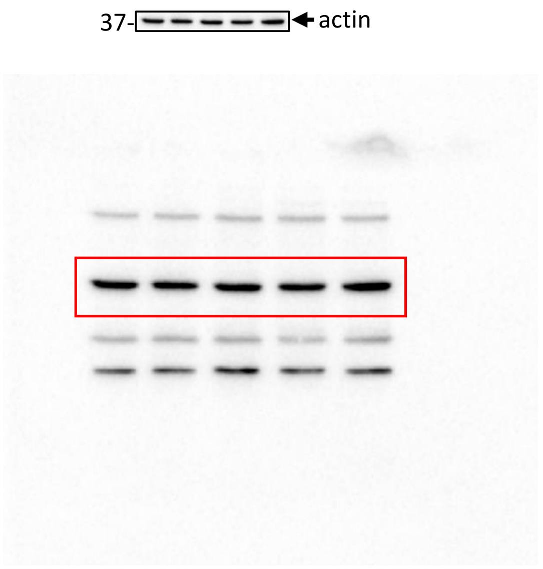
37-
actin

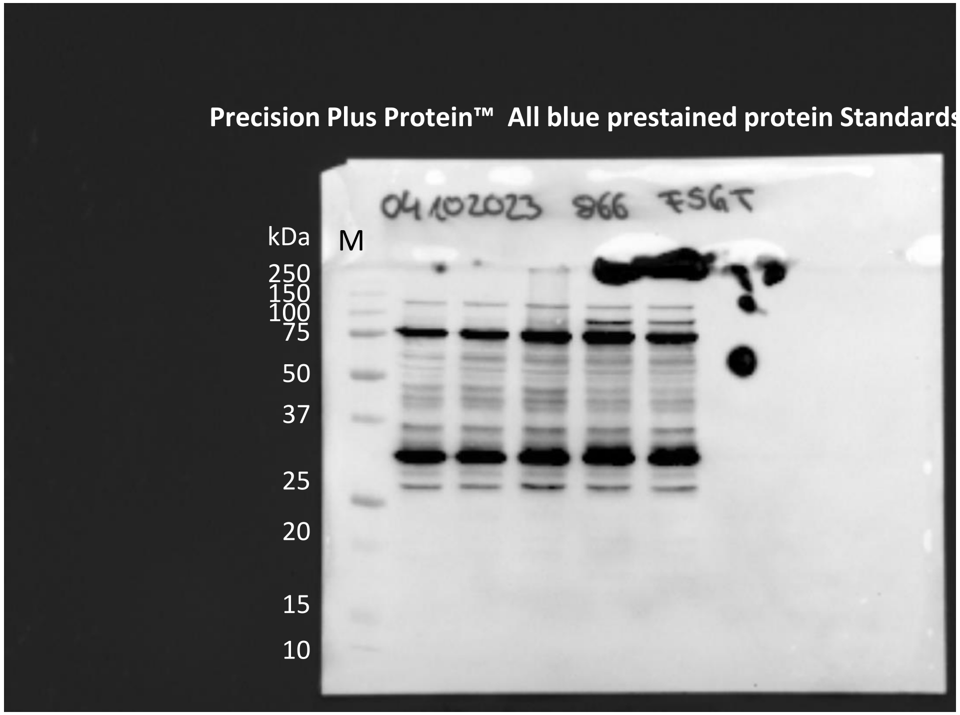
Precision Plus Protein™ All blue prestained protein Standards
kDa
M
250
150
100
75
50
37
25
20
15
10

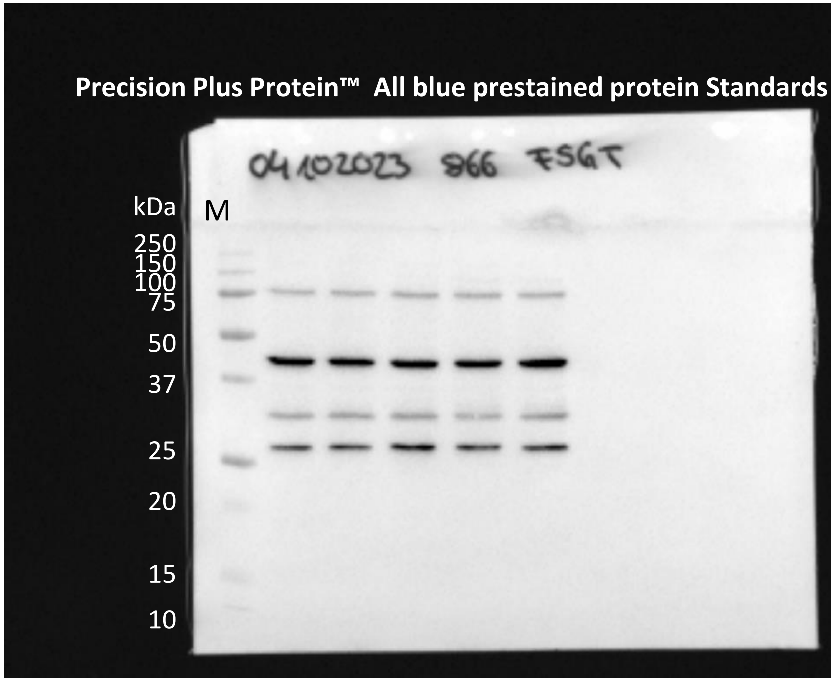
Precision Plus Protein™ All blue prestained protein Standards
kDa
M
250
150
100
75
50
37
25
20
15
10

a

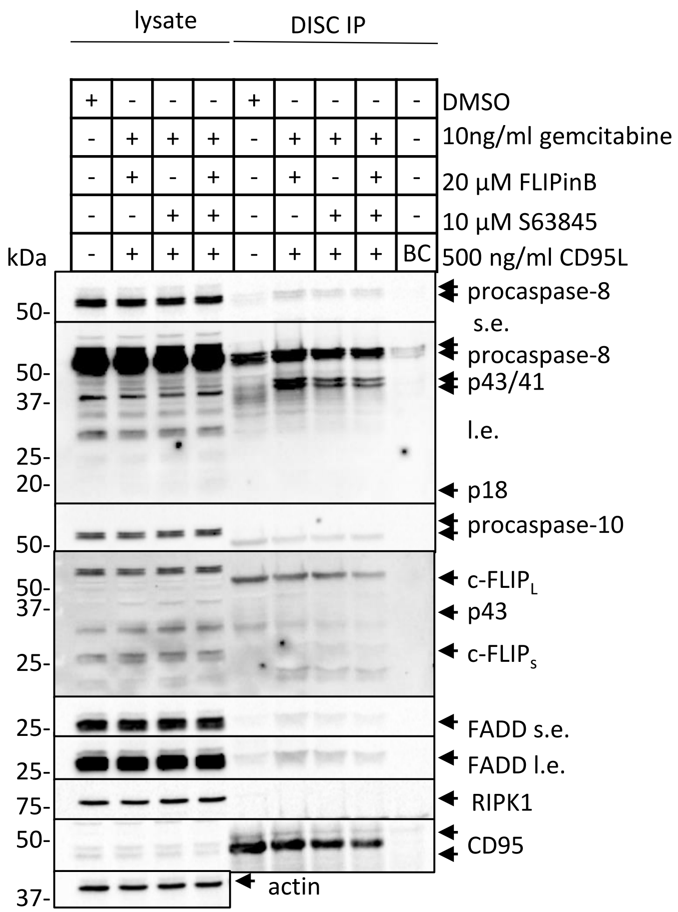
lysate
DISC IP
DMSO
10ng/ml gemcitabine
20 µM FLIPinB
10 µM S63845
500 ng/ml CD95L
BC
kDa
procaspase-8
s.e.
procaspase-8
p43/41
l.e.
p18
procaspase-10
c-FLIP$_L$
p43
c-FLIP$_S$
FADD s.e.
FADD l.e.
RIPK1
CD95
actin

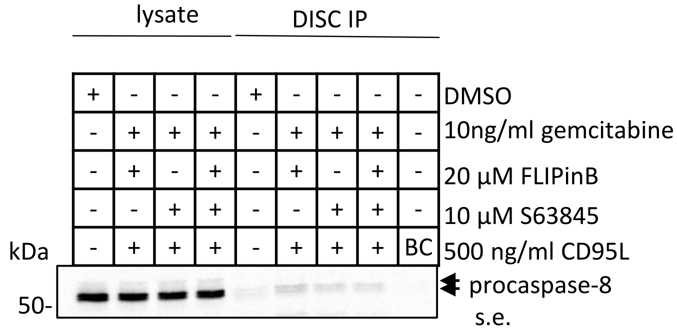
lysate
DISC IP
DMSO
10ng/ml gemcitabine
20 µM FLIPinB
10 µM S63845
500 ng/ml CD95L
kDa
50-
BC
procaspase-8
s.e.

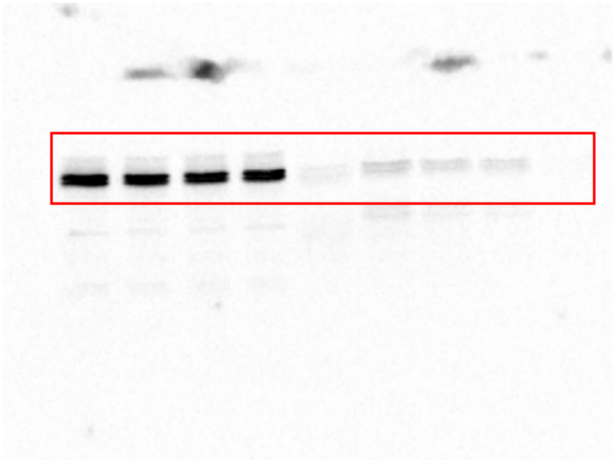

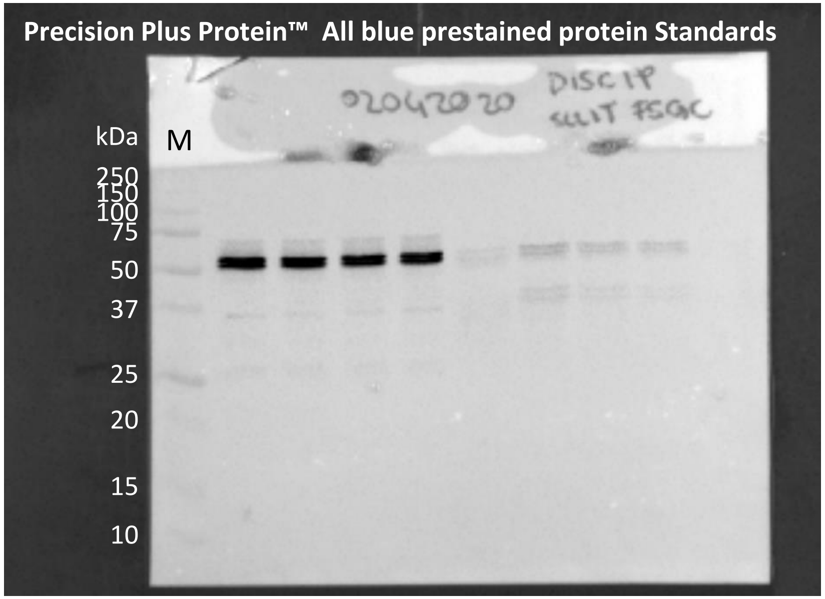
Precision Plus Protein™ All blue prestained protein Standards
kDa
M
250
150
100
75
50
37
25
20
15
10

lysate				DISC IP					
+	-	-	-	+	-	-	-	-	DMSO
-	+	+	+	-	+	+	+	-	10ng/ml gemcitabine
-	+	-	+	-	+	-	+	-	20 µM FLIPinB
-	-	+	+	-	-	+	+	-	10 µM S63845
-	+	+	+	-	+	+	+	BC	500 ng/ml CD95L

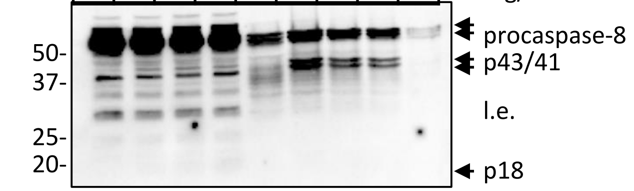

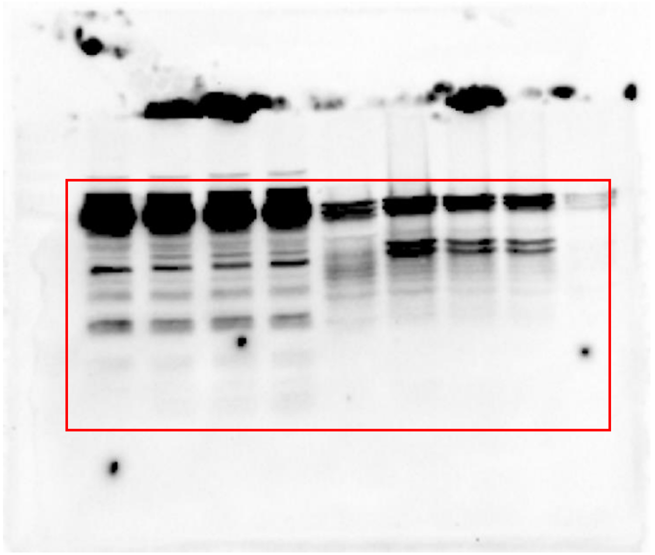

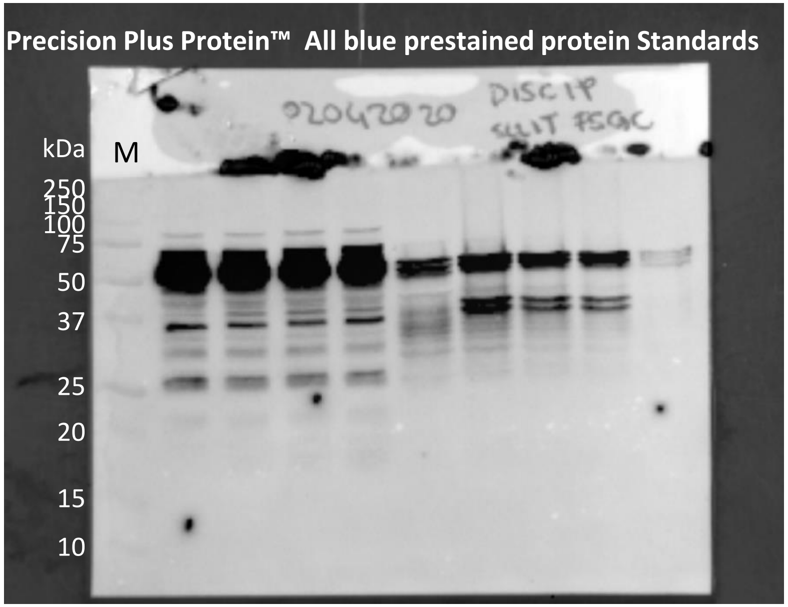

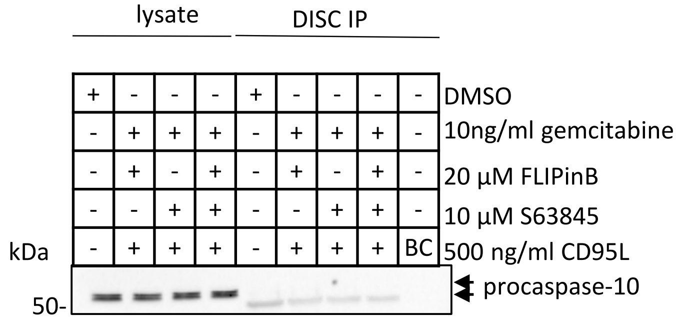
lysate
DISC IP
+ - - - + - - - - DMSO
- + + + - + + + - 10ng/ml gemcitabine
- + - + - + - + - 20 µM FLIPinB
- - + + - - + + - 10 µM S63845
kDa - + + + - + + + BC 500 ng/ml CD95L
procaspase-10
50-

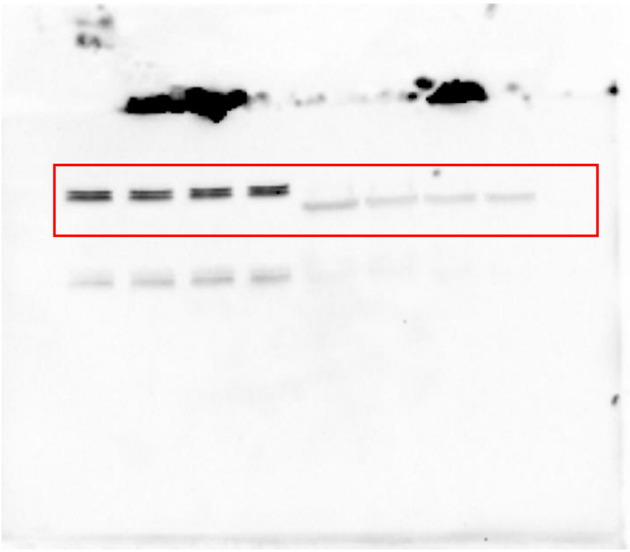

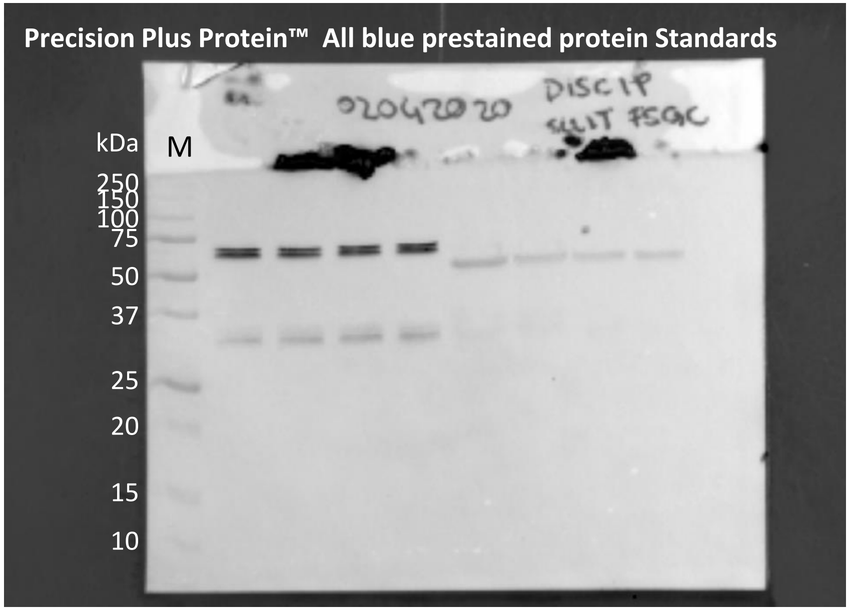
Precision Plus Protein™ All blue prestained protein Standards
kDa
M
250
150
100
75
50
37
25
20
15
10

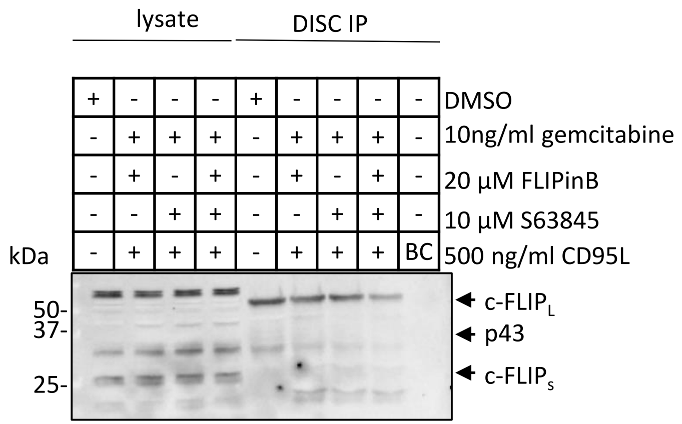
lysate
DISC IP
+ - - - + - - - - DMSO
- + + + - + + + - 10ng/ml gemcitabine
- + - + - + - + - 20 µM FLIPinB
- - + + - - + + - 10 µM S63845
- + + + - + + + BC 500 ng/ml CD95L
kDa
50-
37-
25-
c-FLIPL
p43
c-FLIPS

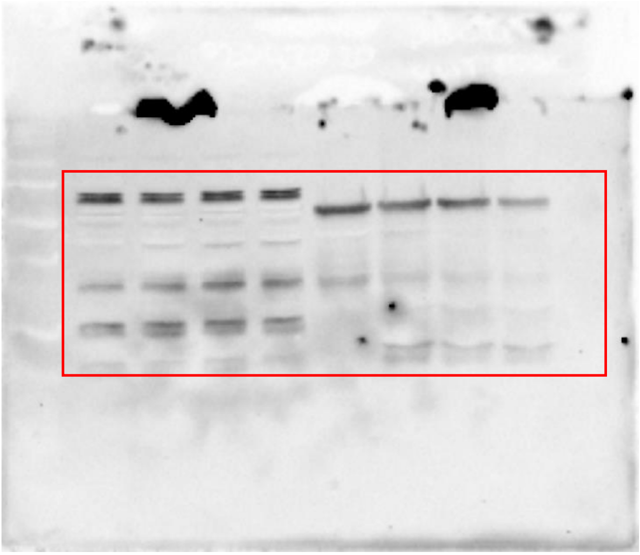

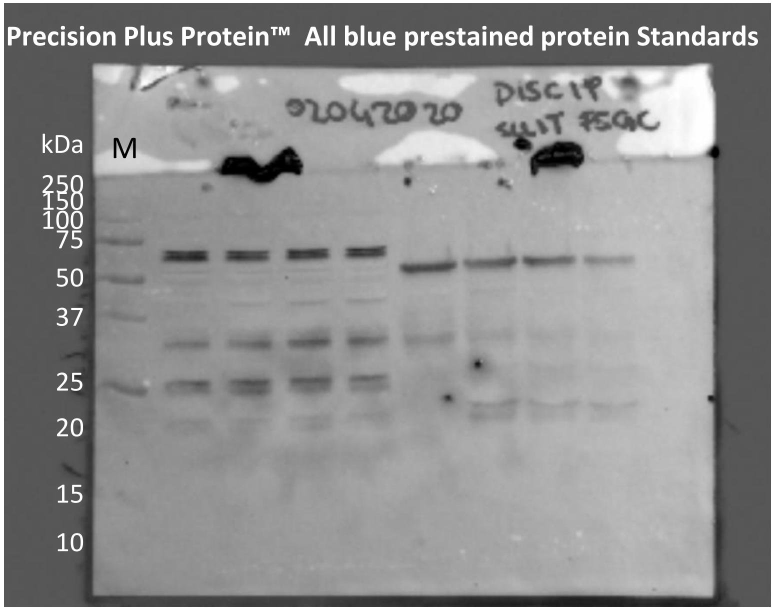
Precision Plus Protein™ All blue prestained protein Standards
kDa
M
250
150
100
75
50
37
25
20
15
10

lysate				DISC IP					
+	-	-	-	+	-	-	-	-	DMSO
-	+	+	+	-	+	+	+	-	10ng/ml gemcitabine
-	+	-	+	-	+	-	+	-	20 µM FLIPinB
-	-	+	+	-	-	+	+	-	10 µM S63845
-	+	+	+	-	+	+	+	BC	500 ng/ml CD95L

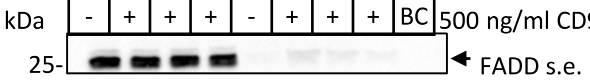

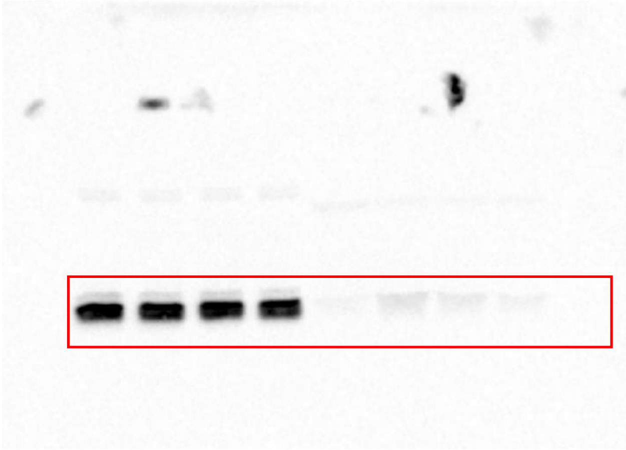

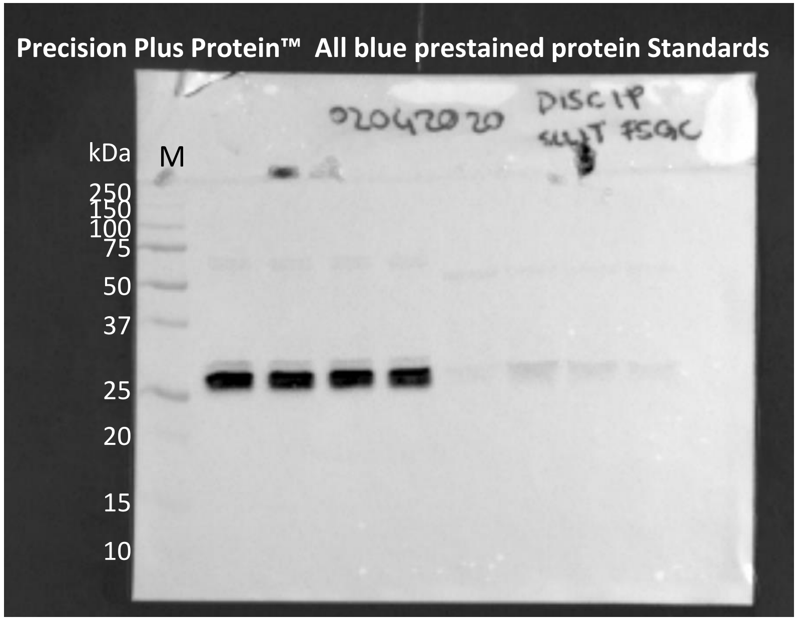

lysate				DISC IP					
+	-	-	-	+	-	-	-	-	DMSO
-	+	+	+	-	+	+	+	-	10ng/ml gemcitabine
-	+	-	+	-	+	-	+	-	20 µM FLIPinB
-	-	+	+	-	-	+	+	-	10 µM S63845
-	+	+	+	-	+	+	+	BC	500 ng/ml CD95L

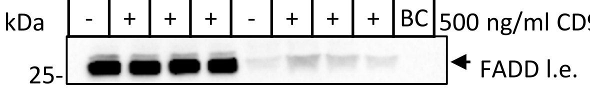

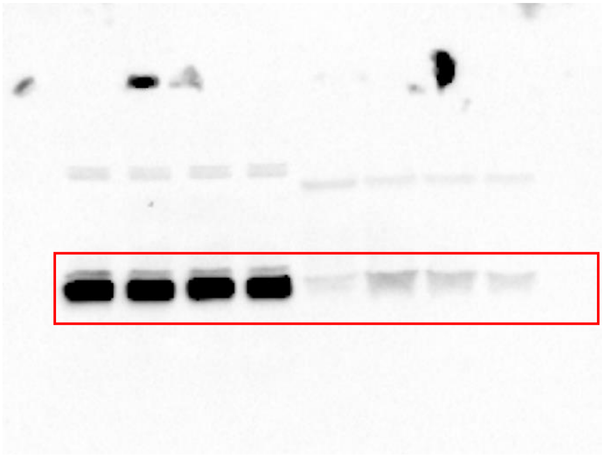

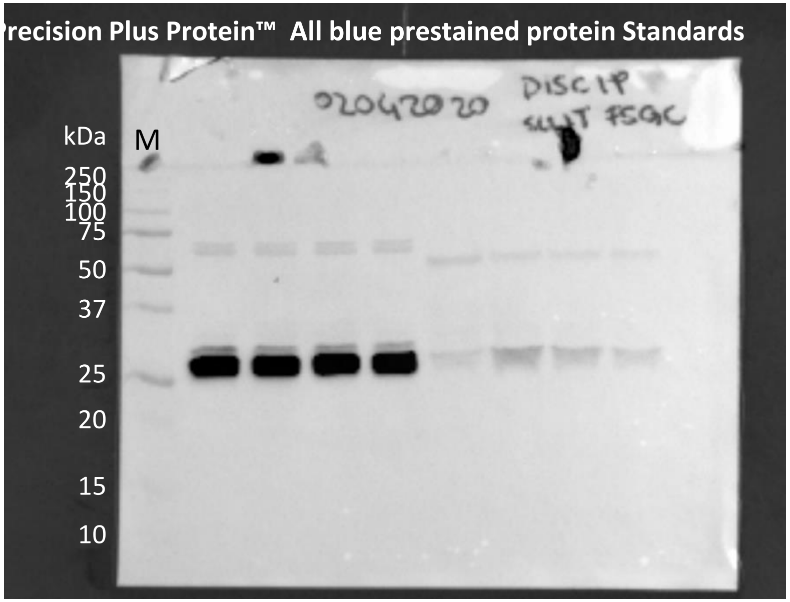

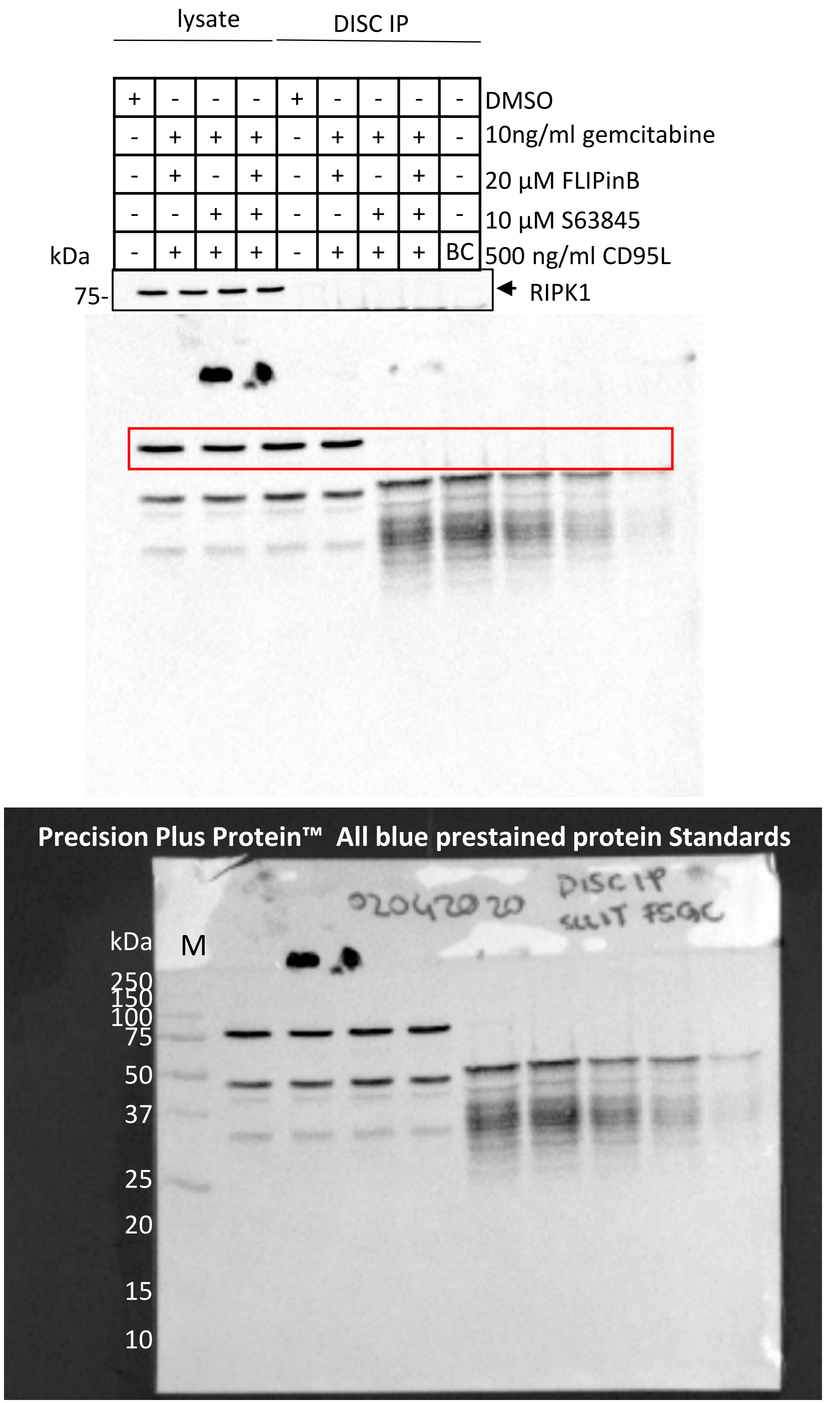
lysate
DISC IP
DMSO: + - - - + - - - -
10ng/ml gemcitabine: - + + + - + + + -
20 µM FLIPinB: - + - + - + - + -
10 µM S63845: - - + + - - + + -
500 ng/ml CD95L: - + + + - + + + BC
kDa
75-
RIPK1
Precision Plus Protein™ All blue prestained protein Standards
kDa
M
250
150
100
75
50
37
25
20
15
10

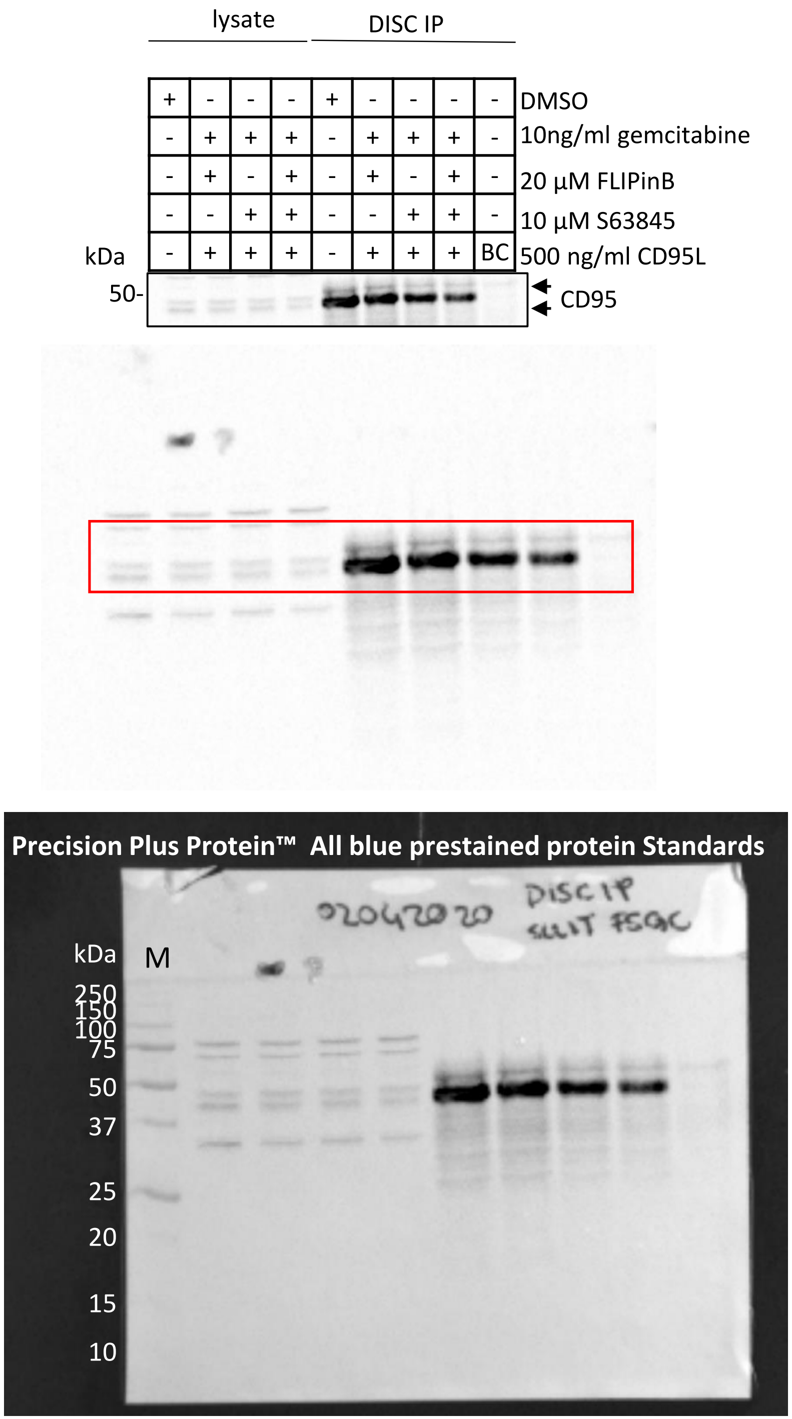
lysate
DISC IP
DMSO: + - - - + - - - -
10ng/ml gemcitabine: - + + + - + + + -
20 µM FLIPinB: - + - + - + - + -
10 µM S63845: - - + + - - + + -
500 ng/ml CD95L: - + + + - + + + BC
kDa
50-
CD95
Precision Plus Protein™ All blue prestained protein Standards
kDa
M
250
150
100
75
50
37
25
20
15
10

lysate				DISC IP					
+	-	-	-	+	-	-	-	-	DMSO
-	+	+	+	-	+	+	+	-	10ng/ml gemcitabine
-	+	-	+	-	+	-	+	-	20 µM FLIPinB
-	-	+	+	-	-	+	+	-	10 µM S63845
-	+	+	+	-	+	+	+	BC	500 ng/ml CD95L

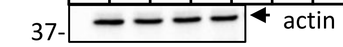

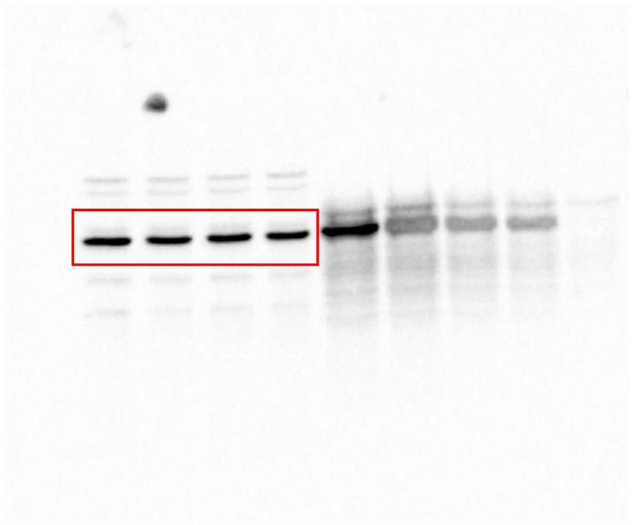

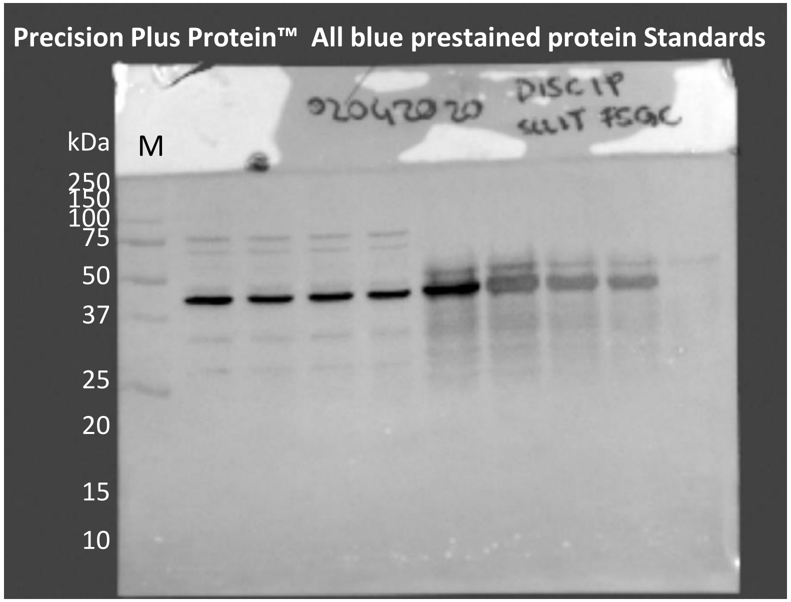

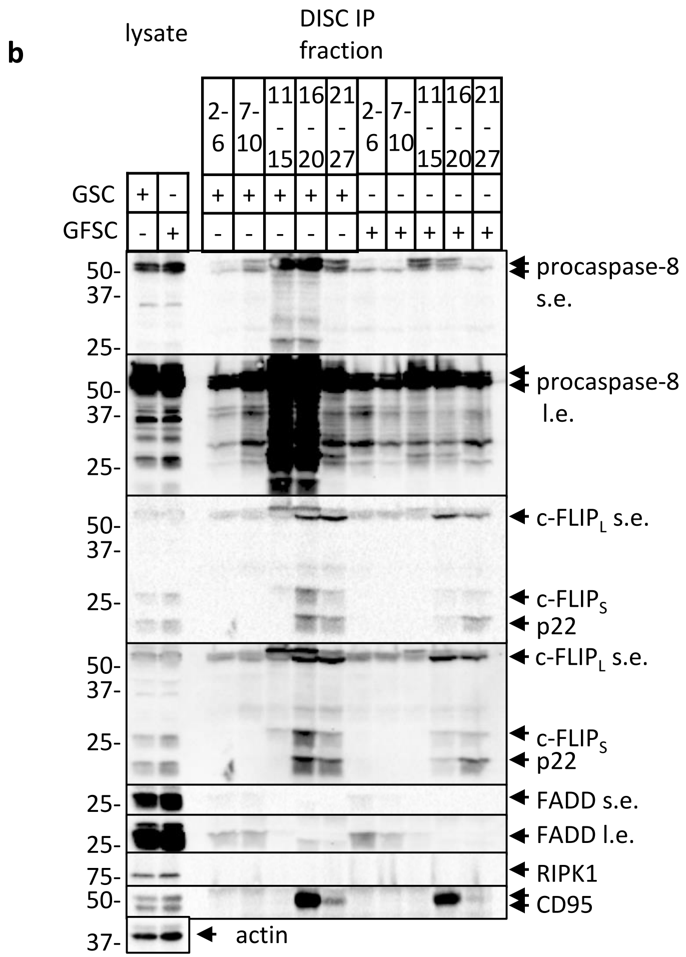
b
lysate
DISC IP fraction
2-6
7-10
11-15
16-20
21-27
GSC
GFSC
50-
37-
25-
75-
procaspase-8 s.e.
procaspase-8 l.e.
c-FLIP$_L$ s.e.
c-FLIP$_S$
p22
FADD s.e.
FADD l.e.
RIPK1
CD95
actin

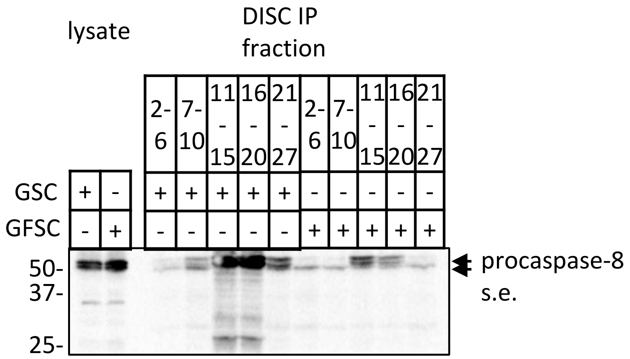
lysate
DISC IP fraction
2-6
7-10
11-15
16-20
21-27
2-6
7-10
11-15
16-20
21-27
GSC
GFSC
50-
37-
25-
procaspase-8
s.e.

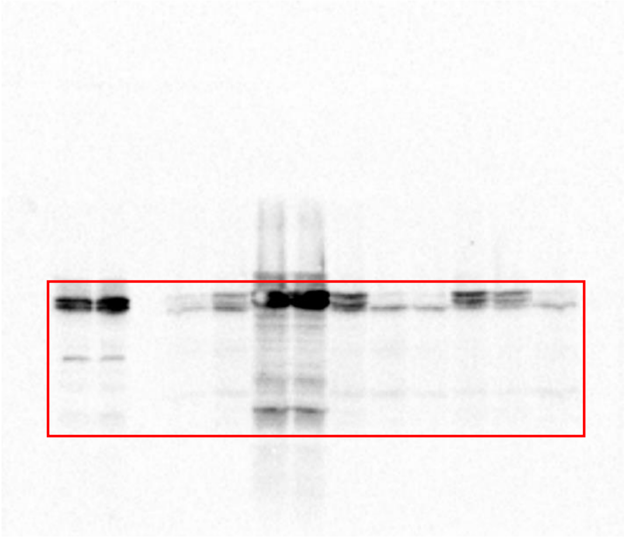

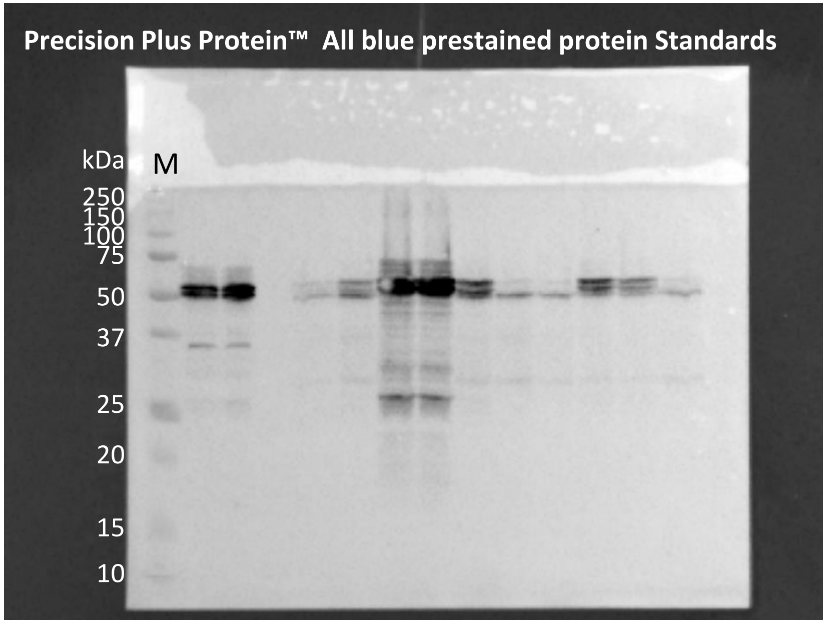
Precision Plus Protein™ All blue prestained protein Standards
kDa
M
250
150
100
75
50
37
25
20
15
10

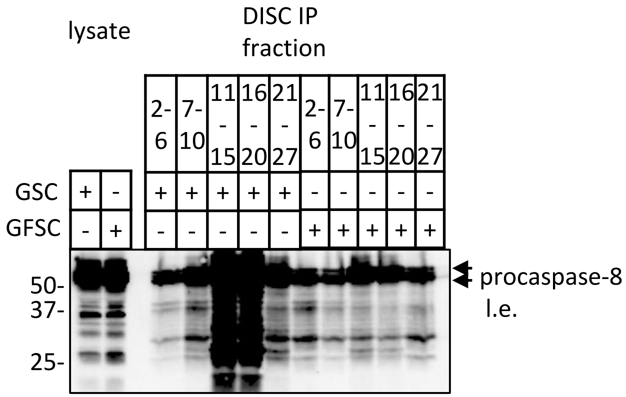
lysate
DISC IP fraction
2-6
7-10
11-15
16-20
21-27
2-6
7-10
11-15
16-20
21-27
GSC + - + + + + + - - - - -
GFSC - + - - - - - + + + + +
50-
37-
25-
procaspase-8
l.e.

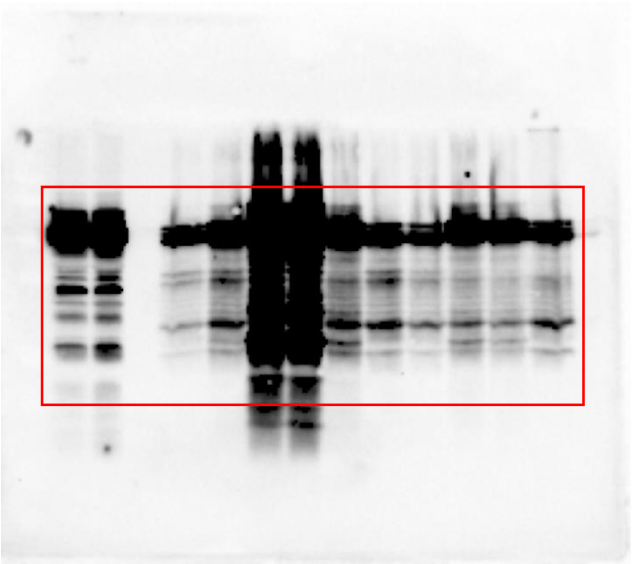

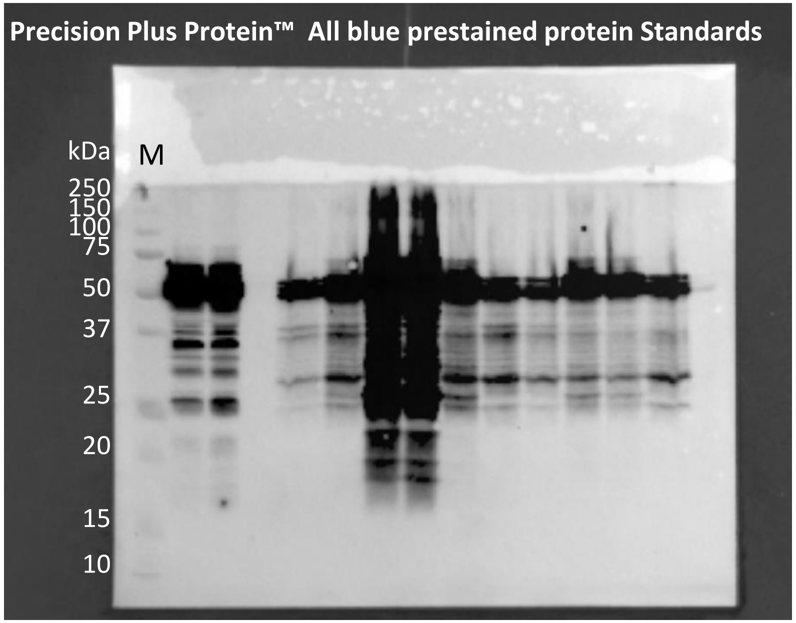
Precision Plus Protein™ All blue prestained protein Standards
kDa
M
250
150
100
75
50
37
25
20
15
10

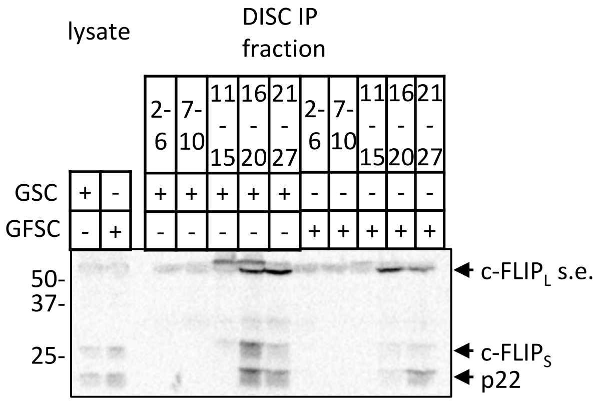
lysate
DISC IP fraction
2-6
7-10
11-15
16-20
21-27
2-6
7-10
11-15
16-20
21-27
GSC
GFSC
50-
37-
25-
c-FLIPL s.e.
c-FLIPS
p22

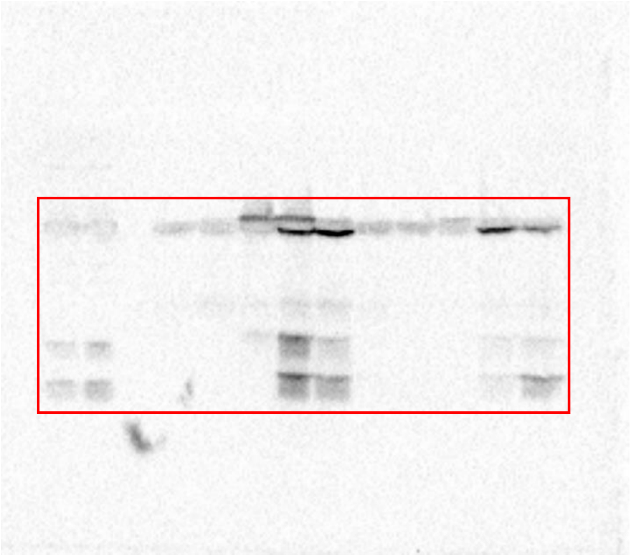

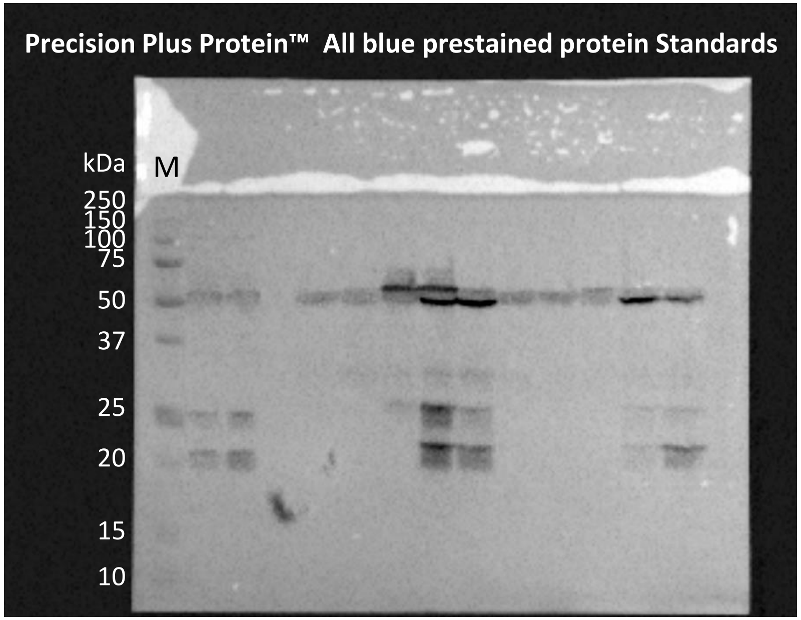
Precision Plus Protein™ All blue prestained protein Standards
kDa
M
250
150
100
75
50
37
25
20
15
10

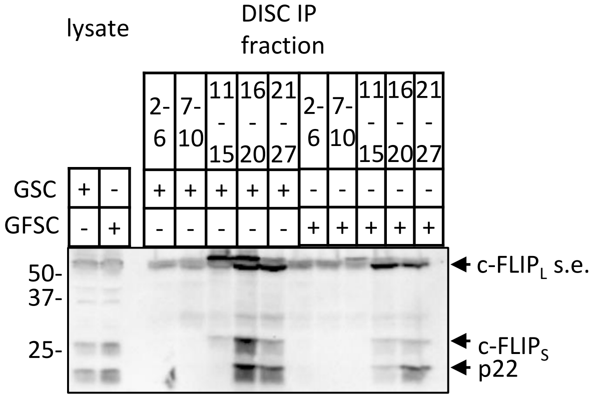
lysate
DISC IP fraction
2-6
7-10
11-15
16-20
21-27
2-6
7-10
11-15
16-20
21-27
GSC
GFSC
50-
37-
25-
c-FLIP$_L$ s.e.
c-FLIP$_S$
p22

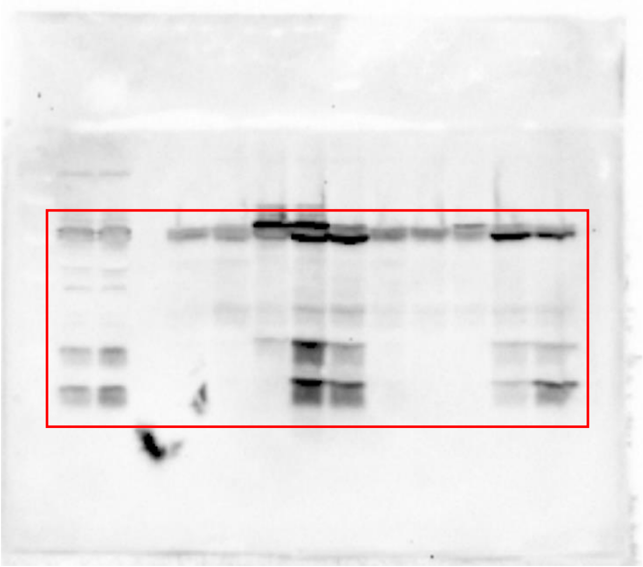

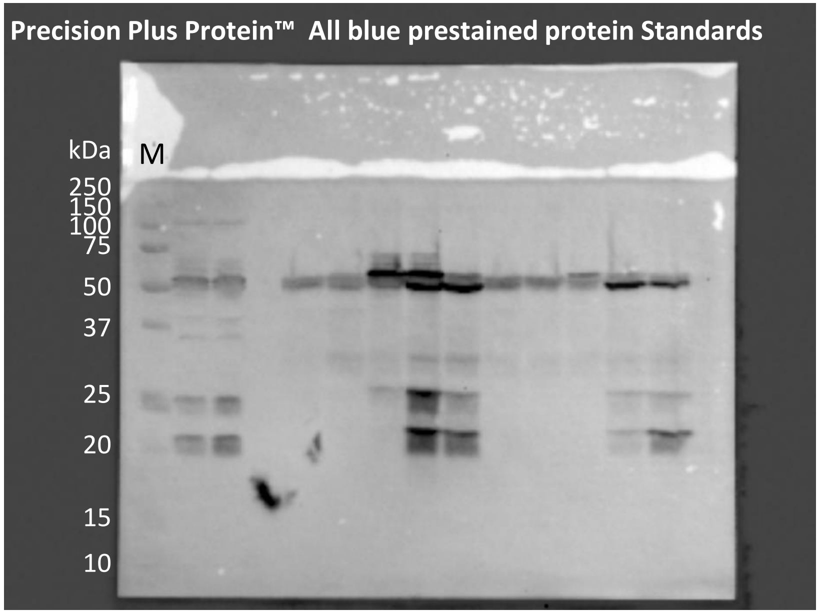
Precision Plus Protein™ All blue prestained protein Standards
kDa
M
250
150
100
75
50
37
25
20
15
10

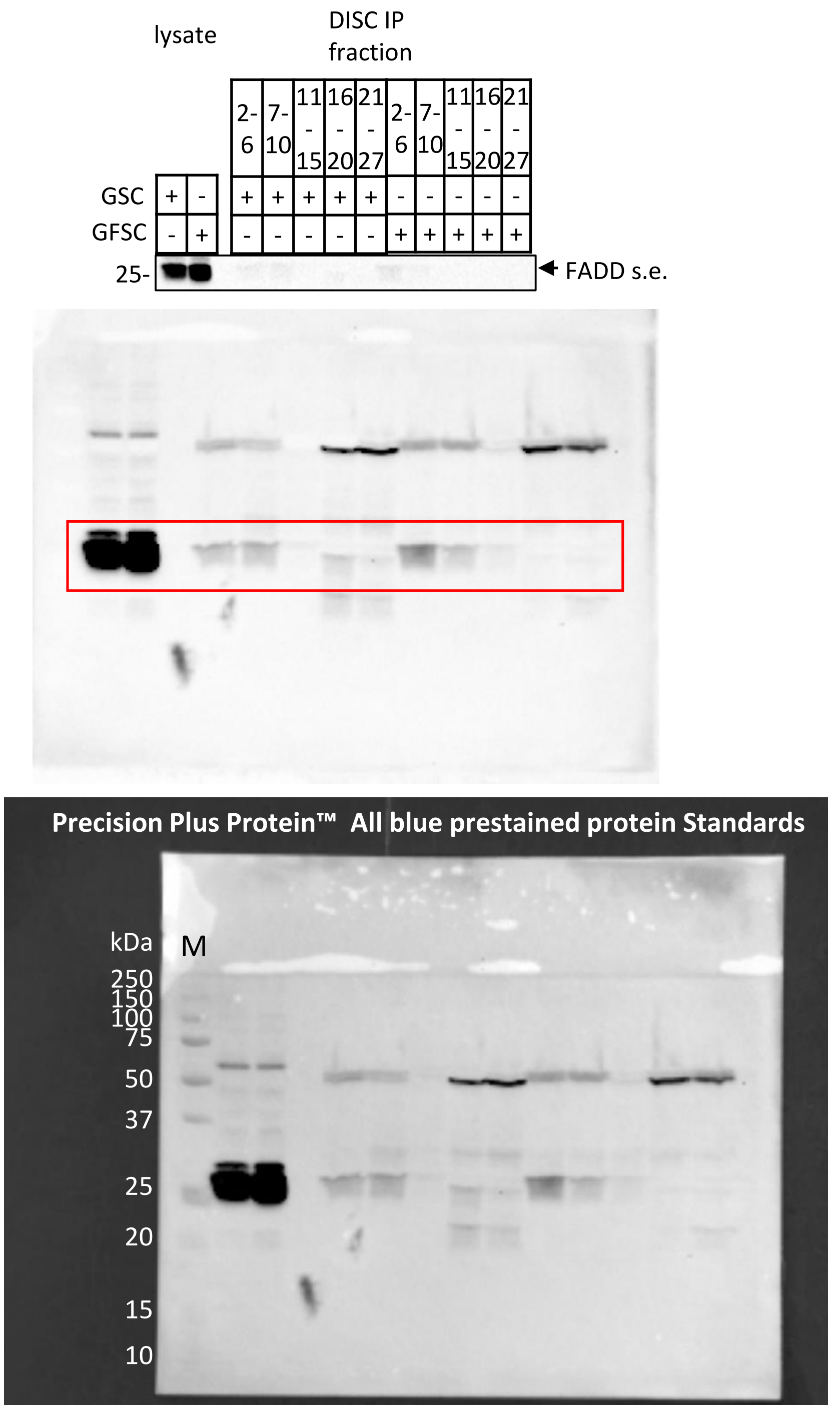
lysate
DISC IP fraction
2-6
7-10
11-15
16-20
21-27
2-6
7-10
11-15
16-20
21-27
GSC
+ - + + + + + - - - - -
GFSC
- + - - - - - + + + + +
25-
FADD s.e.
Precision Plus Protein™ All blue prestained protein Standards
kDa
M
250
150
100
75
50
37
25
20
15
10

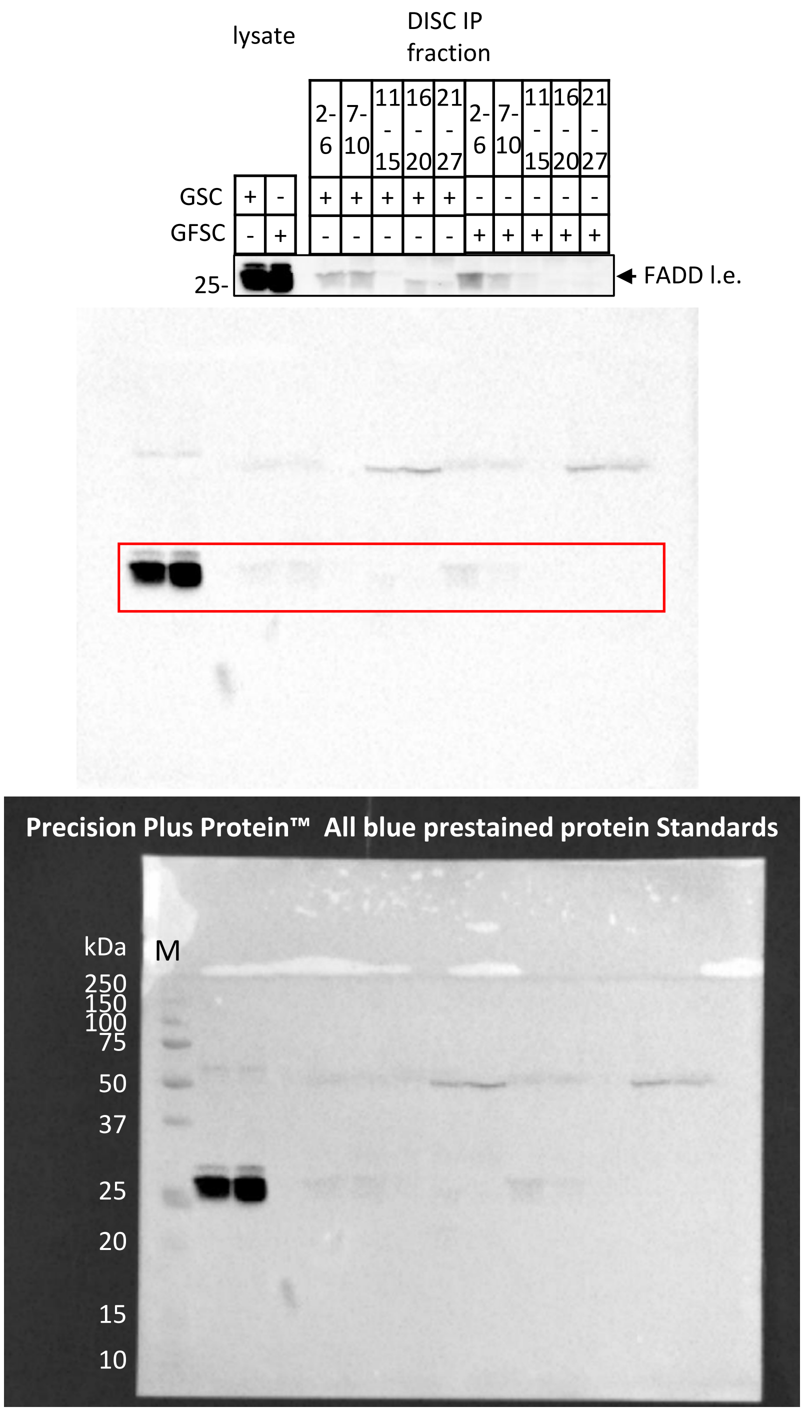
lysate
DISC IP fraction
2-6
7-10
11-15
16-20
21-27
2-6
7-10
11-15
16-20
21-27
GSC
+ - + + + + + - - - - -
GFSC
- + - - - - - + + + + +
25-
FADD l.e.
Precision Plus Protein™ All blue prestained protein Standards
kDa
M
250
150
100
75
50
37
25
20
15
10

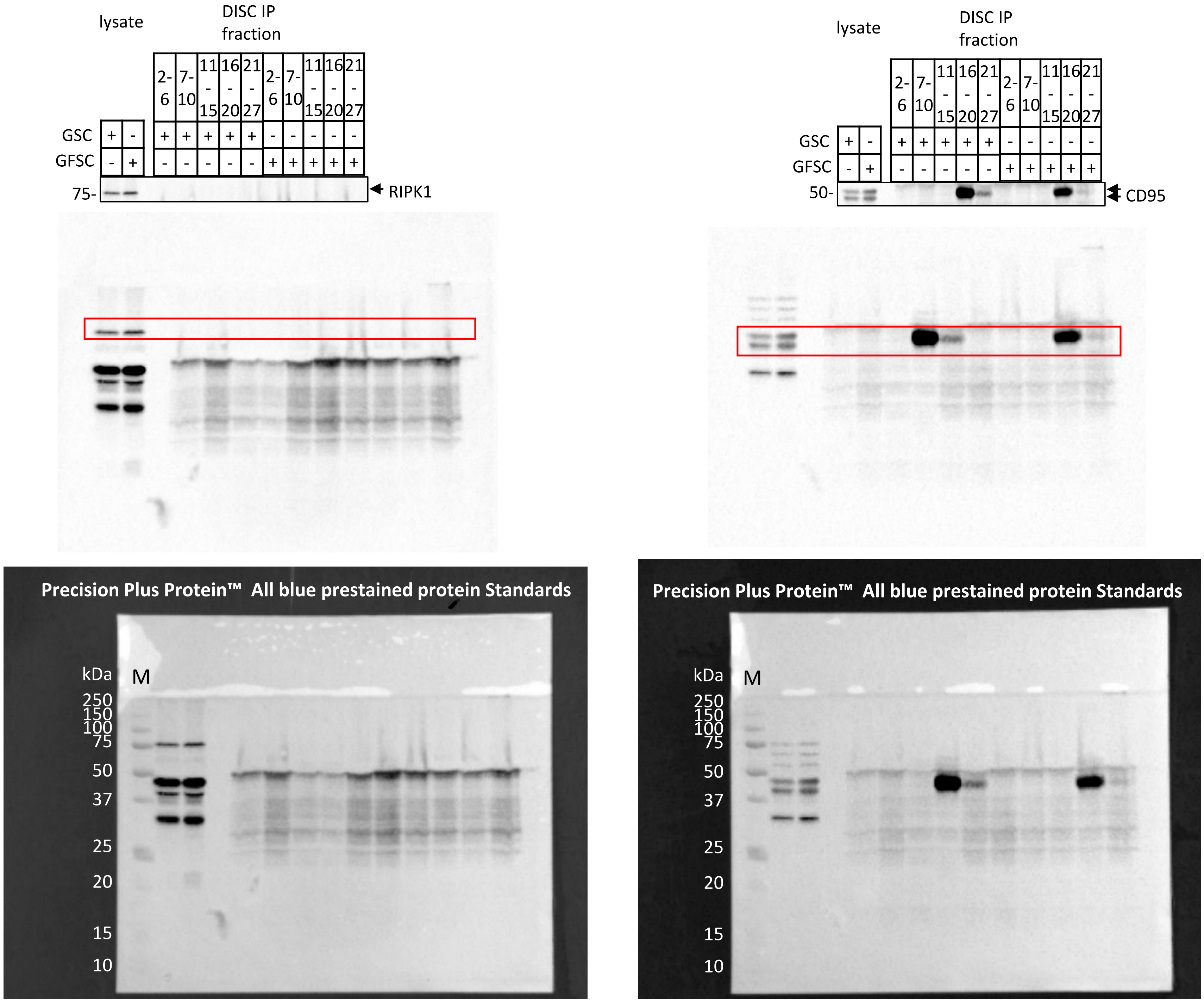

lysate
DISC IP fraction
2-6
7-10
11-15
16-20
21-27
GSC
GFSC
75-
RIPK1
50-
CD95
Precision Plus Protein™ All blue prestained protein Standards
kDa
M
250
150
100
75
50
37
25
20
15
10

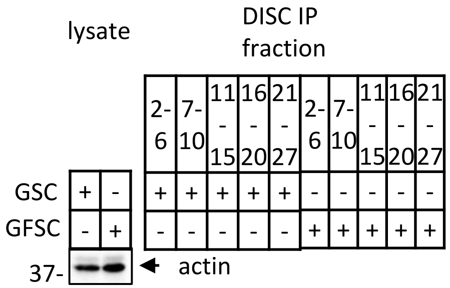
lysate
DISC IP fraction
2-6
7-10
11-15
16-20
21-27
2-6
7-10
11-15
16-20
21-27
GSC
GFSC
37-
actin

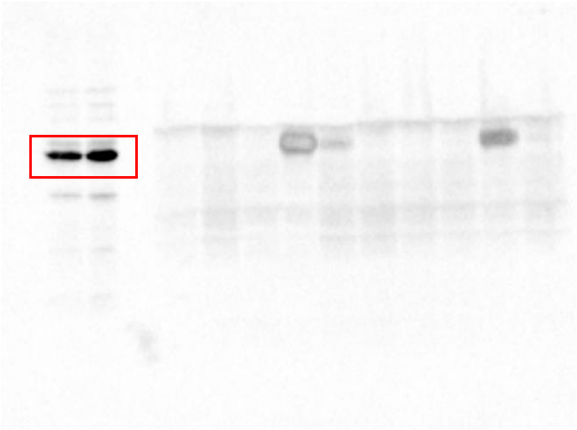

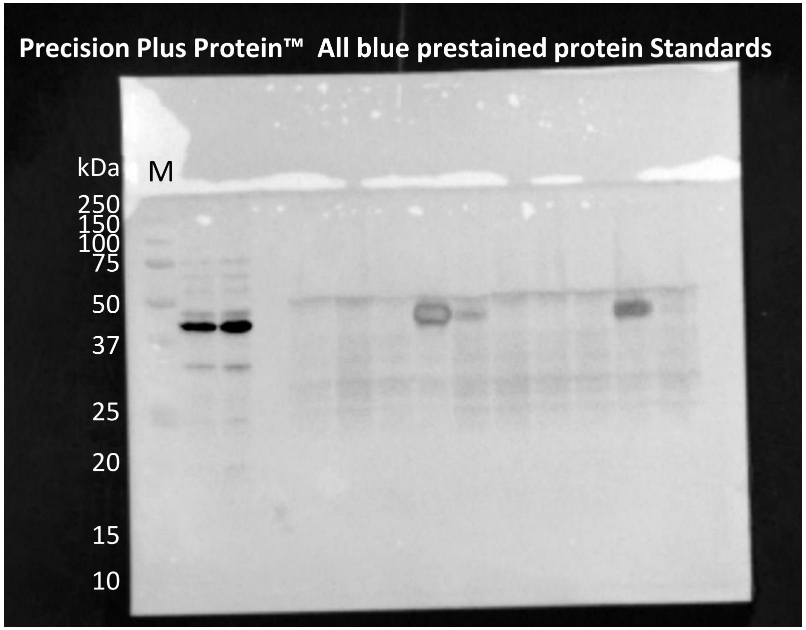
Precision Plus Protein™ All blue prestained protein Standards
kDa
M
250
150
100
75
50
37
25
20
15
10

c

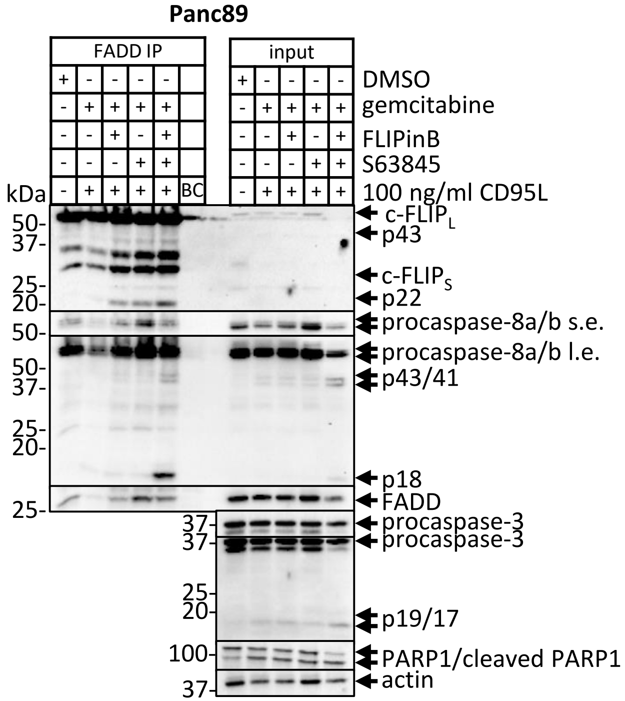
Panc89
FADD IP
input
DMSO
gemcitabine
FLIPinB
S63845
100 ng/ml CD95L
kDa
BC
c-FLIP$_L$
p43
c-FLIP$_S$
p22
procaspase-8a/b s.e.
procaspase-8a/b l.e.
p43/41
p18
FADD
procaspase-3
procaspase-3
p19/17
PARP1/cleaved PARP1
actin
50-
37-
25-
20-
100

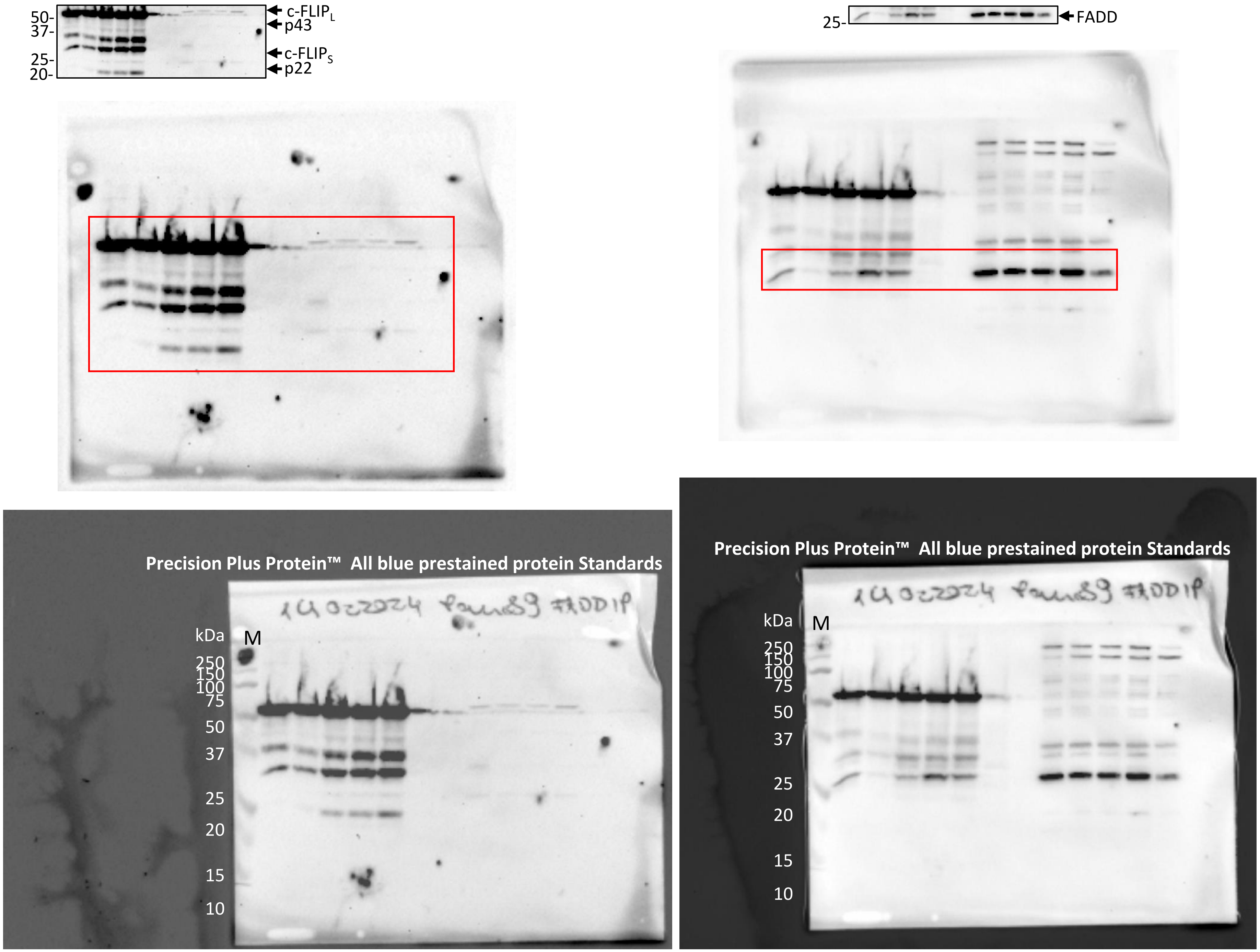
50-
37-
25-
20-
c-FLIP$_L$
p43
c-FLIP$_S$
p22
25-
FADD
Precision Plus Protein™ All blue prestained protein Standards
kDa
M
250
150
100
75
50
37
25
20
15
10
Precision Plus Protein™ All blue prestained protein Standards
kDa
M
250
150
100
75
50
37
25
20
15
10

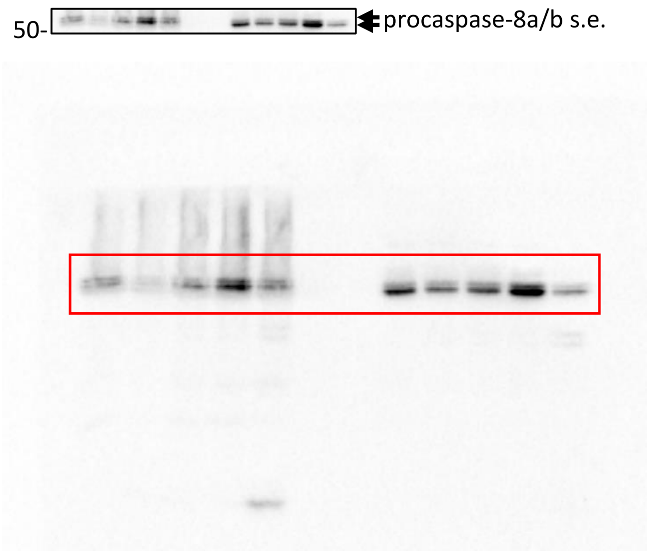
50-
procaspase-8a/b s.e.

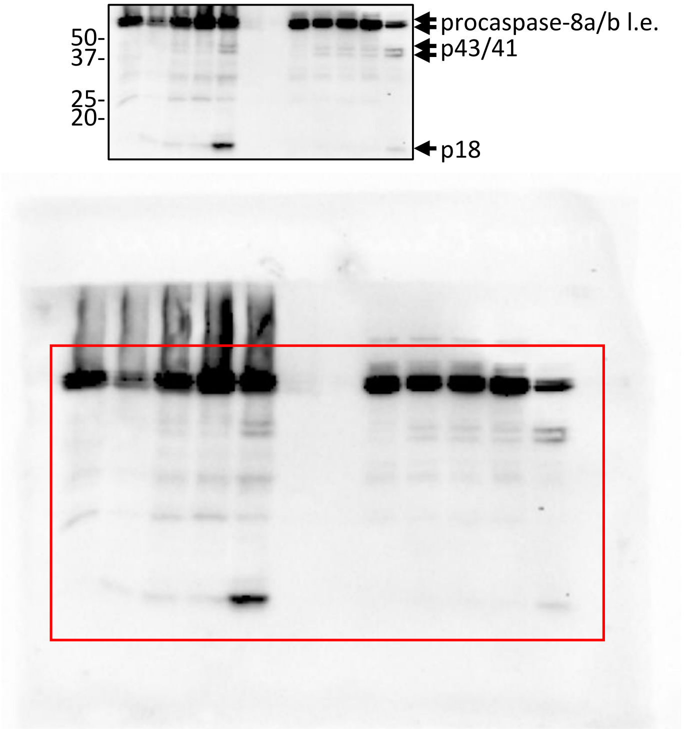
50-
37-
25-
20-
procaspase-8a/b l.e.
p43/41
p18

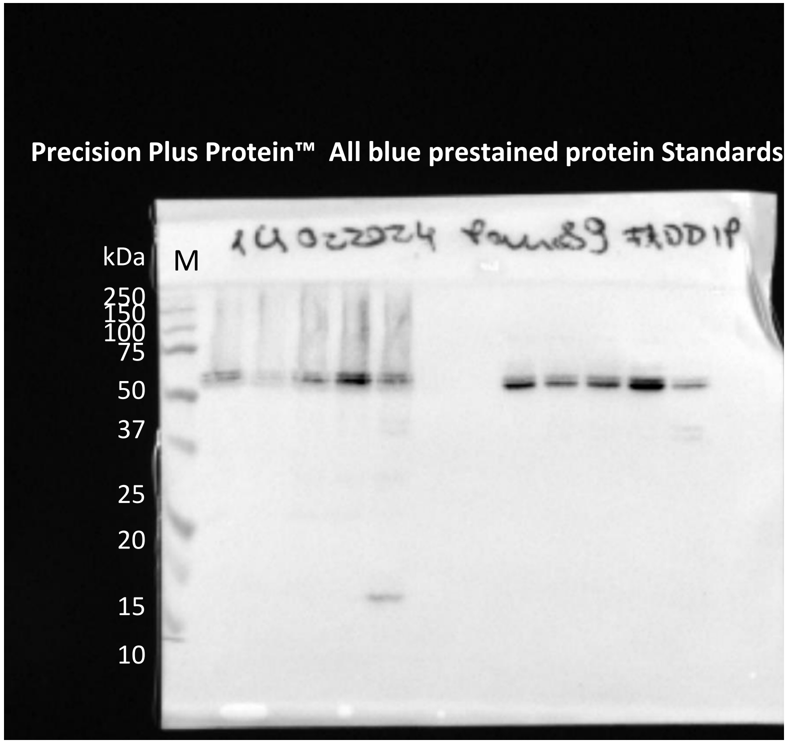
Precision Plus Protein™ All blue prestained protein Standards
kDa
M
250
150
100
75
50
37
25
20
15
10

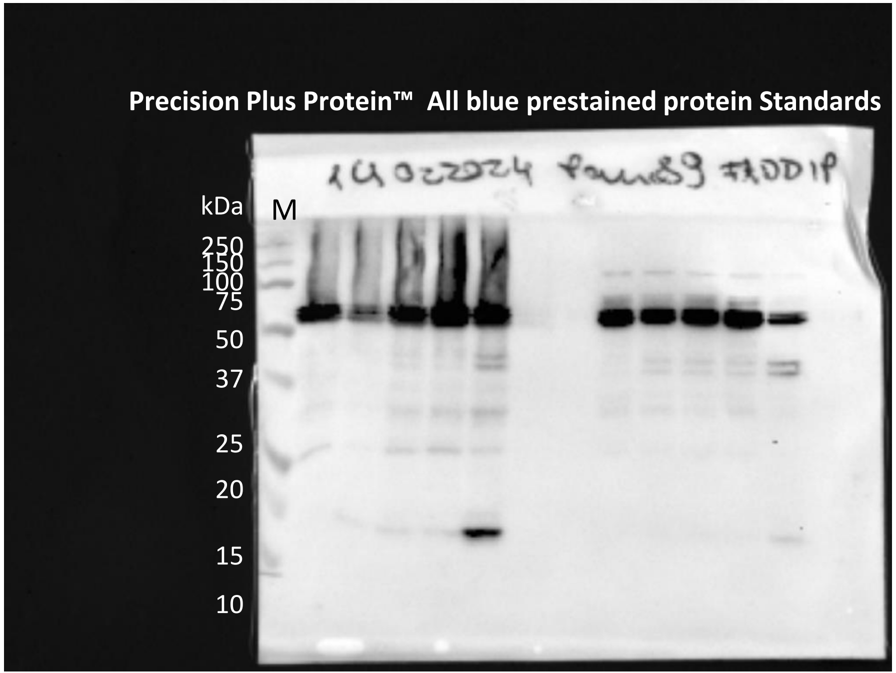
Precision Plus Protein™ All blue prestained protein Standards
kDa
M
250
150
100
75
50
37
25
20
15
10

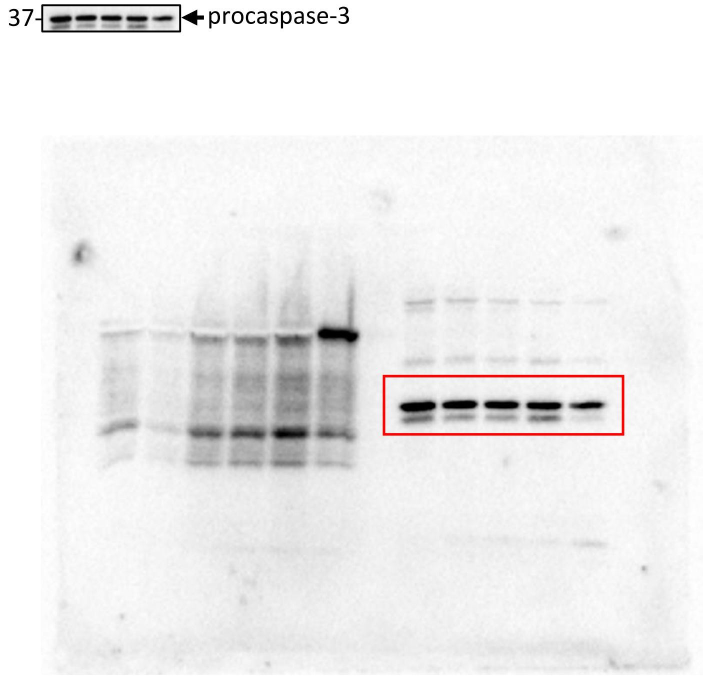
37
procaspase-3

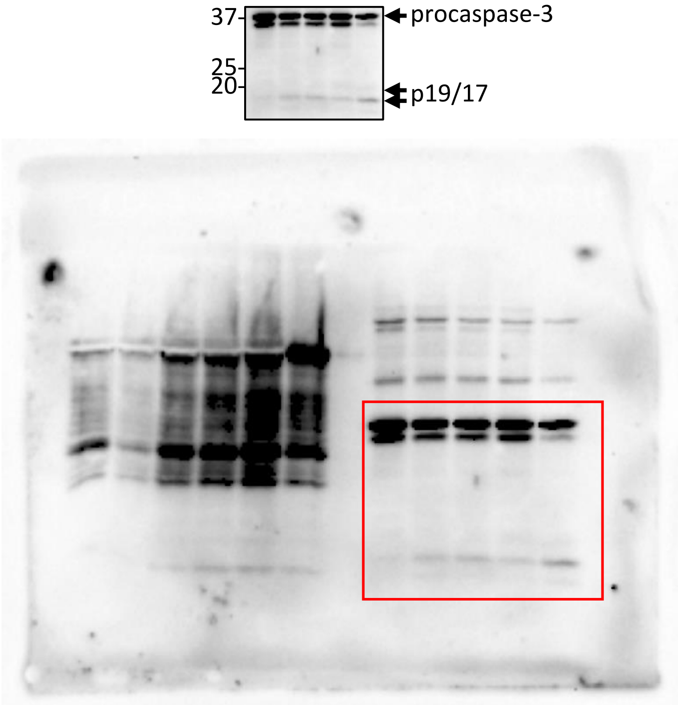
37
25
20
procaspase-3
p19/17

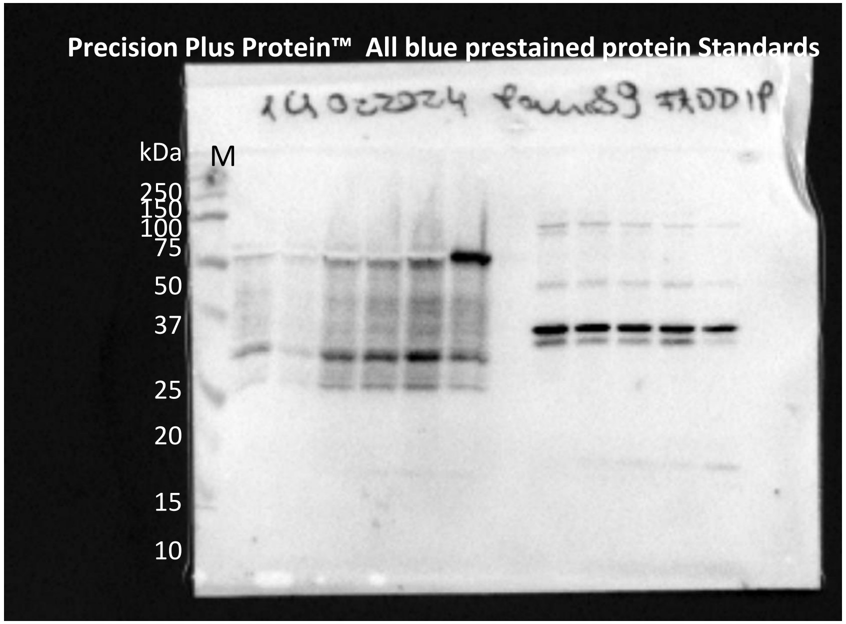
Precision Plus Protein™ All blue prestained protein Standards
kDa
M
250
150
100
75
50
37
25
20
15
10

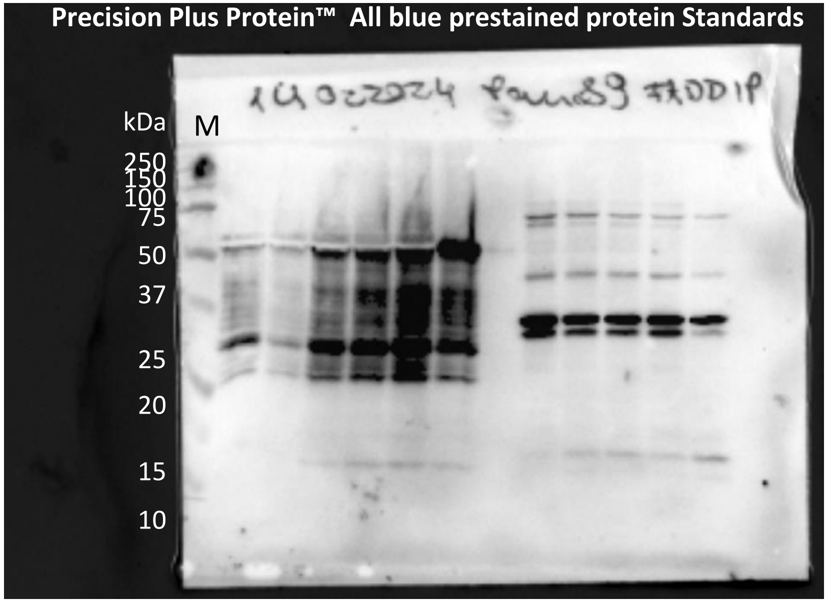
Precision Plus Protein™ All blue prestained protein Standards
kDa
M
250
150
100
75
50
37
25
20
15
10

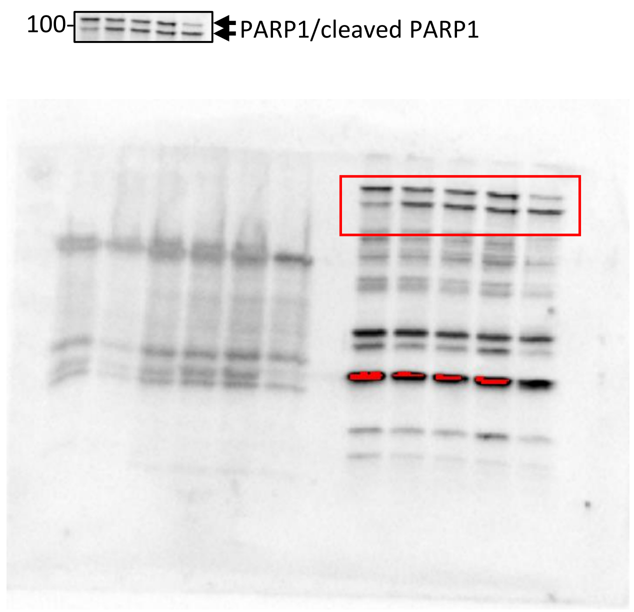
100
PARP1/cleaved PARP1

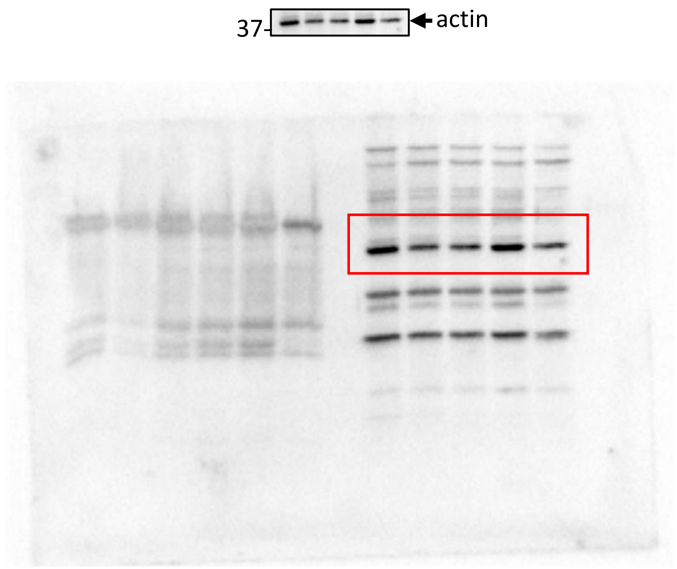
37
actin

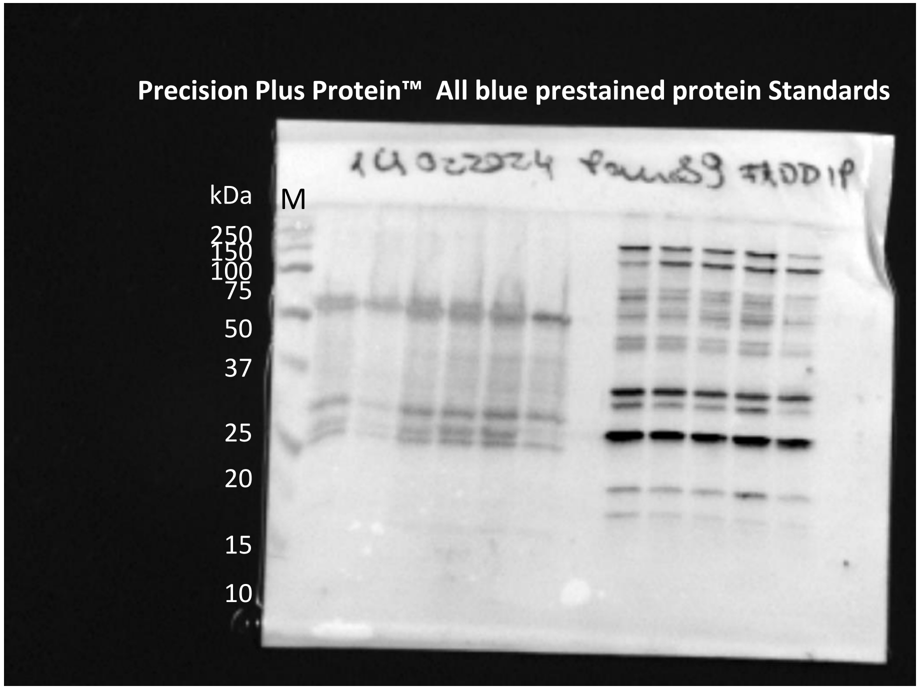
Precision Plus Protein™ All blue prestained protein Standards
kDa
M
250
150
100
75
50
37
25
20
15
10

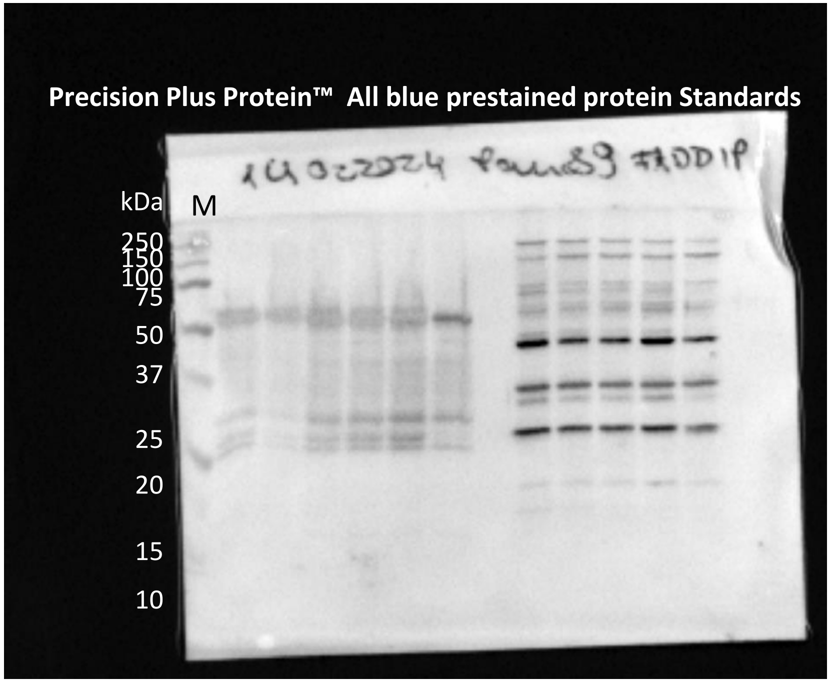
Precision Plus Protein™ All blue prestained protein Standards
kDa
M
250
150
100
75
50
37
25
20
15
10

a

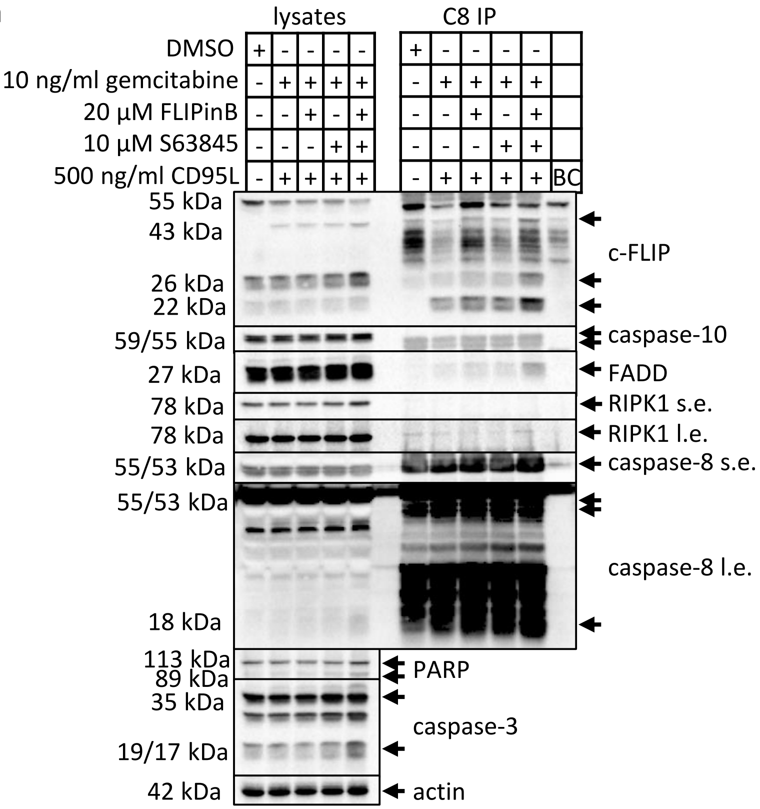

lysates
C8 IP
DMSO
10 ng/ml gemcitabine
20 µM FLIPinB
10 µM S63845
500 ng/ml CD95L
BC
55 kDa
43 kDa
26 kDa
22 kDa
c-FLIP
59/55 kDa
caspase-10
27 kDa
FADD
78 kDa
RIPK1 s.e.
78 kDa
RIPK1 l.e.
55/53 kDa
caspase-8 s.e.
55/53 kDa
caspase-8 l.e.
18 kDa
113 kDa
89 kDa
PARP
35 kDa
caspase-3
19/17 kDa
42 kDa
actin

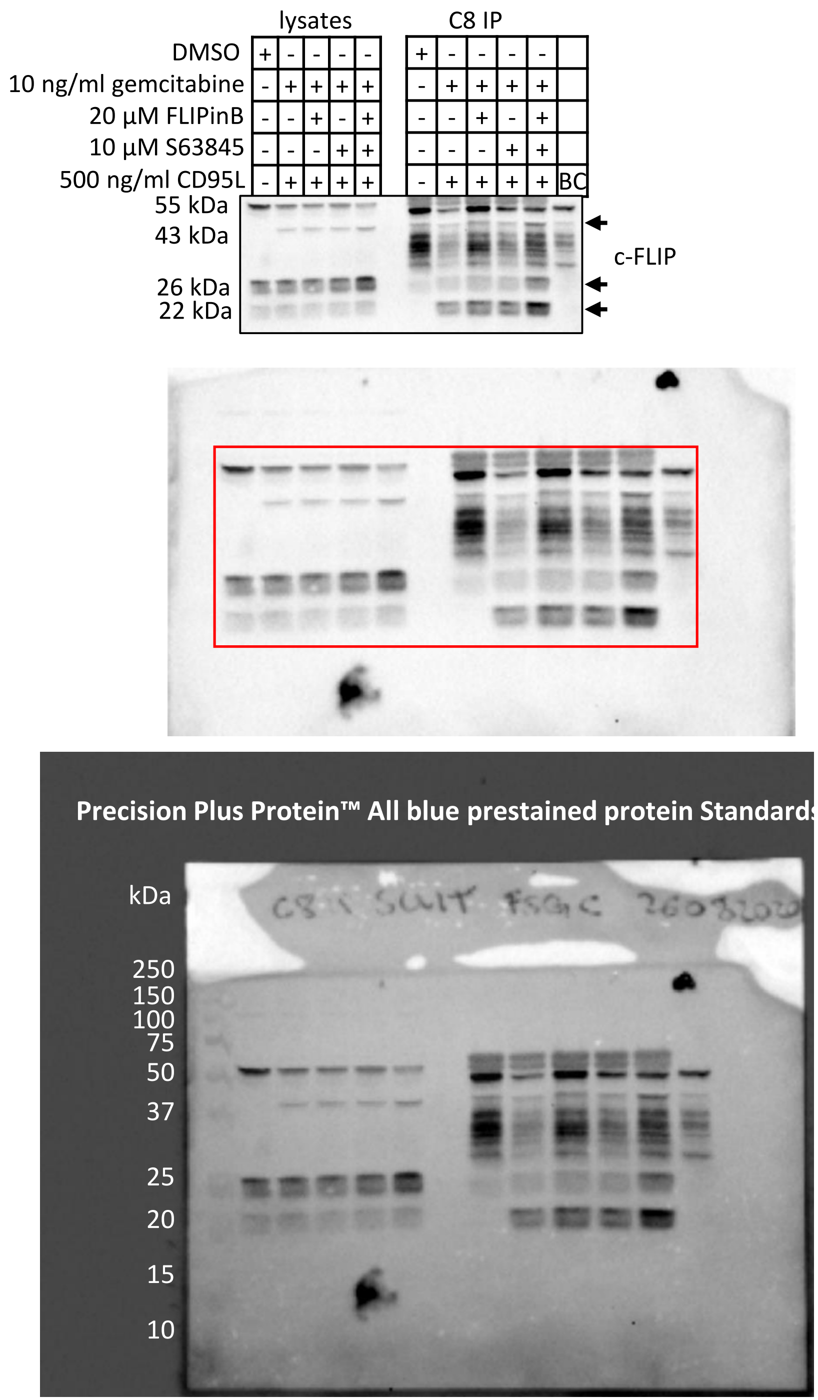
lysates
C8 IP
DMSO
10 ng/ml gemcitabine
20 µM FLIPinB
10 µM S63845
500 ng/ml CD95L
BC
55 kDa
43 kDa
26 kDa
22 kDa
c-FLIP
Precision Plus Protein™ All blue prestained protein Standards
kDa
250
150
100
75
50
37
25
20
15
10

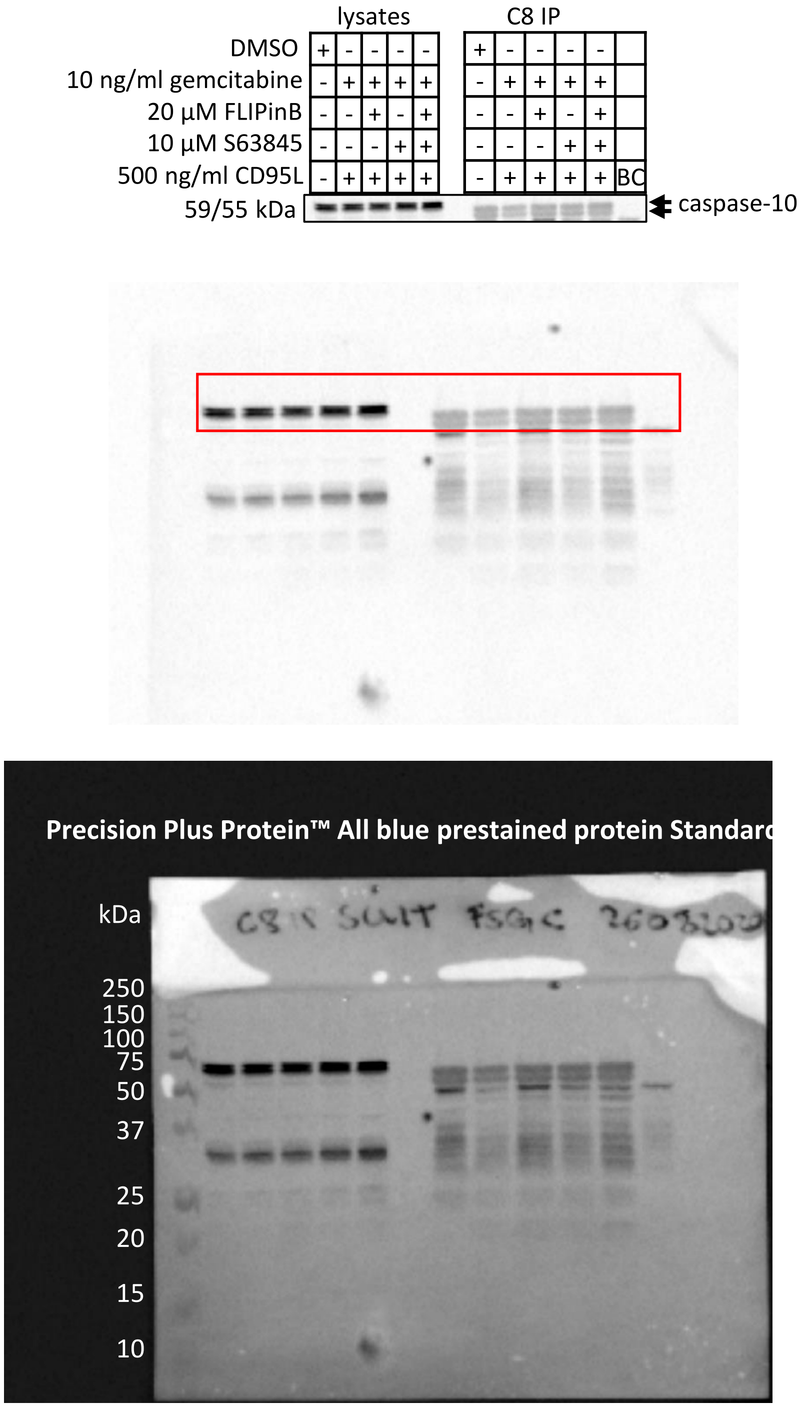
lysates
C8 IP
DMSO
10 ng/ml gemcitabine
20 µM FLIPinB
10 µM S63845
500 ng/ml CD95L
BC
59/55 kDa
caspase-10
Precision Plus Protein™ All blue prestained protein Standard
kDa
250
150
100
75
50
37
25
20
15
10

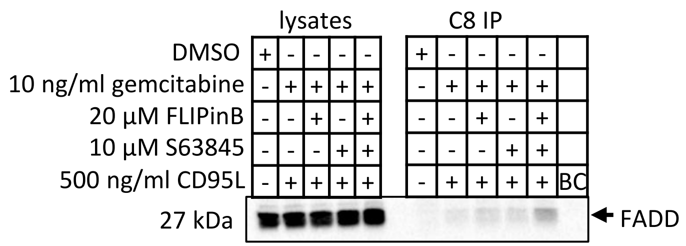
lysates
C8 IP
DMSO
10 ng/ml gemcitabine
20 µM FLIPinB
10 µM S63845
500 ng/ml CD95L
BC
27 kDa
FADD

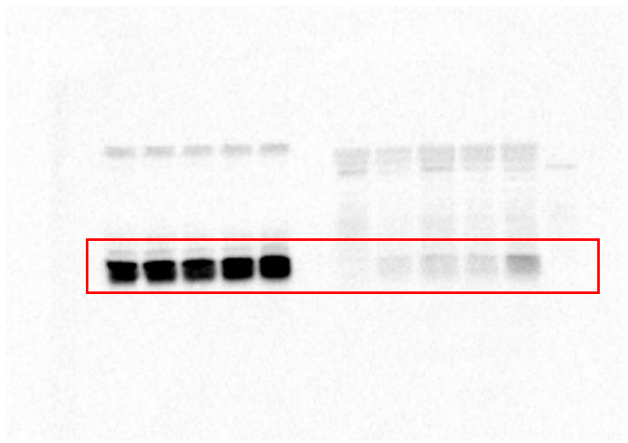

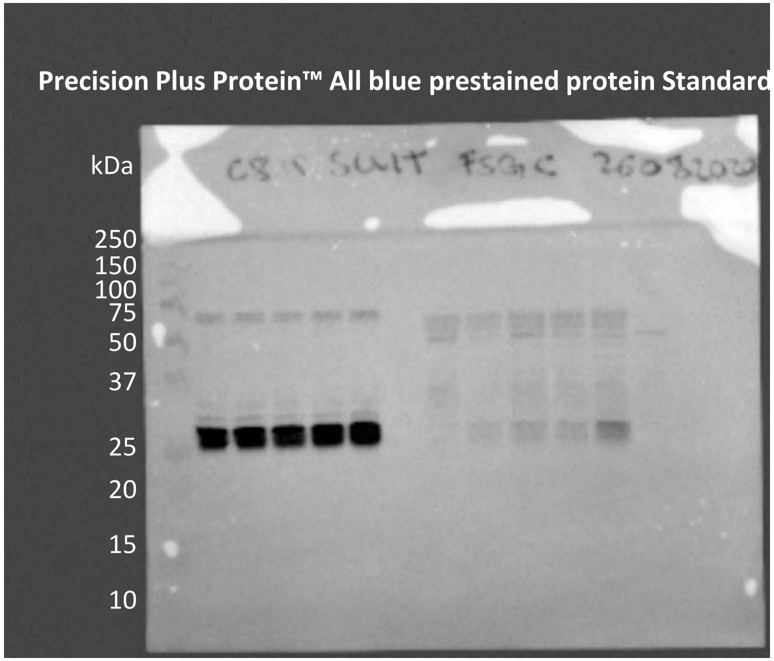
Precision Plus Protein™ All blue prestained protein Standard
kDa
250
150
100
75
50
37
25
20
15
10

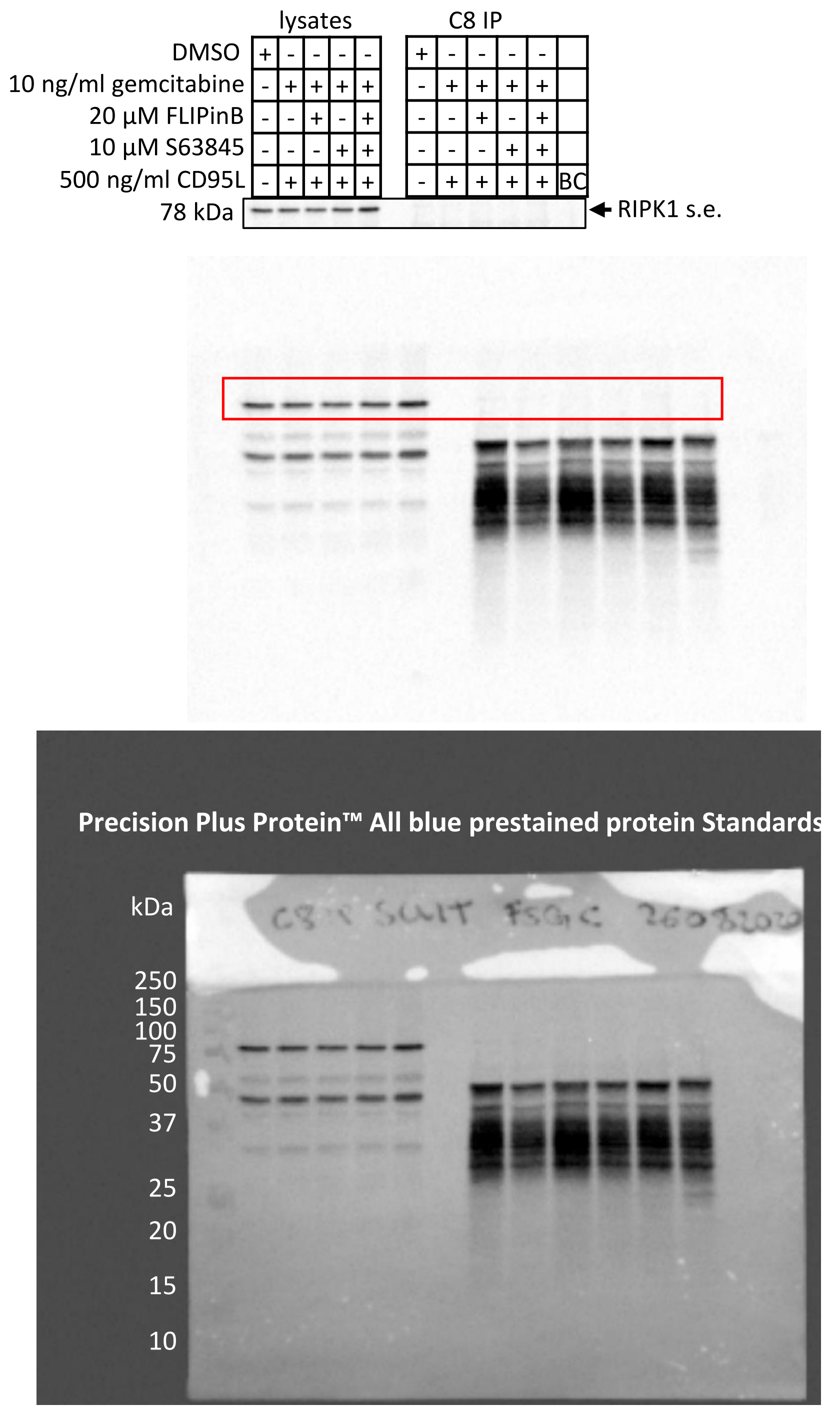
lysates
C8 IP
DMSO
10 ng/ml gemcitabine
20 µM FLIPinB
10 µM S63845
500 ng/ml CD95L
BC
78 kDa
RIPK1 s.e.
Precision Plus Protein™ All blue prestained protein Standards
kDa
250
150
100
75
50
37
25
20
15
10

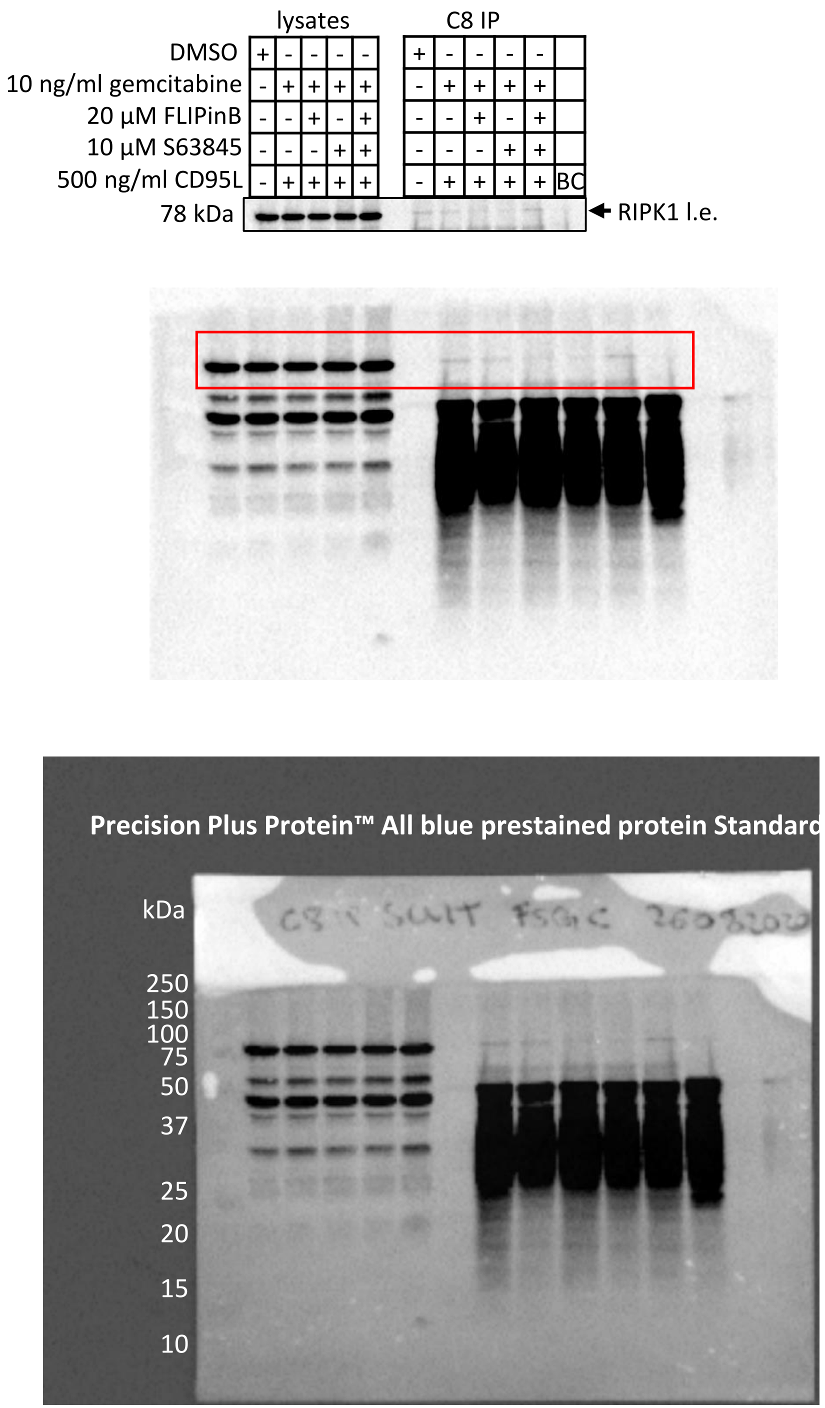
lysates
C8 IP
DMSO
10 ng/ml gemcitabine
20 µM FLIPinB
10 µM S63845
500 ng/ml CD95L
BC
78 kDa
RIPK1 l.e.
Precision Plus Protein™ All blue prestained protein Standard
kDa
250
150
100
75
50
37
25
20
15
10

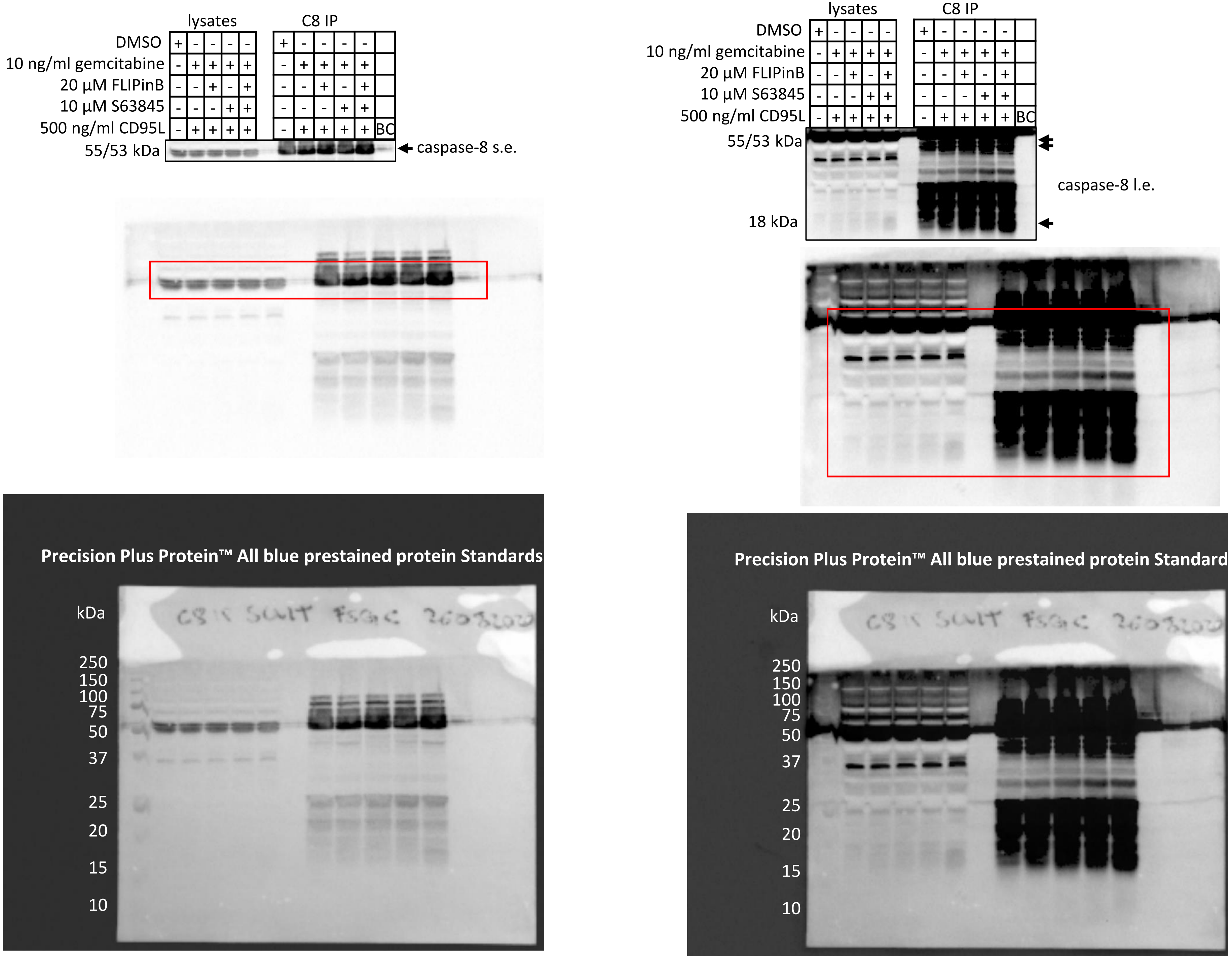
lysates
C8 IP
DMSO
10 ng/ml gemcitabine
20 µM FLIPinB
10 µM S63845
500 ng/ml CD95L
BC
55/53 kDa
caspase-8 s.e.
caspase-8 l.e.
18 kDa
Precision Plus Protein™ All blue prestained protein Standards
kDa
250
150
100
75
50
37
25
20
15
10
Precision Plus Protein™ All blue prestained protein Standard

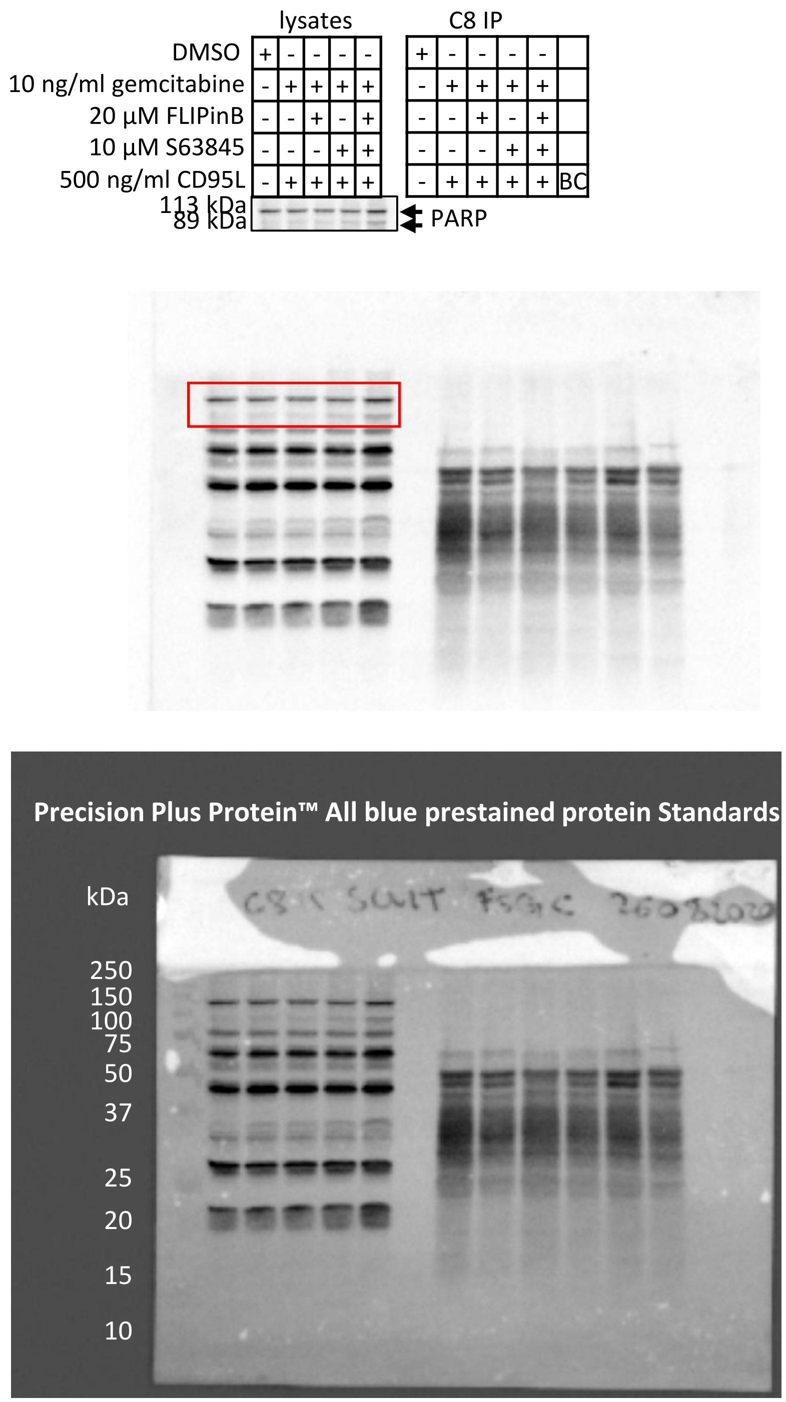
lysates
C8 IP
DMSO
10 ng/ml gemcitabine
20 µM FLIPinB
10 µM S63845
500 ng/ml CD95L
BC
113 kDa
89 kDa
PARP
Precision Plus Protein™ All blue prestained protein Standards
kDa
250
150
100
75
50
37
25
20
15
10

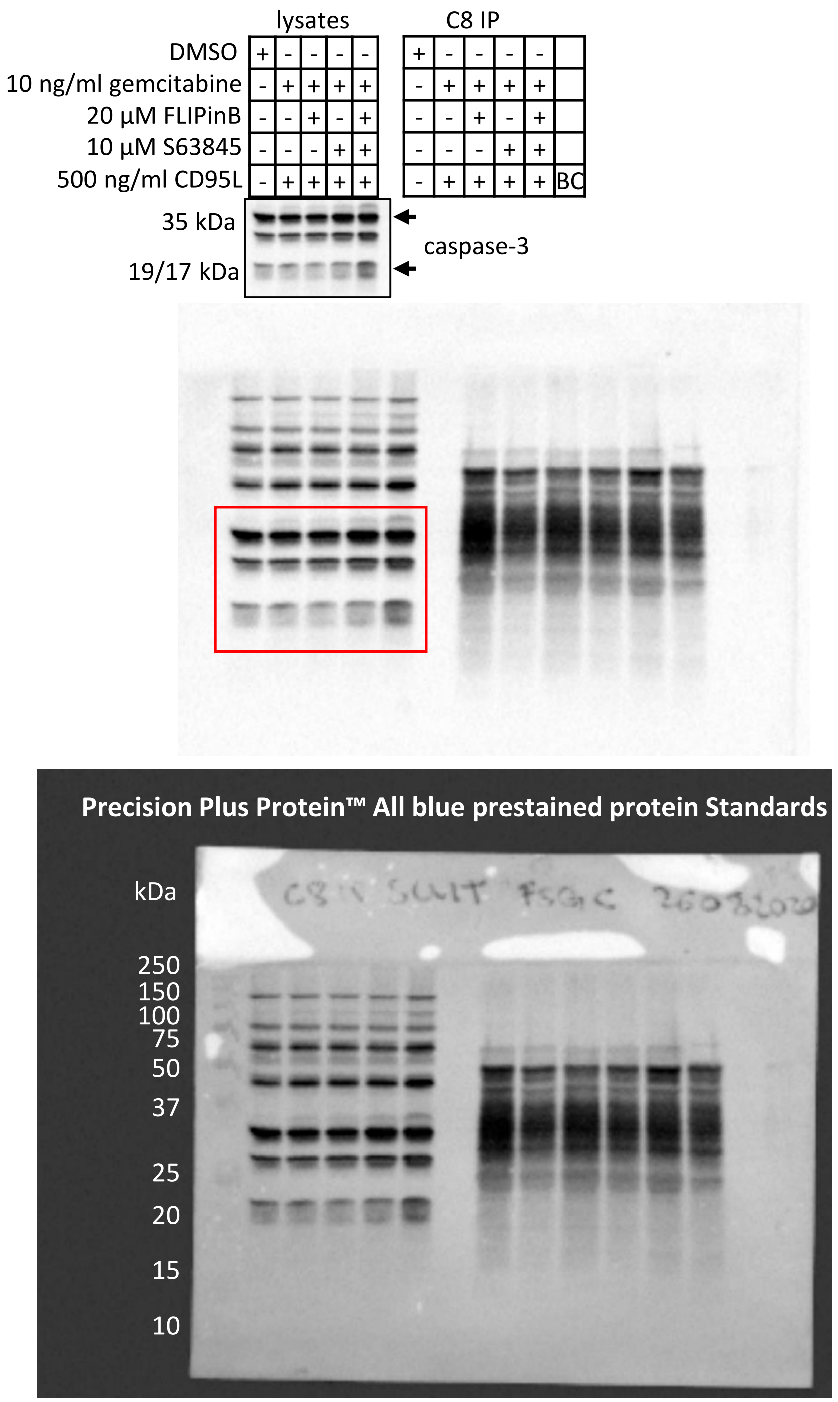
lysates
C8 IP
DMSO
10 ng/ml gemcitabine
20 µM FLIPinB
10 µM S63845
500 ng/ml CD95L
BC
35 kDa
19/17 kDa
caspase-3
Precision Plus Protein™ All blue prestained protein Standards
kDa
250
150
100
75
50
37
25
20
15
10

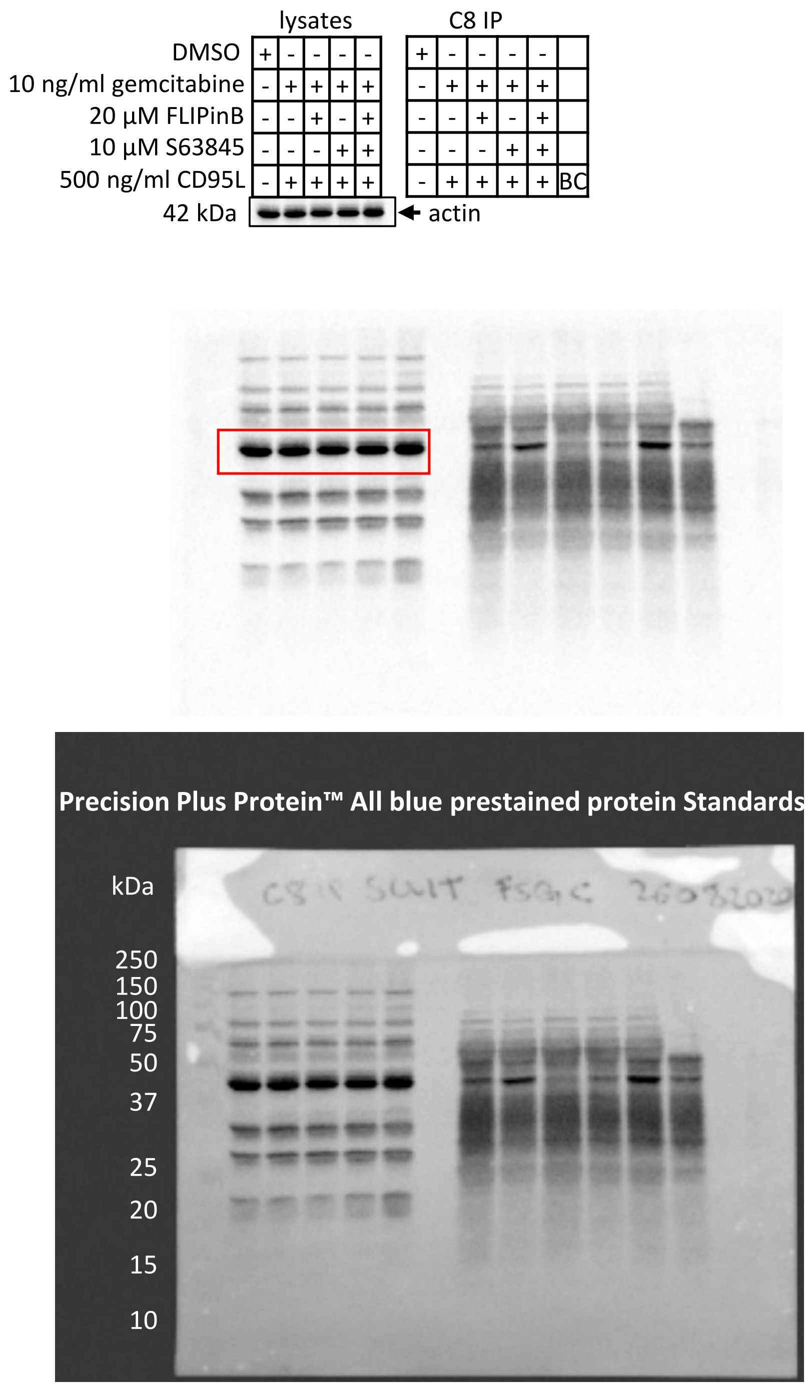

lysates
C8 IP
DMSO
10 ng/ml gemcitabine
20 µM FLIPinB
10 µM S63845
500 ng/ml CD95L
BC
42 kDa
actin
Precision Plus Protein™ All blue prestained protein Standards
kDa
250
150
100
75
50
37
25
20
15
10

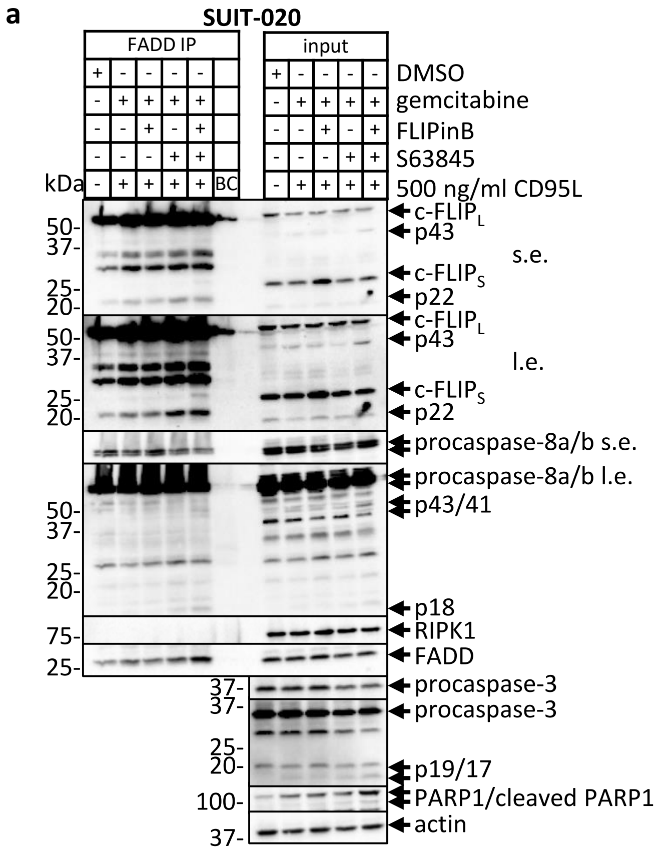
a
SUIT-020
FADD IP
input
DMSO
gemcitabine
FLIPinB
S63845
500 ng/ml CD95L
kDa
BC
c-FLIPL
p43
s.e.
c-FLIPS
p22
l.e.
procaspase-8a/b s.e.
procaspase-8a/b l.e.
p43/41
p18
RIPK1
FADD
procaspase-3
p19/17
PARP1/cleaved PARP1
actin

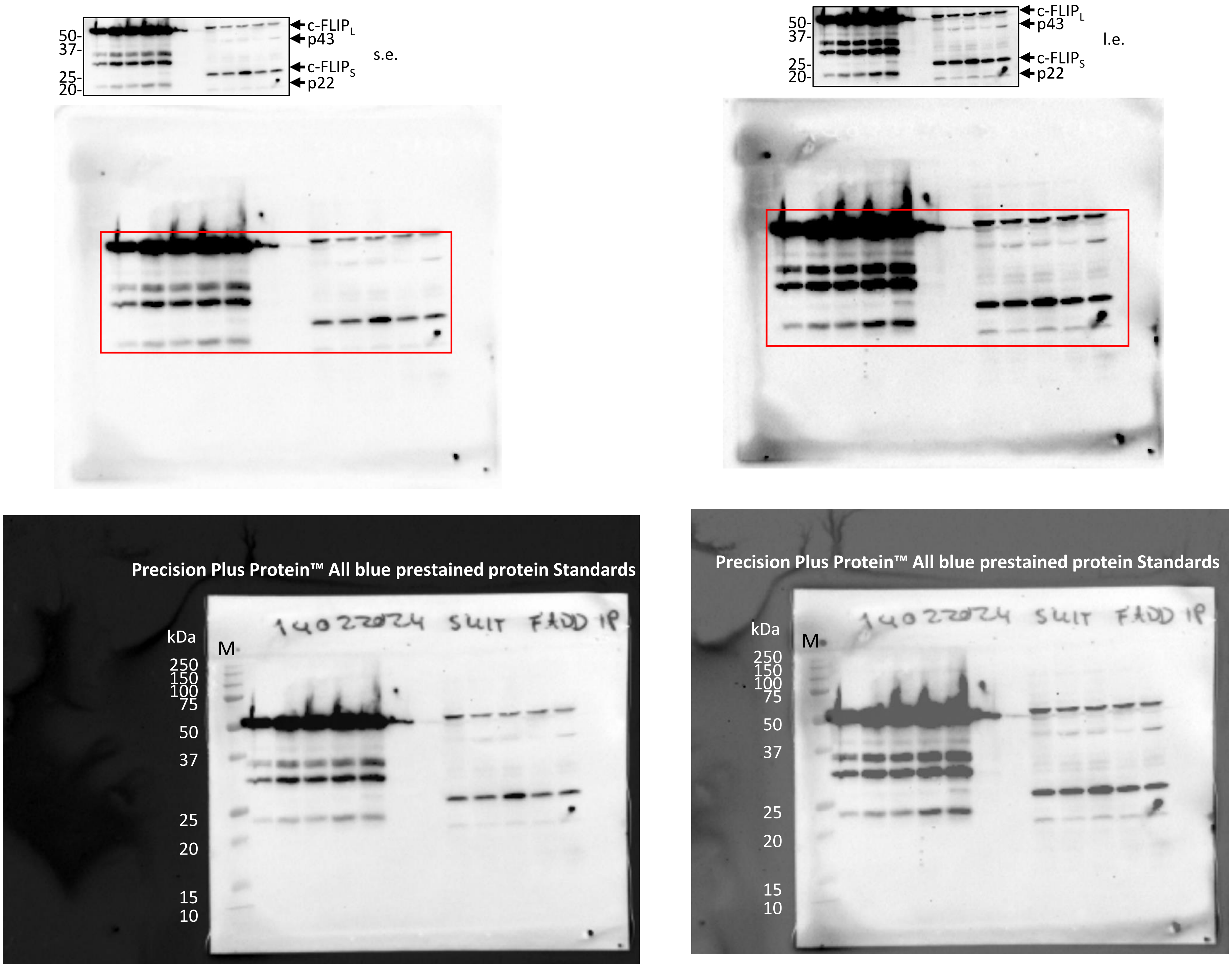

50-
37-
25-
20-
c-FLIP$_L$
p43
c-FLIP$_S$
p22
s.e.
l.e.
Precision Plus Protein™ All blue prestained protein Standards
kDa
M
250
150
100
75
50
37
25
20
15
10

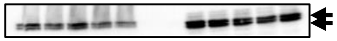
procaspase-8a/b s.e.

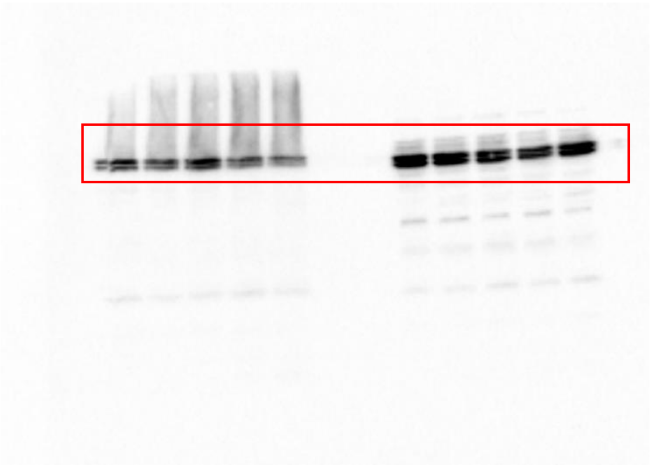

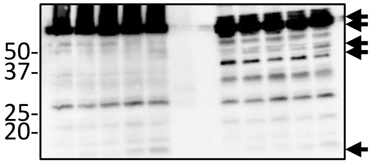
50-
37-
25-
20-
procaspase-8a/b l.e.
p43/41
p18

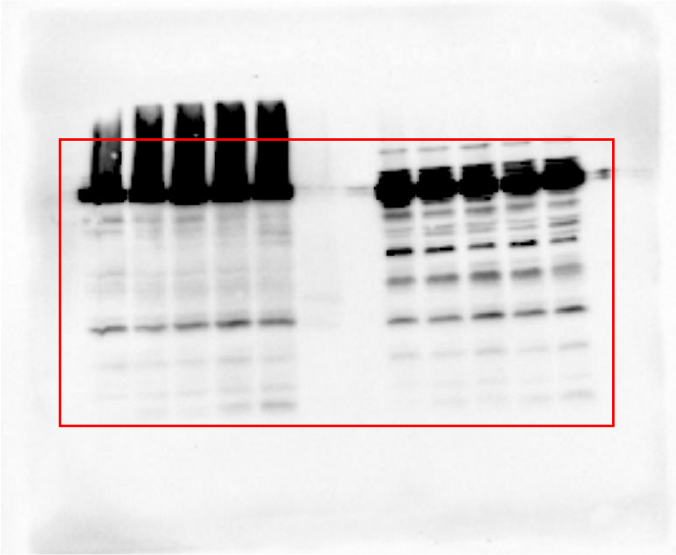

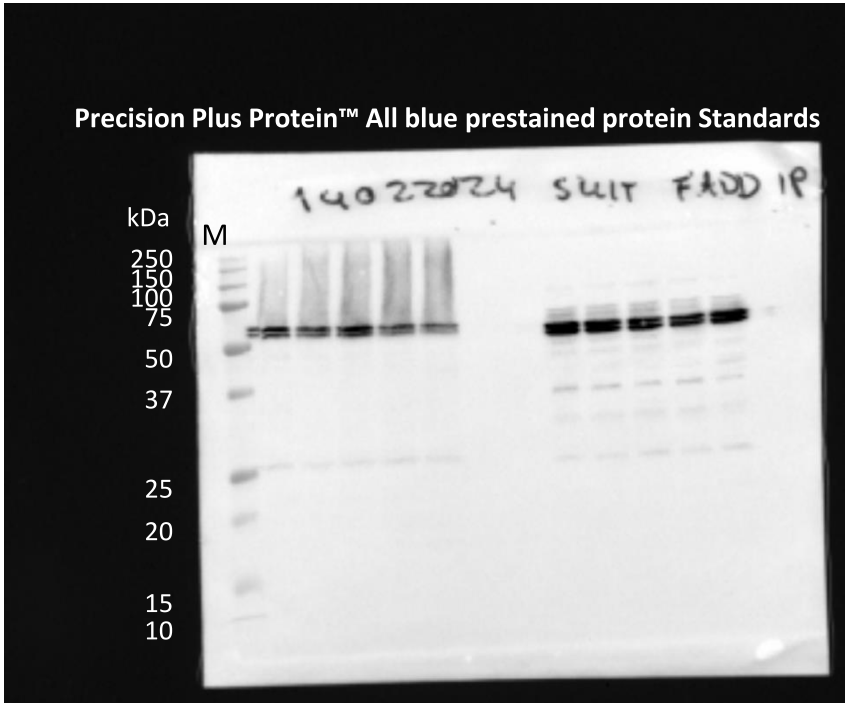
Precision Plus Protein™ All blue prestained protein Standards
kDa
M
250
150
100
75
50
37
25
20
15
10

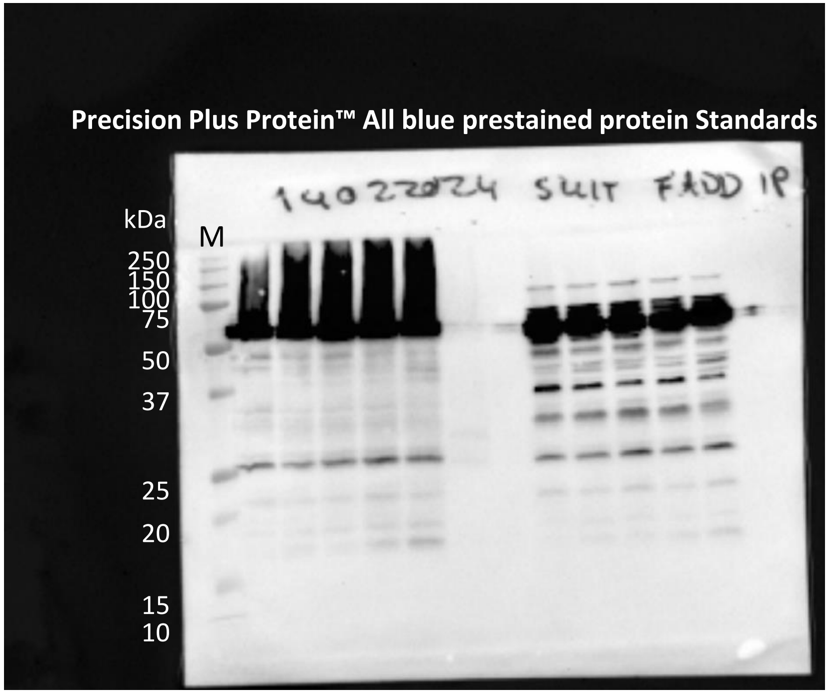
Precision Plus Protein™ All blue prestained protein Standards
kDa
M
250
150
100
75
50
37
25
20
15
10

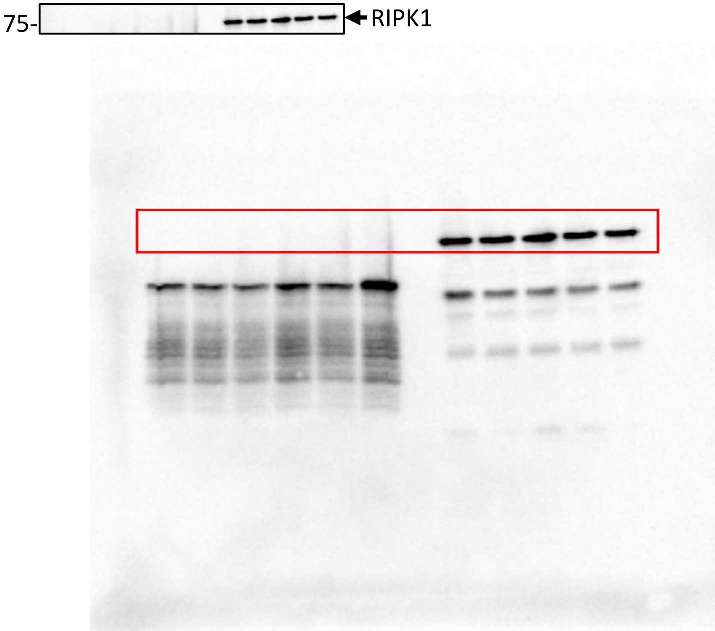
75-
RIPK1

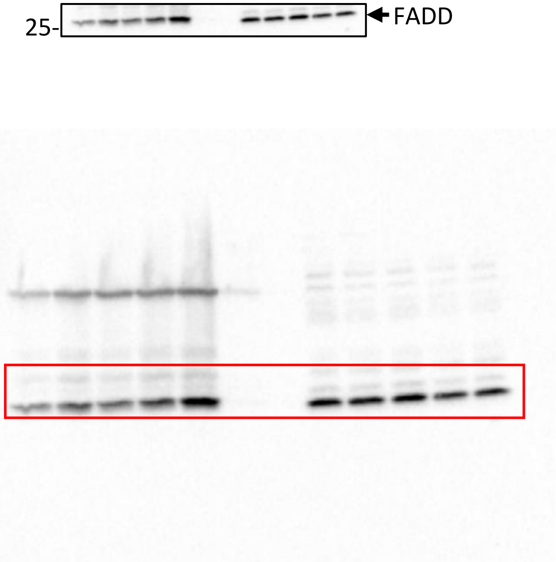
25-
FADD

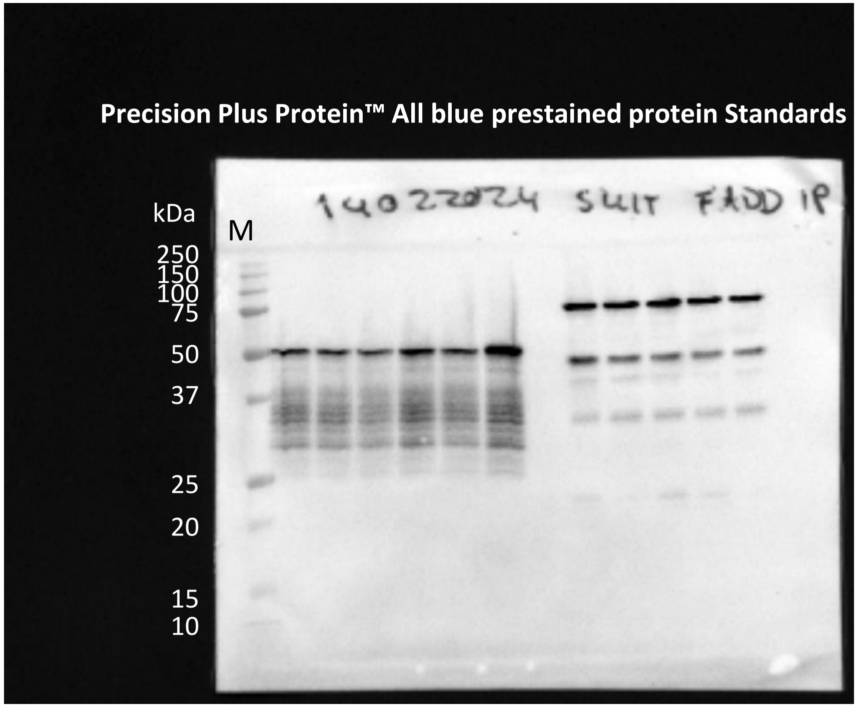
Precision Plus Protein™ All blue prestained protein Standards
kDa
M
250
150
100
75
50
37
25
20
15
10

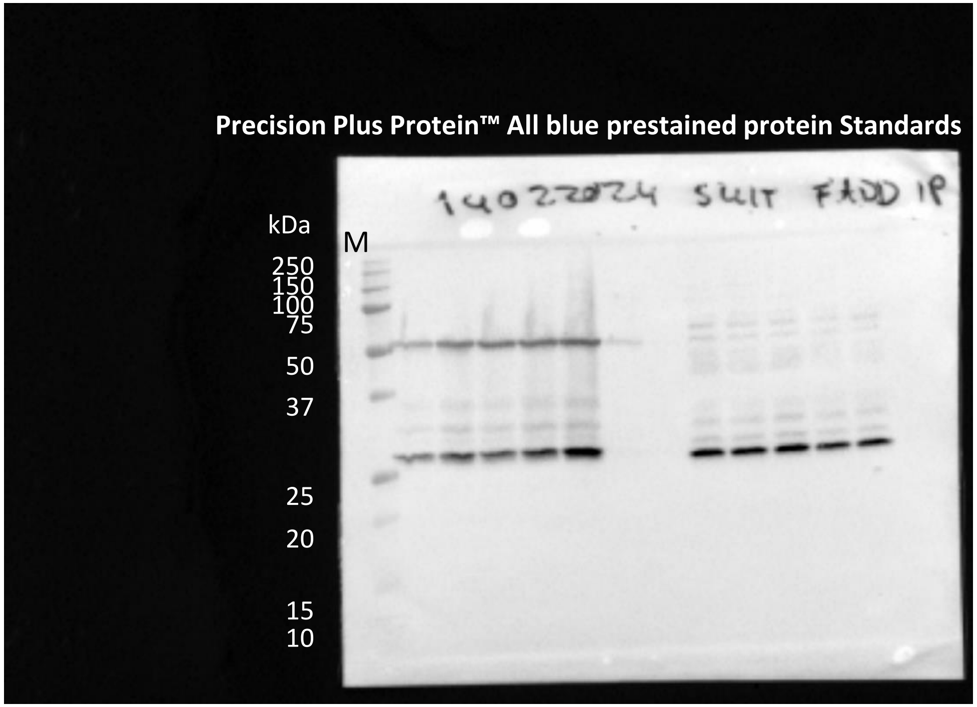
Precision Plus Protein™ All blue prestained protein Standards
kDa
M
250
150
100
75
50
37
25
20
15
10

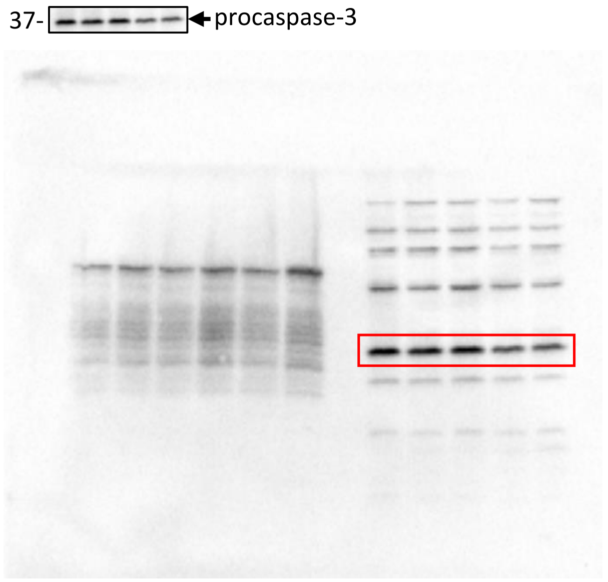
37-
procaspase-3

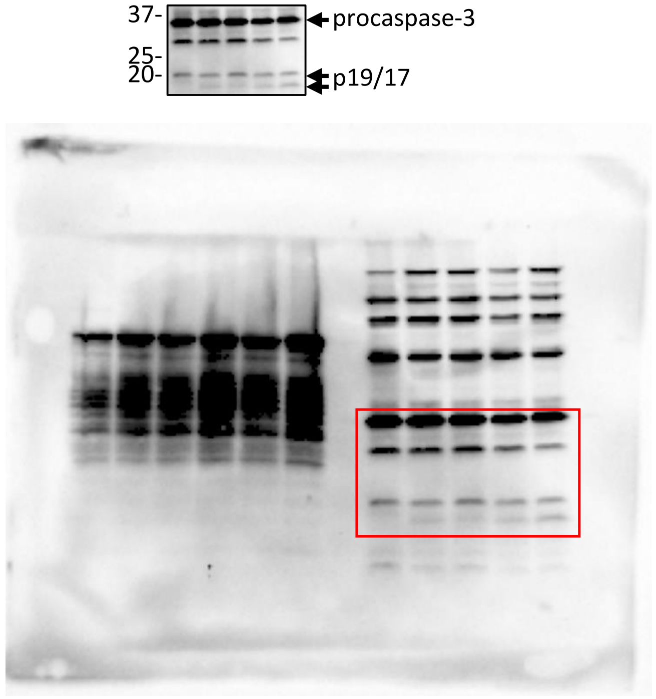
37-
procaspase-3
25-
20-
p19/17

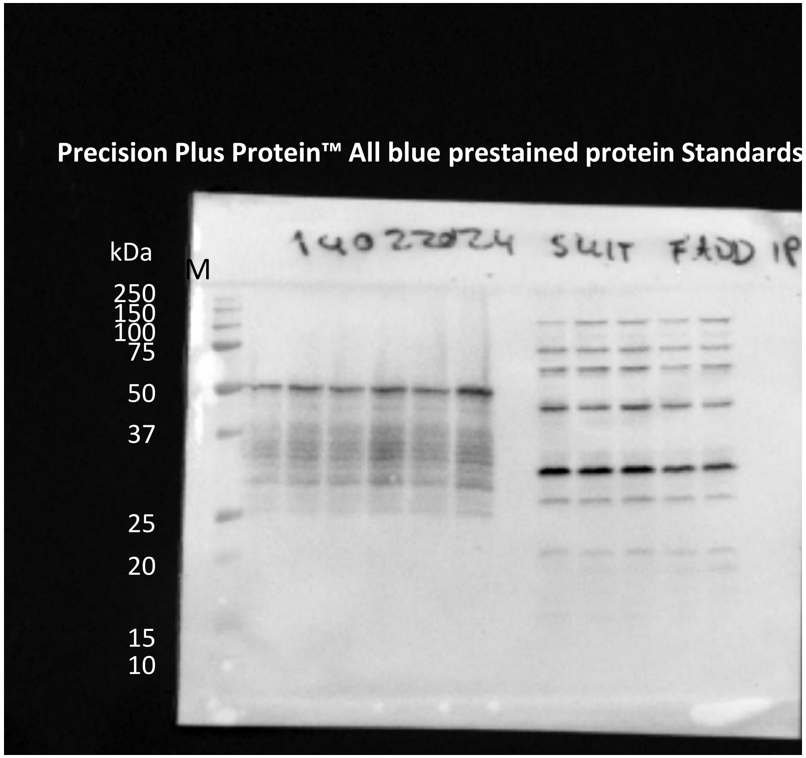
Precision Plus Protein™ All blue prestained protein Standards
kDa
M
14022024
FADD IP
250
150
100
75
50
37
25
20
15
10

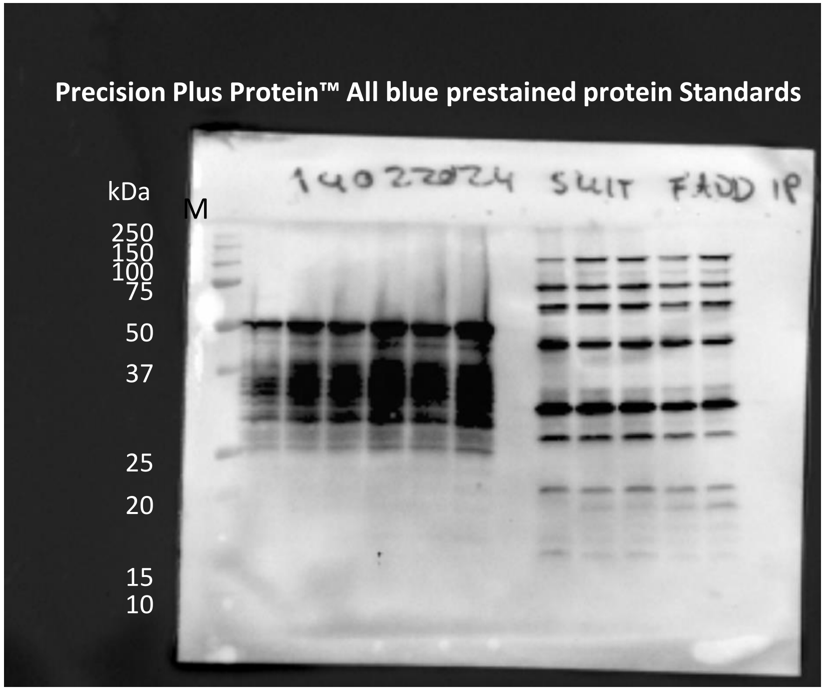
Precision Plus Protein™ All blue prestained protein Standards
kDa
M
14022024
FADD IP
250
150
100
75
50
37
25
20
15
10

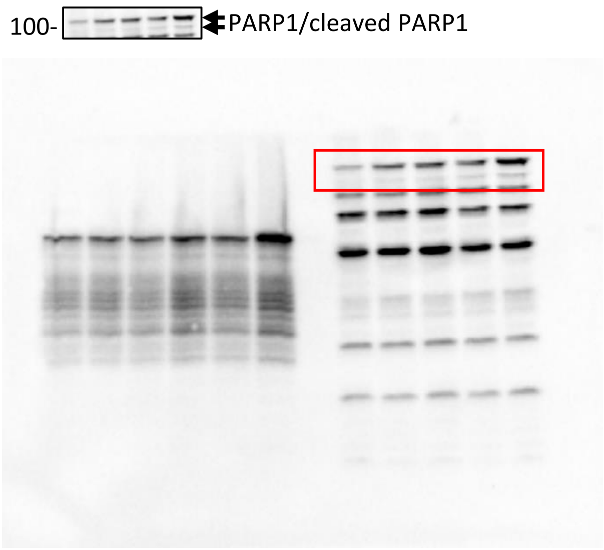
100-
PARP1/cleaved PARP1

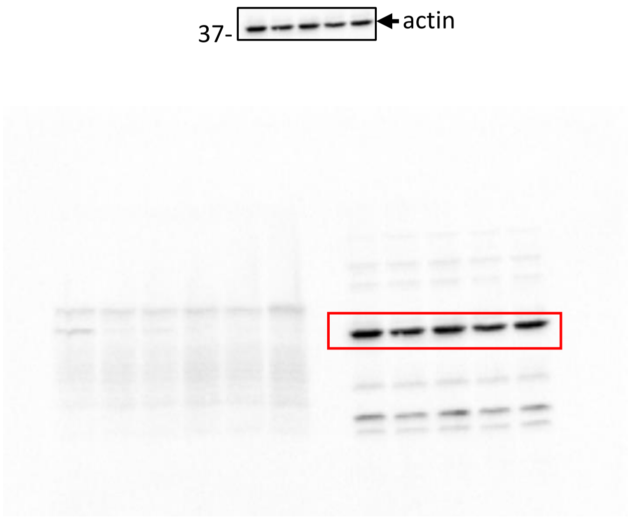
37-
actin

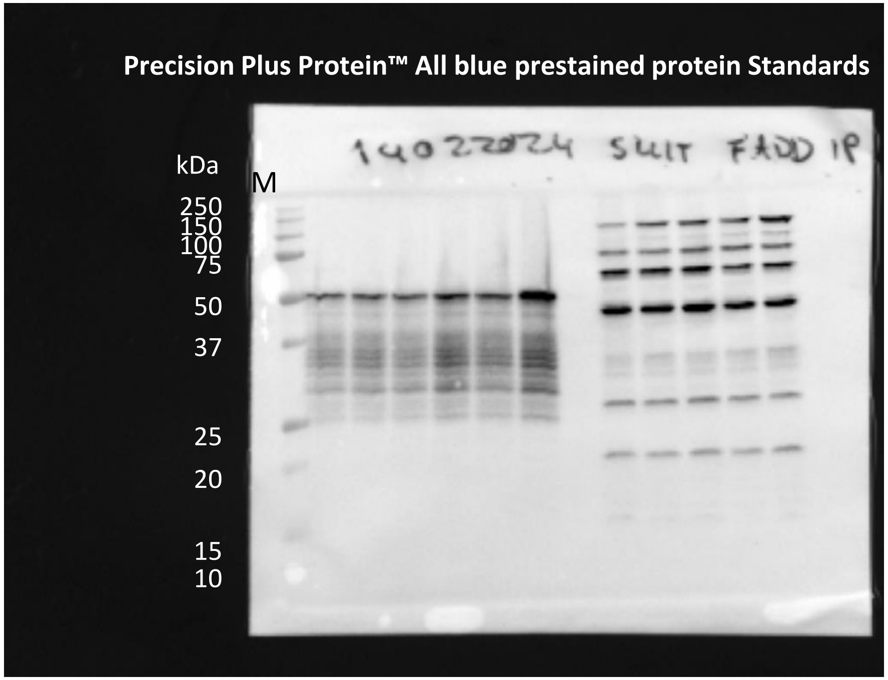
Precision Plus Protein™ All blue prestained protein Standards
kDa
M
250
150
100
75
50
37
25
20
15
10

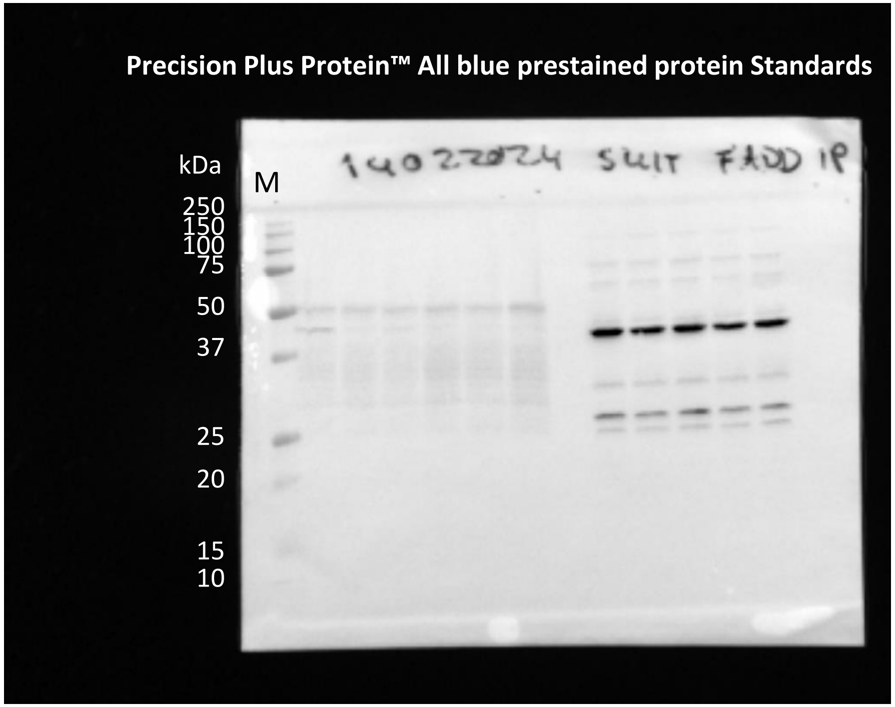
Precision Plus Protein™ All blue prestained protein Standards
kDa
M
250
150
100
75
50
37
25
20
15
10

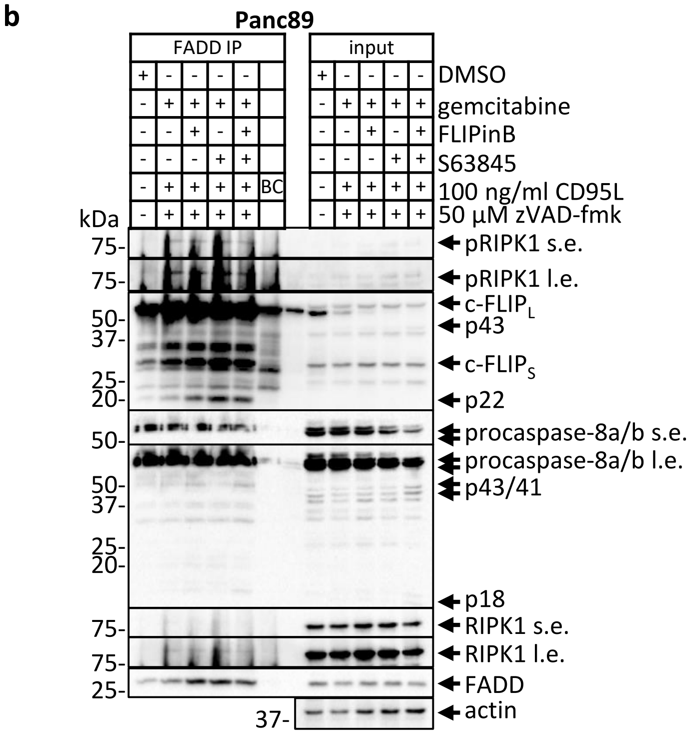
b
Panc89
FADD IP
input
DMSO
gemcitabine
FLIPinB
S63845
100 ng/ml CD95L
50 µM zVAD-fmk
BC
kDa
pRIPK1 s.e.
pRIPK1 l.e.
c-FLIP_L
p43
c-FLIP_S
p22
procaspase-8a/b s.e.
procaspase-8a/b l.e.
p43/41
p18
RIPK1 s.e.
RIPK1 l.e.
FADD
actin

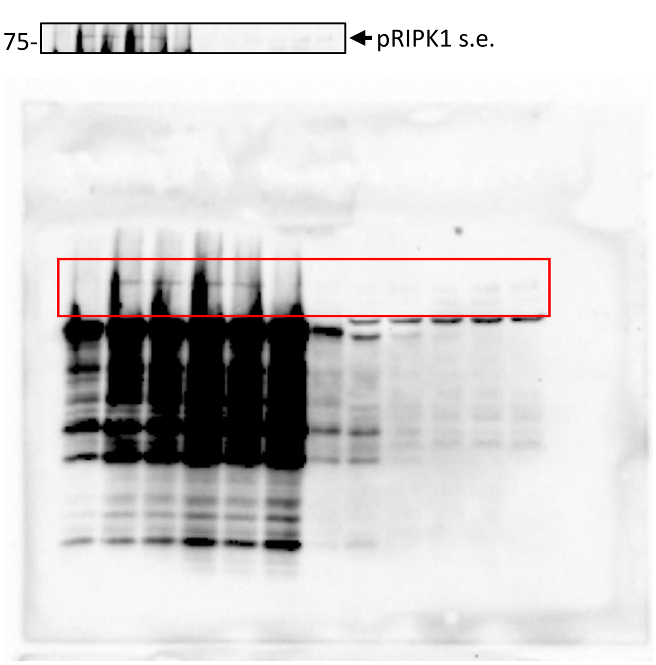
75-
pRIPK1 s.e.

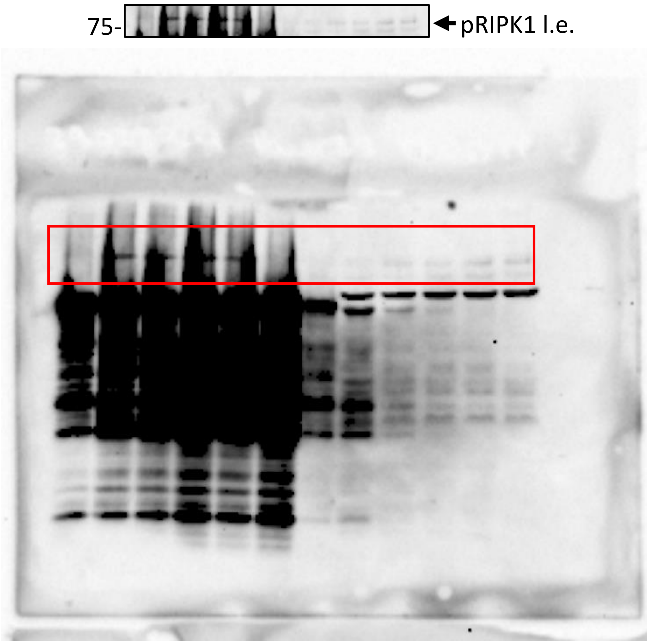
75-
pRIPK1 l.e.

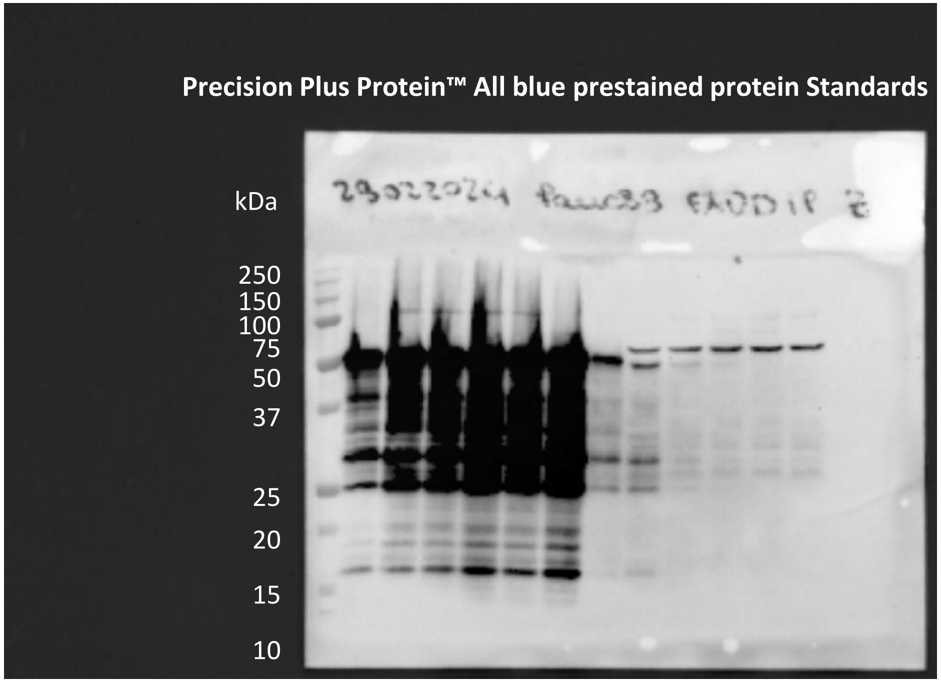
Precision Plus Protein™ All blue prestained protein Standards
kDa
250
150
100
75
50
37
25
20
15
10

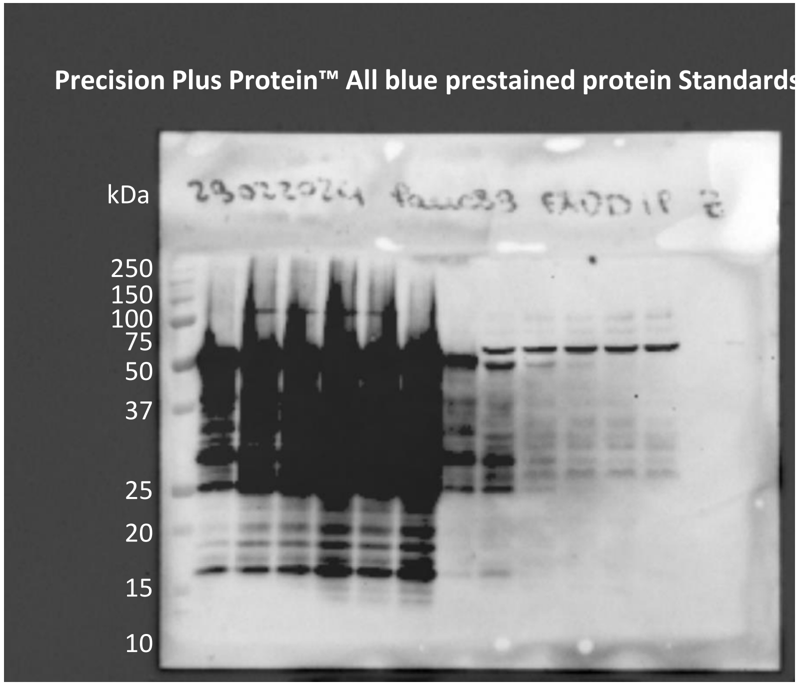
Precision Plus Protein™ All blue prestained protein Standards
kDa
250
150
100
75
50
37
25
20
15
10

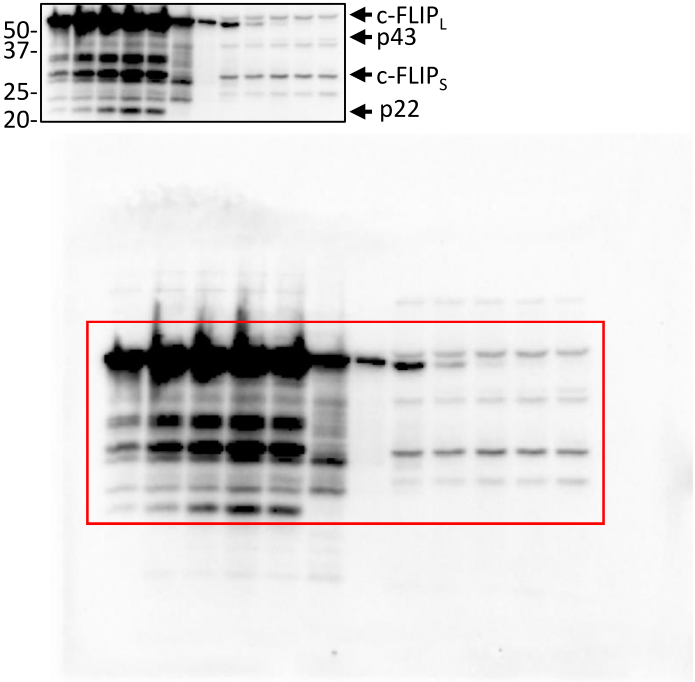
50-
37-
25-
20-
c-FLIP$_L$
p43
c-FLIP$_S$
p22

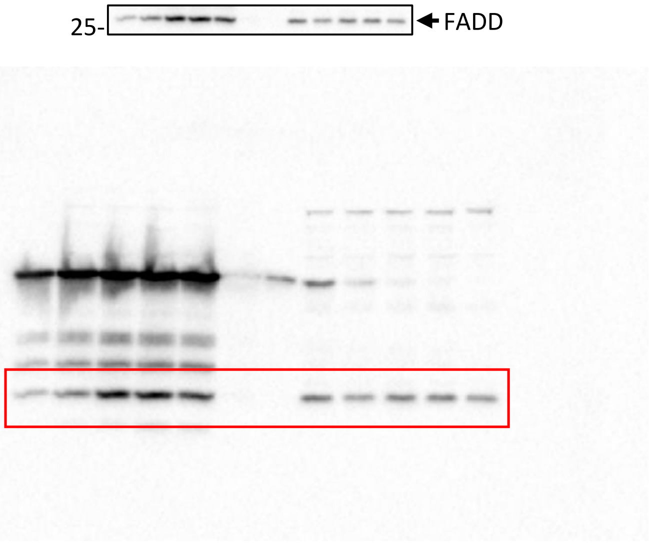
25-
FADD

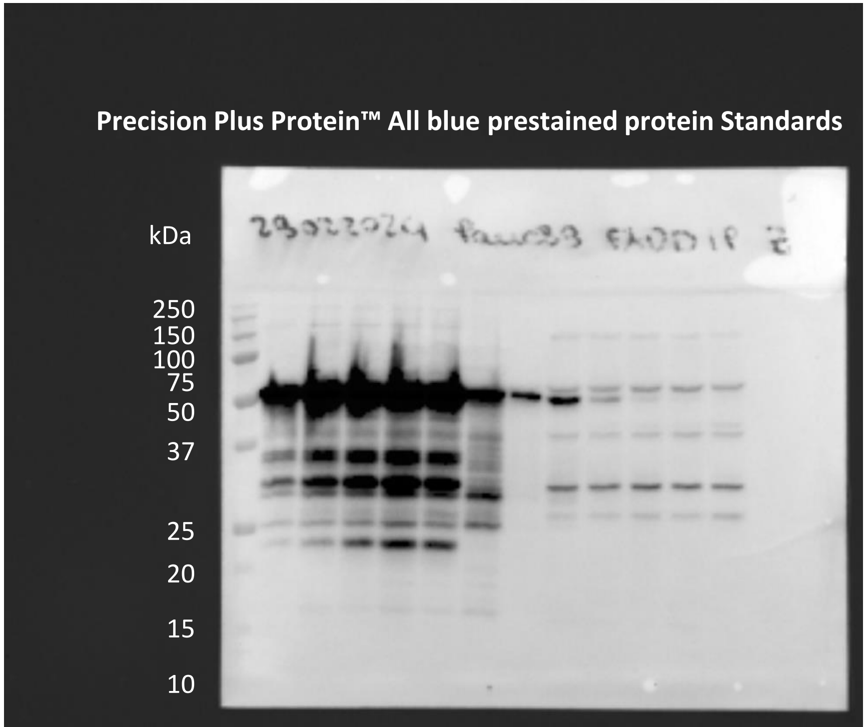
Precision Plus Protein™ All blue prestained protein Standards
kDa
250
150
100
75
50
37
25
20
15
10

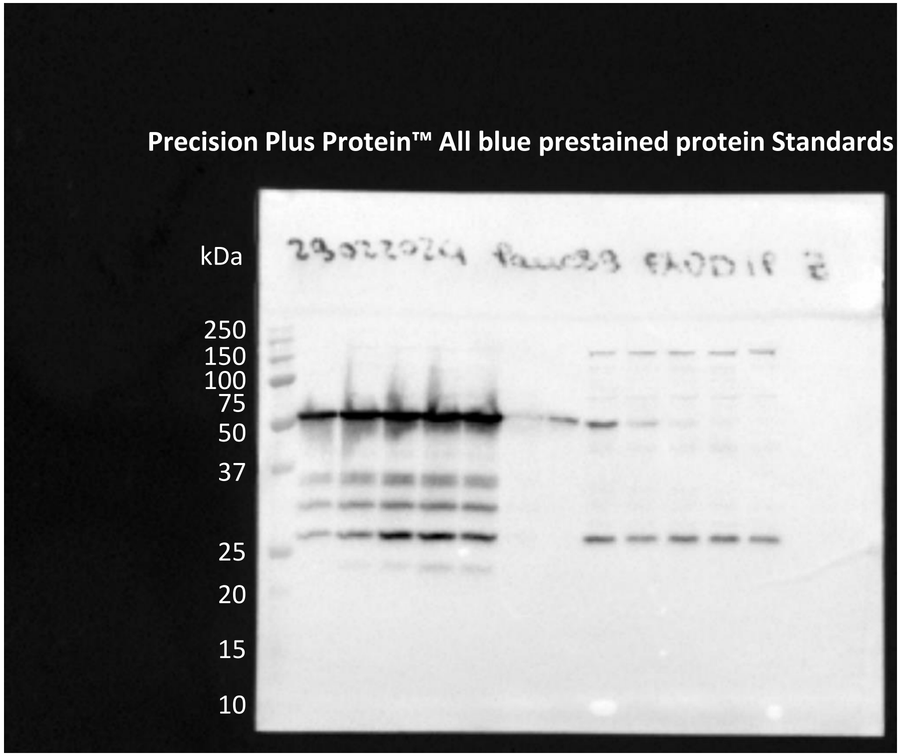
Precision Plus Protein™ All blue prestained protein Standards
kDa
250
150
100
75
50
37
25
20
15
10

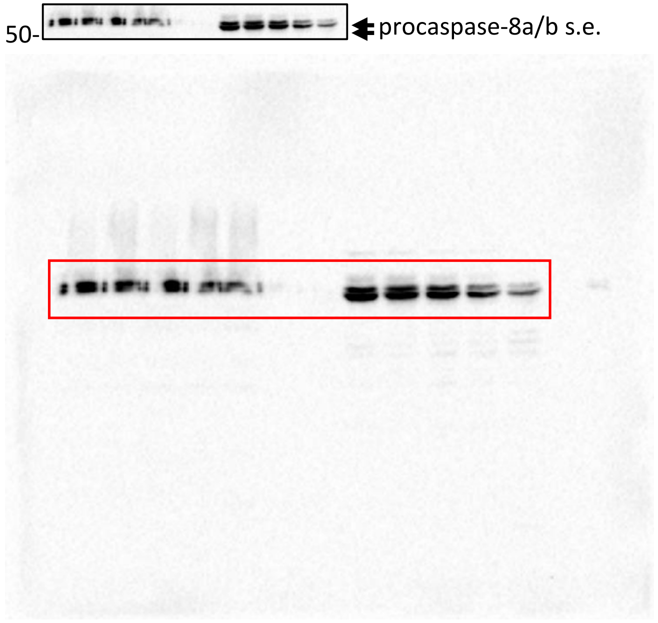
50-
procaspase-8a/b s.e.

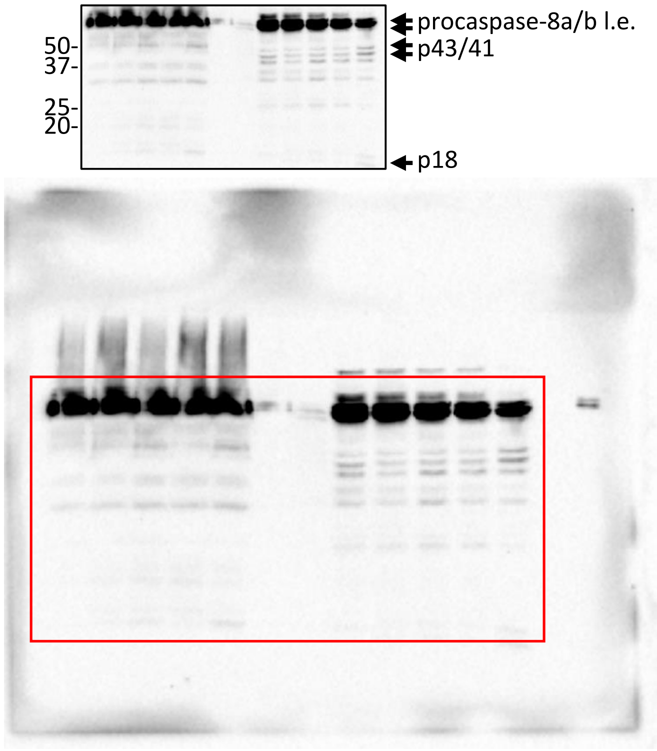
procaspase-8a/b l.e.
p43/41
p18
50-
37-
25-
20-

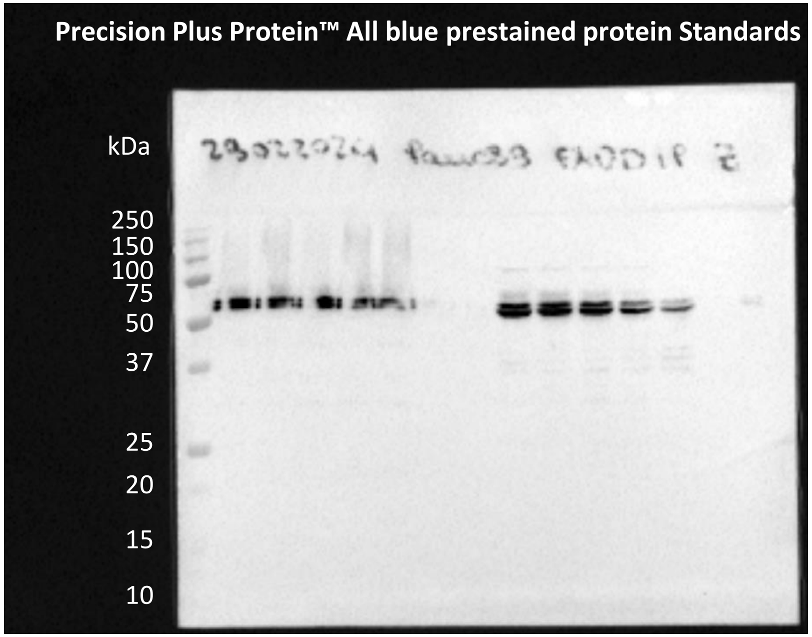
Precision Plus Protein™ All blue prestained protein Standards
kDa
250
150
100
75
50
37
25
20
15
10

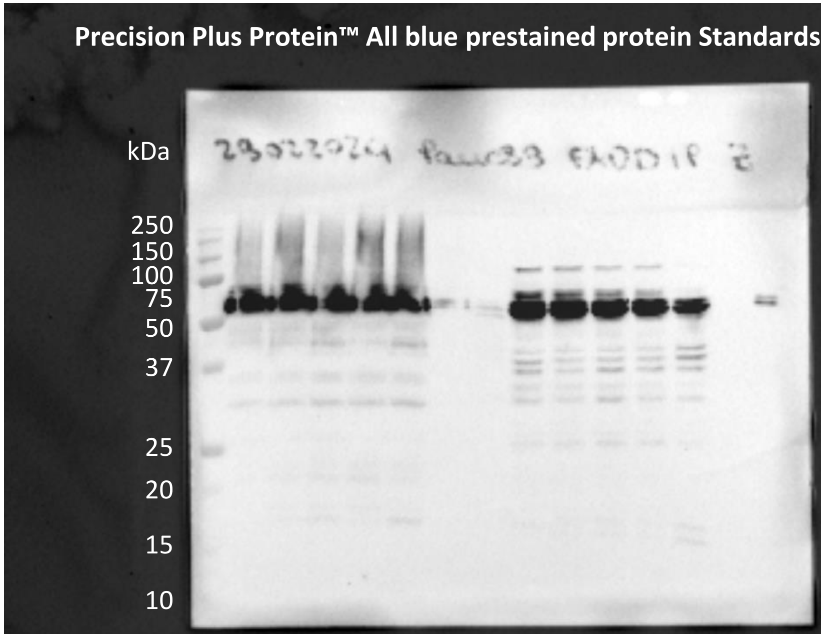
Precision Plus Protein™ All blue prestained protein Standards
kDa
250
150
100
75
50
37
25
20
15
10

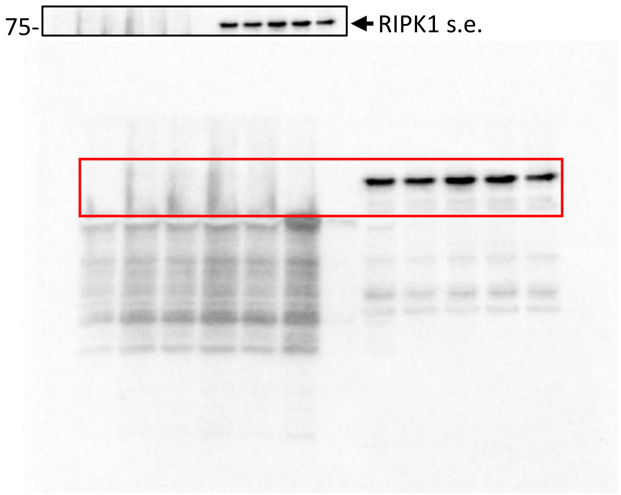
75-
RIPK1 s.e.

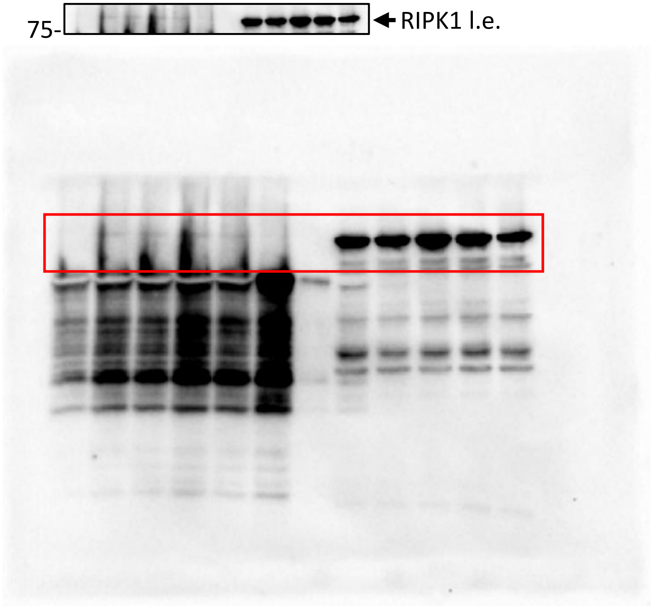
75-
RIPK1 l.e.

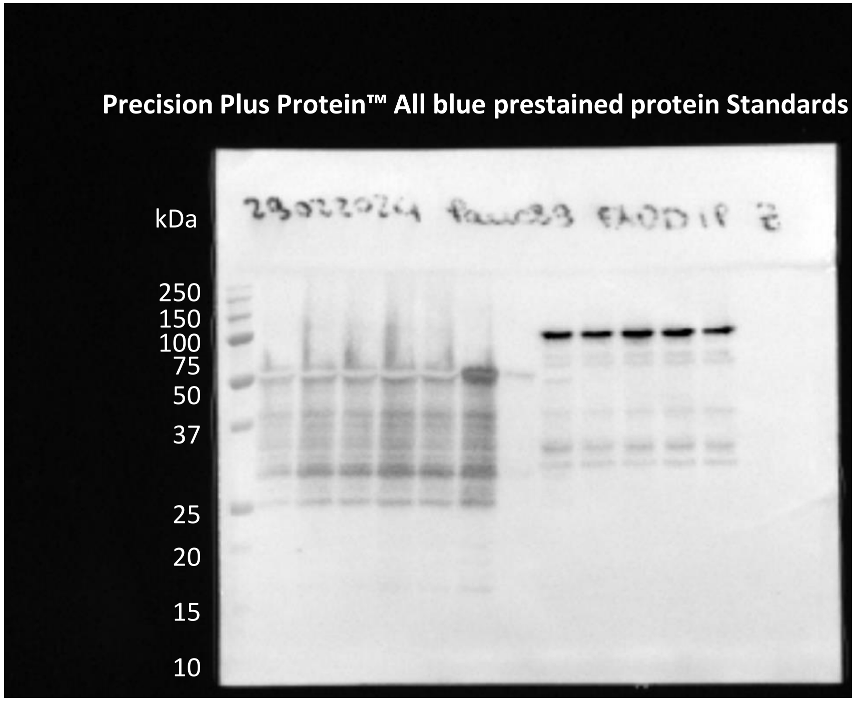
Precision Plus Protein™ All blue prestained protein Standards
kDa
250
150
100
75
50
37
25
20
15
10

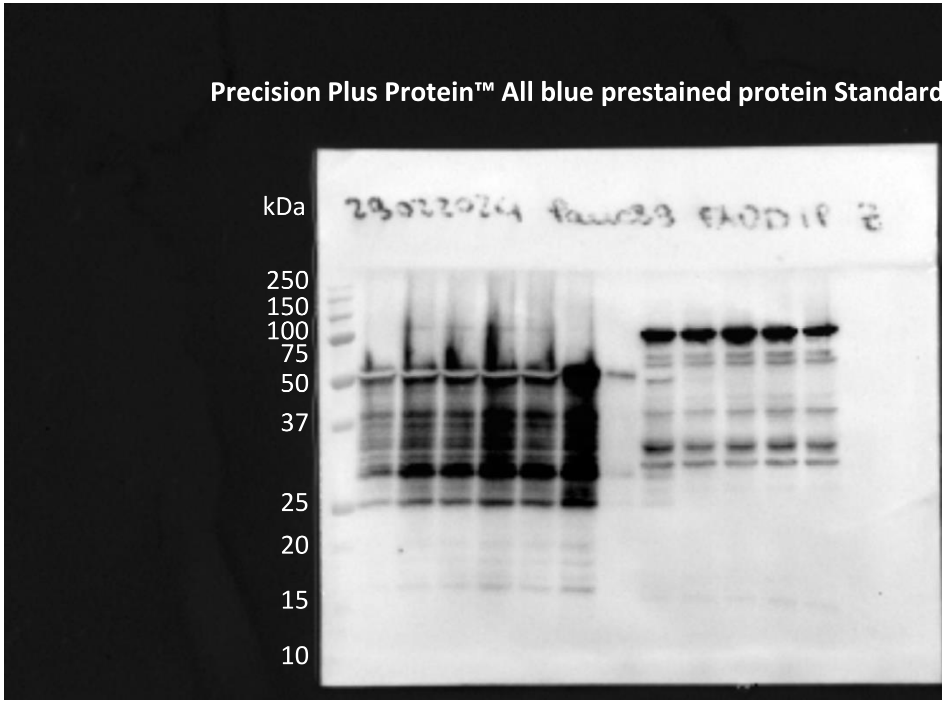
Precision Plus Protein™ All blue prestained protein Standard
kDa
250
150
100
75
50
37
25
20
15
10

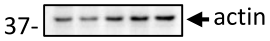
37-
actin

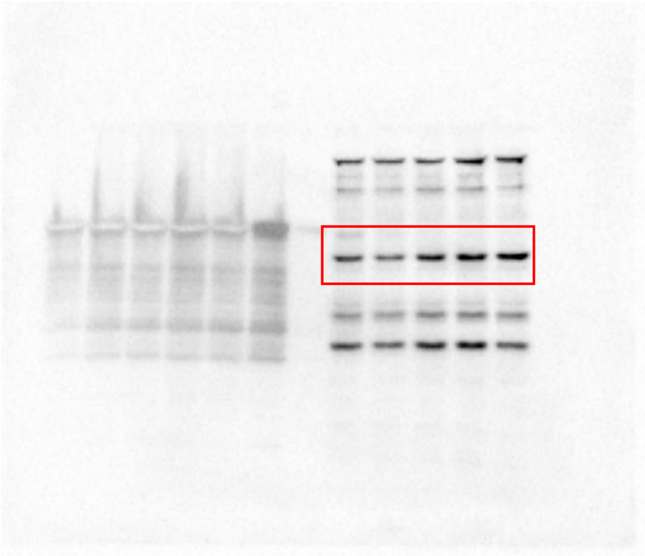

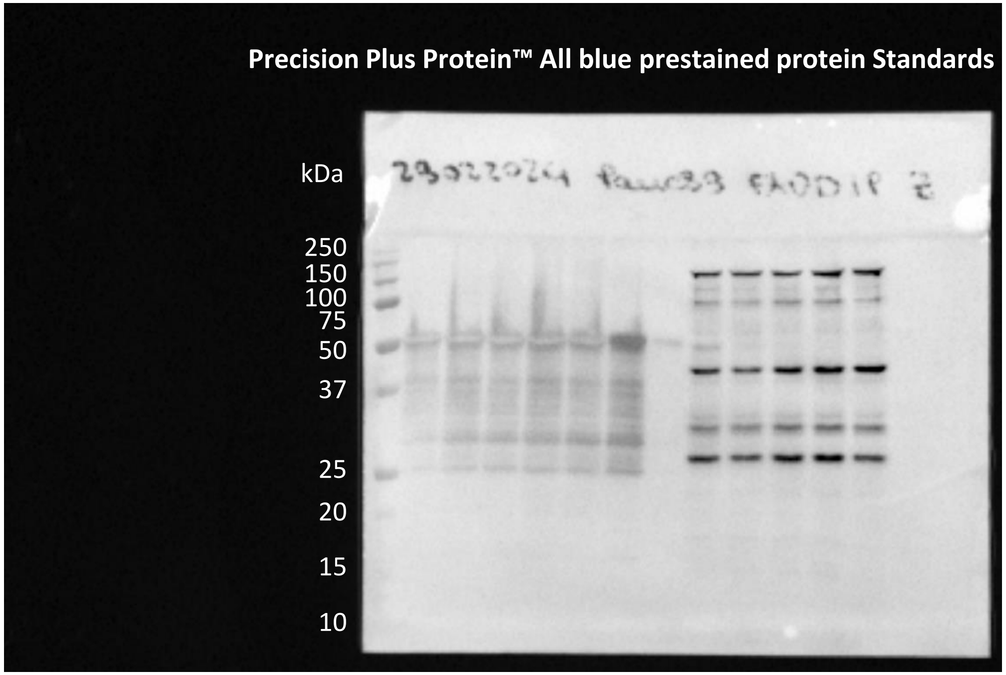
Precision Plus Protein™ All blue prestained protein Standards
kDa
250
150
100
75
50
37
25
20
15
10

c

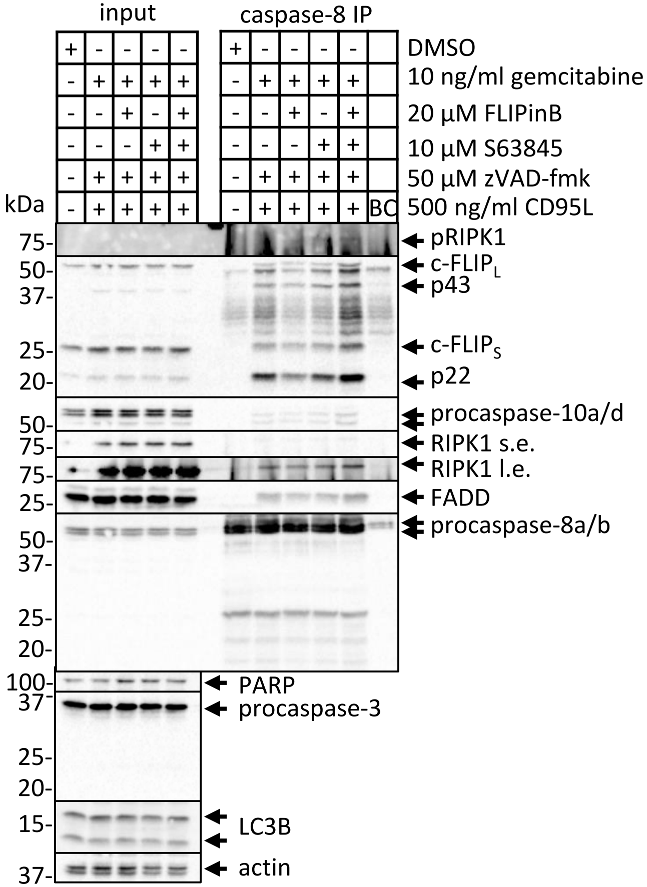

input
caspase-8 IP
DMSO
10 ng/ml gemcitabine
20 µM FLIPinB
10 µM S63845
50 µM zVAD-fmk
500 ng/ml CD95L
BC
kDa
75-
50-
37-
25-
20-
50-
75-
75-
25-
50-
37-
25-
20-
100-
37-
25-
20-
15-
37-
pRIPK1
c-FLIPL
p43
c-FLIPS
p22
procaspase-10a/d
RIPK1 s.e.
RIPK1 l.e.
FADD
procaspase-8a/b
PARP
procaspase-3
LC3B
actin

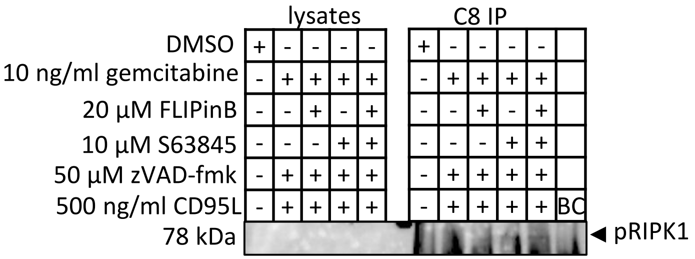
lysates
C8 IP
DMSO
10 ng/ml gemcitabine
20 µM FLIPinB
10 µM S63845
50 µM zVAD-fmk
500 ng/ml CD95L
BC
78 kDa
◀ pRIPK1

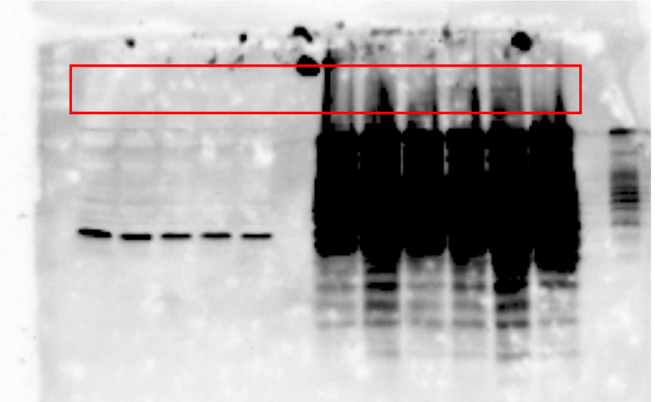

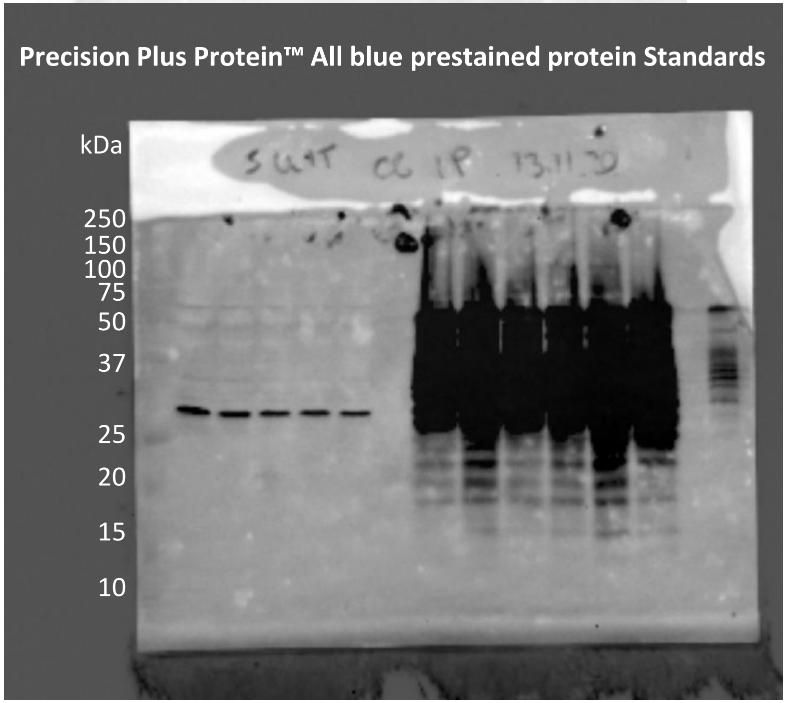
Precision Plus Protein™ All blue prestained protein Standards
kDa
250
150
100
75
50
37
25
20
15
10

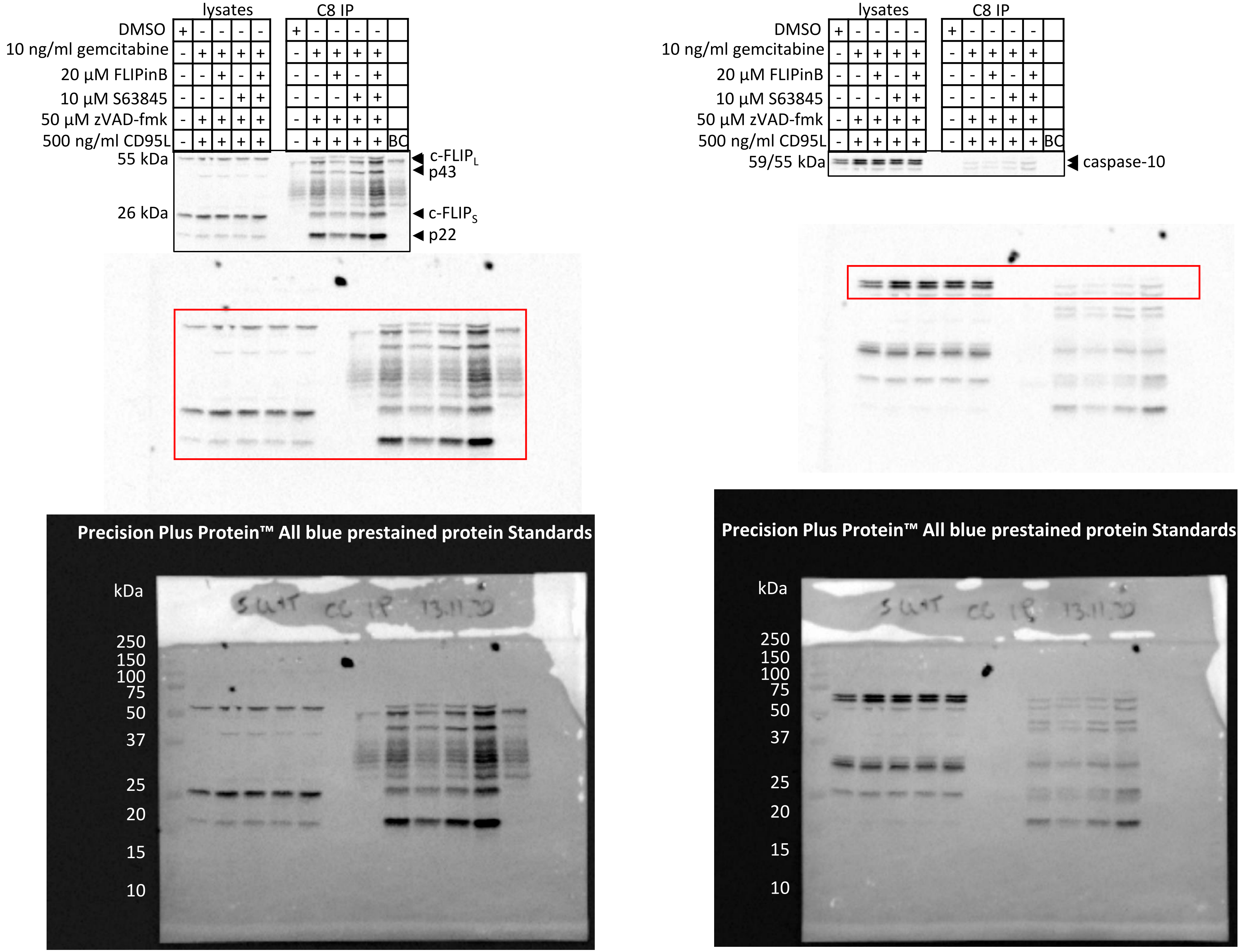

lysates
C8 IP
DMSO
10 ng/ml gemcitabine
20 µM FLIPinB
10 µM S63845
50 µM zVAD-fmk
500 ng/ml CD95L
BC
55 kDa
26 kDa
c-FLIPL
p43
c-FLIPS
p22
59/55 kDa
caspase-10
Precision Plus Protein™ All blue prestained protein Standards
kDa
250
150
100
75
50
37
25
20
15
10

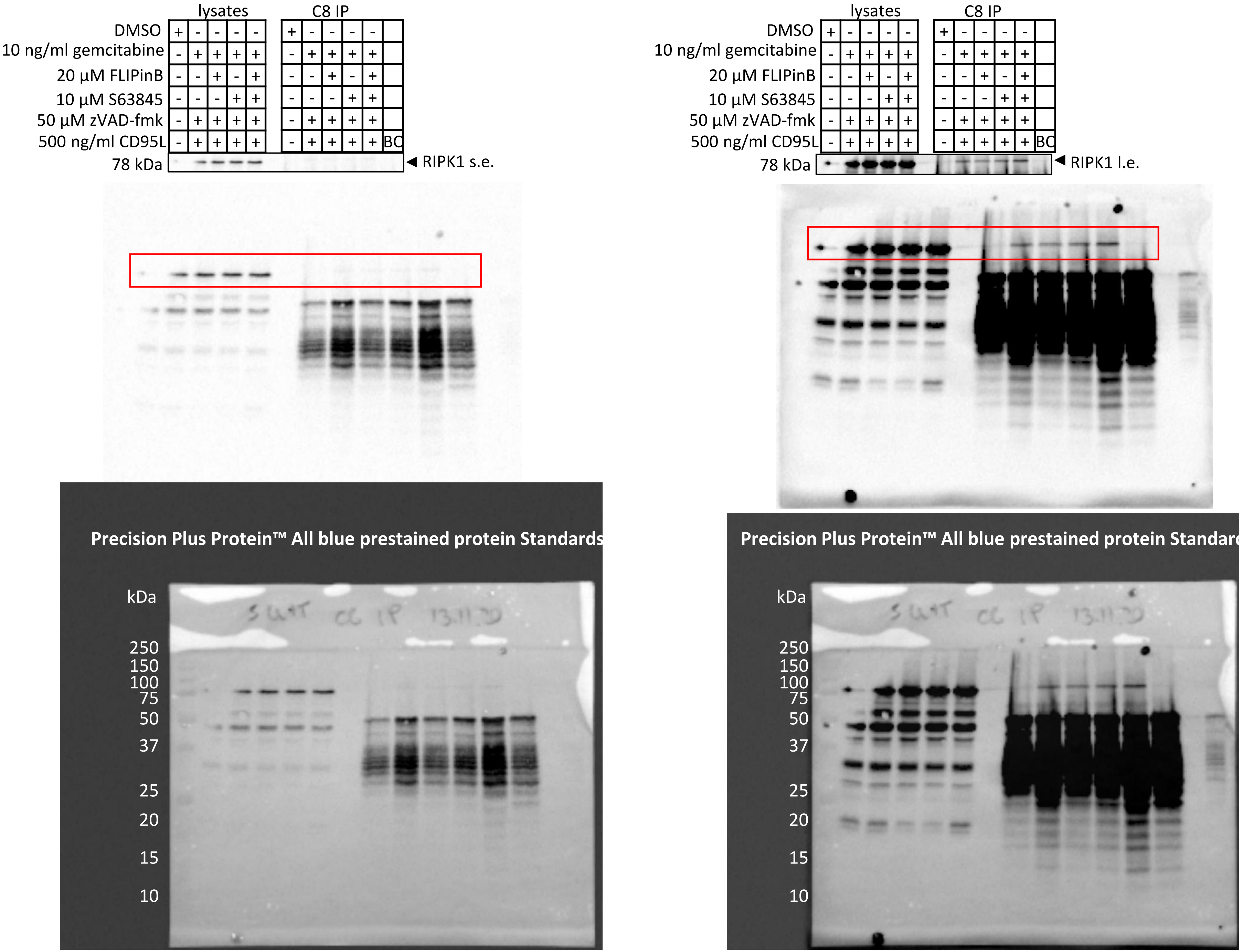

lysates
C8 IP
DMSO
10 ng/ml gemcitabine
20 µM FLIPinB
10 µM S63845
50 µM zVAD-fmk
500 ng/ml CD95L
BC
78 kDa
RIPK1 s.e.
RIPK1 l.e.
Precision Plus Protein™ All blue prestained protein Standards
kDa
250
150
100
75
50
37
25
20
15
10

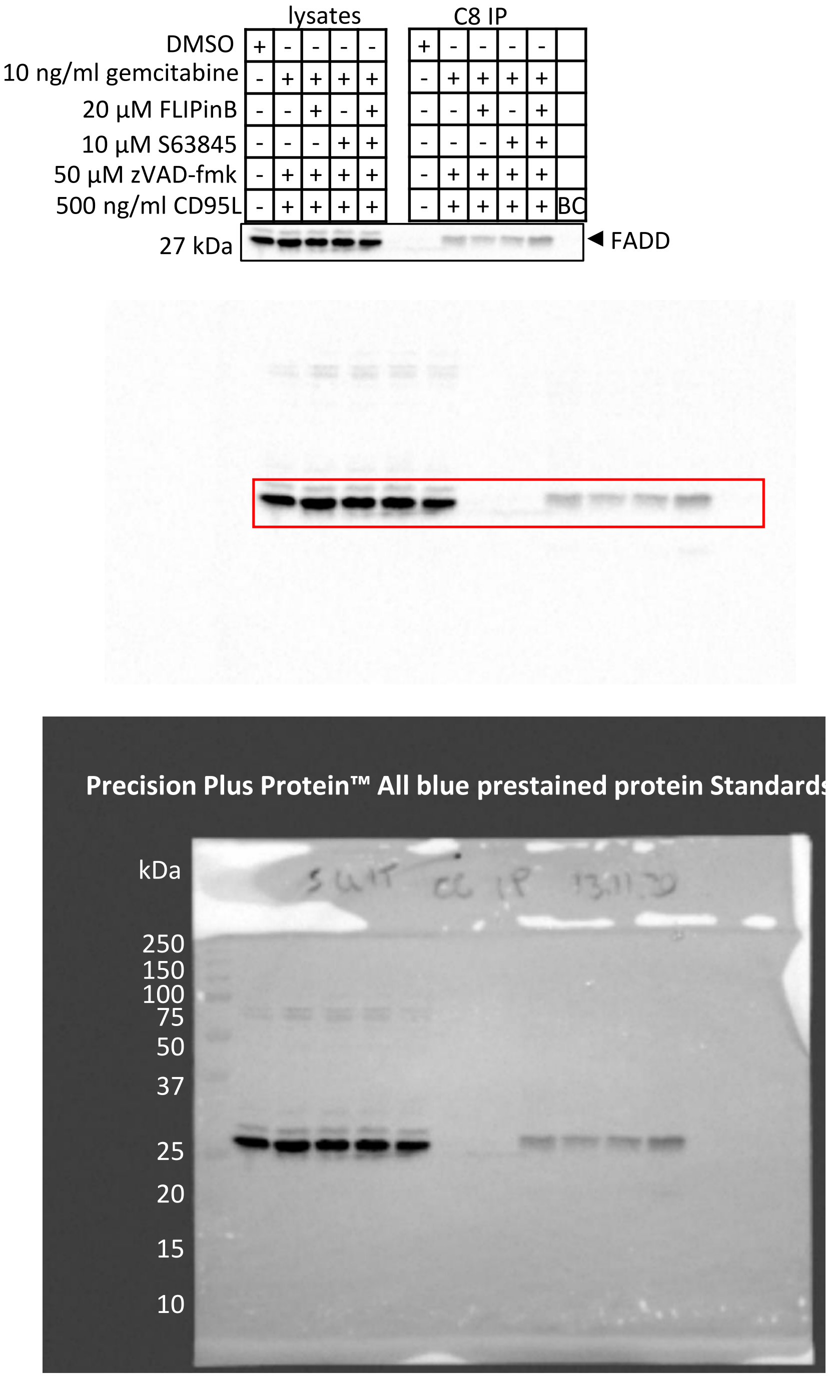
lysates
C8 IP
DMSO
10 ng/ml gemcitabine
20 µM FLIPinB
10 µM S63845
50 µM zVAD-fmk
500 ng/ml CD95L
BC
27 kDa
FADD
Precision Plus Protein™ All blue prestained protein Standards
kDa
250
150
100
75
50
37
25
20
15
10

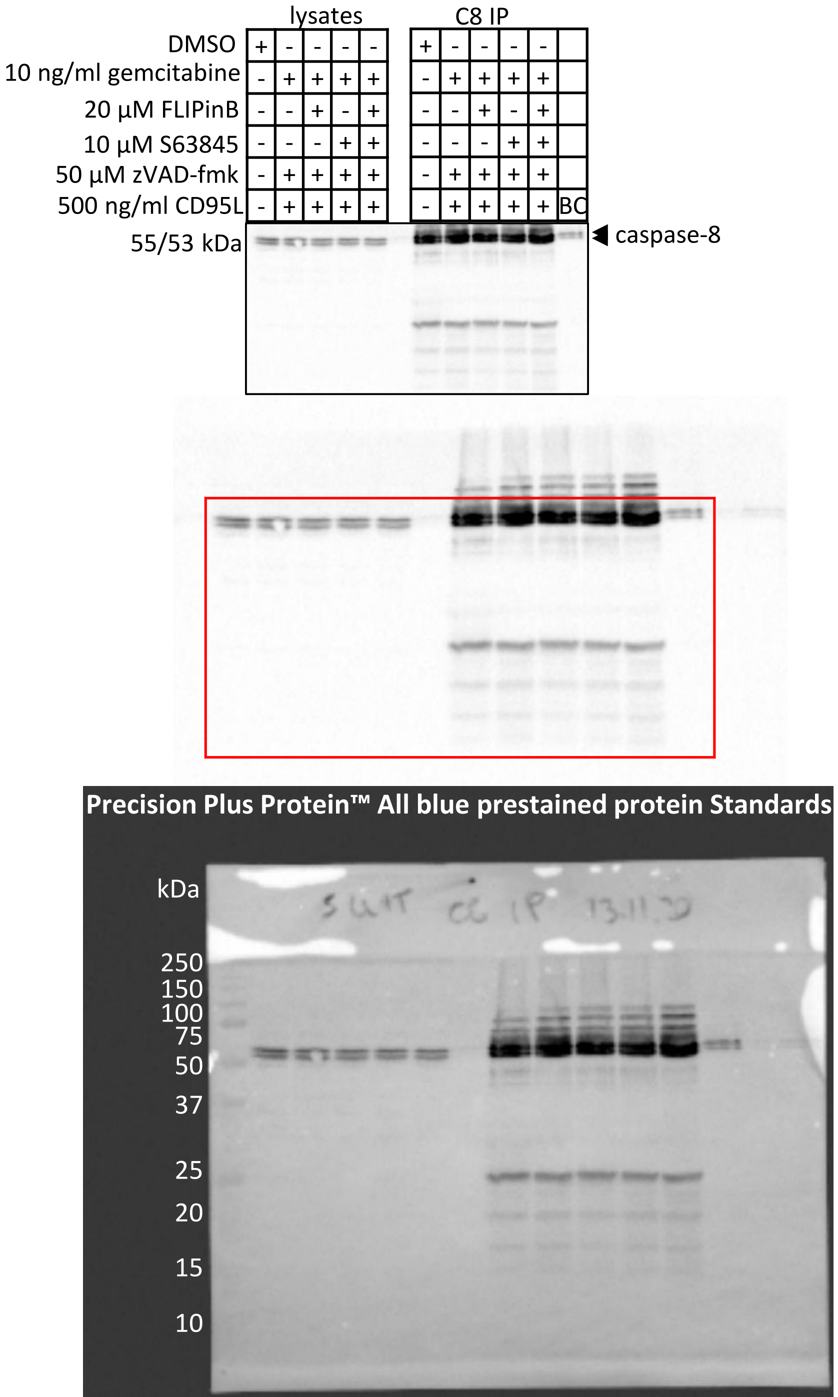
lysates
C8 IP
DMSO
10 ng/ml gemcitabine
20 µM FLIPinB
10 µM S63845
50 µM zVAD-fmk
500 ng/ml CD95L
BC
55/53 kDa
caspase-8
Precision Plus Protein™ All blue prestained protein Standards
kDa
250
150
100
75
50
37
25
20
15
10

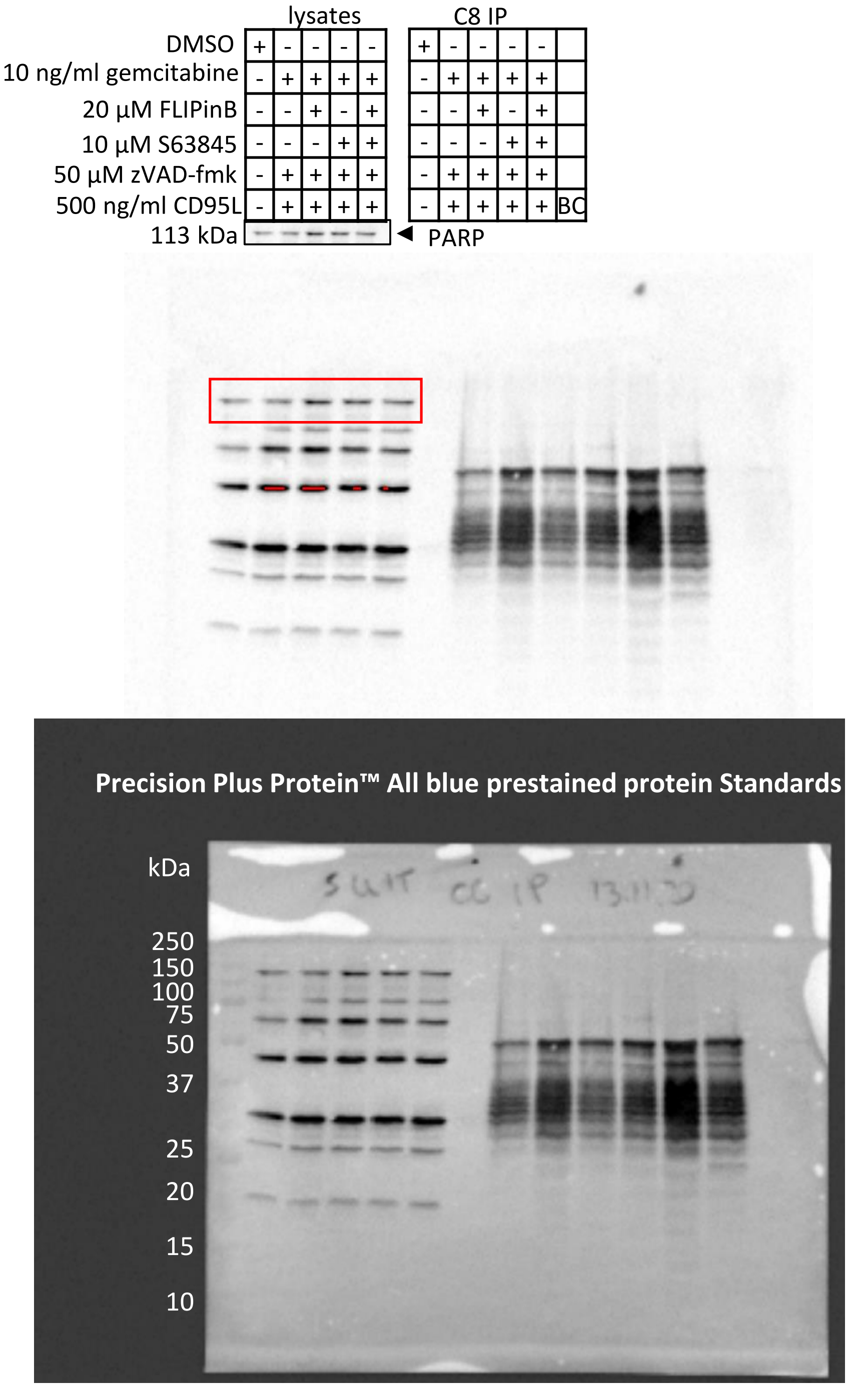
lysates
C8 IP
DMSO
10 ng/ml gemcitabine
20 µM FLIPinB
10 µM S63845
50 µM zVAD-fmk
500 ng/ml CD95L
BC
113 kDa
PARP
Precision Plus Protein™ All blue prestained protein Standards
kDa
250
150
100
75
50
37
25
20
15
10

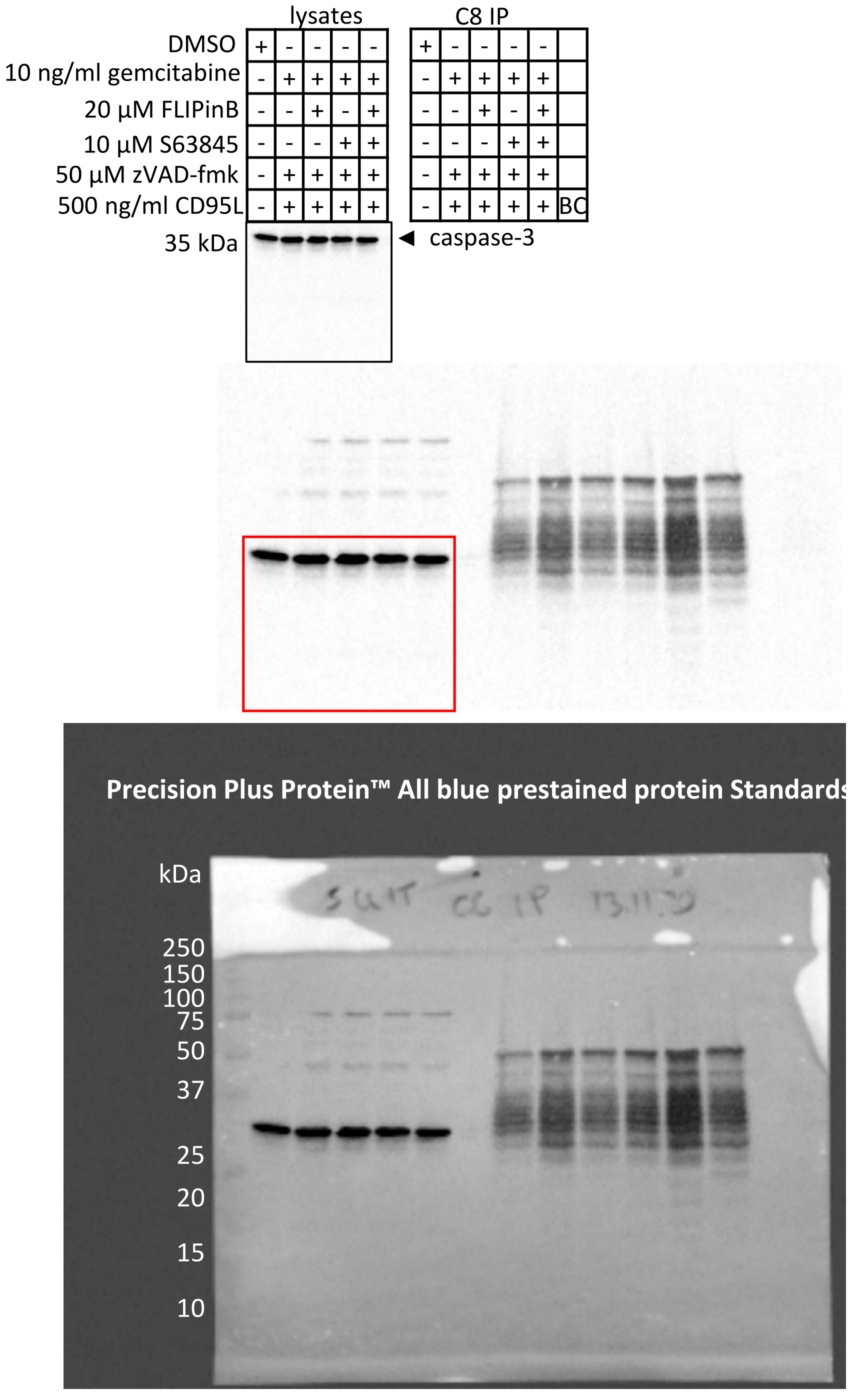
lysates
C8 IP
DMSO
10 ng/ml gemcitabine
20 µM FLIPinB
10 µM S63845
50 µM zVAD-fmk
500 ng/ml CD95L
BC
35 kDa
caspase-3
Precision Plus Protein™ All blue prestained protein Standards
kDa
250
150
100
75
50
37
25
20
15
10

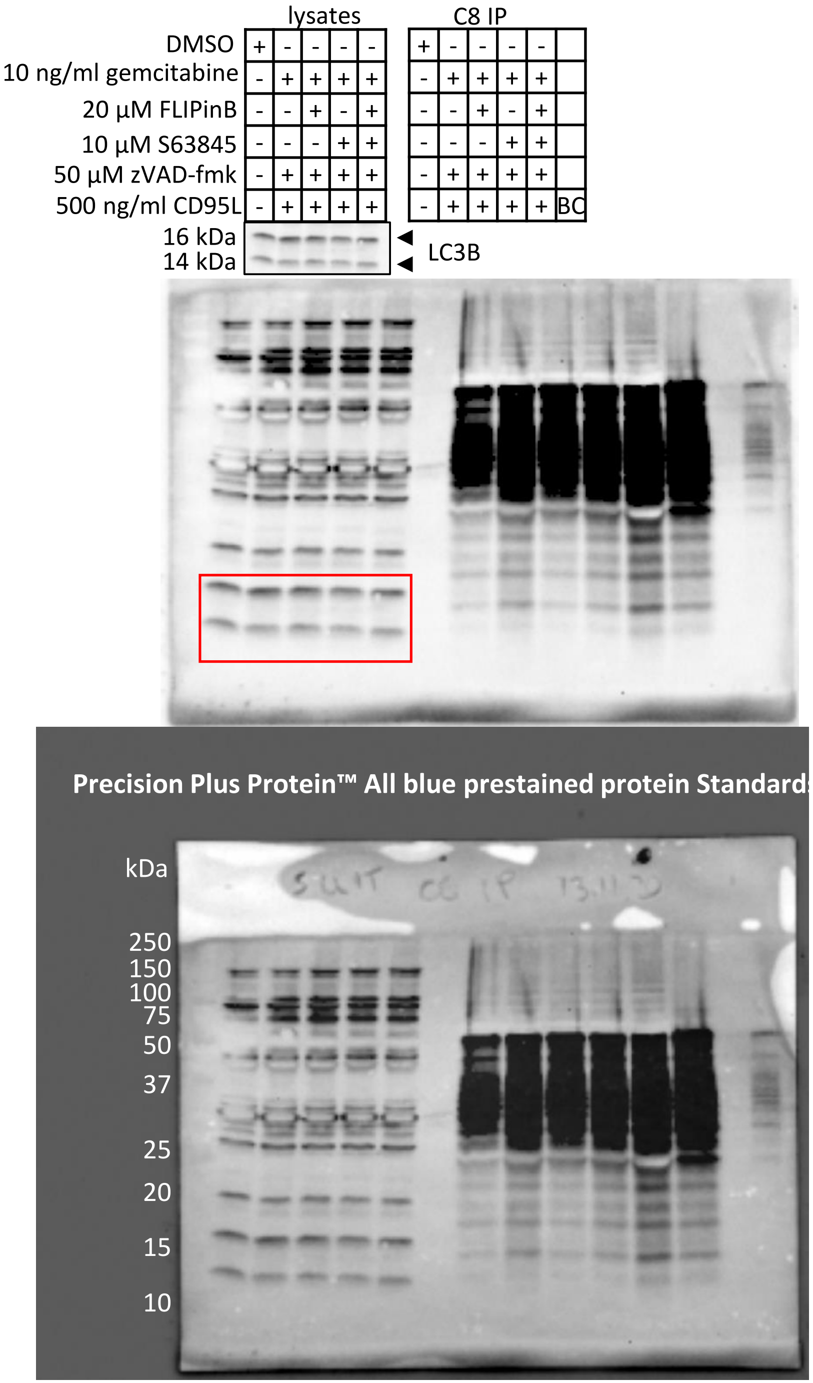
lysates
C8 IP
DMSO
10 ng/ml gemcitabine
20 µM FLIPinB
10 µM S63845
50 µM zVAD-fmk
500 ng/ml CD95L
BC
16 kDa
14 kDa
LC3B
Precision Plus Protein™ All blue prestained protein Standards
kDa
250
150
100
75
50
37
25
20
15
10

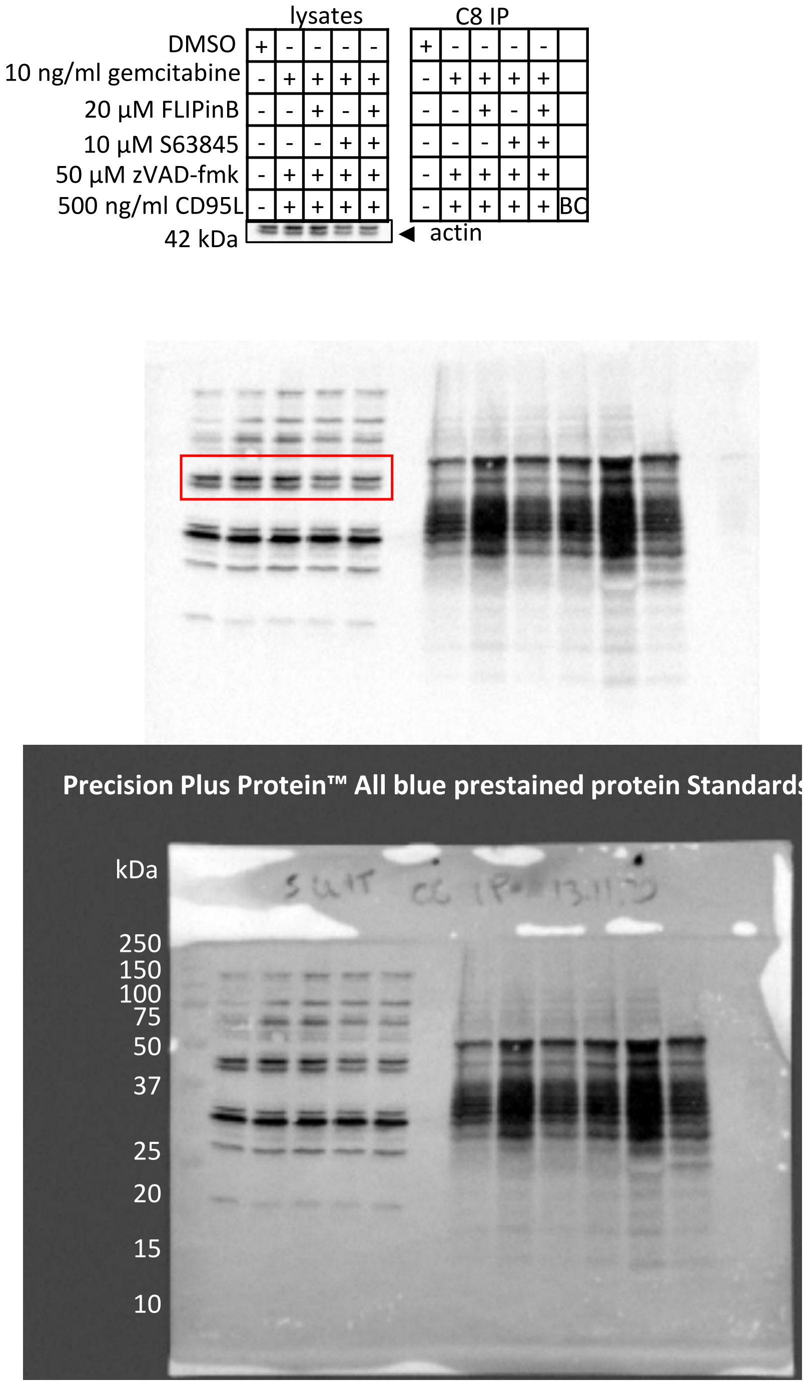
lysates
C8 IP
DMSO
10 ng/ml gemcitabine
20 µM FLIPinB
10 µM S63845
50 µM zVAD-fmk
500 ng/ml CD95L
BC
42 kDa
actin
Precision Plus Protein™ All blue prestained protein Standards
kDa
250
150
100
75
50
37
25
20
15
10

d

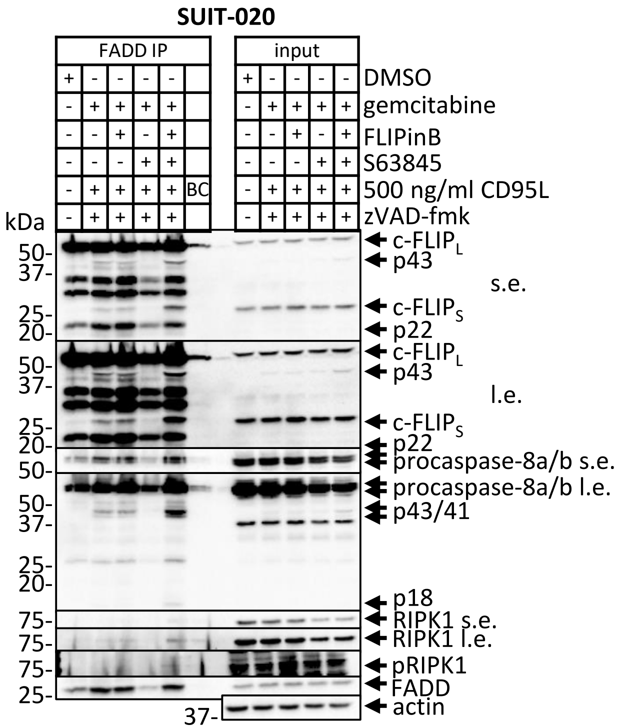
SUIT-020
FADD IP
input
DMSO
gemcitabine
FLIPinB
S63845
500 ng/ml CD95L
zVAD-fmk
BC
kDa
50-
37-
25-
20-
75-
c-FLIPL
p43
s.e.
c-FLIPS
p22
l.e.
procaspase-8a/b s.e.
procaspase-8a/b l.e.
p43/41
p18
RIPK1 s.e.
RIPK1 l.e.
pRIPK1
FADD
actin

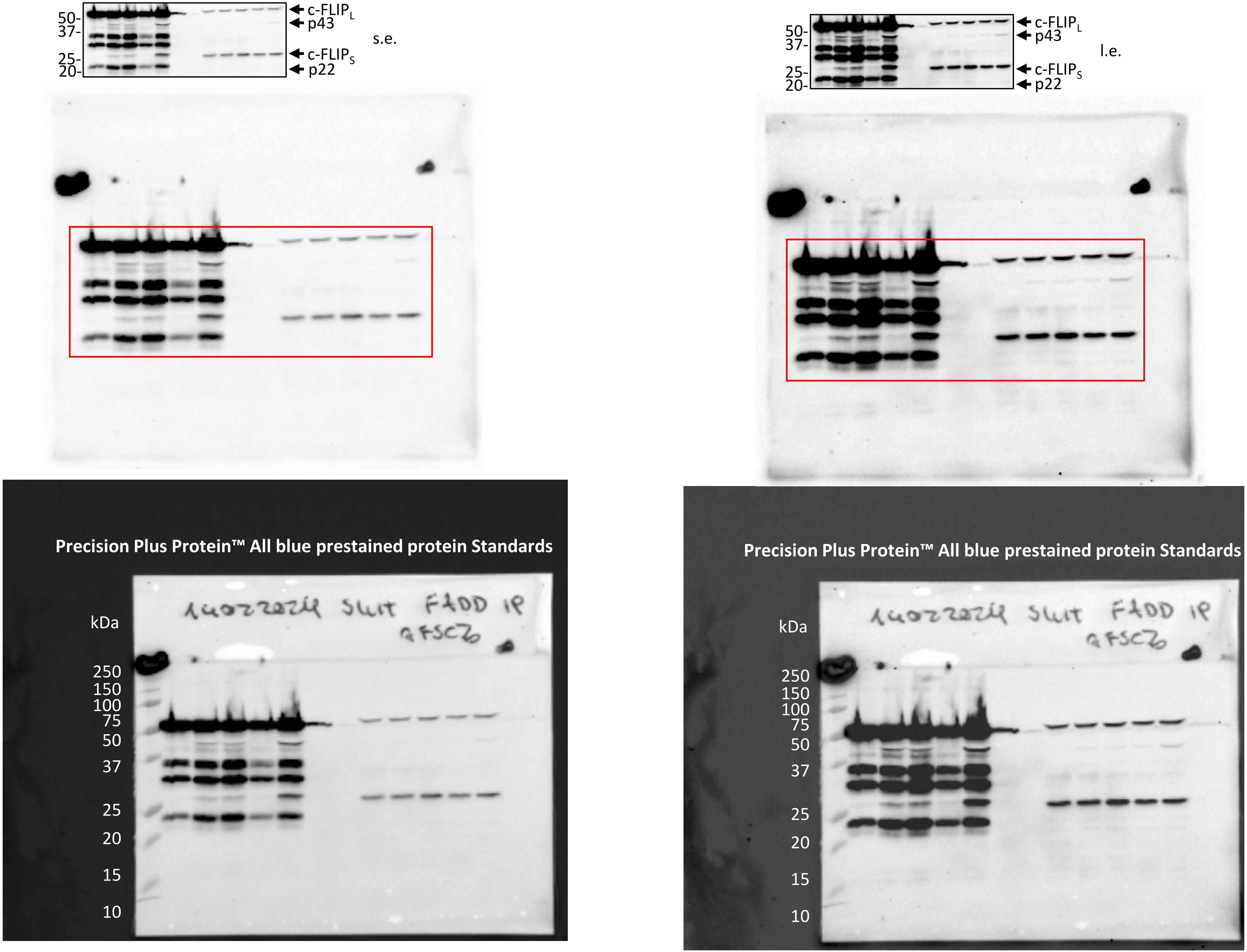
50-
37-
25-
20-
c-FLIP$_L$
p43
c-FLIP$_S$
p22
s.e.
l.e.
Precision Plus Protein™ All blue prestained protein Standards
kDa
250
150
100
75
50
37
25
20
15
10

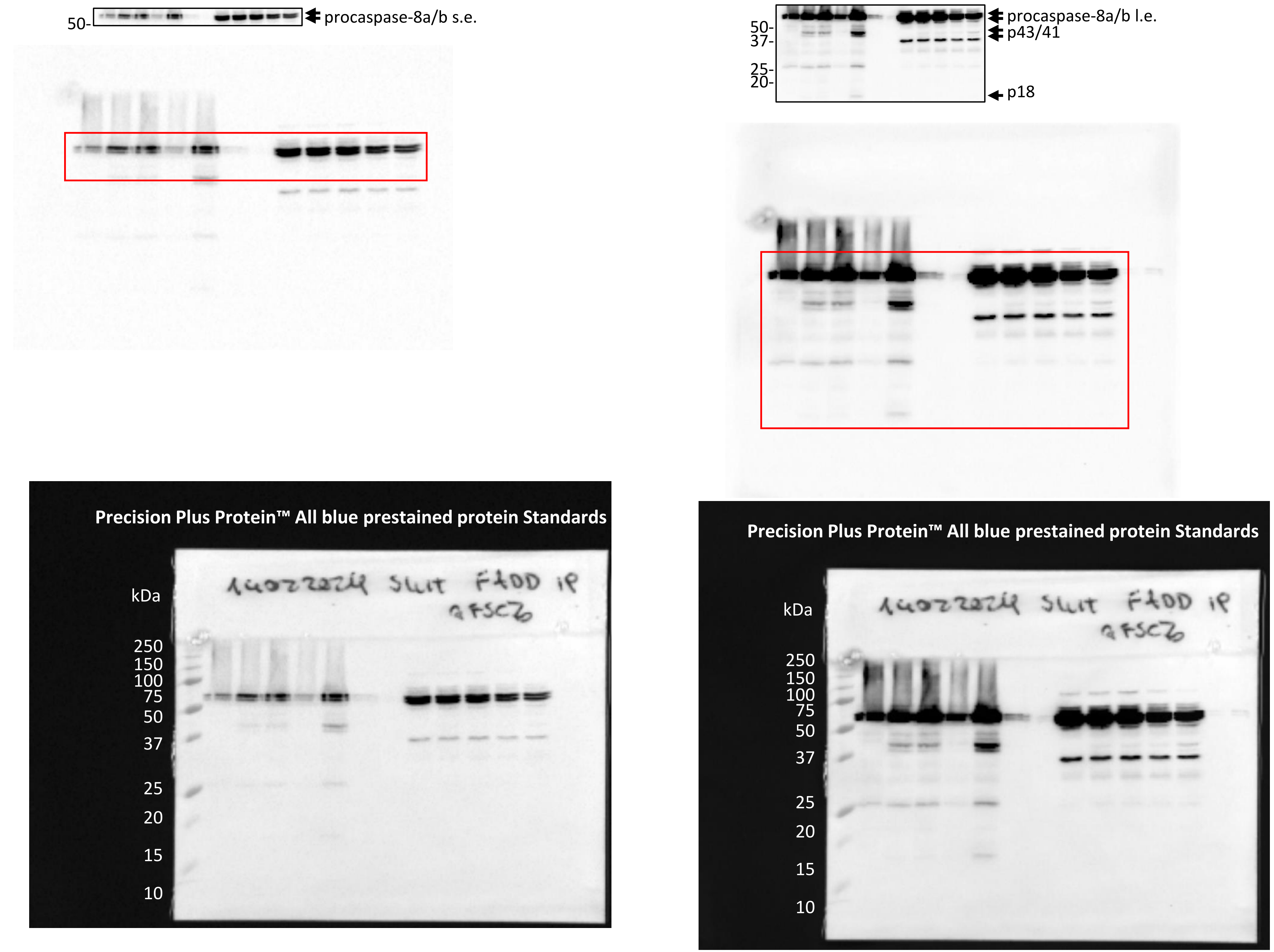
50-
procaspase-8a/b s.e.
50-
37-
25-
20-
procaspase-8a/b l.e.
p43/41
p18
Precision Plus Protein™ All blue prestained protein Standards
kDa
250
150
100
75
50
37
25
20
15
10
Precision Plus Protein™ All blue prestained protein Standards
kDa
250
150
100
75
50
37
25
20
15
10

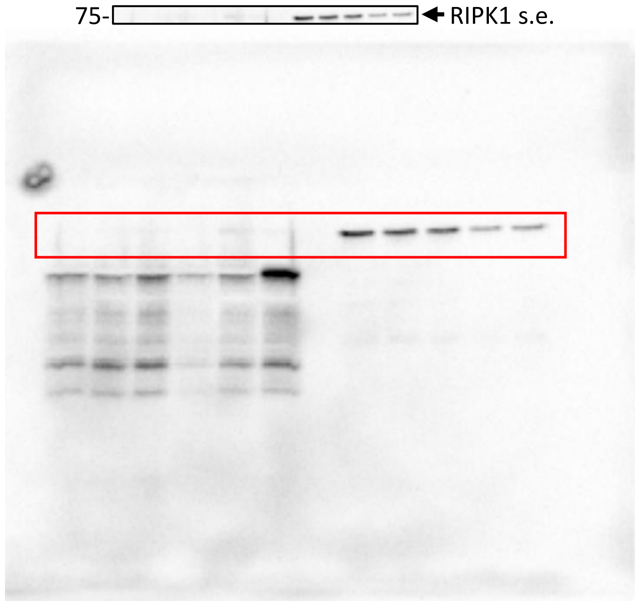
75-
RIPK1 s.e.

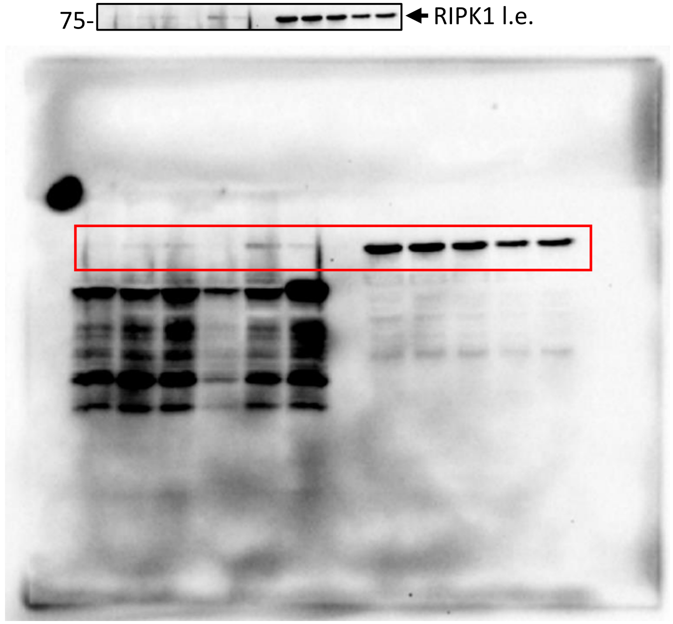
75-
RIPK1 l.e.

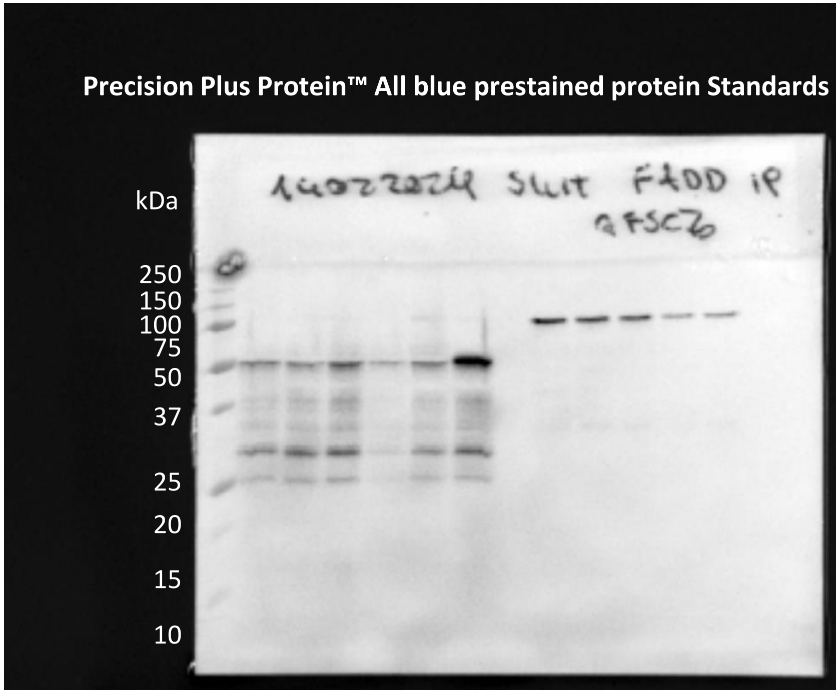
Precision Plus Protein™ All blue prestained protein Standards
kDa
250
150
100
75
50
37
25
20
15
10

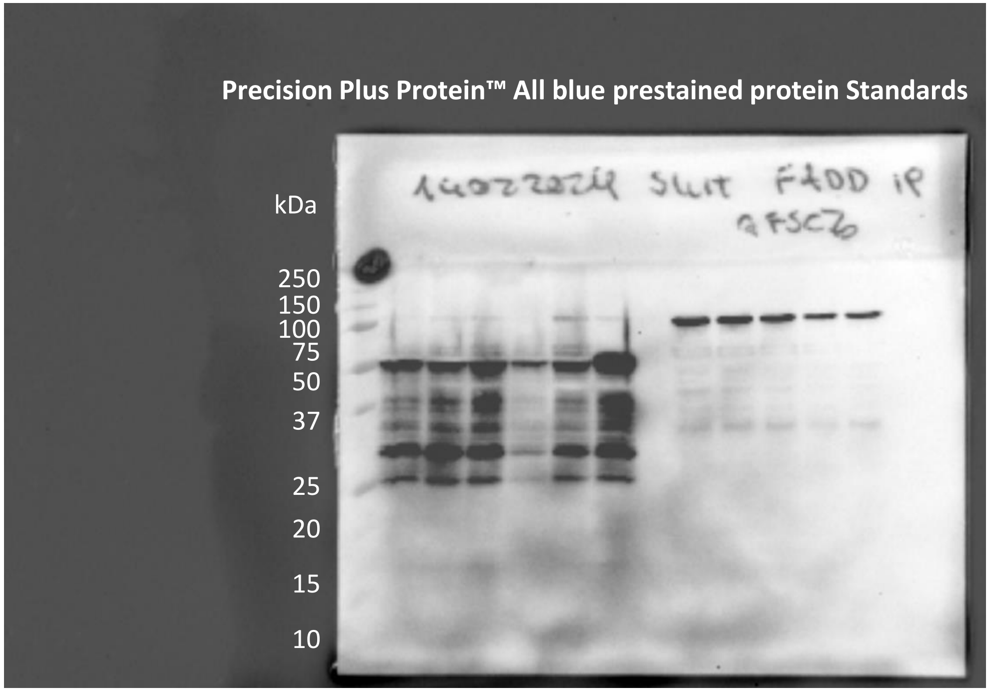
Precision Plus Protein™ All blue prestained protein Standards
kDa
250
150
100
75
50
37
25
20
15
10

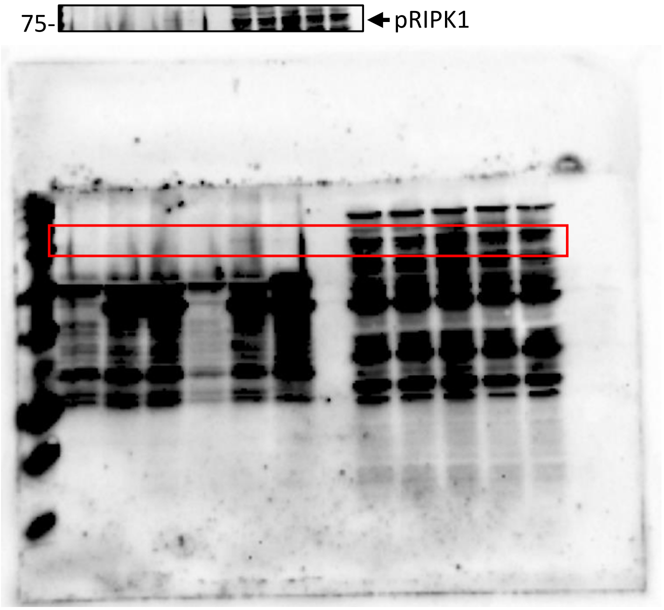
75-
pRIPK1

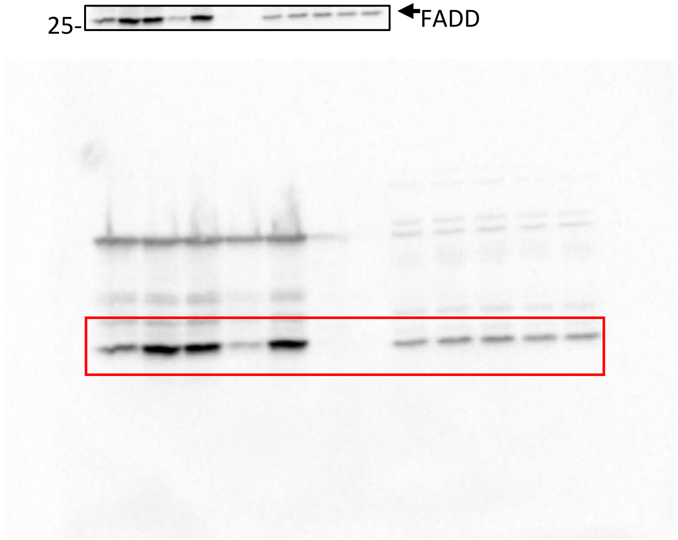
25-
FADD

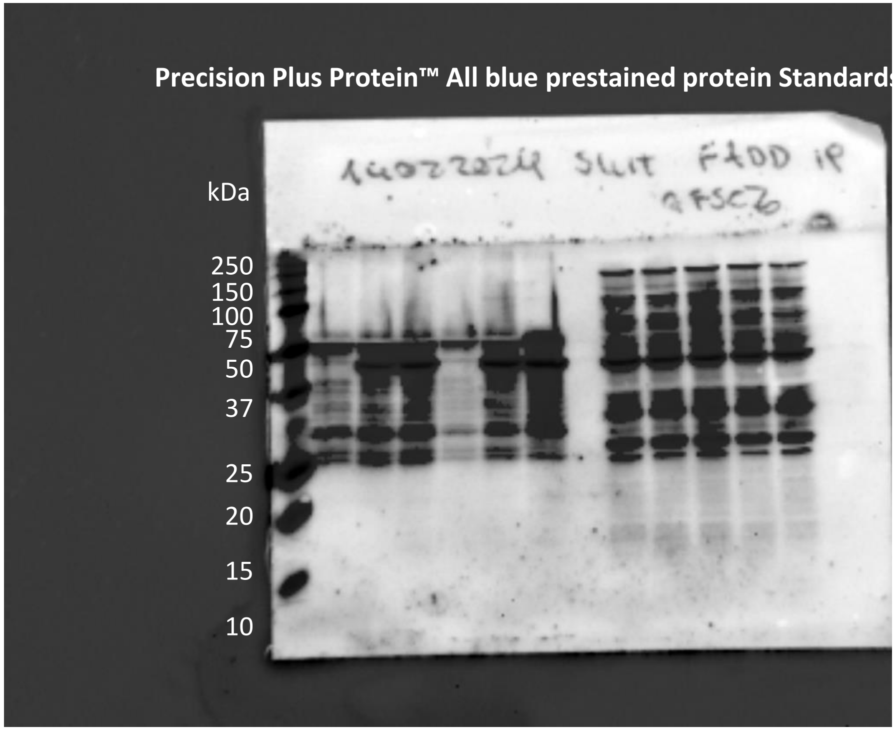
Precision Plus Protein™ All blue prestained protein Standards
kDa
250
150
100
75
50
37
25
20
15
10

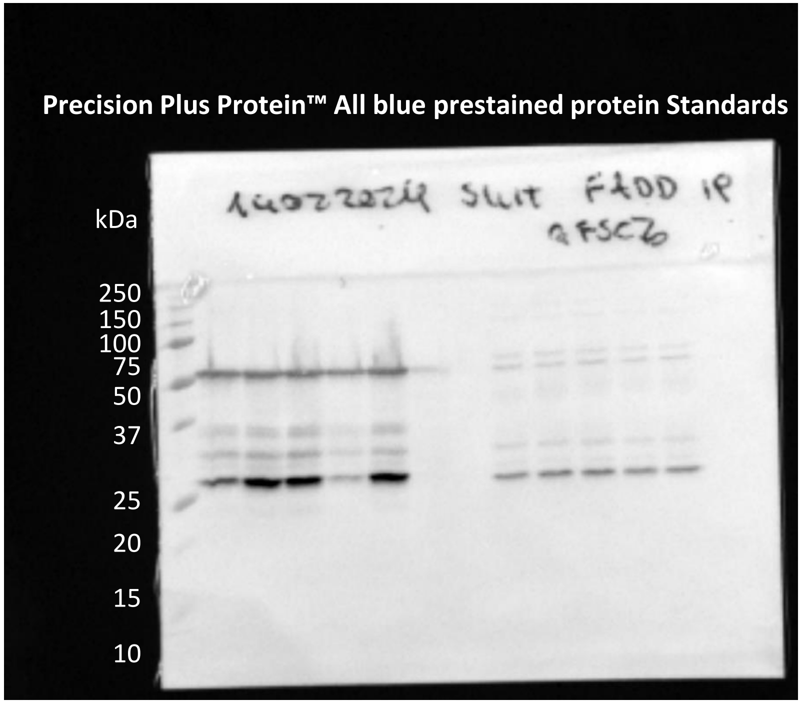
Precision Plus Protein™ All blue prestained protein Standards
kDa
250
150
100
75
50
37
25
20
15
10

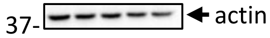
37-
actin

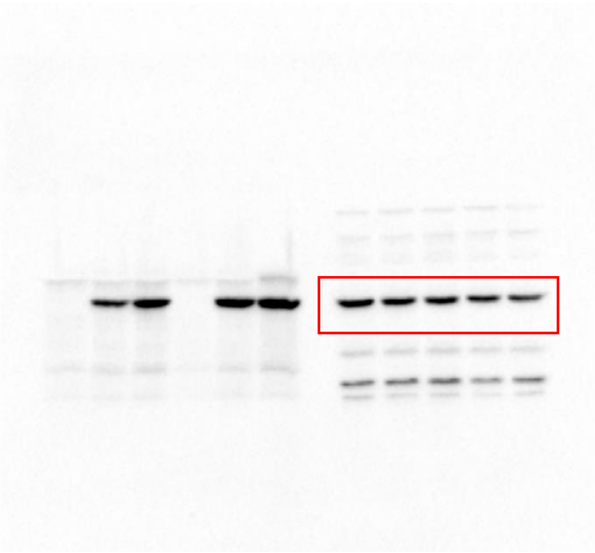

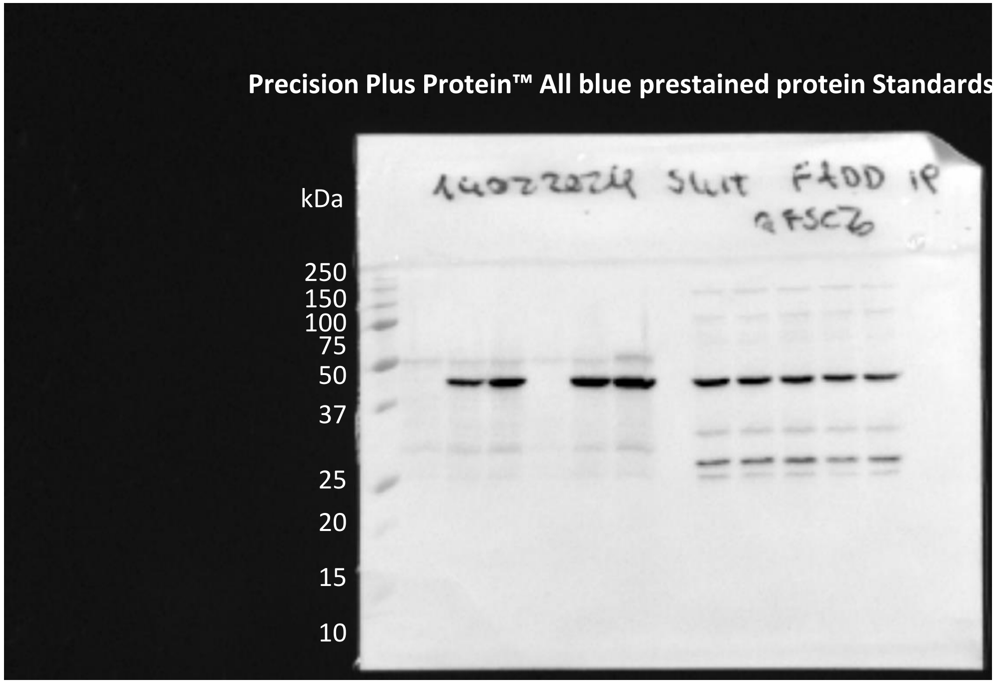
Precision Plus Protein™ All blue prestained protein Standards
kDa
250
150
100
75
50
37
25
20
15
10
FADD IP

a

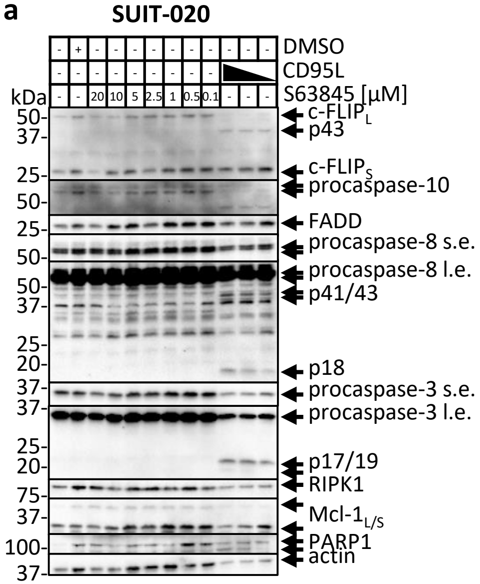
SUIT-020
DMSO
CD95L
S63845 [µM]
kDa
20
10
5
2.5
1
0.5
0.1
c-FLIP$_L$
p43
c-FLIP$_S$
procaspase-10
FADD
procaspase-8 s.e.
procaspase-8 l.e.
p41/43
p18
procaspase-3 s.e.
procaspase-3 l.e.
p17/19
RIPK1
Mcl-1$_{L/S}$
PARP1
actin
50
37
25
75
100
20

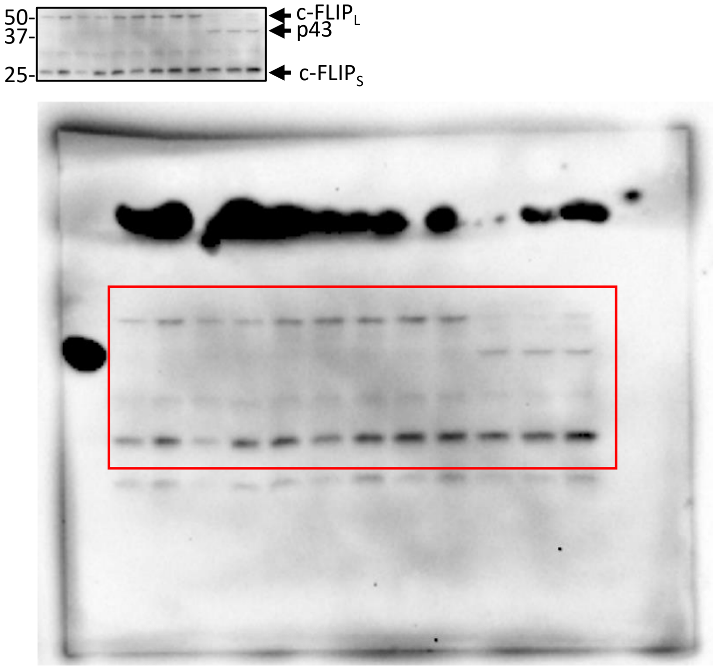
50-
37-
25-
c-FLIP$_L$
p43
c-FLIP$_S$

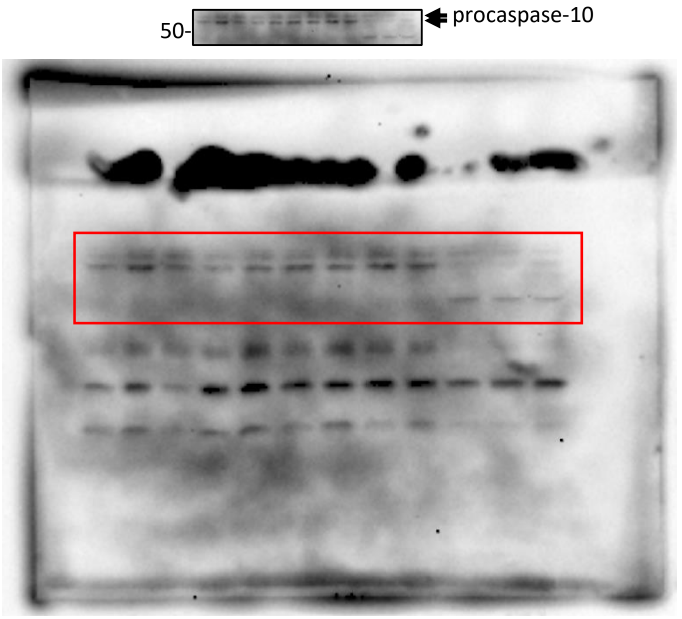
50-
procaspase-10

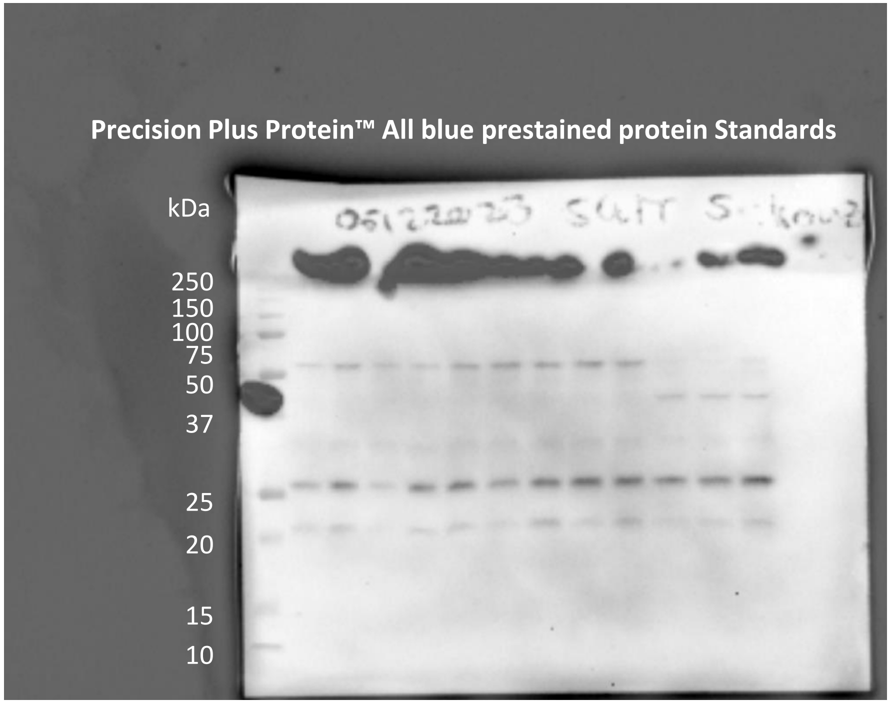
Precision Plus Protein™ All blue prestained protein Standards
kDa
250
150
100
75
50
37
25
20
15
10

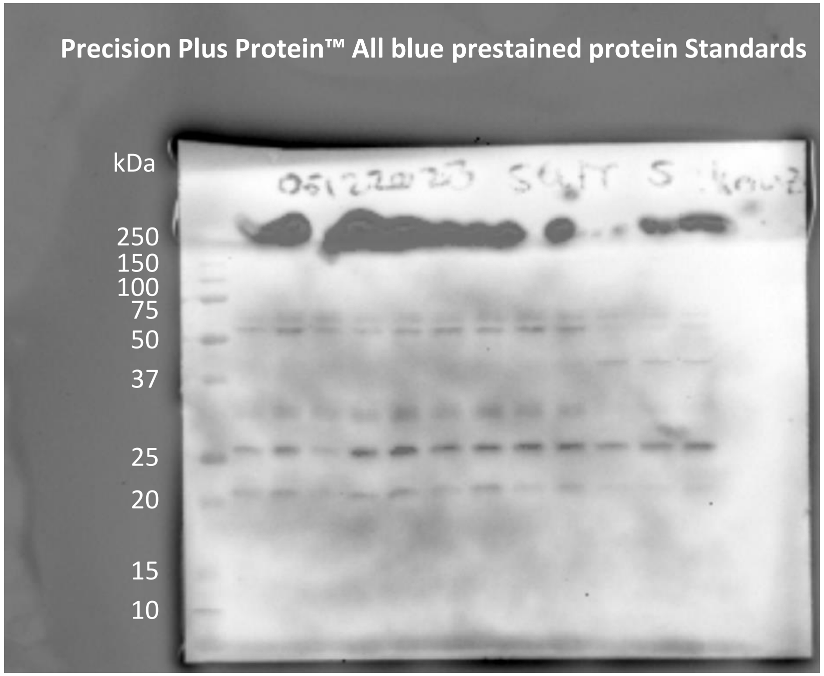
Precision Plus Protein™ All blue prestained protein Standards
kDa
250
150
100
75
50
37
25
20
15
10

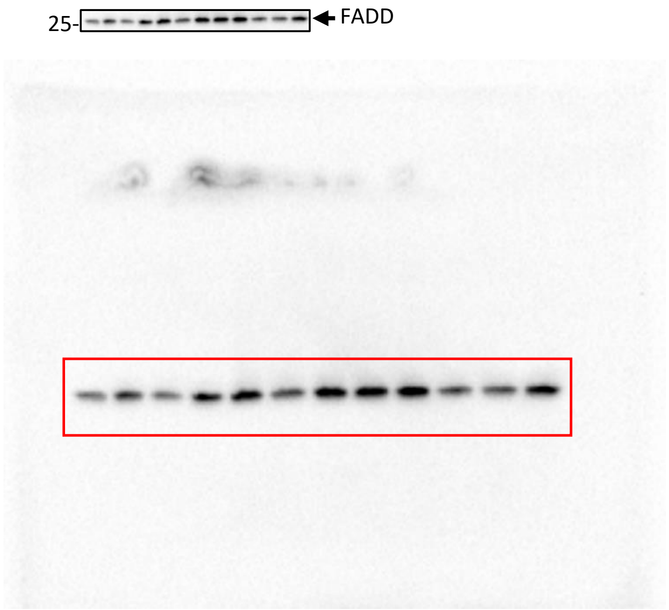
25-
FADD

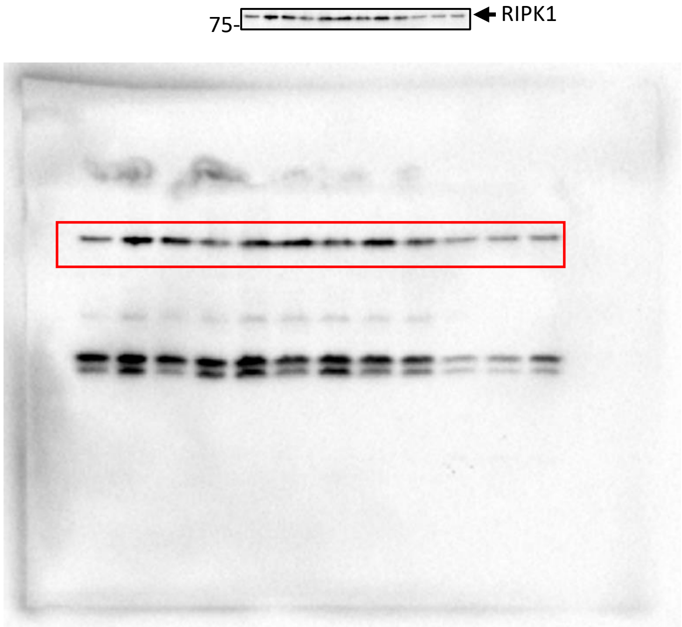
75-
RIPK1

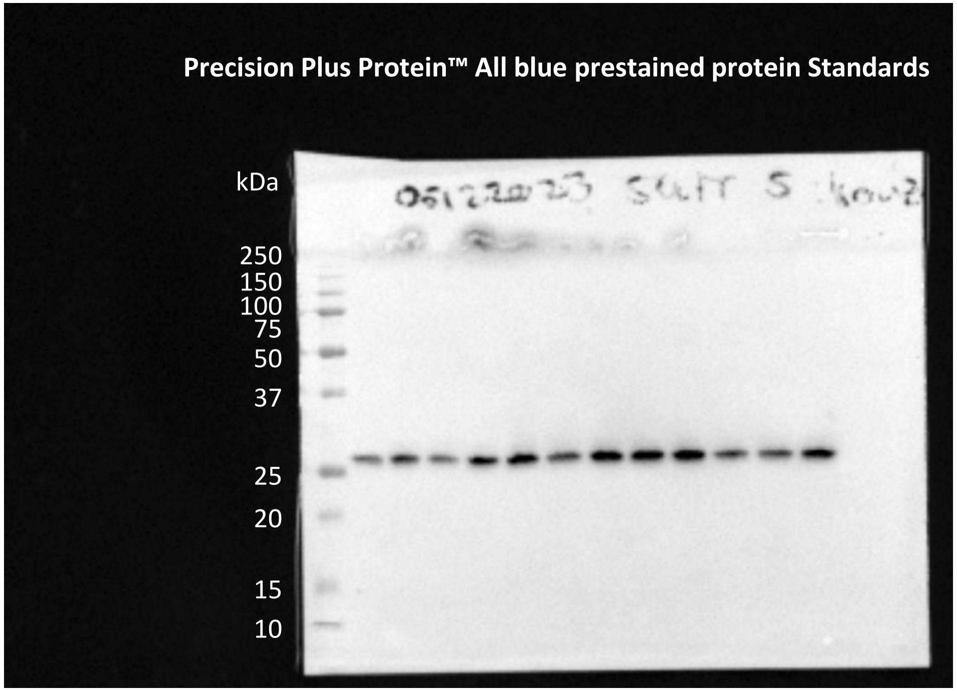
Precision Plus Protein™ All blue prestained protein Standards
kDa
250
150
100
75
50
37
25
20
15
10

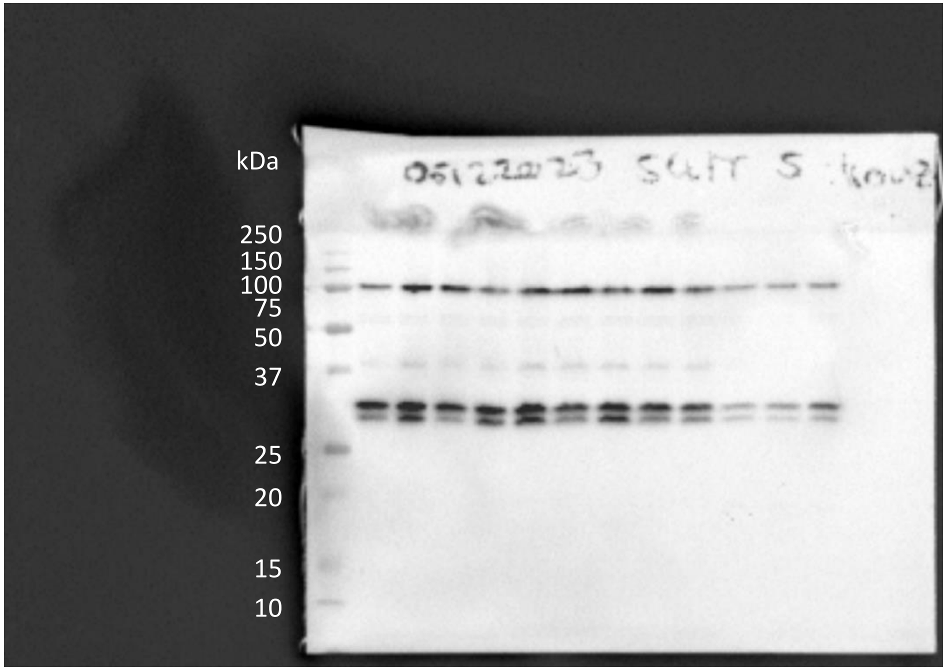
kDa
250
150
100
75
50
37
25
20
15
10

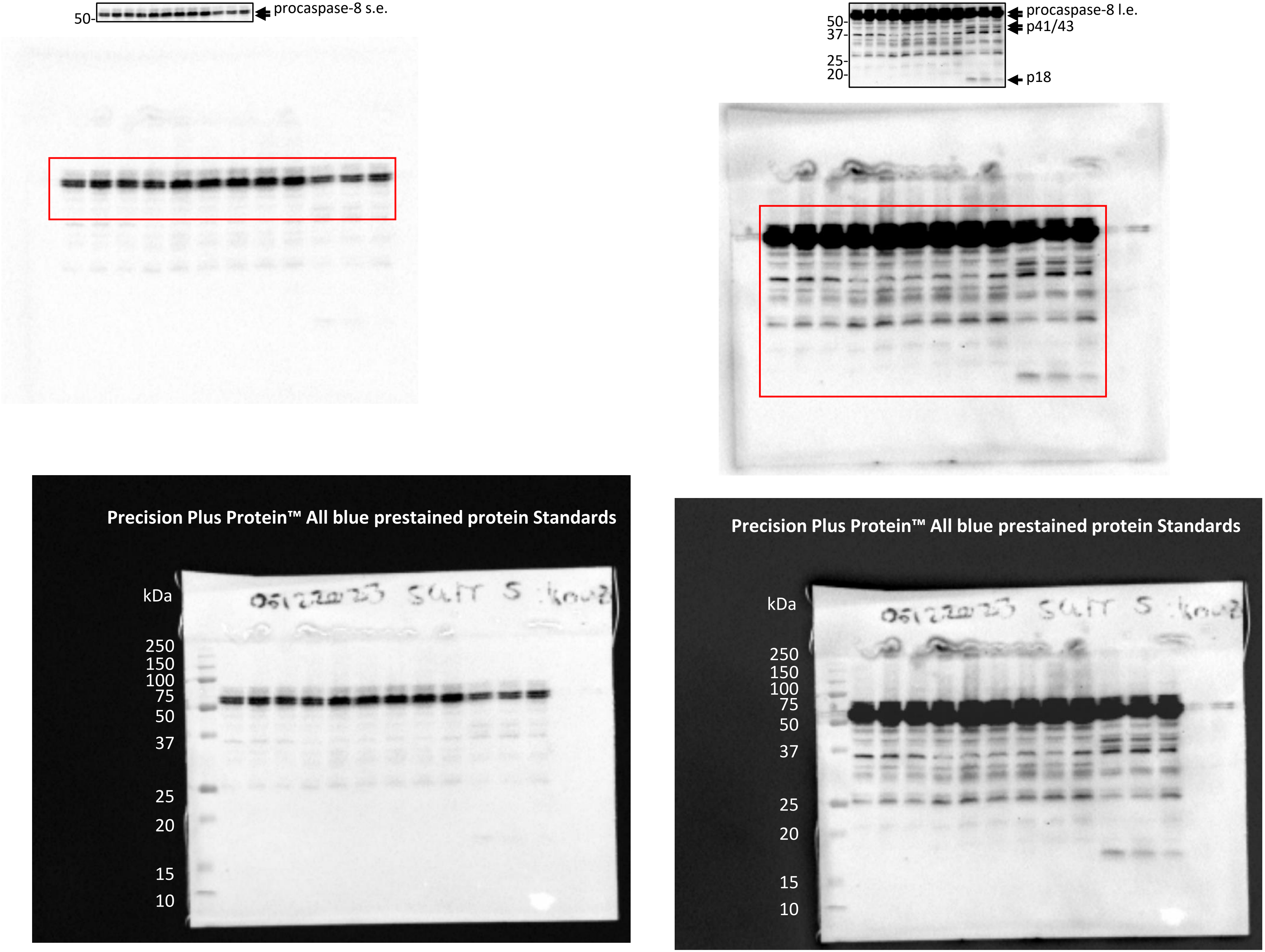
50-
procaspase-8 s.e.
50-
37-
25-
20-
procaspase-8 l.e.
p41/43
p18
Precision Plus Protein™ All blue prestained protein Standards
kDa
250
150
100
75
50
37
25
20
15
10
Precision Plus Protein™ All blue prestained protein Standards
kDa
250
150
100
75
50
37
25
20
15
10

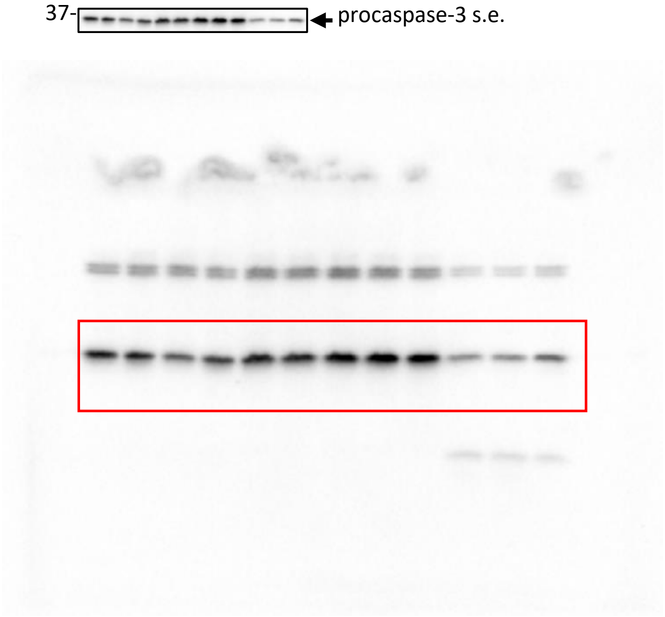
37-
procaspase-3 s.e.

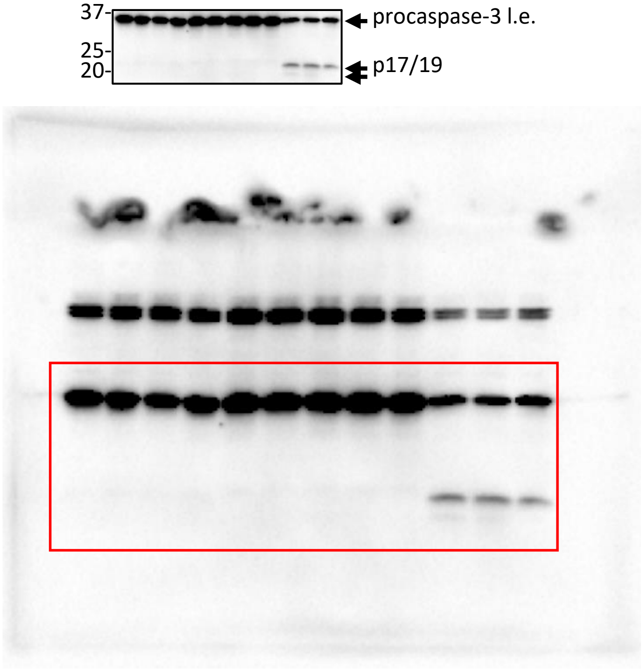
37-
25-
20-
procaspase-3 l.e.
p17/19

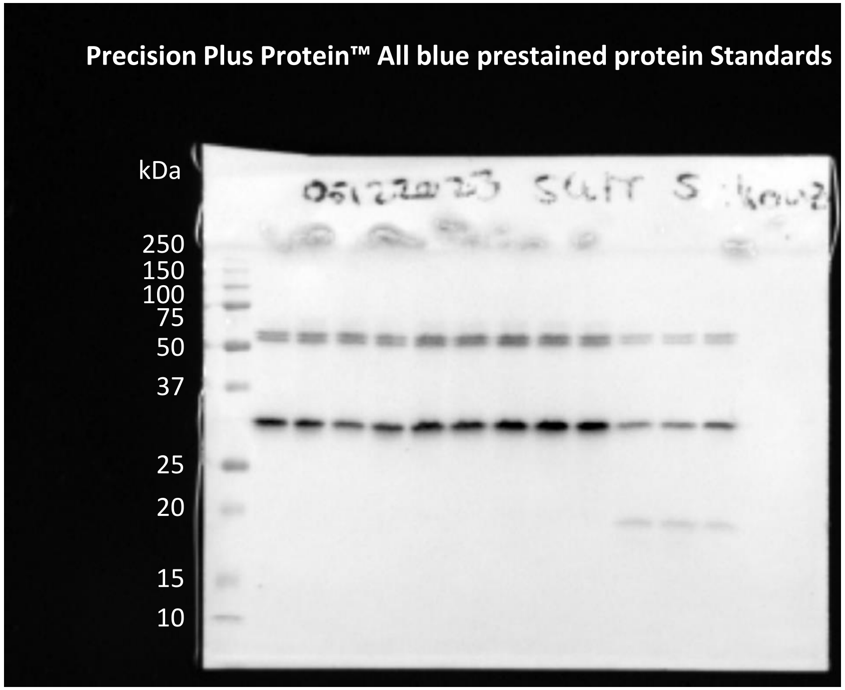
Precision Plus Protein™ All blue prestained protein Standards
kDa
250
150
100
75
50
37
25
20
15
10

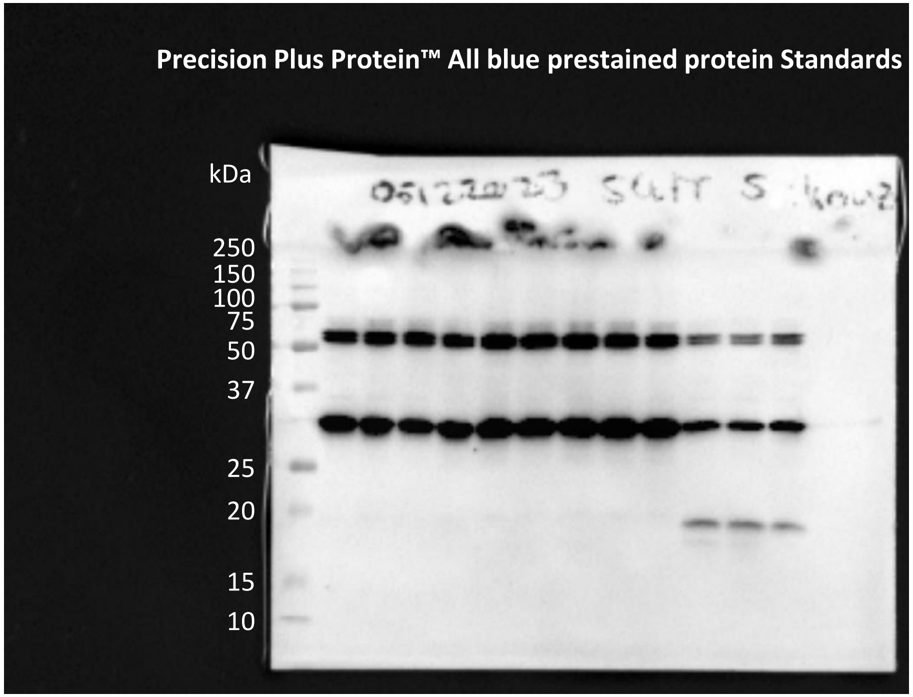
Precision Plus Protein™ All blue prestained protein Standards
kDa
250
150
100
75
50
37
25
20
15
10

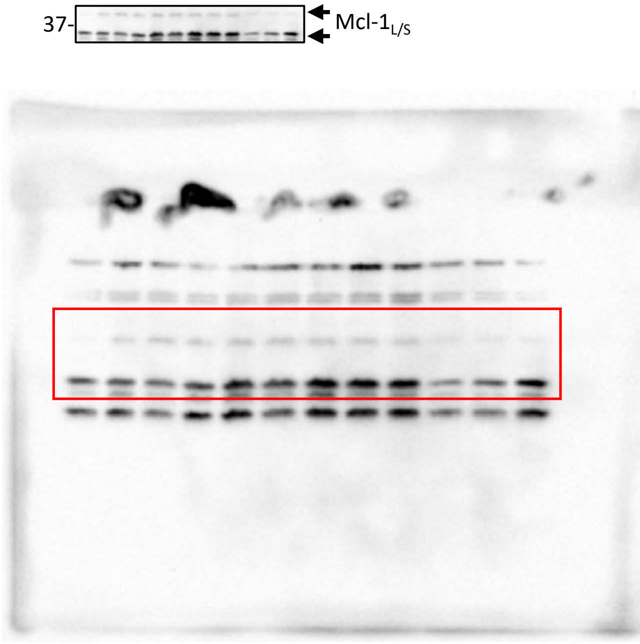
37-
Mcl-1 L/S

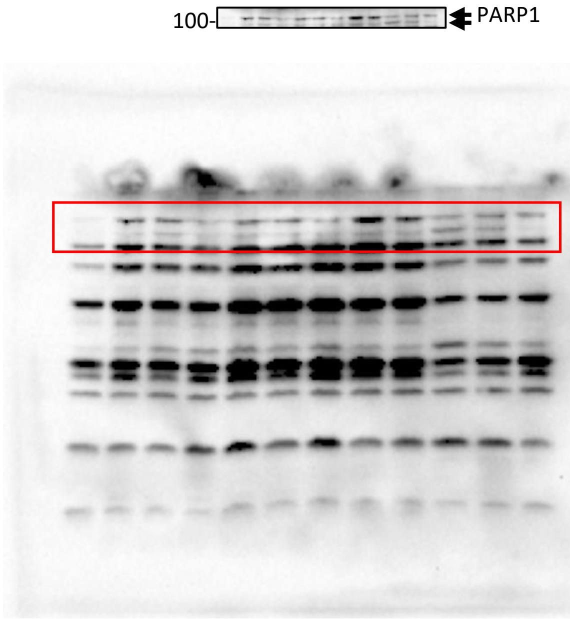
100-
PARP1

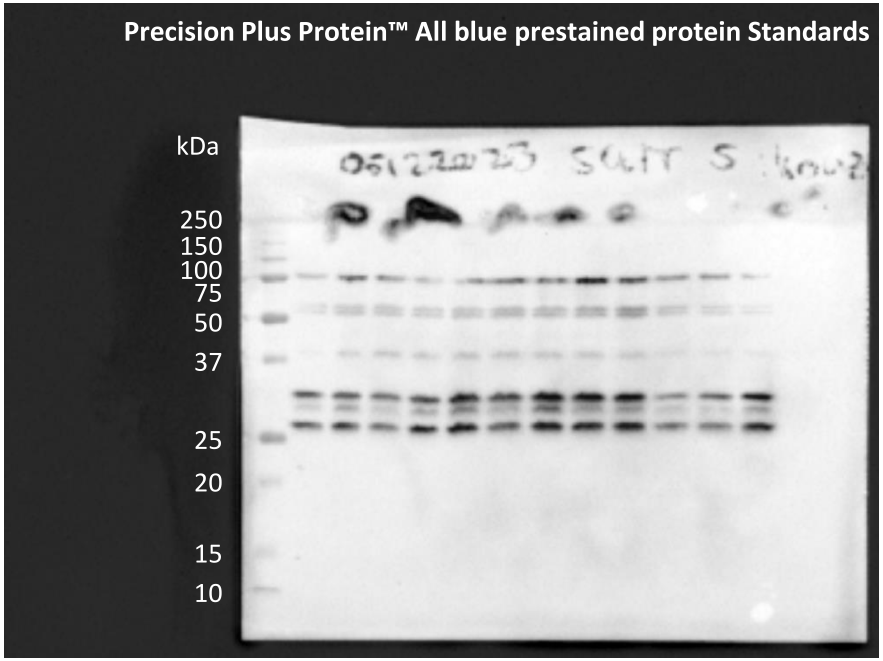
Precision Plus Protein™ All blue prestained protein Standards
kDa
250
150
100
75
50
37
25
20
15
10

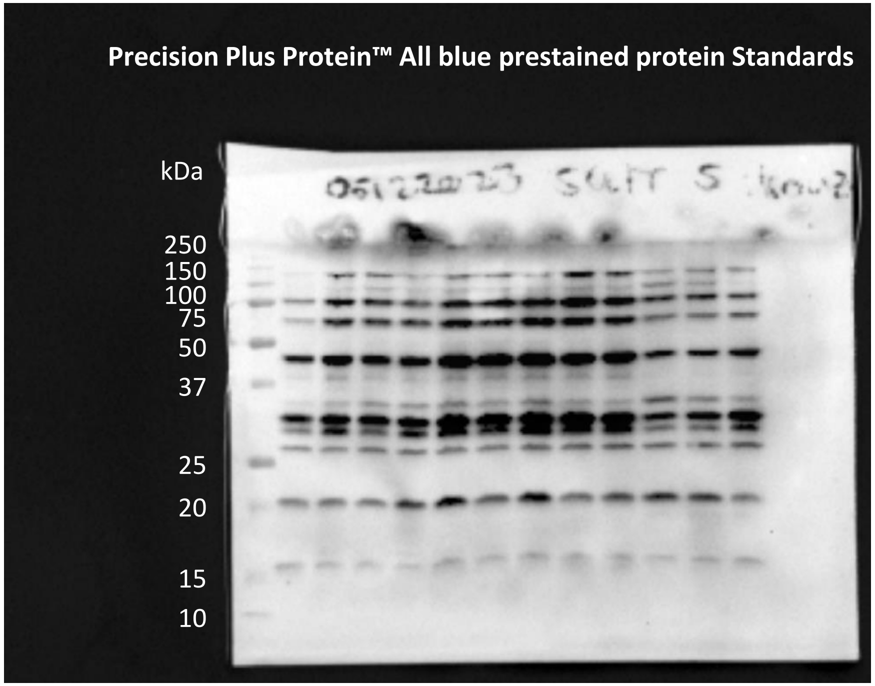
Precision Plus Protein™ All blue prestained protein Standards
kDa
250
150
100
75
50
37
25
20
15
10

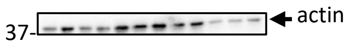
37-
actin

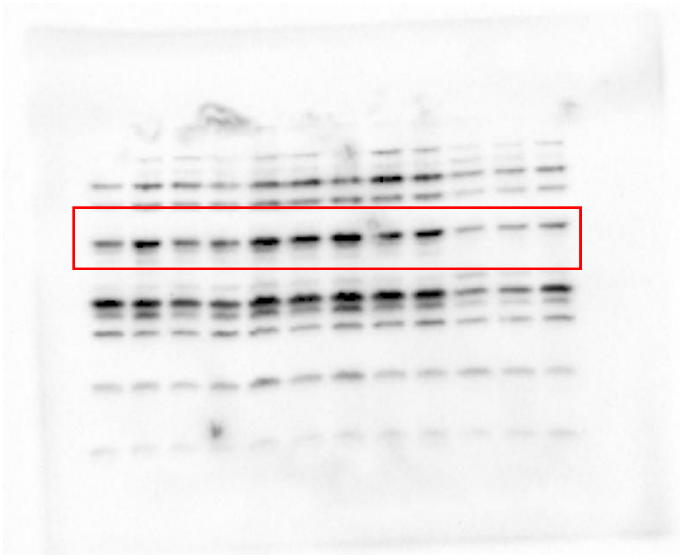

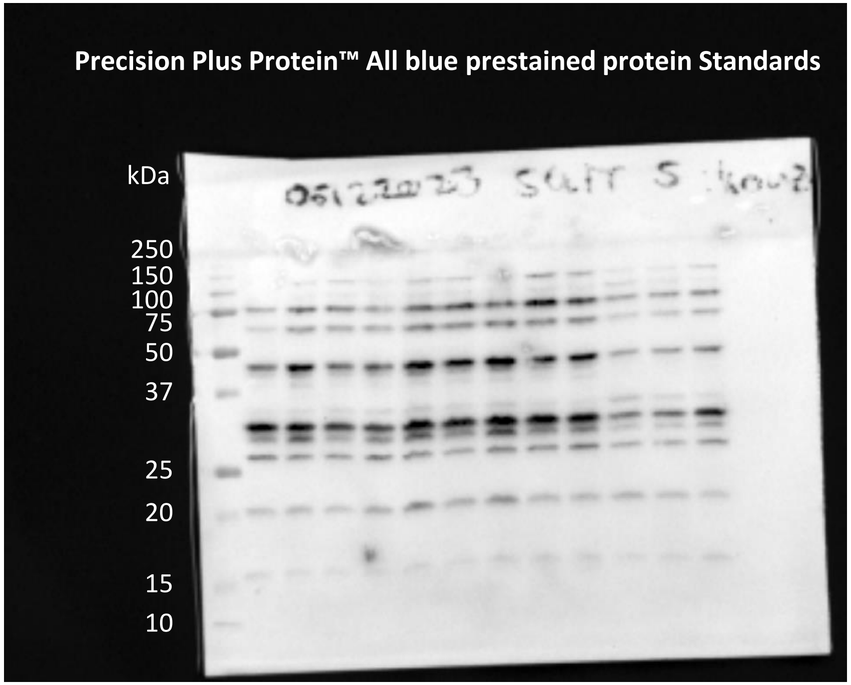
Precision Plus Protein™ All blue prestained protein Standards
kDa
250
150
100
75
50
37
25
20
15
10

b

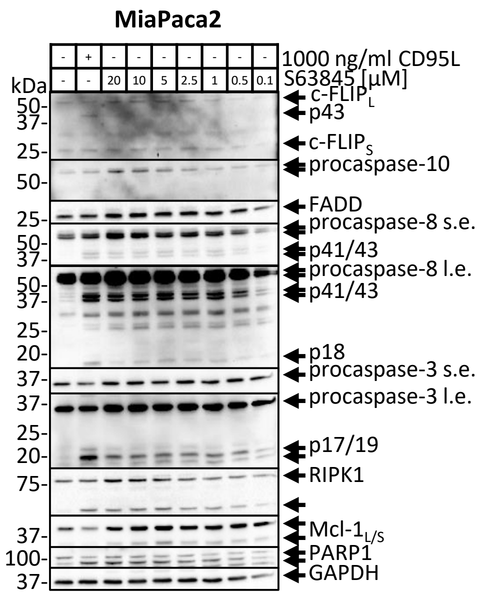
MiaPaca2
1000 ng/ml CD95L
S63845 [µM]
- + - - - - - - -
- - 20 10 5 2.5 1 0.5 0.1
kDa
50-
37-
25-
50-
25-
50-
37-
50-
37-
25-
20-
37-
37-
25-
20-
75-
37-
100-
37-
c-FLIP_L
p43
c-FLIP_S
procaspase-10
FADD
procaspase-8 s.e.
p41/43
procaspase-8 l.e.
p41/43
p18
procaspase-3 s.e.
procaspase-3 l.e.
p17/19
RIPK1
Mcl-1_L/S
PARP1
GAPDH

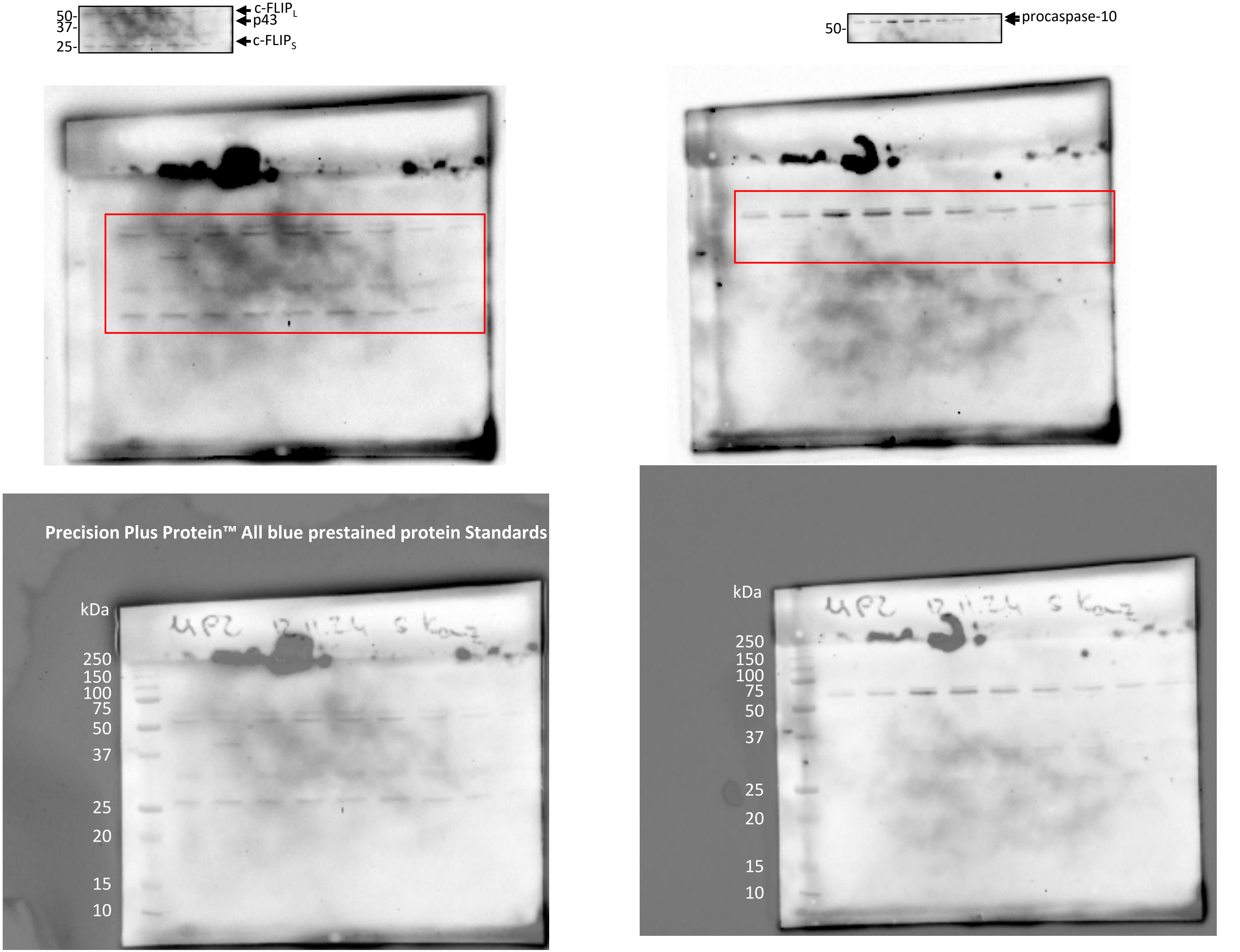

50-
37-
25-
c-FLIP$_L$
p43
c-FLIP$_S$
50-
procaspase-10
Precision Plus Protein™ All blue prestained protein Standards
kDa
250
150
100
75
50
37
25
20
15
10
kDa
250
150
100
75
50
37
25
20
15
10

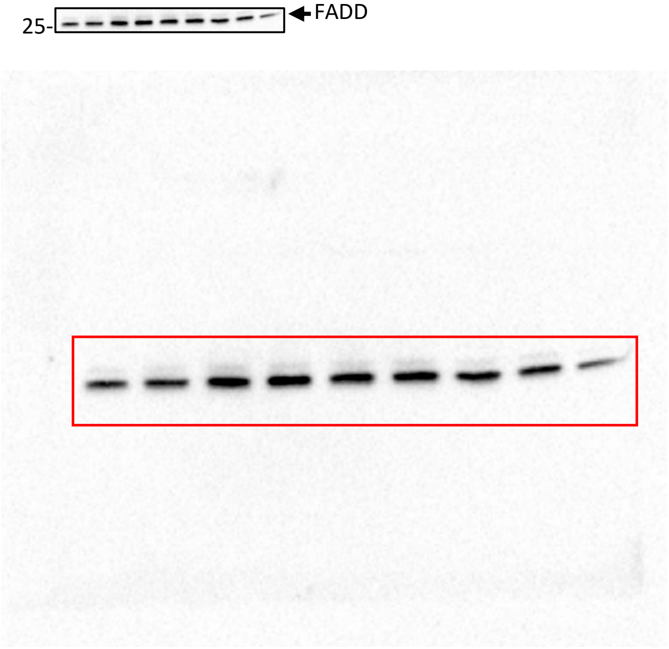
25-
FADD

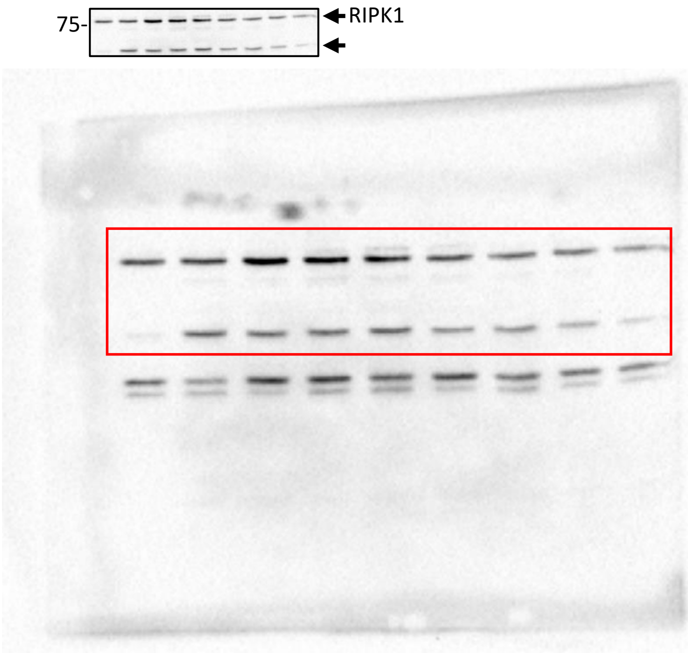
75-
RIPK1

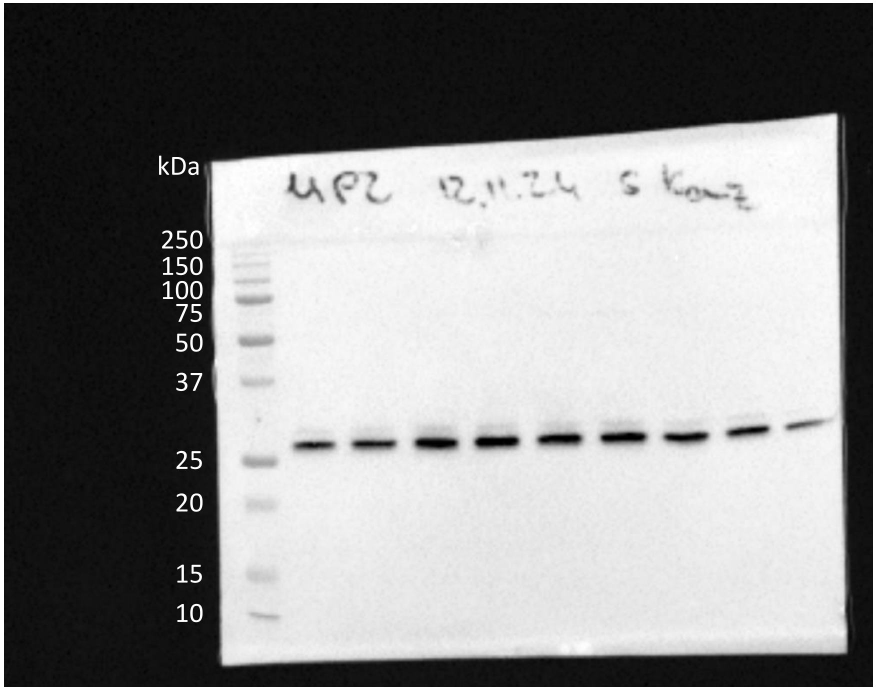
kDa
250
150
100
75
50
37
25
20
15
10

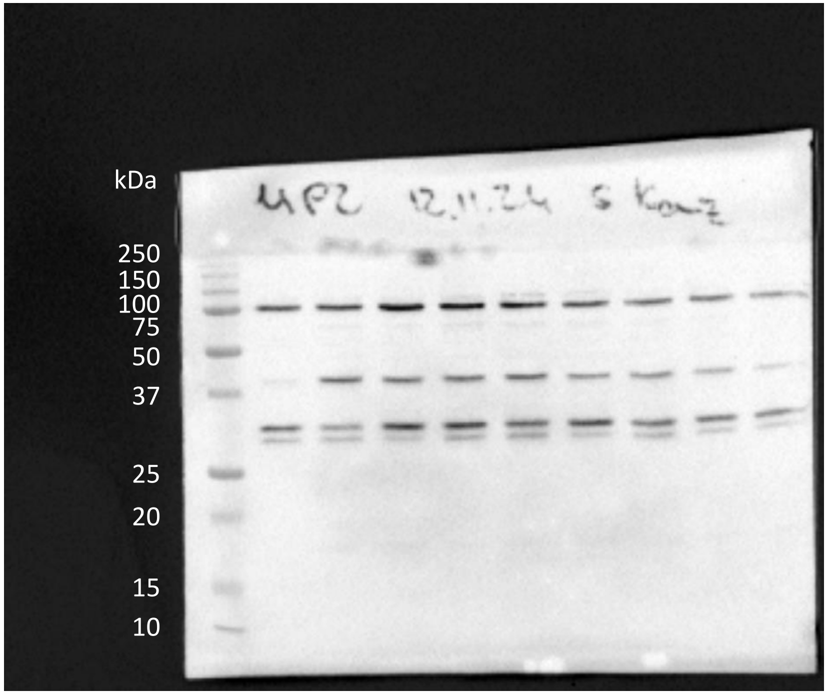
kDa
250
150
100
75
50
37
25
20
15
10

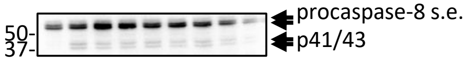
50-
37-
procaspase-8 s.e.
p41/43

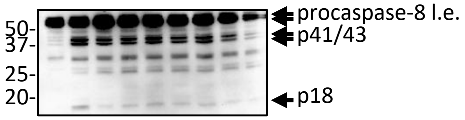
50-
37-
25-
20-
procaspase-8 l.e.
p41/43
p18

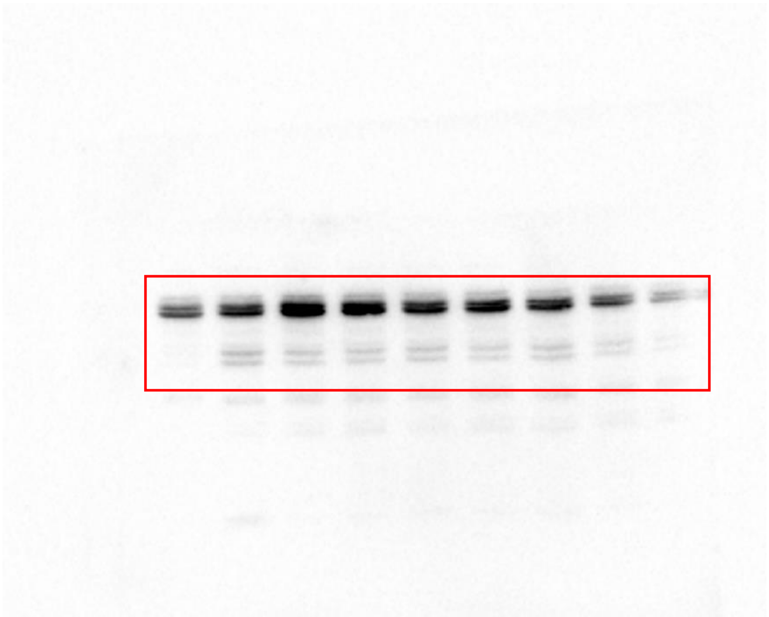

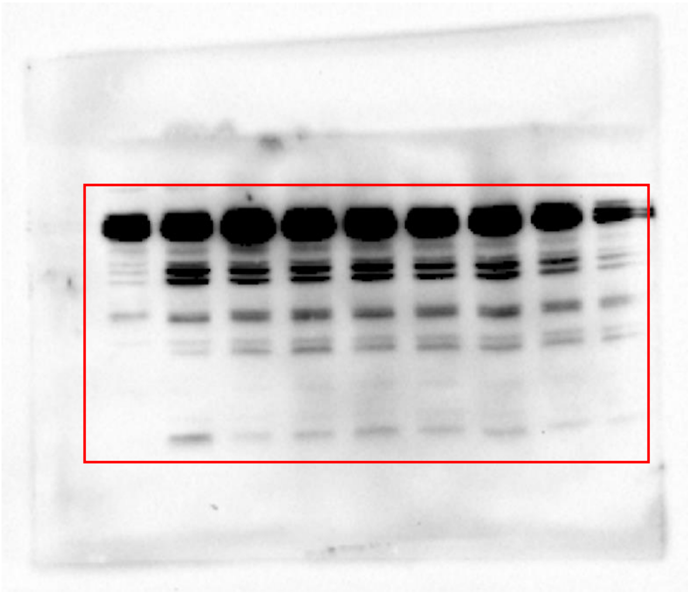

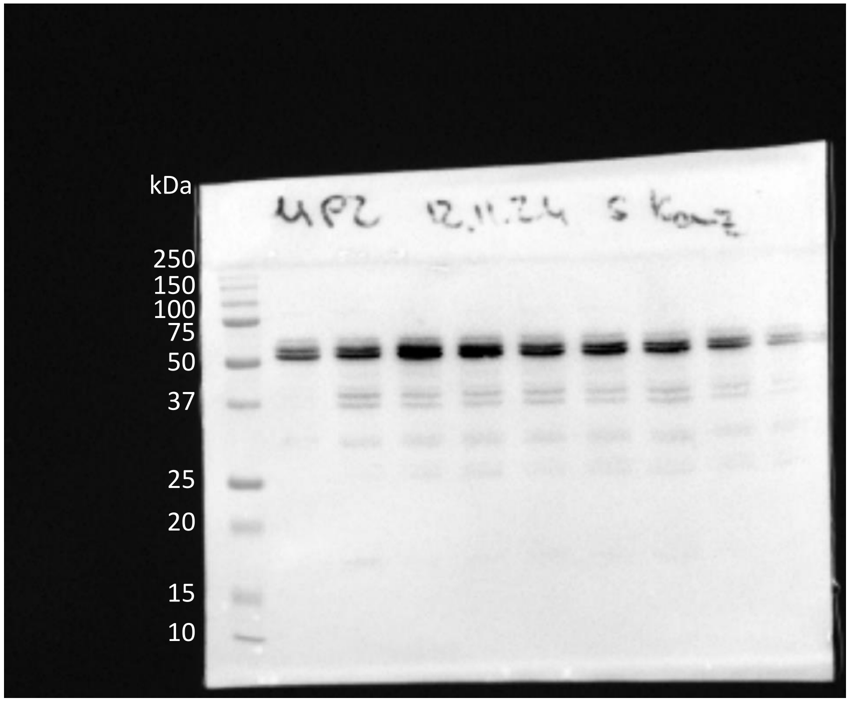
kDa
250
150
100
75
50
37
25
20
15
10

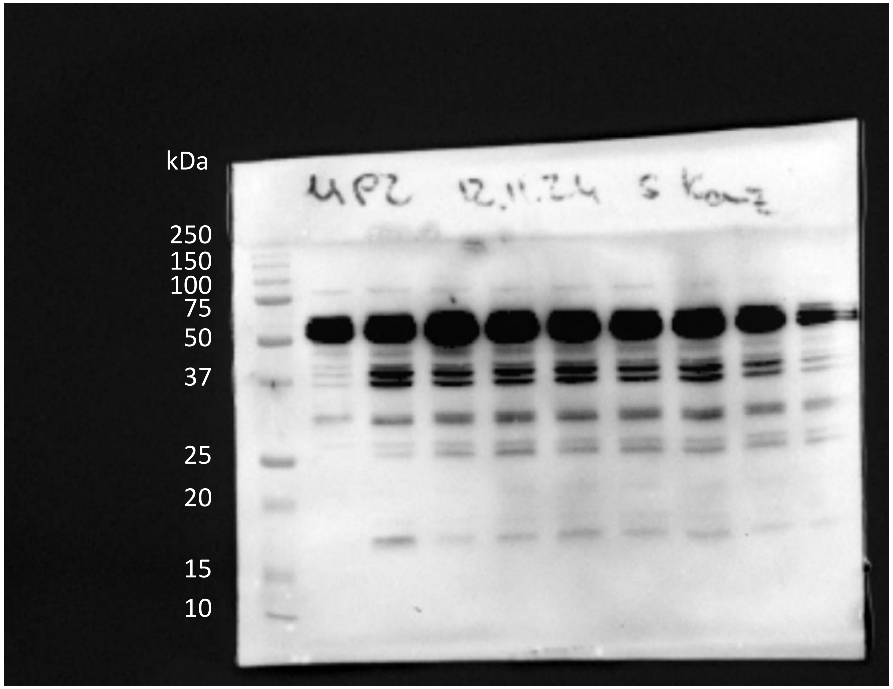
kDa
250
150
100
75
50
37
25
20
15
10

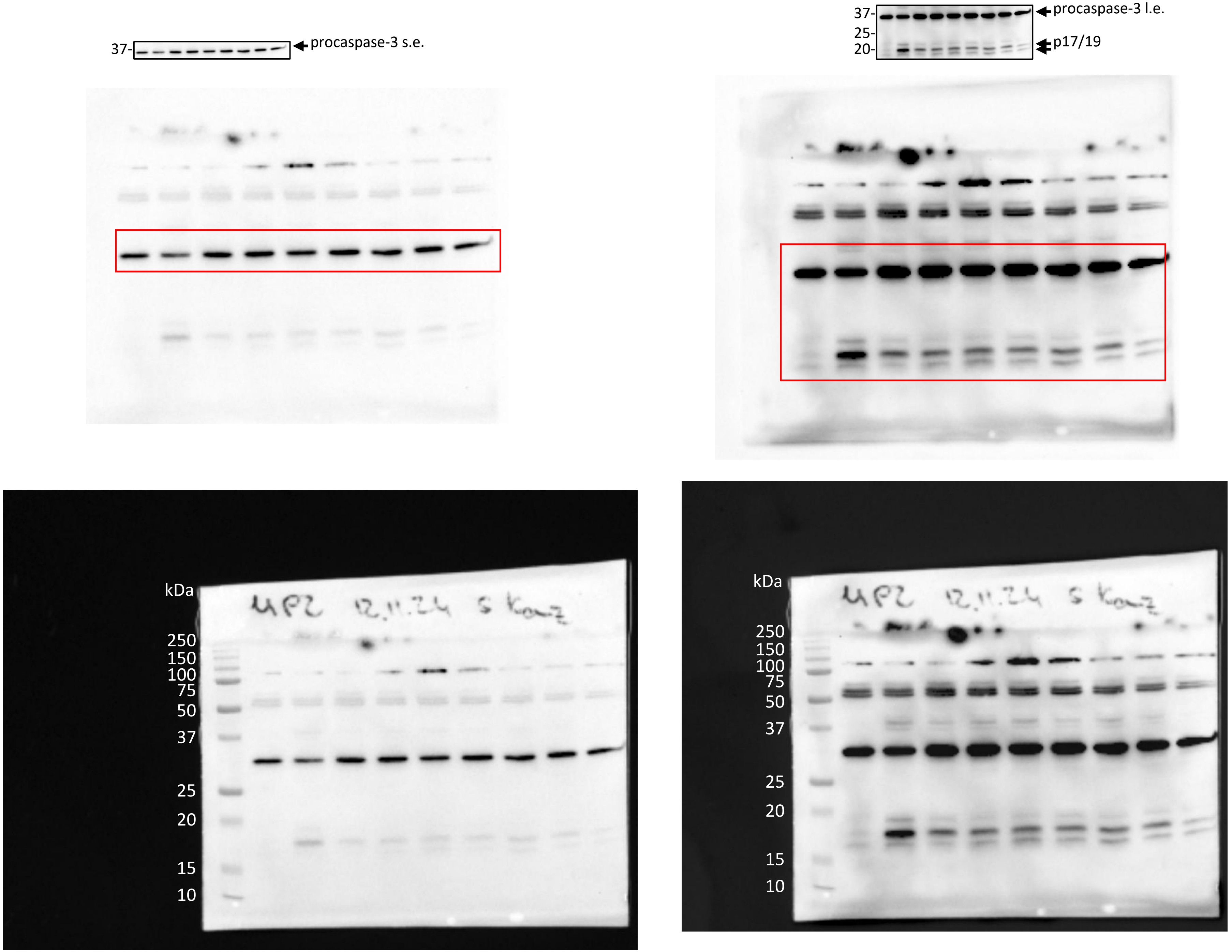
37-
procaspase-3 s.e.
37-
procaspase-3 l.e.
25-
20-
p17/19
kDa
250
150
100
75
50
37
25
20
15
10
kDa
250
150
100
75
50
37
25
20
15
10

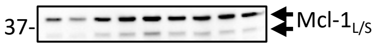
37-
Mcl-1L/S

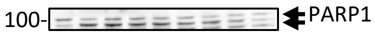
100-
PARP1

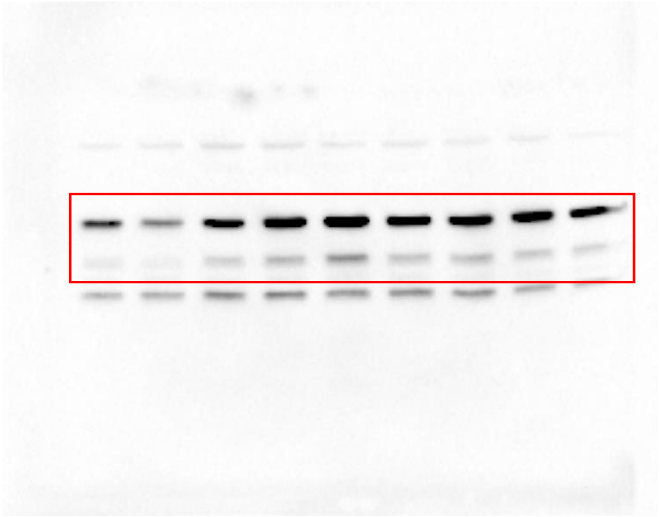

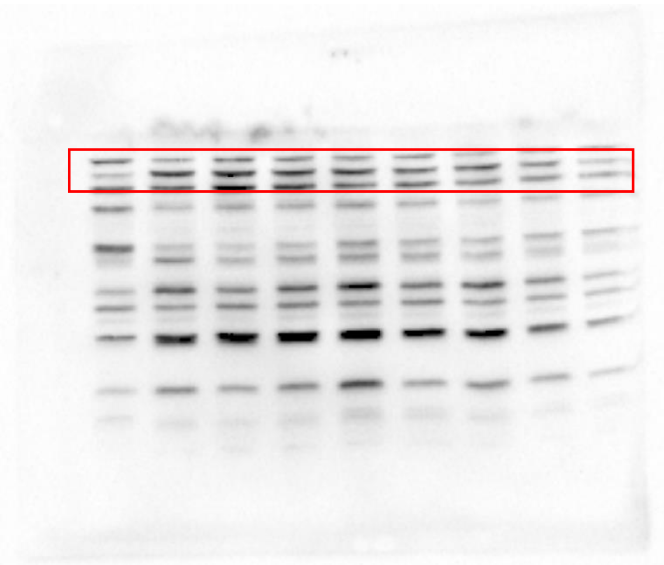

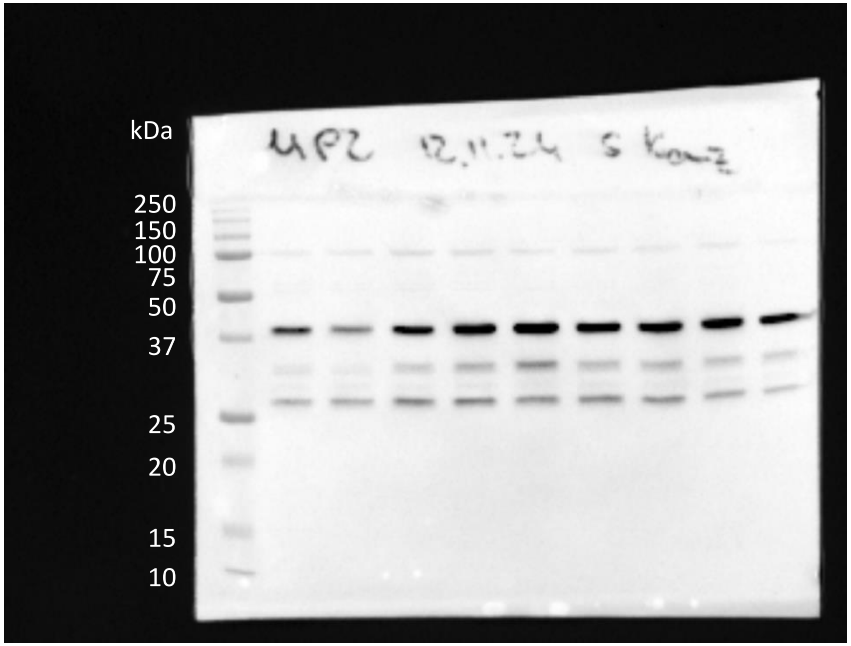
kDa
250
150
100
75
50
37
25
20
15
10

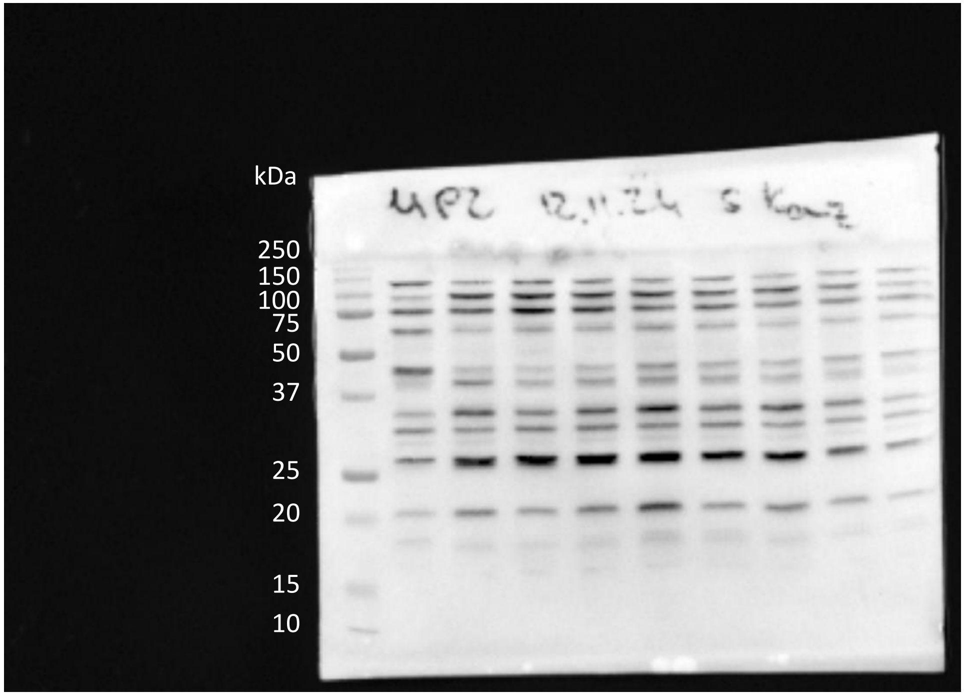
kDa
250
150
100
75
50
37
25
20
15
10

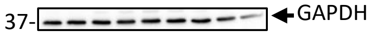
37-
GAPDH

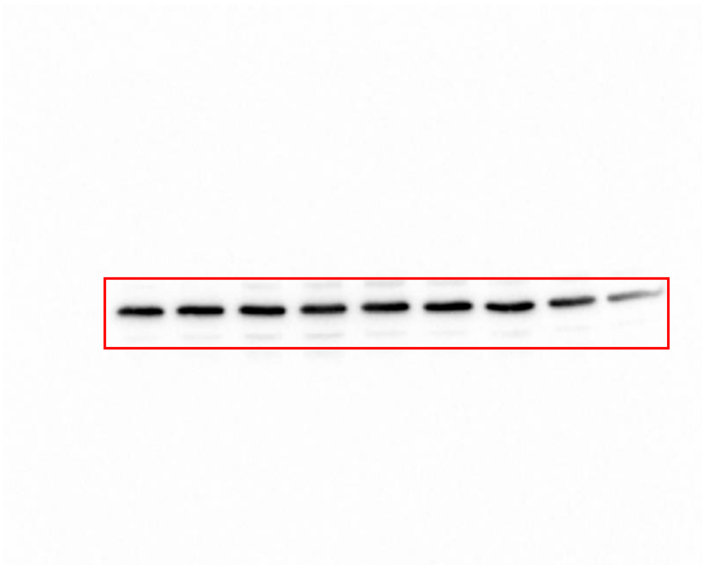

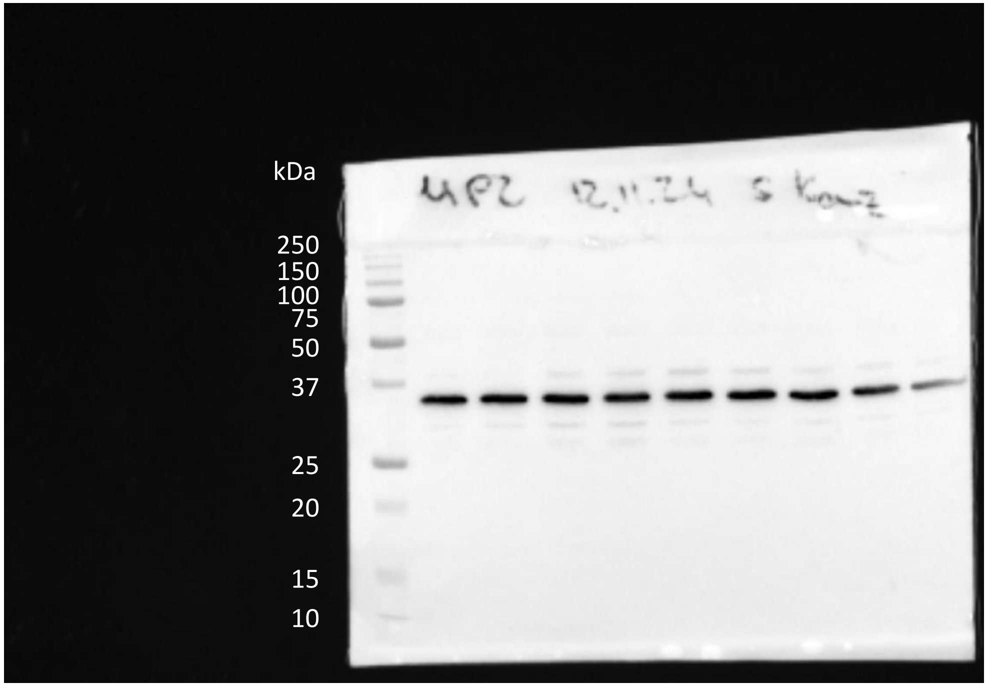
kDa
250
150
100
75
50
37
25
20
15
10

c

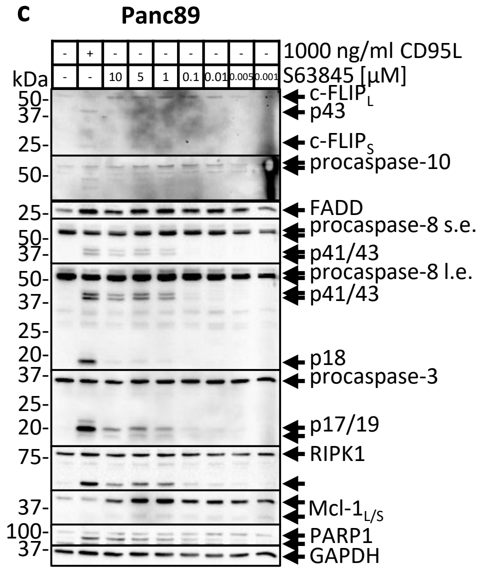
Panc89
1000 ng/ml CD95L
S63845 [µM]
kDa
- + - - - - - - -
- - 10 5 1 0.1 0.01 0.005 0.001
50-
37-
25-
50-
25-
50-
37-
50-
37-
25-
20-
37-
25-
20-
75-
37-
100-
37-
c-FLIPL
p43
c-FLIPS
procaspase-10
FADD
procaspase-8 s.e.
p41/43
procaspase-8 l.e.
p41/43
p18
procaspase-3
p17/19
RIPK1
Mcl-1L/S
PARP1
GAPDH

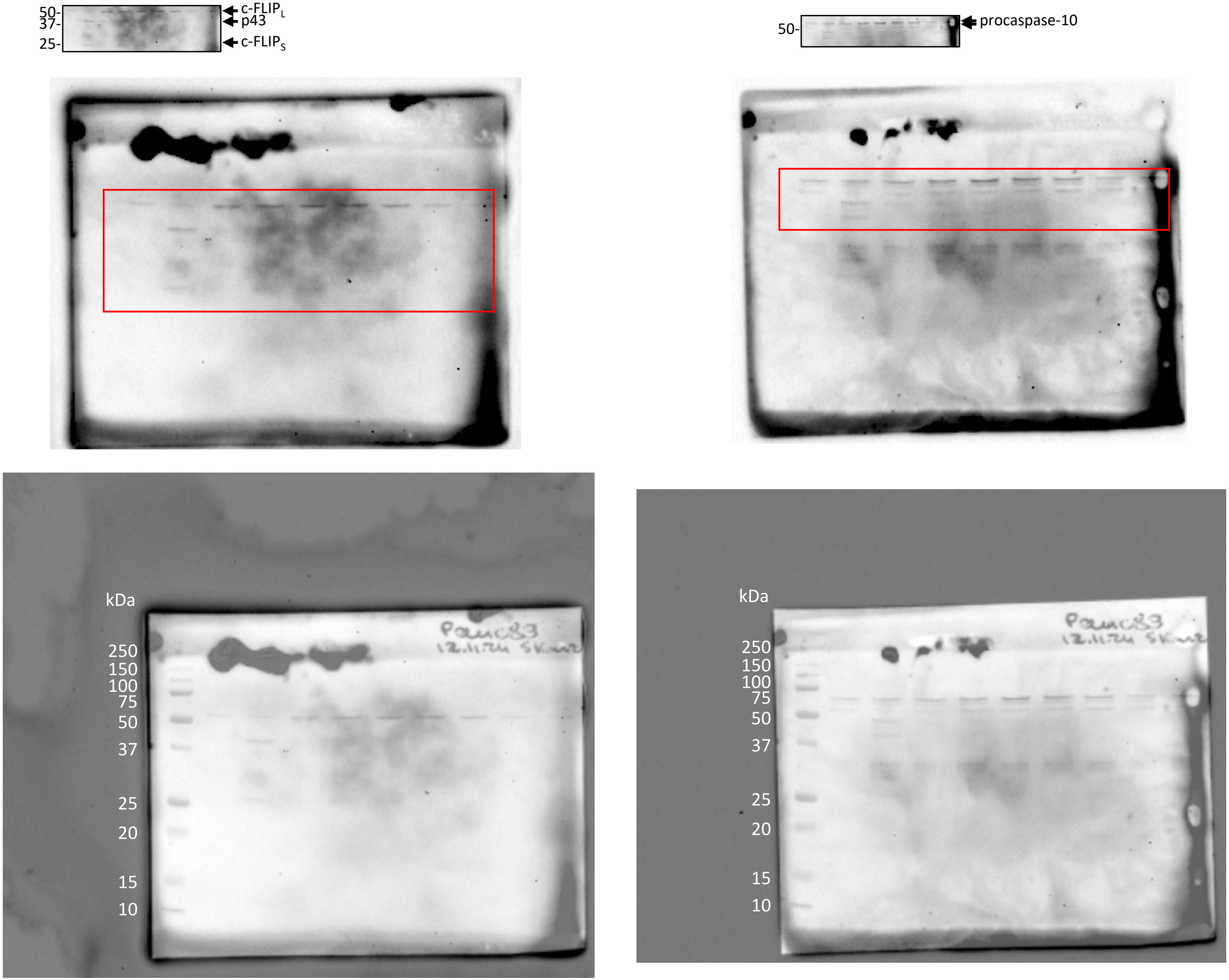
50-
37-
25-
c-FLIP_L
p43
c-FLIP_S
50-
procaspase-10
kDa
250
150
100
75
50
37
25
20
15
10
kDa
250
150
100
75
50
37
25
20
15
10

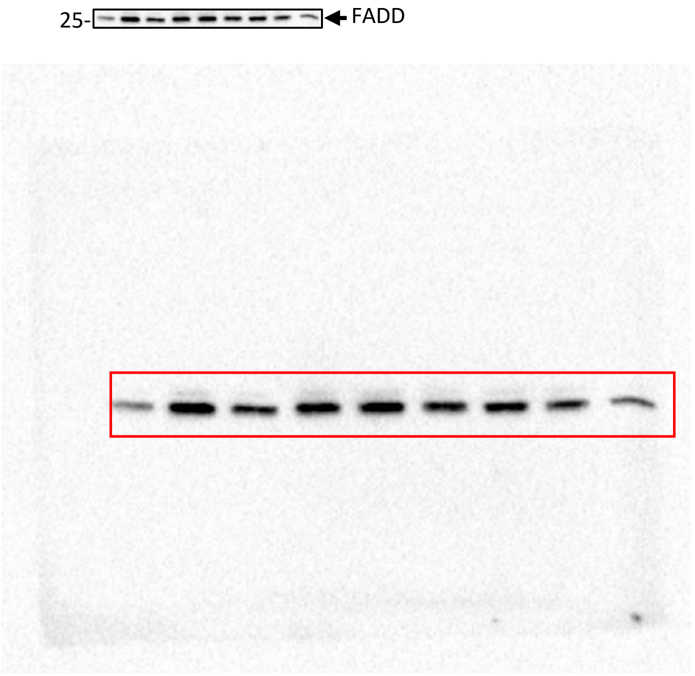
25-
FADD

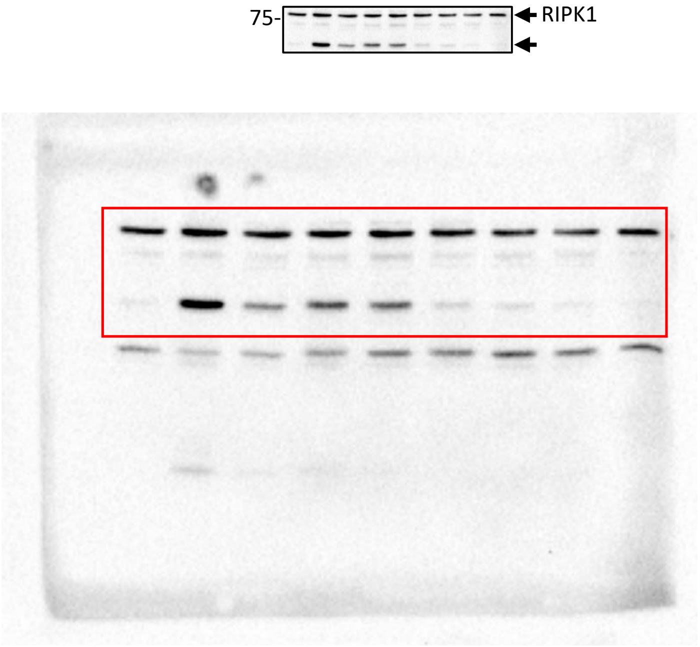
75-
RIPK1

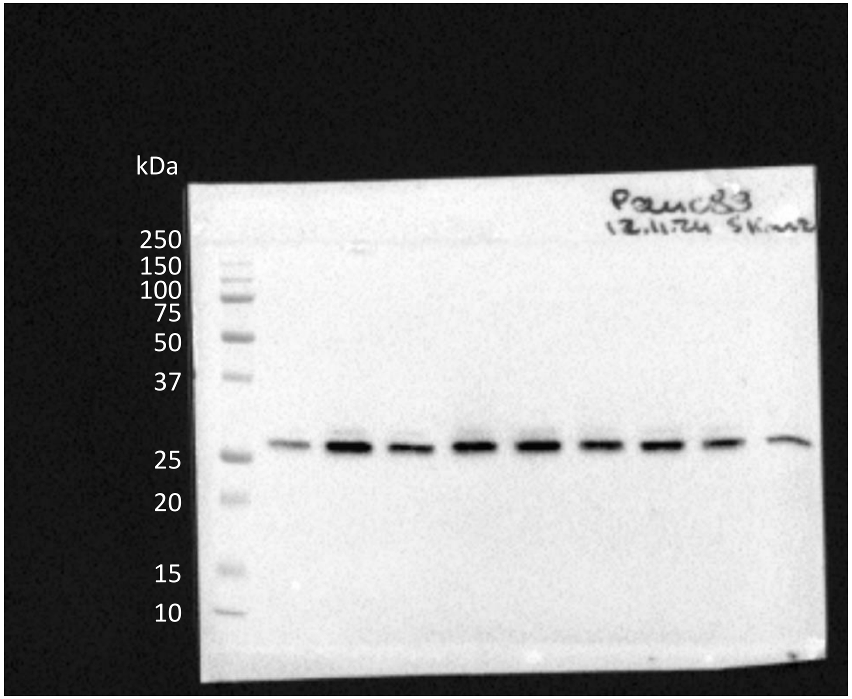
kDa
250
150
100
75
50
37
25
20
15
10

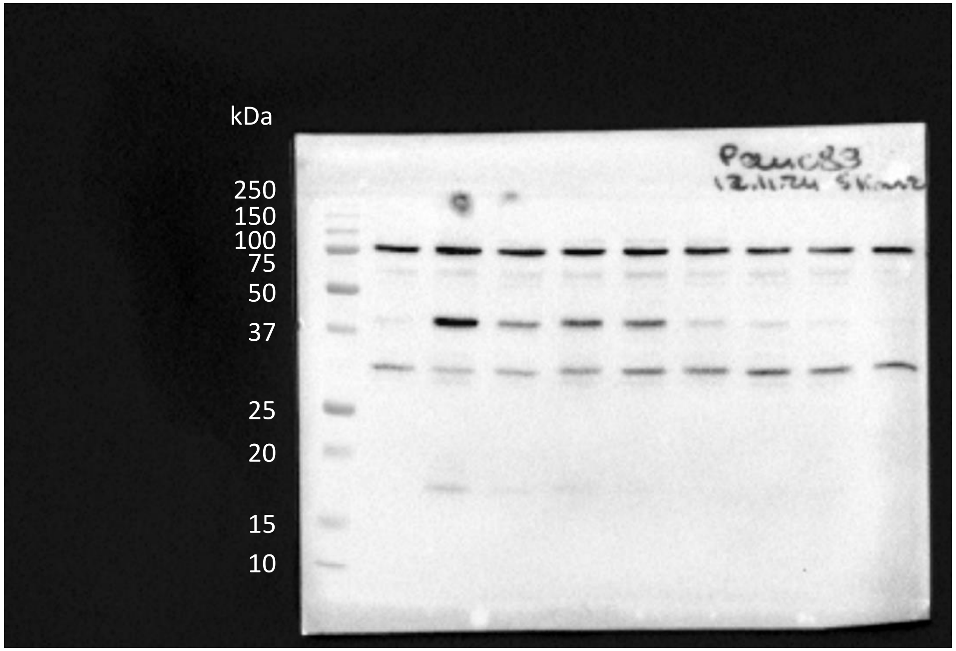
kDa
250
150
100
75
50
37
25
20
15
10

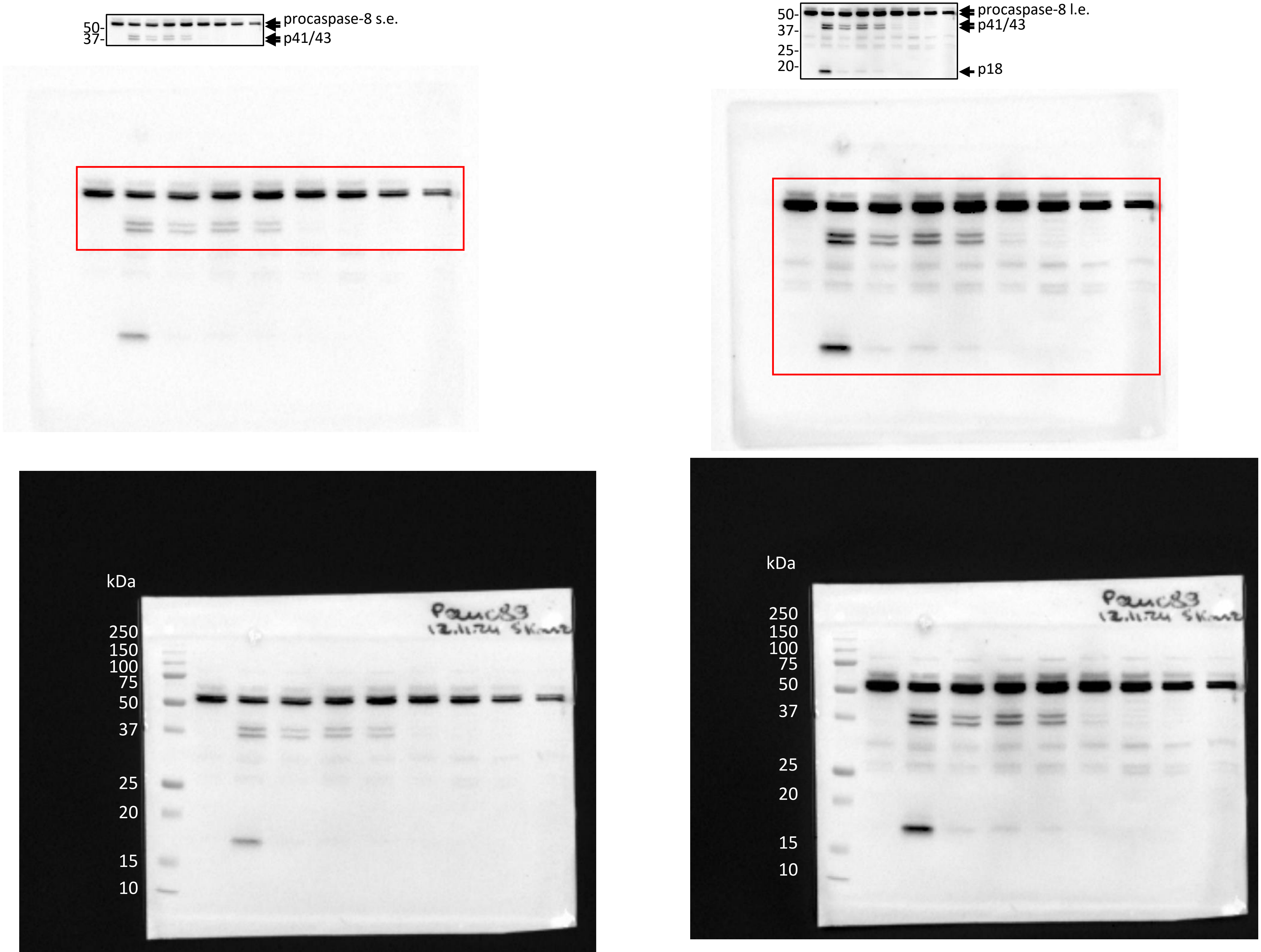
50-
37-
procaspase-8 s.e.
p41/43
50-
37-
25-
20-
procaspase-8 l.e.
p41/43
p18
kDa
250
150
100
75
50
37
25
20
15
10
kDa
250
150
100
75
50
37
25
20
15
10

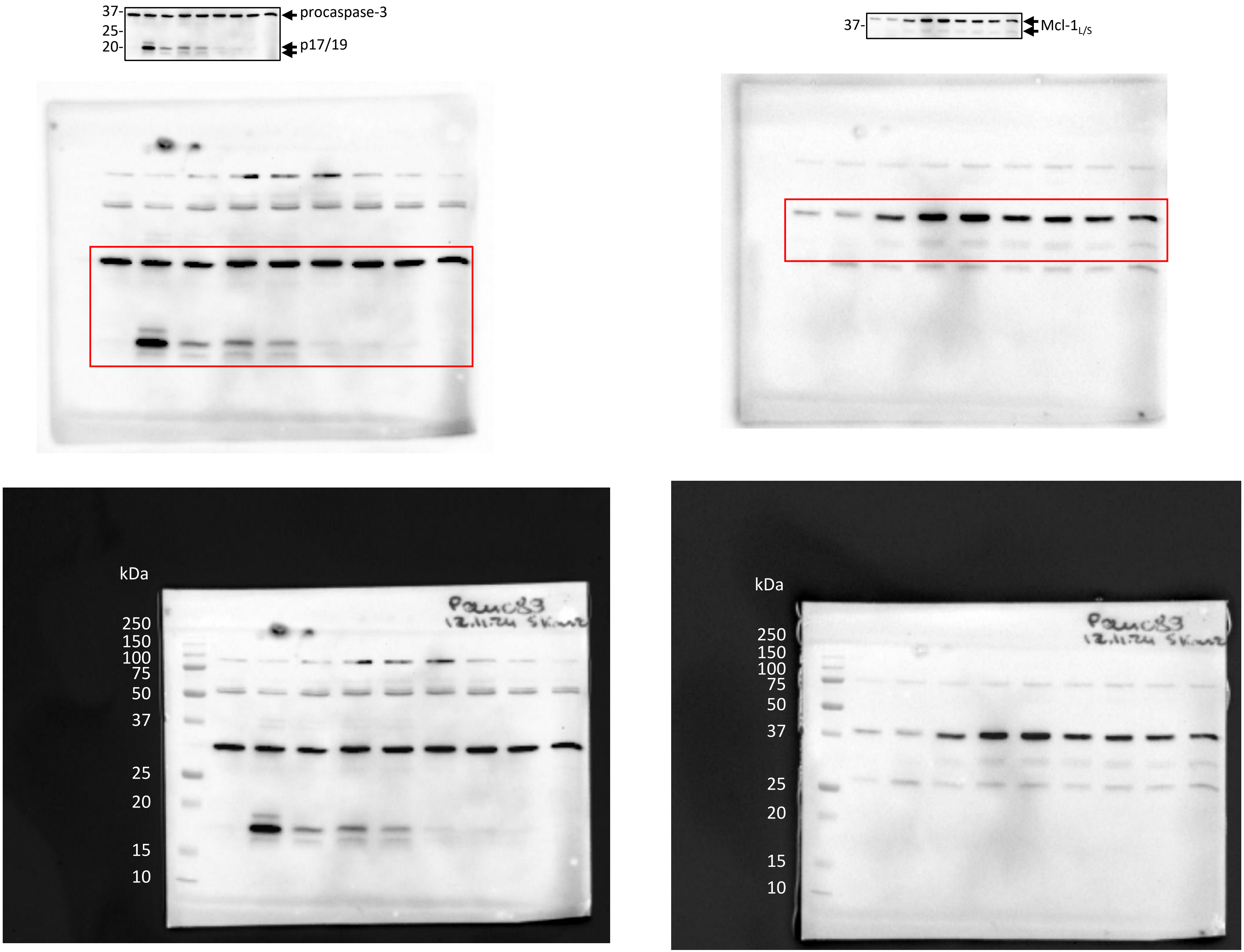
37-
25-
20-
procaspase-3
p17/19
37-
Mcl-1L/S
kDa
250
150
100
75
50
37
25
20
15
10
kDa
250
150
100
75
50
37
25
20
15
10

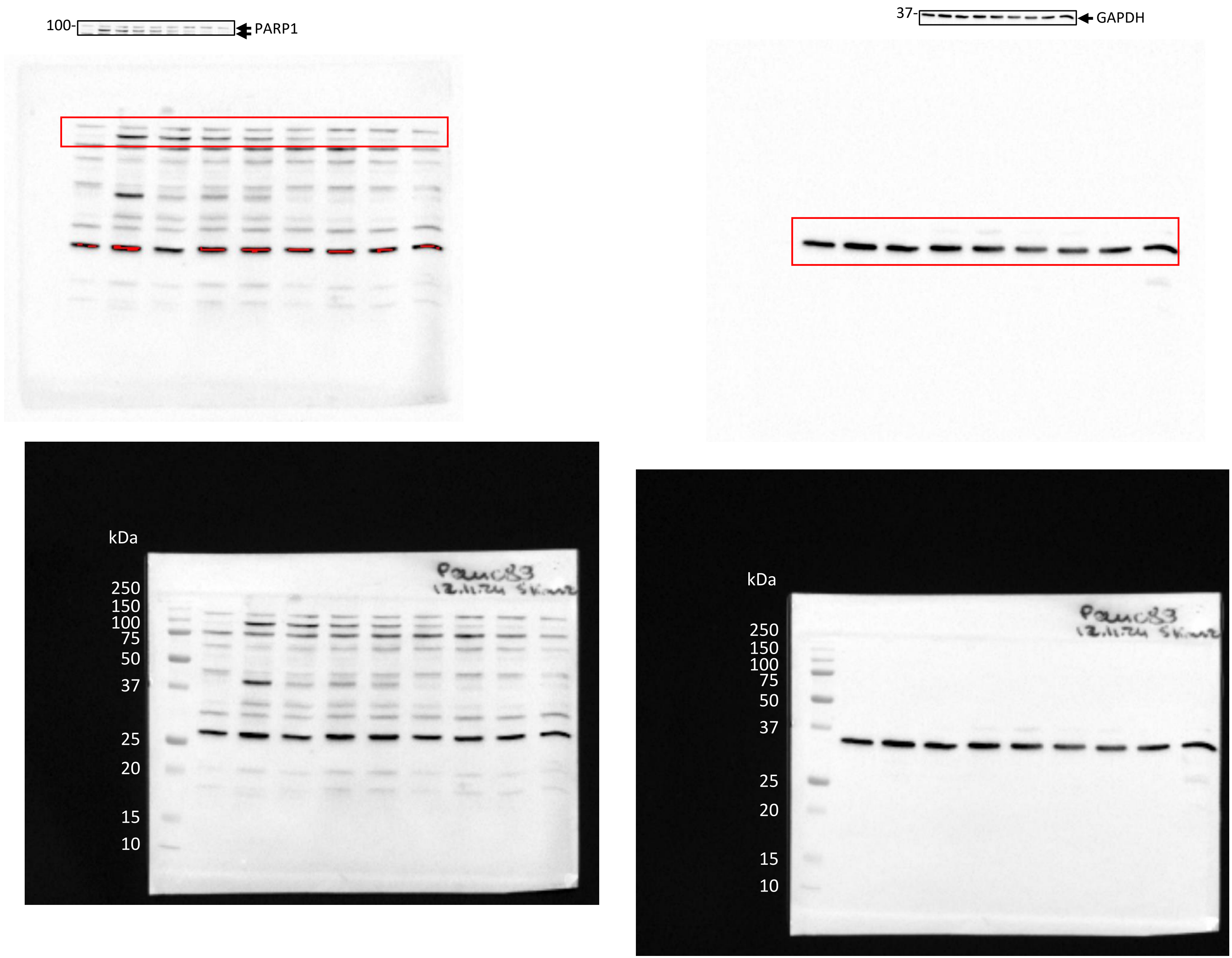
100-
PARP1
37-
GAPDH
kDa
250
150
100
75
50
37
25
20
15
10
kDa
250
150
100
75
50
37
25
20
15
10
