# Supplementary material for: Pharmacological targeting of caspase-8/c-FLIPL heterodimer enhances complex II assembly and elimination of pancreatic cancer cells
Source: Commun Biol. 2025 Jan 3;8:4. doi: 10.1038/s42003-024-07409-6 (PMC11698904; doi:10.1038/s42003-024-07409-6)
Supplement: Supplementary file 2 — Description of Additional Supplementary Files [file 42003_2024_7409_MOESM2_ESM.pdf]

Description of Additional Supplementary Files

File name: Supplementary Information

Description: Supplementary figures and uncropped blots as well as whole blots images

File name: Supplementary Data 1

Description: Numerical data summary
