# Supplementary material for: Pharmacological targeting of caspase-8/c-FLIPL heterodimer enhances complex II assembly and elimination of pancreatic cancer cells
Source: Commun Biol. 2025 Jan 3;8:4. doi: 10.1038/s42003-024-07409-6 (PMC11698904; doi:10.1038/s42003-024-07409-6)
Supplement: Supplementary file 4 — Reporting Summary [file 42003_2024_7409_MOESM4_ESM.pdf]

nature portfolio

Corresponding author(s): Inna Lavrik

Last updated by author(s): Dec 10, 2024

Reporting Summary

Nature Portfolio wishes to improve the reproducibility of the work that we publish. This form provides structure for consistency and transparency in reporting. For further information on Nature Portfolio policies, see our Editorial Policies and the Editorial Policy Checklist.

Statistics

For all statistical analyses, confirm that the following items are present in the figure legend, table legend, main text, or Methods section.

n/a	Confirmed	
☐	☒	The exact sample size (n) for each experimental group/condition, given as a discrete number and unit of measurement
☐	☒	A statement on whether measurements were taken from distinct samples or whether the same sample was measured repeatedly
☐	☒	The statistical test(s) used AND whether they are one- or two-sided<br>*Only common tests should be described solely by name; describe more complex techniques in the Methods section.*
☒	☐	A description of all covariates tested
☒	☐	A description of any assumptions or corrections, such as tests of normality and adjustment for multiple comparisons
☐	☒	A full description of the statistical parameters including central tendency (e.g. means) or other basic estimates (e.g. regression coefficient) AND variation (e.g. standard deviation) or associated estimates of uncertainty (e.g. confidence intervals)
☐	☒	For null hypothesis testing, the test statistic (e.g. F, t, r) with confidence intervals, effect sizes, degrees of freedom and P value noted<br>*Give P values as exact values whenever suitable.*
☒	☐	For Bayesian analysis, information on the choice of priors and Markov chain Monte Carlo settings
☒	☐	For hierarchical and complex designs, identification of the appropriate level for tests and full reporting of outcomes
☒	☐	Estimates of effect sizes (e.g. Cohen's d, Pearson's r), indicating how they were calculated

Our web collection on statistics for biologists contains articles on many of the points above.

Software and code

Policy information about availability of computer code

Data collection	Flow Sight, Image Lab, Unicorn
Data analysis	Excel, Image Lab, IDEAS 2.0

For manuscripts utilizing custom algorithms or software that are central to the research but not yet described in published literature, software must be made available to editors and reviewers. We strongly encourage code deposition in a community repository (e.g. GitHub). See the Nature Portfolio guidelines for submitting code & software for further information.

Data

Policy information about availability of data

All manuscripts must include a data availability statement. This statement should provide the following information, where applicable:

- Accession codes, unique identifiers, or web links for publicly available datasets
- A description of any restrictions on data availability
- For clinical datasets or third party data, please ensure that the statement adheres to our policy

A data availability statement is given in the manuscript

Research involving human participants, their data, or biological material

Policy information about studies with human participants or human data. See also policy information about sex, gender (identity/presentation), and sexual orientation and race, ethnicity and racism.

Reporting on sex and gender	No primary samples were used
Reporting on race, ethnicity, or other socially relevant groupings	No primary samples were used
Population characteristics	No primary samples were used
Recruitment	No primary samples were used
Ethics oversight	No primary samples were used

Note that full information on the approval of the study protocol must also be provided in the manuscript.

Field-specific reporting

Please select the one below that is the best fit for your research. If you are not sure, read the appropriate sections before making your selection.

☒ Life sciences ☐ Behavioural & social sciences ☐ Ecological, evolutionary & environmental sciences

For a reference copy of the document with all sections, see nature.com/documents/nr-reporting-summary-flat.pdf

Life sciences study design

All studies must disclose on these points even when the disclosure is negative.

Sample size	For Assays the work was done in duplicates as technical replications. Every experiment was done three times independently. For Assays 12 000 cells were used per one condition, for flow cytometry 15 000 cells were counted, Western Blot was done with 1x10^6 cells, Immunoprecipitations were carried out with 8x10^6 cells
Data exclusions	No data was excluded
Replication	Experiments were done three times
Randomization	Cells were randomly seeded in plates or wells. Experiments were done with cells of different passages at different days.
Blinding	This was not relevant to our studies

Reporting for specific materials, systems and methods

We require information from authors about some types of materials, experimental systems and methods used in many studies. Here, indicate whether each material, system or method listed is relevant to your study. If you are not sure if a list item applies to your research, read the appropriate section before selecting a response.

Materials & experimental systems

n/a	Involved in the study	
☐	☒	Antibodies
☐	☒	Eukaryotic cell lines
☒	☐	Palaeontology and archaeology
☒	☐	Animals and other organisms
☒	☐	Clinical data
☒	☐	Dual use research of concern
☒	☐	Plants

Methods

n/a	Involved in the study	
☒	☐	ChIP-seq
☐	☒	Flow cytometry
☒	☐	MRI-based neuroimaging

Antibodies

Antibodies used	polyclonal anti-actin antibody (A2103) from Sigma-Aldrich, Germany; monoclonal anti-Bak antibody (#12105), monoclonal anti-Bax antibody (#5023), polyclonal anti-Bid antibody (#2002), polyclonal anti-caspase-3 antibody (#9662), monoclonal anti-DR4 antibody

(#42533), monoclonal anti-DR5 antibody (#8074), monoclonal anti-LC3B antibody (#3868), monoclonal anti-RIPK1 XP antibody (#3493), polyclonal anti-PARP antibody (#9542), monoclonal phospho-RIPK3 (Ser227) (#93654), monoclonal MLKL (#14933), from Cell Signaling Technology, USA; monoclonal anti-Bcl-2 antibody (sc-7382), polyclonal anti-CD95 antibody (sc-715), polyclonal anti-Mcl-1 antibody (sc-819) from Santa Cruz, USA; monoclonal anti-Bcl-x antibody (610209) from BD Transduction Laboratories, USA; monoclonal anti-caspase-10 antibody (M059-3) from MBL International Corporation, USA; monoclonal anti-pMLKL (phospho S358) (ab187091), polyclonal anti-RIPK3 (ab226297) from abcam, UK; monoclonal anti-FADD antibody (clone 1C4), monoclonal anti-caspase-8 antibody (clone C15) and monoclonal c-FLIP antibody (clone NF6). Horseradish peroxidase-conjugated goat anti-mouse IgG1,-2b and goat anti-rabbit were from Southern Biotech, USA

Validation

Antibody Validation for Western Blotting. First of all, all antibodies used in our study were reported in the previous publications. Second, most of our antibodies were from Cell Signaling Technology (CST). CST provides the highest quality primary and secondary antibodies available for western blotting. CST™ antibodies are produced in-house and validated extensively according to a rigorous protocol.
Validation Steps Include:
-Examination of several cell lines and/or tissues of known expression levels allows accurate determination of species cross-reactivity and verifies specificity.
- Treatment of cell lines with growth factors, chemical activators or inhibitors, which induce or inhibit target expression, verifies specificity. Phosphatase treatment confirms phospho-specificity.
- The use of siRNA transfection or knockout cell lines verifies target specificity.
- Side-by-side comparison of lots to ensures lot-to-lot consistency.
- Optimal dilutions and buffers are predetermined, positive and negative cell extracts are specified, and detailed protocols are already optimized, saving valuable time and reagents.

Third, The specificity of the following Antibodies: anti-FADD, anti-Caspase-8, anti-Parp1, anti-c-FLIP, anti-RIPK3 and anti-RIPK1 were addionally validated by knockouts in our lab.

Fourth, CD95 antibody was validated by CD95 overexpression in our lab

Eukaryotic cell lines

Policy information about cell lines and Sex and Gender in Research

Cell line source(s)

SUIT-020 and MiaPaca2 cells were the kind gift of Prof. N. Giese (University of Heidelberg); HT29 cells were the kind gift of Prof. Thomas Brunner (University of Konstanz). Panc89 cells and primary fibroblasts were from National Center for Tumor Diseases (TU-Dresden).

Authentication

Cell lines were authenticated by the provider.

Mycoplasma contamination

The testing for mycoplasma were performed once in four weeks.

Commonly misidentified lines (See ICLAC register)

N/A

Plants

Seed stocks

Report on the source of all seed stocks or other plant material used. If applicable, state the seed stock centre and catalogue number. If plant specimens were collected from the field, describe the collection location, date and sampling procedures.

Novel plant genotypes

Describe the methods by which all novel plant genotypes were produced. This includes those generated by transgenic approaches, gene editing, chemical/radiation-based mutagenesis and hybridization. For transgenic lines, describe the transformation method, the number of independent lines analyzed and the generation upon which experiments were performed. For gene-edited lines, describe the editor used, the endogenous sequence targeted for editing, the targeting guide RNA sequence (if applicable) and how the editor was applied.

Authentication

Describe any authentication procedures for each seed stock used or novel genotype generated. Describe any experiments used to assess the effect of a mutation and, where applicable, how potential secondary effects (e.g. second site T-DNA insertions, mosiacism, off-target gene editing) were examined.

Flow Cytometry

Plots

Confirm that:

☒ The axis labels state the marker and fluorochrome used (e.g. CD4-FITC).

☒ The axis scales are clearly visible. Include numbers along axes only for bottom left plot of group (a 'group' is an analysis of identical markers).

☒ All plots are contour plots with outliers or pseudocolor plots.

☒ A numerical value for number of cells or percentage (with statistics) is provided.

Methodology

Sample preparation	1 x 10^6 SUIT-020 cells were stained with PE anti-human TRAIL-R1 or 2 antibody or FITC anti-human CD95 antibody (BioLegend, USA) according to manufacturer's instructions.<br><br>For cell death measurements 0.2 x 10^6 SUIT-020 cells or 0.5 x 10^6 Panc89 cells were seeded the day before stimulation in 6-Well plates. Next day medium was discarded and fresh medium was added to the cells. Cell were treated. After stimulation time cells were harvested using Trypsin and all cells including medium was transferred in a tube. The tube was centrifuged at 500 x g for 5 min at 4 °C. Cells were washed two times with 1 ml PBS. Cells were stained with Annexin/PI for 15 min in the dark at RT with Binding Buffer containing calcium. Cells were centrifuged and 50 µl Binding Buffer was added to the cells. Cells were measured (Hillert, Bettermann-Bethge et al., 2019, Pietkiewicz et al., 2015b).
Instrument	AMNIS (Cytek)
Software	Flow Sight, IDEAS 2.0
Cell population abundance	15 000 cells were collected. For analyzation more than 11 000 cells were used which were focussed single cells.
Gating strategy	Populations were separated in viable (negative), Annexin-V-FITC positive (single positive) and Annexin V-PI-Positive (double positive) cells. Gating was done by checking the microscopic pictures of the cells (imaging flow cytometry)<br>For receptor staining: Histograms of control cells and cells stained with the receptor antibody were compared and analyzed.

☒ Tick this box to confirm that a figure exemplifying the gating strategy is provided in the Supplementary Information.
